# AI-driven and automated MRI sequence optimization in scanner-independent MRI sequences formulated by a domain-specific language

**DOI:** 10.3389/fnimg.2023.1090054

**Published:** 2023-05-12

**Authors:** Daniel Christopher Hoinkiss, Jörn Huber, Christina Plump, Christoph Lüth, Rolf Drechsler, Matthias Günther

**Affiliations:** ^1^Fraunhofer Institute for Digital Medicine MEVIS, Imaging Physics, Bremen, Germany; ^2^German Research Center for Artificial Intelligence, Cyber-Physical Systems, Bremen, Germany; ^3^Faculty 3 - Mathematics and Computer Science, University of Bremen, Bremen, Germany; ^4^Faculty 1 - Physics/Electrical Engineering, University of Bremen, Bremen, Germany

**Keywords:** domain specific language, magnetic resonance imaging, machine learning, simulation, optimization, automation, evolutionary algorithms

## Abstract

**Introduction:**

The complexity of Magnetic Resonance Imaging (MRI) sequences requires expert knowledge about the underlying contrast mechanisms to select from the wide range of available applications and protocols. Automation of this process using machine learning (ML) can support the radiologists and MR technicians by complementing their experience and finding the optimal MRI sequence and protocol for certain applications.

**Methods:**

We define domain-specific languages (DSL) both for describing MRI sequences and for formulating clinical demands for sequence optimization. By using various abstraction levels, we allow different key users exact definitions of MRI sequences and make them more accessible to ML. We use a vendor-independent MRI framework (gammaSTAR) to build sequences that are formulated by the DSL and export them using the generic file format introduced by the Pulseq framework, making it possible to simulate phantom data using the open-source MR simulation framework JEMRIS to build a training database that relates input MRI sequences to output sets of metrics. Utilizing ML techniques, we learn this correspondence to allow efficient optimization of MRI sequences meeting the clinical demands formulated as a starting point.

**Results:**

ML methods are capable of capturing the relation of input and simulated output parameters. Evolutionary algorithms show promising results in finding optimal MRI sequences with regards to the training data. Simulated and acquired MRI data show high correspondence to the initial set of requirements.

**Discussion:**

This work has the potential to offer optimal solutions for different clinical scenarios, potentially reducing exam times by preventing suboptimal MRI protocol settings. Future work needs to cover additional DSL layers of higher flexibility as well as an optimization of the underlying MRI simulation process together with an extension of the optimization method.

## 1. Introduction

Magnetic Resonance Imaging (MRI) is an indispensable part of modern medical diagnostics. In contrast to, e.g., Computed Tomography it features high versatility concerning the generation of different tissue contrasts using different sequences of RF and magnetic field gradient events. This high flexibility however requires excellent knowledge from radiologists and MR technicians to generate the desired image contrasts, which are needed for a certain diagnosis. An automated optimization strategy could help to find the optimal sequence and protocol for a certain application, reducing the entry hurdle for untrained MR operators. Machine Learning (ML) strategies can be a strong foundation to achieve this goal. During the last few years, ML strategies have been applied to several areas in the MRI toolchain. Most prominently used in image reconstruction (Pal and Rathi, 2022) and post-processing (Radke et al., 2021), the image acquisition got increasing attention in terms of scan acceleration (Bahadir et al., 2020; Sherry et al., 2020; Zeng et al., 2021) and low-level sequence optimization in terms of rf and gradient hardware events (Shinnar et al., 1989; Lustig et al., 2008; Loktyushin et al., 2021) as well as portability (Greer, 2020). Intelligent protocolling using look-up tables has also been proposed (Ravi and Geethanath, 2020; Ravi et al., 2022) in addition to protocol optimization of specific MR sequences based on eigenimage filtering (Soltanian-Zadeh et al., 1994) and real-time adaption of MR sequences using operator requests or automatic feedback (Santos et al., 2004). However, optimization strategies targeting MR sequences formulated on different levels of complexity have yet to be proposed. This might be caused by the fact that the MRI parameter space is not very well-defined in general.

In this paper, we use Domain Specific Languages (DSL, Mernik et al., 2005) to help formulating MRI sequences on various abstraction levels, making it easier to switch between different layers of complexity. This common language resembles the typical patterns and regularities of MRI sequences and allows the exact definition of optimizable parameters, enabling approximation of the relation between the latter and calculated image metrics. Thus, the accessibility of MRI sequences is increased to non-experts as well as machine learning and AI. Eventually, this will allow the automated generation of MRI sequences for optimized image quality given specific requirements for the scan.

A second hurdle when discussing sequence optimization strategies is the limited accessibility of MRI sequences and their programming environments. RF and gradient pulses can be optimized externally and loaded into the sequence with ease. However, defining the structure and components of the sequence needs low-level C++ coding in complex, platform-specific environments requiring long-time experience and expert knowledge (i.e., IDEA by Siemens Healthineers, MR-Paradise by Philips Healthcare or EPIC by GE Healthcare). During the last years, platform-independent alternatives have attracted attention (Jochimsen and von Mengershausen, 2004; Layton et al., 2016; Magland et al., 2016; Nielsen and Noll, 2018; Ravi et al., 2019; Cordes et al., 2020). In this work, we use the gammaSTAR framework that offers the flexibility to build add-on solutions to support the user in generating MRI sequences on a modular level (Cordes et al., 2020). We use it as part of our sequence optimization strategy for easy conversion between DSL and the technical, ready-to-use MRI sequence. This approach allows practitioners as well as researchers to efficiently describe MRI sequences with no knowledge of programming languages without losing the chance of defining low-level details.

This work demonstrates (1) the definition and implementation of a domain-specific language to simplify the specification of MRI sequences for non-experts, (2) the simulation and acquisition of these MRI sequences (3), and an ML-based strategy to create the optimal MRI sequence for a given set of requirements based on simulated training data.

## 2. Materials and methods

**Figure 1:**
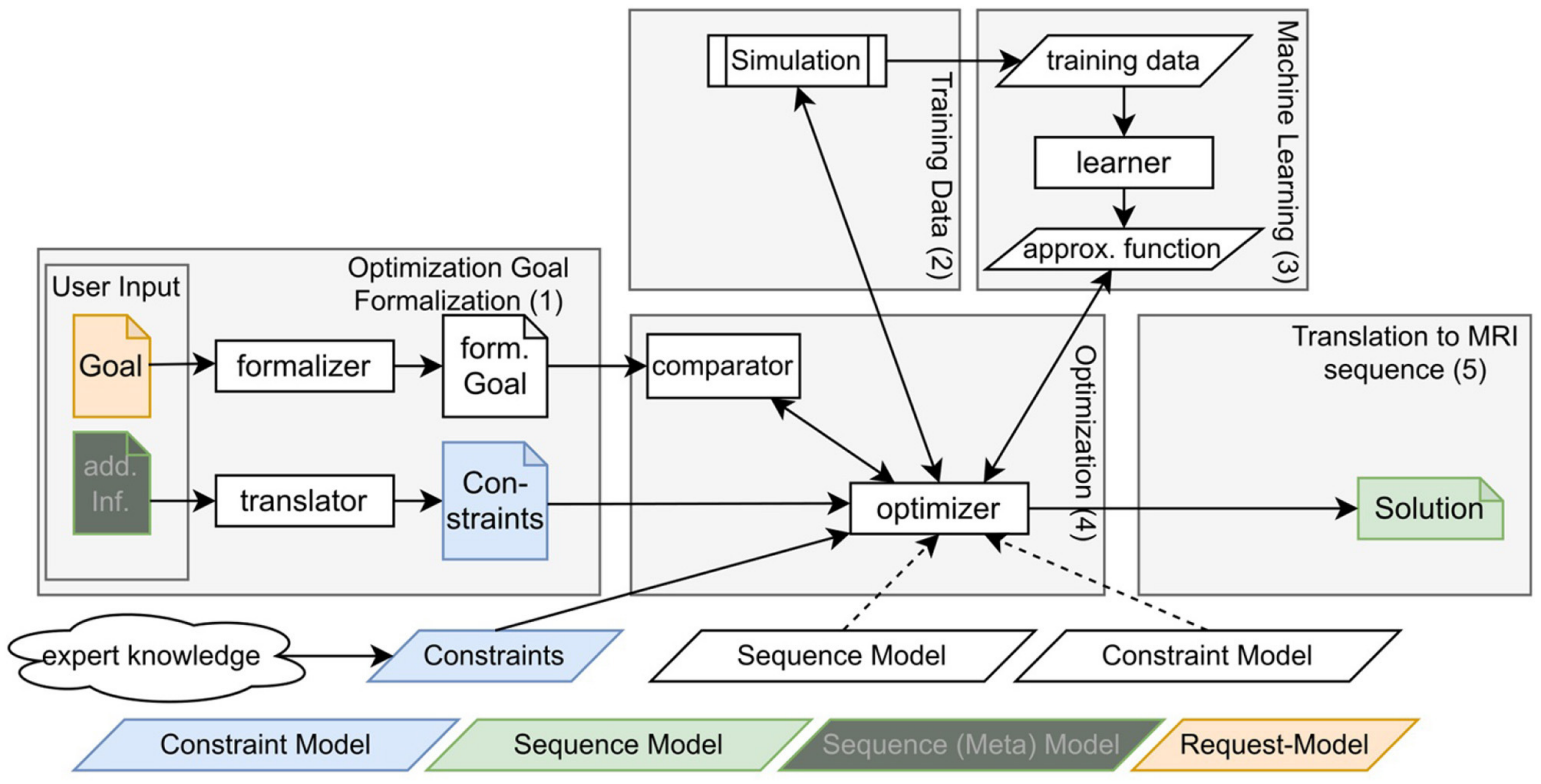
Schematic depiction of the overall flow of the proposed approach.

This section introduces the overall components presented in this paper which are summarized in Figure 1.

The workflow can be separated into five main components: First, there is the formulation of the goal and additional information like goal-specific constraints [see denotation (1) in the Figure 1]. Typical goals are the maximization of signal-to-noise ratios (SNR) or specific tissue contrasts. Goal-specific constraints could, e.g., be the avoidance of ghosting or restrictions in conventional protocol parameters like matrix size or acquisition time. This information must be formulated in a way that it can be interpreted by a program but is still usable for non-programmers. Section 2.1 explains our approach for this component.

To allow efficient sequence optimization, two further components deal with the approximation of simulated MRI results, meaning the correspondence between sequence and resulting image properties. To that end, training data has been collected with simulations [see denotation (2) in the Figure 1] and for every defined image property, an ML-model has been trained [see denotation (3) in the Figure 1]. Sections 2.2 and 2.3 explain this process in more detail.

Fourth, the optimization takes the formalized goal and potential constraints as input and makes use of the constructed ML-model to find a sequence that produces image properties that are as close as possible to the desired goal [see denotation (4) in the Figure 1]. Section 2.4 focuses on this component.

The fifth component is the transformation of a solution of the optimization algorithm into a sequence formulation that is understandable for the non-programmer and non-expert. This aims—similar to the first component—at the definition of a domain-specific language, however, this time for the sequences itself [see denotation (5) in the Figure 1]. Section 2.5 focuses on this component.

### 2.1. Formulation of requirements for sequence optimization

This section explains the formalization of optimization goals as well as additional constraints for this optimization. It has two important usability features to uphold: First, it needs to be easily accessible, i.e., to write for clinical personnel, and second, it needs to be able to be correctly interpreted by the final systems. While the last usability feature suggests the usage of a programming language, the first one strongly argues against it. A compromise is the usage of a domain-specific language. It has precision in the interpretation of a programming language, however, when specified well, it can be very close to natural language and equipped with assistance for the correct syntax. A domain-specific language follows predefined grammar, just like a natural language does. However, it is usually defined more precisely. The definition of grammar determines what expressiveness the language has and at the same time ensures an error-free understanding of the information encoded in the goals or constraints formulated in this language.

The grammar follows a scheme of substitution rules. For each grammatical term (non-terminal in DSL terms), it is specified how it can be substituted. This can either be with other grammatical terms or with final expressions (terminal in DSL terms). For example, in our natural language, a sentence (usually) consists of a subject, a verb, and an object. The subject itself can be a noun with a determiner, a noun with a determiner and an adjective but also a pronoun. The verb can also be substituted with complexer constructions. Additionally, there might be optional expressions of time or place at the end of the sentence.^1^ The grammar may then contain the following rules:


<Sentence>     ::= <Subject> <Verb> <Object> [<ExprOfPlace][<ExprOfTime>]
<Subject>       ::= <Determiner> [<Adjective>] <Noun> | <Pronoun>
<Verb>          ::= make|fly|do|eat|say|speak ..
<Determiner>   ::= the|a|an|some|any|...


The third and fourth rules both contain terminals on their right-hand-side because they are substituted with "real" words. In the end, a *sentence* must only contain terminals and each terminal must have been reached through a series of substitutions. For programming languages, there is the concept of keywords. They help the reader (i.e., the parser) in understanding what comes next. Examples are the keywords *if* —starting an if-clause, and *for*—starting a loop in most standard programming languages. To define a DSL, it is first important to get an overview of the sentences that are required to be expressed. Step-by-step, they can be formalized through the above-described substitutions.

Some exemplary goals for an MRI sequence accompanied by constraints given by domain experts are given below:

- *Maximize contrast between gray and white matter. Avoid ghosting artifacts. Allow some distortion and motion. Aim for an overall high SNR with a special focus on gray matter*.
- *Maximize SNR in CSF. Only allow sharp images. Avoid all types of artifacts and aim for high contrast between CSF and gray/white matter*.

Looking at these formulations, a division between optimization terms (minimize/maximize), strict constraints (avoid ghosting artifacts, only allow sharp images) and vague constraints (allow some distortion, aim for high contrast) seems logical. This already provides the building blocks for the DSL: A text in this DSL consists of three blocks (where the latter two are optional): Optimization goal, strict constraints, and vague constraints. The optimization goals consist of the acquisition metric (for a list, see the next Section 2.2) and the optimization direction, i.e., minimization or maximization. The strict constraints block starts with *obey the following constraints:* and allows inequalities and equalities on acquisition metrics or sequence parameters. The vague constraints' block starts with *aim for the following:* and may contain *allowance, avoidance*, and *ordinal statements* with categories from *very low* to *very high* that describe an acquisition metric. Furthermore, a *basic* sequence can be given that the optimization algorithm is supposed to uphold.

An informal description of the DSL to describe MRI sequence demands is given below. The DSL describing the actual MRI sequence (*startingSequence*) is explained in Section 2.5). Brackets denote optional parts of the rules, the pipe denotes the logical “or”. If a part is printed bold, this means that it can be replaced with a special set of values, e.g., **AcquisitionMetric** can be replaced by all image metrics and the acquisition time.


<Requirement>           ::= <OptimizationGoal> [<StrictConstraint>]
                            [<VagueConstraint>] [<startingSequence>]
<OptimizationGoal>      ::= <OptimizationDirection> **AcquisitionMetric**
<OptimizationDirection> ::= "Minimize" | "Maximize"
<StrictConstraint>      ::= (**AcquisitionMetric**|**SequenceParam**)
                            <RelationIdentifier>
                            ((**AcquisitionMetric**|**SequenceParam**)|**Numerical**)
<RelationIdentifier>    ::= "is smaller than"|"equals"|"is greater than"
<VagueConstraint>       ::= <Avoidance>| <Allowance> | <Aim>
<Allowance>             ::= "Allow" **AcquisitionMetric**
<Avoidance>             ::= "Avoid" **AcquisitionMetric**
<Aim>                   ::= "Have" <Quantifier> **AcquisitionMetric**
<Quantifier>            ::= "very low" | "low" | "decent" |
                            "high" | "very high"


The above-mentioned exemplary goals can then be expressed as follows:

Listing 1 **DSL formulation of the first above mentioned goal.**


define optimization requirement :
  maximize "GWC".
  obey the following constraints:
    "SNR_GM" is higher than 30.
    "SNR_WM" is higher than 30.
    "SNR_GM" is higher than "SNR_WM".
  aim for the following :		
    avoid "ghosting".

Listing 2 **DSL formulation of the second above mentioned goal.**


define optimization requirement :
  maximize "SNR_CSF".
  obey the following constraints:
   "Sharpness" is higher than 0.85.
  aim for the following :
    avoid "ghosting".
    avoid "motion".
    avoid "distortion".
    have high "CGC".
    have high "CWC".

Of course, these formulations look less verbose than the above-mentioned examples. However, they capture many of the above-mentioned formulations and requirements and are—at the same time—parsable by a program and thus a practicable input to an automated optimization flow.

### 2.2. Generation of training data for ML-based optimization of MRI sequences

**Figure 2:**
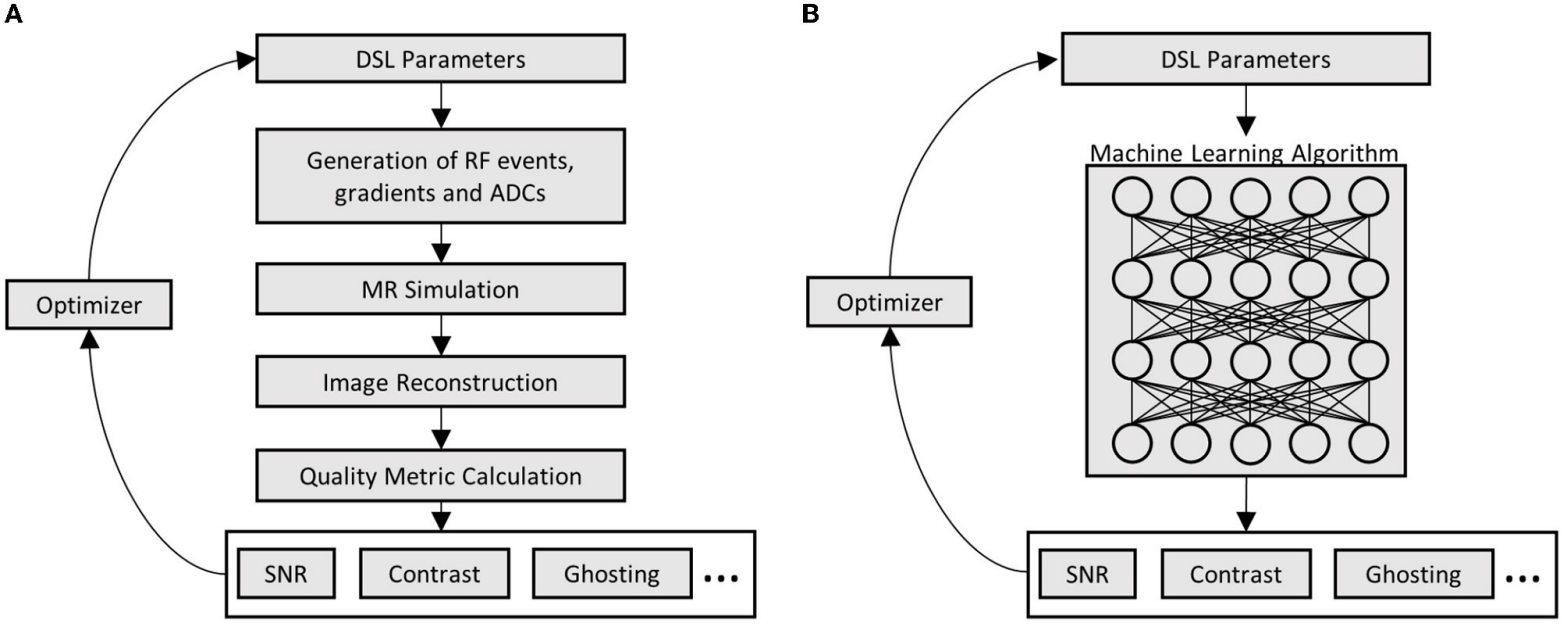
Comparison of conventional and machine learning-based metric optimization. **(A)** Iterative optimization of MRI sequences using MRI simulations; and **(B)** Iterative optimization using machine learning algorithms which learned the relation between DSL and image quality parameters.

**Figure 3:**
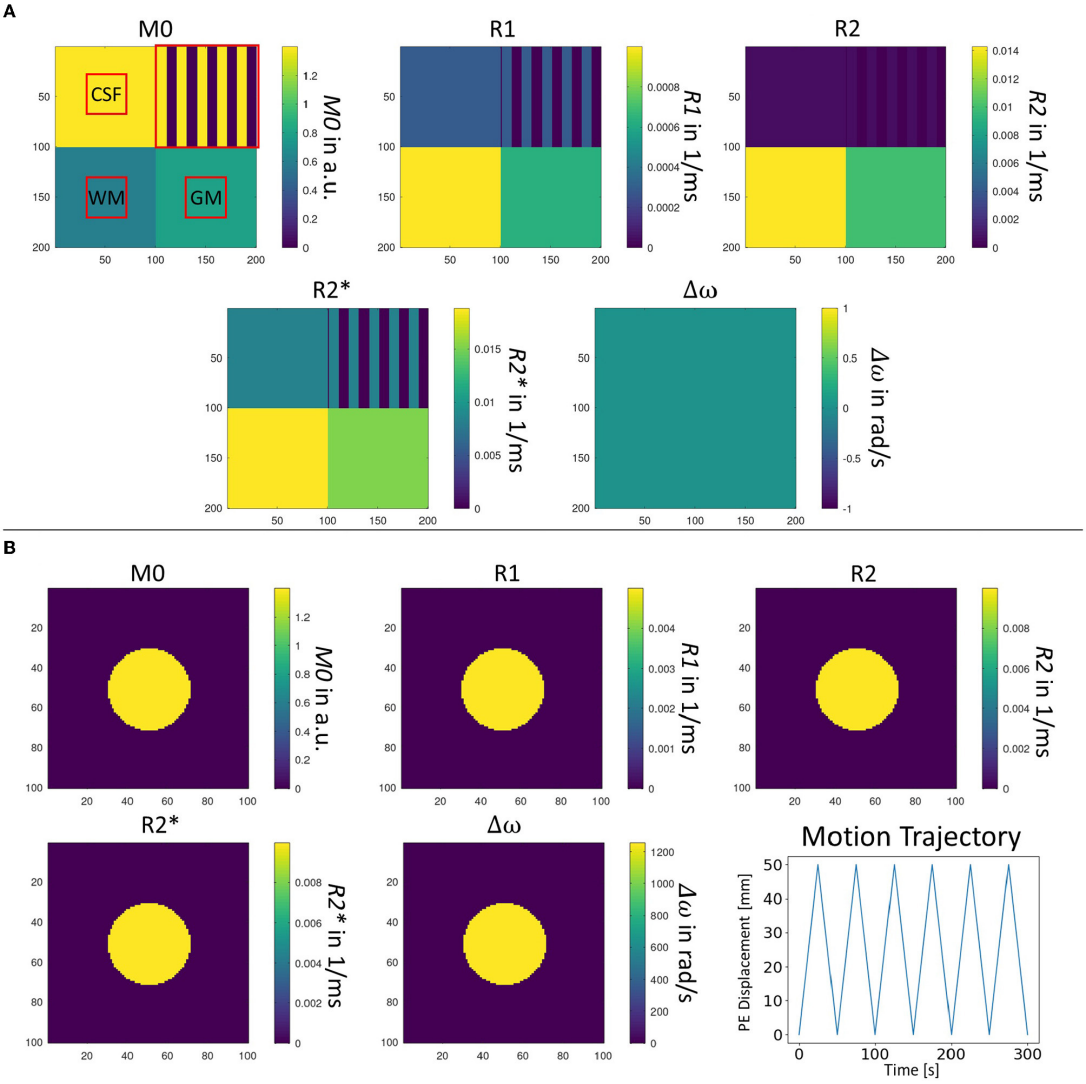
Two different digital phantoms used for evaluation of the image metrics. **(A)** Parameters of the digital phantom used for evaluation of contrast, SNR, ghosting, image sharpness as well as homogeneity metrics. Red squares indicate masks which were used to identify respective tissue regions in reconstructed MRI images; and **(B)** Parameters of the digital phantom used for evaluation of the motion and distortion sensitivity of MRI sequences. A continuous translational pattern in the phase-encoding direction is applied to assess the sensitivity to motion of the MRI sequence.

The previous section already introduced various image metrics such as tissue contrast, SNR or robustness against the subject motion, ghosting and geometric distortion which are typical parameters guiding the selection of a specific MRI sequence in clinical practice. Concerning these demands, automatic optimization of MRI sequences would require time-consuming MRI simulation and metric calculation routines (see Figure 2A). In the case of complex MRI sequences, such optimization can take several hours which is unsuitable for clinical practice. Therefore, an obvious approach is to approximate the relation between MRI sequences and chosen image quality metrics by machine learning techniques. This overcomes the need for the time-consuming parts in the optimization routine (see Figure 2B) but requires a sufficient amount of training data. In this work, those data are generated using two digital phantoms. These phantoms are described in the following section and illustrated in Figure 3.

#### 2.2.1. MRI phantom design for training data generation

**Figure 4:**
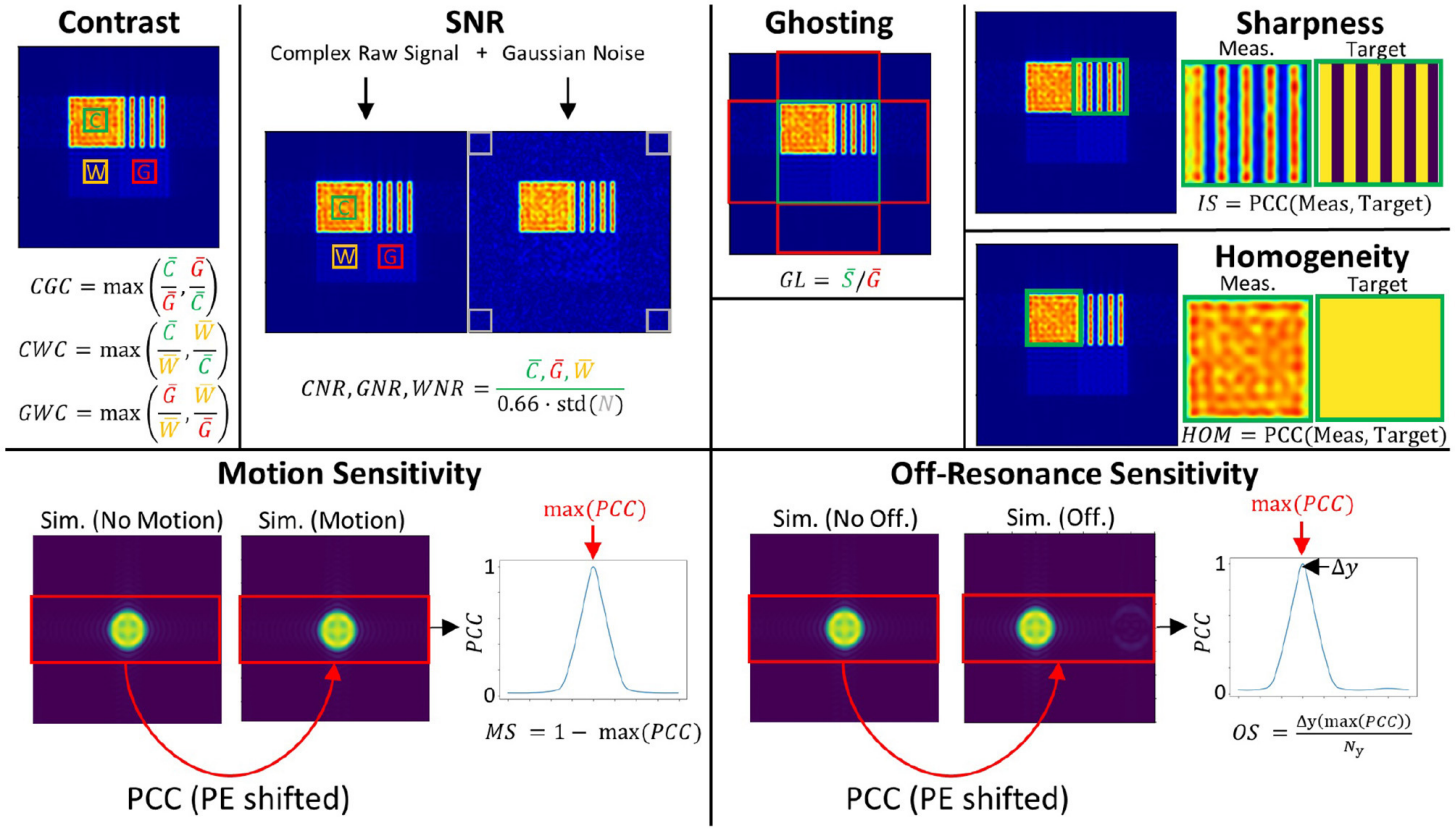
Calculation of image metrics. PCC refers to the Pearson Correlation Coefficient.

The first design (see Figure 3A) uses a squared geometry where tissue parameters of the different sub-regions are chosen to correspond to those of cerebrospinal fluid (CSF), white matter (WM) and gray matter (GM) as typically encountered in brain imaging (Bojorquez et al., 2017). In addition, a structural CSF section is added for evaluating structures after image reconstruction. Overall dimensions of the phantom are 100 × 100 mm^2^ with a simulated spin density of 4/mm^2^. It allows the calculation of contrasts between the three types of simulated tissue, SNR, levels of ghosting artifacts, the sharpness of reconstructed images as well as the homogeneity of signal in reconstructed images, as shown in the top row of Figure 4. The different tissue contrasts (CGC, GWC, CWC) are calculated by dividing the mean signal intensity in tissue areas as defined in the square phantom. Tissue-specific SNR levels (GNR, CNR, WNR) are calculated by adding additional Gaussian noise to the simulated complex MRI raw data, followed by the usual approach for SNR calculation in the context of MRI images (Dietrich et al., 2007). The ghosting level (GL) is calculated by dividing the target signal in the defined phantom region by the ghosting signal in the defined ghosting regions. Image sharpness (IS) is assessed by calculating the Pearson Correlation Coefficient (PCC) between the reconstructed structure area and the simulated target structure. A similar approach is taken to calculate a homogeneity measure (HOM) in the filled CSF area. This yields a general statement about the quality of the image itself which cannot be derived by contrast and/or SNR measures alone.

A second phantom geometry is used to assess the sensitivity to motion and geometric distortions (see Figure 3B). A small circular structure is designed to reduce the number of spins and thus speed up the simulation. Dimensions of the given area are 100 × 100 mm^2^ with a simulated spin density of 2/mm^2^. In contrast to the squared phantom, a non-zero off-resonance value is chosen. A repeating motion pattern can be simulated, corresponding to linear translational shifts in the phase-encoding direction during the simulation process (see bottom right). Motion sensitivity can be assessed by applying a series of linearly increasing one-dimensional shifts in the phase-encoding direction to the reconstructed motion image (see Figure 4, bottom row). For each shift the Pearson Correlation Coefficient (PCC) is estimated in the indicated area. Finally, the maximum PCC is calculated and subtracted from 1 to define the motion sensitivity measure. This procedure is chosen because a general shift of the object would be unproblematic as long as the quality of the reconstructed image is not affected. Off-resonance sensitivity is calculated in a quite similar way. However, in contrast to motion sensitivity, image distortion is directly linked to the shift of the object in image space. Therefore, the shifted position of the maximum PCC value is assessed and divided by the overall size of the interpolated image matrix in the phase-encoding direction which yields the off-resonance sensitivity measure.

#### 2.2.2. Data generation workflow

**Figure 5:**
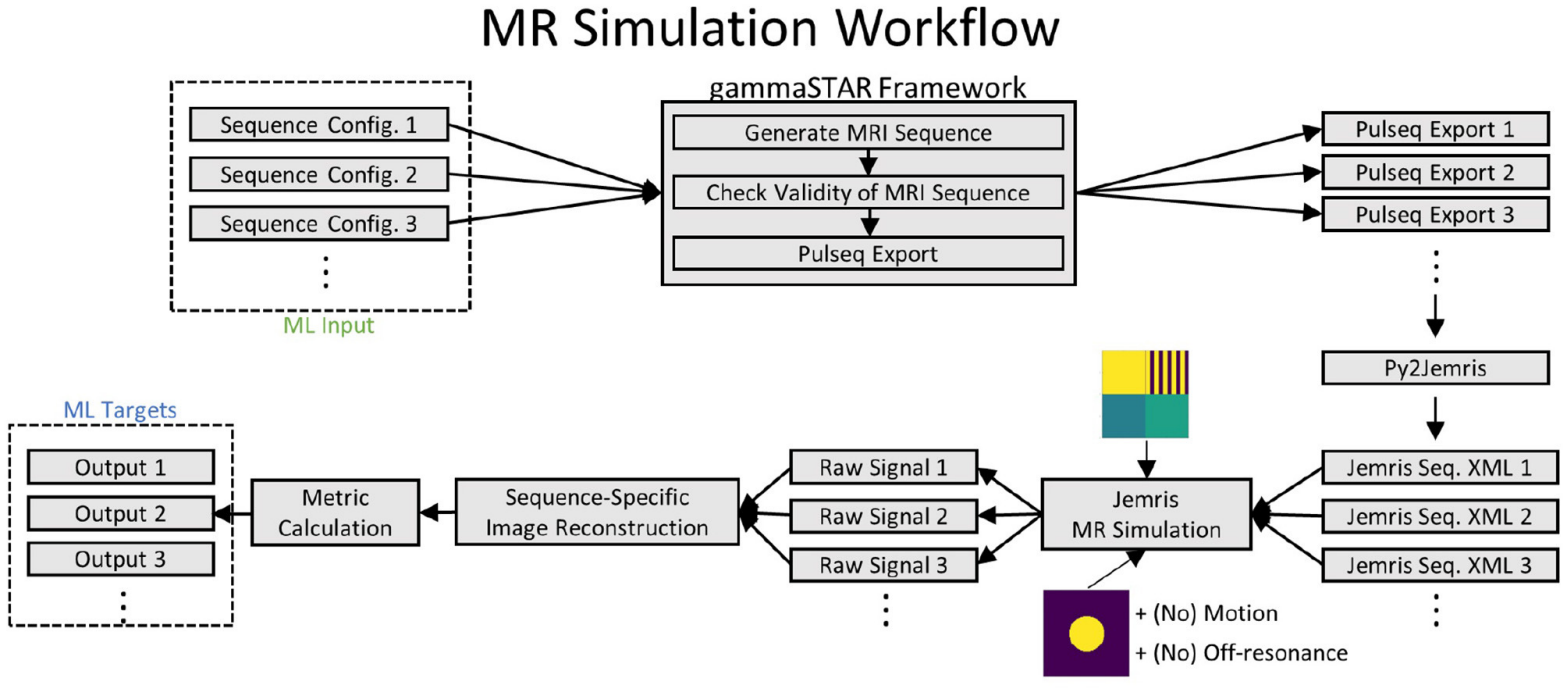
Workflow of MRI simulations. Note that simulations using the circular phantom are performed with and without adding off-resonance or motion effects.

Using both digital phantoms, training data are finally generated in a process as shown in Figure 5. The initial input are several sequence configuration files with given MRI sequence representations as they can be formulated by the DSL. Corresponding radio frequency (RF), gradient and ADC events are generated using the gammaSTAR framework (Cordes et al., 2020) subsequently. The integration of gammaSTAR has several benefits. First, the actual MR logic, which translates the DSL sequence into hardware events is separated from the DSL formulation, reducing the latter to its core functionality. Second, gammaSTAR also allows the execution of generated MR sequences on MR systems of different vendors, making generated sequences widely available. A validity check is performed for each configured gammaSTAR sequence to ensure the physical plausibility of generated hardware events. Afterwards, valid MRI sequences are exported into the Pulseq format (Layton et al., 2016) because a direct JEMRIS export is not available to date. Exported sequences are subsequently processed by the py2Jemris toolbox (Tong et al., 2021) to generate JEMRIS sequence files. Raw MRI signal is then simulated in JEMRIS (Stocker et al., 2010) in combination with one of the predesigned digital phantoms. In total, four simulations are carried out for each generated sequence:

1. Square phantom
2. Circular phantom, without off-resonance, without motion
3. Circular phantom, with off-resonance, without motion
4. Circular phantom, without off-resonance, with motion.

Generated raw data are then reconstructed using sequence-specific image reconstruction routines implemented in Python. Here, the absolute value of the complex MRI signal is calculated and images are interpolated to a pixel size of 1 × 1 mm^2^. Note that a homogeneous coil sensitivity is assumed during the simulation procedure. Afterwards, image quality parameters are calculated and stored for further processing. The interested reader is referred to the Supplementary material for further insights into the implemented reconstruction routines.

### 2.3. Training the ML-model

Training the actual ML-model is an important step toward the usability of our approach. Without a correct approximation of the simulation, the optimization process can not evaluate possible solutions from the search space and is therefore incapable of comparing different solutions with respect to the specified goal. The data used for training the ML model are the sequence configuration files (see Figure 5, green) and the calculated acquisition metrics (see Figure 5, blue).

The function to be trained has a twelve-dimensional input space (see Table 1) and a 12-dimensional output space(see Section 2.2.1 for output variables). Some variables in the configuration files are categorical (e.g., ReadoutType and EchoType) and not numerical, which makes their usage challenging for standard machine-learning techniques. However, the information in these variables can also be found by using or combining other input variables (the information for the readout type is already in the refocussing angle, for example), therefore, they can be left out of the construction of the model (see Table 2) leaving only numerical variables.

**Table 1:** DSL parameters used for generation of training data.

**Sequence name**	**EPI**	**SE EPI**	**bSSFP**	**RARE**
Echo type	Gradient echo	Spin echo	Gradient echo	Spin echo
Readout type	EPI	EPI	Line readout	Line readout
Matrix	32, 64	32, 64	32, 64	32, 64
TE (ms)	10, 60, 110	10, 60, 110	2, 5, 8, 11, 14, 17	10, 60, 110, 160
TR (ms)	100, 1,500, 6,000	100, 1,500, 6,000	4, 12, 20, 28, 36	100, 1,500, 6,000
ETL	-	-	-	16, 32, 64
EPI factor	16, 32, 64	16, 32, 64	-	-
Readout duration (ms)	0.4, 0.5	0.4, 0.5	2	1, 1.1
Excitation angle (°)	90	90	90	90
Refocussing angle (°)	-	180	-	180
Measurements	1, 10	1, 10	1, 2	1, 10
Number prescans	-	-	8	-

Underlined parameters are used for generation of sequence diagrams in Figure 7.

**Table 2:** Results of principal component analysis for input and output data.

	**PC1**	**PC2**	**PC3**	**PC4**	**PC5**	**PC6**	**PC7**	**PC8**	**PC9**	**PC10**	**PC11**	**PC12**
**Input**												
Std. deviation	2.22	1.5	1.20	0.88	0.77	0.63	0.59	0.5	0.18	0.05	0.00	-
Proportion	0.45	0.20	0.13	0.07	0.05	0.04	0.03	0.02	0.00	0.00	0.00	-
Cum. prop.	0.45	0.65	0.78	0.85	0.91	0.94	0.97	1.0	1.0	1.0	1.0	-
**Output**												
Std. deviation	1.97	1.44	1.32	1.18	0.92	0.89	0.72	0.65	0.44	0.33	0.11	0.07
Proportion	0.32	0.17	0.15	0.12	0.07	0.07	0.04	0.04	0.02	0.01	0.0	0.0
Cum. prop.	0.32	0.5	0.64	0.76	0.83	0.90	0.94	0.97	0.99	1.0	1.0	1.0

**Figure 6:**
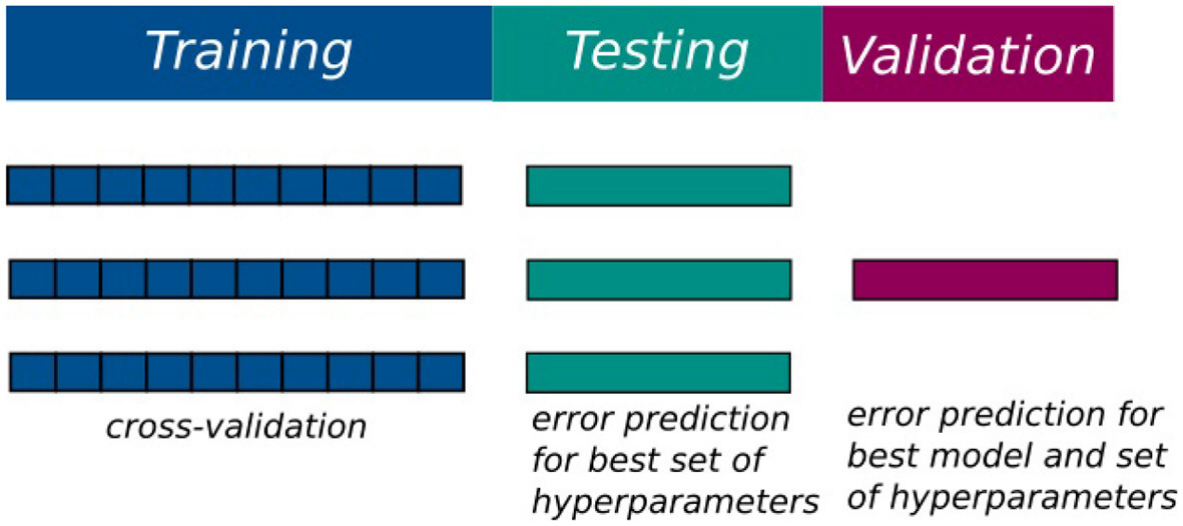
A schematic depiction of the data split based on the three-way-holdout principle.

Generated data is divided into training, testing and validation datasets, making up 60%, 20%, 20% of the training data respectively. This principle is based on the three-way-holdout principles to avoid selection and estimation biases that may occur when results on testing data are used for estimating error measures of the ML-model (Vabalas et al., 2019). Figure 6 depicts this schematically. The training data is used for identifying the best set of hyperparameters for each ML-model used. The evaluation for this tuning process is done via a 10-fold cross-validation (Hastie et al., 2009). The resulting best hyperparameter sets are tested with their respective model on the testing dataset. Based on these results, the best model is chosen and finally evaluated on the validation data set. This process is repeated for every output variable.

To reduce dimensionality and derive an independent search space, we performed a Principal-Component-Analysis (PCA, Pearson, 1901) on the input and output data. We show the results in Section 4.

Most ML-models need tuned hyperparameters to work effectively. We chose three different machine-learning techniques, namely support vector regression (Scholkopf and Smola, 2018), k-nearest neighbor (Fix and Hodges, 1989), and random forests (Ho, 1995) and performed a hyper tuning on their respective hyperparameters using a three-way-holdout principle. The best setting for each model was then tested on a new data set and based on this, we chose the best model for each output variable.

### 2.4. Optimization strategies for finding optimized MRI sequences

One specific goal of this contribution is the possibility for practitioners and MRI experts to optimize MRI sequences to their specified set of requirements at their level of expertise and abstraction. Additionally, the optimization process is supposed to be able to start with no sequence specified as well as an underlying sequence that specifies the general direction of the search. In Section 2.1, the formulation of optimization requirements has already been discussed. For an optimization, several aspects have to be considered to decide which general technique to be used: When dealing with well-defined, differentiable optimization functions, it is generally sensible to use gradient-based techniques to profit from the knowledge (there are, of course, exceptions, e.g., when the computation or evaluation of the derivatives is cost-intensive). When dealing with an optimization function that is not well-defined or not even continuous or when the search space has its atrocities, then the default are stochastic population-based approaches, e.g., evolutionary algorithms or particle swarm optimization. These approaches allow proceeding to a global optimum as they do not stop at a local optimum and, through their population-based approach, can obtain several local optima simultaneously.

In this work, we deal with two challenges simultaneously: The first one is the estimated optimization function through the ML-based approach. Usually, these functions are not differentiable, or—if they are—only in certain areas. The second one is the complex search space. There are several restrictions based on the search space to receive a valid sequence in the end. Additionally, there are different types of optimization requirements. Some of them are strict, others are weak to guide the search. Therefore, we decided to make use of evolutionary algorithms and extend them by gradient-based methods if the local search space area allows it.

In the context of evolutionary algorithms, several techniques have been developed to deal with approximate optimization functions (for a survey, see Jin, 2005) and also to include knowledge about the training data (see Plump et al., 2021b, 2022b). Additionally, for search spaces with dependencies, there are also techniques to adapt the recombination and mutation operators to include this (see Plump et al., 2021a). Furthermore, there is a wide variety of techniques available to deal with constraints (Mezura-Montes and Coello, 2006). Two techniques are to be pointed out here, as they will be applied: First, constraint violations can influence the fitness function as penalties. Thus, the evolutionary algorithm will exclude invalid solutions from the population due to their bad fitness. Second, there is the technique of repairing which is often done when data come from different scales (cardinal vs. categorical). Then, a proposed solution that may itself not be valid gets repaired toward one or two solutions that are valid and in a defined ϵ-neighborhood. For example, if three different options are encoded as cardinal values 1, 2, and 3, the result yields 2.7. This number can not be transferred back to a categorical value. However, rounding this up to 3 allows a retranslation to the third considered value.

As this is a potentially multi-objective optimization problem, it is necessary to keep the technique adaptable to a changing number of optimization goals. Please keep in mind that only goals specified as optimization goals in terms of the introduced domain-specific language are considered the optimization goal. The rest is taken care of via constraints. In Plump et al. (2022a), a technique is presented to equip constraints with different levels of strictness. In this work, we adopt this technique for the acquisition metrics.

### 2.5. Generating a domain-specific language to describe MRI sequences

A substantial part of this work is defining a domain-specific language that allows practitioners as well as experts to define MRI sequences at their level of abstraction. In this work, this is limited to two main levels, but can quickly be expanded to lower levels that allow the definition of MRI sequences at the most specific detail.

The upper level is defined as the most simplified expression of an MRI sequence, namely through its echo statement and its readout statement. The medium level, on the other hand, has (in this setup) the requirement of specifying all elements that were necessary to generate the described training data. However, it is not assumed that both languages are strictly separated. On the contrary, through the addition of *specifications* and *additions* an originally upper-level specification can progress toward a medium-level specification.


<Sequence>        ::= <upperSpec> [<Additions>] [<Specifications>]
<upperSpec>       ::= "define" **name** <Echo> <Readout>
<Addition>        ::= "Add" (<Spoiling> | <Prescans> )
<Specification>   ::= "Specify" (<EchoSpec>|<ReadoutSpec>|
                         <TimingSpec>| <TrajectorySpec> |
                        <Measurements> )
<EchoSpec>        ::= "echo" <ExcitationSpec> (<RefocussingSpec>)
<ExcitationSpec>  ::= "excitation" **pulseType angle**
<RefocussingSpec> ::= "refocussing" **pulseType angle**
<ReadoutSpec>     ::= "readout" **duration number-of-columns**
<TimingSpec>      ::= "timing parameters" **TE TR**
<TrajectorySpec>  ::= "trajectory" **Epi-Factor ETL number-of-rows**
<Measurements>    ::= "measurements" **count**


The rules illustrate the main principle of the DSL. The first sentence is necessary for the upper level, i.e., the *upperSpec*. This first sentence defines the name, echo and refocussing of the MRI sequence [see lines (1) and (2) in the Listings 3–6]. This constitutes the upper level. After that, several specifications can be made. First of all, the echo can have its excitation and refocussing pulse types and angles specified, whereas the refocussing specification is optional. Second, the readout can have the readout duration and the number of columns specified. Third, timing parameters like TE and TR can be specified. Fourth, the trajectory can be refined with the specification of the EPI-factor, the echo train length ETL, and the number of rows. Finally, the number of measurements/repetitions can be given. The original upper-level sentence together with these specifications then constitutes the medium level. Missing specifications are substituted with default values depending on echo type and refocussing type. Complementary to specifications, so-called additions can be made. These can define some spoiling or several prescans, for example. To enable the default values for the sequences that are defined on the upper level, the following four examples (see Listings 3–6) give the default values that will be used for the four types of sequences that were used for the setup of the training data.

Listing 3 **DSL formulation of a default RARE sequence.**


define MRI sequence "RARE-default"
  using a "SpinEcho" with "LineReadout".
Add gradient spoiling around refocussing
  of type balanced.
Add gradient spoiling after echotrain.
Specify echo
  as excitation
    with type sincpulse and angle 90,
  and refocussing
    with type sincpulse and angle 180.
Specify readout
  with duration 2000
  and number-of-columns 64.
Specify timing parameters
  with TE 44
  and TR 500.
Specify trajectory
  with Epi-Factor 1
  and ETL 8
  and number-of-rows 64.
Specify measurements
  with count 1.

Listing 4 **DSL formulation of a default SE-EPI sequence.**


define MRI sequence "SE-EPI-default"
  using a "SpinEcho" with "EPIReadout".
Specify echo
  as excitation
    with type sincpulse and angle 90,
  and refocussing
    with type sincpulse and angle 180.
Specify readout
  with duration 500
  and number-of-columns 64.
Add gradient spoiling around refocussing
  of type balanced.
Add gradient spoiling after segment.
Specify timing parameters
  with TE 50
  and TR 1000.
Specify trajectory
  with Epi-Factor 64
  and ETL 1
  and number-of-rows 64.
Specify measurements
  with count 10.

Listing 5 **DSL formulation of a default EPI sequence.**


Define MRI sequence "EPI-default"
   using a "GradientEcho" with "EPIReadout".
Specify echo
  as excitation
    with type sincpulse and angle 90.
Specify readout
  with duration 500
  and number-of-columns 64.
Add gradient spoiling after segment.
Specify timing parameters
  with TE 35
  and TR 1000.
Specify trajectory
  with Epi-Factor 64
  and ETL 1
  and number-of-rows 64.
Specify measurements
  with count 10.

Listing 6 **DSL formulation of a default bSSFP sequence.**


Define MRI sequence "bSSFP"
  using a "GradientEcho" with "LineReadout".
Add prescans
  with count 8 and type linear.
Specify echo
  as excitation
    with type sincpulse and angle 90.
Specify readout
  with duration 2000
  and number-of-columns 64.
Add gradient spoiling around readout
  of type balanced.
Specify timing parameters
  with TE 4
  and TR 8.
Specify trajectory
  with Epi-Factor 1
  and ETL 1
  and number-of-rows 64.
Specify measurements
  with count 1.

## 3. Experimental setup

This section presents the actual experiments we performed. It concentrates on the presentation of the training data as well as the presentation of four representative cases for optimization.

### 3.1. Generated training data and its properties

**Figure 7:**
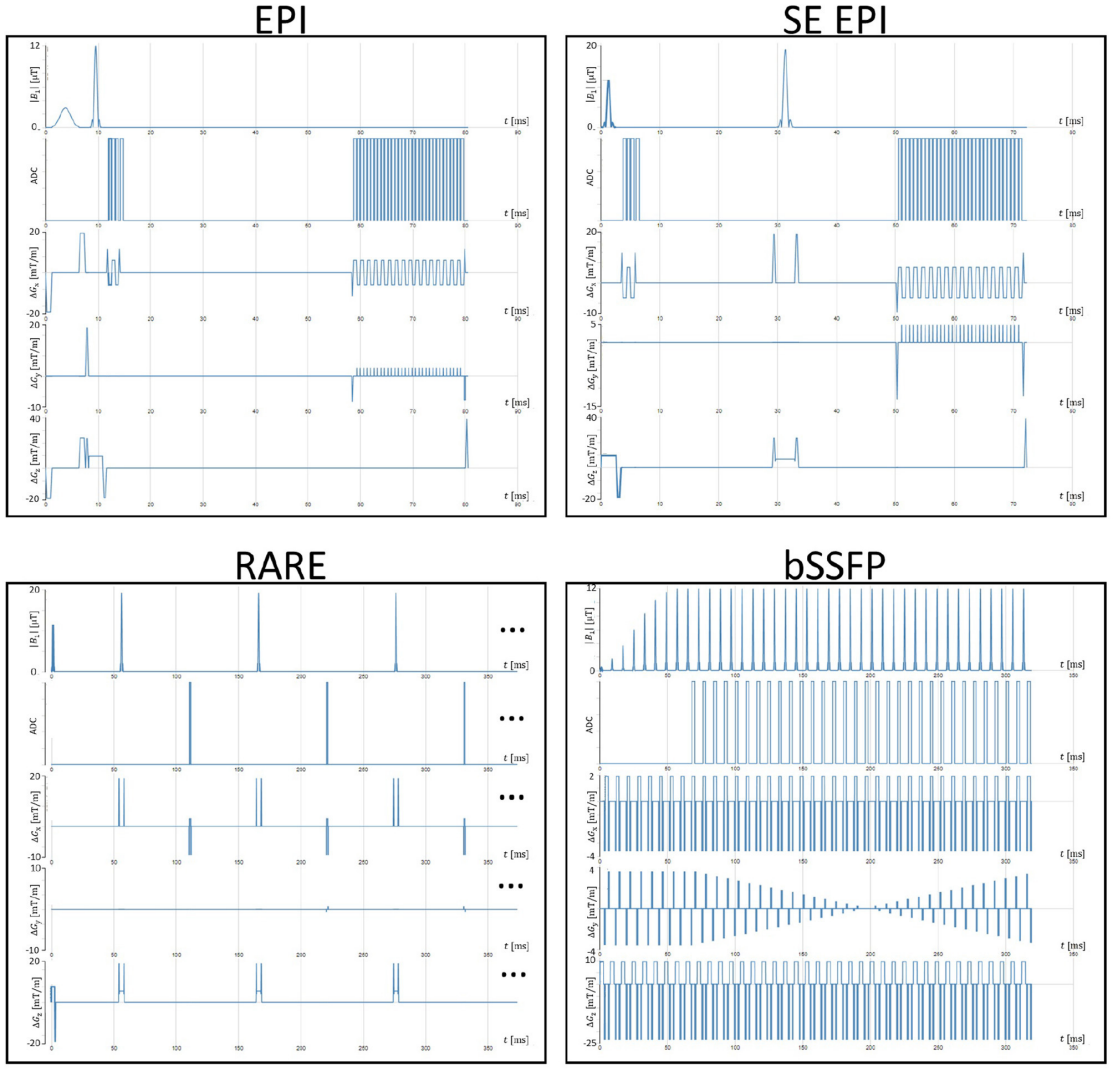
Exemplary sequence diagrams from indicated parameter combinations in Table 1 as generated in the gammaSTAR framework.

For the generation of training data, various sequence configurations are used according to Table 1. Spin echo as well as gradient echo sequences are simulated in combination with echo-planar imaging (EPI) readouts and line readouts. The different combinations of echo and readout type result in four types of MRI sequences, known as gradient and spin-echo echo-planar imaging (EPI, SE EPI; Mansfield, 1977), balanced steady-state free precession (bSSFP, Carr, 1958) and rapid imaging with refocused echoes (RARE, Hennig et al., 1986). Variation of the matrix size yields changes in the expected image sharpness while increasing the measurement time as well as a potential sensitivity to motion. Variation of TE and TR parameters alters the contrast between different types of tissue. ETL and EPI factor parameters have a large impact on, e.g., distortion and motion sensitivity as well as image blurring and ghosting artifacts in reconstructed images. Slight variations in the readout duration were introduced for potential effects on SNR values. Excitation, as well as refocusing angles, were fixed to 90 and 180, respectively. Finally, the number of measurements was altered to introduce potential steady-state effects on the simulation process. Figure 7 shows the sequence diagrams of a single configuration per sequence variant (underlined in Table 1).

### 3.2. Hyperparameters and error metrics

As already mentioned in Section 2, there are three models whose hyperparameters are optimized using error measures. These are the support vector regression, the k-nearest neighbor regression, and a random forest. For the k-nearest neighbor model, we vary the distance parameter (range [0.1, 20]), and the kernel. For the support vector regression, we vary the tolerance parameter ([0.001, 0.9]), the epsilon parameter ([0.1, 0.9]), and again, the kernel. For the random forest mode, we vary the maximum depth ({0, 20}) and the number of trees ({1, 20}). Each model type has 200 evaluations to determine the best hyperparameter set. For implementation, we used the R-package *mlr3verse*. For the implementation of the PCA analysis, we used the built-in functions of R.

There were three different metrics used to guide the optimization of the hyperparameters. The main error metric was the well-known *root mean squared error*. However, this error metric is at a disadvantage when the absolute values are very high. Then, it is more accurate to use a relative measure that motivates the second error metric, the *root relative squared error*. This metric, however, has its difficulties with values close to zero. To have an error metric capable of dealing with that, a logarithmic one was chosen: *root mean squared logarithmic error*. All three values are reported in the results. However, as we saw that the errors did not lead to disparate results, we decided to choose the most common error metric (Shcherbakov et al., 2013).

### 3.3. Specification of optimization algorithms

We specified our evolutionary algorithms to work with real-valued encoding. This can lead to invalid solutions for categorical data, however, as already explained in the methodology, this is taken care of via a repairing function, if possible. Mutation and recombination operators are chosen fittingly to the real-valued encoding. A line crossover chooses a value on a (hyper)line between two values in a room (probability: 0.1) while the Gaussian mutator varies with a given step size around the actual value (probability: 0.9). The selection is guided by an elite approach, together with a tournament approach to ensure diversity in the population. The population size is chosen at 50 as a medium size to keep computation time low, but allow a certain level of diversity. The determination of the maximum number of generations follows a trade-off as well: It should be high enough to enable the algorithm to move toward a global optimum. At the same time, it should be as low as possible to reduce computation time. We chose 100 generations for this experimental setup.

Another important aspect is the construction of the fitness function. Mainly, the fitness function contains the values to be optimized. Additionally, strict constraints are added via a penalty constant, when violated. The same holds for vague constraints, however, the penalty constant is chosen smaller and relative to the optimization value. This is necessary, as the actual optimization goal would have no significant influence on the fitness function. The artifact constraints are included with penalties regarding their discrepancy. If the constraint is only violated slightly, there is only a small penalty, however, for major violations, huge penalties are given.

This leads to the following construction principle for fitness functions based on the optimization goal:

(1) $$\begin{array}{l} fitness\left(x\right)=p{\left(x\right)}_{goal}-\overset{s}{\underset{i=1}{\sum }}ps_{i}\left(x\right)-\overset{t}{\underset{j=1}{\sum }}pv_{j}\left(x\right) \end{array}$$

where *x* is the search space candidate, i.e., the sequence, *p*(*x*) the result of applying the ML-model, *s* is the number of strict constraints, *ps*_*i*_ the penalty awarded for a violation of strict constraint *i*, *t* the number of vague constraints, and *pv*_*j*_ the penalty awarded for a violation of a vague constraint *j*. Usually, penalties are constant values, for constraints regarding ghosting, motion sensibility and distortion sensibility, however, we used an exponential function on the degree of violation, e.g., *exp*(*a*(*p*(*x*)_*ghost*_ − *c*_*ghost*_)), where *a* is some factor, *p*(*x*)_*ghost*_ the predicted ghost value, and *c*_*ghost*_ the boundary from the constraint. This enables small penalties for small violations, and increasing penalties for larger violations.

After sequence optimization, the resulting sequences for both defined goals in Listings 1, 2 are acquired for validation of the provided goals using the gammaSTAR framework on a 3T Siemens VidaFit MR scanner (Siemens Healthineers, Erlangen, Germany). The subject provided informed consent prior to the scan.

## 4. Results

This section contains three main parts: First, we will show and describe data that served for the training of the ML-model. Then, the results of the ML-training are reported. Third, we will show the results of two optimization examples to give the reader an impression.

### 4.1. Training data variation and linearity

**Figure 8:**
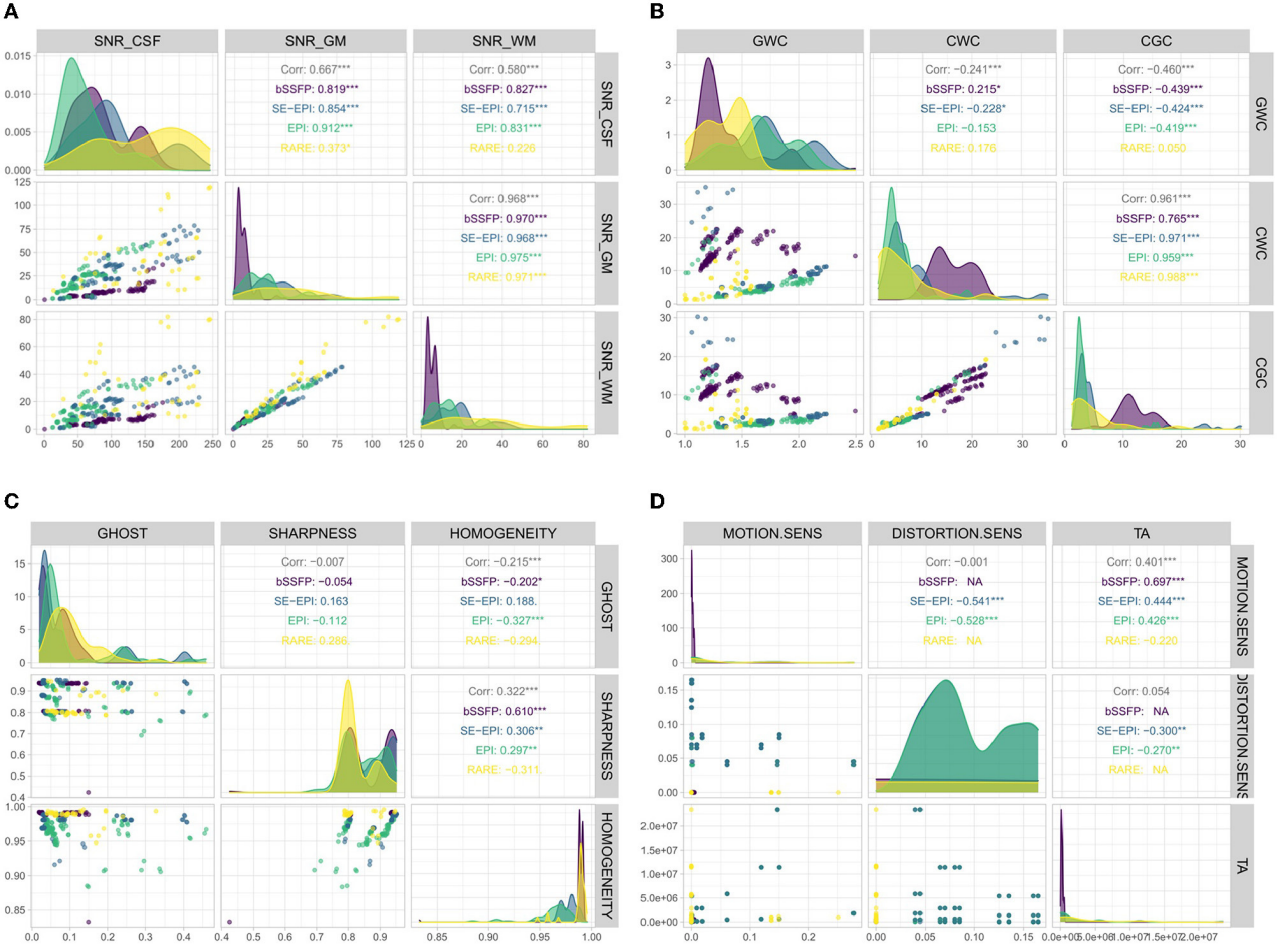
Distribution of output variables differentiated by sequence types. **(A)** SNR-related output variables; **(B)** Contrast-related output variables; **(C)** Ghost, sharpness, and homogeneity; and **(D)** Motion and distortion sensitivity and acquisition time.

For this work 127 bSSFP, 199 EPI/SE EPI and 40 RARE sequences were simulated and respective image metrics were calculated. Figure 8 shows the distribution of the simulated output variables differentiated by the associated sequence type, separated into groups of three variables (Figure 8A: SNR CSF, SNR gray matter, SNR white matter—Figure 8B: gray matter white matter contrast, CSF white matter contrast, CSF gray matter contrast—Figure 8C: Ghosting, sharpness, homogeneity, Figure 8D: Motion sensitivity, distortion sensitivity, acquisition time). All four figures are structured similarly. The different colors represent the different density distributions depending on the underlying sequence type (see colored names in the panels in the upper half). The three upper right panels show the correlation values of the corresponding variables. The number of stars shows the statistical significance. For example, white matter SNR and gray matter SNR seem to be highly correlated, while the other combinations still have high values (besides sequence type RARE) but not as high. The lower left panels show the dependence between both corresponding variables. The high correlations between gray matter SNR and white matter SNR, as well as between CSF white matter contrast and CSF gray matter contrast are visible. Figure 8C shows high values in the training data for Sharpness and Homogeneity as well as the smaller values for Ghost-Artifacts.

After validating that the chosen input variables show a good distribution of output variables, the analysis of the dependency between input and output variables is necessary. Table 2 shows the results of a principal component analysis on the input data in the upper half, and the results for the output variables in the lower half. Principal components differ for input and output variables. The first line in both tables shows the standard deviation that this principle component is responsible for and in the last line, the reader can see the cumulative proportion. Usually, it is sufficient to take as many principal components as are necessary to explain 95% of the data deviation. In this case, it would be sufficient to choose the first seven principal components for the input variables and the first eight principal components for the output variables. The PCA shows that the output variables need more principal components to express their data than the input variables.

**Figure 9:**
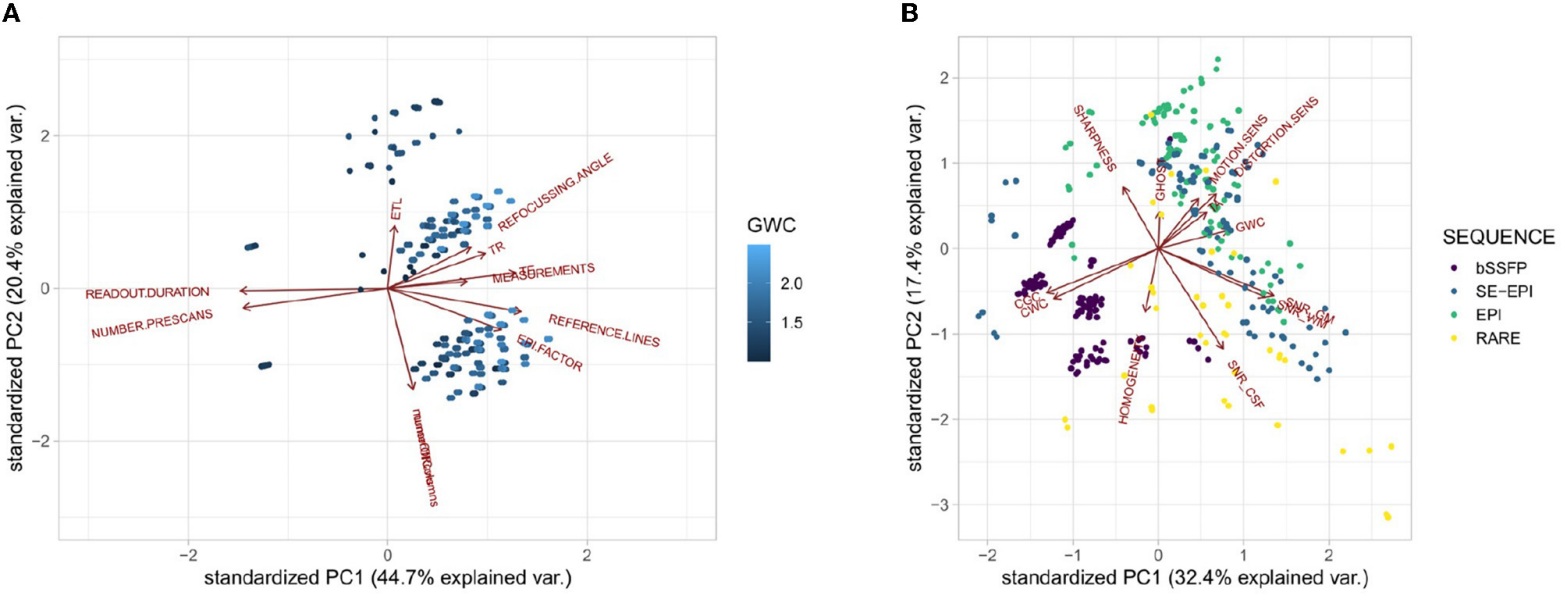
Principal component analysis results. **(A)** PCA Results on the input variables for PC1 and PC2 with output GWC; and **(B)** PCA Results on the output variables for PC1 and PC2 with data colored with respect to the sequence.

Additionally, it is interesting to look at the position of the original data relative to the principal components. To that end, Figures 9A, B each show the direction of the input (output, resp.) variables relative to the first two principal components. For Figure 9A the color of the points shows the GWC value. It is visible that, e.g., the echo train length majorly points in the direction of the second principal component, whereas the input variables TR, TE, refocussing angle, and EPI-factor all point along the direction of the first principal component. They seem to be strongly correlated regarding the training data. Interestingly, the first principal component seems to explain the gray-white matter contrast acceptably well. The more to the right a data point is, the more light the blue and thus, the higher the GWC value. In Figure 9B, the color again represents the sequence type. Gray and white matter SNR point basically in the same direction, as well as gray and white matter contrasts. Additionally, the bSSFP sequences are rather found to the left, whereas both EPI sequences are mostly on the right side of the sharpness/SNR in the CSF border.

### 4.2. Results of ML-training

As discussed in Section 2, a three-way holdout was used for training and evaluating the ML-model. The training data set itself was used for hyperparameter optimization. The resulting best parameters were then applied for all three considered models on the test data set. The results are shown in Table 3 under *test*. These show how well every model in its optimal hyperparameter setting performs on unseen data. Based on these results, the best-performing model was chosen and validated on the validation set. The result of this final test can be found in columns *val*. As these values (again) have been computed on previously unseen data, they can be used as a prognosis of the performance of the machine learning model for new data.

**Table 3:** Result of the machine learning process of test and validation data.

**Model**	**Error**	**GWC**		**CGC**		**CWC**		**SNR-CSF**		**SNR-GM**		**SNR-WM**	
		**test**	**val**	**test**	**val**	**test**	**val**	**test**	**val**	**test**	**val**	**test**	**val**
svm	*rrse*	0.45		0.8		0.77		0.28		0.47	0.49	0.53	0.52
	*rmse*	0.14		5.04		4.06		15.3		**11.5**	10.8	**9.01**	7.75
	*rsle*	0.06		0.39		0.42		0.17		0.55	0.53	0.51	0.46
knn	*rrse*	0.43	0.43	0.38	0.36	0.38	0.41	0.19	0.43	0.52		0.58	
	*rmse*	**0.13**	0.13	**2.38**	2.5	**2.01**	2.48	**10.7**	22.3	12.8		9.88	
	*rsle*	0.05	0.05	0.25	0.3	0.25	0.25	0.21	0.25	0.48		0.45	
ranfor	*rrse*	0.80		0.76		0.72		0.76		0.87		0.88	
	*rmse*	0.25		4.84		3.76		41.7		21.3		14.9	
	*rsle*	0.1		0.46		0.44		0.48		0.80		0.7	
		**Ghost**		**Sharpness**		**Homogeneity**		**Motion**		**Distortion**		**Time**	
		**test**	**val**	**test**	**val**	**test**	**val**	**test**	**val**	**test**	**val**	**test**	**val**
svm	*rrse*	0.53		0.32	0.29	0.75	0.69	0.5	0.48	0.11	0.11	0.54	
	*rmse*	0.05		**0.02**	0.02	**0.01**	0.01	**0.03**	0.03	**0.00**	0.01	5.5e6	
	*rsle*	0.04		0.01	0.01	0.01	0.01	0.02		0.03	0.01	-	
knn	*rrse*	0.45	0.61	0.52		0.76		0.73		0.18		0.58	0.46
	*rmse*	**0.04**	0.04	0.03		0.01		0.04		0.01		**2.7e6**	2.5e6
	*rsle*	0.02	0.04	0.18		0.01		0.04		0.01		1.21	-
ranfor	*rrse*	0.96		0.64		0.85		0.89		0.49		0.88	
	*rmse*	0.08		0.04		0.01		0.49		0.03		4.1e6	
	*rsle*	0.07		0.02		0.01		0.04		0.03		2.3	

Bold values indicate hyperparameter sets and ML models chosen for training based on error metrics.

It is noteworthy that only the *k*-nearest neighbor and the support vector regression performed well enough to be chosen for the final validation. The random regression forest method was discarded due to overall poorer performance. Note that, for some variables however (e.g., homogeneity and sharpness), it performed comparably well. The high values for the *rmse* for acquisition time are a result of the overall high values for this particular variable because the *rmse* is an absolute error metric. One can see that the corresponding *relative* error *rrse* does not strongly deviate from the relative error metric for the other output variables.

**Figure 10:**
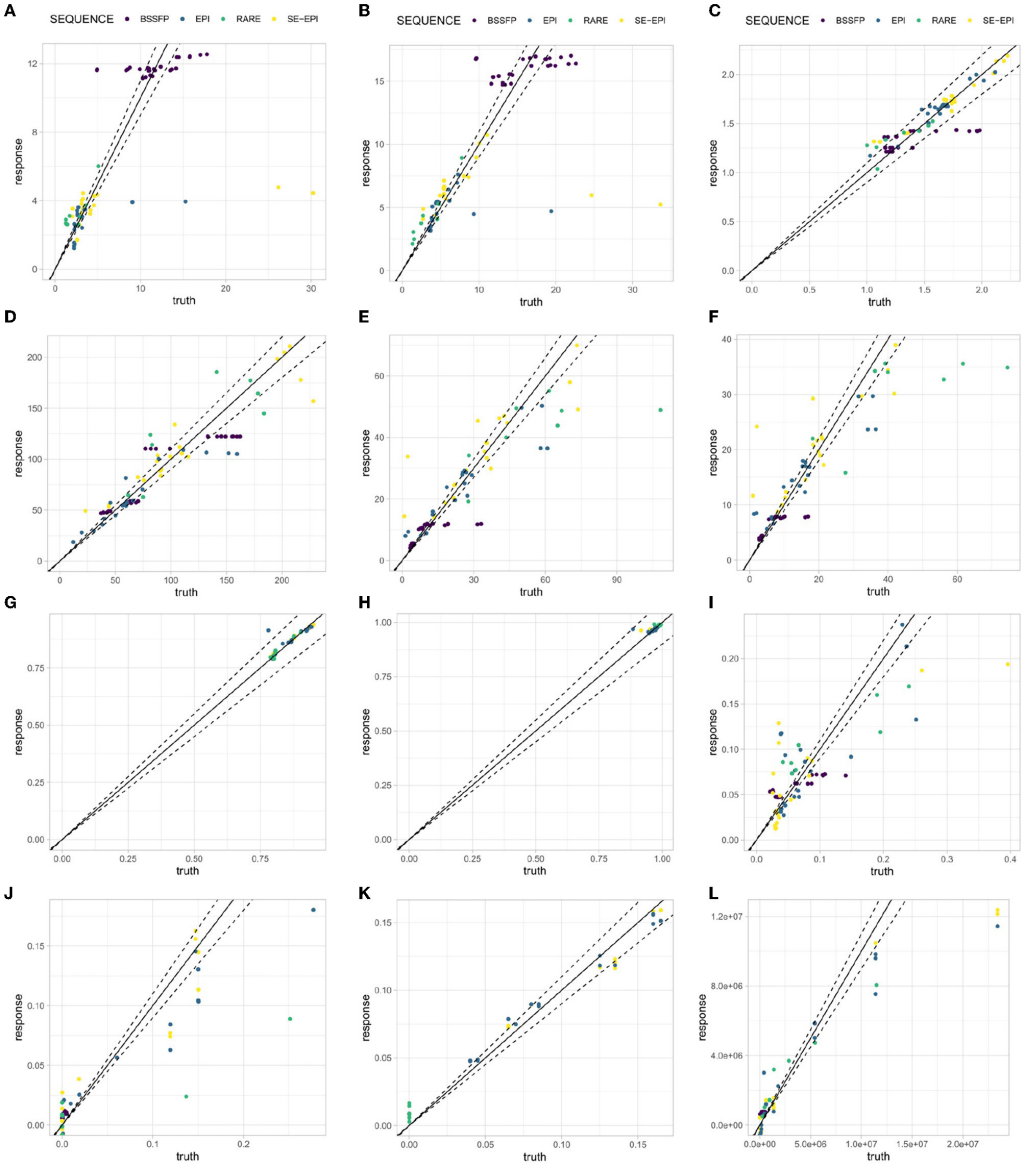
Validation set result on chosen best-performing model with best-performing hyperparameters. **(A)** CGC; **(B)** CWC; **(C)** GWC; **(D)** SNR-CSF; **(E)** SNR-GM; **(F)** SNR-WM; **(G)** Sharpness; **(H)** Homogeneity; **(I)** Ghosting; **(J)** Motion Sens.; **(K)** Distortion Sens.; and **(L)** Acquisition Time.

Figure 10 shows the results of the validation data (the last portion of the overall training data) on the—for each output variable—chosen ML-model and hyperparameter settings. Each subfigure represents one output variable. It compares the predicted value (*response*) to the value from the validation data (*truth*). The colors depict the four different types of sequences present in the training data. The solid line shows the bisector. If the model was a perfect prediction, every point would lie on the bisector. The dashed lines show the 10% margins, i.e., every data point inside the dashed lines deviates at most 10% from the original value.

Overall, the machine learning model predicts the validation data into the 10% deviation corridor around the bisector. However, there are also output variables that have several outliers. Particularly ghosting, acquisition time and motion sensitivity. There is no visible influence regarding the quality of the model with respect to the sequence types, i.e., the sequence type does not seem to influence the quality of the prediction.

### 4.3. Results of optimization flow

Finally, the results of the MRI sequence optimization process and the effect of the repairing step are presented. The exemplary requirement from Listings 1, 2 were transformed to an optimization function and constraints through the use of the DSL as well as a *dictionary* for the ordinal values with regard to the respective variables. This process yielded the following formal constraints for the first task:

$$\begin{array}{ll} GWC & ->max!\\ SNR-GM & >30\\ SNR-WM & >30\\ SNR-GM & >SNR-WM\\ GHOST & <0.05 \end{array}$$

and for the second task:

$$\begin{array}{ll} SNR-CSF & ->max!\\ SHARPNESS & >0.85\\ CGC & >10\\ CWC & >10\\ GHOST & <0.05\\ MOTION.SENS & <0.05\\ DISTORTION.SENS & <0.05 \end{array}$$

These optimization goals were translated to fitness functions as described in Section 3.3.

**Figure 11:**
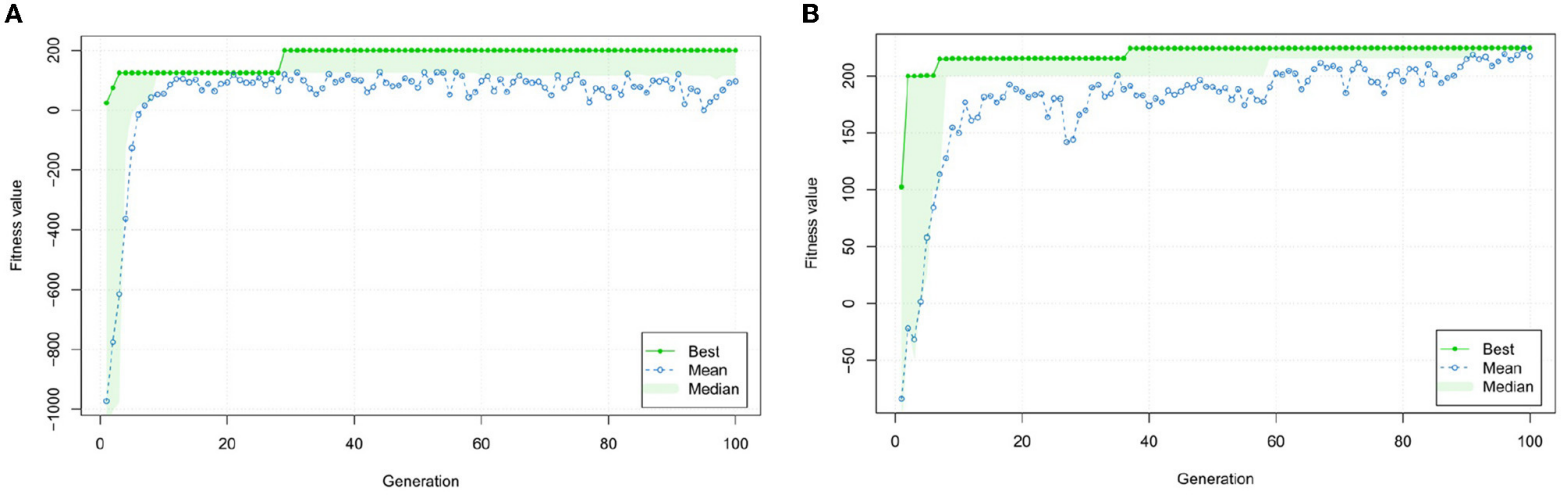
Evolution process of fitness for both optimization tasks. **(A)** Evolution of fitness values throughout the optimization process for the first requirement example; and **(B)** Evolution of fitness values during the optimization process for the second requirement example.

Figures 11A, B show the evolution of the fitness function. Although the goal is maximization, one can see that both start with negative fitness values. This is due to the fact, that the constraints are violated and thus the penalization constant drives the fitness function toward a negative value. Throughout the evaluation, however, it thrives toward the positive area which—through the choice of the penalization constants—signalizes that no constraint is violated anymore.

**Figure 12:**
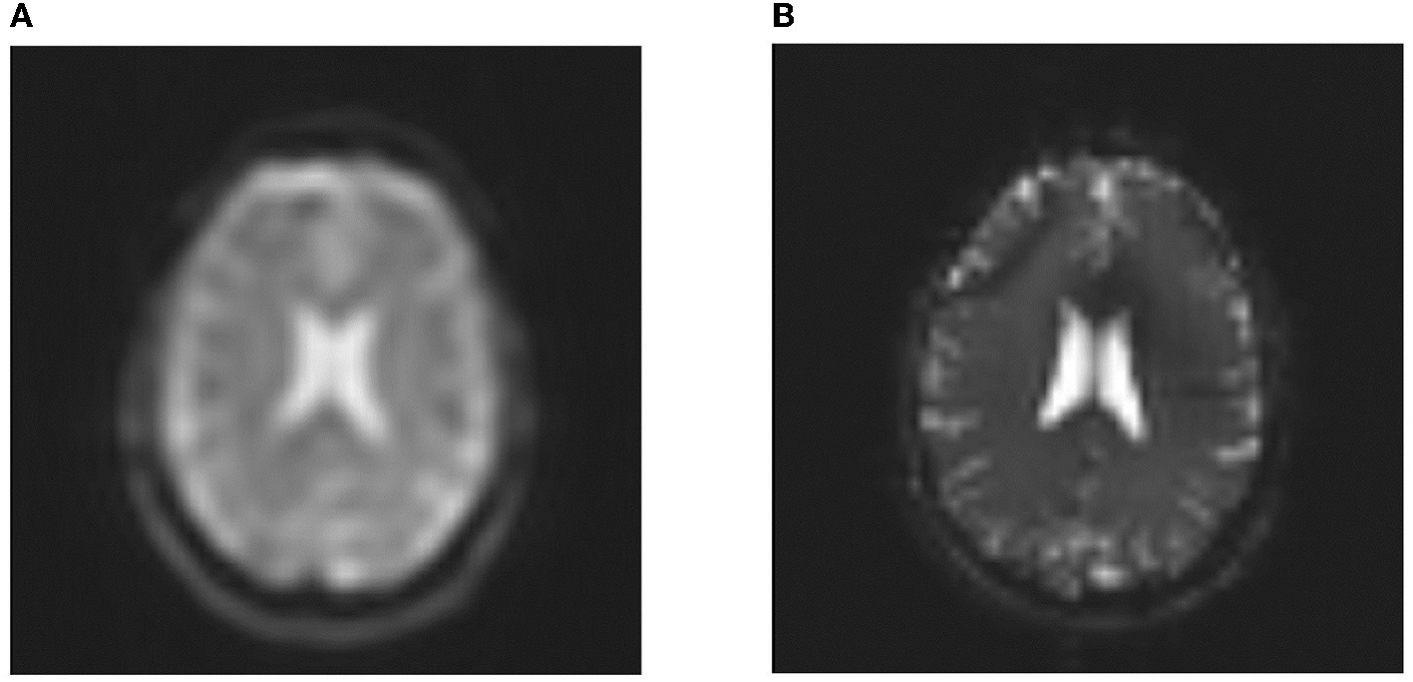
*In-vivo* examples of optimized MR sequence protocols. **(A)** *In-vivo* examples of MR sequences as defined under Listing 7; and **(B)** *In-vivo* examples of MR sequences as defined under Listing 8.

After finishing the evolution process, the solution with the best fitness value is returned. These first need repairing (as mentioned in Section 2) to ensure they represent valid sequences. This is necessary for two reasons: First, as already mentioned, the presence of categorical data. Second, the optimization system does not yet contain physical knowledge about sensible sequences. An example of the first repair is the question of whether the sequence is of type GradientEcho or SpinEcho and if it is of type LineReadout or EpiReadout. This can be done by using the information in the refocussing angle and the EpiFactor. An example of the second problem is the interplay of TE and TR. Here, the optimization does not yet include physical logic to ensure that respective values yield plausible sequences. For instance, the second optimization yields a TE/TR combination of 130/4,300 ms which is not reasonable in combination with a gradient echo and a line readout. Therefore, these values are repaired to the largest TE/TR available in the generated training data (17/36 ms). Additional discussion on how limitations of the repairing function are given in section 5.2. Re-transforming these repaired solutions into the presented DSL yields the MRI sequences as given in Listings 7, 8. Figure 12 finally shows *in-vivo* scans using optimized MR sequence examples from Listings 7, 8.

Listing 7 **DSL formulation of the solution for requirement example 1.**


define MRI sequence "Example 1"
  using a "SpinEcho" with "EPIReadout".
Specify echo
  as excitation
    with type sincpulse and angle 90,
  and refocussing
    with type sincpulse and angle 180.
Specify readout
  with duration 500
  and number-of-columns 32.
Specify timing parameters
  with TE 46
  and TR 5014.
Specify trajectory
  with Epi-Factor 32
  and ETL 1
  and number-of-rows 32.
Specify measurements
  with count 10.

Listing 8 **DSL formulation of the solution for requirement example 2.**


define MRI sequence "Example 2"
  using a "GradientEcho" with "LineReadout".
Specify echo
  as excitation
    with type sincpulse and angle 90,
Specify readout
  with duration 2000
  and number-of-columns 64.
Specify timing parameters
  with TE 17
  and TR 36.
Specify trajectory
  with Epi-Factor 1
  and ETL 1
  and number-of-rows 64.
Specify measurements
  with count 2.
Add prescans
  with count 8.

## 5. Discussion

### 5.1. Restrictions of software tools and MRI simulations

In this work, a chain of different software toolboxes is utilized to generate training data for the ML algorithms (see Figure 5). Due to the lack of a direct JEMRIS export, the workaround using pulseq exports with subsequent conversion to the JEMRIS format is utilized. While appropriate for this work, this approach is highly inefficient concerning computational demands because JEMRIS sequences are usually formulated in a high-level fashion, while Pulseq files implement a low-level formulation of MRI sequences. This results in computation times of several hours for complicated sequences, being a limiting factor during the training data generation process. The latter would therefore benefit from a direct DSL to JEMRIS export. An alternative approach would be the implementation of a low-level differentiable representation of MR sequences as already proposed (Loktyushin et al., 2021). This would not only allow for faster data generation but would also enable hybrid optimization approaches using analytical gradients.

It must also be mentioned that MRI sequences are restricted to Cartesian trajectories in this work. While the implementation of Cartesian image reconstruction methods is simple, non-Cartesian trajectories require more sophisticated approaches. Here, the reconstruction algorithm itself will have a major impact on different image quality metrics and we suggest incorporating open reconstruction frameworks such as Gadgetron (Hansen and Sørensen, 2012) or BART (Uecker et al., 2015) into the reconstruction pipeline providing heavily optimized algorithms for this purpose. Note that these algorithms might also require additional simulation of coil sensitivity profiles. Another challenge will be to align the simulated profiles to the real ones used during the actual MRI experiments. Close alignment is important because the sensitivity profiles have a significant impact on the image quality in parallel imaging scenarios. Finally, it shall be noticed that the calculation of presented image metrics is suitable for Cartesian MRI sequences but might not be reasonable for spiral or radial trajectories. Images reconstructed from radial projections often suffer from streaking artifacts that overlay the background noise, thus making the presented approach for SNR calculation difficult. This also holds for the estimation of ghosting artifacts due to the lack of a phase-encoding direction. Therefore, a comparison of Cartesian and non-Cartesian trajectories in terms of provided metrics is difficult and respective metric algorithms need to be adopted for these scenarios.

### 5.2. Generated training data and its properties

The simulated training data using sequence parameters from Table 1 results in a sufficient variation of quantitative image metrics as can be seen from Figure 8. The given SNR and contrast examples (see Figures 8A, B) clearly show different trends for the different sequence types analyzed in this work. It is reasonable that SNR values of gray and white matter are highly correlated since both types of tissue only show minor variations in their respective T1 and T2 values. The PCA allows getting deeper insights into the relation between input and output variables (see Figure 9). The fact that the first principle component of the GWC value (see Figure 9A) is majorly affected by the TE as well as TR is expected because these parameters have a large impact on the resulting image contrast and the amount of signal which is available during data readout. Nonetheless, the rather small amount of training data, which was generated for this work, is a major restriction that required repairing of generated sequence configurations. Therefore, additional data, generated from input parameters which are kept fixed for this work, will be beneficial for further specification of the relation between input and output parameters. For example, it is well known, that excitation and refocussing flip angles have large effects on resulting SNR levels and that 90/180 combinations are not optimal in many scenarios. In addition, the effect of preparation modules such as inversion recovery and fat saturation, which are part of the DSL, is not used during data generation due to a restricted time for data generation. Including the effects of such preparation modules into the machine learning routine would result in additional contrast flexibility because signal from individual T1 components or off-resonant signal could be canceled out completely, which is exploited in e.g., fluid-attenuated inversion recovery (FLAIR, Hajnal et al., 1992) imaging. Finally, additional types of trajectories such as radial or spiral should be incorporated into the simulation because the type of trajectory has strong effects regarding motion and distortion sensitivity and Cartesian readouts are not always the optimal approach.

### 5.3. ML models and hyperparameters

ML results from Figure 10 show decent predictability of resulting parameters in the validation dataset for the MRI sequences investigated in this work. However, for some sequences, predicted and ground truth values show deviations (see SE EPI CGC values in Figure 10A). Here, image artifacts might result in variations of calculated image metrics, which were not expected from the majority of the remaining data, which guided the learned relations. It should be further investigated, whether this occurs for such image data which also shows low homogeneity scores. This could help to identify misleading simulations and further improve the quality of the ML model in the future. In general, the quality of generated training data was sufficient to apply the DSL optimization routines as introduced in this work.

### 5.4. Optimization strategies

The optimization of DSL sequences concerning two given scenarios (see Listings 1, 2) yields two reasonable configurations of MRI sequences (see Listings 7, 8). In the case of Listing 1, the maximization of contrast between gray and white matter in combination with lower bounds for respective SNR values yields a SE EPI sequence with a low number of rows and columns (32x32) as well as a long readout duration which is beneficial in terms of SNR. The contrast between gray and white matter is mainly adjusted via TE and TR values of respective sequences. To prevent SNR loss, SE EPI seems to be preferred over EPI to enable more flexibility in terms of TE variation. Finally, a large number of measurements (10) results in steady state effects, which seem to be beneficial for the provided goals. Note that no segmentation is suggested here, which is beneficial to prevent ghosting artifacts. Figure 12 (left) shows the corresponding *in-vivo* MR image. The low matrix size results in decreased sharpness of structures in the interpolated image. Note that no Nyquist ghosts are visible due to steady state approaching of the magnetization after ten measurements. The example also shows distinguishable gray and white matter structures as forced by the optimization procedure.

The second optimization aims to maximize the SNR in CSF regions while ensuring adequate image sharpness with the prevention of unwanted image artifacts in terms of motion or geometric distortion. The optimization proposes a bSSFP sequence with a matrix size of 64x64. This is reasonable to ensure adequate image sharpness. The bSSFP sequence also offers robustness against off-resonance effects as well as motion as long as motion appears as a slow drift as simulated in this work. The corresponding *in-vivo* scan (see Figure 12, right) clearly shows sharper brain structures when compared to the previous configuration. Also note the bright CSF signal in the ventricles, which corresponds well to the target of maximized SNR in CSF regions. Note, however, that the optimality of generated image sequences heavily depends on the underlying simulated database, which guides the repairing algorithm. In this work, this database was still small due to high computational demands, being a limiting factor, which will be overcome in the future. Future work should also simulate additional motion patterns on shorter timescales to cover different motion scenarios during the scan. In the case of the second optimization goal, the vague constraint to prevent ghosting artifacts is exceeded in favor of improved image sharpness (see Section 4.3), which could have interesting implications for future scenarios. When it comes to motion and distortion sensitivity, these could be formulated as vague or hard constraints. While vague constraints are an option in brain imaging, where motion is likely but not guaranteed and severe inhomogeneities are mitigated by optimized shimming processes, hard constraints should be formulated in the case of abdominal imaging, where breathing motion can not be neglected and severe off-resonance effects are expected due to air-tissue interfaces near the lungs. Here, the prevention of artifacts should be prioritized because otherwise reconstructed images cannot be used for diagnostic purposes. Future work needs to incorporate these considerations into the repairing function, which might then propose different configurations to the user, optimized for these two scenarios. The quality of repaired sequence configurations also heavily depends on a large amount of training data. The optimization and repairing routines would especially benefit from additional data, corresponding to sequence configurations, yielding images with poor quality and/or low signal, naturally restricting the search space for certain sequence parameters and reducing the number of required repairs. Finally, this work only demonstrated the application of *in-vivo* measurements on a single-vendor system. Future work should therefore further investigate the optimality of generated sequences in a multi-vendor environment, ensuring comparable results on different systems. However, all sequences resulting in the proposed optimization workflow are representable as gammaSTAR sequences which ensures cross-vendor application (Cordes et al., 2020).

### 5.5. Generalizability of DSL approach

This work presents a high-level DSL specifically designed to reduce the complexity of MRI sequences to a small level of flexibility. This could be helpful for users who are not familiar with the underlying physical principles of MRI sequences and thus might be easily overwhelmed by a large variety of options. However, experienced users might want to have more flexibility when designing MRI sequences. We, therefore, propose additional layers in the DSL which introduce different levels of complexity to meet the needs of different groups of users. For example, the upper-level DSL offers the selection between different types of echoes. If spin-echo is selected, the pulse shape is set to a pre-defined sample (e.g., a slice-selective sinc pulse) and the refocusing angle is set to 180°. A second-level DSL could then allow switching between different pulse shapes such as sinc, rect or more complex waveforms and allows adjustments of the refocusing angle. In the lowest DSL layer, actual ADC, RF and gradient events could be defined for each discrete raster timepoint of the MRI system. On this level, the optimization would be closely related to the approaches which were previously presented in Loktyushin et al. (2021) (MRZero) and (Glang et al., 2022) (MR-double-zero). Further investigation is therefore recommended on how to combine the presented ideas with our DSL approach. As seen, the DSL approach has the potential to offer strong flexibility in terms of sequence optimization for different scenarios by introducing different layers of complexity.

## 6. Conclusion

This work demonstrates the definition and optimization of MRI sequences by utilizing a high-level DSL. This DSL allows to build a variety of MRI sequences by combining different sequence components, enabling easy access to the often complex MRI sequence programming for a larger number of users with different levels of experience. In addition, the presented DSL allows automized optimization of MRI sequences regarding a variety of different image quality goals such as SNR, contrasts as well as the sensitivity to motion and geometric distortion by using machine learning approaches. This has the potential to offer optimal solutions for different clinical scenarios, potentially reducing exam times by preventing suboptimal MRI protocol settings. Future work will cover additional DSL layers of higher flexibility to suit the needs of more experienced MRI sequence programmers as well as an optimization of the underlying MRI simulation process. This will eventually enable the machine learning-based proposal of completely new types of generated MR sequences, tackling a specific clinical problem optimally.

## Data availability statement

The original contributions presented in the study are included in the article/Supplementary material, further inquiries can be directed to the corresponding author.

## Author contributions

DH contributed to all work parts that involve setting up MRI sequence and protocols in gammaSTAR and exporting them into the pulseq format. JH covered the MRI simulation tasks using Jemris and setting up the training data. CP contributed by setting up the ML learning and optimization work packages as well as by implementing the domain specific languages. DH, JH, and CP wrote the first draft of this manuscript. CL guided the team in all tasks related to domain specific languages. RD guided the team in all tasks related to machine learning and optimization. MG is the leading MRI domain expert of the team and helped in describing the overall workflow and applications. All authors contributed to the article and approved the submitted version.

## Footnotes

^1^Please note that this example is not complete in any way with regard to the English language. It is only meant to illustrate the substitution structure. A far more precise context-free grammar can be found in Jurafsky and Martin (2008).
