# Supplementary material for: AI-driven and automated MRI sequence optimization in scanner-independent MRI sequences formulated by a domain-specific language
Source: Front Neuroimaging. 2023 May 12;2:1090054. doi: 10.3389/fnimg.2023.1090054 (PMC10406289; doi:10.3389/fnimg.2023.1090054)
Supplement: Supplementary file 1 [file Data_Sheet_1.ZIP › optimisation/analysis.html]

ML-Training and Search


# ML-Training and Search

#### anonymous for double-blind review

#### 2023-02-08

# Optimising MRI sequences - documentation of implementation.

This document is the description of the machine learning and
optimization implementation and results.

## Loading of required packages

First, as usal, we load the required packages. *mlr3verse* is
for ML-training, *GA* for genetic (evolutionary) algorithms,
*ggplot2* and *GGally* for plotting, and the rest for data
handling.

```
## 
## Attache Paket: 'dplyr'
```

```
## Die folgenden Objekte sind maskiert von 'package:stats':
## 
##     filter, lag
```

```
## Die folgenden Objekte sind maskiert von 'package:base':
## 
##     intersect, setdiff, setequal, union
```

```
## 
## Attache Paket: 'tidyjson'
```

```
## Das folgende Objekt ist maskiert 'package:stats':
## 
##     filter
```

```
## 
## Attache Paket: 'jsonlite'
```

```
## Das folgende Objekt ist maskiert 'package:tidyjson':
## 
##     read_json
```

```
## Lade nötiges Paket: mlr3
```

```
## 
## Attache Paket: 'rjson'
```

```
## Die folgenden Objekte sind maskiert von 'package:jsonlite':
## 
##     fromJSON, toJSON
```

```
## Registered S3 method overwritten by 'GGally':
##   method from   
##   +.gg   ggplot2
```

```
## Lade nötiges Paket: foreach
```

```
## Lade nötiges Paket: iterators
```

```
## Package 'GA' version 3.2.3
## Type 'citation("GA")' for citing this R package in publications.
```

```
## 
## Attache Paket: 'GA'
```

```
## Das folgende Objekt ist maskiert 'package:utils':
## 
##     de
```

## Loading of training data

Now, we read the training data from the directory
*training\_database*. These are two json files for each datum -
one with the input data, one with the output data. We find the matching
ones, check for mistakes, join them and build on big table. The final
dataset includes all input data, all output data and all data.

```
#######Reading in Training Data and fixing input issues
###important: please set directory name to training_database directory
directoryName <- "training_database/"
importAndFix <- function(directoryName){
      fileList <- list.files(path=directoryName)
      for(i in 1:length(fileList)){
        fileName <- fileList[i]
        fileNameList <- strsplit(fileName, "_")
        jsonName <- data.frame(name=fileNameList[[1]][2])
        
        if(str_contains(fileNameList[[1]][3], "input")) {
          #print(paste(directoryName, fileName, sep=""))
          jsonResult <- fromJSON(file = paste(directoryName, fileName, sep=""))
          jsonDataInput <- as.data.frame(jsonResult)
          
          
          if(jsonDataInput$SEQUENCE != "RARE"){
            jsonDataInput <- mutate(jsonDataInput, ETL="1")
          }
          if(!str_contains(jsonDataInput$SEQUENCE, "EPI")){
            jsonDataInput <- mutate(jsonDataInput, EPI.FACTOR="1")
          }
          #print(jsonName)
          jsonDataInput <- bind_cols(jsonName, jsonDataInput)
          jsonDataInput$SEQUENCE <- as.factor(jsonDataInput$SEQUENCE)
          jsonDataInput$numberOfRows <- as.numeric(jsonDataInput$numberOfRows)
          jsonDataInput$numberOfColumns <- as.numeric(jsonDataInput$numberOfColumns)
          jsonDataInput$REFERENCE.LINES <- as.numeric(jsonDataInput$REFERENCE.LINES)
          jsonDataInput$TE <- as.numeric(jsonDataInput$TE)
          jsonDataInput$TR <- as.numeric(jsonDataInput$TR)
          jsonDataInput$EPI.FACTOR <- as.numeric(jsonDataInput$EPI.FACTOR)
          jsonDataInput$ETL <- as.numeric(jsonDataInput$ETL)
          jsonDataInput$READOUT.DURATION <- as.numeric(jsonDataInput$READOUT.DURATION)
          jsonDataInput$EXCITATION.ANGLE <- as.numeric(jsonDataInput$EXCITATION.ANGLE)
          jsonDataInput$REFOCUSSING.ANGLE <- as.numeric(jsonDataInput$REFOCUSSING.ANGLE)
          jsonDataInput$MEASUREMENTS <- as.numeric(jsonDataInput$MEASUREMENTS)
          jsonDataInput$NUMBER.PRESCANS <- as.numeric(jsonDataInput$NUMBER.PRESCANS)
          
          if(i==1){
            overall_inputData <- jsonDataInput
            
          } else {
            overall_inputData <- bind_rows(overall_inputData, jsonDataInput)
            
          }
        }
        
        if(str_contains(fileNameList[[1]][3], "output")){
          jsonResult <- fromJSON(file =paste(directoryName, fileName, sep=""))
          
          jsonDataOutput <- as.data.frame(jsonResult)
          
          #print(jsonName)
          jsonDataOutput <- bind_cols(jsonName, jsonDataOutput)
          jsonDataOutput$MOTION.SENS <- as.numeric(jsonDataOutput$MOTION.SENS)
          jsonDataOutput$TA <- as.numeric(jsonDataOutput$TA)
          jsonDataOutput$DISTORTION.SENS <- as.numeric(jsonDataOutput$DISTORTION.SENS)
          
          if(i==2){
            overall_outputData <- jsonDataOutput
          } else {
            overall_outputData <- bind_rows(overall_outputData, jsonDataOutput)
            
          }
        }
        
      }
      overallData <- inner_join(overall_inputData, overall_outputData, by="name")
      overallData[is.na(overallData)] <- 0
      return(list(overall_inputData, overall_outputData, overallData))
}

fullDataSet <- importAndFix(directoryName)
```

## Visualizations of training data

Here, we plot general information about the training data.

```
#### initialise general figure theme, based on themelight by Ggplot2
kimbi_theme <- function(){ theme_light(base_size=13)}

ggpairs(data=fullDataSet[[3]], columns = 7:10, aes(color=SEQUENCE)) + kimbi_theme()
```

```
## Warning in cor(x, y): Standardabweichung ist Null

## Warning in cor(x, y): Standardabweichung ist Null

## Warning in cor(x, y): Standardabweichung ist Null

## Warning in cor(x, y): Standardabweichung ist Null

## Warning in cor(x, y): Standardabweichung ist Null

## Warning in cor(x, y): Standardabweichung ist Null

## Warning in cor(x, y): Standardabweichung ist Null

## Warning in cor(x, y): Standardabweichung ist Null

## Warning in cor(x, y): Standardabweichung ist Null

## Warning in cor(x, y): Standardabweichung ist Null

## Warning in cor(x, y): Standardabweichung ist Null

## Warning in cor(x, y): Standardabweichung ist Null

## Warning in cor(x, y): Standardabweichung ist Null

## Warning in cor(x, y): Standardabweichung ist Null
```

```
##plotting contrasts
ggpairs(data=fullDataSet[[3]], columns = 20:22, aes(fill=SEQUENCE, colour=SEQUENCE, alpha=0.7)) + scale_color_viridis_d() + scale_fill_viridis_d() + kimbi_theme() + theme(strip.background = element_rect(fill="lightgray"), strip.text = element_text(size=14, color="black"))
```

```
##plotting SNR's
ggpairs(data=fullDataSet[[3]], columns = 23:25, aes(fill=SEQUENCE, colour=SEQUENCE, alpha=0.7)) + scale_color_viridis_d() + scale_fill_viridis_d() + kimbi_theme()+ theme(strip.background = element_rect(fill="lightgray"), strip.text = element_text(size=14, color="black"))
```

```
##plotting artifacts
ggpairs(data=fullDataSet[[3]], columns = 26:28, aes(fill=SEQUENCE, colour=SEQUENCE, alpha=0.7)) + scale_color_viridis_d() + scale_fill_viridis_d() + kimbi_theme()+ theme(strip.background = element_rect(fill="lightgray"), strip.text = element_text(size=14, color="black"))
```

```
##plotting sens and TA
ggpairs(data=fullDataSet[[3]], columns = 29:31, aes(fill=SEQUENCE, colour=SEQUENCE, alpha=0.7)) + scale_color_viridis_d() + scale_fill_viridis_d() + kimbi_theme()+ theme(strip.background = element_rect(fill="lightgray"), strip.text = element_text(size=14, color="black"))
```

```
## Warning in cor(x, y): Standardabweichung ist Null

## Warning in cor(x, y): Standardabweichung ist Null
```

## Principal Component Analysis

Next, we use the prcomp-functionality of R to perform a PCA Analysis.
We exclude variables that are constant or non-cardinal.

```
  test.pca <- prcomp(fullDataSet[[3]][,c(4:11, 13:14, 19)], center=TRUE, scale=TRUE)
  test.pca
```

```
## Standard deviations (1, .., p=11):
##  [1] 2.217727e+00 1.501505e+00 1.205156e+00 8.798113e-01 7.691508e-01
##  [6] 6.323002e-01 5.765552e-01 4.923101e-01 1.802542e-01 4.501824e-02
## [11] 4.251284e-16
## 
## Rotation (n x k) = (11 x 11):
##                           PC1          PC2         PC3         PC4         PC5
## numberOfRows       0.07261801 -0.577459914 -0.38748621  0.01789855  0.03677109
## numberOfColumns    0.07261801 -0.577459914 -0.38748621  0.01789855  0.03677109
## REFERENCE.LINES    0.39399641 -0.141947235  0.30754027 -0.06174585 -0.07812924
## TE                 0.38307649  0.084432254  0.03419260 -0.16221025  0.20887654
## TR                 0.28783191  0.213523373 -0.23343911 -0.19204584  0.65692234
## EPI.FACTOR         0.33570812 -0.243074270  0.19885792 -0.07794809 -0.10701936
## ETL                0.02252346  0.363145207 -0.61287548  0.03827924  0.03400813
## READOUT.DURATION  -0.43651566 -0.007039125 -0.09595265  0.05759663  0.07388807
## REFOCUSSING.ANGLE  0.24828250  0.234744217 -0.34928684 -0.05619997 -0.69525021
## MEASUREMENTS       0.23535797  0.055047897  0.01645323  0.95696303  0.10205976
## NUMBER.PRESCANS   -0.42875929 -0.105194252  0.05315226  0.05057373  0.06447507
##                           PC6         PC7         PC8          PC9
## numberOfRows       0.03581936  0.08622737 -0.02628966  0.006269091
## numberOfColumns    0.03581936  0.08622737 -0.02628966  0.006269091
## REFERENCE.LINES   -0.03249651  0.10887465 -0.33209895 -0.698246952
## TE                 0.02371104  0.54452692  0.69020664 -0.039444820
## TR                 0.45211776 -0.33848763 -0.16870491 -0.034509497
## EPI.FACTOR        -0.25189634 -0.72269360  0.42872360  0.039985522
## ETL               -0.60605660 -0.07675402  0.03413441 -0.339139370
## READOUT.DURATION   0.17327456 -0.12012687  0.32194802 -0.103723592
## REFOCUSSING.ANGLE  0.50741308 -0.07803132  0.08884485 -0.057159924
## MEASUREMENTS       0.10115285 -0.01891981  0.06691057 -0.006657740
## NUMBER.PRESCANS    0.25084829 -0.11462117  0.28721316 -0.615582059
##                            PC10          PC11
## numberOfRows       3.556880e-05 -7.071068e-01
## numberOfColumns    3.556880e-05  7.071068e-01
## REFERENCE.LINES   -3.307141e-01 -9.714451e-17
## TE                -6.471676e-06  7.632783e-17
## TR                 1.254418e-03 -4.510281e-17
## EPI.FACTOR         9.281602e-04 -8.326673e-17
## ETL                1.897065e-03  1.387779e-17
## READOUT.DURATION  -7.953346e-01 -1.387779e-17
## REFOCUSSING.ANGLE  8.844936e-04 -2.081668e-17
## MEASUREMENTS      -1.575375e-05  6.938894e-18
## NUMBER.PRESCANS    5.080002e-01  3.122502e-17
```

```
  #g <- ggbiplot(test.pca, groups=fullDataSet[[3]]$GWC) + labs(colour="GWC") + coord_cartesian(xlim=c(-3, 3), ylim=c(-3,3)) + theme_light(base_size = 15)
  #ggsave(filename = paste(finalGraphsFolderPathName, "PCAPlot-","GWC-12.pdf", sep=""))
  #ggsave(filename = paste(finalGraphsFolderPathName, "PCAPlot-","GWC-12.jpeg", sep=""), dpi=300)
  #g <- ggbiplot(test.pca, groups=fullDataSet[[3]]$CWC) + labs(colour="CWC")
  #ggsave(filename = paste(finalGraphsFolderPathName, "PCAPlot-","CWC-12.pdf", sep=""))
  #g <- ggbiplot(test.pca, groups=fullDataSet[[3]]$CGC)
  #ggsave(filename = paste(finalGraphsFolderPathName, "PCAPlot-","CGC-12.pdf", sep=""))
  #g <- ggbiplot(test.pca, groups=fullDataSet[[3]]$GHOST)
  #ggsave(filename = paste(finalGraphsFolderPathName, "PCAPlot-","GHOSTING-12.pdf", sep=""))
  #g <- ggbiplot(test.pca, groups=fullDataSet[[3]]$SNR_CSF)
  #ggsave(filename = paste(finalGraphsFolderPathName, "PCAPlot-","snrcsf-12.pdf", sep=""))
  #g <- ggbiplot(test.pca, groups=fullDataSet[[3]]$SNR_GM)
  #ggsave(filename = paste(finalGraphsFolderPathName, "PCAPlot-","snrg-12.pdf", sep=""))
  #g <- ggbiplot(test.pca, groups=fullDataSet[[3]]$SNR_WM)
  #ggsave(filename = paste(finalGraphsFolderPathName, "PCAPlot-","snrwm-12.pdf", sep=""))
  #g <- ggbiplot(test.pca, groups=fullDataSet[[3]]$SHARPNESS)
  #ggsave(filename = paste(finalGraphsFolderPathName, "PCAPlot-","sharpness-12.pdf", sep=""))
  
  testOutput.pca <- prcomp(fullDataSet[[3]][, 20:31], center=TRUE, scale = TRUE)
  summary(testOutput.pca)
```

```
## Importance of components:
##                           PC1    PC2    PC3    PC4     PC5     PC6     PC7
## Standard deviation     1.9730 1.4509 1.3285 1.1695 0.91708 0.88033 0.73762
## Proportion of Variance 0.3244 0.1754 0.1471 0.1140 0.07009 0.06458 0.04534
## Cumulative Proportion  0.3244 0.4998 0.6469 0.7609 0.83094 0.89553 0.94087
##                            PC8     PC9   PC10    PC11    PC12
## Standard deviation     0.62954 0.43300 0.3304 0.11140 0.06479
## Proportion of Variance 0.03303 0.01562 0.0091 0.00103 0.00035
## Cumulative Proportion  0.97389 0.98952 0.9986 0.99965 1.00000
```

```
  #g <- ggbiplot(testOutput.pca, groups =fullDataSet[[3]]$SEQUENCE) + kimbi_theme() + scale_color_viridis_d() +labs(colour="SEQUENCE") + theme_light(base_size=15)
  #ggsave(filename = paste(finalGraphsFolderPathName, "PCAPlot-","output12.pdf", sep=""))
  #ggsave(filename = paste(finalGraphsFolderPathName, "PCAPlot-","output12.jpeg", sep=""), dpi=300)
```

## ML-Training

Now, we start the actual Ml-Training. Please note, that - as the
hyperparameter training is not deterministic - the results will slightly
vary for each run.

### Division of Training Data

We divide the data using the 3-way holdout. First 60% are for
hyperparameter training, 20% for testing the results, and 20% for
validating the chosen best models. We stratify by sequence.

```
  divideData <- function(data, train, test, validate){
    overallNumber <- dim(data)[1]
    sequenceTypes <- levels(data$SEQUENCE)
    sequencedData <- list()
    informationForData <- list()
    trainRows <- list()
    testRows <- list()
    validateRows <- list()
    for(i in 1:length(sequenceTypes)){
      sequenceData <- filter(fullDataSet[[3]], SEQUENCE==sequenceTypes[i])
      number <- dim(sequenceData)[1]
      information <- list(sequenceTypes[i], number, round(train*number), round(test*number), round(validate*number))
      informationForData[[i]] <- information
      sequencedData[[i]] <- sequenceData
      
      trainIDs <- sample(1:number, information[[3]], replace=F)
      remains <- setdiff(1:number, trainIDs)
      testIDs <- sample(remains, information[[4]], replace=F)
      validateIDs <- setdiff(remains, testIDs)
      
      trainRows[[i]] <- sequenceData[trainIDs,]
      testRows[[i]] <- sequenceData[testIDs,]
      validateRows[[i]] <- sequenceData[validateIDs,]
         
    }
    ###hacky region ahead - please revise when changes occur
    trainData <- bind_rows(trainRows[[1]], trainRows[[2]], trainRows[[3]], trainRows[[4]])
    testData <- bind_rows(testRows[[1]], testRows[[2]], testRows[[3]], testRows[[4]])
    validateData <- bind_rows(validateRows[[1]], validateRows[[2]], validateRows[[3]], validateRows[[4]])
    
    return (list(trainData, testData, validateData))
  }
  
  MLsepData <- divideData(fullDataSet[[3]], 0.6, 0.2, 0.2)
  trainingData <- MLsepData[[1]]

  trainingDataInput.pca <- prcomp(trainingData[, c(4:11, 13:14, 19)], center=TRUE, scale = TRUE)
  trainingDataOutput.pca <- prcomp(trainingData[,c(20:31)], center =TRUE, scale=TRUE)
  testData <- MLsepData[[2]]
  validationData <- MLsepData[[3]]
```

### Hyperparameter Training and ModelBuilding based on best learner

This is the central part. The function *testML* performs a
hyperparameter training on a given output variable with a given output
and a given measure. We specified hyperparameter-ranges for the
respective models. After identifying the best set of hyperparameters,
these are used for training (on the 60%) and directly tested on the
testing data. This function is then called for every output variable and
three different models. Therefore, we receive three results for every
output variable. The best of these three is then picked, used for
training and finally evaluated on the - up until then - unseen
validation data.

```
  testML <- function(indexOfOutput, nameOfOutput, nameOfLearner, measures){
    PredictionSet <- data.frame(trainingData[2], trainingData[4:11], trainingData[13:14], trainingData[19], trainingData[indexOfOutput])
    
    task = as_task_regr(PredictionSet[1:13], target = nameOfOutput)
    task$col_roles$stratum = "SEQUENCE"
    task$col_roles$feature <- c("EPI.FACTOR", "ETL", "READOUT.DURATION", "REFERENCE.LINES", "REFOCUSSING.ANGLE", "TE", "TR", "numberOfColumns", "numberOfRows", "MEASUREMENTS", "NUMBER.PRESCANS")
    rowIdsBSSFP <- task$strata[1]$row_id
    rowIdsSEEPI <- task$strata[2]$row_id
    rowIdsEPI <- task$strata[3]$row_id
    rowIdsRARE <- task$strata[4]$row_id
    
    keySequenceRowBSSFP <- data.frame(row_ids = rowIdsBSSFP[[1]], SEQUENCE= rep("BSSFP", length(rowIdsBSSFP[[1]])))
    keySequenceRowEPI <- data.frame(row_ids= rowIdsEPI[[1]], SEQUENCE=rep("EPI", length(rowIdsEPI[[1]])))
    keySequenceRowSEEPI <- data.frame(row_ids=rowIdsSEEPI[[1]], SEQUENCE=rep("SE-EPI", length(rowIdsSEEPI[[1]])))
    keySequenceRowRARE <- data.frame(row_ids =rowIdsRARE[[1]], SEQUENCE=rep("RARE", length(rowIdsRARE[[1]])))
    keySequenceRow <- bind_rows(keySequenceRowBSSFP, keySequenceRowEPI, keySequenceRowSEEPI, keySequenceRowRARE)
    
    
    learner_mrtprediction = lrn(nameOfLearner)
    
    if(nameOfLearner=="regr.svm"){
      
      hout=rsmp("cv")
      measure = msr("regr.rmse")
      search_space = ps(
        tolerance = p_dbl(lower=0.001, upper =0.9),
        gamma = p_dbl(lower=0.001, upper= 50.0),
        epsilon =p_dbl(lower=0.1, upper = 0.9), 
        type=p_fct(levels="eps-regression"), 
        kernel=p_fct(levels=c("radial", "sigmoid"))
      )
      
      eval50 = trm("evals", n_evals=10)
      
      instance = TuningInstanceSingleCrit$new(
        task=task, 
        learner = learner_mrtprediction, 
        resampling = hout, 
        measure = measure, 
        terminator = eval50, 
        search_space = search_space
      )
      tuner= tnr("grid_search", resolution = 15)
      
      tuner$optimize(instance)
      
      learner_mrtprediction$param_set$values = list(tolerance = instance$result_learner_param_vals[[1]], gamma =instance$result_learner_param_vals[[2]],epsilon = instance$result_learner_param_vals[[3]], type= "eps-regression", kernel = instance$result_learner_param_vals[[5]])
    }
    
    if(nameOfLearner=="regr.kknn"){
      hout=rsmp("cv")
      measure = msr("regr.rmse")
      search_space = ps(
        k = p_int(lower=1, upper =20),
        distance =p_dbl(lower=0.1, upper = 20), 
        
        kernel=p_fct(levels=c("rectangular", "triangular", "epanechnikov", "biweight", "triweight", "cos", "inv", "gaussian", "rank", "optimal"))
      )
      
      eval50 = trm("evals", n_evals=10)
      
      instance = TuningInstanceSingleCrit$new(
        task=task, 
        learner = learner_mrtprediction, 
        resampling = hout, 
        measure = measure, 
        terminator = eval50, 
        search_space = search_space
      )
      tuner= tnr("grid_search", resolution = 15)
      
      tuner$optimize(instance)
      
      learner_mrtprediction$param_set$values = list(k = instance$result_learner_param_vals[[1]], distance =instance$result_learner_param_vals[[2]], kernel= instance$result_learner_param_vals[[3]])
      
      
    }
    
    if(nameOfLearner=="regr.ranger"){
      hout=rsmp("cv")
      measure = msr("regr.rmse")
      search_space = ps(
        
        max.depth =p_int(lower=0, upper=20),
        num.trees = p_int(lower=1, upper=500) 
        
        
      )
      
      eval50 = trm("evals", n_evals=10)
      
      instance = TuningInstanceSingleCrit$new(
        task=task, 
        learner = learner_mrtprediction, 
        resampling = hout, 
        measure = measure, 
        terminator = eval50, 
        search_space = search_space
      )
      tuner= tnr("grid_search", resolution = 20)
      
      tuner$optimize(instance)
      
      learner_mrtprediction$param_set$values = list( max.depth =instance$result_learner_param_vals[[1]])
      
      
    }
    
    
    
    train_set =sample(task$row_ids, 0.67*task$nrow)
    test_set = setdiff(task$row_ids, train_set)
    
    learner_mrtprediction$train(task, row_ids=train_set)
    prediction=learner_mrtprediction$predict(task, row_ids=test_set)
    
    predictionResults <- data.frame(row_ids = prediction$row_ids, truth= prediction$truth, response = prediction$response)
    graphResult <- left_join(predictionResults, keySequenceRow, by="row_ids")
    
    ggplot(data=graphResult, aes(x=truth, y=response, color=SEQUENCE)) + geom_point() + geom_abline(slope=1, intercept= 0) + theme_light() + theme(legend.position = "top") + geom_abline(slope=1.1, intercept=0, linetype="dashed") + geom_abline(slope=0.9, intercept=0, linetype="dashed") + scale_color_viridis_d()
    #ggsave(filename = paste(nameOfOutput, nameOfLearner, "hyperTrain.pdf", sep=""))
    
    PredictionTestSet <- data.frame(testData[2], testData[4:11], testData[13:14], testData[19], testData[indexOfOutput])
    
    taskTest = as_task_regr(PredictionTestSet[1:13], target = nameOfOutput)
    taskTest$col_roles$stratum = "SEQUENCE"
    taskTest$col_roles$feature <- c("EPI.FACTOR", "ETL", "READOUT.DURATION", "REFERENCE.LINES", "REFOCUSSING.ANGLE", "TE", "TR", "numberOfColumns", "numberOfRows", "MEASUREMENTS", "NUMBER.PRESCANS")
    
    rowIdsBSSFPTest <- taskTest$strata[1]$row_id
    rowIdsSEEPITest <- taskTest$strata[2]$row_id
    rowIdsEPITest <- taskTest$strata[3]$row_id
    rowIdsRARETest <- taskTest$strata[4]$row_id
    
    keySequenceRowBSSFPTest <- data.frame(row_ids = rowIdsBSSFPTest[[1]], SEQUENCE= rep("BSSFP", length(rowIdsBSSFPTest[[1]])))
    keySequenceRowEPITest <- data.frame(row_ids= rowIdsEPITest[[1]], SEQUENCE=rep("EPI", length(rowIdsEPITest[[1]])))
    keySequenceRowSEEPITest <- data.frame(row_ids=rowIdsSEEPITest[[1]], SEQUENCE=rep("SE-EPI", length(rowIdsSEEPITest[[1]])))
    keySequenceRowRARETest <- data.frame(row_ids =rowIdsRARETest[[1]], SEQUENCE=rep("RARE", length(rowIdsRARETest[[1]])))
    keySequenceRowTest <- bind_rows(keySequenceRowBSSFPTest, keySequenceRowEPITest, keySequenceRowSEEPITest, keySequenceRowRARETest)
    
    learner_mrtprediction$train(task)
    predictionTest = learner_mrtprediction$predict(taskTest)
    
    
    
    predictionTestResults <- data.frame(row_ids = predictionTest$row_ids, truth= predictionTest$truth, response = predictionTest$response)
    graphResultTest <- left_join(predictionTestResults, keySequenceRowTest, by="row_ids")
    
    
    ggplot(data=graphResultTest, aes(x=truth, y=response, color=SEQUENCE)) + geom_point() + geom_abline(slope=1, intercept= 0) + theme_light() + theme(legend.position = "top") + geom_abline(slope=1.1, intercept=0, linetype="dashed") + geom_abline(slope=0.9, intercept=0, linetype="dashed") + scale_color_viridis_d()
    #ggsave(filename = paste(nameOfOutput, nameOfLearner, "hyperTest.pdf", sep=""))
    #ggsave(filename=paste(nameOfOutput, nameOfLearner, ".pdf"))
    
    
    return(list(prediction$score(measures), predictionTest$score(measures), learner_mrtprediction))
    
  }
  
  outputNumbers <- c(20, 21, 22, 23, 24, 25, 26, 27, 28, 29, 30, 31)
  outputNames <- c("GWC", "CWC", "CGC", "SNR_CSF", "SNR_GM", "SNR_WM", "GHOST", "SHARPNESS", "HOMOGENEITY", "MOTION.SENS", "DISTORTION.SENS", "TA")
  learnerNames <- c("regr.svm", "regr.kknn", "regr.ranger")
  N <- 100
  resultData <- data.frame(num=rep(NA, N), txt=rep("", N), txt=rep("", N), num=rep(NA, N), num=rep(NA, N), num=rep(NA, N), stringsAsFactors = FALSE)
  resultDataTest <- data.frame(num=rep(NA, N), txt=rep("", N), txt=rep("", N), num=rep(NA, N), num=rep(NA, N), num=rep(NA, N), stringsAsFactors = FALSE)
  resultDataValidation <- data.frame(num=rep(NA, N), txt=rep("", N), num=rep(NA, N), num=rep(NA, N), num=rep(NA, N), stringsAsFactors = FALSE)
  measures <- msrs(c("regr.rrse", "regr.rmse", "regr.rmsle"))
  
  MLresult <- list()
  
  for(i in 1:length(outputNumbers)){
    for(j in 1:length(learnerNames)){
    index <- (i-1)*length(learnerNames) + j
      result <- testML(outputNumbers[i], outputNames[i], learnerNames[j], measures)
     resultData[index, ] <- list(outputNumbers[i], outputNames[i], learnerNames[j], result[[1]][[1]], result[[1]][[2]], result[[1]][[3]])
      resultDataTest[index, ] <- list(outputNumbers[i], outputNames[i], learnerNames[j], result[[2]][[1]], result[[2]][[2]], result[[2]][[3]])
      
     if(j==1){
        bestLearner <- result
     }
      
      if(j>1){
        if(result[[2]][[2]] < bestLearner[[2]][[2]]){
         bestLearner <- result
        }
      }
    }
    MLresult[[outputNames[i]]] <- bestLearner
    
    PredictionSet <- data.frame(trainingData[2], trainingData[4:11], trainingData[13:14], trainingData[19], trainingData[outputNumbers[i]])
    
    task = as_task_regr(PredictionSet[1:13], target = outputNames[i])
    task$col_roles$stratum = "SEQUENCE"
    task$col_roles$feature <- c("EPI.FACTOR", "ETL", "READOUT.DURATION", "REFERENCE.LINES", "REFOCUSSING.ANGLE", "TE", "TR", "numberOfColumns", "numberOfRows", "MEASUREMENTS", "NUMBER.PRESCANS")
    
    PredictionValidationSet <- data.frame(validationData[2], validationData[4:11], validationData[13:14], validationData[19], validationData[outputNumbers[i]])
    
    taskValidation = as_task_regr(PredictionValidationSet[1:13], target = outputNames[i])
    taskValidation$col_roles$stratum = "SEQUENCE"
    taskValidation$col_roles$feature <- c("EPI.FACTOR", "ETL", "READOUT.DURATION", "REFERENCE.LINES", "REFOCUSSING.ANGLE", "TE", "TR", "numberOfColumns", "numberOfRows", "MEASUREMENTS", "NUMBER.PRESCANS")
    
    rowIdsBSSFPValidation <- taskValidation$strata[1]$row_id
    rowIdsSEEPIValidation <- taskValidation$strata[2]$row_id
    rowIdsEPIValidation <- taskValidation$strata[3]$row_id
    rowIdsRAREValidation <- taskValidation$strata[4]$row_id
    
    keySequenceRowBSSFPValidation <- data.frame(row_ids = rowIdsBSSFPValidation[[1]], SEQUENCE= rep("BSSFP", length(rowIdsBSSFPValidation[[1]])))
    keySequenceRowEPIValidation <- data.frame(row_ids= rowIdsEPIValidation[[1]], SEQUENCE=rep("EPI", length(rowIdsEPIValidation[[1]])))
    keySequenceRowSEEPIValidation <- data.frame(row_ids=rowIdsSEEPIValidation[[1]], SEQUENCE=rep("SE-EPI", length(rowIdsSEEPIValidation[[1]])))
    keySequenceRowRAREValidation <- data.frame(row_ids =rowIdsRAREValidation[[1]], SEQUENCE=rep("RARE", length(rowIdsRAREValidation[[1]])))
    keySequenceRowValidation <- bind_rows(keySequenceRowBSSFPValidation, keySequenceRowEPIValidation, keySequenceRowSEEPIValidation, keySequenceRowRAREValidation)
    
    bestLearner[[3]]$train(task)
    predictionValidation = bestLearner[[3]]$predict(taskValidation)
    
    
    resultDataValidation[i, ] <- list(outputNumbers[i], outputNames[i],  predictionValidation$score(measures)[[1]], predictionValidation$score(measures)[[2]], predictionValidation$score(measures)[[3]])
    predictionValidationResults <- data.frame(row_ids = predictionValidation$row_ids, truth= predictionValidation$truth, response = predictionValidation$response)
    graphResultValidation <- left_join(predictionValidationResults, keySequenceRowValidation, by="row_ids")
    
    
    ggplot(data=graphResultValidation, aes(x=truth, y=response, color=SEQUENCE)) + geom_point() + geom_abline(slope=1, intercept= 0) + coord_cartesian(xlim=c(0, NA), ylim=c(0, NA)) + theme_light(base_size=13) + theme(legend.position = "top") + geom_abline(slope=1.1, intercept=0, linetype="dashed") + geom_abline(slope=0.9, intercept=0, linetype="dashed") + scale_color_viridis_d()
    #ggsave(filename = paste(finalGraphsFolderPathName, outputNames[i], "Validation.pdf", sep=""), width  = 13, unit="cm" )
    #ggsave(filename = paste(finalGraphsFolderPathName, outputNames[i], "Validation.jpeg", sep=""), dpi=300)
    
  }
```

```
## INFO  [08:01:58.717] [bbotk] Starting to optimize 5 parameter(s) with '<TunerGridSearch>' and '<TerminatorEvals> [n_evals=10, k=0]'
## INFO  [08:01:58.750] [bbotk] Evaluating 1 configuration(s)
## INFO  [08:01:58.783] [mlr3] Running benchmark with 10 resampling iterations
## INFO  [08:01:58.821] [mlr3] Applying learner 'regr.svm' on task 'PredictionSet[1:13]' (iter 1/10)
## INFO  [08:01:58.855] [mlr3] Applying learner 'regr.svm' on task 'PredictionSet[1:13]' (iter 2/10)
## INFO  [08:01:58.872] [mlr3] Applying learner 'regr.svm' on task 'PredictionSet[1:13]' (iter 3/10)
## INFO  [08:01:58.889] [mlr3] Applying learner 'regr.svm' on task 'PredictionSet[1:13]' (iter 4/10)
## INFO  [08:01:58.906] [mlr3] Applying learner 'regr.svm' on task 'PredictionSet[1:13]' (iter 5/10)
## INFO  [08:01:58.924] [mlr3] Applying learner 'regr.svm' on task 'PredictionSet[1:13]' (iter 6/10)
## INFO  [08:01:58.943] [mlr3] Applying learner 'regr.svm' on task 'PredictionSet[1:13]' (iter 7/10)
## INFO  [08:01:58.961] [mlr3] Applying learner 'regr.svm' on task 'PredictionSet[1:13]' (iter 8/10)
## INFO  [08:01:58.980] [mlr3] Applying learner 'regr.svm' on task 'PredictionSet[1:13]' (iter 9/10)
## INFO  [08:01:59.000] [mlr3] Applying learner 'regr.svm' on task 'PredictionSet[1:13]' (iter 10/10)
## INFO  [08:01:59.024] [mlr3] Finished benchmark
## INFO  [08:01:59.069] [bbotk] Result of batch 1:
## INFO  [08:01:59.072] [bbotk]  tolerance    gamma   epsilon           type kernel regr.rmse warnings errors
## INFO  [08:01:59.072] [bbotk]  0.5147143 46.42864 0.5571429 eps-regression radial 0.2593254        0      0
## INFO  [08:01:59.072] [bbotk]  runtime_learners                                uhash
## INFO  [08:01:59.072] [bbotk]             0.077 71926f9b-cb94-44b1-a0c5-44bdfc35d074
## INFO  [08:01:59.074] [bbotk] Evaluating 1 configuration(s)
## INFO  [08:01:59.091] [mlr3] Running benchmark with 10 resampling iterations
## INFO  [08:01:59.096] [mlr3] Applying learner 'regr.svm' on task 'PredictionSet[1:13]' (iter 1/10)
## INFO  [08:01:59.116] [mlr3] Applying learner 'regr.svm' on task 'PredictionSet[1:13]' (iter 2/10)
## INFO  [08:01:59.135] [mlr3] Applying learner 'regr.svm' on task 'PredictionSet[1:13]' (iter 3/10)
## INFO  [08:01:59.155] [mlr3] Applying learner 'regr.svm' on task 'PredictionSet[1:13]' (iter 4/10)
## INFO  [08:01:59.173] [mlr3] Applying learner 'regr.svm' on task 'PredictionSet[1:13]' (iter 5/10)
## INFO  [08:01:59.191] [mlr3] Applying learner 'regr.svm' on task 'PredictionSet[1:13]' (iter 6/10)
## INFO  [08:01:59.209] [mlr3] Applying learner 'regr.svm' on task 'PredictionSet[1:13]' (iter 7/10)
## INFO  [08:01:59.229] [mlr3] Applying learner 'regr.svm' on task 'PredictionSet[1:13]' (iter 8/10)
## INFO  [08:01:59.247] [mlr3] Applying learner 'regr.svm' on task 'PredictionSet[1:13]' (iter 9/10)
## INFO  [08:01:59.266] [mlr3] Applying learner 'regr.svm' on task 'PredictionSet[1:13]' (iter 10/10)
## INFO  [08:01:59.290] [mlr3] Finished benchmark
## INFO  [08:01:59.323] [bbotk] Result of batch 2:
## INFO  [08:01:59.324] [bbotk]  tolerance    gamma   epsilon           type  kernel regr.rmse warnings errors
## INFO  [08:01:59.324] [bbotk]  0.8357857 32.14321 0.2714286 eps-regression sigmoid  10.10583        0      0
## INFO  [08:01:59.324] [bbotk]  runtime_learners                                uhash
## INFO  [08:01:59.324] [bbotk]             0.081 3dc3adaa-d834-4230-8a2e-7d3b8d18de85
## INFO  [08:01:59.326] [bbotk] Evaluating 1 configuration(s)
## INFO  [08:01:59.341] [mlr3] Running benchmark with 10 resampling iterations
## INFO  [08:01:59.347] [mlr3] Applying learner 'regr.svm' on task 'PredictionSet[1:13]' (iter 1/10)
## INFO  [08:01:59.365] [mlr3] Applying learner 'regr.svm' on task 'PredictionSet[1:13]' (iter 2/10)
## INFO  [08:01:59.385] [mlr3] Applying learner 'regr.svm' on task 'PredictionSet[1:13]' (iter 3/10)
## INFO  [08:01:59.403] [mlr3] Applying learner 'regr.svm' on task 'PredictionSet[1:13]' (iter 4/10)
## INFO  [08:01:59.421] [mlr3] Applying learner 'regr.svm' on task 'PredictionSet[1:13]' (iter 5/10)
## INFO  [08:01:59.441] [mlr3] Applying learner 'regr.svm' on task 'PredictionSet[1:13]' (iter 6/10)
## INFO  [08:01:59.460] [mlr3] Applying learner 'regr.svm' on task 'PredictionSet[1:13]' (iter 7/10)
## INFO  [08:01:59.478] [mlr3] Applying learner 'regr.svm' on task 'PredictionSet[1:13]' (iter 8/10)
## INFO  [08:01:59.501] [mlr3] Applying learner 'regr.svm' on task 'PredictionSet[1:13]' (iter 9/10)
## INFO  [08:01:59.520] [mlr3] Applying learner 'regr.svm' on task 'PredictionSet[1:13]' (iter 10/10)
## INFO  [08:01:59.539] [mlr3] Finished benchmark
## INFO  [08:01:59.570] [bbotk] Result of batch 3:
## INFO  [08:01:59.571] [bbotk]  tolerance    gamma epsilon           type  kernel regr.rmse warnings errors
## INFO  [08:01:59.571] [bbotk]  0.7715714 46.42864     0.9 eps-regression sigmoid  10.02131        0      0
## INFO  [08:01:59.571] [bbotk]  runtime_learners                                uhash
## INFO  [08:01:59.571] [bbotk]             0.082 55905fd2-1ee3-45d4-9d98-b9cc574b3197
## INFO  [08:01:59.573] [bbotk] Evaluating 1 configuration(s)
## INFO  [08:01:59.589] [mlr3] Running benchmark with 10 resampling iterations
## INFO  [08:01:59.594] [mlr3] Applying learner 'regr.svm' on task 'PredictionSet[1:13]' (iter 1/10)
## INFO  [08:01:59.610] [mlr3] Applying learner 'regr.svm' on task 'PredictionSet[1:13]' (iter 2/10)
## INFO  [08:01:59.625] [mlr3] Applying learner 'regr.svm' on task 'PredictionSet[1:13]' (iter 3/10)
## INFO  [08:01:59.641] [mlr3] Applying learner 'regr.svm' on task 'PredictionSet[1:13]' (iter 4/10)
## INFO  [08:01:59.657] [mlr3] Applying learner 'regr.svm' on task 'PredictionSet[1:13]' (iter 5/10)
## INFO  [08:01:59.675] [mlr3] Applying learner 'regr.svm' on task 'PredictionSet[1:13]' (iter 6/10)
## INFO  [08:01:59.691] [mlr3] Applying learner 'regr.svm' on task 'PredictionSet[1:13]' (iter 7/10)
## INFO  [08:01:59.713] [mlr3] Applying learner 'regr.svm' on task 'PredictionSet[1:13]' (iter 8/10)
## INFO  [08:01:59.730] [mlr3] Applying learner 'regr.svm' on task 'PredictionSet[1:13]' (iter 9/10)
## INFO  [08:01:59.748] [mlr3] Applying learner 'regr.svm' on task 'PredictionSet[1:13]' (iter 10/10)
## INFO  [08:01:59.766] [mlr3] Finished benchmark
## INFO  [08:01:59.800] [bbotk] Result of batch 4:
## INFO  [08:01:59.802] [bbotk]  tolerance gamma   epsilon           type kernel regr.rmse warnings errors
## INFO  [08:01:59.802] [bbotk]  0.6431429    50 0.6714286 eps-regression radial 0.2707132        0      0
## INFO  [08:01:59.802] [bbotk]  runtime_learners                                uhash
## INFO  [08:01:59.802] [bbotk]              0.06 362487e3-6556-4649-8035-38981fde2de8
## INFO  [08:01:59.803] [bbotk] Evaluating 1 configuration(s)
## INFO  [08:01:59.820] [mlr3] Running benchmark with 10 resampling iterations
## INFO  [08:01:59.825] [mlr3] Applying learner 'regr.svm' on task 'PredictionSet[1:13]' (iter 1/10)
## INFO  [08:01:59.842] [mlr3] Applying learner 'regr.svm' on task 'PredictionSet[1:13]' (iter 2/10)
## INFO  [08:01:59.858] [mlr3] Applying learner 'regr.svm' on task 'PredictionSet[1:13]' (iter 3/10)
## INFO  [08:01:59.874] [mlr3] Applying learner 'regr.svm' on task 'PredictionSet[1:13]' (iter 4/10)
## INFO  [08:01:59.893] [mlr3] Applying learner 'regr.svm' on task 'PredictionSet[1:13]' (iter 5/10)
## INFO  [08:01:59.915] [mlr3] Applying learner 'regr.svm' on task 'PredictionSet[1:13]' (iter 6/10)
## INFO  [08:01:59.932] [mlr3] Applying learner 'regr.svm' on task 'PredictionSet[1:13]' (iter 7/10)
## INFO  [08:01:59.948] [mlr3] Applying learner 'regr.svm' on task 'PredictionSet[1:13]' (iter 8/10)
## INFO  [08:01:59.966] [mlr3] Applying learner 'regr.svm' on task 'PredictionSet[1:13]' (iter 9/10)
## INFO  [08:01:59.983] [mlr3] Applying learner 'regr.svm' on task 'PredictionSet[1:13]' (iter 10/10)
## INFO  [08:01:59.998] [mlr3] Finished benchmark
## INFO  [08:02:00.030] [bbotk] Result of batch 5:
## INFO  [08:02:00.032] [bbotk]  tolerance    gamma   epsilon           type kernel regr.rmse warnings errors
## INFO  [08:02:00.032] [bbotk]  0.3862857 3.572357 0.3857143 eps-regression radial 0.2239294        0      0
## INFO  [08:02:00.032] [bbotk]  runtime_learners                                uhash
## INFO  [08:02:00.032] [bbotk]              0.07 99a4ec4c-d65e-4e5a-b9bb-6b17ee8f6bd3
## INFO  [08:02:00.034] [bbotk] Evaluating 1 configuration(s)
## INFO  [08:02:00.050] [mlr3] Running benchmark with 10 resampling iterations
## INFO  [08:02:00.055] [mlr3] Applying learner 'regr.svm' on task 'PredictionSet[1:13]' (iter 1/10)
## INFO  [08:02:00.072] [mlr3] Applying learner 'regr.svm' on task 'PredictionSet[1:13]' (iter 2/10)
## INFO  [08:02:00.095] [mlr3] Applying learner 'regr.svm' on task 'PredictionSet[1:13]' (iter 3/10)
## INFO  [08:02:00.113] [mlr3] Applying learner 'regr.svm' on task 'PredictionSet[1:13]' (iter 4/10)
## INFO  [08:02:00.132] [mlr3] Applying learner 'regr.svm' on task 'PredictionSet[1:13]' (iter 5/10)
## INFO  [08:02:00.151] [mlr3] Applying learner 'regr.svm' on task 'PredictionSet[1:13]' (iter 6/10)
## INFO  [08:02:00.169] [mlr3] Applying learner 'regr.svm' on task 'PredictionSet[1:13]' (iter 7/10)
## INFO  [08:02:00.188] [mlr3] Applying learner 'regr.svm' on task 'PredictionSet[1:13]' (iter 8/10)
## INFO  [08:02:00.206] [mlr3] Applying learner 'regr.svm' on task 'PredictionSet[1:13]' (iter 9/10)
## INFO  [08:02:00.226] [mlr3] Applying learner 'regr.svm' on task 'PredictionSet[1:13]' (iter 10/10)
## INFO  [08:02:00.243] [mlr3] Finished benchmark
## INFO  [08:02:00.282] [bbotk] Result of batch 6:
## INFO  [08:02:00.284] [bbotk]  tolerance gamma   epsilon           type kernel regr.rmse warnings errors
## INFO  [08:02:00.284] [bbotk]  0.6431429    50 0.2714286 eps-regression radial 0.2541768        0      0
## INFO  [08:02:00.284] [bbotk]  runtime_learners                                uhash
## INFO  [08:02:00.284] [bbotk]             0.082 9a74a349-ac22-4304-b033-3b8390f1746e
## INFO  [08:02:00.285] [bbotk] Evaluating 1 configuration(s)
## INFO  [08:02:00.301] [mlr3] Running benchmark with 10 resampling iterations
## INFO  [08:02:00.306] [mlr3] Applying learner 'regr.svm' on task 'PredictionSet[1:13]' (iter 1/10)
## INFO  [08:02:00.323] [mlr3] Applying learner 'regr.svm' on task 'PredictionSet[1:13]' (iter 2/10)
## INFO  [08:02:00.341] [mlr3] Applying learner 'regr.svm' on task 'PredictionSet[1:13]' (iter 3/10)
## INFO  [08:02:00.358] [mlr3] Applying learner 'regr.svm' on task 'PredictionSet[1:13]' (iter 4/10)
## INFO  [08:02:00.375] [mlr3] Applying learner 'regr.svm' on task 'PredictionSet[1:13]' (iter 5/10)
## INFO  [08:02:00.391] [mlr3] Applying learner 'regr.svm' on task 'PredictionSet[1:13]' (iter 6/10)
## INFO  [08:02:00.409] [mlr3] Applying learner 'regr.svm' on task 'PredictionSet[1:13]' (iter 7/10)
## INFO  [08:02:00.427] [mlr3] Applying learner 'regr.svm' on task 'PredictionSet[1:13]' (iter 8/10)
## INFO  [08:02:00.444] [mlr3] Applying learner 'regr.svm' on task 'PredictionSet[1:13]' (iter 9/10)
## INFO  [08:02:00.461] [mlr3] Applying learner 'regr.svm' on task 'PredictionSet[1:13]' (iter 10/10)
## INFO  [08:02:00.483] [mlr3] Finished benchmark
## INFO  [08:02:00.515] [bbotk] Result of batch 7:
## INFO  [08:02:00.517] [bbotk]  tolerance gamma   epsilon           type kernel regr.rmse warnings errors
## INFO  [08:02:00.517] [bbotk]  0.8357857 0.001 0.1571429 eps-regression radial 0.2804834        0      0
## INFO  [08:02:00.517] [bbotk]  runtime_learners                                uhash
## INFO  [08:02:00.517] [bbotk]             0.073 9fb49c64-d146-4c5e-a22a-bf1a7ecaff56
## INFO  [08:02:00.518] [bbotk] Evaluating 1 configuration(s)
## INFO  [08:02:00.533] [mlr3] Running benchmark with 10 resampling iterations
## INFO  [08:02:00.538] [mlr3] Applying learner 'regr.svm' on task 'PredictionSet[1:13]' (iter 1/10)
## INFO  [08:02:00.558] [mlr3] Applying learner 'regr.svm' on task 'PredictionSet[1:13]' (iter 2/10)
## INFO  [08:02:00.578] [mlr3] Applying learner 'regr.svm' on task 'PredictionSet[1:13]' (iter 3/10)
## INFO  [08:02:00.597] [mlr3] Applying learner 'regr.svm' on task 'PredictionSet[1:13]' (iter 4/10)
## INFO  [08:02:00.620] [mlr3] Applying learner 'regr.svm' on task 'PredictionSet[1:13]' (iter 5/10)
## INFO  [08:02:00.642] [mlr3] Applying learner 'regr.svm' on task 'PredictionSet[1:13]' (iter 6/10)
## INFO  [08:02:00.662] [mlr3] Applying learner 'regr.svm' on task 'PredictionSet[1:13]' (iter 7/10)
## INFO  [08:02:00.686] [mlr3] Applying learner 'regr.svm' on task 'PredictionSet[1:13]' (iter 8/10)
## INFO  [08:02:00.707] [mlr3] Applying learner 'regr.svm' on task 'PredictionSet[1:13]' (iter 9/10)
## INFO  [08:02:00.727] [mlr3] Applying learner 'regr.svm' on task 'PredictionSet[1:13]' (iter 10/10)
## INFO  [08:02:00.745] [mlr3] Finished benchmark
## INFO  [08:02:00.776] [bbotk] Result of batch 8:
## INFO  [08:02:00.778] [bbotk]   tolerance    gamma   epsilon           type  kernel regr.rmse warnings errors
## INFO  [08:02:00.778] [bbotk]  0.06521429 7.143714 0.5571429 eps-regression sigmoid  10.13325        0      0
## INFO  [08:02:00.778] [bbotk]  runtime_learners                                uhash
## INFO  [08:02:00.778] [bbotk]               0.1 8b166faf-99d0-4b30-9144-8a09478a30ca
## INFO  [08:02:00.779] [bbotk] Evaluating 1 configuration(s)
## INFO  [08:02:00.796] [mlr3] Running benchmark with 10 resampling iterations
## INFO  [08:02:00.801] [mlr3] Applying learner 'regr.svm' on task 'PredictionSet[1:13]' (iter 1/10)
## INFO  [08:02:00.819] [mlr3] Applying learner 'regr.svm' on task 'PredictionSet[1:13]' (iter 2/10)
## INFO  [08:02:00.880] [mlr3] Applying learner 'regr.svm' on task 'PredictionSet[1:13]' (iter 3/10)
## INFO  [08:02:00.903] [mlr3] Applying learner 'regr.svm' on task 'PredictionSet[1:13]' (iter 4/10)
## INFO  [08:02:00.922] [mlr3] Applying learner 'regr.svm' on task 'PredictionSet[1:13]' (iter 5/10)
## INFO  [08:02:00.942] [mlr3] Applying learner 'regr.svm' on task 'PredictionSet[1:13]' (iter 6/10)
## INFO  [08:02:00.960] [mlr3] Applying learner 'regr.svm' on task 'PredictionSet[1:13]' (iter 7/10)
## INFO  [08:02:00.978] [mlr3] Applying learner 'regr.svm' on task 'PredictionSet[1:13]' (iter 8/10)
## INFO  [08:02:00.996] [mlr3] Applying learner 'regr.svm' on task 'PredictionSet[1:13]' (iter 9/10)
## INFO  [08:02:01.016] [mlr3] Applying learner 'regr.svm' on task 'PredictionSet[1:13]' (iter 10/10)
## INFO  [08:02:01.034] [mlr3] Finished benchmark
## INFO  [08:02:01.065] [bbotk] Result of batch 9:
## INFO  [08:02:01.066] [bbotk]  tolerance    gamma epsilon           type  kernel regr.rmse warnings errors
## INFO  [08:02:01.066] [bbotk]  0.8357857 46.42864     0.9 eps-regression sigmoid  10.02131        0      0
## INFO  [08:02:01.066] [bbotk]  runtime_learners                                uhash
## INFO  [08:02:01.066] [bbotk]             0.087 32ee2266-ceb8-4882-bfb4-2bd134c3af91
## INFO  [08:02:01.068] [bbotk] Evaluating 1 configuration(s)
## INFO  [08:02:01.085] [mlr3] Running benchmark with 10 resampling iterations
## INFO  [08:02:01.090] [mlr3] Applying learner 'regr.svm' on task 'PredictionSet[1:13]' (iter 1/10)
## INFO  [08:02:01.109] [mlr3] Applying learner 'regr.svm' on task 'PredictionSet[1:13]' (iter 2/10)
## INFO  [08:02:01.134] [mlr3] Applying learner 'regr.svm' on task 'PredictionSet[1:13]' (iter 3/10)
## INFO  [08:02:01.153] [mlr3] Applying learner 'regr.svm' on task 'PredictionSet[1:13]' (iter 4/10)
## INFO  [08:02:01.172] [mlr3] Applying learner 'regr.svm' on task 'PredictionSet[1:13]' (iter 5/10)
## INFO  [08:02:01.190] [mlr3] Applying learner 'regr.svm' on task 'PredictionSet[1:13]' (iter 6/10)
## INFO  [08:02:01.208] [mlr3] Applying learner 'regr.svm' on task 'PredictionSet[1:13]' (iter 7/10)
## INFO  [08:02:01.227] [mlr3] Applying learner 'regr.svm' on task 'PredictionSet[1:13]' (iter 8/10)
## INFO  [08:02:01.246] [mlr3] Applying learner 'regr.svm' on task 'PredictionSet[1:13]' (iter 9/10)
## INFO  [08:02:01.265] [mlr3] Applying learner 'regr.svm' on task 'PredictionSet[1:13]' (iter 10/10)
## INFO  [08:02:01.283] [mlr3] Finished benchmark
## INFO  [08:02:01.318] [bbotk] Result of batch 10:
## INFO  [08:02:01.320] [bbotk]  tolerance   gamma   epsilon           type  kernel regr.rmse warnings errors
## INFO  [08:02:01.320] [bbotk]  0.7073571 25.0005 0.4428571 eps-regression sigmoid  9.915723        0      0
## INFO  [08:02:01.320] [bbotk]  runtime_learners                                uhash
## INFO  [08:02:01.320] [bbotk]             0.088 f56c686c-7012-456a-ac5e-f1e013dc1b35
## INFO  [08:02:01.327] [bbotk] Finished optimizing after 10 evaluation(s)
## INFO  [08:02:01.327] [bbotk] Result:
## INFO  [08:02:01.329] [bbotk]  tolerance    gamma   epsilon           type kernel learner_param_vals
## INFO  [08:02:01.329] [bbotk]  0.3862857 3.572357 0.3857143 eps-regression radial          <list[5]>
## INFO  [08:02:01.329] [bbotk]   x_domain regr.rmse
## INFO  [08:02:01.329] [bbotk]  <list[5]> 0.2239294
## INFO  [08:02:01.493] [bbotk] Starting to optimize 3 parameter(s) with '<TunerGridSearch>' and '<TerminatorEvals> [n_evals=10, k=0]'
## INFO  [08:02:01.499] [bbotk] Evaluating 1 configuration(s)
## INFO  [08:02:01.520] [mlr3] Running benchmark with 10 resampling iterations
## INFO  [08:02:01.527] [mlr3] Applying learner 'regr.kknn' on task 'PredictionSet[1:13]' (iter 1/10)
## INFO  [08:02:02.458] [mlr3] Applying learner 'regr.kknn' on task 'PredictionSet[1:13]' (iter 2/10)
## INFO  [08:02:02.479] [mlr3] Applying learner 'regr.kknn' on task 'PredictionSet[1:13]' (iter 3/10)
## INFO  [08:02:02.500] [mlr3] Applying learner 'regr.kknn' on task 'PredictionSet[1:13]' (iter 4/10)
## INFO  [08:02:02.524] [mlr3] Applying learner 'regr.kknn' on task 'PredictionSet[1:13]' (iter 5/10)
## INFO  [08:02:02.547] [mlr3] Applying learner 'regr.kknn' on task 'PredictionSet[1:13]' (iter 6/10)
## INFO  [08:02:02.569] [mlr3] Applying learner 'regr.kknn' on task 'PredictionSet[1:13]' (iter 7/10)
## INFO  [08:02:02.594] [mlr3] Applying learner 'regr.kknn' on task 'PredictionSet[1:13]' (iter 8/10)
## INFO  [08:02:02.617] [mlr3] Applying learner 'regr.kknn' on task 'PredictionSet[1:13]' (iter 9/10)
## INFO  [08:02:02.640] [mlr3] Applying learner 'regr.kknn' on task 'PredictionSet[1:13]' (iter 10/10)
## INFO  [08:02:02.664] [mlr3] Finished benchmark
## INFO  [08:02:02.710] [bbotk] Result of batch 1:
## INFO  [08:02:02.712] [bbotk]  k distance   kernel regr.rmse warnings errors runtime_learners
## INFO  [08:02:02.712] [bbotk]  2 14.31429 gaussian 0.1462348        0      0            0.114
## INFO  [08:02:02.712] [bbotk]                                 uhash
## INFO  [08:02:02.712] [bbotk]  174d2977-43ee-4736-8d19-3f4a311fd8ce
## INFO  [08:02:02.714] [bbotk] Evaluating 1 configuration(s)
## INFO  [08:02:02.725] [mlr3] Running benchmark with 10 resampling iterations
## INFO  [08:02:02.730] [mlr3] Applying learner 'regr.kknn' on task 'PredictionSet[1:13]' (iter 1/10)
## INFO  [08:02:02.753] [mlr3] Applying learner 'regr.kknn' on task 'PredictionSet[1:13]' (iter 2/10)
## INFO  [08:02:02.774] [mlr3] Applying learner 'regr.kknn' on task 'PredictionSet[1:13]' (iter 3/10)
## INFO  [08:02:02.795] [mlr3] Applying learner 'regr.kknn' on task 'PredictionSet[1:13]' (iter 4/10)
## INFO  [08:02:02.816] [mlr3] Applying learner 'regr.kknn' on task 'PredictionSet[1:13]' (iter 5/10)
## INFO  [08:02:02.838] [mlr3] Applying learner 'regr.kknn' on task 'PredictionSet[1:13]' (iter 6/10)
## INFO  [08:02:02.859] [mlr3] Applying learner 'regr.kknn' on task 'PredictionSet[1:13]' (iter 7/10)
## INFO  [08:02:02.879] [mlr3] Applying learner 'regr.kknn' on task 'PredictionSet[1:13]' (iter 8/10)
## INFO  [08:02:02.902] [mlr3] Applying learner 'regr.kknn' on task 'PredictionSet[1:13]' (iter 9/10)
## INFO  [08:02:02.923] [mlr3] Applying learner 'regr.kknn' on task 'PredictionSet[1:13]' (iter 10/10)
## INFO  [08:02:02.944] [mlr3] Finished benchmark
## INFO  [08:02:02.982] [bbotk] Result of batch 2:
## INFO  [08:02:02.984] [bbotk]  k distance      kernel regr.rmse warnings errors runtime_learners
## INFO  [08:02:02.984] [bbotk]  6    10.05 rectangular 0.1971575        0      0            0.108
## INFO  [08:02:02.984] [bbotk]                                 uhash
## INFO  [08:02:02.984] [bbotk]  c38adfa9-620b-49cd-80a1-f33f06689baf
## INFO  [08:02:02.985] [bbotk] Evaluating 1 configuration(s)
## INFO  [08:02:02.996] [mlr3] Running benchmark with 10 resampling iterations
## INFO  [08:02:03.001] [mlr3] Applying learner 'regr.kknn' on task 'PredictionSet[1:13]' (iter 1/10)
## INFO  [08:02:03.022] [mlr3] Applying learner 'regr.kknn' on task 'PredictionSet[1:13]' (iter 2/10)
## INFO  [08:02:03.045] [mlr3] Applying learner 'regr.kknn' on task 'PredictionSet[1:13]' (iter 3/10)
## INFO  [08:02:03.066] [mlr3] Applying learner 'regr.kknn' on task 'PredictionSet[1:13]' (iter 4/10)
## INFO  [08:02:03.088] [mlr3] Applying learner 'regr.kknn' on task 'PredictionSet[1:13]' (iter 5/10)
## INFO  [08:02:03.118] [mlr3] Applying learner 'regr.kknn' on task 'PredictionSet[1:13]' (iter 6/10)
## INFO  [08:02:03.140] [mlr3] Applying learner 'regr.kknn' on task 'PredictionSet[1:13]' (iter 7/10)
## INFO  [08:02:03.160] [mlr3] Applying learner 'regr.kknn' on task 'PredictionSet[1:13]' (iter 8/10)
## INFO  [08:02:03.181] [mlr3] Applying learner 'regr.kknn' on task 'PredictionSet[1:13]' (iter 9/10)
## INFO  [08:02:03.203] [mlr3] Applying learner 'regr.kknn' on task 'PredictionSet[1:13]' (iter 10/10)
## INFO  [08:02:03.223] [mlr3] Finished benchmark
## INFO  [08:02:03.255] [bbotk] Result of batch 3:
## INFO  [08:02:03.257] [bbotk]  k distance     kernel regr.rmse warnings errors runtime_learners
## INFO  [08:02:03.257] [bbotk]  5 4.364286 triangular 0.1685129        0      0            0.105
## INFO  [08:02:03.257] [bbotk]                                 uhash
## INFO  [08:02:03.257] [bbotk]  a831ca7e-e2f6-48e8-9d86-45e247c8fa7d
## INFO  [08:02:03.259] [bbotk] Evaluating 1 configuration(s)
## INFO  [08:02:03.270] [mlr3] Running benchmark with 10 resampling iterations
## INFO  [08:02:03.275] [mlr3] Applying learner 'regr.kknn' on task 'PredictionSet[1:13]' (iter 1/10)
## INFO  [08:02:03.295] [mlr3] Applying learner 'regr.kknn' on task 'PredictionSet[1:13]' (iter 2/10)
## INFO  [08:02:03.314] [mlr3] Applying learner 'regr.kknn' on task 'PredictionSet[1:13]' (iter 3/10)
## INFO  [08:02:03.336] [mlr3] Applying learner 'regr.kknn' on task 'PredictionSet[1:13]' (iter 4/10)
## INFO  [08:02:03.357] [mlr3] Applying learner 'regr.kknn' on task 'PredictionSet[1:13]' (iter 5/10)
## INFO  [08:02:03.377] [mlr3] Applying learner 'regr.kknn' on task 'PredictionSet[1:13]' (iter 6/10)
## INFO  [08:02:03.398] [mlr3] Applying learner 'regr.kknn' on task 'PredictionSet[1:13]' (iter 7/10)
## INFO  [08:02:03.418] [mlr3] Applying learner 'regr.kknn' on task 'PredictionSet[1:13]' (iter 8/10)
## INFO  [08:02:03.440] [mlr3] Applying learner 'regr.kknn' on task 'PredictionSet[1:13]' (iter 9/10)
## INFO  [08:02:03.463] [mlr3] Applying learner 'regr.kknn' on task 'PredictionSet[1:13]' (iter 10/10)
## INFO  [08:02:03.484] [mlr3] Finished benchmark
## INFO  [08:02:03.526] [bbotk] Result of batch 4:
## INFO  [08:02:03.527] [bbotk]   k distance kernel regr.rmse warnings errors runtime_learners
## INFO  [08:02:03.527] [bbotk]  20 17.15714    cos 0.1874314        0      0            0.103
## INFO  [08:02:03.527] [bbotk]                                 uhash
## INFO  [08:02:03.527] [bbotk]  1acd5f5f-e25a-4e7c-8d3b-0c6129fff650
## INFO  [08:02:03.529] [bbotk] Evaluating 1 configuration(s)
## INFO  [08:02:03.542] [mlr3] Running benchmark with 10 resampling iterations
## INFO  [08:02:03.549] [mlr3] Applying learner 'regr.kknn' on task 'PredictionSet[1:13]' (iter 1/10)
## INFO  [08:02:03.580] [mlr3] Applying learner 'regr.kknn' on task 'PredictionSet[1:13]' (iter 2/10)
## INFO  [08:02:03.610] [mlr3] Applying learner 'regr.kknn' on task 'PredictionSet[1:13]' (iter 3/10)
## INFO  [08:02:03.638] [mlr3] Applying learner 'regr.kknn' on task 'PredictionSet[1:13]' (iter 4/10)
## INFO  [08:02:03.670] [mlr3] Applying learner 'regr.kknn' on task 'PredictionSet[1:13]' (iter 5/10)
## INFO  [08:02:03.701] [mlr3] Applying learner 'regr.kknn' on task 'PredictionSet[1:13]' (iter 6/10)
## INFO  [08:02:03.732] [mlr3] Applying learner 'regr.kknn' on task 'PredictionSet[1:13]' (iter 7/10)
## INFO  [08:02:03.760] [mlr3] Applying learner 'regr.kknn' on task 'PredictionSet[1:13]' (iter 8/10)
## INFO  [08:02:03.789] [mlr3] Applying learner 'regr.kknn' on task 'PredictionSet[1:13]' (iter 9/10)
## INFO  [08:02:03.816] [mlr3] Applying learner 'regr.kknn' on task 'PredictionSet[1:13]' (iter 10/10)
## INFO  [08:02:03.843] [mlr3] Finished benchmark
## INFO  [08:02:03.882] [bbotk] Result of batch 5:
## INFO  [08:02:03.885] [bbotk]   k distance  kernel regr.rmse warnings errors runtime_learners
## INFO  [08:02:03.885] [bbotk]  19      0.1 optimal 0.1468974        0      0            0.145
## INFO  [08:02:03.885] [bbotk]                                 uhash
## INFO  [08:02:03.885] [bbotk]  971adfda-4a1e-4a4d-9355-87989ff866d2
## INFO  [08:02:03.887] [bbotk] Evaluating 1 configuration(s)
## INFO  [08:02:03.900] [mlr3] Running benchmark with 10 resampling iterations
## INFO  [08:02:03.907] [mlr3] Applying learner 'regr.kknn' on task 'PredictionSet[1:13]' (iter 1/10)
## INFO  [08:02:03.930] [mlr3] Applying learner 'regr.kknn' on task 'PredictionSet[1:13]' (iter 2/10)
## INFO  [08:02:03.954] [mlr3] Applying learner 'regr.kknn' on task 'PredictionSet[1:13]' (iter 3/10)
## INFO  [08:02:03.975] [mlr3] Applying learner 'regr.kknn' on task 'PredictionSet[1:13]' (iter 4/10)
## INFO  [08:02:03.996] [mlr3] Applying learner 'regr.kknn' on task 'PredictionSet[1:13]' (iter 5/10)
## INFO  [08:02:04.024] [mlr3] Applying learner 'regr.kknn' on task 'PredictionSet[1:13]' (iter 6/10)
## INFO  [08:02:04.045] [mlr3] Applying learner 'regr.kknn' on task 'PredictionSet[1:13]' (iter 7/10)
## INFO  [08:02:04.067] [mlr3] Applying learner 'regr.kknn' on task 'PredictionSet[1:13]' (iter 8/10)
## INFO  [08:02:04.090] [mlr3] Applying learner 'regr.kknn' on task 'PredictionSet[1:13]' (iter 9/10)
## INFO  [08:02:04.112] [mlr3] Applying learner 'regr.kknn' on task 'PredictionSet[1:13]' (iter 10/10)
## INFO  [08:02:04.133] [mlr3] Finished benchmark
## INFO  [08:02:04.166] [bbotk] Result of batch 6:
## INFO  [08:02:04.168] [bbotk]  k distance kernel regr.rmse warnings errors runtime_learners
## INFO  [08:02:04.168] [bbotk]  1 4.364286   rank 0.1326832        0      0            0.113
## INFO  [08:02:04.168] [bbotk]                                 uhash
## INFO  [08:02:04.168] [bbotk]  4cbf6586-6a03-4bc8-a934-76532f4ce684
## INFO  [08:02:04.169] [bbotk] Evaluating 1 configuration(s)
## INFO  [08:02:04.181] [mlr3] Running benchmark with 10 resampling iterations
## INFO  [08:02:04.187] [mlr3] Applying learner 'regr.kknn' on task 'PredictionSet[1:13]' (iter 1/10)
## INFO  [08:02:04.207] [mlr3] Applying learner 'regr.kknn' on task 'PredictionSet[1:13]' (iter 2/10)
## INFO  [08:02:04.230] [mlr3] Applying learner 'regr.kknn' on task 'PredictionSet[1:13]' (iter 3/10)
## INFO  [08:02:04.252] [mlr3] Applying learner 'regr.kknn' on task 'PredictionSet[1:13]' (iter 4/10)
## INFO  [08:02:04.273] [mlr3] Applying learner 'regr.kknn' on task 'PredictionSet[1:13]' (iter 5/10)
## INFO  [08:02:04.295] [mlr3] Applying learner 'regr.kknn' on task 'PredictionSet[1:13]' (iter 6/10)
## INFO  [08:02:04.316] [mlr3] Applying learner 'regr.kknn' on task 'PredictionSet[1:13]' (iter 7/10)
## INFO  [08:02:04.338] [mlr3] Applying learner 'regr.kknn' on task 'PredictionSet[1:13]' (iter 8/10)
## INFO  [08:02:04.359] [mlr3] Applying learner 'regr.kknn' on task 'PredictionSet[1:13]' (iter 9/10)
## INFO  [08:02:04.381] [mlr3] Applying learner 'regr.kknn' on task 'PredictionSet[1:13]' (iter 10/10)
## INFO  [08:02:04.403] [mlr3] Finished benchmark
## INFO  [08:02:04.440] [bbotk] Result of batch 7:
## INFO  [08:02:04.441] [bbotk]  k distance    kernel regr.rmse warnings errors runtime_learners
## INFO  [08:02:04.441] [bbotk]  8 7.207143 triweight 0.1789001        0      0            0.108
## INFO  [08:02:04.441] [bbotk]                                 uhash
## INFO  [08:02:04.441] [bbotk]  ba5dc176-d6d0-4cd4-9cf6-e944456737c3
## INFO  [08:02:04.443] [bbotk] Evaluating 1 configuration(s)
## INFO  [08:02:04.453] [mlr3] Running benchmark with 10 resampling iterations
## INFO  [08:02:04.459] [mlr3] Applying learner 'regr.kknn' on task 'PredictionSet[1:13]' (iter 1/10)
## INFO  [08:02:04.479] [mlr3] Applying learner 'regr.kknn' on task 'PredictionSet[1:13]' (iter 2/10)
## INFO  [08:02:04.499] [mlr3] Applying learner 'regr.kknn' on task 'PredictionSet[1:13]' (iter 3/10)
## INFO  [08:02:04.519] [mlr3] Applying learner 'regr.kknn' on task 'PredictionSet[1:13]' (iter 4/10)
## INFO  [08:02:04.541] [mlr3] Applying learner 'regr.kknn' on task 'PredictionSet[1:13]' (iter 5/10)
## INFO  [08:02:04.561] [mlr3] Applying learner 'regr.kknn' on task 'PredictionSet[1:13]' (iter 6/10)
## INFO  [08:02:04.581] [mlr3] Applying learner 'regr.kknn' on task 'PredictionSet[1:13]' (iter 7/10)
## INFO  [08:02:04.601] [mlr3] Applying learner 'regr.kknn' on task 'PredictionSet[1:13]' (iter 8/10)
## INFO  [08:02:04.625] [mlr3] Applying learner 'regr.kknn' on task 'PredictionSet[1:13]' (iter 9/10)
## INFO  [08:02:04.645] [mlr3] Applying learner 'regr.kknn' on task 'PredictionSet[1:13]' (iter 10/10)
## INFO  [08:02:04.665] [mlr3] Finished benchmark
## INFO  [08:02:04.701] [bbotk] Result of batch 8:
## INFO  [08:02:04.702] [bbotk]   k distance    kernel regr.rmse warnings errors runtime_learners
## INFO  [08:02:04.702] [bbotk]  15 4.364286 triweight 0.1694516        0      0            0.104
## INFO  [08:02:04.702] [bbotk]                                 uhash
## INFO  [08:02:04.702] [bbotk]  91b0b056-986e-4c80-bb25-078908cb98ef
## INFO  [08:02:04.704] [bbotk] Evaluating 1 configuration(s)
## INFO  [08:02:04.715] [mlr3] Running benchmark with 10 resampling iterations
## INFO  [08:02:04.721] [mlr3] Applying learner 'regr.kknn' on task 'PredictionSet[1:13]' (iter 1/10)
## INFO  [08:02:04.742] [mlr3] Applying learner 'regr.kknn' on task 'PredictionSet[1:13]' (iter 2/10)
## INFO  [08:02:04.764] [mlr3] Applying learner 'regr.kknn' on task 'PredictionSet[1:13]' (iter 3/10)
## INFO  [08:02:04.787] [mlr3] Applying learner 'regr.kknn' on task 'PredictionSet[1:13]' (iter 4/10)
## INFO  [08:02:04.807] [mlr3] Applying learner 'regr.kknn' on task 'PredictionSet[1:13]' (iter 5/10)
## INFO  [08:02:04.835] [mlr3] Applying learner 'regr.kknn' on task 'PredictionSet[1:13]' (iter 6/10)
## INFO  [08:02:04.856] [mlr3] Applying learner 'regr.kknn' on task 'PredictionSet[1:13]' (iter 7/10)
## INFO  [08:02:04.877] [mlr3] Applying learner 'regr.kknn' on task 'PredictionSet[1:13]' (iter 8/10)
## INFO  [08:02:04.897] [mlr3] Applying learner 'regr.kknn' on task 'PredictionSet[1:13]' (iter 9/10)
## INFO  [08:02:04.920] [mlr3] Applying learner 'regr.kknn' on task 'PredictionSet[1:13]' (iter 10/10)
## INFO  [08:02:04.941] [mlr3] Finished benchmark
## INFO  [08:02:04.980] [bbotk] Result of batch 9:
## INFO  [08:02:04.982] [bbotk]   k distance   kernel regr.rmse warnings errors runtime_learners
## INFO  [08:02:04.982] [bbotk]  13 12.89286 gaussian 0.1887184        0      0            0.112
## INFO  [08:02:04.982] [bbotk]                                 uhash
## INFO  [08:02:04.982] [bbotk]  69a5b963-c322-4e7e-ae13-885bc077d8a1
## INFO  [08:02:04.983] [bbotk] Evaluating 1 configuration(s)
## INFO  [08:02:04.996] [mlr3] Running benchmark with 10 resampling iterations
## INFO  [08:02:05.002] [mlr3] Applying learner 'regr.kknn' on task 'PredictionSet[1:13]' (iter 1/10)
## INFO  [08:02:05.027] [mlr3] Applying learner 'regr.kknn' on task 'PredictionSet[1:13]' (iter 2/10)
## INFO  [08:02:05.049] [mlr3] Applying learner 'regr.kknn' on task 'PredictionSet[1:13]' (iter 3/10)
## INFO  [08:02:05.071] [mlr3] Applying learner 'regr.kknn' on task 'PredictionSet[1:13]' (iter 4/10)
## INFO  [08:02:05.091] [mlr3] Applying learner 'regr.kknn' on task 'PredictionSet[1:13]' (iter 5/10)
## INFO  [08:02:05.111] [mlr3] Applying learner 'regr.kknn' on task 'PredictionSet[1:13]' (iter 6/10)
## INFO  [08:02:05.132] [mlr3] Applying learner 'regr.kknn' on task 'PredictionSet[1:13]' (iter 7/10)
## INFO  [08:02:05.154] [mlr3] Applying learner 'regr.kknn' on task 'PredictionSet[1:13]' (iter 8/10)
## INFO  [08:02:05.174] [mlr3] Applying learner 'regr.kknn' on task 'PredictionSet[1:13]' (iter 9/10)
## INFO  [08:02:05.194] [mlr3] Applying learner 'regr.kknn' on task 'PredictionSet[1:13]' (iter 10/10)
## INFO  [08:02:05.215] [mlr3] Finished benchmark
## INFO  [08:02:05.253] [bbotk] Result of batch 10:
## INFO  [08:02:05.255] [bbotk]  k distance      kernel regr.rmse warnings errors runtime_learners
## INFO  [08:02:05.255] [bbotk]  8 2.942857 rectangular 0.1895676        0      0             0.11
## INFO  [08:02:05.255] [bbotk]                                 uhash
## INFO  [08:02:05.255] [bbotk]  815a278f-e3b0-44bb-a208-10dfbea10d84
## INFO  [08:02:05.259] [bbotk] Finished optimizing after 10 evaluation(s)
## INFO  [08:02:05.260] [bbotk] Result:
## INFO  [08:02:05.261] [bbotk]  k distance kernel learner_param_vals  x_domain regr.rmse
## INFO  [08:02:05.261] [bbotk]  1 4.364286   rank          <list[3]> <list[3]> 0.1326832
## INFO  [08:02:05.469] [bbotk] Starting to optimize 2 parameter(s) with '<TunerGridSearch>' and '<TerminatorEvals> [n_evals=10, k=0]'
## INFO  [08:02:05.472] [bbotk] Evaluating 1 configuration(s)
## INFO  [08:02:05.493] [mlr3] Running benchmark with 10 resampling iterations
## INFO  [08:02:05.504] [mlr3] Applying learner 'regr.ranger' on task 'PredictionSet[1:13]' (iter 1/10)
## INFO  [08:02:05.540] [mlr3] Applying learner 'regr.ranger' on task 'PredictionSet[1:13]' (iter 2/10)
## INFO  [08:02:05.560] [mlr3] Applying learner 'regr.ranger' on task 'PredictionSet[1:13]' (iter 3/10)
## INFO  [08:02:05.581] [mlr3] Applying learner 'regr.ranger' on task 'PredictionSet[1:13]' (iter 4/10)
## INFO  [08:02:05.602] [mlr3] Applying learner 'regr.ranger' on task 'PredictionSet[1:13]' (iter 5/10)
## INFO  [08:02:05.622] [mlr3] Applying learner 'regr.ranger' on task 'PredictionSet[1:13]' (iter 6/10)
## INFO  [08:02:05.642] [mlr3] Applying learner 'regr.ranger' on task 'PredictionSet[1:13]' (iter 7/10)
## INFO  [08:02:05.665] [mlr3] Applying learner 'regr.ranger' on task 'PredictionSet[1:13]' (iter 8/10)
## INFO  [08:02:05.685] [mlr3] Applying learner 'regr.ranger' on task 'PredictionSet[1:13]' (iter 9/10)
## INFO  [08:02:05.705] [mlr3] Applying learner 'regr.ranger' on task 'PredictionSet[1:13]' (iter 10/10)
## INFO  [08:02:05.725] [mlr3] Finished benchmark
## INFO  [08:02:05.756] [bbotk] Result of batch 1:
## INFO  [08:02:05.758] [bbotk]  max.depth num.trees regr.rmse warnings errors runtime_learners
## INFO  [08:02:05.758] [bbotk]         13        79  0.129427        0      0            0.114
## INFO  [08:02:05.758] [bbotk]                                 uhash
## INFO  [08:02:05.758] [bbotk]  43b37e49-8b6d-43cb-8414-52c1ac073f5f
## INFO  [08:02:05.759] [bbotk] Evaluating 1 configuration(s)
## INFO  [08:02:05.780] [mlr3] Running benchmark with 10 resampling iterations
## INFO  [08:02:05.785] [mlr3] Applying learner 'regr.ranger' on task 'PredictionSet[1:13]' (iter 1/10)
## INFO  [08:02:05.816] [mlr3] Applying learner 'regr.ranger' on task 'PredictionSet[1:13]' (iter 2/10)
## INFO  [08:02:05.843] [mlr3] Applying learner 'regr.ranger' on task 'PredictionSet[1:13]' (iter 3/10)
## INFO  [08:02:05.871] [mlr3] Applying learner 'regr.ranger' on task 'PredictionSet[1:13]' (iter 4/10)
## INFO  [08:02:05.900] [mlr3] Applying learner 'regr.ranger' on task 'PredictionSet[1:13]' (iter 5/10)
## INFO  [08:02:05.927] [mlr3] Applying learner 'regr.ranger' on task 'PredictionSet[1:13]' (iter 6/10)
## INFO  [08:02:05.955] [mlr3] Applying learner 'regr.ranger' on task 'PredictionSet[1:13]' (iter 7/10)
## INFO  [08:02:05.984] [mlr3] Applying learner 'regr.ranger' on task 'PredictionSet[1:13]' (iter 8/10)
## INFO  [08:02:06.019] [mlr3] Applying learner 'regr.ranger' on task 'PredictionSet[1:13]' (iter 9/10)
## INFO  [08:02:06.049] [mlr3] Applying learner 'regr.ranger' on task 'PredictionSet[1:13]' (iter 10/10)
## INFO  [08:02:06.076] [mlr3] Finished benchmark
## INFO  [08:02:06.107] [bbotk] Result of batch 2:
## INFO  [08:02:06.109] [bbotk]  max.depth num.trees regr.rmse warnings errors runtime_learners
## INFO  [08:02:06.109] [bbotk]         18       185 0.1280601        0      0            0.182
## INFO  [08:02:06.109] [bbotk]                                 uhash
## INFO  [08:02:06.109] [bbotk]  38c1c952-fd2f-4195-90de-c7d3c06d3bf9
## INFO  [08:02:06.110] [bbotk] Evaluating 1 configuration(s)
## INFO  [08:02:06.132] [mlr3] Running benchmark with 10 resampling iterations
## INFO  [08:02:06.137] [mlr3] Applying learner 'regr.ranger' on task 'PredictionSet[1:13]' (iter 1/10)
## INFO  [08:02:06.159] [mlr3] Applying learner 'regr.ranger' on task 'PredictionSet[1:13]' (iter 2/10)
## INFO  [08:02:06.182] [mlr3] Applying learner 'regr.ranger' on task 'PredictionSet[1:13]' (iter 3/10)
## INFO  [08:02:06.206] [mlr3] Applying learner 'regr.ranger' on task 'PredictionSet[1:13]' (iter 4/10)
## INFO  [08:02:06.228] [mlr3] Applying learner 'regr.ranger' on task 'PredictionSet[1:13]' (iter 5/10)
## INFO  [08:02:06.250] [mlr3] Applying learner 'regr.ranger' on task 'PredictionSet[1:13]' (iter 6/10)
## INFO  [08:02:06.274] [mlr3] Applying learner 'regr.ranger' on task 'PredictionSet[1:13]' (iter 7/10)
## INFO  [08:02:06.296] [mlr3] Applying learner 'regr.ranger' on task 'PredictionSet[1:13]' (iter 8/10)
## INFO  [08:02:06.318] [mlr3] Applying learner 'regr.ranger' on task 'PredictionSet[1:13]' (iter 9/10)
## INFO  [08:02:06.342] [mlr3] Applying learner 'regr.ranger' on task 'PredictionSet[1:13]' (iter 10/10)
## INFO  [08:02:06.364] [mlr3] Finished benchmark
## INFO  [08:02:06.396] [bbotk] Result of batch 3:
## INFO  [08:02:06.397] [bbotk]  max.depth num.trees regr.rmse warnings errors runtime_learners
## INFO  [08:02:06.397] [bbotk]          1       343 0.2591671        0      0            0.119
## INFO  [08:02:06.397] [bbotk]                                 uhash
## INFO  [08:02:06.397] [bbotk]  476b6082-ee9e-446c-9cf7-e98cd6686313
## INFO  [08:02:06.399] [bbotk] Evaluating 1 configuration(s)
## INFO  [08:02:06.428] [mlr3] Running benchmark with 10 resampling iterations
## INFO  [08:02:06.433] [mlr3] Applying learner 'regr.ranger' on task 'PredictionSet[1:13]' (iter 1/10)
## INFO  [08:02:06.460] [mlr3] Applying learner 'regr.ranger' on task 'PredictionSet[1:13]' (iter 2/10)
## INFO  [08:02:06.490] [mlr3] Applying learner 'regr.ranger' on task 'PredictionSet[1:13]' (iter 3/10)
## INFO  [08:02:06.518] [mlr3] Applying learner 'regr.ranger' on task 'PredictionSet[1:13]' (iter 4/10)
## INFO  [08:02:06.545] [mlr3] Applying learner 'regr.ranger' on task 'PredictionSet[1:13]' (iter 5/10)
## INFO  [08:02:06.575] [mlr3] Applying learner 'regr.ranger' on task 'PredictionSet[1:13]' (iter 6/10)
## INFO  [08:02:06.602] [mlr3] Applying learner 'regr.ranger' on task 'PredictionSet[1:13]' (iter 7/10)
## INFO  [08:02:06.631] [mlr3] Applying learner 'regr.ranger' on task 'PredictionSet[1:13]' (iter 8/10)
## INFO  [08:02:06.660] [mlr3] Applying learner 'regr.ranger' on task 'PredictionSet[1:13]' (iter 9/10)
## INFO  [08:02:06.687] [mlr3] Applying learner 'regr.ranger' on task 'PredictionSet[1:13]' (iter 10/10)
## INFO  [08:02:06.716] [mlr3] Finished benchmark
## INFO  [08:02:06.747] [bbotk] Result of batch 4:
## INFO  [08:02:06.748] [bbotk]  max.depth num.trees regr.rmse warnings errors runtime_learners
## INFO  [08:02:06.748] [bbotk]          0       185 0.1242315        0      0            0.179
## INFO  [08:02:06.748] [bbotk]                                 uhash
## INFO  [08:02:06.748] [bbotk]  d10afa90-2871-43b4-867f-056638963733
## INFO  [08:02:06.750] [bbotk] Evaluating 1 configuration(s)
## INFO  [08:02:06.771] [mlr3] Running benchmark with 10 resampling iterations
## INFO  [08:02:06.776] [mlr3] Applying learner 'regr.ranger' on task 'PredictionSet[1:13]' (iter 1/10)
## INFO  [08:02:06.823] [mlr3] Applying learner 'regr.ranger' on task 'PredictionSet[1:13]' (iter 2/10)
## INFO  [08:02:06.864] [mlr3] Applying learner 'regr.ranger' on task 'PredictionSet[1:13]' (iter 3/10)
## INFO  [08:02:06.903] [mlr3] Applying learner 'regr.ranger' on task 'PredictionSet[1:13]' (iter 4/10)
## INFO  [08:02:06.943] [mlr3] Applying learner 'regr.ranger' on task 'PredictionSet[1:13]' (iter 5/10)
## INFO  [08:02:06.982] [mlr3] Applying learner 'regr.ranger' on task 'PredictionSet[1:13]' (iter 6/10)
## INFO  [08:02:07.022] [mlr3] Applying learner 'regr.ranger' on task 'PredictionSet[1:13]' (iter 7/10)
## INFO  [08:02:07.061] [mlr3] Applying learner 'regr.ranger' on task 'PredictionSet[1:13]' (iter 8/10)
## INFO  [08:02:07.103] [mlr3] Applying learner 'regr.ranger' on task 'PredictionSet[1:13]' (iter 9/10)
## INFO  [08:02:07.142] [mlr3] Applying learner 'regr.ranger' on task 'PredictionSet[1:13]' (iter 10/10)
## INFO  [08:02:07.183] [mlr3] Finished benchmark
## INFO  [08:02:07.215] [bbotk] Result of batch 5:
## INFO  [08:02:07.217] [bbotk]  max.depth num.trees regr.rmse warnings errors runtime_learners
## INFO  [08:02:07.217] [bbotk]         17       343 0.1287563        0      0            0.303
## INFO  [08:02:07.217] [bbotk]                                 uhash
## INFO  [08:02:07.217] [bbotk]  6be532f6-f1fd-41f5-bc65-a07ea387a292
## INFO  [08:02:07.218] [bbotk] Evaluating 1 configuration(s)
## INFO  [08:02:07.241] [mlr3] Running benchmark with 10 resampling iterations
## INFO  [08:02:07.246] [mlr3] Applying learner 'regr.ranger' on task 'PredictionSet[1:13]' (iter 1/10)
## INFO  [08:02:07.282] [mlr3] Applying learner 'regr.ranger' on task 'PredictionSet[1:13]' (iter 2/10)
## INFO  [08:02:07.314] [mlr3] Applying learner 'regr.ranger' on task 'PredictionSet[1:13]' (iter 3/10)
## INFO  [08:02:07.344] [mlr3] Applying learner 'regr.ranger' on task 'PredictionSet[1:13]' (iter 4/10)
## INFO  [08:02:07.374] [mlr3] Applying learner 'regr.ranger' on task 'PredictionSet[1:13]' (iter 5/10)
## INFO  [08:02:07.405] [mlr3] Applying learner 'regr.ranger' on task 'PredictionSet[1:13]' (iter 6/10)
## INFO  [08:02:07.436] [mlr3] Applying learner 'regr.ranger' on task 'PredictionSet[1:13]' (iter 7/10)
## INFO  [08:02:07.467] [mlr3] Applying learner 'regr.ranger' on task 'PredictionSet[1:13]' (iter 8/10)
## INFO  [08:02:07.499] [mlr3] Applying learner 'regr.ranger' on task 'PredictionSet[1:13]' (iter 9/10)
## INFO  [08:02:07.528] [mlr3] Applying learner 'regr.ranger' on task 'PredictionSet[1:13]' (iter 10/10)
## INFO  [08:02:07.558] [mlr3] Finished benchmark
## INFO  [08:02:07.590] [bbotk] Result of batch 6:
## INFO  [08:02:07.591] [bbotk]  max.depth num.trees regr.rmse warnings errors runtime_learners
## INFO  [08:02:07.591] [bbotk]          2       500 0.2194331        0      0            0.205
## INFO  [08:02:07.591] [bbotk]                                 uhash
## INFO  [08:02:07.591] [bbotk]  754c146b-ef98-4db4-a084-74bb9ae30ee7
## INFO  [08:02:07.593] [bbotk] Evaluating 1 configuration(s)
## INFO  [08:02:07.615] [mlr3] Running benchmark with 10 resampling iterations
## INFO  [08:02:07.621] [mlr3] Applying learner 'regr.ranger' on task 'PredictionSet[1:13]' (iter 1/10)
## INFO  [08:02:07.662] [mlr3] Applying learner 'regr.ranger' on task 'PredictionSet[1:13]' (iter 2/10)
## INFO  [08:02:07.704] [mlr3] Applying learner 'regr.ranger' on task 'PredictionSet[1:13]' (iter 3/10)
## INFO  [08:02:07.745] [mlr3] Applying learner 'regr.ranger' on task 'PredictionSet[1:13]' (iter 4/10)
## INFO  [08:02:07.794] [mlr3] Applying learner 'regr.ranger' on task 'PredictionSet[1:13]' (iter 5/10)
## INFO  [08:02:07.838] [mlr3] Applying learner 'regr.ranger' on task 'PredictionSet[1:13]' (iter 6/10)
## INFO  [08:02:07.880] [mlr3] Applying learner 'regr.ranger' on task 'PredictionSet[1:13]' (iter 7/10)
## INFO  [08:02:07.923] [mlr3] Applying learner 'regr.ranger' on task 'PredictionSet[1:13]' (iter 8/10)
## INFO  [08:02:07.965] [mlr3] Applying learner 'regr.ranger' on task 'PredictionSet[1:13]' (iter 9/10)
## INFO  [08:02:08.007] [mlr3] Applying learner 'regr.ranger' on task 'PredictionSet[1:13]' (iter 10/10)
## INFO  [08:02:08.048] [mlr3] Finished benchmark
## INFO  [08:02:08.082] [bbotk] Result of batch 7:
## INFO  [08:02:08.083] [bbotk]  max.depth num.trees regr.rmse warnings errors runtime_learners
## INFO  [08:02:08.083] [bbotk]          7       369 0.1298585        0      0            0.317
## INFO  [08:02:08.083] [bbotk]                                 uhash
## INFO  [08:02:08.083] [bbotk]  88c39ca9-7a6d-4b94-bfdb-286f472dce06
## INFO  [08:02:08.085] [bbotk] Evaluating 1 configuration(s)
## INFO  [08:02:08.107] [mlr3] Running benchmark with 10 resampling iterations
## INFO  [08:02:08.112] [mlr3] Applying learner 'regr.ranger' on task 'PredictionSet[1:13]' (iter 1/10)
## INFO  [08:02:08.143] [mlr3] Applying learner 'regr.ranger' on task 'PredictionSet[1:13]' (iter 2/10)
## INFO  [08:02:08.219] [mlr3] Applying learner 'regr.ranger' on task 'PredictionSet[1:13]' (iter 3/10)
## INFO  [08:02:08.251] [mlr3] Applying learner 'regr.ranger' on task 'PredictionSet[1:13]' (iter 4/10)
## INFO  [08:02:08.280] [mlr3] Applying learner 'regr.ranger' on task 'PredictionSet[1:13]' (iter 5/10)
## INFO  [08:02:08.309] [mlr3] Applying learner 'regr.ranger' on task 'PredictionSet[1:13]' (iter 6/10)
## INFO  [08:02:08.337] [mlr3] Applying learner 'regr.ranger' on task 'PredictionSet[1:13]' (iter 7/10)
## INFO  [08:02:08.366] [mlr3] Applying learner 'regr.ranger' on task 'PredictionSet[1:13]' (iter 8/10)
## INFO  [08:02:08.394] [mlr3] Applying learner 'regr.ranger' on task 'PredictionSet[1:13]' (iter 9/10)
## INFO  [08:02:08.422] [mlr3] Applying learner 'regr.ranger' on task 'PredictionSet[1:13]' (iter 10/10)
## INFO  [08:02:08.450] [mlr3] Finished benchmark
## INFO  [08:02:08.481] [bbotk] Result of batch 8:
## INFO  [08:02:08.483] [bbotk]  max.depth num.trees regr.rmse warnings errors runtime_learners
## INFO  [08:02:08.483] [bbotk]         11       185 0.1287338        0      0            0.224
## INFO  [08:02:08.483] [bbotk]                                 uhash
## INFO  [08:02:08.483] [bbotk]  e62021fa-b0a6-4194-aea1-d4d1fde66af5
## INFO  [08:02:08.484] [bbotk] Evaluating 1 configuration(s)
## INFO  [08:02:08.506] [mlr3] Running benchmark with 10 resampling iterations
## INFO  [08:02:08.512] [mlr3] Applying learner 'regr.ranger' on task 'PredictionSet[1:13]' (iter 1/10)
## INFO  [08:02:08.544] [mlr3] Applying learner 'regr.ranger' on task 'PredictionSet[1:13]' (iter 2/10)
## INFO  [08:02:08.577] [mlr3] Applying learner 'regr.ranger' on task 'PredictionSet[1:13]' (iter 3/10)
## INFO  [08:02:08.610] [mlr3] Applying learner 'regr.ranger' on task 'PredictionSet[1:13]' (iter 4/10)
## INFO  [08:02:08.643] [mlr3] Applying learner 'regr.ranger' on task 'PredictionSet[1:13]' (iter 5/10)
## INFO  [08:02:08.685] [mlr3] Applying learner 'regr.ranger' on task 'PredictionSet[1:13]' (iter 6/10)
## INFO  [08:02:08.718] [mlr3] Applying learner 'regr.ranger' on task 'PredictionSet[1:13]' (iter 7/10)
## INFO  [08:02:08.752] [mlr3] Applying learner 'regr.ranger' on task 'PredictionSet[1:13]' (iter 8/10)
## INFO  [08:02:08.784] [mlr3] Applying learner 'regr.ranger' on task 'PredictionSet[1:13]' (iter 9/10)
## INFO  [08:02:08.819] [mlr3] Applying learner 'regr.ranger' on task 'PredictionSet[1:13]' (iter 10/10)
## INFO  [08:02:08.851] [mlr3] Finished benchmark
## INFO  [08:02:08.883] [bbotk] Result of batch 9:
## INFO  [08:02:08.885] [bbotk]  max.depth num.trees regr.rmse warnings errors runtime_learners
## INFO  [08:02:08.885] [bbotk]          4       395 0.1606327        0      0            0.229
## INFO  [08:02:08.885] [bbotk]                                 uhash
## INFO  [08:02:08.885] [bbotk]  90a3a728-0a53-4b7c-a3b0-f26f03875eb2
## INFO  [08:02:08.886] [bbotk] Evaluating 1 configuration(s)
## INFO  [08:02:08.908] [mlr3] Running benchmark with 10 resampling iterations
## INFO  [08:02:08.913] [mlr3] Applying learner 'regr.ranger' on task 'PredictionSet[1:13]' (iter 1/10)
## INFO  [08:02:08.932] [mlr3] Applying learner 'regr.ranger' on task 'PredictionSet[1:13]' (iter 2/10)
## INFO  [08:02:08.952] [mlr3] Applying learner 'regr.ranger' on task 'PredictionSet[1:13]' (iter 3/10)
## INFO  [08:02:08.973] [mlr3] Applying learner 'regr.ranger' on task 'PredictionSet[1:13]' (iter 4/10)
## INFO  [08:02:08.993] [mlr3] Applying learner 'regr.ranger' on task 'PredictionSet[1:13]' (iter 5/10)
## INFO  [08:02:09.012] [mlr3] Applying learner 'regr.ranger' on task 'PredictionSet[1:13]' (iter 6/10)
## INFO  [08:02:09.033] [mlr3] Applying learner 'regr.ranger' on task 'PredictionSet[1:13]' (iter 7/10)
## INFO  [08:02:09.054] [mlr3] Applying learner 'regr.ranger' on task 'PredictionSet[1:13]' (iter 8/10)
## INFO  [08:02:09.073] [mlr3] Applying learner 'regr.ranger' on task 'PredictionSet[1:13]' (iter 9/10)
## INFO  [08:02:09.093] [mlr3] Applying learner 'regr.ranger' on task 'PredictionSet[1:13]' (iter 10/10)
## INFO  [08:02:09.115] [mlr3] Finished benchmark
## INFO  [08:02:09.155] [bbotk] Result of batch 10:
## INFO  [08:02:09.156] [bbotk]  max.depth num.trees regr.rmse warnings errors runtime_learners
## INFO  [08:02:09.156] [bbotk]          6        79 0.1354343        0      0            0.099
## INFO  [08:02:09.156] [bbotk]                                 uhash
## INFO  [08:02:09.156] [bbotk]  fdc6ab6d-336a-4f86-8784-5a7f28f294bc
## INFO  [08:02:09.161] [bbotk] Finished optimizing after 10 evaluation(s)
## INFO  [08:02:09.162] [bbotk] Result:
## INFO  [08:02:09.163] [bbotk]  max.depth num.trees learner_param_vals  x_domain regr.rmse
## INFO  [08:02:09.163] [bbotk]          0       185          <list[3]> <list[2]> 0.1242315
## INFO  [08:02:09.392] [bbotk] Starting to optimize 5 parameter(s) with '<TunerGridSearch>' and '<TerminatorEvals> [n_evals=10, k=0]'
## INFO  [08:02:09.399] [bbotk] Evaluating 1 configuration(s)
## INFO  [08:02:09.417] [mlr3] Running benchmark with 10 resampling iterations
## INFO  [08:02:09.429] [mlr3] Applying learner 'regr.svm' on task 'PredictionSet[1:13]' (iter 1/10)
## INFO  [08:02:09.450] [mlr3] Applying learner 'regr.svm' on task 'PredictionSet[1:13]' (iter 2/10)
## INFO  [08:02:09.468] [mlr3] Applying learner 'regr.svm' on task 'PredictionSet[1:13]' (iter 3/10)
## INFO  [08:02:09.490] [mlr3] Applying learner 'regr.svm' on task 'PredictionSet[1:13]' (iter 4/10)
## INFO  [08:02:09.510] [mlr3] Applying learner 'regr.svm' on task 'PredictionSet[1:13]' (iter 5/10)
## INFO  [08:02:09.529] [mlr3] Applying learner 'regr.svm' on task 'PredictionSet[1:13]' (iter 6/10)
## INFO  [08:02:09.549] [mlr3] Applying learner 'regr.svm' on task 'PredictionSet[1:13]' (iter 7/10)
## INFO  [08:02:09.569] [mlr3] Applying learner 'regr.svm' on task 'PredictionSet[1:13]' (iter 8/10)
## INFO  [08:02:09.588] [mlr3] Applying learner 'regr.svm' on task 'PredictionSet[1:13]' (iter 9/10)
## INFO  [08:02:09.608] [mlr3] Applying learner 'regr.svm' on task 'PredictionSet[1:13]' (iter 10/10)
## INFO  [08:02:09.627] [mlr3] Finished benchmark
## INFO  [08:02:09.657] [bbotk] Result of batch 1:
## INFO  [08:02:09.658] [bbotk]  tolerance    gamma   epsilon           type kernel regr.rmse warnings errors
## INFO  [08:02:09.658] [bbotk]      0.001 32.14321 0.1571429 eps-regression radial  4.311199        0      0
## INFO  [08:02:09.658] [bbotk]  runtime_learners                                uhash
## INFO  [08:02:09.658] [bbotk]             0.099 41b73336-2dfe-498b-a73c-c961563682eb
## INFO  [08:02:09.660] [bbotk] Evaluating 1 configuration(s)
## INFO  [08:02:09.680] [mlr3] Running benchmark with 10 resampling iterations
## INFO  [08:02:09.686] [mlr3] Applying learner 'regr.svm' on task 'PredictionSet[1:13]' (iter 1/10)
## INFO  [08:02:09.704] [mlr3] Applying learner 'regr.svm' on task 'PredictionSet[1:13]' (iter 2/10)
## INFO  [08:02:09.721] [mlr3] Applying learner 'regr.svm' on task 'PredictionSet[1:13]' (iter 3/10)
## INFO  [08:02:09.736] [mlr3] Applying learner 'regr.svm' on task 'PredictionSet[1:13]' (iter 4/10)
## INFO  [08:02:09.751] [mlr3] Applying learner 'regr.svm' on task 'PredictionSet[1:13]' (iter 5/10)
## INFO  [08:02:09.766] [mlr3] Applying learner 'regr.svm' on task 'PredictionSet[1:13]' (iter 6/10)
## INFO  [08:02:09.782] [mlr3] Applying learner 'regr.svm' on task 'PredictionSet[1:13]' (iter 7/10)
## INFO  [08:02:09.799] [mlr3] Applying learner 'regr.svm' on task 'PredictionSet[1:13]' (iter 8/10)
## INFO  [08:02:09.814] [mlr3] Applying learner 'regr.svm' on task 'PredictionSet[1:13]' (iter 9/10)
## INFO  [08:02:09.829] [mlr3] Applying learner 'regr.svm' on task 'PredictionSet[1:13]' (iter 10/10)
## INFO  [08:02:09.844] [mlr3] Finished benchmark
## INFO  [08:02:09.877] [bbotk] Result of batch 2:
## INFO  [08:02:09.879] [bbotk]  tolerance    gamma   epsilon           type kernel regr.rmse warnings errors
## INFO  [08:02:09.879] [bbotk]  0.7073571 14.28643 0.6714286 eps-regression radial  5.679432        0      0
## INFO  [08:02:09.879] [bbotk]  runtime_learners                                uhash
## INFO  [08:02:09.879] [bbotk]             0.051 012ba51c-a65a-4fa8-9833-7d143d70fce3
## INFO  [08:02:09.880] [bbotk] Evaluating 1 configuration(s)
## INFO  [08:02:09.895] [mlr3] Running benchmark with 10 resampling iterations
## INFO  [08:02:09.900] [mlr3] Applying learner 'regr.svm' on task 'PredictionSet[1:13]' (iter 1/10)
## INFO  [08:02:09.927] [mlr3] Applying learner 'regr.svm' on task 'PredictionSet[1:13]' (iter 2/10)
## INFO  [08:02:09.947] [mlr3] Applying learner 'regr.svm' on task 'PredictionSet[1:13]' (iter 3/10)
## INFO  [08:02:09.965] [mlr3] Applying learner 'regr.svm' on task 'PredictionSet[1:13]' (iter 4/10)
## INFO  [08:02:09.983] [mlr3] Applying learner 'regr.svm' on task 'PredictionSet[1:13]' (iter 5/10)
## INFO  [08:02:10.002] [mlr3] Applying learner 'regr.svm' on task 'PredictionSet[1:13]' (iter 6/10)
## INFO  [08:02:10.022] [mlr3] Applying learner 'regr.svm' on task 'PredictionSet[1:13]' (iter 7/10)
## INFO  [08:02:10.041] [mlr3] Applying learner 'regr.svm' on task 'PredictionSet[1:13]' (iter 8/10)
## INFO  [08:02:10.059] [mlr3] Applying learner 'regr.svm' on task 'PredictionSet[1:13]' (iter 9/10)
## INFO  [08:02:10.078] [mlr3] Applying learner 'regr.svm' on task 'PredictionSet[1:13]' (iter 10/10)
## INFO  [08:02:10.097] [mlr3] Finished benchmark
## INFO  [08:02:10.127] [bbotk] Result of batch 3:
## INFO  [08:02:10.129] [bbotk]  tolerance    gamma epsilon           type  kernel regr.rmse warnings errors
## INFO  [08:02:10.129] [bbotk]  0.7073571 21.42914     0.1 eps-regression sigmoid  233.5323        0      0
## INFO  [08:02:10.129] [bbotk]  runtime_learners                                uhash
## INFO  [08:02:10.129] [bbotk]             0.091 1e9cd196-17dc-45d2-b278-a9bac9d1142a
## INFO  [08:02:10.130] [bbotk] Evaluating 1 configuration(s)
## INFO  [08:02:10.349] [mlr3] Running benchmark with 10 resampling iterations
## INFO  [08:02:10.356] [mlr3] Applying learner 'regr.svm' on task 'PredictionSet[1:13]' (iter 1/10)
## INFO  [08:02:10.373] [mlr3] Applying learner 'regr.svm' on task 'PredictionSet[1:13]' (iter 2/10)
## INFO  [08:02:10.391] [mlr3] Applying learner 'regr.svm' on task 'PredictionSet[1:13]' (iter 3/10)
## INFO  [08:02:10.407] [mlr3] Applying learner 'regr.svm' on task 'PredictionSet[1:13]' (iter 4/10)
## INFO  [08:02:10.423] [mlr3] Applying learner 'regr.svm' on task 'PredictionSet[1:13]' (iter 5/10)
## INFO  [08:02:10.440] [mlr3] Applying learner 'regr.svm' on task 'PredictionSet[1:13]' (iter 6/10)
## INFO  [08:02:10.456] [mlr3] Applying learner 'regr.svm' on task 'PredictionSet[1:13]' (iter 7/10)
## INFO  [08:02:10.473] [mlr3] Applying learner 'regr.svm' on task 'PredictionSet[1:13]' (iter 8/10)
## INFO  [08:02:10.489] [mlr3] Applying learner 'regr.svm' on task 'PredictionSet[1:13]' (iter 9/10)
## INFO  [08:02:10.504] [mlr3] Applying learner 'regr.svm' on task 'PredictionSet[1:13]' (iter 10/10)
## INFO  [08:02:10.519] [mlr3] Finished benchmark
## INFO  [08:02:10.551] [bbotk] Result of batch 4:
## INFO  [08:02:10.553] [bbotk]  tolerance   gamma epsilon           type kernel regr.rmse warnings errors
## INFO  [08:02:10.553] [bbotk]  0.5147143 25.0005     0.5 eps-regression radial  5.065844        0      0
## INFO  [08:02:10.553] [bbotk]  runtime_learners                                uhash
## INFO  [08:02:10.553] [bbotk]             0.059 388741e3-5664-4b9a-a351-995fc6d0628d
## INFO  [08:02:10.554] [bbotk] Evaluating 1 configuration(s)
## INFO  [08:02:10.569] [mlr3] Running benchmark with 10 resampling iterations
## INFO  [08:02:10.574] [mlr3] Applying learner 'regr.svm' on task 'PredictionSet[1:13]' (iter 1/10)
## INFO  [08:02:10.589] [mlr3] Applying learner 'regr.svm' on task 'PredictionSet[1:13]' (iter 2/10)
## INFO  [08:02:10.605] [mlr3] Applying learner 'regr.svm' on task 'PredictionSet[1:13]' (iter 3/10)
## INFO  [08:02:10.622] [mlr3] Applying learner 'regr.svm' on task 'PredictionSet[1:13]' (iter 4/10)
## INFO  [08:02:10.637] [mlr3] Applying learner 'regr.svm' on task 'PredictionSet[1:13]' (iter 5/10)
## INFO  [08:02:10.652] [mlr3] Applying learner 'regr.svm' on task 'PredictionSet[1:13]' (iter 6/10)
## INFO  [08:02:10.668] [mlr3] Applying learner 'regr.svm' on task 'PredictionSet[1:13]' (iter 7/10)
## INFO  [08:02:10.684] [mlr3] Applying learner 'regr.svm' on task 'PredictionSet[1:13]' (iter 8/10)
## INFO  [08:02:10.701] [mlr3] Applying learner 'regr.svm' on task 'PredictionSet[1:13]' (iter 9/10)
## INFO  [08:02:10.723] [mlr3] Applying learner 'regr.svm' on task 'PredictionSet[1:13]' (iter 10/10)
## INFO  [08:02:10.738] [mlr3] Finished benchmark
## INFO  [08:02:10.770] [bbotk] Result of batch 5:
## INFO  [08:02:10.771] [bbotk]  tolerance   gamma   epsilon           type kernel regr.rmse warnings errors
## INFO  [08:02:10.771] [bbotk]  0.2578571 25.0005 0.7285714 eps-regression radial  5.495351        0      0
## INFO  [08:02:10.771] [bbotk]  runtime_learners                                uhash
## INFO  [08:02:10.771] [bbotk]             0.066 d78bfa7c-f73b-4dbc-bf10-2de79eb6d3d0
## INFO  [08:02:10.773] [bbotk] Evaluating 1 configuration(s)
## INFO  [08:02:10.788] [mlr3] Running benchmark with 10 resampling iterations
## INFO  [08:02:10.792] [mlr3] Applying learner 'regr.svm' on task 'PredictionSet[1:13]' (iter 1/10)
## INFO  [08:02:10.807] [mlr3] Applying learner 'regr.svm' on task 'PredictionSet[1:13]' (iter 2/10)
## INFO  [08:02:10.822] [mlr3] Applying learner 'regr.svm' on task 'PredictionSet[1:13]' (iter 3/10)
## INFO  [08:02:10.839] [mlr3] Applying learner 'regr.svm' on task 'PredictionSet[1:13]' (iter 4/10)
## INFO  [08:02:10.854] [mlr3] Applying learner 'regr.svm' on task 'PredictionSet[1:13]' (iter 5/10)
## INFO  [08:02:10.868] [mlr3] Applying learner 'regr.svm' on task 'PredictionSet[1:13]' (iter 6/10)
## INFO  [08:02:10.883] [mlr3] Applying learner 'regr.svm' on task 'PredictionSet[1:13]' (iter 7/10)
## INFO  [08:02:10.897] [mlr3] Applying learner 'regr.svm' on task 'PredictionSet[1:13]' (iter 8/10)
## INFO  [08:02:10.914] [mlr3] Applying learner 'regr.svm' on task 'PredictionSet[1:13]' (iter 9/10)
## INFO  [08:02:10.929] [mlr3] Applying learner 'regr.svm' on task 'PredictionSet[1:13]' (iter 10/10)
## INFO  [08:02:10.943] [mlr3] Finished benchmark
## INFO  [08:02:10.973] [bbotk] Result of batch 6:
## INFO  [08:02:10.975] [bbotk]  tolerance gamma   epsilon           type kernel regr.rmse warnings errors
## INFO  [08:02:10.975] [bbotk]  0.8357857    50 0.8428571 eps-regression radial  6.729496        0      0
## INFO  [08:02:10.975] [bbotk]  runtime_learners                                uhash
## INFO  [08:02:10.975] [bbotk]             0.051 0ae2393c-4994-4007-ba5e-666fad6dad14
## INFO  [08:02:10.976] [bbotk] Evaluating 1 configuration(s)
## INFO  [08:02:10.992] [mlr3] Running benchmark with 10 resampling iterations
## INFO  [08:02:10.997] [mlr3] Applying learner 'regr.svm' on task 'PredictionSet[1:13]' (iter 1/10)
## INFO  [08:02:11.013] [mlr3] Applying learner 'regr.svm' on task 'PredictionSet[1:13]' (iter 2/10)
## INFO  [08:02:11.029] [mlr3] Applying learner 'regr.svm' on task 'PredictionSet[1:13]' (iter 3/10)
## INFO  [08:02:11.051] [mlr3] Applying learner 'regr.svm' on task 'PredictionSet[1:13]' (iter 4/10)
## INFO  [08:02:11.069] [mlr3] Applying learner 'regr.svm' on task 'PredictionSet[1:13]' (iter 5/10)
## INFO  [08:02:11.085] [mlr3] Applying learner 'regr.svm' on task 'PredictionSet[1:13]' (iter 6/10)
## INFO  [08:02:11.101] [mlr3] Applying learner 'regr.svm' on task 'PredictionSet[1:13]' (iter 7/10)
## INFO  [08:02:11.116] [mlr3] Applying learner 'regr.svm' on task 'PredictionSet[1:13]' (iter 8/10)
## INFO  [08:02:11.133] [mlr3] Applying learner 'regr.svm' on task 'PredictionSet[1:13]' (iter 9/10)
## INFO  [08:02:11.150] [mlr3] Applying learner 'regr.svm' on task 'PredictionSet[1:13]' (iter 10/10)
## INFO  [08:02:11.165] [mlr3] Finished benchmark
## INFO  [08:02:11.195] [bbotk] Result of batch 7:
## INFO  [08:02:11.196] [bbotk]  tolerance    gamma   epsilon           type kernel regr.rmse warnings errors
## INFO  [08:02:11.196] [bbotk]  0.7073571 28.57186 0.2142857 eps-regression radial  4.591775        0      0
## INFO  [08:02:11.196] [bbotk]  runtime_learners                                uhash
## INFO  [08:02:11.196] [bbotk]             0.062 2a60eb3b-2aee-4547-817f-0929d7ba1855
## INFO  [08:02:11.198] [bbotk] Evaluating 1 configuration(s)
## INFO  [08:02:11.214] [mlr3] Running benchmark with 10 resampling iterations
## INFO  [08:02:11.219] [mlr3] Applying learner 'regr.svm' on task 'PredictionSet[1:13]' (iter 1/10)
## INFO  [08:02:11.234] [mlr3] Applying learner 'regr.svm' on task 'PredictionSet[1:13]' (iter 2/10)
## INFO  [08:02:11.249] [mlr3] Applying learner 'regr.svm' on task 'PredictionSet[1:13]' (iter 3/10)
## INFO  [08:02:11.264] [mlr3] Applying learner 'regr.svm' on task 'PredictionSet[1:13]' (iter 4/10)
## INFO  [08:02:11.279] [mlr3] Applying learner 'regr.svm' on task 'PredictionSet[1:13]' (iter 5/10)
## INFO  [08:02:11.296] [mlr3] Applying learner 'regr.svm' on task 'PredictionSet[1:13]' (iter 6/10)
## INFO  [08:02:11.311] [mlr3] Applying learner 'regr.svm' on task 'PredictionSet[1:13]' (iter 7/10)
## INFO  [08:02:11.326] [mlr3] Applying learner 'regr.svm' on task 'PredictionSet[1:13]' (iter 8/10)
## INFO  [08:02:11.341] [mlr3] Applying learner 'regr.svm' on task 'PredictionSet[1:13]' (iter 9/10)
## INFO  [08:02:11.356] [mlr3] Applying learner 'regr.svm' on task 'PredictionSet[1:13]' (iter 10/10)
## INFO  [08:02:11.373] [mlr3] Finished benchmark
## INFO  [08:02:11.410] [bbotk] Result of batch 8:
## INFO  [08:02:11.411] [bbotk]  tolerance    gamma   epsilon           type kernel regr.rmse warnings errors
## INFO  [08:02:11.411] [bbotk]  0.7715714 7.143714 0.2714286 eps-regression radial  4.517133        0      0
## INFO  [08:02:11.411] [bbotk]  runtime_learners                                uhash
## INFO  [08:02:11.411] [bbotk]             0.052 c98bcfb1-a48a-4ed6-bb9f-21b4bfe77519
## INFO  [08:02:11.413] [bbotk] Evaluating 1 configuration(s)
## INFO  [08:02:11.428] [mlr3] Running benchmark with 10 resampling iterations
## INFO  [08:02:11.433] [mlr3] Applying learner 'regr.svm' on task 'PredictionSet[1:13]' (iter 1/10)
## INFO  [08:02:11.450] [mlr3] Applying learner 'regr.svm' on task 'PredictionSet[1:13]' (iter 2/10)
## INFO  [08:02:11.466] [mlr3] Applying learner 'regr.svm' on task 'PredictionSet[1:13]' (iter 3/10)
## INFO  [08:02:11.481] [mlr3] Applying learner 'regr.svm' on task 'PredictionSet[1:13]' (iter 4/10)
## INFO  [08:02:11.497] [mlr3] Applying learner 'regr.svm' on task 'PredictionSet[1:13]' (iter 5/10)
## INFO  [08:02:11.514] [mlr3] Applying learner 'regr.svm' on task 'PredictionSet[1:13]' (iter 6/10)
## INFO  [08:02:11.531] [mlr3] Applying learner 'regr.svm' on task 'PredictionSet[1:13]' (iter 7/10)
## INFO  [08:02:11.546] [mlr3] Applying learner 'regr.svm' on task 'PredictionSet[1:13]' (iter 8/10)
## INFO  [08:02:11.562] [mlr3] Applying learner 'regr.svm' on task 'PredictionSet[1:13]' (iter 9/10)
## INFO  [08:02:11.578] [mlr3] Applying learner 'regr.svm' on task 'PredictionSet[1:13]' (iter 10/10)
## INFO  [08:02:11.595] [mlr3] Finished benchmark
## INFO  [08:02:11.625] [bbotk] Result of batch 9:
## INFO  [08:02:11.626] [bbotk]  tolerance gamma   epsilon           type kernel regr.rmse warnings errors
## INFO  [08:02:11.626] [bbotk]  0.2578571    50 0.5571429 eps-regression radial  5.236905        0      0
## INFO  [08:02:11.626] [bbotk]  runtime_learners                                uhash
## INFO  [08:02:11.626] [bbotk]             0.065 47088c21-b6a9-48e1-adcf-c3168bcc9e5e
## INFO  [08:02:11.628] [bbotk] Evaluating 1 configuration(s)
## INFO  [08:02:11.642] [mlr3] Running benchmark with 10 resampling iterations
## INFO  [08:02:11.647] [mlr3] Applying learner 'regr.svm' on task 'PredictionSet[1:13]' (iter 1/10)
## INFO  [08:02:11.664] [mlr3] Applying learner 'regr.svm' on task 'PredictionSet[1:13]' (iter 2/10)
## INFO  [08:02:11.680] [mlr3] Applying learner 'regr.svm' on task 'PredictionSet[1:13]' (iter 3/10)
## INFO  [08:02:11.696] [mlr3] Applying learner 'regr.svm' on task 'PredictionSet[1:13]' (iter 4/10)
## INFO  [08:02:11.712] [mlr3] Applying learner 'regr.svm' on task 'PredictionSet[1:13]' (iter 5/10)
## INFO  [08:02:11.728] [mlr3] Applying learner 'regr.svm' on task 'PredictionSet[1:13]' (iter 6/10)
## INFO  [08:02:11.751] [mlr3] Applying learner 'regr.svm' on task 'PredictionSet[1:13]' (iter 7/10)
## INFO  [08:02:11.768] [mlr3] Applying learner 'regr.svm' on task 'PredictionSet[1:13]' (iter 8/10)
## INFO  [08:02:11.783] [mlr3] Applying learner 'regr.svm' on task 'PredictionSet[1:13]' (iter 9/10)
## INFO  [08:02:11.799] [mlr3] Applying learner 'regr.svm' on task 'PredictionSet[1:13]' (iter 10/10)
## INFO  [08:02:11.816] [mlr3] Finished benchmark
## INFO  [08:02:11.846] [bbotk] Result of batch 10:
## INFO  [08:02:11.848] [bbotk]  tolerance    gamma   epsilon           type kernel regr.rmse warnings errors
## INFO  [08:02:11.848] [bbotk]  0.6431429 39.28593 0.2714286 eps-regression radial  4.685028        0      0
## INFO  [08:02:11.848] [bbotk]  runtime_learners                                uhash
## INFO  [08:02:11.848] [bbotk]             0.064 7a3c1a01-bab2-4b09-b200-35da40ed86d4
## INFO  [08:02:11.853] [bbotk] Finished optimizing after 10 evaluation(s)
## INFO  [08:02:11.853] [bbotk] Result:
## INFO  [08:02:11.854] [bbotk]  tolerance    gamma   epsilon           type kernel learner_param_vals
## INFO  [08:02:11.854] [bbotk]      0.001 32.14321 0.1571429 eps-regression radial          <list[5]>
## INFO  [08:02:11.854] [bbotk]   x_domain regr.rmse
## INFO  [08:02:11.854] [bbotk]  <list[5]>  4.311199
## INFO  [08:02:11.995] [bbotk] Starting to optimize 3 parameter(s) with '<TunerGridSearch>' and '<TerminatorEvals> [n_evals=10, k=0]'
## INFO  [08:02:11.998] [bbotk] Evaluating 1 configuration(s)
## INFO  [08:02:12.008] [mlr3] Running benchmark with 10 resampling iterations
## INFO  [08:02:12.014] [mlr3] Applying learner 'regr.kknn' on task 'PredictionSet[1:13]' (iter 1/10)
## INFO  [08:02:12.035] [mlr3] Applying learner 'regr.kknn' on task 'PredictionSet[1:13]' (iter 2/10)
## INFO  [08:02:12.055] [mlr3] Applying learner 'regr.kknn' on task 'PredictionSet[1:13]' (iter 3/10)
## INFO  [08:02:12.082] [mlr3] Applying learner 'regr.kknn' on task 'PredictionSet[1:13]' (iter 4/10)
## INFO  [08:02:12.102] [mlr3] Applying learner 'regr.kknn' on task 'PredictionSet[1:13]' (iter 5/10)
## INFO  [08:02:12.124] [mlr3] Applying learner 'regr.kknn' on task 'PredictionSet[1:13]' (iter 6/10)
## INFO  [08:02:12.144] [mlr3] Applying learner 'regr.kknn' on task 'PredictionSet[1:13]' (iter 7/10)
## INFO  [08:02:12.164] [mlr3] Applying learner 'regr.kknn' on task 'PredictionSet[1:13]' (iter 8/10)
## INFO  [08:02:12.184] [mlr3] Applying learner 'regr.kknn' on task 'PredictionSet[1:13]' (iter 9/10)
## INFO  [08:02:12.205] [mlr3] Applying learner 'regr.kknn' on task 'PredictionSet[1:13]' (iter 10/10)
## INFO  [08:02:12.225] [mlr3] Finished benchmark
## INFO  [08:02:12.253] [bbotk] Result of batch 1:
## INFO  [08:02:12.255] [bbotk]   k distance     kernel regr.rmse warnings errors runtime_learners
## INFO  [08:02:12.255] [bbotk]  18 5.785714 triangular  4.561065        0      0            0.104
## INFO  [08:02:12.255] [bbotk]                                 uhash
## INFO  [08:02:12.255] [bbotk]  34faeaa5-9ef1-412e-9af8-bb0057d50905
## INFO  [08:02:12.256] [bbotk] Evaluating 1 configuration(s)
## INFO  [08:02:12.267] [mlr3] Running benchmark with 10 resampling iterations
## INFO  [08:02:12.272] [mlr3] Applying learner 'regr.kknn' on task 'PredictionSet[1:13]' (iter 1/10)
## INFO  [08:02:12.293] [mlr3] Applying learner 'regr.kknn' on task 'PredictionSet[1:13]' (iter 2/10)
## INFO  [08:02:12.314] [mlr3] Applying learner 'regr.kknn' on task 'PredictionSet[1:13]' (iter 3/10)
## INFO  [08:02:12.336] [mlr3] Applying learner 'regr.kknn' on task 'PredictionSet[1:13]' (iter 4/10)
## INFO  [08:02:12.357] [mlr3] Applying learner 'regr.kknn' on task 'PredictionSet[1:13]' (iter 5/10)
## INFO  [08:02:12.378] [mlr3] Applying learner 'regr.kknn' on task 'PredictionSet[1:13]' (iter 6/10)
## INFO  [08:02:12.398] [mlr3] Applying learner 'regr.kknn' on task 'PredictionSet[1:13]' (iter 7/10)
## INFO  [08:02:12.421] [mlr3] Applying learner 'regr.kknn' on task 'PredictionSet[1:13]' (iter 8/10)
## INFO  [08:02:12.441] [mlr3] Applying learner 'regr.kknn' on task 'PredictionSet[1:13]' (iter 9/10)
## INFO  [08:02:12.468] [mlr3] Applying learner 'regr.kknn' on task 'PredictionSet[1:13]' (iter 10/10)
## INFO  [08:02:12.489] [mlr3] Finished benchmark
## INFO  [08:02:12.519] [bbotk] Result of batch 2:
## INFO  [08:02:12.521] [bbotk]   k distance kernel regr.rmse warnings errors runtime_learners
## INFO  [08:02:12.521] [bbotk]  15      0.1   rank  4.141792        0      0            0.108
## INFO  [08:02:12.521] [bbotk]                                 uhash
## INFO  [08:02:12.521] [bbotk]  8d1063e0-e1ea-48a8-ad3b-ca3ec8fdc070
## INFO  [08:02:12.522] [bbotk] Evaluating 1 configuration(s)
## INFO  [08:02:12.532] [mlr3] Running benchmark with 10 resampling iterations
## INFO  [08:02:12.537] [mlr3] Applying learner 'regr.kknn' on task 'PredictionSet[1:13]' (iter 1/10)
## INFO  [08:02:12.557] [mlr3] Applying learner 'regr.kknn' on task 'PredictionSet[1:13]' (iter 2/10)
## INFO  [08:02:12.579] [mlr3] Applying learner 'regr.kknn' on task 'PredictionSet[1:13]' (iter 3/10)
## INFO  [08:02:12.599] [mlr3] Applying learner 'regr.kknn' on task 'PredictionSet[1:13]' (iter 4/10)
## INFO  [08:02:12.619] [mlr3] Applying learner 'regr.kknn' on task 'PredictionSet[1:13]' (iter 5/10)
## INFO  [08:02:12.640] [mlr3] Applying learner 'regr.kknn' on task 'PredictionSet[1:13]' (iter 6/10)
## INFO  [08:02:12.660] [mlr3] Applying learner 'regr.kknn' on task 'PredictionSet[1:13]' (iter 7/10)
## INFO  [08:02:12.680] [mlr3] Applying learner 'regr.kknn' on task 'PredictionSet[1:13]' (iter 8/10)
## INFO  [08:02:12.699] [mlr3] Applying learner 'regr.kknn' on task 'PredictionSet[1:13]' (iter 9/10)
## INFO  [08:02:12.721] [mlr3] Applying learner 'regr.kknn' on task 'PredictionSet[1:13]' (iter 10/10)
## INFO  [08:02:12.740] [mlr3] Finished benchmark
## INFO  [08:02:12.771] [bbotk] Result of batch 3:
## INFO  [08:02:12.772] [bbotk]  k distance   kernel regr.rmse warnings errors runtime_learners
## INFO  [08:02:12.772] [bbotk]  6 12.89286 gaussian  4.591968        0      0            0.098
## INFO  [08:02:12.772] [bbotk]                                 uhash
## INFO  [08:02:12.772] [bbotk]  5a552968-3792-4f80-bca1-f7bc2a31787f
## INFO  [08:02:12.774] [bbotk] Evaluating 1 configuration(s)
## INFO  [08:02:12.786] [mlr3] Running benchmark with 10 resampling iterations
## INFO  [08:02:12.791] [mlr3] Applying learner 'regr.kknn' on task 'PredictionSet[1:13]' (iter 1/10)
## INFO  [08:02:12.817] [mlr3] Applying learner 'regr.kknn' on task 'PredictionSet[1:13]' (iter 2/10)
## INFO  [08:02:12.837] [mlr3] Applying learner 'regr.kknn' on task 'PredictionSet[1:13]' (iter 3/10)
## INFO  [08:02:12.858] [mlr3] Applying learner 'regr.kknn' on task 'PredictionSet[1:13]' (iter 4/10)
## INFO  [08:02:12.879] [mlr3] Applying learner 'regr.kknn' on task 'PredictionSet[1:13]' (iter 5/10)
## INFO  [08:02:12.902] [mlr3] Applying learner 'regr.kknn' on task 'PredictionSet[1:13]' (iter 6/10)
## INFO  [08:02:12.922] [mlr3] Applying learner 'regr.kknn' on task 'PredictionSet[1:13]' (iter 7/10)
## INFO  [08:02:12.944] [mlr3] Applying learner 'regr.kknn' on task 'PredictionSet[1:13]' (iter 8/10)
## INFO  [08:02:12.964] [mlr3] Applying learner 'regr.kknn' on task 'PredictionSet[1:13]' (iter 9/10)
## INFO  [08:02:12.984] [mlr3] Applying learner 'regr.kknn' on task 'PredictionSet[1:13]' (iter 10/10)
## INFO  [08:02:13.004] [mlr3] Finished benchmark
## INFO  [08:02:13.036] [bbotk] Result of batch 4:
## INFO  [08:02:13.037] [bbotk]   k distance   kernel regr.rmse warnings errors runtime_learners
## INFO  [08:02:13.037] [bbotk]  13       20 biweight  4.645491        0      0             0.11
## INFO  [08:02:13.037] [bbotk]                                 uhash
## INFO  [08:02:13.037] [bbotk]  08ef0d27-d10b-443f-9c10-52107ca189cf
## INFO  [08:02:13.038] [bbotk] Evaluating 1 configuration(s)
## INFO  [08:02:13.049] [mlr3] Running benchmark with 10 resampling iterations
## INFO  [08:02:13.053] [mlr3] Applying learner 'regr.kknn' on task 'PredictionSet[1:13]' (iter 1/10)
## INFO  [08:02:13.073] [mlr3] Applying learner 'regr.kknn' on task 'PredictionSet[1:13]' (iter 2/10)
## INFO  [08:02:13.094] [mlr3] Applying learner 'regr.kknn' on task 'PredictionSet[1:13]' (iter 3/10)
## INFO  [08:02:13.114] [mlr3] Applying learner 'regr.kknn' on task 'PredictionSet[1:13]' (iter 4/10)
## INFO  [08:02:13.133] [mlr3] Applying learner 'regr.kknn' on task 'PredictionSet[1:13]' (iter 5/10)
## INFO  [08:02:13.153] [mlr3] Applying learner 'regr.kknn' on task 'PredictionSet[1:13]' (iter 6/10)
## INFO  [08:02:13.175] [mlr3] Applying learner 'regr.kknn' on task 'PredictionSet[1:13]' (iter 7/10)
## INFO  [08:02:13.201] [mlr3] Applying learner 'regr.kknn' on task 'PredictionSet[1:13]' (iter 8/10)
## INFO  [08:02:13.221] [mlr3] Applying learner 'regr.kknn' on task 'PredictionSet[1:13]' (iter 9/10)
## INFO  [08:02:13.242] [mlr3] Applying learner 'regr.kknn' on task 'PredictionSet[1:13]' (iter 10/10)
## INFO  [08:02:13.261] [mlr3] Finished benchmark
## INFO  [08:02:13.291] [bbotk] Result of batch 5:
## INFO  [08:02:13.293] [bbotk]  k distance      kernel regr.rmse warnings errors runtime_learners
## INFO  [08:02:13.293] [bbotk]  1 5.785714 rectangular  4.151686        0      0            0.104
## INFO  [08:02:13.293] [bbotk]                                 uhash
## INFO  [08:02:13.293] [bbotk]  e6ccf6f6-702a-4b98-9215-f372d8061925
## INFO  [08:02:13.294] [bbotk] Evaluating 1 configuration(s)
## INFO  [08:02:13.305] [mlr3] Running benchmark with 10 resampling iterations
## INFO  [08:02:13.311] [mlr3] Applying learner 'regr.kknn' on task 'PredictionSet[1:13]' (iter 1/10)
## INFO  [08:02:13.331] [mlr3] Applying learner 'regr.kknn' on task 'PredictionSet[1:13]' (iter 2/10)
## INFO  [08:02:13.351] [mlr3] Applying learner 'regr.kknn' on task 'PredictionSet[1:13]' (iter 3/10)
## INFO  [08:02:13.370] [mlr3] Applying learner 'regr.kknn' on task 'PredictionSet[1:13]' (iter 4/10)
## INFO  [08:02:13.392] [mlr3] Applying learner 'regr.kknn' on task 'PredictionSet[1:13]' (iter 5/10)
## INFO  [08:02:13.412] [mlr3] Applying learner 'regr.kknn' on task 'PredictionSet[1:13]' (iter 6/10)
## INFO  [08:02:13.432] [mlr3] Applying learner 'regr.kknn' on task 'PredictionSet[1:13]' (iter 7/10)
## INFO  [08:02:13.451] [mlr3] Applying learner 'regr.kknn' on task 'PredictionSet[1:13]' (iter 8/10)
## INFO  [08:02:13.473] [mlr3] Applying learner 'regr.kknn' on task 'PredictionSet[1:13]' (iter 9/10)
## INFO  [08:02:13.493] [mlr3] Applying learner 'regr.kknn' on task 'PredictionSet[1:13]' (iter 10/10)
## INFO  [08:02:13.512] [mlr3] Finished benchmark
## INFO  [08:02:13.545] [bbotk] Result of batch 6:
## INFO  [08:02:13.546] [bbotk]   k distance     kernel regr.rmse warnings errors runtime_learners
## INFO  [08:02:13.546] [bbotk]  10    10.05 triangular  4.506027        0      0            0.096
## INFO  [08:02:13.546] [bbotk]                                 uhash
## INFO  [08:02:13.546] [bbotk]  bf1c9876-1c80-47b4-8963-26e2950e9dbb
## INFO  [08:02:13.548] [bbotk] Evaluating 1 configuration(s)
## INFO  [08:02:13.564] [mlr3] Running benchmark with 10 resampling iterations
## INFO  [08:02:13.569] [mlr3] Applying learner 'regr.kknn' on task 'PredictionSet[1:13]' (iter 1/10)
## INFO  [08:02:13.589] [mlr3] Applying learner 'regr.kknn' on task 'PredictionSet[1:13]' (iter 2/10)
## INFO  [08:02:13.610] [mlr3] Applying learner 'regr.kknn' on task 'PredictionSet[1:13]' (iter 3/10)
## INFO  [08:02:13.631] [mlr3] Applying learner 'regr.kknn' on task 'PredictionSet[1:13]' (iter 4/10)
## INFO  [08:02:13.651] [mlr3] Applying learner 'regr.kknn' on task 'PredictionSet[1:13]' (iter 5/10)
## INFO  [08:02:13.671] [mlr3] Applying learner 'regr.kknn' on task 'PredictionSet[1:13]' (iter 6/10)
## INFO  [08:02:13.692] [mlr3] Applying learner 'regr.kknn' on task 'PredictionSet[1:13]' (iter 7/10)
## INFO  [08:02:13.713] [mlr3] Applying learner 'regr.kknn' on task 'PredictionSet[1:13]' (iter 8/10)
## INFO  [08:02:13.733] [mlr3] Applying learner 'regr.kknn' on task 'PredictionSet[1:13]' (iter 9/10)
## INFO  [08:02:13.753] [mlr3] Applying learner 'regr.kknn' on task 'PredictionSet[1:13]' (iter 10/10)
## INFO  [08:02:13.774] [mlr3] Finished benchmark
## INFO  [08:02:13.804] [bbotk] Result of batch 7:
## INFO  [08:02:13.806] [bbotk]   k distance       kernel regr.rmse warnings errors runtime_learners
## INFO  [08:02:13.806] [bbotk]  18 15.73571 epanechnikov  4.680778        0      0            0.101
## INFO  [08:02:13.806] [bbotk]                                 uhash
## INFO  [08:02:13.806] [bbotk]  628b7a14-eb59-4b5a-83aa-25d2561fbd60
## INFO  [08:02:13.807] [bbotk] Evaluating 1 configuration(s)
## INFO  [08:02:13.817] [mlr3] Running benchmark with 10 resampling iterations
## INFO  [08:02:13.822] [mlr3] Applying learner 'regr.kknn' on task 'PredictionSet[1:13]' (iter 1/10)
## INFO  [08:02:13.843] [mlr3] Applying learner 'regr.kknn' on task 'PredictionSet[1:13]' (iter 2/10)
## INFO  [08:02:13.863] [mlr3] Applying learner 'regr.kknn' on task 'PredictionSet[1:13]' (iter 3/10)
## INFO  [08:02:13.883] [mlr3] Applying learner 'regr.kknn' on task 'PredictionSet[1:13]' (iter 4/10)
## INFO  [08:02:13.904] [mlr3] Applying learner 'regr.kknn' on task 'PredictionSet[1:13]' (iter 5/10)
## INFO  [08:02:13.931] [mlr3] Applying learner 'regr.kknn' on task 'PredictionSet[1:13]' (iter 6/10)
## INFO  [08:02:13.951] [mlr3] Applying learner 'regr.kknn' on task 'PredictionSet[1:13]' (iter 7/10)
## INFO  [08:02:13.971] [mlr3] Applying learner 'regr.kknn' on task 'PredictionSet[1:13]' (iter 8/10)
## INFO  [08:02:13.992] [mlr3] Applying learner 'regr.kknn' on task 'PredictionSet[1:13]' (iter 9/10)
## INFO  [08:02:14.012] [mlr3] Applying learner 'regr.kknn' on task 'PredictionSet[1:13]' (iter 10/10)
## INFO  [08:02:14.032] [mlr3] Finished benchmark
## INFO  [08:02:14.064] [bbotk] Result of batch 8:
## INFO  [08:02:14.065] [bbotk]  k distance   kernel regr.rmse warnings errors runtime_learners
## INFO  [08:02:14.065] [bbotk]  2 2.942857 gaussian  4.135906        0      0            0.106
## INFO  [08:02:14.065] [bbotk]                                 uhash
## INFO  [08:02:14.065] [bbotk]  4653502f-cf55-463d-8cc6-83e9d0e7710d
## INFO  [08:02:14.067] [bbotk] Evaluating 1 configuration(s)
## INFO  [08:02:14.077] [mlr3] Running benchmark with 10 resampling iterations
## INFO  [08:02:14.082] [mlr3] Applying learner 'regr.kknn' on task 'PredictionSet[1:13]' (iter 1/10)
## INFO  [08:02:14.101] [mlr3] Applying learner 'regr.kknn' on task 'PredictionSet[1:13]' (iter 2/10)
## INFO  [08:02:14.121] [mlr3] Applying learner 'regr.kknn' on task 'PredictionSet[1:13]' (iter 3/10)
## INFO  [08:02:14.142] [mlr3] Applying learner 'regr.kknn' on task 'PredictionSet[1:13]' (iter 4/10)
## INFO  [08:02:14.162] [mlr3] Applying learner 'regr.kknn' on task 'PredictionSet[1:13]' (iter 5/10)
## INFO  [08:02:14.182] [mlr3] Applying learner 'regr.kknn' on task 'PredictionSet[1:13]' (iter 6/10)
## INFO  [08:02:14.202] [mlr3] Applying learner 'regr.kknn' on task 'PredictionSet[1:13]' (iter 7/10)
## INFO  [08:02:14.224] [mlr3] Applying learner 'regr.kknn' on task 'PredictionSet[1:13]' (iter 8/10)
## INFO  [08:02:14.244] [mlr3] Applying learner 'regr.kknn' on task 'PredictionSet[1:13]' (iter 9/10)
## INFO  [08:02:14.264] [mlr3] Applying learner 'regr.kknn' on task 'PredictionSet[1:13]' (iter 10/10)
## INFO  [08:02:14.285] [mlr3] Finished benchmark
## INFO  [08:02:14.323] [bbotk] Result of batch 9:
## INFO  [08:02:14.324] [bbotk]  k distance    kernel regr.rmse warnings errors runtime_learners
## INFO  [08:02:14.324] [bbotk]  1 18.57857 triweight  7.034419        0      0              0.1
## INFO  [08:02:14.324] [bbotk]                                 uhash
## INFO  [08:02:14.324] [bbotk]  1c4ab270-ce16-4867-ad1b-4311ecd98252
## INFO  [08:02:14.325] [bbotk] Evaluating 1 configuration(s)
## INFO  [08:02:14.335] [mlr3] Running benchmark with 10 resampling iterations
## INFO  [08:02:14.340] [mlr3] Applying learner 'regr.kknn' on task 'PredictionSet[1:13]' (iter 1/10)
## INFO  [08:02:14.361] [mlr3] Applying learner 'regr.kknn' on task 'PredictionSet[1:13]' (iter 2/10)
## INFO  [08:02:14.381] [mlr3] Applying learner 'regr.kknn' on task 'PredictionSet[1:13]' (iter 3/10)
## INFO  [08:02:14.401] [mlr3] Applying learner 'regr.kknn' on task 'PredictionSet[1:13]' (iter 4/10)
## INFO  [08:02:14.421] [mlr3] Applying learner 'regr.kknn' on task 'PredictionSet[1:13]' (iter 5/10)
## INFO  [08:02:14.443] [mlr3] Applying learner 'regr.kknn' on task 'PredictionSet[1:13]' (iter 6/10)
## INFO  [08:02:14.463] [mlr3] Applying learner 'regr.kknn' on task 'PredictionSet[1:13]' (iter 7/10)
## INFO  [08:02:14.482] [mlr3] Applying learner 'regr.kknn' on task 'PredictionSet[1:13]' (iter 8/10)
## INFO  [08:02:14.502] [mlr3] Applying learner 'regr.kknn' on task 'PredictionSet[1:13]' (iter 9/10)
## INFO  [08:02:14.524] [mlr3] Applying learner 'regr.kknn' on task 'PredictionSet[1:13]' (iter 10/10)
## INFO  [08:02:14.543] [mlr3] Finished benchmark
## INFO  [08:02:14.575] [bbotk] Result of batch 10:
## INFO  [08:02:14.576] [bbotk]  k distance   kernel regr.rmse warnings errors runtime_learners
## INFO  [08:02:14.576] [bbotk]  3 12.89286 gaussian   4.44831        0      0            0.104
## INFO  [08:02:14.576] [bbotk]                                 uhash
## INFO  [08:02:14.576] [bbotk]  5988d1ef-10bc-4464-a24f-be5177d26621
## INFO  [08:02:14.581] [bbotk] Finished optimizing after 10 evaluation(s)
## INFO  [08:02:14.582] [bbotk] Result:
## INFO  [08:02:14.583] [bbotk]  k distance   kernel learner_param_vals  x_domain regr.rmse
## INFO  [08:02:14.583] [bbotk]  2 2.942857 gaussian          <list[3]> <list[3]>  4.135906
## INFO  [08:02:14.793] [bbotk] Starting to optimize 2 parameter(s) with '<TunerGridSearch>' and '<TerminatorEvals> [n_evals=10, k=0]'
## INFO  [08:02:14.796] [bbotk] Evaluating 1 configuration(s)
## INFO  [08:02:14.818] [mlr3] Running benchmark with 10 resampling iterations
## INFO  [08:02:14.823] [mlr3] Applying learner 'regr.ranger' on task 'PredictionSet[1:13]' (iter 1/10)
## INFO  [08:02:14.863] [mlr3] Applying learner 'regr.ranger' on task 'PredictionSet[1:13]' (iter 2/10)
## INFO  [08:02:14.905] [mlr3] Applying learner 'regr.ranger' on task 'PredictionSet[1:13]' (iter 3/10)
## INFO  [08:02:14.945] [mlr3] Applying learner 'regr.ranger' on task 'PredictionSet[1:13]' (iter 4/10)
## INFO  [08:02:14.987] [mlr3] Applying learner 'regr.ranger' on task 'PredictionSet[1:13]' (iter 5/10)
## INFO  [08:02:15.027] [mlr3] Applying learner 'regr.ranger' on task 'PredictionSet[1:13]' (iter 6/10)
## INFO  [08:02:15.078] [mlr3] Applying learner 'regr.ranger' on task 'PredictionSet[1:13]' (iter 7/10)
## INFO  [08:02:15.120] [mlr3] Applying learner 'regr.ranger' on task 'PredictionSet[1:13]' (iter 8/10)
## INFO  [08:02:15.160] [mlr3] Applying learner 'regr.ranger' on task 'PredictionSet[1:13]' (iter 9/10)
## INFO  [08:02:15.201] [mlr3] Applying learner 'regr.ranger' on task 'PredictionSet[1:13]' (iter 10/10)
## INFO  [08:02:15.241] [mlr3] Finished benchmark
## INFO  [08:02:15.271] [bbotk] Result of batch 1:
## INFO  [08:02:15.273] [bbotk]  max.depth num.trees regr.rmse warnings errors runtime_learners
## INFO  [08:02:15.273] [bbotk]         20       369  3.196576        0      0            0.309
## INFO  [08:02:15.273] [bbotk]                                 uhash
## INFO  [08:02:15.273] [bbotk]  78766e2c-0dc3-4eac-b418-e5889182733f
## INFO  [08:02:15.274] [bbotk] Evaluating 1 configuration(s)
## INFO  [08:02:15.295] [mlr3] Running benchmark with 10 resampling iterations
## INFO  [08:02:15.300] [mlr3] Applying learner 'regr.ranger' on task 'PredictionSet[1:13]' (iter 1/10)
## INFO  [08:02:15.338] [mlr3] Applying learner 'regr.ranger' on task 'PredictionSet[1:13]' (iter 2/10)
## INFO  [08:02:15.375] [mlr3] Applying learner 'regr.ranger' on task 'PredictionSet[1:13]' (iter 3/10)
## INFO  [08:02:15.413] [mlr3] Applying learner 'regr.ranger' on task 'PredictionSet[1:13]' (iter 4/10)
## INFO  [08:02:15.449] [mlr3] Applying learner 'regr.ranger' on task 'PredictionSet[1:13]' (iter 5/10)
## INFO  [08:02:15.487] [mlr3] Applying learner 'regr.ranger' on task 'PredictionSet[1:13]' (iter 6/10)
## INFO  [08:02:15.523] [mlr3] Applying learner 'regr.ranger' on task 'PredictionSet[1:13]' (iter 7/10)
## INFO  [08:02:15.562] [mlr3] Applying learner 'regr.ranger' on task 'PredictionSet[1:13]' (iter 8/10)
## INFO  [08:02:15.598] [mlr3] Applying learner 'regr.ranger' on task 'PredictionSet[1:13]' (iter 9/10)
## INFO  [08:02:15.636] [mlr3] Applying learner 'regr.ranger' on task 'PredictionSet[1:13]' (iter 10/10)
## INFO  [08:02:15.672] [mlr3] Finished benchmark
## INFO  [08:02:15.713] [bbotk] Result of batch 2:
## INFO  [08:02:15.715] [bbotk]  max.depth num.trees regr.rmse warnings errors runtime_learners
## INFO  [08:02:15.715] [bbotk]         15       316  3.190571        0      0            0.267
## INFO  [08:02:15.715] [bbotk]                                 uhash
## INFO  [08:02:15.715] [bbotk]  2fc3cd3d-eef9-479c-bee9-daf09faaec38
## INFO  [08:02:15.716] [bbotk] Evaluating 1 configuration(s)
## INFO  [08:02:15.737] [mlr3] Running benchmark with 10 resampling iterations
## INFO  [08:02:15.742] [mlr3] Applying learner 'regr.ranger' on task 'PredictionSet[1:13]' (iter 1/10)
## INFO  [08:02:15.779] [mlr3] Applying learner 'regr.ranger' on task 'PredictionSet[1:13]' (iter 2/10)
## INFO  [08:02:15.817] [mlr3] Applying learner 'regr.ranger' on task 'PredictionSet[1:13]' (iter 3/10)
## INFO  [08:02:15.853] [mlr3] Applying learner 'regr.ranger' on task 'PredictionSet[1:13]' (iter 4/10)
## INFO  [08:02:15.891] [mlr3] Applying learner 'regr.ranger' on task 'PredictionSet[1:13]' (iter 5/10)
## INFO  [08:02:15.927] [mlr3] Applying learner 'regr.ranger' on task 'PredictionSet[1:13]' (iter 6/10)
## INFO  [08:02:15.965] [mlr3] Applying learner 'regr.ranger' on task 'PredictionSet[1:13]' (iter 7/10)
## INFO  [08:02:16.002] [mlr3] Applying learner 'regr.ranger' on task 'PredictionSet[1:13]' (iter 8/10)
## INFO  [08:02:16.039] [mlr3] Applying learner 'regr.ranger' on task 'PredictionSet[1:13]' (iter 9/10)
## INFO  [08:02:16.076] [mlr3] Applying learner 'regr.ranger' on task 'PredictionSet[1:13]' (iter 10/10)
## INFO  [08:02:16.114] [mlr3] Finished benchmark
## INFO  [08:02:16.144] [bbotk] Result of batch 3:
## INFO  [08:02:16.145] [bbotk]  max.depth num.trees regr.rmse warnings errors runtime_learners
## INFO  [08:02:16.145] [bbotk]         14       316  3.193766        0      0            0.269
## INFO  [08:02:16.145] [bbotk]                                 uhash
## INFO  [08:02:16.145] [bbotk]  5e9f2c94-dd4c-458a-912a-0956f318fdfd
## INFO  [08:02:16.146] [bbotk] Evaluating 1 configuration(s)
## INFO  [08:02:16.168] [mlr3] Running benchmark with 10 resampling iterations
## INFO  [08:02:16.173] [mlr3] Applying learner 'regr.ranger' on task 'PredictionSet[1:13]' (iter 1/10)
## INFO  [08:02:16.211] [mlr3] Applying learner 'regr.ranger' on task 'PredictionSet[1:13]' (iter 2/10)
## INFO  [08:02:16.251] [mlr3] Applying learner 'regr.ranger' on task 'PredictionSet[1:13]' (iter 3/10)
## INFO  [08:02:16.290] [mlr3] Applying learner 'regr.ranger' on task 'PredictionSet[1:13]' (iter 4/10)
## INFO  [08:02:16.338] [mlr3] Applying learner 'regr.ranger' on task 'PredictionSet[1:13]' (iter 5/10)
## INFO  [08:02:16.377] [mlr3] Applying learner 'regr.ranger' on task 'PredictionSet[1:13]' (iter 6/10)
## INFO  [08:02:16.416] [mlr3] Applying learner 'regr.ranger' on task 'PredictionSet[1:13]' (iter 7/10)
## INFO  [08:02:16.455] [mlr3] Applying learner 'regr.ranger' on task 'PredictionSet[1:13]' (iter 8/10)
## INFO  [08:02:16.494] [mlr3] Applying learner 'regr.ranger' on task 'PredictionSet[1:13]' (iter 9/10)
## INFO  [08:02:16.533] [mlr3] Applying learner 'regr.ranger' on task 'PredictionSet[1:13]' (iter 10/10)
## INFO  [08:02:16.572] [mlr3] Finished benchmark
## INFO  [08:02:16.603] [bbotk] Result of batch 4:
## INFO  [08:02:16.604] [bbotk]  max.depth num.trees regr.rmse warnings errors runtime_learners
## INFO  [08:02:16.604] [bbotk]         18       343  3.177151        0      0            0.285
## INFO  [08:02:16.604] [bbotk]                                 uhash
## INFO  [08:02:16.604] [bbotk]  366d7871-afe1-4029-af9e-4579a34d802c
## INFO  [08:02:16.606] [bbotk] Evaluating 1 configuration(s)
## INFO  [08:02:16.627] [mlr3] Running benchmark with 10 resampling iterations
## INFO  [08:02:16.632] [mlr3] Applying learner 'regr.ranger' on task 'PredictionSet[1:13]' (iter 1/10)
## INFO  [08:02:16.670] [mlr3] Applying learner 'regr.ranger' on task 'PredictionSet[1:13]' (iter 2/10)
## INFO  [08:02:16.708] [mlr3] Applying learner 'regr.ranger' on task 'PredictionSet[1:13]' (iter 3/10)
## INFO  [08:02:16.746] [mlr3] Applying learner 'regr.ranger' on task 'PredictionSet[1:13]' (iter 4/10)
## INFO  [08:02:16.786] [mlr3] Applying learner 'regr.ranger' on task 'PredictionSet[1:13]' (iter 5/10)
## INFO  [08:02:16.824] [mlr3] Applying learner 'regr.ranger' on task 'PredictionSet[1:13]' (iter 6/10)
## INFO  [08:02:16.863] [mlr3] Applying learner 'regr.ranger' on task 'PredictionSet[1:13]' (iter 7/10)
## INFO  [08:02:16.902] [mlr3] Applying learner 'regr.ranger' on task 'PredictionSet[1:13]' (iter 8/10)
## INFO  [08:02:16.941] [mlr3] Applying learner 'regr.ranger' on task 'PredictionSet[1:13]' (iter 9/10)
## INFO  [08:02:16.980] [mlr3] Applying learner 'regr.ranger' on task 'PredictionSet[1:13]' (iter 10/10)
## INFO  [08:02:17.029] [mlr3] Finished benchmark
## INFO  [08:02:17.061] [bbotk] Result of batch 5:
## INFO  [08:02:17.063] [bbotk]  max.depth num.trees regr.rmse warnings errors runtime_learners
## INFO  [08:02:17.063] [bbotk]         20       343  3.219656        0      0            0.293
## INFO  [08:02:17.063] [bbotk]                                 uhash
## INFO  [08:02:17.063] [bbotk]  a3745cfe-346c-4881-97db-9429de27c014
## INFO  [08:02:17.064] [bbotk] Evaluating 1 configuration(s)
## INFO  [08:02:17.085] [mlr3] Running benchmark with 10 resampling iterations
## INFO  [08:02:17.090] [mlr3] Applying learner 'regr.ranger' on task 'PredictionSet[1:13]' (iter 1/10)
## INFO  [08:02:17.106] [mlr3] Applying learner 'regr.ranger' on task 'PredictionSet[1:13]' (iter 2/10)
## INFO  [08:02:17.122] [mlr3] Applying learner 'regr.ranger' on task 'PredictionSet[1:13]' (iter 3/10)
## INFO  [08:02:17.140] [mlr3] Applying learner 'regr.ranger' on task 'PredictionSet[1:13]' (iter 4/10)
## INFO  [08:02:17.157] [mlr3] Applying learner 'regr.ranger' on task 'PredictionSet[1:13]' (iter 5/10)
## INFO  [08:02:17.173] [mlr3] Applying learner 'regr.ranger' on task 'PredictionSet[1:13]' (iter 6/10)
## INFO  [08:02:17.189] [mlr3] Applying learner 'regr.ranger' on task 'PredictionSet[1:13]' (iter 7/10)
## INFO  [08:02:17.206] [mlr3] Applying learner 'regr.ranger' on task 'PredictionSet[1:13]' (iter 8/10)
## INFO  [08:02:17.223] [mlr3] Applying learner 'regr.ranger' on task 'PredictionSet[1:13]' (iter 9/10)
## INFO  [08:02:17.239] [mlr3] Applying learner 'regr.ranger' on task 'PredictionSet[1:13]' (iter 10/10)
## INFO  [08:02:17.255] [mlr3] Finished benchmark
## INFO  [08:02:17.287] [bbotk] Result of batch 6:
## INFO  [08:02:17.289] [bbotk]  max.depth num.trees regr.rmse warnings errors runtime_learners
## INFO  [08:02:17.289] [bbotk]          7        27  3.256355        0      0            0.065
## INFO  [08:02:17.289] [bbotk]                                 uhash
## INFO  [08:02:17.289] [bbotk]  bd9ec784-9c02-4117-8988-c718fef8bd49
## INFO  [08:02:17.290] [bbotk] Evaluating 1 configuration(s)
## INFO  [08:02:17.311] [mlr3] Running benchmark with 10 resampling iterations
## INFO  [08:02:17.316] [mlr3] Applying learner 'regr.ranger' on task 'PredictionSet[1:13]' (iter 1/10)
## INFO  [08:02:17.349] [mlr3] Applying learner 'regr.ranger' on task 'PredictionSet[1:13]' (iter 2/10)
## INFO  [08:02:17.376] [mlr3] Applying learner 'regr.ranger' on task 'PredictionSet[1:13]' (iter 3/10)
## INFO  [08:02:17.402] [mlr3] Applying learner 'regr.ranger' on task 'PredictionSet[1:13]' (iter 4/10)
## INFO  [08:02:17.428] [mlr3] Applying learner 'regr.ranger' on task 'PredictionSet[1:13]' (iter 5/10)
## INFO  [08:02:17.454] [mlr3] Applying learner 'regr.ranger' on task 'PredictionSet[1:13]' (iter 6/10)
## INFO  [08:02:17.480] [mlr3] Applying learner 'regr.ranger' on task 'PredictionSet[1:13]' (iter 7/10)
## INFO  [08:02:17.507] [mlr3] Applying learner 'regr.ranger' on task 'PredictionSet[1:13]' (iter 8/10)
## INFO  [08:02:17.533] [mlr3] Applying learner 'regr.ranger' on task 'PredictionSet[1:13]' (iter 9/10)
## INFO  [08:02:17.559] [mlr3] Applying learner 'regr.ranger' on task 'PredictionSet[1:13]' (iter 10/10)
## INFO  [08:02:17.585] [mlr3] Finished benchmark
## INFO  [08:02:17.617] [bbotk] Result of batch 7:
## INFO  [08:02:17.618] [bbotk]  max.depth num.trees regr.rmse warnings errors runtime_learners
## INFO  [08:02:17.618] [bbotk]         14       158  3.265485        0      0             0.16
## INFO  [08:02:17.618] [bbotk]                                 uhash
## INFO  [08:02:17.618] [bbotk]  802efc62-add3-414b-9d41-09564505ea10
## INFO  [08:02:17.619] [bbotk] Evaluating 1 configuration(s)
## INFO  [08:02:17.640] [mlr3] Running benchmark with 10 resampling iterations
## INFO  [08:02:17.645] [mlr3] Applying learner 'regr.ranger' on task 'PredictionSet[1:13]' (iter 1/10)
## INFO  [08:02:17.670] [mlr3] Applying learner 'regr.ranger' on task 'PredictionSet[1:13]' (iter 2/10)
## INFO  [08:02:17.693] [mlr3] Applying learner 'regr.ranger' on task 'PredictionSet[1:13]' (iter 3/10)
## INFO  [08:02:17.716] [mlr3] Applying learner 'regr.ranger' on task 'PredictionSet[1:13]' (iter 4/10)
## INFO  [08:02:17.741] [mlr3] Applying learner 'regr.ranger' on task 'PredictionSet[1:13]' (iter 5/10)
## INFO  [08:02:17.764] [mlr3] Applying learner 'regr.ranger' on task 'PredictionSet[1:13]' (iter 6/10)
## INFO  [08:02:17.787] [mlr3] Applying learner 'regr.ranger' on task 'PredictionSet[1:13]' (iter 7/10)
## INFO  [08:02:17.820] [mlr3] Applying learner 'regr.ranger' on task 'PredictionSet[1:13]' (iter 8/10)
## INFO  [08:02:17.843] [mlr3] Applying learner 'regr.ranger' on task 'PredictionSet[1:13]' (iter 9/10)
## INFO  [08:02:17.866] [mlr3] Applying learner 'regr.ranger' on task 'PredictionSet[1:13]' (iter 10/10)
## INFO  [08:02:17.890] [mlr3] Finished benchmark
## INFO  [08:02:17.921] [bbotk] Result of batch 8:
## INFO  [08:02:17.922] [bbotk]  max.depth num.trees regr.rmse warnings errors runtime_learners
## INFO  [08:02:17.922] [bbotk]          5       158  3.518019        0      0            0.127
## INFO  [08:02:17.922] [bbotk]                                 uhash
## INFO  [08:02:17.922] [bbotk]  3f2bb802-60d9-4d79-8967-4d7e29f67539
## INFO  [08:02:17.924] [bbotk] Evaluating 1 configuration(s)
## INFO  [08:02:17.945] [mlr3] Running benchmark with 10 resampling iterations
## INFO  [08:02:17.950] [mlr3] Applying learner 'regr.ranger' on task 'PredictionSet[1:13]' (iter 1/10)
## INFO  [08:02:17.975] [mlr3] Applying learner 'regr.ranger' on task 'PredictionSet[1:13]' (iter 2/10)
## INFO  [08:02:17.999] [mlr3] Applying learner 'regr.ranger' on task 'PredictionSet[1:13]' (iter 3/10)
## INFO  [08:02:18.023] [mlr3] Applying learner 'regr.ranger' on task 'PredictionSet[1:13]' (iter 4/10)
## INFO  [08:02:18.049] [mlr3] Applying learner 'regr.ranger' on task 'PredictionSet[1:13]' (iter 5/10)
## INFO  [08:02:18.073] [mlr3] Applying learner 'regr.ranger' on task 'PredictionSet[1:13]' (iter 6/10)
## INFO  [08:02:18.097] [mlr3] Applying learner 'regr.ranger' on task 'PredictionSet[1:13]' (iter 7/10)
## INFO  [08:02:18.123] [mlr3] Applying learner 'regr.ranger' on task 'PredictionSet[1:13]' (iter 8/10)
## INFO  [08:02:18.148] [mlr3] Applying learner 'regr.ranger' on task 'PredictionSet[1:13]' (iter 9/10)
## INFO  [08:02:18.172] [mlr3] Applying learner 'regr.ranger' on task 'PredictionSet[1:13]' (iter 10/10)
## INFO  [08:02:18.198] [mlr3] Finished benchmark
## INFO  [08:02:18.236] [bbotk] Result of batch 9:
## INFO  [08:02:18.237] [bbotk]  max.depth num.trees regr.rmse warnings errors runtime_learners
## INFO  [08:02:18.237] [bbotk]          1       422  5.076654        0      0            0.145
## INFO  [08:02:18.237] [bbotk]                                 uhash
## INFO  [08:02:18.237] [bbotk]  3d8a2960-0468-4076-9a0d-cc6c1ad30209
## INFO  [08:02:18.239] [bbotk] Evaluating 1 configuration(s)
## INFO  [08:02:18.261] [mlr3] Running benchmark with 10 resampling iterations
## INFO  [08:02:18.266] [mlr3] Applying learner 'regr.ranger' on task 'PredictionSet[1:13]' (iter 1/10)
## INFO  [08:02:18.307] [mlr3] Applying learner 'regr.ranger' on task 'PredictionSet[1:13]' (iter 2/10)
## INFO  [08:02:18.349] [mlr3] Applying learner 'regr.ranger' on task 'PredictionSet[1:13]' (iter 3/10)
## INFO  [08:02:18.389] [mlr3] Applying learner 'regr.ranger' on task 'PredictionSet[1:13]' (iter 4/10)
## INFO  [08:02:18.430] [mlr3] Applying learner 'regr.ranger' on task 'PredictionSet[1:13]' (iter 5/10)
## INFO  [08:02:18.470] [mlr3] Applying learner 'regr.ranger' on task 'PredictionSet[1:13]' (iter 6/10)
## INFO  [08:02:18.511] [mlr3] Applying learner 'regr.ranger' on task 'PredictionSet[1:13]' (iter 7/10)
## INFO  [08:02:18.551] [mlr3] Applying learner 'regr.ranger' on task 'PredictionSet[1:13]' (iter 8/10)
## INFO  [08:02:18.592] [mlr3] Applying learner 'regr.ranger' on task 'PredictionSet[1:13]' (iter 9/10)
## INFO  [08:02:18.633] [mlr3] Applying learner 'regr.ranger' on task 'PredictionSet[1:13]' (iter 10/10)
## INFO  [08:02:18.673] [mlr3] Finished benchmark
## INFO  [08:02:18.704] [bbotk] Result of batch 10:
## INFO  [08:02:18.706] [bbotk]  max.depth num.trees regr.rmse warnings errors runtime_learners
## INFO  [08:02:18.706] [bbotk]         18       369   3.19626        0      0            0.303
## INFO  [08:02:18.706] [bbotk]                                 uhash
## INFO  [08:02:18.706] [bbotk]  ced6dcde-8da3-47b4-80b9-c5ff4f729b2e
## INFO  [08:02:18.711] [bbotk] Finished optimizing after 10 evaluation(s)
## INFO  [08:02:18.712] [bbotk] Result:
## INFO  [08:02:18.713] [bbotk]  max.depth num.trees learner_param_vals  x_domain regr.rmse
## INFO  [08:02:18.713] [bbotk]         18       343          <list[3]> <list[2]>  3.177151
## INFO  [08:02:18.945] [bbotk] Starting to optimize 5 parameter(s) with '<TunerGridSearch>' and '<TerminatorEvals> [n_evals=10, k=0]'
## INFO  [08:02:18.952] [bbotk] Evaluating 1 configuration(s)
## INFO  [08:02:18.968] [mlr3] Running benchmark with 10 resampling iterations
## INFO  [08:02:18.973] [mlr3] Applying learner 'regr.svm' on task 'PredictionSet[1:13]' (iter 1/10)
## INFO  [08:02:18.990] [mlr3] Applying learner 'regr.svm' on task 'PredictionSet[1:13]' (iter 2/10)
## INFO  [08:02:19.008] [mlr3] Applying learner 'regr.svm' on task 'PredictionSet[1:13]' (iter 3/10)
## INFO  [08:02:19.027] [mlr3] Applying learner 'regr.svm' on task 'PredictionSet[1:13]' (iter 4/10)
## INFO  [08:02:19.044] [mlr3] Applying learner 'regr.svm' on task 'PredictionSet[1:13]' (iter 5/10)
## INFO  [08:02:19.062] [mlr3] Applying learner 'regr.svm' on task 'PredictionSet[1:13]' (iter 6/10)
## INFO  [08:02:19.119] [mlr3] Applying learner 'regr.svm' on task 'PredictionSet[1:13]' (iter 7/10)
## INFO  [08:02:19.142] [mlr3] Applying learner 'regr.svm' on task 'PredictionSet[1:13]' (iter 8/10)
## INFO  [08:02:19.162] [mlr3] Applying learner 'regr.svm' on task 'PredictionSet[1:13]' (iter 9/10)
## INFO  [08:02:19.180] [mlr3] Applying learner 'regr.svm' on task 'PredictionSet[1:13]' (iter 10/10)
## INFO  [08:02:19.197] [mlr3] Finished benchmark
## INFO  [08:02:19.226] [bbotk] Result of batch 1:
## INFO  [08:02:19.228] [bbotk]   tolerance    gamma epsilon           type kernel regr.rmse warnings errors
## INFO  [08:02:19.228] [bbotk]  0.06521429 39.28593     0.5 eps-regression radial  4.261889        0      0
## INFO  [08:02:19.228] [bbotk]  runtime_learners                                uhash
## INFO  [08:02:19.228] [bbotk]             0.118 32e141bf-ca3b-4d6c-a23e-cef4abf9e321
## INFO  [08:02:19.229] [bbotk] Evaluating 1 configuration(s)
## INFO  [08:02:19.245] [mlr3] Running benchmark with 10 resampling iterations
## INFO  [08:02:19.250] [mlr3] Applying learner 'regr.svm' on task 'PredictionSet[1:13]' (iter 1/10)
## INFO  [08:02:19.270] [mlr3] Applying learner 'regr.svm' on task 'PredictionSet[1:13]' (iter 2/10)
## INFO  [08:02:19.289] [mlr3] Applying learner 'regr.svm' on task 'PredictionSet[1:13]' (iter 3/10)
## INFO  [08:02:19.311] [mlr3] Applying learner 'regr.svm' on task 'PredictionSet[1:13]' (iter 4/10)
## INFO  [08:02:19.331] [mlr3] Applying learner 'regr.svm' on task 'PredictionSet[1:13]' (iter 5/10)
## INFO  [08:02:19.351] [mlr3] Applying learner 'regr.svm' on task 'PredictionSet[1:13]' (iter 6/10)
## INFO  [08:02:19.370] [mlr3] Applying learner 'regr.svm' on task 'PredictionSet[1:13]' (iter 7/10)
## INFO  [08:02:19.392] [mlr3] Applying learner 'regr.svm' on task 'PredictionSet[1:13]' (iter 8/10)
## INFO  [08:02:19.413] [mlr3] Applying learner 'regr.svm' on task 'PredictionSet[1:13]' (iter 9/10)
## INFO  [08:02:19.433] [mlr3] Applying learner 'regr.svm' on task 'PredictionSet[1:13]' (iter 10/10)
## INFO  [08:02:19.453] [mlr3] Finished benchmark
## INFO  [08:02:19.485] [bbotk] Result of batch 2:
## INFO  [08:02:19.487] [bbotk]  tolerance    gamma   epsilon           type  kernel regr.rmse warnings errors
## INFO  [08:02:19.487] [bbotk]  0.8357857 3.572357 0.5571429 eps-regression sigmoid  174.7835        0      0
## INFO  [08:02:19.487] [bbotk]  runtime_learners                                uhash
## INFO  [08:02:19.487] [bbotk]             0.103 3938f629-5107-4280-bf43-5960cdc3e6f9
## INFO  [08:02:19.488] [bbotk] Evaluating 1 configuration(s)
## INFO  [08:02:19.510] [mlr3] Running benchmark with 10 resampling iterations
## INFO  [08:02:19.517] [mlr3] Applying learner 'regr.svm' on task 'PredictionSet[1:13]' (iter 1/10)
## INFO  [08:02:19.540] [mlr3] Applying learner 'regr.svm' on task 'PredictionSet[1:13]' (iter 2/10)
## INFO  [08:02:19.559] [mlr3] Applying learner 'regr.svm' on task 'PredictionSet[1:13]' (iter 3/10)
## INFO  [08:02:19.577] [mlr3] Applying learner 'regr.svm' on task 'PredictionSet[1:13]' (iter 4/10)
## INFO  [08:02:19.594] [mlr3] Applying learner 'regr.svm' on task 'PredictionSet[1:13]' (iter 5/10)
## INFO  [08:02:19.614] [mlr3] Applying learner 'regr.svm' on task 'PredictionSet[1:13]' (iter 6/10)
## INFO  [08:02:19.632] [mlr3] Applying learner 'regr.svm' on task 'PredictionSet[1:13]' (iter 7/10)
## INFO  [08:02:19.650] [mlr3] Applying learner 'regr.svm' on task 'PredictionSet[1:13]' (iter 8/10)
## INFO  [08:02:19.668] [mlr3] Applying learner 'regr.svm' on task 'PredictionSet[1:13]' (iter 9/10)
## INFO  [08:02:19.687] [mlr3] Applying learner 'regr.svm' on task 'PredictionSet[1:13]' (iter 10/10)
## INFO  [08:02:19.705] [mlr3] Finished benchmark
## INFO  [08:02:19.735] [bbotk] Result of batch 3:
## INFO  [08:02:19.737] [bbotk]  tolerance    gamma epsilon           type  kernel regr.rmse warnings errors
## INFO  [08:02:19.737] [bbotk]     0.4505 42.85729     0.9 eps-regression sigmoid   176.268        0      0
## INFO  [08:02:19.737] [bbotk]  runtime_learners                                uhash
## INFO  [08:02:19.737] [bbotk]             0.087 55421903-c64e-4188-aaf8-7e004cda2899
## INFO  [08:02:19.738] [bbotk] Evaluating 1 configuration(s)
## INFO  [08:02:19.754] [mlr3] Running benchmark with 10 resampling iterations
## INFO  [08:02:19.760] [mlr3] Applying learner 'regr.svm' on task 'PredictionSet[1:13]' (iter 1/10)
## INFO  [08:02:19.777] [mlr3] Applying learner 'regr.svm' on task 'PredictionSet[1:13]' (iter 2/10)
## INFO  [08:02:19.793] [mlr3] Applying learner 'regr.svm' on task 'PredictionSet[1:13]' (iter 3/10)
## INFO  [08:02:19.809] [mlr3] Applying learner 'regr.svm' on task 'PredictionSet[1:13]' (iter 4/10)
## INFO  [08:02:19.826] [mlr3] Applying learner 'regr.svm' on task 'PredictionSet[1:13]' (iter 5/10)
## INFO  [08:02:19.843] [mlr3] Applying learner 'regr.svm' on task 'PredictionSet[1:13]' (iter 6/10)
## INFO  [08:02:19.860] [mlr3] Applying learner 'regr.svm' on task 'PredictionSet[1:13]' (iter 7/10)
## INFO  [08:02:19.876] [mlr3] Applying learner 'regr.svm' on task 'PredictionSet[1:13]' (iter 8/10)
## INFO  [08:02:19.899] [mlr3] Applying learner 'regr.svm' on task 'PredictionSet[1:13]' (iter 9/10)
## INFO  [08:02:19.920] [mlr3] Applying learner 'regr.svm' on task 'PredictionSet[1:13]' (iter 10/10)
## INFO  [08:02:19.938] [mlr3] Finished benchmark
## INFO  [08:02:19.969] [bbotk] Result of batch 4:
## INFO  [08:02:19.970] [bbotk]  tolerance    gamma   epsilon           type kernel regr.rmse warnings errors
## INFO  [08:02:19.970] [bbotk]  0.1936429 10.71507 0.4428571 eps-regression radial  3.885715        0      0
## INFO  [08:02:19.970] [bbotk]  runtime_learners                                uhash
## INFO  [08:02:19.970] [bbotk]             0.068 fa818c56-73d4-43f6-b559-c00462bb7180
## INFO  [08:02:19.972] [bbotk] Evaluating 1 configuration(s)
## INFO  [08:02:19.988] [mlr3] Running benchmark with 10 resampling iterations
## INFO  [08:02:19.993] [mlr3] Applying learner 'regr.svm' on task 'PredictionSet[1:13]' (iter 1/10)
## INFO  [08:02:20.012] [mlr3] Applying learner 'regr.svm' on task 'PredictionSet[1:13]' (iter 2/10)
## INFO  [08:02:20.029] [mlr3] Applying learner 'regr.svm' on task 'PredictionSet[1:13]' (iter 3/10)
## INFO  [08:02:20.047] [mlr3] Applying learner 'regr.svm' on task 'PredictionSet[1:13]' (iter 4/10)
## INFO  [08:02:20.067] [mlr3] Applying learner 'regr.svm' on task 'PredictionSet[1:13]' (iter 5/10)
## INFO  [08:02:20.085] [mlr3] Applying learner 'regr.svm' on task 'PredictionSet[1:13]' (iter 6/10)
## INFO  [08:02:20.103] [mlr3] Applying learner 'regr.svm' on task 'PredictionSet[1:13]' (iter 7/10)
## INFO  [08:02:20.120] [mlr3] Applying learner 'regr.svm' on task 'PredictionSet[1:13]' (iter 8/10)
## INFO  [08:02:20.141] [mlr3] Applying learner 'regr.svm' on task 'PredictionSet[1:13]' (iter 9/10)
## INFO  [08:02:20.159] [mlr3] Applying learner 'regr.svm' on task 'PredictionSet[1:13]' (iter 10/10)
## INFO  [08:02:20.176] [mlr3] Finished benchmark
## INFO  [08:02:20.208] [bbotk] Result of batch 5:
## INFO  [08:02:20.210] [bbotk]  tolerance    gamma   epsilon           type  kernel regr.rmse warnings errors
## INFO  [08:02:20.210] [bbotk]  0.2578571 32.14321 0.1571429 eps-regression sigmoid  177.4522        0      0
## INFO  [08:02:20.210] [bbotk]  runtime_learners                                uhash
## INFO  [08:02:20.210] [bbotk]             0.083 607ff1ff-f66f-44a5-9438-06abe746676e
## INFO  [08:02:20.212] [bbotk] Evaluating 1 configuration(s)
## INFO  [08:02:20.228] [mlr3] Running benchmark with 10 resampling iterations
## INFO  [08:02:20.233] [mlr3] Applying learner 'regr.svm' on task 'PredictionSet[1:13]' (iter 1/10)
## INFO  [08:02:20.250] [mlr3] Applying learner 'regr.svm' on task 'PredictionSet[1:13]' (iter 2/10)
## INFO  [08:02:20.276] [mlr3] Applying learner 'regr.svm' on task 'PredictionSet[1:13]' (iter 3/10)
## INFO  [08:02:20.299] [mlr3] Applying learner 'regr.svm' on task 'PredictionSet[1:13]' (iter 4/10)
## INFO  [08:02:20.319] [mlr3] Applying learner 'regr.svm' on task 'PredictionSet[1:13]' (iter 5/10)
## INFO  [08:02:20.337] [mlr3] Applying learner 'regr.svm' on task 'PredictionSet[1:13]' (iter 6/10)
## INFO  [08:02:20.355] [mlr3] Applying learner 'regr.svm' on task 'PredictionSet[1:13]' (iter 7/10)
## INFO  [08:02:20.375] [mlr3] Applying learner 'regr.svm' on task 'PredictionSet[1:13]' (iter 8/10)
## INFO  [08:02:20.392] [mlr3] Applying learner 'regr.svm' on task 'PredictionSet[1:13]' (iter 9/10)
## INFO  [08:02:20.410] [mlr3] Applying learner 'regr.svm' on task 'PredictionSet[1:13]' (iter 10/10)
## INFO  [08:02:20.428] [mlr3] Finished benchmark
## INFO  [08:02:20.460] [bbotk] Result of batch 6:
## INFO  [08:02:20.461] [bbotk]  tolerance    gamma epsilon           type  kernel regr.rmse warnings errors
## INFO  [08:02:20.461] [bbotk]  0.3862857 39.28593     0.1 eps-regression sigmoid  177.4129        0      0
## INFO  [08:02:20.461] [bbotk]  runtime_learners                                uhash
## INFO  [08:02:20.461] [bbotk]             0.093 1a3ec845-0ad5-440d-8228-7546b2527be0
## INFO  [08:02:20.463] [bbotk] Evaluating 1 configuration(s)
## INFO  [08:02:20.478] [mlr3] Running benchmark with 10 resampling iterations
## INFO  [08:02:20.483] [mlr3] Applying learner 'regr.svm' on task 'PredictionSet[1:13]' (iter 1/10)
## INFO  [08:02:20.499] [mlr3] Applying learner 'regr.svm' on task 'PredictionSet[1:13]' (iter 2/10)
## INFO  [08:02:20.517] [mlr3] Applying learner 'regr.svm' on task 'PredictionSet[1:13]' (iter 3/10)
## INFO  [08:02:20.534] [mlr3] Applying learner 'regr.svm' on task 'PredictionSet[1:13]' (iter 4/10)
## INFO  [08:02:20.550] [mlr3] Applying learner 'regr.svm' on task 'PredictionSet[1:13]' (iter 5/10)
## INFO  [08:02:20.566] [mlr3] Applying learner 'regr.svm' on task 'PredictionSet[1:13]' (iter 6/10)
## INFO  [08:02:20.583] [mlr3] Applying learner 'regr.svm' on task 'PredictionSet[1:13]' (iter 7/10)
## INFO  [08:02:20.600] [mlr3] Applying learner 'regr.svm' on task 'PredictionSet[1:13]' (iter 8/10)
## INFO  [08:02:20.617] [mlr3] Applying learner 'regr.svm' on task 'PredictionSet[1:13]' (iter 9/10)
## INFO  [08:02:20.633] [mlr3] Applying learner 'regr.svm' on task 'PredictionSet[1:13]' (iter 10/10)
## INFO  [08:02:20.658] [mlr3] Finished benchmark
## INFO  [08:02:20.694] [bbotk] Result of batch 7:
## INFO  [08:02:20.696] [bbotk]  tolerance    gamma   epsilon           type kernel regr.rmse warnings errors
## INFO  [08:02:20.696] [bbotk]  0.5147143 39.28593 0.3857143 eps-regression radial  4.229401        0      0
## INFO  [08:02:20.696] [bbotk]  runtime_learners                                uhash
## INFO  [08:02:20.696] [bbotk]             0.071 039ca986-9edc-41a2-a697-486c08efbddd
## INFO  [08:02:20.697] [bbotk] Evaluating 1 configuration(s)
## INFO  [08:02:20.713] [mlr3] Running benchmark with 10 resampling iterations
## INFO  [08:02:20.718] [mlr3] Applying learner 'regr.svm' on task 'PredictionSet[1:13]' (iter 1/10)
## INFO  [08:02:20.734] [mlr3] Applying learner 'regr.svm' on task 'PredictionSet[1:13]' (iter 2/10)
## INFO  [08:02:20.750] [mlr3] Applying learner 'regr.svm' on task 'PredictionSet[1:13]' (iter 3/10)
## INFO  [08:02:20.765] [mlr3] Applying learner 'regr.svm' on task 'PredictionSet[1:13]' (iter 4/10)
## INFO  [08:02:20.780] [mlr3] Applying learner 'regr.svm' on task 'PredictionSet[1:13]' (iter 5/10)
## INFO  [08:02:20.794] [mlr3] Applying learner 'regr.svm' on task 'PredictionSet[1:13]' (iter 6/10)
## INFO  [08:02:20.811] [mlr3] Applying learner 'regr.svm' on task 'PredictionSet[1:13]' (iter 7/10)
## INFO  [08:02:20.827] [mlr3] Applying learner 'regr.svm' on task 'PredictionSet[1:13]' (iter 8/10)
## INFO  [08:02:20.842] [mlr3] Applying learner 'regr.svm' on task 'PredictionSet[1:13]' (iter 9/10)
## INFO  [08:02:20.857] [mlr3] Applying learner 'regr.svm' on task 'PredictionSet[1:13]' (iter 10/10)
## INFO  [08:02:20.871] [mlr3] Finished benchmark
## INFO  [08:02:20.905] [bbotk] Result of batch 8:
## INFO  [08:02:20.906] [bbotk]  tolerance    gamma   epsilon           type kernel regr.rmse warnings errors
## INFO  [08:02:20.906] [bbotk]  0.7073571 17.85779 0.7857143 eps-regression radial  5.328353        0      0
## INFO  [08:02:20.906] [bbotk]  runtime_learners                                uhash
## INFO  [08:02:20.906] [bbotk]             0.054 da8728da-9084-43da-a96e-d41b5f8f8b36
## INFO  [08:02:20.908] [bbotk] Evaluating 1 configuration(s)
## INFO  [08:02:20.923] [mlr3] Running benchmark with 10 resampling iterations
## INFO  [08:02:20.928] [mlr3] Applying learner 'regr.svm' on task 'PredictionSet[1:13]' (iter 1/10)
## INFO  [08:02:20.948] [mlr3] Applying learner 'regr.svm' on task 'PredictionSet[1:13]' (iter 2/10)
## INFO  [08:02:20.969] [mlr3] Applying learner 'regr.svm' on task 'PredictionSet[1:13]' (iter 3/10)
## INFO  [08:02:20.989] [mlr3] Applying learner 'regr.svm' on task 'PredictionSet[1:13]' (iter 4/10)
## INFO  [08:02:21.018] [mlr3] Applying learner 'regr.svm' on task 'PredictionSet[1:13]' (iter 5/10)
## INFO  [08:02:21.043] [mlr3] Applying learner 'regr.svm' on task 'PredictionSet[1:13]' (iter 6/10)
## INFO  [08:02:21.064] [mlr3] Applying learner 'regr.svm' on task 'PredictionSet[1:13]' (iter 7/10)
## INFO  [08:02:21.084] [mlr3] Applying learner 'regr.svm' on task 'PredictionSet[1:13]' (iter 8/10)
## INFO  [08:02:21.105] [mlr3] Applying learner 'regr.svm' on task 'PredictionSet[1:13]' (iter 9/10)
## INFO  [08:02:21.126] [mlr3] Applying learner 'regr.svm' on task 'PredictionSet[1:13]' (iter 10/10)
## INFO  [08:02:21.146] [mlr3] Finished benchmark
## INFO  [08:02:21.177] [bbotk] Result of batch 9:
## INFO  [08:02:21.178] [bbotk]  tolerance    gamma   epsilon           type  kernel regr.rmse warnings errors
## INFO  [08:02:21.178] [bbotk]  0.5147143 3.572357 0.3857143 eps-regression sigmoid  174.7064        0      0
## INFO  [08:02:21.178] [bbotk]  runtime_learners                                uhash
## INFO  [08:02:21.178] [bbotk]             0.105 c993beaf-9237-4339-8cba-9861250f1c8e
## INFO  [08:02:21.180] [bbotk] Evaluating 1 configuration(s)
## INFO  [08:02:21.196] [mlr3] Running benchmark with 10 resampling iterations
## INFO  [08:02:21.201] [mlr3] Applying learner 'regr.svm' on task 'PredictionSet[1:13]' (iter 1/10)
## INFO  [08:02:21.217] [mlr3] Applying learner 'regr.svm' on task 'PredictionSet[1:13]' (iter 2/10)
## INFO  [08:02:21.232] [mlr3] Applying learner 'regr.svm' on task 'PredictionSet[1:13]' (iter 3/10)
## INFO  [08:02:21.247] [mlr3] Applying learner 'regr.svm' on task 'PredictionSet[1:13]' (iter 4/10)
## INFO  [08:02:21.265] [mlr3] Applying learner 'regr.svm' on task 'PredictionSet[1:13]' (iter 5/10)
## INFO  [08:02:21.281] [mlr3] Applying learner 'regr.svm' on task 'PredictionSet[1:13]' (iter 6/10)
## INFO  [08:02:21.296] [mlr3] Applying learner 'regr.svm' on task 'PredictionSet[1:13]' (iter 7/10)
## INFO  [08:02:21.312] [mlr3] Applying learner 'regr.svm' on task 'PredictionSet[1:13]' (iter 8/10)
## INFO  [08:02:21.327] [mlr3] Applying learner 'regr.svm' on task 'PredictionSet[1:13]' (iter 9/10)
## INFO  [08:02:21.344] [mlr3] Applying learner 'regr.svm' on task 'PredictionSet[1:13]' (iter 10/10)
## INFO  [08:02:21.359] [mlr3] Finished benchmark
## INFO  [08:02:21.402] [bbotk] Result of batch 10:
## INFO  [08:02:21.404] [bbotk]  tolerance    gamma epsilon           type kernel regr.rmse warnings errors
## INFO  [08:02:21.404] [bbotk]  0.2578571 21.42914     0.9 eps-regression radial   5.15375        0      0
## INFO  [08:02:21.404] [bbotk]  runtime_learners                                uhash
## INFO  [08:02:21.404] [bbotk]             0.057 54cf7a92-140d-472d-9411-b4aad0df4a21
## INFO  [08:02:21.410] [bbotk] Finished optimizing after 10 evaluation(s)
## INFO  [08:02:21.411] [bbotk] Result:
## INFO  [08:02:21.413] [bbotk]  tolerance    gamma   epsilon           type kernel learner_param_vals
## INFO  [08:02:21.413] [bbotk]  0.1936429 10.71507 0.4428571 eps-regression radial          <list[5]>
## INFO  [08:02:21.413] [bbotk]   x_domain regr.rmse
## INFO  [08:02:21.413] [bbotk]  <list[5]>  3.885715
## INFO  [08:02:21.550] [bbotk] Starting to optimize 3 parameter(s) with '<TunerGridSearch>' and '<TerminatorEvals> [n_evals=10, k=0]'
## INFO  [08:02:21.554] [bbotk] Evaluating 1 configuration(s)
## INFO  [08:02:21.566] [mlr3] Running benchmark with 10 resampling iterations
## INFO  [08:02:21.571] [mlr3] Applying learner 'regr.kknn' on task 'PredictionSet[1:13]' (iter 1/10)
## INFO  [08:02:21.591] [mlr3] Applying learner 'regr.kknn' on task 'PredictionSet[1:13]' (iter 2/10)
## INFO  [08:02:21.611] [mlr3] Applying learner 'regr.kknn' on task 'PredictionSet[1:13]' (iter 3/10)
## INFO  [08:02:21.632] [mlr3] Applying learner 'regr.kknn' on task 'PredictionSet[1:13]' (iter 4/10)
## INFO  [08:02:21.653] [mlr3] Applying learner 'regr.kknn' on task 'PredictionSet[1:13]' (iter 5/10)
## INFO  [08:02:21.673] [mlr3] Applying learner 'regr.kknn' on task 'PredictionSet[1:13]' (iter 6/10)
## INFO  [08:02:21.701] [mlr3] Applying learner 'regr.kknn' on task 'PredictionSet[1:13]' (iter 7/10)
## INFO  [08:02:21.727] [mlr3] Applying learner 'regr.kknn' on task 'PredictionSet[1:13]' (iter 8/10)
## INFO  [08:02:21.749] [mlr3] Applying learner 'regr.kknn' on task 'PredictionSet[1:13]' (iter 9/10)
## INFO  [08:02:21.769] [mlr3] Applying learner 'regr.kknn' on task 'PredictionSet[1:13]' (iter 10/10)
## INFO  [08:02:21.790] [mlr3] Finished benchmark
## INFO  [08:02:21.819] [bbotk] Result of batch 1:
## INFO  [08:02:21.821] [bbotk]   k distance     kernel regr.rmse warnings errors runtime_learners
## INFO  [08:02:21.821] [bbotk]  13 1.521429 triangular  3.607803        0      0            0.108
## INFO  [08:02:21.821] [bbotk]                                 uhash
## INFO  [08:02:21.821] [bbotk]  18dec424-347b-43bc-93e3-0ccf486a75ee
## INFO  [08:02:21.822] [bbotk] Evaluating 1 configuration(s)
## INFO  [08:02:21.832] [mlr3] Running benchmark with 10 resampling iterations
## INFO  [08:02:21.837] [mlr3] Applying learner 'regr.kknn' on task 'PredictionSet[1:13]' (iter 1/10)
## INFO  [08:02:21.858] [mlr3] Applying learner 'regr.kknn' on task 'PredictionSet[1:13]' (iter 2/10)
## INFO  [08:02:21.879] [mlr3] Applying learner 'regr.kknn' on task 'PredictionSet[1:13]' (iter 3/10)
## INFO  [08:02:21.899] [mlr3] Applying learner 'regr.kknn' on task 'PredictionSet[1:13]' (iter 4/10)
## INFO  [08:02:21.919] [mlr3] Applying learner 'regr.kknn' on task 'PredictionSet[1:13]' (iter 5/10)
## INFO  [08:02:21.941] [mlr3] Applying learner 'regr.kknn' on task 'PredictionSet[1:13]' (iter 6/10)
## INFO  [08:02:21.964] [mlr3] Applying learner 'regr.kknn' on task 'PredictionSet[1:13]' (iter 7/10)
## INFO  [08:02:21.984] [mlr3] Applying learner 'regr.kknn' on task 'PredictionSet[1:13]' (iter 8/10)
## INFO  [08:02:22.004] [mlr3] Applying learner 'regr.kknn' on task 'PredictionSet[1:13]' (iter 9/10)
## INFO  [08:02:22.026] [mlr3] Applying learner 'regr.kknn' on task 'PredictionSet[1:13]' (iter 10/10)
## INFO  [08:02:22.046] [mlr3] Finished benchmark
## INFO  [08:02:22.087] [bbotk] Result of batch 2:
## INFO  [08:02:22.089] [bbotk]  k distance kernel regr.rmse warnings errors runtime_learners
## INFO  [08:02:22.089] [bbotk]  9 15.73571    cos  3.911422        0      0              0.1
## INFO  [08:02:22.089] [bbotk]                                 uhash
## INFO  [08:02:22.089] [bbotk]  8eabc3e8-a0fc-4571-9ef8-1b466e3b4776
## INFO  [08:02:22.091] [bbotk] Evaluating 1 configuration(s)
## INFO  [08:02:22.103] [mlr3] Running benchmark with 10 resampling iterations
## INFO  [08:02:22.109] [mlr3] Applying learner 'regr.kknn' on task 'PredictionSet[1:13]' (iter 1/10)
## INFO  [08:02:22.129] [mlr3] Applying learner 'regr.kknn' on task 'PredictionSet[1:13]' (iter 2/10)
## INFO  [08:02:22.150] [mlr3] Applying learner 'regr.kknn' on task 'PredictionSet[1:13]' (iter 3/10)
## INFO  [08:02:22.172] [mlr3] Applying learner 'regr.kknn' on task 'PredictionSet[1:13]' (iter 4/10)
## INFO  [08:02:22.193] [mlr3] Applying learner 'regr.kknn' on task 'PredictionSet[1:13]' (iter 5/10)
## INFO  [08:02:22.213] [mlr3] Applying learner 'regr.kknn' on task 'PredictionSet[1:13]' (iter 6/10)
## INFO  [08:02:22.234] [mlr3] Applying learner 'regr.kknn' on task 'PredictionSet[1:13]' (iter 7/10)
## INFO  [08:02:22.255] [mlr3] Applying learner 'regr.kknn' on task 'PredictionSet[1:13]' (iter 8/10)
## INFO  [08:02:22.275] [mlr3] Applying learner 'regr.kknn' on task 'PredictionSet[1:13]' (iter 9/10)
## INFO  [08:02:22.295] [mlr3] Applying learner 'regr.kknn' on task 'PredictionSet[1:13]' (iter 10/10)
## INFO  [08:02:22.317] [mlr3] Finished benchmark
## INFO  [08:02:22.352] [bbotk] Result of batch 3:
## INFO  [08:02:22.353] [bbotk]   k distance kernel regr.rmse warnings errors runtime_learners
## INFO  [08:02:22.353] [bbotk]  16       20    cos   3.87573        0      0            0.107
## INFO  [08:02:22.353] [bbotk]                                 uhash
## INFO  [08:02:22.353] [bbotk]  8acd559c-cae2-4297-a3fc-e49a1fd70293
## INFO  [08:02:22.355] [bbotk] Evaluating 1 configuration(s)
## INFO  [08:02:22.365] [mlr3] Running benchmark with 10 resampling iterations
## INFO  [08:02:22.370] [mlr3] Applying learner 'regr.kknn' on task 'PredictionSet[1:13]' (iter 1/10)
## INFO  [08:02:22.392] [mlr3] Applying learner 'regr.kknn' on task 'PredictionSet[1:13]' (iter 2/10)
## INFO  [08:02:22.412] [mlr3] Applying learner 'regr.kknn' on task 'PredictionSet[1:13]' (iter 3/10)
## INFO  [08:02:22.433] [mlr3] Applying learner 'regr.kknn' on task 'PredictionSet[1:13]' (iter 4/10)
## INFO  [08:02:22.463] [mlr3] Applying learner 'regr.kknn' on task 'PredictionSet[1:13]' (iter 5/10)
## INFO  [08:02:22.487] [mlr3] Applying learner 'regr.kknn' on task 'PredictionSet[1:13]' (iter 6/10)
## INFO  [08:02:22.508] [mlr3] Applying learner 'regr.kknn' on task 'PredictionSet[1:13]' (iter 7/10)
## INFO  [08:02:22.528] [mlr3] Applying learner 'regr.kknn' on task 'PredictionSet[1:13]' (iter 8/10)
## INFO  [08:02:22.550] [mlr3] Applying learner 'regr.kknn' on task 'PredictionSet[1:13]' (iter 9/10)
## INFO  [08:02:22.570] [mlr3] Applying learner 'regr.kknn' on task 'PredictionSet[1:13]' (iter 10/10)
## INFO  [08:02:22.590] [mlr3] Finished benchmark
## INFO  [08:02:22.623] [bbotk] Result of batch 4:
## INFO  [08:02:22.624] [bbotk]   k distance  kernel regr.rmse warnings errors runtime_learners
## INFO  [08:02:22.624] [bbotk]  10 4.364286 optimal   3.46317        0      0            0.114
## INFO  [08:02:22.624] [bbotk]                                 uhash
## INFO  [08:02:22.624] [bbotk]  90ebf032-5240-41e6-abd8-00c7f33358bc
## INFO  [08:02:22.625] [bbotk] Evaluating 1 configuration(s)
## INFO  [08:02:22.636] [mlr3] Running benchmark with 10 resampling iterations
## INFO  [08:02:22.641] [mlr3] Applying learner 'regr.kknn' on task 'PredictionSet[1:13]' (iter 1/10)
## INFO  [08:02:22.660] [mlr3] Applying learner 'regr.kknn' on task 'PredictionSet[1:13]' (iter 2/10)
## INFO  [08:02:22.681] [mlr3] Applying learner 'regr.kknn' on task 'PredictionSet[1:13]' (iter 3/10)
## INFO  [08:02:22.703] [mlr3] Applying learner 'regr.kknn' on task 'PredictionSet[1:13]' (iter 4/10)
## INFO  [08:02:22.724] [mlr3] Applying learner 'regr.kknn' on task 'PredictionSet[1:13]' (iter 5/10)
## INFO  [08:02:22.744] [mlr3] Applying learner 'regr.kknn' on task 'PredictionSet[1:13]' (iter 6/10)
## INFO  [08:02:22.766] [mlr3] Applying learner 'regr.kknn' on task 'PredictionSet[1:13]' (iter 7/10)
## INFO  [08:02:22.786] [mlr3] Applying learner 'regr.kknn' on task 'PredictionSet[1:13]' (iter 8/10)
## INFO  [08:02:22.807] [mlr3] Applying learner 'regr.kknn' on task 'PredictionSet[1:13]' (iter 9/10)
## INFO  [08:02:22.836] [mlr3] Applying learner 'regr.kknn' on task 'PredictionSet[1:13]' (iter 10/10)
## INFO  [08:02:22.860] [mlr3] Finished benchmark
## INFO  [08:02:22.892] [bbotk] Result of batch 5:
## INFO  [08:02:22.893] [bbotk]   k distance  kernel regr.rmse warnings errors runtime_learners
## INFO  [08:02:22.893] [bbotk]  19 1.521429 optimal  3.575456        0      0            0.113
## INFO  [08:02:22.893] [bbotk]                                 uhash
## INFO  [08:02:22.893] [bbotk]  88d3101b-9011-4edd-983a-ef62387bb431
## INFO  [08:02:22.895] [bbotk] Evaluating 1 configuration(s)
## INFO  [08:02:22.906] [mlr3] Running benchmark with 10 resampling iterations
## INFO  [08:02:22.911] [mlr3] Applying learner 'regr.kknn' on task 'PredictionSet[1:13]' (iter 1/10)
## INFO  [08:02:22.932] [mlr3] Applying learner 'regr.kknn' on task 'PredictionSet[1:13]' (iter 2/10)
## INFO  [08:02:22.952] [mlr3] Applying learner 'regr.kknn' on task 'PredictionSet[1:13]' (iter 3/10)
## INFO  [08:02:22.972] [mlr3] Applying learner 'regr.kknn' on task 'PredictionSet[1:13]' (iter 4/10)
## INFO  [08:02:22.993] [mlr3] Applying learner 'regr.kknn' on task 'PredictionSet[1:13]' (iter 5/10)
## INFO  [08:02:23.014] [mlr3] Applying learner 'regr.kknn' on task 'PredictionSet[1:13]' (iter 6/10)
## INFO  [08:02:23.034] [mlr3] Applying learner 'regr.kknn' on task 'PredictionSet[1:13]' (iter 7/10)
## INFO  [08:02:23.054] [mlr3] Applying learner 'regr.kknn' on task 'PredictionSet[1:13]' (iter 8/10)
## INFO  [08:02:23.076] [mlr3] Applying learner 'regr.kknn' on task 'PredictionSet[1:13]' (iter 9/10)
## INFO  [08:02:23.096] [mlr3] Applying learner 'regr.kknn' on task 'PredictionSet[1:13]' (iter 10/10)
## INFO  [08:02:23.116] [mlr3] Finished benchmark
## INFO  [08:02:23.149] [bbotk] Result of batch 6:
## INFO  [08:02:23.151] [bbotk]  k distance kernel regr.rmse warnings errors runtime_learners
## INFO  [08:02:23.151] [bbotk]  3    10.05    cos  3.535843        0      0            0.098
## INFO  [08:02:23.151] [bbotk]                                 uhash
## INFO  [08:02:23.151] [bbotk]  660e9f21-7aeb-41db-b7d5-1c2b38d96717
## INFO  [08:02:23.152] [bbotk] Evaluating 1 configuration(s)
## INFO  [08:02:23.162] [mlr3] Running benchmark with 10 resampling iterations
## INFO  [08:02:23.167] [mlr3] Applying learner 'regr.kknn' on task 'PredictionSet[1:13]' (iter 1/10)
## INFO  [08:02:23.196] [mlr3] Applying learner 'regr.kknn' on task 'PredictionSet[1:13]' (iter 2/10)
## INFO  [08:02:23.222] [mlr3] Applying learner 'regr.kknn' on task 'PredictionSet[1:13]' (iter 3/10)
## INFO  [08:02:23.243] [mlr3] Applying learner 'regr.kknn' on task 'PredictionSet[1:13]' (iter 4/10)
## INFO  [08:02:23.264] [mlr3] Applying learner 'regr.kknn' on task 'PredictionSet[1:13]' (iter 5/10)
## INFO  [08:02:23.285] [mlr3] Applying learner 'regr.kknn' on task 'PredictionSet[1:13]' (iter 6/10)
## INFO  [08:02:23.306] [mlr3] Applying learner 'regr.kknn' on task 'PredictionSet[1:13]' (iter 7/10)
## INFO  [08:02:23.326] [mlr3] Applying learner 'regr.kknn' on task 'PredictionSet[1:13]' (iter 8/10)
## INFO  [08:02:23.346] [mlr3] Applying learner 'regr.kknn' on task 'PredictionSet[1:13]' (iter 9/10)
## INFO  [08:02:23.368] [mlr3] Applying learner 'regr.kknn' on task 'PredictionSet[1:13]' (iter 10/10)
## INFO  [08:02:23.388] [mlr3] Finished benchmark
## INFO  [08:02:23.419] [bbotk] Result of batch 7:
## INFO  [08:02:23.420] [bbotk]   k distance kernel regr.rmse warnings errors runtime_learners
## INFO  [08:02:23.420] [bbotk]  13      0.1    inv  2.158055        0      0            0.116
## INFO  [08:02:23.420] [bbotk]                                 uhash
## INFO  [08:02:23.420] [bbotk]  cf2009bf-8d8d-460c-a40c-7b01f1aa8713
## INFO  [08:02:23.422] [bbotk] Evaluating 1 configuration(s)
## INFO  [08:02:23.434] [mlr3] Running benchmark with 10 resampling iterations
## INFO  [08:02:23.439] [mlr3] Applying learner 'regr.kknn' on task 'PredictionSet[1:13]' (iter 1/10)
## INFO  [08:02:23.460] [mlr3] Applying learner 'regr.kknn' on task 'PredictionSet[1:13]' (iter 2/10)
## INFO  [08:02:23.480] [mlr3] Applying learner 'regr.kknn' on task 'PredictionSet[1:13]' (iter 3/10)
## INFO  [08:02:23.500] [mlr3] Applying learner 'regr.kknn' on task 'PredictionSet[1:13]' (iter 4/10)
## INFO  [08:02:23.523] [mlr3] Applying learner 'regr.kknn' on task 'PredictionSet[1:13]' (iter 5/10)
## INFO  [08:02:23.543] [mlr3] Applying learner 'regr.kknn' on task 'PredictionSet[1:13]' (iter 6/10)
## INFO  [08:02:23.572] [mlr3] Applying learner 'regr.kknn' on task 'PredictionSet[1:13]' (iter 7/10)
## INFO  [08:02:23.598] [mlr3] Applying learner 'regr.kknn' on task 'PredictionSet[1:13]' (iter 8/10)
## INFO  [08:02:23.619] [mlr3] Applying learner 'regr.kknn' on task 'PredictionSet[1:13]' (iter 9/10)
## INFO  [08:02:23.639] [mlr3] Applying learner 'regr.kknn' on task 'PredictionSet[1:13]' (iter 10/10)
## INFO  [08:02:23.661] [mlr3] Finished benchmark
## INFO  [08:02:23.692] [bbotk] Result of batch 8:
## INFO  [08:02:23.694] [bbotk]  k distance kernel regr.rmse warnings errors runtime_learners
## INFO  [08:02:23.694] [bbotk]  5 5.785714    inv  2.705185        0      0            0.116
## INFO  [08:02:23.694] [bbotk]                                 uhash
## INFO  [08:02:23.694] [bbotk]  3948172e-51b6-412b-954b-9404b7e74208
## INFO  [08:02:23.695] [bbotk] Evaluating 1 configuration(s)
## INFO  [08:02:23.705] [mlr3] Running benchmark with 10 resampling iterations
## INFO  [08:02:23.710] [mlr3] Applying learner 'regr.kknn' on task 'PredictionSet[1:13]' (iter 1/10)
## INFO  [08:02:23.731] [mlr3] Applying learner 'regr.kknn' on task 'PredictionSet[1:13]' (iter 2/10)
## INFO  [08:02:23.752] [mlr3] Applying learner 'regr.kknn' on task 'PredictionSet[1:13]' (iter 3/10)
## INFO  [08:02:23.773] [mlr3] Applying learner 'regr.kknn' on task 'PredictionSet[1:13]' (iter 4/10)
## INFO  [08:02:23.793] [mlr3] Applying learner 'regr.kknn' on task 'PredictionSet[1:13]' (iter 5/10)
## INFO  [08:02:23.815] [mlr3] Applying learner 'regr.kknn' on task 'PredictionSet[1:13]' (iter 6/10)
## INFO  [08:02:23.836] [mlr3] Applying learner 'regr.kknn' on task 'PredictionSet[1:13]' (iter 7/10)
## INFO  [08:02:23.856] [mlr3] Applying learner 'regr.kknn' on task 'PredictionSet[1:13]' (iter 8/10)
## INFO  [08:02:23.877] [mlr3] Applying learner 'regr.kknn' on task 'PredictionSet[1:13]' (iter 9/10)
## INFO  [08:02:23.899] [mlr3] Applying learner 'regr.kknn' on task 'PredictionSet[1:13]' (iter 10/10)
## INFO  [08:02:23.919] [mlr3] Finished benchmark
## INFO  [08:02:23.963] [bbotk] Result of batch 9:
## INFO  [08:02:23.965] [bbotk]   k distance       kernel regr.rmse warnings errors runtime_learners
## INFO  [08:02:23.965] [bbotk]  20 11.47143 epanechnikov   3.86951        0      0            0.103
## INFO  [08:02:23.965] [bbotk]                                 uhash
## INFO  [08:02:23.965] [bbotk]  eb54e541-b351-48de-985c-3caa2539bfe9
## INFO  [08:02:23.967] [bbotk] Evaluating 1 configuration(s)
## INFO  [08:02:23.977] [mlr3] Running benchmark with 10 resampling iterations
## INFO  [08:02:23.983] [mlr3] Applying learner 'regr.kknn' on task 'PredictionSet[1:13]' (iter 1/10)
## INFO  [08:02:24.003] [mlr3] Applying learner 'regr.kknn' on task 'PredictionSet[1:13]' (iter 2/10)
## INFO  [08:02:24.023] [mlr3] Applying learner 'regr.kknn' on task 'PredictionSet[1:13]' (iter 3/10)
## INFO  [08:02:24.045] [mlr3] Applying learner 'regr.kknn' on task 'PredictionSet[1:13]' (iter 4/10)
## INFO  [08:02:24.066] [mlr3] Applying learner 'regr.kknn' on task 'PredictionSet[1:13]' (iter 5/10)
## INFO  [08:02:24.086] [mlr3] Applying learner 'regr.kknn' on task 'PredictionSet[1:13]' (iter 6/10)
## INFO  [08:02:24.107] [mlr3] Applying learner 'regr.kknn' on task 'PredictionSet[1:13]' (iter 7/10)
## INFO  [08:02:24.129] [mlr3] Applying learner 'regr.kknn' on task 'PredictionSet[1:13]' (iter 8/10)
## INFO  [08:02:24.149] [mlr3] Applying learner 'regr.kknn' on task 'PredictionSet[1:13]' (iter 9/10)
## INFO  [08:02:24.169] [mlr3] Applying learner 'regr.kknn' on task 'PredictionSet[1:13]' (iter 10/10)
## INFO  [08:02:24.191] [mlr3] Finished benchmark
## INFO  [08:02:24.222] [bbotk] Result of batch 10:
## INFO  [08:02:24.224] [bbotk]   k distance       kernel regr.rmse warnings errors runtime_learners
## INFO  [08:02:24.224] [bbotk]  20 2.942857 epanechnikov  3.829641        0      0            0.101
## INFO  [08:02:24.224] [bbotk]                                 uhash
## INFO  [08:02:24.224] [bbotk]  590b97d9-9823-4af5-895a-320762c0eacf
## INFO  [08:02:24.229] [bbotk] Finished optimizing after 10 evaluation(s)
## INFO  [08:02:24.229] [bbotk] Result:
## INFO  [08:02:24.230] [bbotk]   k distance kernel learner_param_vals  x_domain regr.rmse
## INFO  [08:02:24.230] [bbotk]  13      0.1    inv          <list[3]> <list[3]>  2.158055
## INFO  [08:02:24.451] [bbotk] Starting to optimize 2 parameter(s) with '<TunerGridSearch>' and '<TerminatorEvals> [n_evals=10, k=0]'
## INFO  [08:02:24.453] [bbotk] Evaluating 1 configuration(s)
## INFO  [08:02:24.475] [mlr3] Running benchmark with 10 resampling iterations
## INFO  [08:02:24.481] [mlr3] Applying learner 'regr.ranger' on task 'PredictionSet[1:13]' (iter 1/10)
## INFO  [08:02:24.514] [mlr3] Applying learner 'regr.ranger' on task 'PredictionSet[1:13]' (iter 2/10)
## INFO  [08:02:24.545] [mlr3] Applying learner 'regr.ranger' on task 'PredictionSet[1:13]' (iter 3/10)
## INFO  [08:02:24.577] [mlr3] Applying learner 'regr.ranger' on task 'PredictionSet[1:13]' (iter 4/10)
## INFO  [08:02:24.618] [mlr3] Applying learner 'regr.ranger' on task 'PredictionSet[1:13]' (iter 5/10)
## INFO  [08:02:24.654] [mlr3] Applying learner 'regr.ranger' on task 'PredictionSet[1:13]' (iter 6/10)
## INFO  [08:02:24.685] [mlr3] Applying learner 'regr.ranger' on task 'PredictionSet[1:13]' (iter 7/10)
## INFO  [08:02:24.718] [mlr3] Applying learner 'regr.ranger' on task 'PredictionSet[1:13]' (iter 8/10)
## INFO  [08:02:24.750] [mlr3] Applying learner 'regr.ranger' on task 'PredictionSet[1:13]' (iter 9/10)
## INFO  [08:02:24.781] [mlr3] Applying learner 'regr.ranger' on task 'PredictionSet[1:13]' (iter 10/10)
## INFO  [08:02:24.813] [mlr3] Finished benchmark
## INFO  [08:02:24.842] [bbotk] Result of batch 1:
## INFO  [08:02:24.843] [bbotk]  max.depth num.trees regr.rmse warnings errors runtime_learners
## INFO  [08:02:24.843] [bbotk]         12       237   2.45986        0      0            0.225
## INFO  [08:02:24.843] [bbotk]                                 uhash
## INFO  [08:02:24.843] [bbotk]  f63eb8f1-b07a-4020-83d5-3c015821825f
## INFO  [08:02:24.845] [bbotk] Evaluating 1 configuration(s)
## INFO  [08:02:24.867] [mlr3] Running benchmark with 10 resampling iterations
## INFO  [08:02:24.872] [mlr3] Applying learner 'regr.ranger' on task 'PredictionSet[1:13]' (iter 1/10)
## INFO  [08:02:24.892] [mlr3] Applying learner 'regr.ranger' on task 'PredictionSet[1:13]' (iter 2/10)
## INFO  [08:02:24.913] [mlr3] Applying learner 'regr.ranger' on task 'PredictionSet[1:13]' (iter 3/10)
## INFO  [08:02:24.934] [mlr3] Applying learner 'regr.ranger' on task 'PredictionSet[1:13]' (iter 4/10)
## INFO  [08:02:24.955] [mlr3] Applying learner 'regr.ranger' on task 'PredictionSet[1:13]' (iter 5/10)
## INFO  [08:02:24.975] [mlr3] Applying learner 'regr.ranger' on task 'PredictionSet[1:13]' (iter 6/10)
## INFO  [08:02:24.995] [mlr3] Applying learner 'regr.ranger' on task 'PredictionSet[1:13]' (iter 7/10)
## INFO  [08:02:25.018] [mlr3] Applying learner 'regr.ranger' on task 'PredictionSet[1:13]' (iter 8/10)
## INFO  [08:02:25.038] [mlr3] Applying learner 'regr.ranger' on task 'PredictionSet[1:13]' (iter 9/10)
## INFO  [08:02:25.058] [mlr3] Applying learner 'regr.ranger' on task 'PredictionSet[1:13]' (iter 10/10)
## INFO  [08:02:25.090] [mlr3] Finished benchmark
## INFO  [08:02:25.124] [bbotk] Result of batch 2:
## INFO  [08:02:25.125] [bbotk]  max.depth num.trees regr.rmse warnings errors runtime_learners
## INFO  [08:02:25.125] [bbotk]         17        79  2.430331        0      0            0.105
## INFO  [08:02:25.125] [bbotk]                                 uhash
## INFO  [08:02:25.125] [bbotk]  4803fda3-d14a-4013-9497-16c26f40688f
## INFO  [08:02:25.127] [bbotk] Evaluating 1 configuration(s)
## INFO  [08:02:25.148] [mlr3] Running benchmark with 10 resampling iterations
## INFO  [08:02:25.153] [mlr3] Applying learner 'regr.ranger' on task 'PredictionSet[1:13]' (iter 1/10)
## INFO  [08:02:25.195] [mlr3] Applying learner 'regr.ranger' on task 'PredictionSet[1:13]' (iter 2/10)
## INFO  [08:02:25.238] [mlr3] Applying learner 'regr.ranger' on task 'PredictionSet[1:13]' (iter 3/10)
## INFO  [08:02:25.278] [mlr3] Applying learner 'regr.ranger' on task 'PredictionSet[1:13]' (iter 4/10)
## INFO  [08:02:25.320] [mlr3] Applying learner 'regr.ranger' on task 'PredictionSet[1:13]' (iter 5/10)
## INFO  [08:02:25.360] [mlr3] Applying learner 'regr.ranger' on task 'PredictionSet[1:13]' (iter 6/10)
## INFO  [08:02:25.401] [mlr3] Applying learner 'regr.ranger' on task 'PredictionSet[1:13]' (iter 7/10)
## INFO  [08:02:25.441] [mlr3] Applying learner 'regr.ranger' on task 'PredictionSet[1:13]' (iter 8/10)
## INFO  [08:02:25.482] [mlr3] Applying learner 'regr.ranger' on task 'PredictionSet[1:13]' (iter 9/10)
## INFO  [08:02:25.522] [mlr3] Applying learner 'regr.ranger' on task 'PredictionSet[1:13]' (iter 10/10)
## INFO  [08:02:25.563] [mlr3] Finished benchmark
## INFO  [08:02:25.594] [bbotk] Result of batch 3:
## INFO  [08:02:25.595] [bbotk]  max.depth num.trees regr.rmse warnings errors runtime_learners
## INFO  [08:02:25.595] [bbotk]         18       369  2.422086        0      0            0.305
## INFO  [08:02:25.595] [bbotk]                                 uhash
## INFO  [08:02:25.595] [bbotk]  394422f5-d417-4c74-96f9-987f75a82655
## INFO  [08:02:25.597] [bbotk] Evaluating 1 configuration(s)
## INFO  [08:02:25.619] [mlr3] Running benchmark with 10 resampling iterations
## INFO  [08:02:25.633] [mlr3] Applying learner 'regr.ranger' on task 'PredictionSet[1:13]' (iter 1/10)
## INFO  [08:02:25.664] [mlr3] Applying learner 'regr.ranger' on task 'PredictionSet[1:13]' (iter 2/10)
## INFO  [08:02:25.694] [mlr3] Applying learner 'regr.ranger' on task 'PredictionSet[1:13]' (iter 3/10)
## INFO  [08:02:25.722] [mlr3] Applying learner 'regr.ranger' on task 'PredictionSet[1:13]' (iter 4/10)
## INFO  [08:02:25.749] [mlr3] Applying learner 'regr.ranger' on task 'PredictionSet[1:13]' (iter 5/10)
## INFO  [08:02:25.777] [mlr3] Applying learner 'regr.ranger' on task 'PredictionSet[1:13]' (iter 6/10)
## INFO  [08:02:25.804] [mlr3] Applying learner 'regr.ranger' on task 'PredictionSet[1:13]' (iter 7/10)
## INFO  [08:02:25.833] [mlr3] Applying learner 'regr.ranger' on task 'PredictionSet[1:13]' (iter 8/10)
## INFO  [08:02:25.861] [mlr3] Applying learner 'regr.ranger' on task 'PredictionSet[1:13]' (iter 9/10)
## INFO  [08:02:25.888] [mlr3] Applying learner 'regr.ranger' on task 'PredictionSet[1:13]' (iter 10/10)
## INFO  [08:02:25.917] [mlr3] Finished benchmark
## INFO  [08:02:25.947] [bbotk] Result of batch 4:
## INFO  [08:02:25.949] [bbotk]  max.depth num.trees regr.rmse warnings errors runtime_learners
## INFO  [08:02:25.949] [bbotk]          3       343  3.233941        0      0            0.175
## INFO  [08:02:25.949] [bbotk]                                 uhash
## INFO  [08:02:25.949] [bbotk]  2671828e-018f-414f-b77c-253b9b58f060
## INFO  [08:02:25.950] [bbotk] Evaluating 1 configuration(s)
## INFO  [08:02:25.972] [mlr3] Running benchmark with 10 resampling iterations
## INFO  [08:02:25.977] [mlr3] Applying learner 'regr.ranger' on task 'PredictionSet[1:13]' (iter 1/10)
## INFO  [08:02:26.001] [mlr3] Applying learner 'regr.ranger' on task 'PredictionSet[1:13]' (iter 2/10)
## INFO  [08:02:26.023] [mlr3] Applying learner 'regr.ranger' on task 'PredictionSet[1:13]' (iter 3/10)
## INFO  [08:02:26.045] [mlr3] Applying learner 'regr.ranger' on task 'PredictionSet[1:13]' (iter 4/10)
## INFO  [08:02:26.068] [mlr3] Applying learner 'regr.ranger' on task 'PredictionSet[1:13]' (iter 5/10)
## INFO  [08:02:26.091] [mlr3] Applying learner 'regr.ranger' on task 'PredictionSet[1:13]' (iter 6/10)
## INFO  [08:02:26.122] [mlr3] Applying learner 'regr.ranger' on task 'PredictionSet[1:13]' (iter 7/10)
## INFO  [08:02:26.149] [mlr3] Applying learner 'regr.ranger' on task 'PredictionSet[1:13]' (iter 8/10)
## INFO  [08:02:26.172] [mlr3] Applying learner 'regr.ranger' on task 'PredictionSet[1:13]' (iter 9/10)
## INFO  [08:02:26.194] [mlr3] Applying learner 'regr.ranger' on task 'PredictionSet[1:13]' (iter 10/10)
## INFO  [08:02:26.217] [mlr3] Finished benchmark
## INFO  [08:02:26.248] [bbotk] Result of batch 5:
## INFO  [08:02:26.250] [bbotk]  max.depth num.trees regr.rmse warnings errors runtime_learners
## INFO  [08:02:26.250] [bbotk]         13       106  2.463189        0      0            0.129
## INFO  [08:02:26.250] [bbotk]                                 uhash
## INFO  [08:02:26.250] [bbotk]  4ae08b30-fc0a-4053-ad95-8315196beb06
## INFO  [08:02:26.251] [bbotk] Evaluating 1 configuration(s)
## INFO  [08:02:26.272] [mlr3] Running benchmark with 10 resampling iterations
## INFO  [08:02:26.277] [mlr3] Applying learner 'regr.ranger' on task 'PredictionSet[1:13]' (iter 1/10)
## INFO  [08:02:26.309] [mlr3] Applying learner 'regr.ranger' on task 'PredictionSet[1:13]' (iter 2/10)
## INFO  [08:02:26.340] [mlr3] Applying learner 'regr.ranger' on task 'PredictionSet[1:13]' (iter 3/10)
## INFO  [08:02:26.373] [mlr3] Applying learner 'regr.ranger' on task 'PredictionSet[1:13]' (iter 4/10)
## INFO  [08:02:26.404] [mlr3] Applying learner 'regr.ranger' on task 'PredictionSet[1:13]' (iter 5/10)
## INFO  [08:02:26.437] [mlr3] Applying learner 'regr.ranger' on task 'PredictionSet[1:13]' (iter 6/10)
## INFO  [08:02:26.469] [mlr3] Applying learner 'regr.ranger' on task 'PredictionSet[1:13]' (iter 7/10)
## INFO  [08:02:26.500] [mlr3] Applying learner 'regr.ranger' on task 'PredictionSet[1:13]' (iter 8/10)
## INFO  [08:02:26.533] [mlr3] Applying learner 'regr.ranger' on task 'PredictionSet[1:13]' (iter 9/10)
## INFO  [08:02:26.564] [mlr3] Applying learner 'regr.ranger' on task 'PredictionSet[1:13]' (iter 10/10)
## INFO  [08:02:26.596] [mlr3] Finished benchmark
## INFO  [08:02:26.641] [bbotk] Result of batch 6:
## INFO  [08:02:26.642] [bbotk]  max.depth num.trees regr.rmse warnings errors runtime_learners
## INFO  [08:02:26.642] [bbotk]         13       237  2.466989        0      0            0.215
## INFO  [08:02:26.642] [bbotk]                                 uhash
## INFO  [08:02:26.642] [bbotk]  e1d15137-4b1f-4ab5-b07c-25d4fe89d61b
## INFO  [08:02:26.644] [bbotk] Evaluating 1 configuration(s)
## INFO  [08:02:26.666] [mlr3] Running benchmark with 10 resampling iterations
## INFO  [08:02:26.671] [mlr3] Applying learner 'regr.ranger' on task 'PredictionSet[1:13]' (iter 1/10)
## INFO  [08:02:26.706] [mlr3] Applying learner 'regr.ranger' on task 'PredictionSet[1:13]' (iter 2/10)
## INFO  [08:02:26.743] [mlr3] Applying learner 'regr.ranger' on task 'PredictionSet[1:13]' (iter 3/10)
## INFO  [08:02:26.778] [mlr3] Applying learner 'regr.ranger' on task 'PredictionSet[1:13]' (iter 4/10)
## INFO  [08:02:26.815] [mlr3] Applying learner 'regr.ranger' on task 'PredictionSet[1:13]' (iter 5/10)
## INFO  [08:02:26.850] [mlr3] Applying learner 'regr.ranger' on task 'PredictionSet[1:13]' (iter 6/10)
## INFO  [08:02:26.887] [mlr3] Applying learner 'regr.ranger' on task 'PredictionSet[1:13]' (iter 7/10)
## INFO  [08:02:26.922] [mlr3] Applying learner 'regr.ranger' on task 'PredictionSet[1:13]' (iter 8/10)
## INFO  [08:02:26.958] [mlr3] Applying learner 'regr.ranger' on task 'PredictionSet[1:13]' (iter 9/10)
## INFO  [08:02:26.994] [mlr3] Applying learner 'regr.ranger' on task 'PredictionSet[1:13]' (iter 10/10)
## INFO  [08:02:27.029] [mlr3] Finished benchmark
## INFO  [08:02:27.063] [bbotk] Result of batch 7:
## INFO  [08:02:27.064] [bbotk]  max.depth num.trees regr.rmse warnings errors runtime_learners
## INFO  [08:02:27.064] [bbotk]         18       290  2.442986        0      0             0.25
## INFO  [08:02:27.064] [bbotk]                                 uhash
## INFO  [08:02:27.064] [bbotk]  faf5bddc-3a20-400f-bee5-5088f4c069e1
## INFO  [08:02:27.066] [bbotk] Evaluating 1 configuration(s)
## INFO  [08:02:27.087] [mlr3] Running benchmark with 10 resampling iterations
## INFO  [08:02:27.092] [mlr3] Applying learner 'regr.ranger' on task 'PredictionSet[1:13]' (iter 1/10)
## INFO  [08:02:27.138] [mlr3] Applying learner 'regr.ranger' on task 'PredictionSet[1:13]' (iter 2/10)
## INFO  [08:02:27.177] [mlr3] Applying learner 'regr.ranger' on task 'PredictionSet[1:13]' (iter 3/10)
## INFO  [08:02:27.214] [mlr3] Applying learner 'regr.ranger' on task 'PredictionSet[1:13]' (iter 4/10)
## INFO  [08:02:27.250] [mlr3] Applying learner 'regr.ranger' on task 'PredictionSet[1:13]' (iter 5/10)
## INFO  [08:02:27.286] [mlr3] Applying learner 'regr.ranger' on task 'PredictionSet[1:13]' (iter 6/10)
## INFO  [08:02:27.321] [mlr3] Applying learner 'regr.ranger' on task 'PredictionSet[1:13]' (iter 7/10)
## INFO  [08:02:27.357] [mlr3] Applying learner 'regr.ranger' on task 'PredictionSet[1:13]' (iter 8/10)
## INFO  [08:02:27.391] [mlr3] Applying learner 'regr.ranger' on task 'PredictionSet[1:13]' (iter 9/10)
## INFO  [08:02:27.427] [mlr3] Applying learner 'regr.ranger' on task 'PredictionSet[1:13]' (iter 10/10)
## INFO  [08:02:27.461] [mlr3] Finished benchmark
## INFO  [08:02:27.494] [bbotk] Result of batch 8:
## INFO  [08:02:27.495] [bbotk]  max.depth num.trees regr.rmse warnings errors runtime_learners
## INFO  [08:02:27.495] [bbotk]         16       290  2.431797        0      0            0.254
## INFO  [08:02:27.495] [bbotk]                                 uhash
## INFO  [08:02:27.495] [bbotk]  d0dd5bfd-7e67-4cd5-8c6f-87a7da151373
## INFO  [08:02:27.496] [bbotk] Evaluating 1 configuration(s)
## INFO  [08:02:27.518] [mlr3] Running benchmark with 10 resampling iterations
## INFO  [08:02:27.523] [mlr3] Applying learner 'regr.ranger' on task 'PredictionSet[1:13]' (iter 1/10)
## INFO  [08:02:27.548] [mlr3] Applying learner 'regr.ranger' on task 'PredictionSet[1:13]' (iter 2/10)
## INFO  [08:02:27.575] [mlr3] Applying learner 'regr.ranger' on task 'PredictionSet[1:13]' (iter 3/10)
## INFO  [08:02:27.600] [mlr3] Applying learner 'regr.ranger' on task 'PredictionSet[1:13]' (iter 4/10)
## INFO  [08:02:27.624] [mlr3] Applying learner 'regr.ranger' on task 'PredictionSet[1:13]' (iter 5/10)
## INFO  [08:02:27.683] [mlr3] Applying learner 'regr.ranger' on task 'PredictionSet[1:13]' (iter 6/10)
## INFO  [08:02:27.716] [mlr3] Applying learner 'regr.ranger' on task 'PredictionSet[1:13]' (iter 7/10)
## INFO  [08:02:27.745] [mlr3] Applying learner 'regr.ranger' on task 'PredictionSet[1:13]' (iter 8/10)
## INFO  [08:02:27.775] [mlr3] Applying learner 'regr.ranger' on task 'PredictionSet[1:13]' (iter 9/10)
## INFO  [08:02:27.802] [mlr3] Applying learner 'regr.ranger' on task 'PredictionSet[1:13]' (iter 10/10)
## INFO  [08:02:27.827] [mlr3] Finished benchmark
## INFO  [08:02:27.860] [bbotk] Result of batch 9:
## INFO  [08:02:27.862] [bbotk]  max.depth num.trees regr.rmse warnings errors runtime_learners
## INFO  [08:02:27.862] [bbotk]          7       158  2.481738        0      0            0.163
## INFO  [08:02:27.862] [bbotk]                                 uhash
## INFO  [08:02:27.862] [bbotk]  99565529-7641-47ec-b6d1-53caea3259e3
## INFO  [08:02:27.863] [bbotk] Evaluating 1 configuration(s)
## INFO  [08:02:27.886] [mlr3] Running benchmark with 10 resampling iterations
## INFO  [08:02:27.892] [mlr3] Applying learner 'regr.ranger' on task 'PredictionSet[1:13]' (iter 1/10)
## INFO  [08:02:27.927] [mlr3] Applying learner 'regr.ranger' on task 'PredictionSet[1:13]' (iter 2/10)
## INFO  [08:02:27.961] [mlr3] Applying learner 'regr.ranger' on task 'PredictionSet[1:13]' (iter 3/10)
## INFO  [08:02:27.994] [mlr3] Applying learner 'regr.ranger' on task 'PredictionSet[1:13]' (iter 4/10)
## INFO  [08:02:28.029] [mlr3] Applying learner 'regr.ranger' on task 'PredictionSet[1:13]' (iter 5/10)
## INFO  [08:02:28.062] [mlr3] Applying learner 'regr.ranger' on task 'PredictionSet[1:13]' (iter 6/10)
## INFO  [08:02:28.096] [mlr3] Applying learner 'regr.ranger' on task 'PredictionSet[1:13]' (iter 7/10)
## INFO  [08:02:28.129] [mlr3] Applying learner 'regr.ranger' on task 'PredictionSet[1:13]' (iter 8/10)
## INFO  [08:02:28.165] [mlr3] Applying learner 'regr.ranger' on task 'PredictionSet[1:13]' (iter 9/10)
## INFO  [08:02:28.198] [mlr3] Applying learner 'regr.ranger' on task 'PredictionSet[1:13]' (iter 10/10)
## INFO  [08:02:28.232] [mlr3] Finished benchmark
## INFO  [08:02:28.274] [bbotk] Result of batch 10:
## INFO  [08:02:28.275] [bbotk]  max.depth num.trees regr.rmse warnings errors runtime_learners
## INFO  [08:02:28.275] [bbotk]         20       264  2.439478        0      0            0.234
## INFO  [08:02:28.275] [bbotk]                                 uhash
## INFO  [08:02:28.275] [bbotk]  96b331e7-e8a0-44ef-a06b-56b3ce5f0c49
## INFO  [08:02:28.281] [bbotk] Finished optimizing after 10 evaluation(s)
## INFO  [08:02:28.282] [bbotk] Result:
## INFO  [08:02:28.283] [bbotk]  max.depth num.trees learner_param_vals  x_domain regr.rmse
## INFO  [08:02:28.283] [bbotk]         18       369          <list[3]> <list[2]>  2.422086
## INFO  [08:02:28.525] [bbotk] Starting to optimize 5 parameter(s) with '<TunerGridSearch>' and '<TerminatorEvals> [n_evals=10, k=0]'
## INFO  [08:02:28.532] [bbotk] Evaluating 1 configuration(s)
## INFO  [08:02:28.549] [mlr3] Running benchmark with 10 resampling iterations
## INFO  [08:02:28.554] [mlr3] Applying learner 'regr.svm' on task 'PredictionSet[1:13]' (iter 1/10)
## INFO  [08:02:28.572] [mlr3] Applying learner 'regr.svm' on task 'PredictionSet[1:13]' (iter 2/10)
## INFO  [08:02:28.599] [mlr3] Applying learner 'regr.svm' on task 'PredictionSet[1:13]' (iter 3/10)
## INFO  [08:02:28.622] [mlr3] Applying learner 'regr.svm' on task 'PredictionSet[1:13]' (iter 4/10)
## INFO  [08:02:28.644] [mlr3] Applying learner 'regr.svm' on task 'PredictionSet[1:13]' (iter 5/10)
## INFO  [08:02:28.664] [mlr3] Applying learner 'regr.svm' on task 'PredictionSet[1:13]' (iter 6/10)
## INFO  [08:02:28.684] [mlr3] Applying learner 'regr.svm' on task 'PredictionSet[1:13]' (iter 7/10)
## INFO  [08:02:28.703] [mlr3] Applying learner 'regr.svm' on task 'PredictionSet[1:13]' (iter 8/10)
## INFO  [08:02:28.721] [mlr3] Applying learner 'regr.svm' on task 'PredictionSet[1:13]' (iter 9/10)
## INFO  [08:02:28.739] [mlr3] Applying learner 'regr.svm' on task 'PredictionSet[1:13]' (iter 10/10)
## INFO  [08:02:28.757] [mlr3] Finished benchmark
## INFO  [08:02:28.787] [bbotk] Result of batch 1:
## INFO  [08:02:28.788] [bbotk]  tolerance    gamma   epsilon           type  kernel regr.rmse warnings errors
## INFO  [08:02:28.788] [bbotk]  0.6431429 42.85729 0.8428571 eps-regression sigmoid  1713.642        0      0
## INFO  [08:02:28.788] [bbotk]  runtime_learners                                uhash
## INFO  [08:02:28.788] [bbotk]             0.086 7b1cc11e-3cae-4b5c-92a9-95c174c82b8d
## INFO  [08:02:28.790] [bbotk] Evaluating 1 configuration(s)
## INFO  [08:02:28.805] [mlr3] Running benchmark with 10 resampling iterations
## INFO  [08:02:28.810] [mlr3] Applying learner 'regr.svm' on task 'PredictionSet[1:13]' (iter 1/10)
## INFO  [08:02:28.829] [mlr3] Applying learner 'regr.svm' on task 'PredictionSet[1:13]' (iter 2/10)
## INFO  [08:02:28.849] [mlr3] Applying learner 'regr.svm' on task 'PredictionSet[1:13]' (iter 3/10)
## INFO  [08:02:28.868] [mlr3] Applying learner 'regr.svm' on task 'PredictionSet[1:13]' (iter 4/10)
## INFO  [08:02:28.886] [mlr3] Applying learner 'regr.svm' on task 'PredictionSet[1:13]' (iter 5/10)
## INFO  [08:02:28.905] [mlr3] Applying learner 'regr.svm' on task 'PredictionSet[1:13]' (iter 6/10)
## INFO  [08:02:28.925] [mlr3] Applying learner 'regr.svm' on task 'PredictionSet[1:13]' (iter 7/10)
## INFO  [08:02:28.944] [mlr3] Applying learner 'regr.svm' on task 'PredictionSet[1:13]' (iter 8/10)
## INFO  [08:02:28.962] [mlr3] Applying learner 'regr.svm' on task 'PredictionSet[1:13]' (iter 9/10)
## INFO  [08:02:28.990] [mlr3] Applying learner 'regr.svm' on task 'PredictionSet[1:13]' (iter 10/10)
## INFO  [08:02:29.012] [mlr3] Finished benchmark
## INFO  [08:02:29.051] [bbotk] Result of batch 2:
## INFO  [08:02:29.052] [bbotk]  tolerance    gamma   epsilon           type  kernel regr.rmse warnings errors
## INFO  [08:02:29.052] [bbotk]      0.001 14.28643 0.3857143 eps-regression sigmoid  1686.489        0      0
## INFO  [08:02:29.052] [bbotk]  runtime_learners                                uhash
## INFO  [08:02:29.052] [bbotk]              0.09 85fcb030-9755-4aea-a218-1eaa8474b1ce
## INFO  [08:02:29.054] [bbotk] Evaluating 1 configuration(s)
## INFO  [08:02:29.071] [mlr3] Running benchmark with 10 resampling iterations
## INFO  [08:02:29.077] [mlr3] Applying learner 'regr.svm' on task 'PredictionSet[1:13]' (iter 1/10)
## INFO  [08:02:29.095] [mlr3] Applying learner 'regr.svm' on task 'PredictionSet[1:13]' (iter 2/10)
## INFO  [08:02:29.113] [mlr3] Applying learner 'regr.svm' on task 'PredictionSet[1:13]' (iter 3/10)
## INFO  [08:02:29.132] [mlr3] Applying learner 'regr.svm' on task 'PredictionSet[1:13]' (iter 4/10)
## INFO  [08:02:29.151] [mlr3] Applying learner 'regr.svm' on task 'PredictionSet[1:13]' (iter 5/10)
## INFO  [08:02:29.169] [mlr3] Applying learner 'regr.svm' on task 'PredictionSet[1:13]' (iter 6/10)
## INFO  [08:02:29.187] [mlr3] Applying learner 'regr.svm' on task 'PredictionSet[1:13]' (iter 7/10)
## INFO  [08:02:29.206] [mlr3] Applying learner 'regr.svm' on task 'PredictionSet[1:13]' (iter 8/10)
## INFO  [08:02:29.225] [mlr3] Applying learner 'regr.svm' on task 'PredictionSet[1:13]' (iter 9/10)
## INFO  [08:02:29.243] [mlr3] Applying learner 'regr.svm' on task 'PredictionSet[1:13]' (iter 10/10)
## INFO  [08:02:29.261] [mlr3] Finished benchmark
## INFO  [08:02:29.294] [bbotk] Result of batch 3:
## INFO  [08:02:29.296] [bbotk]   tolerance   gamma   epsilon           type  kernel regr.rmse warnings errors
## INFO  [08:02:29.296] [bbotk]  0.06521429 25.0005 0.7857143 eps-regression sigmoid  1706.152        0      0
## INFO  [08:02:29.296] [bbotk]  runtime_learners                                uhash
## INFO  [08:02:29.296] [bbotk]             0.081 a09931a5-b8ca-4662-b3f7-a7fa60b40e27
## INFO  [08:02:29.297] [bbotk] Evaluating 1 configuration(s)
## INFO  [08:02:29.313] [mlr3] Running benchmark with 10 resampling iterations
## INFO  [08:02:29.318] [mlr3] Applying learner 'regr.svm' on task 'PredictionSet[1:13]' (iter 1/10)
## INFO  [08:02:29.334] [mlr3] Applying learner 'regr.svm' on task 'PredictionSet[1:13]' (iter 2/10)
## INFO  [08:02:29.357] [mlr3] Applying learner 'regr.svm' on task 'PredictionSet[1:13]' (iter 3/10)
## INFO  [08:02:29.378] [mlr3] Applying learner 'regr.svm' on task 'PredictionSet[1:13]' (iter 4/10)
## INFO  [08:02:29.397] [mlr3] Applying learner 'regr.svm' on task 'PredictionSet[1:13]' (iter 5/10)
## INFO  [08:02:29.416] [mlr3] Applying learner 'regr.svm' on task 'PredictionSet[1:13]' (iter 6/10)
## INFO  [08:02:29.436] [mlr3] Applying learner 'regr.svm' on task 'PredictionSet[1:13]' (iter 7/10)
## INFO  [08:02:29.453] [mlr3] Applying learner 'regr.svm' on task 'PredictionSet[1:13]' (iter 8/10)
## INFO  [08:02:29.469] [mlr3] Applying learner 'regr.svm' on task 'PredictionSet[1:13]' (iter 9/10)
## INFO  [08:02:29.485] [mlr3] Applying learner 'regr.svm' on task 'PredictionSet[1:13]' (iter 10/10)
## INFO  [08:02:29.501] [mlr3] Finished benchmark
## INFO  [08:02:29.534] [bbotk] Result of batch 4:
## INFO  [08:02:29.535] [bbotk]  tolerance    gamma   epsilon           type kernel regr.rmse warnings errors
## INFO  [08:02:29.535] [bbotk]  0.1936429 17.85779 0.8428571 eps-regression radial  44.11481        0      0
## INFO  [08:02:29.535] [bbotk]  runtime_learners                                uhash
## INFO  [08:02:29.535] [bbotk]             0.066 c17bb31d-cc7c-48e6-bf37-6a08275e0ec7
## INFO  [08:02:29.537] [bbotk] Evaluating 1 configuration(s)
## INFO  [08:02:29.552] [mlr3] Running benchmark with 10 resampling iterations
## INFO  [08:02:29.557] [mlr3] Applying learner 'regr.svm' on task 'PredictionSet[1:13]' (iter 1/10)
## INFO  [08:02:29.575] [mlr3] Applying learner 'regr.svm' on task 'PredictionSet[1:13]' (iter 2/10)
## INFO  [08:02:29.594] [mlr3] Applying learner 'regr.svm' on task 'PredictionSet[1:13]' (iter 3/10)
## INFO  [08:02:29.612] [mlr3] Applying learner 'regr.svm' on task 'PredictionSet[1:13]' (iter 4/10)
## INFO  [08:02:29.630] [mlr3] Applying learner 'regr.svm' on task 'PredictionSet[1:13]' (iter 5/10)
## INFO  [08:02:29.648] [mlr3] Applying learner 'regr.svm' on task 'PredictionSet[1:13]' (iter 6/10)
## INFO  [08:02:29.668] [mlr3] Applying learner 'regr.svm' on task 'PredictionSet[1:13]' (iter 7/10)
## INFO  [08:02:29.686] [mlr3] Applying learner 'regr.svm' on task 'PredictionSet[1:13]' (iter 8/10)
## INFO  [08:02:29.704] [mlr3] Applying learner 'regr.svm' on task 'PredictionSet[1:13]' (iter 9/10)
## INFO  [08:02:29.722] [mlr3] Applying learner 'regr.svm' on task 'PredictionSet[1:13]' (iter 10/10)
## INFO  [08:02:29.751] [mlr3] Finished benchmark
## INFO  [08:02:29.790] [bbotk] Result of batch 5:
## INFO  [08:02:29.792] [bbotk]  tolerance    gamma   epsilon           type  kernel regr.rmse warnings errors
## INFO  [08:02:29.792] [bbotk]  0.7073571 42.85729 0.2142857 eps-regression sigmoid   1706.92        0      0
## INFO  [08:02:29.792] [bbotk]  runtime_learners                                uhash
## INFO  [08:02:29.792] [bbotk]              0.08 a53003e8-9809-437a-99be-3b5891a8f318
## INFO  [08:02:29.794] [bbotk] Evaluating 1 configuration(s)
## INFO  [08:02:29.812] [mlr3] Running benchmark with 10 resampling iterations
## INFO  [08:02:29.817] [mlr3] Applying learner 'regr.svm' on task 'PredictionSet[1:13]' (iter 1/10)
## INFO  [08:02:29.834] [mlr3] Applying learner 'regr.svm' on task 'PredictionSet[1:13]' (iter 2/10)
## INFO  [08:02:29.850] [mlr3] Applying learner 'regr.svm' on task 'PredictionSet[1:13]' (iter 3/10)
## INFO  [08:02:29.866] [mlr3] Applying learner 'regr.svm' on task 'PredictionSet[1:13]' (iter 4/10)
## INFO  [08:02:29.882] [mlr3] Applying learner 'regr.svm' on task 'PredictionSet[1:13]' (iter 5/10)
## INFO  [08:02:29.899] [mlr3] Applying learner 'regr.svm' on task 'PredictionSet[1:13]' (iter 6/10)
## INFO  [08:02:29.915] [mlr3] Applying learner 'regr.svm' on task 'PredictionSet[1:13]' (iter 7/10)
## INFO  [08:02:29.931] [mlr3] Applying learner 'regr.svm' on task 'PredictionSet[1:13]' (iter 8/10)
## INFO  [08:02:29.947] [mlr3] Applying learner 'regr.svm' on task 'PredictionSet[1:13]' (iter 9/10)
## INFO  [08:02:29.965] [mlr3] Applying learner 'regr.svm' on task 'PredictionSet[1:13]' (iter 10/10)
## INFO  [08:02:29.981] [mlr3] Finished benchmark
## INFO  [08:02:30.012] [bbotk] Result of batch 6:
## INFO  [08:02:30.014] [bbotk]  tolerance    gamma   epsilon           type kernel regr.rmse warnings errors
## INFO  [08:02:30.014] [bbotk]  0.3862857 32.14321 0.7857143 eps-regression radial  44.65529        0      0
## INFO  [08:02:30.014] [bbotk]  runtime_learners                                uhash
## INFO  [08:02:30.014] [bbotk]             0.059 00ad3f00-0df4-48b2-a402-4e10b51694d1
## INFO  [08:02:30.015] [bbotk] Evaluating 1 configuration(s)
## INFO  [08:02:30.032] [mlr3] Running benchmark with 10 resampling iterations
## INFO  [08:02:30.038] [mlr3] Applying learner 'regr.svm' on task 'PredictionSet[1:13]' (iter 1/10)
## INFO  [08:02:30.056] [mlr3] Applying learner 'regr.svm' on task 'PredictionSet[1:13]' (iter 2/10)
## INFO  [08:02:30.081] [mlr3] Applying learner 'regr.svm' on task 'PredictionSet[1:13]' (iter 3/10)
## INFO  [08:02:30.102] [mlr3] Applying learner 'regr.svm' on task 'PredictionSet[1:13]' (iter 4/10)
## INFO  [08:02:30.125] [mlr3] Applying learner 'regr.svm' on task 'PredictionSet[1:13]' (iter 5/10)
## INFO  [08:02:30.147] [mlr3] Applying learner 'regr.svm' on task 'PredictionSet[1:13]' (iter 6/10)
## INFO  [08:02:30.166] [mlr3] Applying learner 'regr.svm' on task 'PredictionSet[1:13]' (iter 7/10)
## INFO  [08:02:30.187] [mlr3] Applying learner 'regr.svm' on task 'PredictionSet[1:13]' (iter 8/10)
## INFO  [08:02:30.205] [mlr3] Applying learner 'regr.svm' on task 'PredictionSet[1:13]' (iter 9/10)
## INFO  [08:02:30.223] [mlr3] Applying learner 'regr.svm' on task 'PredictionSet[1:13]' (iter 10/10)
## INFO  [08:02:30.241] [mlr3] Finished benchmark
## INFO  [08:02:30.274] [bbotk] Result of batch 7:
## INFO  [08:02:30.275] [bbotk]  tolerance gamma   epsilon           type  kernel regr.rmse warnings errors
## INFO  [08:02:30.275] [bbotk]  0.5147143    50 0.3857143 eps-regression sigmoid  1705.155        0      0
## INFO  [08:02:30.275] [bbotk]  runtime_learners                                uhash
## INFO  [08:02:30.275] [bbotk]              0.09 01c32bf8-f649-47e5-848a-8c8bdda0a2c5
## INFO  [08:02:30.277] [bbotk] Evaluating 1 configuration(s)
## INFO  [08:02:30.292] [mlr3] Running benchmark with 10 resampling iterations
## INFO  [08:02:30.297] [mlr3] Applying learner 'regr.svm' on task 'PredictionSet[1:13]' (iter 1/10)
## INFO  [08:02:30.316] [mlr3] Applying learner 'regr.svm' on task 'PredictionSet[1:13]' (iter 2/10)
## INFO  [08:02:30.337] [mlr3] Applying learner 'regr.svm' on task 'PredictionSet[1:13]' (iter 3/10)
## INFO  [08:02:30.357] [mlr3] Applying learner 'regr.svm' on task 'PredictionSet[1:13]' (iter 4/10)
## INFO  [08:02:30.376] [mlr3] Applying learner 'regr.svm' on task 'PredictionSet[1:13]' (iter 5/10)
## INFO  [08:02:30.395] [mlr3] Applying learner 'regr.svm' on task 'PredictionSet[1:13]' (iter 6/10)
## INFO  [08:02:30.416] [mlr3] Applying learner 'regr.svm' on task 'PredictionSet[1:13]' (iter 7/10)
## INFO  [08:02:30.436] [mlr3] Applying learner 'regr.svm' on task 'PredictionSet[1:13]' (iter 8/10)
## INFO  [08:02:30.455] [mlr3] Applying learner 'regr.svm' on task 'PredictionSet[1:13]' (iter 9/10)
## INFO  [08:02:30.484] [mlr3] Applying learner 'regr.svm' on task 'PredictionSet[1:13]' (iter 10/10)
## INFO  [08:02:30.507] [mlr3] Finished benchmark
## INFO  [08:02:30.545] [bbotk] Result of batch 8:
## INFO  [08:02:30.547] [bbotk]  tolerance    gamma   epsilon           type  kernel regr.rmse warnings errors
## INFO  [08:02:30.547] [bbotk]      0.001 7.143714 0.6142857 eps-regression sigmoid  1700.018        0      0
## INFO  [08:02:30.547] [bbotk]  runtime_learners                                uhash
## INFO  [08:02:30.547] [bbotk]             0.105 4b638e63-51c4-4356-9b91-6cb632a610a6
## INFO  [08:02:30.549] [bbotk] Evaluating 1 configuration(s)
## INFO  [08:02:30.566] [mlr3] Running benchmark with 10 resampling iterations
## INFO  [08:02:30.571] [mlr3] Applying learner 'regr.svm' on task 'PredictionSet[1:13]' (iter 1/10)
## INFO  [08:02:30.587] [mlr3] Applying learner 'regr.svm' on task 'PredictionSet[1:13]' (iter 2/10)
## INFO  [08:02:30.602] [mlr3] Applying learner 'regr.svm' on task 'PredictionSet[1:13]' (iter 3/10)
## INFO  [08:02:30.617] [mlr3] Applying learner 'regr.svm' on task 'PredictionSet[1:13]' (iter 4/10)
## INFO  [08:02:30.634] [mlr3] Applying learner 'regr.svm' on task 'PredictionSet[1:13]' (iter 5/10)
## INFO  [08:02:30.650] [mlr3] Applying learner 'regr.svm' on task 'PredictionSet[1:13]' (iter 6/10)
## INFO  [08:02:30.666] [mlr3] Applying learner 'regr.svm' on task 'PredictionSet[1:13]' (iter 7/10)
## INFO  [08:02:30.681] [mlr3] Applying learner 'regr.svm' on task 'PredictionSet[1:13]' (iter 8/10)
## INFO  [08:02:30.696] [mlr3] Applying learner 'regr.svm' on task 'PredictionSet[1:13]' (iter 9/10)
## INFO  [08:02:30.714] [mlr3] Applying learner 'regr.svm' on task 'PredictionSet[1:13]' (iter 10/10)
## INFO  [08:02:30.729] [mlr3] Finished benchmark
## INFO  [08:02:30.761] [bbotk] Result of batch 9:
## INFO  [08:02:30.762] [bbotk]  tolerance    gamma   epsilon           type kernel regr.rmse warnings errors
## INFO  [08:02:30.762] [bbotk]  0.7073571 42.85729 0.8428571 eps-regression radial  47.54574        0      0
## INFO  [08:02:30.762] [bbotk]  runtime_learners                                uhash
## INFO  [08:02:30.762] [bbotk]             0.058 bc9ccd86-6feb-4fa5-9755-d15b5f796197
## INFO  [08:02:30.764] [bbotk] Evaluating 1 configuration(s)
## INFO  [08:02:30.779] [mlr3] Running benchmark with 10 resampling iterations
## INFO  [08:02:30.785] [mlr3] Applying learner 'regr.svm' on task 'PredictionSet[1:13]' (iter 1/10)
## INFO  [08:02:30.810] [mlr3] Applying learner 'regr.svm' on task 'PredictionSet[1:13]' (iter 2/10)
## INFO  [08:02:30.829] [mlr3] Applying learner 'regr.svm' on task 'PredictionSet[1:13]' (iter 3/10)
## INFO  [08:02:30.849] [mlr3] Applying learner 'regr.svm' on task 'PredictionSet[1:13]' (iter 4/10)
## INFO  [08:02:30.871] [mlr3] Applying learner 'regr.svm' on task 'PredictionSet[1:13]' (iter 5/10)
## INFO  [08:02:30.889] [mlr3] Applying learner 'regr.svm' on task 'PredictionSet[1:13]' (iter 6/10)
## INFO  [08:02:30.907] [mlr3] Applying learner 'regr.svm' on task 'PredictionSet[1:13]' (iter 7/10)
## INFO  [08:02:30.923] [mlr3] Applying learner 'regr.svm' on task 'PredictionSet[1:13]' (iter 8/10)
## INFO  [08:02:30.942] [mlr3] Applying learner 'regr.svm' on task 'PredictionSet[1:13]' (iter 9/10)
## INFO  [08:02:30.958] [mlr3] Applying learner 'regr.svm' on task 'PredictionSet[1:13]' (iter 10/10)
## INFO  [08:02:30.975] [mlr3] Finished benchmark
## INFO  [08:02:31.006] [bbotk] Result of batch 10:
## INFO  [08:02:31.008] [bbotk]  tolerance gamma   epsilon           type kernel regr.rmse warnings errors
## INFO  [08:02:31.008] [bbotk]  0.2578571 0.001 0.4428571 eps-regression radial  39.05147        0      0
## INFO  [08:02:31.008] [bbotk]  runtime_learners                                uhash
## INFO  [08:02:31.008] [bbotk]             0.074 d1a2b4fe-738c-45e7-b0b0-2a4de7f769ad
## INFO  [08:02:31.014] [bbotk] Finished optimizing after 10 evaluation(s)
## INFO  [08:02:31.014] [bbotk] Result:
## INFO  [08:02:31.015] [bbotk]  tolerance gamma   epsilon           type kernel learner_param_vals  x_domain
## INFO  [08:02:31.015] [bbotk]  0.2578571 0.001 0.4428571 eps-regression radial          <list[5]> <list[5]>
## INFO  [08:02:31.015] [bbotk]  regr.rmse
## INFO  [08:02:31.015] [bbotk]   39.05147
## INFO  [08:02:31.172] [bbotk] Starting to optimize 3 parameter(s) with '<TunerGridSearch>' and '<TerminatorEvals> [n_evals=10, k=0]'
## INFO  [08:02:31.176] [bbotk] Evaluating 1 configuration(s)
## INFO  [08:02:31.189] [mlr3] Running benchmark with 10 resampling iterations
## INFO  [08:02:31.195] [mlr3] Applying learner 'regr.kknn' on task 'PredictionSet[1:13]' (iter 1/10)
## INFO  [08:02:31.216] [mlr3] Applying learner 'regr.kknn' on task 'PredictionSet[1:13]' (iter 2/10)
## INFO  [08:02:31.238] [mlr3] Applying learner 'regr.kknn' on task 'PredictionSet[1:13]' (iter 3/10)
## INFO  [08:02:31.259] [mlr3] Applying learner 'regr.kknn' on task 'PredictionSet[1:13]' (iter 4/10)
## INFO  [08:02:31.280] [mlr3] Applying learner 'regr.kknn' on task 'PredictionSet[1:13]' (iter 5/10)
## INFO  [08:02:31.301] [mlr3] Applying learner 'regr.kknn' on task 'PredictionSet[1:13]' (iter 6/10)
## INFO  [08:02:31.323] [mlr3] Applying learner 'regr.kknn' on task 'PredictionSet[1:13]' (iter 7/10)
## INFO  [08:02:31.344] [mlr3] Applying learner 'regr.kknn' on task 'PredictionSet[1:13]' (iter 8/10)
## INFO  [08:02:31.364] [mlr3] Applying learner 'regr.kknn' on task 'PredictionSet[1:13]' (iter 9/10)
## INFO  [08:02:31.386] [mlr3] Applying learner 'regr.kknn' on task 'PredictionSet[1:13]' (iter 10/10)
## INFO  [08:02:31.407] [mlr3] Finished benchmark
## INFO  [08:02:31.436] [bbotk] Result of batch 1:
## INFO  [08:02:31.438] [bbotk]   k distance kernel regr.rmse warnings errors runtime_learners
## INFO  [08:02:31.438] [bbotk]  10 12.89286   rank  23.24775        0      0            0.107
## INFO  [08:02:31.438] [bbotk]                                 uhash
## INFO  [08:02:31.438] [bbotk]  47dbcae7-5814-4a44-afb7-ec8280da4b19
## INFO  [08:02:31.439] [bbotk] Evaluating 1 configuration(s)
## INFO  [08:02:31.450] [mlr3] Running benchmark with 10 resampling iterations
## INFO  [08:02:31.455] [mlr3] Applying learner 'regr.kknn' on task 'PredictionSet[1:13]' (iter 1/10)
## INFO  [08:02:31.484] [mlr3] Applying learner 'regr.kknn' on task 'PredictionSet[1:13]' (iter 2/10)
## INFO  [08:02:31.507] [mlr3] Applying learner 'regr.kknn' on task 'PredictionSet[1:13]' (iter 3/10)
## INFO  [08:02:31.532] [mlr3] Applying learner 'regr.kknn' on task 'PredictionSet[1:13]' (iter 4/10)
## INFO  [08:02:31.557] [mlr3] Applying learner 'regr.kknn' on task 'PredictionSet[1:13]' (iter 5/10)
## INFO  [08:02:31.578] [mlr3] Applying learner 'regr.kknn' on task 'PredictionSet[1:13]' (iter 6/10)
## INFO  [08:02:31.598] [mlr3] Applying learner 'regr.kknn' on task 'PredictionSet[1:13]' (iter 7/10)
## INFO  [08:02:31.620] [mlr3] Applying learner 'regr.kknn' on task 'PredictionSet[1:13]' (iter 8/10)
## INFO  [08:02:31.641] [mlr3] Applying learner 'regr.kknn' on task 'PredictionSet[1:13]' (iter 9/10)
## INFO  [08:02:31.661] [mlr3] Applying learner 'regr.kknn' on task 'PredictionSet[1:13]' (iter 10/10)
## INFO  [08:02:31.681] [mlr3] Finished benchmark
## INFO  [08:02:31.713] [bbotk] Result of batch 2:
## INFO  [08:02:31.715] [bbotk]  k distance   kernel regr.rmse warnings errors runtime_learners
## INFO  [08:02:31.715] [bbotk]  2 7.207143 biweight  21.78817        0      0            0.108
## INFO  [08:02:31.715] [bbotk]                                 uhash
## INFO  [08:02:31.715] [bbotk]  079f27e5-4920-4025-bcb9-c43901b2beff
## INFO  [08:02:31.716] [bbotk] Evaluating 1 configuration(s)
## INFO  [08:02:31.726] [mlr3] Running benchmark with 10 resampling iterations
## INFO  [08:02:31.731] [mlr3] Applying learner 'regr.kknn' on task 'PredictionSet[1:13]' (iter 1/10)
## INFO  [08:02:31.752] [mlr3] Applying learner 'regr.kknn' on task 'PredictionSet[1:13]' (iter 2/10)
## INFO  [08:02:31.774] [mlr3] Applying learner 'regr.kknn' on task 'PredictionSet[1:13]' (iter 3/10)
## INFO  [08:02:31.794] [mlr3] Applying learner 'regr.kknn' on task 'PredictionSet[1:13]' (iter 4/10)
## INFO  [08:02:31.815] [mlr3] Applying learner 'regr.kknn' on task 'PredictionSet[1:13]' (iter 5/10)
## INFO  [08:02:31.836] [mlr3] Applying learner 'regr.kknn' on task 'PredictionSet[1:13]' (iter 6/10)
## INFO  [08:02:31.866] [mlr3] Applying learner 'regr.kknn' on task 'PredictionSet[1:13]' (iter 7/10)
## INFO  [08:02:31.890] [mlr3] Applying learner 'regr.kknn' on task 'PredictionSet[1:13]' (iter 8/10)
## INFO  [08:02:31.916] [mlr3] Applying learner 'regr.kknn' on task 'PredictionSet[1:13]' (iter 9/10)
## INFO  [08:02:31.938] [mlr3] Applying learner 'regr.kknn' on task 'PredictionSet[1:13]' (iter 10/10)
## INFO  [08:02:31.959] [mlr3] Finished benchmark
## INFO  [08:02:31.992] [bbotk] Result of batch 3:
## INFO  [08:02:31.993] [bbotk]   k distance     kernel regr.rmse warnings errors runtime_learners
## INFO  [08:02:31.993] [bbotk]  12 11.47143 triangular  21.55582        0      0            0.115
## INFO  [08:02:31.993] [bbotk]                                 uhash
## INFO  [08:02:31.993] [bbotk]  a458ecc3-9f42-4316-8d6e-143fb98bbc24
## INFO  [08:02:31.995] [bbotk] Evaluating 1 configuration(s)
## INFO  [08:02:32.005] [mlr3] Running benchmark with 10 resampling iterations
## INFO  [08:02:32.010] [mlr3] Applying learner 'regr.kknn' on task 'PredictionSet[1:13]' (iter 1/10)
## INFO  [08:02:32.030] [mlr3] Applying learner 'regr.kknn' on task 'PredictionSet[1:13]' (iter 2/10)
## INFO  [08:02:32.051] [mlr3] Applying learner 'regr.kknn' on task 'PredictionSet[1:13]' (iter 3/10)
## INFO  [08:02:32.073] [mlr3] Applying learner 'regr.kknn' on task 'PredictionSet[1:13]' (iter 4/10)
## INFO  [08:02:32.093] [mlr3] Applying learner 'regr.kknn' on task 'PredictionSet[1:13]' (iter 5/10)
## INFO  [08:02:32.114] [mlr3] Applying learner 'regr.kknn' on task 'PredictionSet[1:13]' (iter 6/10)
## INFO  [08:02:32.137] [mlr3] Applying learner 'regr.kknn' on task 'PredictionSet[1:13]' (iter 7/10)
## INFO  [08:02:32.157] [mlr3] Applying learner 'regr.kknn' on task 'PredictionSet[1:13]' (iter 8/10)
## INFO  [08:02:32.178] [mlr3] Applying learner 'regr.kknn' on task 'PredictionSet[1:13]' (iter 9/10)
## INFO  [08:02:32.199] [mlr3] Applying learner 'regr.kknn' on task 'PredictionSet[1:13]' (iter 10/10)
## INFO  [08:02:32.228] [mlr3] Finished benchmark
## INFO  [08:02:32.266] [bbotk] Result of batch 4:
## INFO  [08:02:32.268] [bbotk]  k distance    kernel regr.rmse warnings errors runtime_learners
## INFO  [08:02:32.268] [bbotk]  3 7.207143 triweight  19.80634        0      0            0.102
## INFO  [08:02:32.268] [bbotk]                                 uhash
## INFO  [08:02:32.268] [bbotk]  27479cf1-442d-43c7-966c-d1fd1ec303fe
## INFO  [08:02:32.270] [bbotk] Evaluating 1 configuration(s)
## INFO  [08:02:32.285] [mlr3] Running benchmark with 10 resampling iterations
## INFO  [08:02:32.291] [mlr3] Applying learner 'regr.kknn' on task 'PredictionSet[1:13]' (iter 1/10)
## INFO  [08:02:32.312] [mlr3] Applying learner 'regr.kknn' on task 'PredictionSet[1:13]' (iter 2/10)
## INFO  [08:02:32.332] [mlr3] Applying learner 'regr.kknn' on task 'PredictionSet[1:13]' (iter 3/10)
## INFO  [08:02:32.354] [mlr3] Applying learner 'regr.kknn' on task 'PredictionSet[1:13]' (iter 4/10)
## INFO  [08:02:32.379] [mlr3] Applying learner 'regr.kknn' on task 'PredictionSet[1:13]' (iter 5/10)
## INFO  [08:02:32.400] [mlr3] Applying learner 'regr.kknn' on task 'PredictionSet[1:13]' (iter 6/10)
## INFO  [08:02:32.421] [mlr3] Applying learner 'regr.kknn' on task 'PredictionSet[1:13]' (iter 7/10)
## INFO  [08:02:32.444] [mlr3] Applying learner 'regr.kknn' on task 'PredictionSet[1:13]' (iter 8/10)
## INFO  [08:02:32.464] [mlr3] Applying learner 'regr.kknn' on task 'PredictionSet[1:13]' (iter 9/10)
## INFO  [08:02:32.485] [mlr3] Applying learner 'regr.kknn' on task 'PredictionSet[1:13]' (iter 10/10)
## INFO  [08:02:32.506] [mlr3] Finished benchmark
## INFO  [08:02:32.539] [bbotk] Result of batch 5:
## INFO  [08:02:32.540] [bbotk]   k distance   kernel regr.rmse warnings errors runtime_learners
## INFO  [08:02:32.540] [bbotk]  20      0.1 biweight   28.4517        0      0            0.111
## INFO  [08:02:32.540] [bbotk]                                 uhash
## INFO  [08:02:32.540] [bbotk]  88b247a0-8161-496b-ad10-ed8cc5739ecf
## INFO  [08:02:32.542] [bbotk] Evaluating 1 configuration(s)
## INFO  [08:02:32.552] [mlr3] Running benchmark with 10 resampling iterations
## INFO  [08:02:32.557] [mlr3] Applying learner 'regr.kknn' on task 'PredictionSet[1:13]' (iter 1/10)
## INFO  [08:02:32.586] [mlr3] Applying learner 'regr.kknn' on task 'PredictionSet[1:13]' (iter 2/10)
## INFO  [08:02:32.611] [mlr3] Applying learner 'regr.kknn' on task 'PredictionSet[1:13]' (iter 3/10)
## INFO  [08:02:32.635] [mlr3] Applying learner 'regr.kknn' on task 'PredictionSet[1:13]' (iter 4/10)
## INFO  [08:02:32.659] [mlr3] Applying learner 'regr.kknn' on task 'PredictionSet[1:13]' (iter 5/10)
## INFO  [08:02:32.681] [mlr3] Applying learner 'regr.kknn' on task 'PredictionSet[1:13]' (iter 6/10)
## INFO  [08:02:32.701] [mlr3] Applying learner 'regr.kknn' on task 'PredictionSet[1:13]' (iter 7/10)
## INFO  [08:02:32.721] [mlr3] Applying learner 'regr.kknn' on task 'PredictionSet[1:13]' (iter 8/10)
## INFO  [08:02:32.744] [mlr3] Applying learner 'regr.kknn' on task 'PredictionSet[1:13]' (iter 9/10)
## INFO  [08:02:32.764] [mlr3] Applying learner 'regr.kknn' on task 'PredictionSet[1:13]' (iter 10/10)
## INFO  [08:02:32.784] [mlr3] Finished benchmark
## INFO  [08:02:32.817] [bbotk] Result of batch 6:
## INFO  [08:02:32.819] [bbotk]   k distance  kernel regr.rmse warnings errors runtime_learners
## INFO  [08:02:32.819] [bbotk]  10 8.628571 optimal  20.42672        0      0            0.113
## INFO  [08:02:32.819] [bbotk]                                 uhash
## INFO  [08:02:32.819] [bbotk]  f3aec736-0f5f-4d4b-b21d-3dbd3187c625
## INFO  [08:02:32.820] [bbotk] Evaluating 1 configuration(s)
## INFO  [08:02:32.831] [mlr3] Running benchmark with 10 resampling iterations
## INFO  [08:02:32.836] [mlr3] Applying learner 'regr.kknn' on task 'PredictionSet[1:13]' (iter 1/10)
## INFO  [08:02:32.856] [mlr3] Applying learner 'regr.kknn' on task 'PredictionSet[1:13]' (iter 2/10)
## INFO  [08:02:32.877] [mlr3] Applying learner 'regr.kknn' on task 'PredictionSet[1:13]' (iter 3/10)
## INFO  [08:02:32.899] [mlr3] Applying learner 'regr.kknn' on task 'PredictionSet[1:13]' (iter 4/10)
## INFO  [08:02:32.920] [mlr3] Applying learner 'regr.kknn' on task 'PredictionSet[1:13]' (iter 5/10)
## INFO  [08:02:32.940] [mlr3] Applying learner 'regr.kknn' on task 'PredictionSet[1:13]' (iter 6/10)
## INFO  [08:02:32.973] [mlr3] Applying learner 'regr.kknn' on task 'PredictionSet[1:13]' (iter 7/10)
## INFO  [08:02:32.998] [mlr3] Applying learner 'regr.kknn' on task 'PredictionSet[1:13]' (iter 8/10)
## INFO  [08:02:33.023] [mlr3] Applying learner 'regr.kknn' on task 'PredictionSet[1:13]' (iter 9/10)
## INFO  [08:02:33.046] [mlr3] Applying learner 'regr.kknn' on task 'PredictionSet[1:13]' (iter 10/10)
## INFO  [08:02:33.066] [mlr3] Finished benchmark
## INFO  [08:02:33.097] [bbotk] Result of batch 7:
## INFO  [08:02:33.099] [bbotk]  k distance   kernel regr.rmse warnings errors runtime_learners
## INFO  [08:02:33.099] [bbotk]  9 12.89286 biweight  19.59117        0      0            0.114
## INFO  [08:02:33.099] [bbotk]                                 uhash
## INFO  [08:02:33.099] [bbotk]  a80848b4-cb8c-4e3e-a575-39b058121695
## INFO  [08:02:33.101] [bbotk] Evaluating 1 configuration(s)
## INFO  [08:02:33.112] [mlr3] Running benchmark with 10 resampling iterations
## INFO  [08:02:33.118] [mlr3] Applying learner 'regr.kknn' on task 'PredictionSet[1:13]' (iter 1/10)
## INFO  [08:02:33.138] [mlr3] Applying learner 'regr.kknn' on task 'PredictionSet[1:13]' (iter 2/10)
## INFO  [08:02:33.158] [mlr3] Applying learner 'regr.kknn' on task 'PredictionSet[1:13]' (iter 3/10)
## INFO  [08:02:33.179] [mlr3] Applying learner 'regr.kknn' on task 'PredictionSet[1:13]' (iter 4/10)
## INFO  [08:02:33.201] [mlr3] Applying learner 'regr.kknn' on task 'PredictionSet[1:13]' (iter 5/10)
## INFO  [08:02:33.222] [mlr3] Applying learner 'regr.kknn' on task 'PredictionSet[1:13]' (iter 6/10)
## INFO  [08:02:33.242] [mlr3] Applying learner 'regr.kknn' on task 'PredictionSet[1:13]' (iter 7/10)
## INFO  [08:02:33.265] [mlr3] Applying learner 'regr.kknn' on task 'PredictionSet[1:13]' (iter 8/10)
## INFO  [08:02:33.285] [mlr3] Applying learner 'regr.kknn' on task 'PredictionSet[1:13]' (iter 9/10)
## INFO  [08:02:33.306] [mlr3] Applying learner 'regr.kknn' on task 'PredictionSet[1:13]' (iter 10/10)
## INFO  [08:02:33.338] [mlr3] Finished benchmark
## INFO  [08:02:33.377] [bbotk] Result of batch 8:
## INFO  [08:02:33.379] [bbotk]   k distance      kernel regr.rmse warnings errors runtime_learners
## INFO  [08:02:33.379] [bbotk]  12      0.1 rectangular  26.12269        0      0            0.108
## INFO  [08:02:33.379] [bbotk]                                 uhash
## INFO  [08:02:33.379] [bbotk]  ebcfd21d-e517-4d8b-b666-95687b46558b
## INFO  [08:02:33.380] [bbotk] Evaluating 1 configuration(s)
## INFO  [08:02:33.391] [mlr3] Running benchmark with 10 resampling iterations
## INFO  [08:02:33.397] [mlr3] Applying learner 'regr.kknn' on task 'PredictionSet[1:13]' (iter 1/10)
## INFO  [08:02:33.419] [mlr3] Applying learner 'regr.kknn' on task 'PredictionSet[1:13]' (iter 2/10)
## INFO  [08:02:33.440] [mlr3] Applying learner 'regr.kknn' on task 'PredictionSet[1:13]' (iter 3/10)
## INFO  [08:02:33.460] [mlr3] Applying learner 'regr.kknn' on task 'PredictionSet[1:13]' (iter 4/10)
## INFO  [08:02:33.482] [mlr3] Applying learner 'regr.kknn' on task 'PredictionSet[1:13]' (iter 5/10)
## INFO  [08:02:33.503] [mlr3] Applying learner 'regr.kknn' on task 'PredictionSet[1:13]' (iter 6/10)
## INFO  [08:02:33.524] [mlr3] Applying learner 'regr.kknn' on task 'PredictionSet[1:13]' (iter 7/10)
## INFO  [08:02:33.544] [mlr3] Applying learner 'regr.kknn' on task 'PredictionSet[1:13]' (iter 8/10)
## INFO  [08:02:33.567] [mlr3] Applying learner 'regr.kknn' on task 'PredictionSet[1:13]' (iter 9/10)
## INFO  [08:02:33.587] [mlr3] Applying learner 'regr.kknn' on task 'PredictionSet[1:13]' (iter 10/10)
## INFO  [08:02:33.607] [mlr3] Finished benchmark
## INFO  [08:02:33.641] [bbotk] Result of batch 9:
## INFO  [08:02:33.643] [bbotk]   k distance kernel regr.rmse warnings errors runtime_learners
## INFO  [08:02:33.643] [bbotk]  19 17.15714    cos  24.48112        0      0            0.105
## INFO  [08:02:33.643] [bbotk]                                 uhash
## INFO  [08:02:33.643] [bbotk]  6ad6bcde-58ef-474c-ac92-b20cce5ea485
## INFO  [08:02:33.644] [bbotk] Evaluating 1 configuration(s)
## INFO  [08:02:33.655] [mlr3] Running benchmark with 10 resampling iterations
## INFO  [08:02:33.666] [mlr3] Applying learner 'regr.kknn' on task 'PredictionSet[1:13]' (iter 1/10)
## INFO  [08:02:33.690] [mlr3] Applying learner 'regr.kknn' on task 'PredictionSet[1:13]' (iter 2/10)
## INFO  [08:02:33.716] [mlr3] Applying learner 'regr.kknn' on task 'PredictionSet[1:13]' (iter 3/10)
## INFO  [08:02:33.741] [mlr3] Applying learner 'regr.kknn' on task 'PredictionSet[1:13]' (iter 4/10)
## INFO  [08:02:33.762] [mlr3] Applying learner 'regr.kknn' on task 'PredictionSet[1:13]' (iter 5/10)
## INFO  [08:02:33.783] [mlr3] Applying learner 'regr.kknn' on task 'PredictionSet[1:13]' (iter 6/10)
## INFO  [08:02:33.805] [mlr3] Applying learner 'regr.kknn' on task 'PredictionSet[1:13]' (iter 7/10)
## INFO  [08:02:33.825] [mlr3] Applying learner 'regr.kknn' on task 'PredictionSet[1:13]' (iter 8/10)
## INFO  [08:02:33.845] [mlr3] Applying learner 'regr.kknn' on task 'PredictionSet[1:13]' (iter 9/10)
## INFO  [08:02:33.868] [mlr3] Applying learner 'regr.kknn' on task 'PredictionSet[1:13]' (iter 10/10)
## INFO  [08:02:33.888] [mlr3] Finished benchmark
## INFO  [08:02:33.919] [bbotk] Result of batch 10:
## INFO  [08:02:33.921] [bbotk]   k distance       kernel regr.rmse warnings errors runtime_learners
## INFO  [08:02:33.921] [bbotk]  19 15.73571 epanechnikov  24.76533        0      0             0.11
## INFO  [08:02:33.921] [bbotk]                                 uhash
## INFO  [08:02:33.921] [bbotk]  ad62ddb8-7859-4a99-bcad-95b3b36ffba0
## INFO  [08:02:33.927] [bbotk] Finished optimizing after 10 evaluation(s)
## INFO  [08:02:33.927] [bbotk] Result:
## INFO  [08:02:33.928] [bbotk]  k distance   kernel learner_param_vals  x_domain regr.rmse
## INFO  [08:02:33.928] [bbotk]  9 12.89286 biweight          <list[3]> <list[3]>  19.59117
## INFO  [08:02:34.160] [bbotk] Starting to optimize 2 parameter(s) with '<TunerGridSearch>' and '<TerminatorEvals> [n_evals=10, k=0]'
## INFO  [08:02:34.163] [bbotk] Evaluating 1 configuration(s)
## INFO  [08:02:34.185] [mlr3] Running benchmark with 10 resampling iterations
## INFO  [08:02:34.190] [mlr3] Applying learner 'regr.ranger' on task 'PredictionSet[1:13]' (iter 1/10)
## INFO  [08:02:34.215] [mlr3] Applying learner 'regr.ranger' on task 'PredictionSet[1:13]' (iter 2/10)
## INFO  [08:02:34.241] [mlr3] Applying learner 'regr.ranger' on task 'PredictionSet[1:13]' (iter 3/10)
## INFO  [08:02:34.265] [mlr3] Applying learner 'regr.ranger' on task 'PredictionSet[1:13]' (iter 4/10)
## INFO  [08:02:34.289] [mlr3] Applying learner 'regr.ranger' on task 'PredictionSet[1:13]' (iter 5/10)
## INFO  [08:02:34.324] [mlr3] Applying learner 'regr.ranger' on task 'PredictionSet[1:13]' (iter 6/10)
## INFO  [08:02:34.351] [mlr3] Applying learner 'regr.ranger' on task 'PredictionSet[1:13]' (iter 7/10)
## INFO  [08:02:34.379] [mlr3] Applying learner 'regr.ranger' on task 'PredictionSet[1:13]' (iter 8/10)
## INFO  [08:02:34.408] [mlr3] Applying learner 'regr.ranger' on task 'PredictionSet[1:13]' (iter 9/10)
## INFO  [08:02:34.433] [mlr3] Applying learner 'regr.ranger' on task 'PredictionSet[1:13]' (iter 10/10)
## INFO  [08:02:34.457] [mlr3] Finished benchmark
## INFO  [08:02:34.487] [bbotk] Result of batch 1:
## INFO  [08:02:34.488] [bbotk]  max.depth num.trees regr.rmse warnings errors runtime_learners
## INFO  [08:02:34.488] [bbotk]          0       132  19.90124        0      0            0.146
## INFO  [08:02:34.488] [bbotk]                                 uhash
## INFO  [08:02:34.488] [bbotk]  907a9a4a-674b-4fbf-833a-a3260ca3e06a
## INFO  [08:02:34.489] [bbotk] Evaluating 1 configuration(s)
## INFO  [08:02:34.510] [mlr3] Running benchmark with 10 resampling iterations
## INFO  [08:02:34.516] [mlr3] Applying learner 'regr.ranger' on task 'PredictionSet[1:13]' (iter 1/10)
## INFO  [08:02:34.536] [mlr3] Applying learner 'regr.ranger' on task 'PredictionSet[1:13]' (iter 2/10)
## INFO  [08:02:34.556] [mlr3] Applying learner 'regr.ranger' on task 'PredictionSet[1:13]' (iter 3/10)
## INFO  [08:02:34.574] [mlr3] Applying learner 'regr.ranger' on task 'PredictionSet[1:13]' (iter 4/10)
## INFO  [08:02:34.593] [mlr3] Applying learner 'regr.ranger' on task 'PredictionSet[1:13]' (iter 5/10)
## INFO  [08:02:34.615] [mlr3] Applying learner 'regr.ranger' on task 'PredictionSet[1:13]' (iter 6/10)
## INFO  [08:02:34.634] [mlr3] Applying learner 'regr.ranger' on task 'PredictionSet[1:13]' (iter 7/10)
## INFO  [08:02:34.653] [mlr3] Applying learner 'regr.ranger' on task 'PredictionSet[1:13]' (iter 8/10)
## INFO  [08:02:34.672] [mlr3] Applying learner 'regr.ranger' on task 'PredictionSet[1:13]' (iter 9/10)
## INFO  [08:02:34.694] [mlr3] Applying learner 'regr.ranger' on task 'PredictionSet[1:13]' (iter 10/10)
## INFO  [08:02:34.722] [mlr3] Finished benchmark
## INFO  [08:02:34.762] [bbotk] Result of batch 2:
## INFO  [08:02:34.764] [bbotk]  max.depth num.trees regr.rmse warnings errors runtime_learners
## INFO  [08:02:34.764] [bbotk]          1       185  38.90246        0      0            0.101
## INFO  [08:02:34.764] [bbotk]                                 uhash
## INFO  [08:02:34.764] [bbotk]  bda3f26a-d2ef-40b4-9344-5689f1c64497
## INFO  [08:02:34.766] [bbotk] Evaluating 1 configuration(s)
## INFO  [08:02:34.789] [mlr3] Running benchmark with 10 resampling iterations
## INFO  [08:02:34.794] [mlr3] Applying learner 'regr.ranger' on task 'PredictionSet[1:13]' (iter 1/10)
## INFO  [08:02:34.808] [mlr3] Applying learner 'regr.ranger' on task 'PredictionSet[1:13]' (iter 2/10)
## INFO  [08:02:34.823] [mlr3] Applying learner 'regr.ranger' on task 'PredictionSet[1:13]' (iter 3/10)
## INFO  [08:02:34.840] [mlr3] Applying learner 'regr.ranger' on task 'PredictionSet[1:13]' (iter 4/10)
## INFO  [08:02:34.855] [mlr3] Applying learner 'regr.ranger' on task 'PredictionSet[1:13]' (iter 5/10)
## INFO  [08:02:34.870] [mlr3] Applying learner 'regr.ranger' on task 'PredictionSet[1:13]' (iter 6/10)
## INFO  [08:02:34.884] [mlr3] Applying learner 'regr.ranger' on task 'PredictionSet[1:13]' (iter 7/10)
## INFO  [08:02:34.899] [mlr3] Applying learner 'regr.ranger' on task 'PredictionSet[1:13]' (iter 8/10)
## INFO  [08:02:34.916] [mlr3] Applying learner 'regr.ranger' on task 'PredictionSet[1:13]' (iter 9/10)
## INFO  [08:02:34.932] [mlr3] Applying learner 'regr.ranger' on task 'PredictionSet[1:13]' (iter 10/10)
## INFO  [08:02:34.946] [mlr3] Finished benchmark
## INFO  [08:02:34.979] [bbotk] Result of batch 3:
## INFO  [08:02:34.981] [bbotk]  max.depth num.trees regr.rmse warnings errors runtime_learners
## INFO  [08:02:34.981] [bbotk]          6         1  33.76491        0      0            0.051
## INFO  [08:02:34.981] [bbotk]                                 uhash
## INFO  [08:02:34.981] [bbotk]  0b16e2d8-7342-4520-a664-27b55e2d972e
## INFO  [08:02:34.983] [bbotk] Evaluating 1 configuration(s)
## INFO  [08:02:35.043] [mlr3] Running benchmark with 10 resampling iterations
## INFO  [08:02:35.050] [mlr3] Applying learner 'regr.ranger' on task 'PredictionSet[1:13]' (iter 1/10)
## INFO  [08:02:35.095] [mlr3] Applying learner 'regr.ranger' on task 'PredictionSet[1:13]' (iter 2/10)
## INFO  [08:02:35.137] [mlr3] Applying learner 'regr.ranger' on task 'PredictionSet[1:13]' (iter 3/10)
## INFO  [08:02:35.178] [mlr3] Applying learner 'regr.ranger' on task 'PredictionSet[1:13]' (iter 4/10)
## INFO  [08:02:35.219] [mlr3] Applying learner 'regr.ranger' on task 'PredictionSet[1:13]' (iter 5/10)
## INFO  [08:02:35.256] [mlr3] Applying learner 'regr.ranger' on task 'PredictionSet[1:13]' (iter 6/10)
## INFO  [08:02:35.295] [mlr3] Applying learner 'regr.ranger' on task 'PredictionSet[1:13]' (iter 7/10)
## INFO  [08:02:35.332] [mlr3] Applying learner 'regr.ranger' on task 'PredictionSet[1:13]' (iter 8/10)
## INFO  [08:02:35.371] [mlr3] Applying learner 'regr.ranger' on task 'PredictionSet[1:13]' (iter 9/10)
## INFO  [08:02:35.407] [mlr3] Applying learner 'regr.ranger' on task 'PredictionSet[1:13]' (iter 10/10)
## INFO  [08:02:35.445] [mlr3] Finished benchmark
## INFO  [08:02:35.476] [bbotk] Result of batch 4:
## INFO  [08:02:35.478] [bbotk]  max.depth num.trees regr.rmse warnings errors runtime_learners
## INFO  [08:02:35.478] [bbotk]          0       316  19.85887        0      0            0.279
## INFO  [08:02:35.478] [bbotk]                                 uhash
## INFO  [08:02:35.478] [bbotk]  656ffaab-f533-47d0-89ba-c0e92699164f
## INFO  [08:02:35.479] [bbotk] Evaluating 1 configuration(s)
## INFO  [08:02:35.511] [mlr3] Running benchmark with 10 resampling iterations
## INFO  [08:02:35.517] [mlr3] Applying learner 'regr.ranger' on task 'PredictionSet[1:13]' (iter 1/10)
## INFO  [08:02:35.560] [mlr3] Applying learner 'regr.ranger' on task 'PredictionSet[1:13]' (iter 2/10)
## INFO  [08:02:35.603] [mlr3] Applying learner 'regr.ranger' on task 'PredictionSet[1:13]' (iter 3/10)
## INFO  [08:02:35.644] [mlr3] Applying learner 'regr.ranger' on task 'PredictionSet[1:13]' (iter 4/10)
## INFO  [08:02:35.687] [mlr3] Applying learner 'regr.ranger' on task 'PredictionSet[1:13]' (iter 5/10)
## INFO  [08:02:35.727] [mlr3] Applying learner 'regr.ranger' on task 'PredictionSet[1:13]' (iter 6/10)
## INFO  [08:02:35.767] [mlr3] Applying learner 'regr.ranger' on task 'PredictionSet[1:13]' (iter 7/10)
## INFO  [08:02:35.807] [mlr3] Applying learner 'regr.ranger' on task 'PredictionSet[1:13]' (iter 8/10)
## INFO  [08:02:35.847] [mlr3] Applying learner 'regr.ranger' on task 'PredictionSet[1:13]' (iter 9/10)
## INFO  [08:02:35.887] [mlr3] Applying learner 'regr.ranger' on task 'PredictionSet[1:13]' (iter 10/10)
## INFO  [08:02:35.926] [mlr3] Finished benchmark
## INFO  [08:02:35.958] [bbotk] Result of batch 5:
## INFO  [08:02:35.959] [bbotk]  max.depth num.trees regr.rmse warnings errors runtime_learners
## INFO  [08:02:35.959] [bbotk]          9       343  20.08087        0      0            0.293
## INFO  [08:02:35.959] [bbotk]                                 uhash
## INFO  [08:02:35.959] [bbotk]  394210e8-63ba-4c79-ab84-74fccfa0b550
## INFO  [08:02:35.961] [bbotk] Evaluating 1 configuration(s)
## INFO  [08:02:35.983] [mlr3] Running benchmark with 10 resampling iterations
## INFO  [08:02:35.988] [mlr3] Applying learner 'regr.ranger' on task 'PredictionSet[1:13]' (iter 1/10)
## INFO  [08:02:36.006] [mlr3] Applying learner 'regr.ranger' on task 'PredictionSet[1:13]' (iter 2/10)
## INFO  [08:02:36.023] [mlr3] Applying learner 'regr.ranger' on task 'PredictionSet[1:13]' (iter 3/10)
## INFO  [08:02:36.042] [mlr3] Applying learner 'regr.ranger' on task 'PredictionSet[1:13]' (iter 4/10)
## INFO  [08:02:36.059] [mlr3] Applying learner 'regr.ranger' on task 'PredictionSet[1:13]' (iter 5/10)
## INFO  [08:02:36.077] [mlr3] Applying learner 'regr.ranger' on task 'PredictionSet[1:13]' (iter 6/10)
## INFO  [08:02:36.094] [mlr3] Applying learner 'regr.ranger' on task 'PredictionSet[1:13]' (iter 7/10)
## INFO  [08:02:36.126] [mlr3] Applying learner 'regr.ranger' on task 'PredictionSet[1:13]' (iter 8/10)
## INFO  [08:02:36.147] [mlr3] Applying learner 'regr.ranger' on task 'PredictionSet[1:13]' (iter 9/10)
## INFO  [08:02:36.167] [mlr3] Applying learner 'regr.ranger' on task 'PredictionSet[1:13]' (iter 10/10)
## INFO  [08:02:36.190] [mlr3] Finished benchmark
## INFO  [08:02:36.226] [bbotk] Result of batch 6:
## INFO  [08:02:36.228] [bbotk]  max.depth num.trees regr.rmse warnings errors runtime_learners
## INFO  [08:02:36.228] [bbotk]          2        79  32.17157        0      0            0.088
## INFO  [08:02:36.228] [bbotk]                                 uhash
## INFO  [08:02:36.228] [bbotk]  0be18bbc-ac6e-4aea-b397-bf4033aff353
## INFO  [08:02:36.229] [bbotk] Evaluating 1 configuration(s)
## INFO  [08:02:36.251] [mlr3] Running benchmark with 10 resampling iterations
## INFO  [08:02:36.257] [mlr3] Applying learner 'regr.ranger' on task 'PredictionSet[1:13]' (iter 1/10)
## INFO  [08:02:36.284] [mlr3] Applying learner 'regr.ranger' on task 'PredictionSet[1:13]' (iter 2/10)
## INFO  [08:02:36.310] [mlr3] Applying learner 'regr.ranger' on task 'PredictionSet[1:13]' (iter 3/10)
## INFO  [08:02:36.337] [mlr3] Applying learner 'regr.ranger' on task 'PredictionSet[1:13]' (iter 4/10)
## INFO  [08:02:36.364] [mlr3] Applying learner 'regr.ranger' on task 'PredictionSet[1:13]' (iter 5/10)
## INFO  [08:02:36.390] [mlr3] Applying learner 'regr.ranger' on task 'PredictionSet[1:13]' (iter 6/10)
## INFO  [08:02:36.417] [mlr3] Applying learner 'regr.ranger' on task 'PredictionSet[1:13]' (iter 7/10)
## INFO  [08:02:36.443] [mlr3] Applying learner 'regr.ranger' on task 'PredictionSet[1:13]' (iter 8/10)
## INFO  [08:02:36.469] [mlr3] Applying learner 'regr.ranger' on task 'PredictionSet[1:13]' (iter 9/10)
## INFO  [08:02:36.498] [mlr3] Applying learner 'regr.ranger' on task 'PredictionSet[1:13]' (iter 10/10)
## INFO  [08:02:36.523] [mlr3] Finished benchmark
## INFO  [08:02:36.555] [bbotk] Result of batch 7:
## INFO  [08:02:36.557] [bbotk]  max.depth num.trees regr.rmse warnings errors runtime_learners
## INFO  [08:02:36.557] [bbotk]         13       158  20.12065        0      0            0.162
## INFO  [08:02:36.557] [bbotk]                                 uhash
## INFO  [08:02:36.557] [bbotk]  e3087bd6-092e-464a-8537-ef6023640790
## INFO  [08:02:36.559] [bbotk] Evaluating 1 configuration(s)
## INFO  [08:02:36.594] [mlr3] Running benchmark with 10 resampling iterations
## INFO  [08:02:36.600] [mlr3] Applying learner 'regr.ranger' on task 'PredictionSet[1:13]' (iter 1/10)
## INFO  [08:02:36.618] [mlr3] Applying learner 'regr.ranger' on task 'PredictionSet[1:13]' (iter 2/10)
## INFO  [08:02:36.638] [mlr3] Applying learner 'regr.ranger' on task 'PredictionSet[1:13]' (iter 3/10)
## INFO  [08:02:36.656] [mlr3] Applying learner 'regr.ranger' on task 'PredictionSet[1:13]' (iter 4/10)
## INFO  [08:02:36.672] [mlr3] Applying learner 'regr.ranger' on task 'PredictionSet[1:13]' (iter 5/10)
## INFO  [08:02:36.688] [mlr3] Applying learner 'regr.ranger' on task 'PredictionSet[1:13]' (iter 6/10)
## INFO  [08:02:36.704] [mlr3] Applying learner 'regr.ranger' on task 'PredictionSet[1:13]' (iter 7/10)
## INFO  [08:02:36.721] [mlr3] Applying learner 'regr.ranger' on task 'PredictionSet[1:13]' (iter 8/10)
## INFO  [08:02:36.736] [mlr3] Applying learner 'regr.ranger' on task 'PredictionSet[1:13]' (iter 9/10)
## INFO  [08:02:36.751] [mlr3] Applying learner 'regr.ranger' on task 'PredictionSet[1:13]' (iter 10/10)
## INFO  [08:02:36.765] [mlr3] Finished benchmark
## INFO  [08:02:36.799] [bbotk] Result of batch 8:
## INFO  [08:02:36.800] [bbotk]  max.depth num.trees regr.rmse warnings errors runtime_learners
## INFO  [08:02:36.800] [bbotk]         13         1  29.31267        0      0            0.052
## INFO  [08:02:36.800] [bbotk]                                 uhash
## INFO  [08:02:36.800] [bbotk]  dd94eb2f-4649-4767-aa24-a7607e041770
## INFO  [08:02:36.802] [bbotk] Evaluating 1 configuration(s)
## INFO  [08:02:36.824] [mlr3] Running benchmark with 10 resampling iterations
## INFO  [08:02:36.829] [mlr3] Applying learner 'regr.ranger' on task 'PredictionSet[1:13]' (iter 1/10)
## INFO  [08:02:36.875] [mlr3] Applying learner 'regr.ranger' on task 'PredictionSet[1:13]' (iter 2/10)
## INFO  [08:02:36.919] [mlr3] Applying learner 'regr.ranger' on task 'PredictionSet[1:13]' (iter 3/10)
## INFO  [08:02:36.965] [mlr3] Applying learner 'regr.ranger' on task 'PredictionSet[1:13]' (iter 4/10)
## INFO  [08:02:37.010] [mlr3] Applying learner 'regr.ranger' on task 'PredictionSet[1:13]' (iter 5/10)
## INFO  [08:02:37.064] [mlr3] Applying learner 'regr.ranger' on task 'PredictionSet[1:13]' (iter 6/10)
## INFO  [08:02:37.114] [mlr3] Applying learner 'regr.ranger' on task 'PredictionSet[1:13]' (iter 7/10)
## INFO  [08:02:37.164] [mlr3] Applying learner 'regr.ranger' on task 'PredictionSet[1:13]' (iter 8/10)
## INFO  [08:02:37.212] [mlr3] Applying learner 'regr.ranger' on task 'PredictionSet[1:13]' (iter 9/10)
## INFO  [08:02:37.261] [mlr3] Applying learner 'regr.ranger' on task 'PredictionSet[1:13]' (iter 10/10)
## INFO  [08:02:37.307] [mlr3] Finished benchmark
## INFO  [08:02:37.340] [bbotk] Result of batch 9:
## INFO  [08:02:37.342] [bbotk]  max.depth num.trees regr.rmse warnings errors runtime_learners
## INFO  [08:02:37.342] [bbotk]         11       422  19.94941        0      0            0.356
## INFO  [08:02:37.342] [bbotk]                                 uhash
## INFO  [08:02:37.342] [bbotk]  62e587e7-521e-4535-b9a7-89d17ae0bce6
## INFO  [08:02:37.343] [bbotk] Evaluating 1 configuration(s)
## INFO  [08:02:37.364] [mlr3] Running benchmark with 10 resampling iterations
## INFO  [08:02:37.369] [mlr3] Applying learner 'regr.ranger' on task 'PredictionSet[1:13]' (iter 1/10)
## INFO  [08:02:37.409] [mlr3] Applying learner 'regr.ranger' on task 'PredictionSet[1:13]' (iter 2/10)
## INFO  [08:02:37.447] [mlr3] Applying learner 'regr.ranger' on task 'PredictionSet[1:13]' (iter 3/10)
## INFO  [08:02:37.486] [mlr3] Applying learner 'regr.ranger' on task 'PredictionSet[1:13]' (iter 4/10)
## INFO  [08:02:37.525] [mlr3] Applying learner 'regr.ranger' on task 'PredictionSet[1:13]' (iter 5/10)
## INFO  [08:02:37.564] [mlr3] Applying learner 'regr.ranger' on task 'PredictionSet[1:13]' (iter 6/10)
## INFO  [08:02:37.603] [mlr3] Applying learner 'regr.ranger' on task 'PredictionSet[1:13]' (iter 7/10)
## INFO  [08:02:37.642] [mlr3] Applying learner 'regr.ranger' on task 'PredictionSet[1:13]' (iter 8/10)
## INFO  [08:02:37.681] [mlr3] Applying learner 'regr.ranger' on task 'PredictionSet[1:13]' (iter 9/10)
## INFO  [08:02:37.732] [mlr3] Applying learner 'regr.ranger' on task 'PredictionSet[1:13]' (iter 10/10)
## INFO  [08:02:37.775] [mlr3] Finished benchmark
## INFO  [08:02:37.814] [bbotk] Result of batch 10:
## INFO  [08:02:37.816] [bbotk]  max.depth num.trees regr.rmse warnings errors runtime_learners
## INFO  [08:02:37.816] [bbotk]          5       422  21.59584        0      0            0.294
## INFO  [08:02:37.816] [bbotk]                                 uhash
## INFO  [08:02:37.816] [bbotk]  02542bd3-7dc6-4070-87d3-d469b66142df
## INFO  [08:02:37.821] [bbotk] Finished optimizing after 10 evaluation(s)
## INFO  [08:02:37.823] [bbotk] Result:
## INFO  [08:02:37.824] [bbotk]  max.depth num.trees learner_param_vals  x_domain regr.rmse
## INFO  [08:02:37.824] [bbotk]          0       316          <list[3]> <list[2]>  19.85887
## INFO  [08:02:38.072] [bbotk] Starting to optimize 5 parameter(s) with '<TunerGridSearch>' and '<TerminatorEvals> [n_evals=10, k=0]'
## INFO  [08:02:38.080] [bbotk] Evaluating 1 configuration(s)
## INFO  [08:02:38.100] [mlr3] Running benchmark with 10 resampling iterations
## INFO  [08:02:38.107] [mlr3] Applying learner 'regr.svm' on task 'PredictionSet[1:13]' (iter 1/10)
## INFO  [08:02:38.129] [mlr3] Applying learner 'regr.svm' on task 'PredictionSet[1:13]' (iter 2/10)
## INFO  [08:02:38.151] [mlr3] Applying learner 'regr.svm' on task 'PredictionSet[1:13]' (iter 3/10)
## INFO  [08:02:38.170] [mlr3] Applying learner 'regr.svm' on task 'PredictionSet[1:13]' (iter 4/10)
## INFO  [08:02:38.188] [mlr3] Applying learner 'regr.svm' on task 'PredictionSet[1:13]' (iter 5/10)
## INFO  [08:02:38.208] [mlr3] Applying learner 'regr.svm' on task 'PredictionSet[1:13]' (iter 6/10)
## INFO  [08:02:38.227] [mlr3] Applying learner 'regr.svm' on task 'PredictionSet[1:13]' (iter 7/10)
## INFO  [08:02:38.246] [mlr3] Applying learner 'regr.svm' on task 'PredictionSet[1:13]' (iter 8/10)
## INFO  [08:02:38.264] [mlr3] Applying learner 'regr.svm' on task 'PredictionSet[1:13]' (iter 9/10)
## INFO  [08:02:38.283] [mlr3] Applying learner 'regr.svm' on task 'PredictionSet[1:13]' (iter 10/10)
## INFO  [08:02:38.302] [mlr3] Finished benchmark
## INFO  [08:02:38.332] [bbotk] Result of batch 1:
## INFO  [08:02:38.334] [bbotk]  tolerance    gamma   epsilon           type  kernel regr.rmse warnings errors
## INFO  [08:02:38.334] [bbotk]  0.5789286 21.42914 0.3857143 eps-regression sigmoid  657.1984        0      0
## INFO  [08:02:38.334] [bbotk]  runtime_learners                                uhash
## INFO  [08:02:38.334] [bbotk]             0.086 ad1e9e6f-1035-461c-b8cd-4ae092fd170f
## INFO  [08:02:38.335] [bbotk] Evaluating 1 configuration(s)
## INFO  [08:02:38.351] [mlr3] Running benchmark with 10 resampling iterations
## INFO  [08:02:38.357] [mlr3] Applying learner 'regr.svm' on task 'PredictionSet[1:13]' (iter 1/10)
## INFO  [08:02:38.376] [mlr3] Applying learner 'regr.svm' on task 'PredictionSet[1:13]' (iter 2/10)
## INFO  [08:02:38.404] [mlr3] Applying learner 'regr.svm' on task 'PredictionSet[1:13]' (iter 3/10)
## INFO  [08:02:38.426] [mlr3] Applying learner 'regr.svm' on task 'PredictionSet[1:13]' (iter 4/10)
## INFO  [08:02:38.450] [mlr3] Applying learner 'regr.svm' on task 'PredictionSet[1:13]' (iter 5/10)
## INFO  [08:02:38.471] [mlr3] Applying learner 'regr.svm' on task 'PredictionSet[1:13]' (iter 6/10)
## INFO  [08:02:38.493] [mlr3] Applying learner 'regr.svm' on task 'PredictionSet[1:13]' (iter 7/10)
## INFO  [08:02:38.514] [mlr3] Applying learner 'regr.svm' on task 'PredictionSet[1:13]' (iter 8/10)
## INFO  [08:02:38.533] [mlr3] Applying learner 'regr.svm' on task 'PredictionSet[1:13]' (iter 9/10)
## INFO  [08:02:38.551] [mlr3] Applying learner 'regr.svm' on task 'PredictionSet[1:13]' (iter 10/10)
## INFO  [08:02:38.569] [mlr3] Finished benchmark
## INFO  [08:02:38.601] [bbotk] Result of batch 2:
## INFO  [08:02:38.603] [bbotk]  tolerance    gamma   epsilon           type  kernel regr.rmse warnings errors
## INFO  [08:02:38.603] [bbotk]  0.3862857 42.85729 0.4428571 eps-regression sigmoid  649.7602        0      0
## INFO  [08:02:38.603] [bbotk]  runtime_learners                                uhash
## INFO  [08:02:38.603] [bbotk]             0.095 c8bf45ef-a561-4147-ba01-a8e724014eca
## INFO  [08:02:38.605] [bbotk] Evaluating 1 configuration(s)
## INFO  [08:02:38.620] [mlr3] Running benchmark with 10 resampling iterations
## INFO  [08:02:38.625] [mlr3] Applying learner 'regr.svm' on task 'PredictionSet[1:13]' (iter 1/10)
## INFO  [08:02:38.643] [mlr3] Applying learner 'regr.svm' on task 'PredictionSet[1:13]' (iter 2/10)
## INFO  [08:02:38.664] [mlr3] Applying learner 'regr.svm' on task 'PredictionSet[1:13]' (iter 3/10)
## INFO  [08:02:38.682] [mlr3] Applying learner 'regr.svm' on task 'PredictionSet[1:13]' (iter 4/10)
## INFO  [08:02:38.708] [mlr3] Applying learner 'regr.svm' on task 'PredictionSet[1:13]' (iter 5/10)
## INFO  [08:02:38.731] [mlr3] Applying learner 'regr.svm' on task 'PredictionSet[1:13]' (iter 6/10)
## INFO  [08:02:38.754] [mlr3] Applying learner 'regr.svm' on task 'PredictionSet[1:13]' (iter 7/10)
## INFO  [08:02:38.775] [mlr3] Applying learner 'regr.svm' on task 'PredictionSet[1:13]' (iter 8/10)
## INFO  [08:02:38.797] [mlr3] Applying learner 'regr.svm' on task 'PredictionSet[1:13]' (iter 9/10)
## INFO  [08:02:38.818] [mlr3] Applying learner 'regr.svm' on task 'PredictionSet[1:13]' (iter 10/10)
## INFO  [08:02:38.837] [mlr3] Finished benchmark
## INFO  [08:02:38.868] [bbotk] Result of batch 3:
## INFO  [08:02:38.870] [bbotk]  tolerance    gamma   epsilon           type  kernel regr.rmse warnings errors
## INFO  [08:02:38.870] [bbotk]        0.9 39.28593 0.4428571 eps-regression sigmoid   654.278        0      0
## INFO  [08:02:38.870] [bbotk]  runtime_learners                                uhash
## INFO  [08:02:38.870] [bbotk]             0.099 8710c0e0-8d79-448b-b469-0ed00511eb27
## INFO  [08:02:38.871] [bbotk] Evaluating 1 configuration(s)
## INFO  [08:02:38.888] [mlr3] Running benchmark with 10 resampling iterations
## INFO  [08:02:38.893] [mlr3] Applying learner 'regr.svm' on task 'PredictionSet[1:13]' (iter 1/10)
## INFO  [08:02:38.912] [mlr3] Applying learner 'regr.svm' on task 'PredictionSet[1:13]' (iter 2/10)
## INFO  [08:02:38.930] [mlr3] Applying learner 'regr.svm' on task 'PredictionSet[1:13]' (iter 3/10)
## INFO  [08:02:38.949] [mlr3] Applying learner 'regr.svm' on task 'PredictionSet[1:13]' (iter 4/10)
## INFO  [08:02:38.969] [mlr3] Applying learner 'regr.svm' on task 'PredictionSet[1:13]' (iter 5/10)
## INFO  [08:02:38.987] [mlr3] Applying learner 'regr.svm' on task 'PredictionSet[1:13]' (iter 6/10)
## INFO  [08:02:39.014] [mlr3] Applying learner 'regr.svm' on task 'PredictionSet[1:13]' (iter 7/10)
## INFO  [08:02:39.038] [mlr3] Applying learner 'regr.svm' on task 'PredictionSet[1:13]' (iter 8/10)
## INFO  [08:02:39.060] [mlr3] Applying learner 'regr.svm' on task 'PredictionSet[1:13]' (iter 9/10)
## INFO  [08:02:39.082] [mlr3] Applying learner 'regr.svm' on task 'PredictionSet[1:13]' (iter 10/10)
## INFO  [08:02:39.104] [mlr3] Finished benchmark
## INFO  [08:02:39.138] [bbotk] Result of batch 4:
## INFO  [08:02:39.140] [bbotk]  tolerance    gamma epsilon           type  kernel regr.rmse warnings errors
## INFO  [08:02:39.140] [bbotk]  0.2578571 21.42914     0.5 eps-regression sigmoid  656.3068        0      0
## INFO  [08:02:39.140] [bbotk]  runtime_learners                                uhash
## INFO  [08:02:39.140] [bbotk]              0.09 28e1a1ad-a459-4d74-88c0-687219107dd2
## INFO  [08:02:39.141] [bbotk] Evaluating 1 configuration(s)
## INFO  [08:02:39.157] [mlr3] Running benchmark with 10 resampling iterations
## INFO  [08:02:39.162] [mlr3] Applying learner 'regr.svm' on task 'PredictionSet[1:13]' (iter 1/10)
## INFO  [08:02:39.178] [mlr3] Applying learner 'regr.svm' on task 'PredictionSet[1:13]' (iter 2/10)
## INFO  [08:02:39.195] [mlr3] Applying learner 'regr.svm' on task 'PredictionSet[1:13]' (iter 3/10)
## INFO  [08:02:39.210] [mlr3] Applying learner 'regr.svm' on task 'PredictionSet[1:13]' (iter 4/10)
## INFO  [08:02:39.226] [mlr3] Applying learner 'regr.svm' on task 'PredictionSet[1:13]' (iter 5/10)
## INFO  [08:02:39.241] [mlr3] Applying learner 'regr.svm' on task 'PredictionSet[1:13]' (iter 6/10)
## INFO  [08:02:39.258] [mlr3] Applying learner 'regr.svm' on task 'PredictionSet[1:13]' (iter 7/10)
## INFO  [08:02:39.275] [mlr3] Applying learner 'regr.svm' on task 'PredictionSet[1:13]' (iter 8/10)
## INFO  [08:02:39.299] [mlr3] Applying learner 'regr.svm' on task 'PredictionSet[1:13]' (iter 9/10)
## INFO  [08:02:39.318] [mlr3] Applying learner 'regr.svm' on task 'PredictionSet[1:13]' (iter 10/10)
## INFO  [08:02:39.338] [mlr3] Finished benchmark
## INFO  [08:02:39.377] [bbotk] Result of batch 5:
## INFO  [08:02:39.379] [bbotk]  tolerance gamma   epsilon           type kernel regr.rmse warnings errors
## INFO  [08:02:39.379] [bbotk]  0.7073571 0.001 0.6142857 eps-regression radial  18.64818        0      0
## INFO  [08:02:39.379] [bbotk]  runtime_learners                                uhash
## INFO  [08:02:39.379] [bbotk]             0.066 db2cc61f-1714-4ddd-a934-007d815c5b23
## INFO  [08:02:39.380] [bbotk] Evaluating 1 configuration(s)
## INFO  [08:02:39.396] [mlr3] Running benchmark with 10 resampling iterations
## INFO  [08:02:39.402] [mlr3] Applying learner 'regr.svm' on task 'PredictionSet[1:13]' (iter 1/10)
## INFO  [08:02:39.419] [mlr3] Applying learner 'regr.svm' on task 'PredictionSet[1:13]' (iter 2/10)
## INFO  [08:02:39.434] [mlr3] Applying learner 'regr.svm' on task 'PredictionSet[1:13]' (iter 3/10)
## INFO  [08:02:39.449] [mlr3] Applying learner 'regr.svm' on task 'PredictionSet[1:13]' (iter 4/10)
## INFO  [08:02:39.464] [mlr3] Applying learner 'regr.svm' on task 'PredictionSet[1:13]' (iter 5/10)
## INFO  [08:02:39.480] [mlr3] Applying learner 'regr.svm' on task 'PredictionSet[1:13]' (iter 6/10)
## INFO  [08:02:39.497] [mlr3] Applying learner 'regr.svm' on task 'PredictionSet[1:13]' (iter 7/10)
## INFO  [08:02:39.513] [mlr3] Applying learner 'regr.svm' on task 'PredictionSet[1:13]' (iter 8/10)
## INFO  [08:02:39.528] [mlr3] Applying learner 'regr.svm' on task 'PredictionSet[1:13]' (iter 9/10)
## INFO  [08:02:39.543] [mlr3] Applying learner 'regr.svm' on task 'PredictionSet[1:13]' (iter 10/10)
## INFO  [08:02:39.569] [mlr3] Finished benchmark
## INFO  [08:02:39.607] [bbotk] Result of batch 6:
## INFO  [08:02:39.610] [bbotk]  tolerance    gamma epsilon           type kernel regr.rmse warnings errors
## INFO  [08:02:39.610] [bbotk]  0.7073571 28.57186     0.9 eps-regression radial   20.7955        0      0
## INFO  [08:02:39.610] [bbotk]  runtime_learners                                uhash
## INFO  [08:02:39.610] [bbotk]             0.066 4bc622cd-a87a-4bcc-9166-a870417f4443
## INFO  [08:02:39.611] [bbotk] Evaluating 1 configuration(s)
## INFO  [08:02:39.633] [mlr3] Running benchmark with 10 resampling iterations
## INFO  [08:02:39.639] [mlr3] Applying learner 'regr.svm' on task 'PredictionSet[1:13]' (iter 1/10)
## INFO  [08:02:39.659] [mlr3] Applying learner 'regr.svm' on task 'PredictionSet[1:13]' (iter 2/10)
## INFO  [08:02:39.677] [mlr3] Applying learner 'regr.svm' on task 'PredictionSet[1:13]' (iter 3/10)
## INFO  [08:02:39.695] [mlr3] Applying learner 'regr.svm' on task 'PredictionSet[1:13]' (iter 4/10)
## INFO  [08:02:39.716] [mlr3] Applying learner 'regr.svm' on task 'PredictionSet[1:13]' (iter 5/10)
## INFO  [08:02:39.734] [mlr3] Applying learner 'regr.svm' on task 'PredictionSet[1:13]' (iter 6/10)
## INFO  [08:02:39.752] [mlr3] Applying learner 'regr.svm' on task 'PredictionSet[1:13]' (iter 7/10)
## INFO  [08:02:39.770] [mlr3] Applying learner 'regr.svm' on task 'PredictionSet[1:13]' (iter 8/10)
## INFO  [08:02:39.790] [mlr3] Applying learner 'regr.svm' on task 'PredictionSet[1:13]' (iter 9/10)
## INFO  [08:02:39.809] [mlr3] Applying learner 'regr.svm' on task 'PredictionSet[1:13]' (iter 10/10)
## INFO  [08:02:39.858] [mlr3] Finished benchmark
## INFO  [08:02:39.900] [bbotk] Result of batch 7:
## INFO  [08:02:39.902] [bbotk]  tolerance    gamma epsilon           type  kernel regr.rmse warnings errors
## INFO  [08:02:39.902] [bbotk]  0.8357857 28.57186     0.9 eps-regression sigmoid  641.4867        0      0
## INFO  [08:02:39.902] [bbotk]  runtime_learners                                uhash
## INFO  [08:02:39.902] [bbotk]              0.11 5ca11013-0c55-43e1-b213-97b18d27b778
## INFO  [08:02:39.904] [bbotk] Evaluating 1 configuration(s)
## INFO  [08:02:39.925] [mlr3] Running benchmark with 10 resampling iterations
## INFO  [08:02:39.931] [mlr3] Applying learner 'regr.svm' on task 'PredictionSet[1:13]' (iter 1/10)
## INFO  [08:02:39.950] [mlr3] Applying learner 'regr.svm' on task 'PredictionSet[1:13]' (iter 2/10)
## INFO  [08:02:39.967] [mlr3] Applying learner 'regr.svm' on task 'PredictionSet[1:13]' (iter 3/10)
## INFO  [08:02:39.983] [mlr3] Applying learner 'regr.svm' on task 'PredictionSet[1:13]' (iter 4/10)
## INFO  [08:02:39.999] [mlr3] Applying learner 'regr.svm' on task 'PredictionSet[1:13]' (iter 5/10)
## INFO  [08:02:40.016] [mlr3] Applying learner 'regr.svm' on task 'PredictionSet[1:13]' (iter 6/10)
## INFO  [08:02:40.032] [mlr3] Applying learner 'regr.svm' on task 'PredictionSet[1:13]' (iter 7/10)
## INFO  [08:02:40.048] [mlr3] Applying learner 'regr.svm' on task 'PredictionSet[1:13]' (iter 8/10)
## INFO  [08:02:40.063] [mlr3] Applying learner 'regr.svm' on task 'PredictionSet[1:13]' (iter 9/10)
## INFO  [08:02:40.079] [mlr3] Applying learner 'regr.svm' on task 'PredictionSet[1:13]' (iter 10/10)
## INFO  [08:02:40.096] [mlr3] Finished benchmark
## INFO  [08:02:40.128] [bbotk] Result of batch 8:
## INFO  [08:02:40.130] [bbotk]  tolerance   gamma   epsilon           type kernel regr.rmse warnings errors
## INFO  [08:02:40.130] [bbotk]  0.6431429 25.0005 0.6142857 eps-regression radial  17.30811        0      0
## INFO  [08:02:40.130] [bbotk]  runtime_learners                                uhash
## INFO  [08:02:40.130] [bbotk]             0.059 43936e13-e733-4b0a-8a2c-ec7cc75d6a64
## INFO  [08:02:40.131] [bbotk] Evaluating 1 configuration(s)
## INFO  [08:02:40.160] [mlr3] Running benchmark with 10 resampling iterations
## INFO  [08:02:40.166] [mlr3] Applying learner 'regr.svm' on task 'PredictionSet[1:13]' (iter 1/10)
## INFO  [08:02:40.185] [mlr3] Applying learner 'regr.svm' on task 'PredictionSet[1:13]' (iter 2/10)
## INFO  [08:02:40.204] [mlr3] Applying learner 'regr.svm' on task 'PredictionSet[1:13]' (iter 3/10)
## INFO  [08:02:40.222] [mlr3] Applying learner 'regr.svm' on task 'PredictionSet[1:13]' (iter 4/10)
## INFO  [08:02:40.243] [mlr3] Applying learner 'regr.svm' on task 'PredictionSet[1:13]' (iter 5/10)
## INFO  [08:02:40.259] [mlr3] Applying learner 'regr.svm' on task 'PredictionSet[1:13]' (iter 6/10)
## INFO  [08:02:40.275] [mlr3] Applying learner 'regr.svm' on task 'PredictionSet[1:13]' (iter 7/10)
## INFO  [08:02:40.290] [mlr3] Applying learner 'regr.svm' on task 'PredictionSet[1:13]' (iter 8/10)
## INFO  [08:02:40.306] [mlr3] Applying learner 'regr.svm' on task 'PredictionSet[1:13]' (iter 9/10)
## INFO  [08:02:40.323] [mlr3] Applying learner 'regr.svm' on task 'PredictionSet[1:13]' (iter 10/10)
## INFO  [08:02:40.338] [mlr3] Finished benchmark
## INFO  [08:02:40.369] [bbotk] Result of batch 9:
## INFO  [08:02:40.371] [bbotk]  tolerance    gamma   epsilon           type kernel regr.rmse warnings errors
## INFO  [08:02:40.371] [bbotk]  0.7073571 42.85729 0.8428571 eps-regression radial  20.25093        0      0
## INFO  [08:02:40.371] [bbotk]  runtime_learners                                uhash
## INFO  [08:02:40.371] [bbotk]              0.06 e5a7af1e-b3be-47c6-8f8b-b547a2d73945
## INFO  [08:02:40.372] [bbotk] Evaluating 1 configuration(s)
## INFO  [08:02:40.390] [mlr3] Running benchmark with 10 resampling iterations
## INFO  [08:02:40.395] [mlr3] Applying learner 'regr.svm' on task 'PredictionSet[1:13]' (iter 1/10)
## INFO  [08:02:40.411] [mlr3] Applying learner 'regr.svm' on task 'PredictionSet[1:13]' (iter 2/10)
## INFO  [08:02:40.427] [mlr3] Applying learner 'regr.svm' on task 'PredictionSet[1:13]' (iter 3/10)
## INFO  [08:02:40.453] [mlr3] Applying learner 'regr.svm' on task 'PredictionSet[1:13]' (iter 4/10)
## INFO  [08:02:40.475] [mlr3] Applying learner 'regr.svm' on task 'PredictionSet[1:13]' (iter 5/10)
## INFO  [08:02:40.494] [mlr3] Applying learner 'regr.svm' on task 'PredictionSet[1:13]' (iter 6/10)
## INFO  [08:02:40.512] [mlr3] Applying learner 'regr.svm' on task 'PredictionSet[1:13]' (iter 7/10)
## INFO  [08:02:40.532] [mlr3] Applying learner 'regr.svm' on task 'PredictionSet[1:13]' (iter 8/10)
## INFO  [08:02:40.551] [mlr3] Applying learner 'regr.svm' on task 'PredictionSet[1:13]' (iter 9/10)
## INFO  [08:02:40.568] [mlr3] Applying learner 'regr.svm' on task 'PredictionSet[1:13]' (iter 10/10)
## INFO  [08:02:40.583] [mlr3] Finished benchmark
## INFO  [08:02:40.616] [bbotk] Result of batch 10:
## INFO  [08:02:40.618] [bbotk]  tolerance    gamma   epsilon           type kernel regr.rmse warnings errors
## INFO  [08:02:40.618] [bbotk]     0.4505 46.42864 0.7857143 eps-regression radial  18.54283        0      0
## INFO  [08:02:40.618] [bbotk]  runtime_learners                                uhash
## INFO  [08:02:40.618] [bbotk]             0.066 30f9d5e7-94f7-44fa-a41c-6f6fdf67973e
## INFO  [08:02:40.623] [bbotk] Finished optimizing after 10 evaluation(s)
## INFO  [08:02:40.624] [bbotk] Result:
## INFO  [08:02:40.625] [bbotk]  tolerance   gamma   epsilon           type kernel learner_param_vals  x_domain
## INFO  [08:02:40.625] [bbotk]  0.6431429 25.0005 0.6142857 eps-regression radial          <list[5]> <list[5]>
## INFO  [08:02:40.625] [bbotk]  regr.rmse
## INFO  [08:02:40.625] [bbotk]   17.30811
## INFO  [08:02:40.779] [bbotk] Starting to optimize 3 parameter(s) with '<TunerGridSearch>' and '<TerminatorEvals> [n_evals=10, k=0]'
## INFO  [08:02:40.783] [bbotk] Evaluating 1 configuration(s)
## INFO  [08:02:40.797] [mlr3] Running benchmark with 10 resampling iterations
## INFO  [08:02:40.803] [mlr3] Applying learner 'regr.kknn' on task 'PredictionSet[1:13]' (iter 1/10)
## INFO  [08:02:40.827] [mlr3] Applying learner 'regr.kknn' on task 'PredictionSet[1:13]' (iter 2/10)
## INFO  [08:02:40.853] [mlr3] Applying learner 'regr.kknn' on task 'PredictionSet[1:13]' (iter 3/10)
## INFO  [08:02:40.874] [mlr3] Applying learner 'regr.kknn' on task 'PredictionSet[1:13]' (iter 4/10)
## INFO  [08:02:40.895] [mlr3] Applying learner 'regr.kknn' on task 'PredictionSet[1:13]' (iter 5/10)
## INFO  [08:02:40.917] [mlr3] Applying learner 'regr.kknn' on task 'PredictionSet[1:13]' (iter 6/10)
## INFO  [08:02:40.938] [mlr3] Applying learner 'regr.kknn' on task 'PredictionSet[1:13]' (iter 7/10)
## INFO  [08:02:40.958] [mlr3] Applying learner 'regr.kknn' on task 'PredictionSet[1:13]' (iter 8/10)
## INFO  [08:02:40.978] [mlr3] Applying learner 'regr.kknn' on task 'PredictionSet[1:13]' (iter 9/10)
## INFO  [08:02:41.001] [mlr3] Applying learner 'regr.kknn' on task 'PredictionSet[1:13]' (iter 10/10)
## INFO  [08:02:41.021] [mlr3] Finished benchmark
## INFO  [08:02:41.051] [bbotk] Result of batch 1:
## INFO  [08:02:41.052] [bbotk]  k distance     kernel regr.rmse warnings errors runtime_learners
## INFO  [08:02:41.052] [bbotk]  1 18.57857 triangular  11.51955        0      0            0.106
## INFO  [08:02:41.052] [bbotk]                                 uhash
## INFO  [08:02:41.052] [bbotk]  4b3edaac-e23c-4e36-8d82-03850df00a04
## INFO  [08:02:41.054] [bbotk] Evaluating 1 configuration(s)
## INFO  [08:02:41.066] [mlr3] Running benchmark with 10 resampling iterations
## INFO  [08:02:41.071] [mlr3] Applying learner 'regr.kknn' on task 'PredictionSet[1:13]' (iter 1/10)
## INFO  [08:02:41.092] [mlr3] Applying learner 'regr.kknn' on task 'PredictionSet[1:13]' (iter 2/10)
## INFO  [08:02:41.112] [mlr3] Applying learner 'regr.kknn' on task 'PredictionSet[1:13]' (iter 3/10)
## INFO  [08:02:41.145] [mlr3] Applying learner 'regr.kknn' on task 'PredictionSet[1:13]' (iter 4/10)
## INFO  [08:02:41.171] [mlr3] Applying learner 'regr.kknn' on task 'PredictionSet[1:13]' (iter 5/10)
## INFO  [08:02:41.195] [mlr3] Applying learner 'regr.kknn' on task 'PredictionSet[1:13]' (iter 6/10)
## INFO  [08:02:41.223] [mlr3] Applying learner 'regr.kknn' on task 'PredictionSet[1:13]' (iter 7/10)
## INFO  [08:02:41.247] [mlr3] Applying learner 'regr.kknn' on task 'PredictionSet[1:13]' (iter 8/10)
## INFO  [08:02:41.268] [mlr3] Applying learner 'regr.kknn' on task 'PredictionSet[1:13]' (iter 9/10)
## INFO  [08:02:41.290] [mlr3] Applying learner 'regr.kknn' on task 'PredictionSet[1:13]' (iter 10/10)
## INFO  [08:02:41.310] [mlr3] Finished benchmark
## INFO  [08:02:41.342] [bbotk] Result of batch 2:
## INFO  [08:02:41.343] [bbotk]   k distance  kernel regr.rmse warnings errors runtime_learners
## INFO  [08:02:41.343] [bbotk]  19 2.942857 optimal  10.97958        0      0            0.125
## INFO  [08:02:41.343] [bbotk]                                 uhash
## INFO  [08:02:41.343] [bbotk]  f6103d8a-4ad8-4161-b2e8-82233382814f
## INFO  [08:02:41.345] [bbotk] Evaluating 1 configuration(s)
## INFO  [08:02:41.356] [mlr3] Running benchmark with 10 resampling iterations
## INFO  [08:02:41.362] [mlr3] Applying learner 'regr.kknn' on task 'PredictionSet[1:13]' (iter 1/10)
## INFO  [08:02:41.383] [mlr3] Applying learner 'regr.kknn' on task 'PredictionSet[1:13]' (iter 2/10)
## INFO  [08:02:41.403] [mlr3] Applying learner 'regr.kknn' on task 'PredictionSet[1:13]' (iter 3/10)
## INFO  [08:02:41.424] [mlr3] Applying learner 'regr.kknn' on task 'PredictionSet[1:13]' (iter 4/10)
## INFO  [08:02:41.447] [mlr3] Applying learner 'regr.kknn' on task 'PredictionSet[1:13]' (iter 5/10)
## INFO  [08:02:41.468] [mlr3] Applying learner 'regr.kknn' on task 'PredictionSet[1:13]' (iter 6/10)
## INFO  [08:02:41.489] [mlr3] Applying learner 'regr.kknn' on task 'PredictionSet[1:13]' (iter 7/10)
## INFO  [08:02:41.524] [mlr3] Applying learner 'regr.kknn' on task 'PredictionSet[1:13]' (iter 8/10)
## INFO  [08:02:41.549] [mlr3] Applying learner 'regr.kknn' on task 'PredictionSet[1:13]' (iter 9/10)
## INFO  [08:02:41.573] [mlr3] Applying learner 'regr.kknn' on task 'PredictionSet[1:13]' (iter 10/10)
## INFO  [08:02:41.600] [mlr3] Finished benchmark
## INFO  [08:02:41.635] [bbotk] Result of batch 3:
## INFO  [08:02:41.637] [bbotk]   k distance      kernel regr.rmse warnings errors runtime_learners
## INFO  [08:02:41.637] [bbotk]  16 7.207143 rectangular  12.48946        0      0            0.115
## INFO  [08:02:41.637] [bbotk]                                 uhash
## INFO  [08:02:41.637] [bbotk]  3cc6f712-fa22-4aae-a1eb-ffccbb592893
## INFO  [08:02:41.638] [bbotk] Evaluating 1 configuration(s)
## INFO  [08:02:41.649] [mlr3] Running benchmark with 10 resampling iterations
## INFO  [08:02:41.654] [mlr3] Applying learner 'regr.kknn' on task 'PredictionSet[1:13]' (iter 1/10)
## INFO  [08:02:41.677] [mlr3] Applying learner 'regr.kknn' on task 'PredictionSet[1:13]' (iter 2/10)
## INFO  [08:02:41.698] [mlr3] Applying learner 'regr.kknn' on task 'PredictionSet[1:13]' (iter 3/10)
## INFO  [08:02:41.718] [mlr3] Applying learner 'regr.kknn' on task 'PredictionSet[1:13]' (iter 4/10)
## INFO  [08:02:41.741] [mlr3] Applying learner 'regr.kknn' on task 'PredictionSet[1:13]' (iter 5/10)
## INFO  [08:02:41.762] [mlr3] Applying learner 'regr.kknn' on task 'PredictionSet[1:13]' (iter 6/10)
## INFO  [08:02:41.782] [mlr3] Applying learner 'regr.kknn' on task 'PredictionSet[1:13]' (iter 7/10)
## INFO  [08:02:41.803] [mlr3] Applying learner 'regr.kknn' on task 'PredictionSet[1:13]' (iter 8/10)
## INFO  [08:02:41.826] [mlr3] Applying learner 'regr.kknn' on task 'PredictionSet[1:13]' (iter 9/10)
## INFO  [08:02:41.847] [mlr3] Applying learner 'regr.kknn' on task 'PredictionSet[1:13]' (iter 10/10)
## INFO  [08:02:42.093] [mlr3] Finished benchmark
## INFO  [08:02:42.127] [bbotk] Result of batch 4:
## INFO  [08:02:42.129] [bbotk]   k distance    kernel regr.rmse warnings errors runtime_learners
## INFO  [08:02:42.129] [bbotk]  18 1.521429 triweight  11.11598        0      0             0.11
## INFO  [08:02:42.129] [bbotk]                                 uhash
## INFO  [08:02:42.129] [bbotk]  d1ed50c8-d41d-4e67-b4e5-2e643218d443
## INFO  [08:02:42.130] [bbotk] Evaluating 1 configuration(s)
## INFO  [08:02:42.140] [mlr3] Running benchmark with 10 resampling iterations
## INFO  [08:02:42.145] [mlr3] Applying learner 'regr.kknn' on task 'PredictionSet[1:13]' (iter 1/10)
## INFO  [08:02:42.165] [mlr3] Applying learner 'regr.kknn' on task 'PredictionSet[1:13]' (iter 2/10)
## INFO  [08:02:42.186] [mlr3] Applying learner 'regr.kknn' on task 'PredictionSet[1:13]' (iter 3/10)
## INFO  [08:02:42.207] [mlr3] Applying learner 'regr.kknn' on task 'PredictionSet[1:13]' (iter 4/10)
## INFO  [08:02:42.227] [mlr3] Applying learner 'regr.kknn' on task 'PredictionSet[1:13]' (iter 5/10)
## INFO  [08:02:42.247] [mlr3] Applying learner 'regr.kknn' on task 'PredictionSet[1:13]' (iter 6/10)
## INFO  [08:02:42.269] [mlr3] Applying learner 'regr.kknn' on task 'PredictionSet[1:13]' (iter 7/10)
## INFO  [08:02:42.289] [mlr3] Applying learner 'regr.kknn' on task 'PredictionSet[1:13]' (iter 8/10)
## INFO  [08:02:42.309] [mlr3] Applying learner 'regr.kknn' on task 'PredictionSet[1:13]' (iter 9/10)
## INFO  [08:02:42.329] [mlr3] Applying learner 'regr.kknn' on task 'PredictionSet[1:13]' (iter 10/10)
## INFO  [08:02:42.350] [mlr3] Finished benchmark
## INFO  [08:02:42.381] [bbotk] Result of batch 5:
## INFO  [08:02:42.382] [bbotk]  k distance kernel regr.rmse warnings errors runtime_learners
## INFO  [08:02:42.382] [bbotk]  8 2.942857    cos  11.66575        0      0            0.098
## INFO  [08:02:42.382] [bbotk]                                 uhash
## INFO  [08:02:42.382] [bbotk]  97af393c-2f90-48ac-9d97-fae06e51e9c9
## INFO  [08:02:42.384] [bbotk] Evaluating 1 configuration(s)
## INFO  [08:02:42.395] [mlr3] Running benchmark with 10 resampling iterations
## INFO  [08:02:42.400] [mlr3] Applying learner 'regr.kknn' on task 'PredictionSet[1:13]' (iter 1/10)
## INFO  [08:02:42.424] [mlr3] Applying learner 'regr.kknn' on task 'PredictionSet[1:13]' (iter 2/10)
## INFO  [08:02:42.445] [mlr3] Applying learner 'regr.kknn' on task 'PredictionSet[1:13]' (iter 3/10)
## INFO  [08:02:42.472] [mlr3] Applying learner 'regr.kknn' on task 'PredictionSet[1:13]' (iter 4/10)
## INFO  [08:02:42.494] [mlr3] Applying learner 'regr.kknn' on task 'PredictionSet[1:13]' (iter 5/10)
## INFO  [08:02:42.514] [mlr3] Applying learner 'regr.kknn' on task 'PredictionSet[1:13]' (iter 6/10)
## INFO  [08:02:42.534] [mlr3] Applying learner 'regr.kknn' on task 'PredictionSet[1:13]' (iter 7/10)
## INFO  [08:02:42.554] [mlr3] Applying learner 'regr.kknn' on task 'PredictionSet[1:13]' (iter 8/10)
## INFO  [08:02:42.576] [mlr3] Applying learner 'regr.kknn' on task 'PredictionSet[1:13]' (iter 9/10)
## INFO  [08:02:42.597] [mlr3] Applying learner 'regr.kknn' on task 'PredictionSet[1:13]' (iter 10/10)
## INFO  [08:02:42.616] [mlr3] Finished benchmark
## INFO  [08:02:42.649] [bbotk] Result of batch 6:
## INFO  [08:02:42.650] [bbotk]   k distance    kernel regr.rmse warnings errors runtime_learners
## INFO  [08:02:42.650] [bbotk]  10 15.73571 triweight  12.22028        0      0            0.116
## INFO  [08:02:42.650] [bbotk]                                 uhash
## INFO  [08:02:42.650] [bbotk]  fe2d8583-040b-43a4-9a1b-8fe87448cf17
## INFO  [08:02:42.652] [bbotk] Evaluating 1 configuration(s)
## INFO  [08:02:42.662] [mlr3] Running benchmark with 10 resampling iterations
## INFO  [08:02:42.667] [mlr3] Applying learner 'regr.kknn' on task 'PredictionSet[1:13]' (iter 1/10)
## INFO  [08:02:42.686] [mlr3] Applying learner 'regr.kknn' on task 'PredictionSet[1:13]' (iter 2/10)
## INFO  [08:02:42.707] [mlr3] Applying learner 'regr.kknn' on task 'PredictionSet[1:13]' (iter 3/10)
## INFO  [08:02:42.728] [mlr3] Applying learner 'regr.kknn' on task 'PredictionSet[1:13]' (iter 4/10)
## INFO  [08:02:42.748] [mlr3] Applying learner 'regr.kknn' on task 'PredictionSet[1:13]' (iter 5/10)
## INFO  [08:02:42.768] [mlr3] Applying learner 'regr.kknn' on task 'PredictionSet[1:13]' (iter 6/10)
## INFO  [08:02:42.790] [mlr3] Applying learner 'regr.kknn' on task 'PredictionSet[1:13]' (iter 7/10)
## INFO  [08:02:42.811] [mlr3] Applying learner 'regr.kknn' on task 'PredictionSet[1:13]' (iter 8/10)
## INFO  [08:02:42.831] [mlr3] Applying learner 'regr.kknn' on task 'PredictionSet[1:13]' (iter 9/10)
## INFO  [08:02:42.858] [mlr3] Applying learner 'regr.kknn' on task 'PredictionSet[1:13]' (iter 10/10)
## INFO  [08:02:42.879] [mlr3] Finished benchmark
## INFO  [08:02:42.914] [bbotk] Result of batch 7:
## INFO  [08:02:42.916] [bbotk]  k distance       kernel regr.rmse warnings errors runtime_learners
## INFO  [08:02:42.916] [bbotk]  8 17.15714 epanechnikov  12.34296        0      0            0.104
## INFO  [08:02:42.916] [bbotk]                                 uhash
## INFO  [08:02:42.916] [bbotk]  91ac915d-3c14-4afc-962a-afbbce336bc9
## INFO  [08:02:42.918] [bbotk] Evaluating 1 configuration(s)
## INFO  [08:02:42.928] [mlr3] Running benchmark with 10 resampling iterations
## INFO  [08:02:42.934] [mlr3] Applying learner 'regr.kknn' on task 'PredictionSet[1:13]' (iter 1/10)
## INFO  [08:02:42.955] [mlr3] Applying learner 'regr.kknn' on task 'PredictionSet[1:13]' (iter 2/10)
## INFO  [08:02:42.975] [mlr3] Applying learner 'regr.kknn' on task 'PredictionSet[1:13]' (iter 3/10)
## INFO  [08:02:42.995] [mlr3] Applying learner 'regr.kknn' on task 'PredictionSet[1:13]' (iter 4/10)
## INFO  [08:02:43.017] [mlr3] Applying learner 'regr.kknn' on task 'PredictionSet[1:13]' (iter 5/10)
## INFO  [08:02:43.038] [mlr3] Applying learner 'regr.kknn' on task 'PredictionSet[1:13]' (iter 6/10)
## INFO  [08:02:43.058] [mlr3] Applying learner 'regr.kknn' on task 'PredictionSet[1:13]' (iter 7/10)
## INFO  [08:02:43.078] [mlr3] Applying learner 'regr.kknn' on task 'PredictionSet[1:13]' (iter 8/10)
## INFO  [08:02:43.100] [mlr3] Applying learner 'regr.kknn' on task 'PredictionSet[1:13]' (iter 9/10)
## INFO  [08:02:43.120] [mlr3] Applying learner 'regr.kknn' on task 'PredictionSet[1:13]' (iter 10/10)
## INFO  [08:02:43.139] [mlr3] Finished benchmark
## INFO  [08:02:43.172] [bbotk] Result of batch 8:
## INFO  [08:02:43.174] [bbotk]   k distance    kernel regr.rmse warnings errors runtime_learners
## INFO  [08:02:43.174] [bbotk]  12 18.57857 triweight  11.85149        0      0            0.101
## INFO  [08:02:43.174] [bbotk]                                 uhash
## INFO  [08:02:43.174] [bbotk]  f04b1516-2f20-4c3b-894a-e0c1467ef414
## INFO  [08:02:43.175] [bbotk] Evaluating 1 configuration(s)
## INFO  [08:02:43.186] [mlr3] Running benchmark with 10 resampling iterations
## INFO  [08:02:43.191] [mlr3] Applying learner 'regr.kknn' on task 'PredictionSet[1:13]' (iter 1/10)
## INFO  [08:02:43.211] [mlr3] Applying learner 'regr.kknn' on task 'PredictionSet[1:13]' (iter 2/10)
## INFO  [08:02:43.239] [mlr3] Applying learner 'regr.kknn' on task 'PredictionSet[1:13]' (iter 3/10)
## INFO  [08:02:43.260] [mlr3] Applying learner 'regr.kknn' on task 'PredictionSet[1:13]' (iter 4/10)
## INFO  [08:02:43.280] [mlr3] Applying learner 'regr.kknn' on task 'PredictionSet[1:13]' (iter 5/10)
## INFO  [08:02:43.301] [mlr3] Applying learner 'regr.kknn' on task 'PredictionSet[1:13]' (iter 6/10)
## INFO  [08:02:43.323] [mlr3] Applying learner 'regr.kknn' on task 'PredictionSet[1:13]' (iter 7/10)
## INFO  [08:02:43.344] [mlr3] Applying learner 'regr.kknn' on task 'PredictionSet[1:13]' (iter 8/10)
## INFO  [08:02:43.364] [mlr3] Applying learner 'regr.kknn' on task 'PredictionSet[1:13]' (iter 9/10)
## INFO  [08:02:43.385] [mlr3] Applying learner 'regr.kknn' on task 'PredictionSet[1:13]' (iter 10/10)
## INFO  [08:02:43.405] [mlr3] Finished benchmark
## INFO  [08:02:43.436] [bbotk] Result of batch 9:
## INFO  [08:02:43.438] [bbotk]  k distance kernel regr.rmse warnings errors runtime_learners
## INFO  [08:02:43.438] [bbotk]  1       20   rank  11.51955        0      0            0.105
## INFO  [08:02:43.438] [bbotk]                                 uhash
## INFO  [08:02:43.438] [bbotk]  ffb38c07-33f8-47e6-84d4-d9a23285fdb3
## INFO  [08:02:43.439] [bbotk] Evaluating 1 configuration(s)
## INFO  [08:02:43.450] [mlr3] Running benchmark with 10 resampling iterations
## INFO  [08:02:43.455] [mlr3] Applying learner 'regr.kknn' on task 'PredictionSet[1:13]' (iter 1/10)
## INFO  [08:02:43.476] [mlr3] Applying learner 'regr.kknn' on task 'PredictionSet[1:13]' (iter 2/10)
## INFO  [08:02:43.496] [mlr3] Applying learner 'regr.kknn' on task 'PredictionSet[1:13]' (iter 3/10)
## INFO  [08:02:43.516] [mlr3] Applying learner 'regr.kknn' on task 'PredictionSet[1:13]' (iter 4/10)
## INFO  [08:02:43.538] [mlr3] Applying learner 'regr.kknn' on task 'PredictionSet[1:13]' (iter 5/10)
## INFO  [08:02:43.558] [mlr3] Applying learner 'regr.kknn' on task 'PredictionSet[1:13]' (iter 6/10)
## INFO  [08:02:43.578] [mlr3] Applying learner 'regr.kknn' on task 'PredictionSet[1:13]' (iter 7/10)
## INFO  [08:02:43.598] [mlr3] Applying learner 'regr.kknn' on task 'PredictionSet[1:13]' (iter 8/10)
## INFO  [08:02:43.627] [mlr3] Applying learner 'regr.kknn' on task 'PredictionSet[1:13]' (iter 9/10)
## INFO  [08:02:43.647] [mlr3] Applying learner 'regr.kknn' on task 'PredictionSet[1:13]' (iter 10/10)
## INFO  [08:02:43.667] [mlr3] Finished benchmark
## INFO  [08:02:43.700] [bbotk] Result of batch 10:
## INFO  [08:02:43.701] [bbotk]  k distance kernel regr.rmse warnings errors runtime_learners
## INFO  [08:02:43.701] [bbotk]  3 12.89286    cos  12.07449        0      0            0.107
## INFO  [08:02:43.701] [bbotk]                                 uhash
## INFO  [08:02:43.701] [bbotk]  c7af7577-d1c6-478f-b146-08187a6f8274
## INFO  [08:02:43.706] [bbotk] Finished optimizing after 10 evaluation(s)
## INFO  [08:02:43.707] [bbotk] Result:
## INFO  [08:02:43.708] [bbotk]   k distance  kernel learner_param_vals  x_domain regr.rmse
## INFO  [08:02:43.708] [bbotk]  19 2.942857 optimal          <list[3]> <list[3]>  10.97958
## INFO  [08:02:43.922] [bbotk] Starting to optimize 2 parameter(s) with '<TunerGridSearch>' and '<TerminatorEvals> [n_evals=10, k=0]'
## INFO  [08:02:43.925] [bbotk] Evaluating 1 configuration(s)
## INFO  [08:02:43.946] [mlr3] Running benchmark with 10 resampling iterations
## INFO  [08:02:43.951] [mlr3] Applying learner 'regr.ranger' on task 'PredictionSet[1:13]' (iter 1/10)
## INFO  [08:02:43.991] [mlr3] Applying learner 'regr.ranger' on task 'PredictionSet[1:13]' (iter 2/10)
## INFO  [08:02:44.028] [mlr3] Applying learner 'regr.ranger' on task 'PredictionSet[1:13]' (iter 3/10)
## INFO  [08:02:44.067] [mlr3] Applying learner 'regr.ranger' on task 'PredictionSet[1:13]' (iter 4/10)
## INFO  [08:02:44.103] [mlr3] Applying learner 'regr.ranger' on task 'PredictionSet[1:13]' (iter 5/10)
## INFO  [08:02:44.142] [mlr3] Applying learner 'regr.ranger' on task 'PredictionSet[1:13]' (iter 6/10)
## INFO  [08:02:44.179] [mlr3] Applying learner 'regr.ranger' on task 'PredictionSet[1:13]' (iter 7/10)
## INFO  [08:02:44.218] [mlr3] Applying learner 'regr.ranger' on task 'PredictionSet[1:13]' (iter 8/10)
## INFO  [08:02:44.254] [mlr3] Applying learner 'regr.ranger' on task 'PredictionSet[1:13]' (iter 9/10)
## INFO  [08:02:44.292] [mlr3] Applying learner 'regr.ranger' on task 'PredictionSet[1:13]' (iter 10/10)
## INFO  [08:02:44.328] [mlr3] Finished benchmark
## INFO  [08:02:44.357] [bbotk] Result of batch 1:
## INFO  [08:02:44.359] [bbotk]  max.depth num.trees regr.rmse warnings errors runtime_learners
## INFO  [08:02:44.359] [bbotk]          4       500  11.89201        0      0            0.273
## INFO  [08:02:44.359] [bbotk]                                 uhash
## INFO  [08:02:44.359] [bbotk]  60d2329c-1e9d-47a8-b2f6-9f53a4cec7b2
## INFO  [08:02:44.360] [bbotk] Evaluating 1 configuration(s)
## INFO  [08:02:44.382] [mlr3] Running benchmark with 10 resampling iterations
## INFO  [08:02:44.387] [mlr3] Applying learner 'regr.ranger' on task 'PredictionSet[1:13]' (iter 1/10)
## INFO  [08:02:44.421] [mlr3] Applying learner 'regr.ranger' on task 'PredictionSet[1:13]' (iter 2/10)
## INFO  [08:02:44.457] [mlr3] Applying learner 'regr.ranger' on task 'PredictionSet[1:13]' (iter 3/10)
## INFO  [08:02:44.491] [mlr3] Applying learner 'regr.ranger' on task 'PredictionSet[1:13]' (iter 4/10)
## INFO  [08:02:44.534] [mlr3] Applying learner 'regr.ranger' on task 'PredictionSet[1:13]' (iter 5/10)
## INFO  [08:02:44.568] [mlr3] Applying learner 'regr.ranger' on task 'PredictionSet[1:13]' (iter 6/10)
## INFO  [08:02:44.603] [mlr3] Applying learner 'regr.ranger' on task 'PredictionSet[1:13]' (iter 7/10)
## INFO  [08:02:44.637] [mlr3] Applying learner 'regr.ranger' on task 'PredictionSet[1:13]' (iter 8/10)
## INFO  [08:02:44.673] [mlr3] Applying learner 'regr.ranger' on task 'PredictionSet[1:13]' (iter 9/10)
## INFO  [08:02:44.707] [mlr3] Applying learner 'regr.ranger' on task 'PredictionSet[1:13]' (iter 10/10)
## INFO  [08:02:44.742] [mlr3] Finished benchmark
## INFO  [08:02:44.773] [bbotk] Result of batch 2:
## INFO  [08:02:44.774] [bbotk]  max.depth num.trees regr.rmse warnings errors runtime_learners
## INFO  [08:02:44.774] [bbotk]         12       290  10.33727        0      0            0.243
## INFO  [08:02:44.774] [bbotk]                                 uhash
## INFO  [08:02:44.774] [bbotk]  b15e00b1-8b21-49ab-b766-8a3a9b3378a0
## INFO  [08:02:44.775] [bbotk] Evaluating 1 configuration(s)
## INFO  [08:02:44.796] [mlr3] Running benchmark with 10 resampling iterations
## INFO  [08:02:44.801] [mlr3] Applying learner 'regr.ranger' on task 'PredictionSet[1:13]' (iter 1/10)
## INFO  [08:02:44.825] [mlr3] Applying learner 'regr.ranger' on task 'PredictionSet[1:13]' (iter 2/10)
## INFO  [08:02:44.846] [mlr3] Applying learner 'regr.ranger' on task 'PredictionSet[1:13]' (iter 3/10)
## INFO  [08:02:44.867] [mlr3] Applying learner 'regr.ranger' on task 'PredictionSet[1:13]' (iter 4/10)
## INFO  [08:02:44.891] [mlr3] Applying learner 'regr.ranger' on task 'PredictionSet[1:13]' (iter 5/10)
## INFO  [08:02:44.912] [mlr3] Applying learner 'regr.ranger' on task 'PredictionSet[1:13]' (iter 6/10)
## INFO  [08:02:44.934] [mlr3] Applying learner 'regr.ranger' on task 'PredictionSet[1:13]' (iter 7/10)
## INFO  [08:02:44.956] [mlr3] Applying learner 'regr.ranger' on task 'PredictionSet[1:13]' (iter 8/10)
## INFO  [08:02:44.979] [mlr3] Applying learner 'regr.ranger' on task 'PredictionSet[1:13]' (iter 9/10)
## INFO  [08:02:45.001] [mlr3] Applying learner 'regr.ranger' on task 'PredictionSet[1:13]' (iter 10/10)
## INFO  [08:02:45.022] [mlr3] Finished benchmark
## INFO  [08:02:45.081] [bbotk] Result of batch 3:
## INFO  [08:02:45.083] [bbotk]  max.depth num.trees regr.rmse warnings errors runtime_learners
## INFO  [08:02:45.083] [bbotk]          4       158  12.02848        0      0            0.119
## INFO  [08:02:45.083] [bbotk]                                 uhash
## INFO  [08:02:45.083] [bbotk]  f44fb706-efe8-46b2-9a3c-076f82ba9b26
## INFO  [08:02:45.084] [bbotk] Evaluating 1 configuration(s)
## INFO  [08:02:45.107] [mlr3] Running benchmark with 10 resampling iterations
## INFO  [08:02:45.112] [mlr3] Applying learner 'regr.ranger' on task 'PredictionSet[1:13]' (iter 1/10)
## INFO  [08:02:45.128] [mlr3] Applying learner 'regr.ranger' on task 'PredictionSet[1:13]' (iter 2/10)
## INFO  [08:02:45.143] [mlr3] Applying learner 'regr.ranger' on task 'PredictionSet[1:13]' (iter 3/10)
## INFO  [08:02:45.158] [mlr3] Applying learner 'regr.ranger' on task 'PredictionSet[1:13]' (iter 4/10)
## INFO  [08:02:45.174] [mlr3] Applying learner 'regr.ranger' on task 'PredictionSet[1:13]' (iter 5/10)
## INFO  [08:02:45.191] [mlr3] Applying learner 'regr.ranger' on task 'PredictionSet[1:13]' (iter 6/10)
## INFO  [08:02:45.206] [mlr3] Applying learner 'regr.ranger' on task 'PredictionSet[1:13]' (iter 7/10)
## INFO  [08:02:45.221] [mlr3] Applying learner 'regr.ranger' on task 'PredictionSet[1:13]' (iter 8/10)
## INFO  [08:02:45.237] [mlr3] Applying learner 'regr.ranger' on task 'PredictionSet[1:13]' (iter 9/10)
## INFO  [08:02:45.252] [mlr3] Applying learner 'regr.ranger' on task 'PredictionSet[1:13]' (iter 10/10)
## INFO  [08:02:45.269] [mlr3] Finished benchmark
## INFO  [08:02:45.300] [bbotk] Result of batch 4:
## INFO  [08:02:45.302] [bbotk]  max.depth num.trees regr.rmse warnings errors runtime_learners
## INFO  [08:02:45.302] [bbotk]          1        27  16.36753        0      0            0.056
## INFO  [08:02:45.302] [bbotk]                                 uhash
## INFO  [08:02:45.302] [bbotk]  35f75fa6-0fa3-49ef-ab2c-fb18dfe184b4
## INFO  [08:02:45.303] [bbotk] Evaluating 1 configuration(s)
## INFO  [08:02:45.325] [mlr3] Running benchmark with 10 resampling iterations
## INFO  [08:02:45.331] [mlr3] Applying learner 'regr.ranger' on task 'PredictionSet[1:13]' (iter 1/10)
## INFO  [08:02:45.369] [mlr3] Applying learner 'regr.ranger' on task 'PredictionSet[1:13]' (iter 2/10)
## INFO  [08:02:45.405] [mlr3] Applying learner 'regr.ranger' on task 'PredictionSet[1:13]' (iter 3/10)
## INFO  [08:02:45.443] [mlr3] Applying learner 'regr.ranger' on task 'PredictionSet[1:13]' (iter 4/10)
## INFO  [08:02:45.488] [mlr3] Applying learner 'regr.ranger' on task 'PredictionSet[1:13]' (iter 5/10)
## INFO  [08:02:45.527] [mlr3] Applying learner 'regr.ranger' on task 'PredictionSet[1:13]' (iter 6/10)
## INFO  [08:02:45.565] [mlr3] Applying learner 'regr.ranger' on task 'PredictionSet[1:13]' (iter 7/10)
## INFO  [08:02:45.601] [mlr3] Applying learner 'regr.ranger' on task 'PredictionSet[1:13]' (iter 8/10)
## INFO  [08:02:45.639] [mlr3] Applying learner 'regr.ranger' on task 'PredictionSet[1:13]' (iter 9/10)
## INFO  [08:02:45.674] [mlr3] Applying learner 'regr.ranger' on task 'PredictionSet[1:13]' (iter 10/10)
## INFO  [08:02:45.711] [mlr3] Finished benchmark
## INFO  [08:02:45.742] [bbotk] Result of batch 5:
## INFO  [08:02:45.743] [bbotk]  max.depth num.trees regr.rmse warnings errors runtime_learners
## INFO  [08:02:45.743] [bbotk]          9       316  10.25108        0      0            0.265
## INFO  [08:02:45.743] [bbotk]                                 uhash
## INFO  [08:02:45.743] [bbotk]  e9c448e5-a565-473a-a631-a9571e794424
## INFO  [08:02:45.745] [bbotk] Evaluating 1 configuration(s)
## INFO  [08:02:45.766] [mlr3] Running benchmark with 10 resampling iterations
## INFO  [08:02:45.770] [mlr3] Applying learner 'regr.ranger' on task 'PredictionSet[1:13]' (iter 1/10)
## INFO  [08:02:45.795] [mlr3] Applying learner 'regr.ranger' on task 'PredictionSet[1:13]' (iter 2/10)
## INFO  [08:02:45.817] [mlr3] Applying learner 'regr.ranger' on task 'PredictionSet[1:13]' (iter 3/10)
## INFO  [08:02:45.839] [mlr3] Applying learner 'regr.ranger' on task 'PredictionSet[1:13]' (iter 4/10)
## INFO  [08:02:45.863] [mlr3] Applying learner 'regr.ranger' on task 'PredictionSet[1:13]' (iter 5/10)
## INFO  [08:02:45.886] [mlr3] Applying learner 'regr.ranger' on task 'PredictionSet[1:13]' (iter 6/10)
## INFO  [08:02:45.908] [mlr3] Applying learner 'regr.ranger' on task 'PredictionSet[1:13]' (iter 7/10)
## INFO  [08:02:45.932] [mlr3] Applying learner 'regr.ranger' on task 'PredictionSet[1:13]' (iter 8/10)
## INFO  [08:02:45.955] [mlr3] Applying learner 'regr.ranger' on task 'PredictionSet[1:13]' (iter 9/10)
## INFO  [08:02:45.977] [mlr3] Applying learner 'regr.ranger' on task 'PredictionSet[1:13]' (iter 10/10)
## INFO  [08:02:45.999] [mlr3] Finished benchmark
## INFO  [08:02:46.040] [bbotk] Result of batch 6:
## INFO  [08:02:46.041] [bbotk]  max.depth num.trees regr.rmse warnings errors runtime_learners
## INFO  [08:02:46.041] [bbotk]          1       343  16.25381        0      0            0.124
## INFO  [08:02:46.041] [bbotk]                                 uhash
## INFO  [08:02:46.041] [bbotk]  0cded2c3-994e-47c7-a4c0-c1c3b7dad70e
## INFO  [08:02:46.043] [bbotk] Evaluating 1 configuration(s)
## INFO  [08:02:46.064] [mlr3] Running benchmark with 10 resampling iterations
## INFO  [08:02:46.069] [mlr3] Applying learner 'regr.ranger' on task 'PredictionSet[1:13]' (iter 1/10)
## INFO  [08:02:46.106] [mlr3] Applying learner 'regr.ranger' on task 'PredictionSet[1:13]' (iter 2/10)
## INFO  [08:02:46.141] [mlr3] Applying learner 'regr.ranger' on task 'PredictionSet[1:13]' (iter 3/10)
## INFO  [08:02:46.178] [mlr3] Applying learner 'regr.ranger' on task 'PredictionSet[1:13]' (iter 4/10)
## INFO  [08:02:46.214] [mlr3] Applying learner 'regr.ranger' on task 'PredictionSet[1:13]' (iter 5/10)
## INFO  [08:02:46.251] [mlr3] Applying learner 'regr.ranger' on task 'PredictionSet[1:13]' (iter 6/10)
## INFO  [08:02:46.286] [mlr3] Applying learner 'regr.ranger' on task 'PredictionSet[1:13]' (iter 7/10)
## INFO  [08:02:46.323] [mlr3] Applying learner 'regr.ranger' on task 'PredictionSet[1:13]' (iter 8/10)
## INFO  [08:02:46.359] [mlr3] Applying learner 'regr.ranger' on task 'PredictionSet[1:13]' (iter 9/10)
## INFO  [08:02:46.396] [mlr3] Applying learner 'regr.ranger' on task 'PredictionSet[1:13]' (iter 10/10)
## INFO  [08:02:46.431] [mlr3] Finished benchmark
## INFO  [08:02:46.464] [bbotk] Result of batch 7:
## INFO  [08:02:46.466] [bbotk]  max.depth num.trees regr.rmse warnings errors runtime_learners
## INFO  [08:02:46.466] [bbotk]         15       316  10.19507        0      0            0.259
## INFO  [08:02:46.466] [bbotk]                                 uhash
## INFO  [08:02:46.466] [bbotk]  2c33233f-4d91-4671-8240-0f49ba8d0550
## INFO  [08:02:46.467] [bbotk] Evaluating 1 configuration(s)
## INFO  [08:02:46.489] [mlr3] Running benchmark with 10 resampling iterations
## INFO  [08:02:46.494] [mlr3] Applying learner 'regr.ranger' on task 'PredictionSet[1:13]' (iter 1/10)
## INFO  [08:02:46.542] [mlr3] Applying learner 'regr.ranger' on task 'PredictionSet[1:13]' (iter 2/10)
## INFO  [08:02:46.587] [mlr3] Applying learner 'regr.ranger' on task 'PredictionSet[1:13]' (iter 3/10)
## INFO  [08:02:46.634] [mlr3] Applying learner 'regr.ranger' on task 'PredictionSet[1:13]' (iter 4/10)
## INFO  [08:02:46.690] [mlr3] Applying learner 'regr.ranger' on task 'PredictionSet[1:13]' (iter 5/10)
## INFO  [08:02:46.739] [mlr3] Applying learner 'regr.ranger' on task 'PredictionSet[1:13]' (iter 6/10)
## INFO  [08:02:46.786] [mlr3] Applying learner 'regr.ranger' on task 'PredictionSet[1:13]' (iter 7/10)
## INFO  [08:02:46.833] [mlr3] Applying learner 'regr.ranger' on task 'PredictionSet[1:13]' (iter 8/10)
## INFO  [08:02:46.880] [mlr3] Applying learner 'regr.ranger' on task 'PredictionSet[1:13]' (iter 9/10)
## INFO  [08:02:46.928] [mlr3] Applying learner 'regr.ranger' on task 'PredictionSet[1:13]' (iter 10/10)
## INFO  [08:02:46.973] [mlr3] Finished benchmark
## INFO  [08:02:47.005] [bbotk] Result of batch 8:
## INFO  [08:02:47.007] [bbotk]  max.depth num.trees regr.rmse warnings errors runtime_learners
## INFO  [08:02:47.007] [bbotk]          8       474  10.25135        0      0            0.362
## INFO  [08:02:47.007] [bbotk]                                 uhash
## INFO  [08:02:47.007] [bbotk]  e02a7607-24c5-4954-bfd2-2570c2d8d717
## INFO  [08:02:47.008] [bbotk] Evaluating 1 configuration(s)
## INFO  [08:02:47.029] [mlr3] Running benchmark with 10 resampling iterations
## INFO  [08:02:47.034] [mlr3] Applying learner 'regr.ranger' on task 'PredictionSet[1:13]' (iter 1/10)
## INFO  [08:02:47.074] [mlr3] Applying learner 'regr.ranger' on task 'PredictionSet[1:13]' (iter 2/10)
## INFO  [08:02:47.113] [mlr3] Applying learner 'regr.ranger' on task 'PredictionSet[1:13]' (iter 3/10)
## INFO  [08:02:47.153] [mlr3] Applying learner 'regr.ranger' on task 'PredictionSet[1:13]' (iter 4/10)
## INFO  [08:02:47.193] [mlr3] Applying learner 'regr.ranger' on task 'PredictionSet[1:13]' (iter 5/10)
## INFO  [08:02:47.234] [mlr3] Applying learner 'regr.ranger' on task 'PredictionSet[1:13]' (iter 6/10)
## INFO  [08:02:47.274] [mlr3] Applying learner 'regr.ranger' on task 'PredictionSet[1:13]' (iter 7/10)
## INFO  [08:02:47.314] [mlr3] Applying learner 'regr.ranger' on task 'PredictionSet[1:13]' (iter 8/10)
## INFO  [08:02:47.354] [mlr3] Applying learner 'regr.ranger' on task 'PredictionSet[1:13]' (iter 9/10)
## INFO  [08:02:47.394] [mlr3] Applying learner 'regr.ranger' on task 'PredictionSet[1:13]' (iter 10/10)
## INFO  [08:02:47.434] [mlr3] Finished benchmark
## INFO  [08:02:47.476] [bbotk] Result of batch 9:
## INFO  [08:02:47.477] [bbotk]  max.depth num.trees regr.rmse warnings errors runtime_learners
## INFO  [08:02:47.477] [bbotk]         19       369  10.22038        0      0            0.298
## INFO  [08:02:47.477] [bbotk]                                 uhash
## INFO  [08:02:47.477] [bbotk]  36fd5816-b194-4ba6-aa66-52a74b5fb218
## INFO  [08:02:47.479] [bbotk] Evaluating 1 configuration(s)
## INFO  [08:02:47.501] [mlr3] Running benchmark with 10 resampling iterations
## INFO  [08:02:47.506] [mlr3] Applying learner 'regr.ranger' on task 'PredictionSet[1:13]' (iter 1/10)
## INFO  [08:02:47.548] [mlr3] Applying learner 'regr.ranger' on task 'PredictionSet[1:13]' (iter 2/10)
## INFO  [08:02:47.592] [mlr3] Applying learner 'regr.ranger' on task 'PredictionSet[1:13]' (iter 3/10)
## INFO  [08:02:47.633] [mlr3] Applying learner 'regr.ranger' on task 'PredictionSet[1:13]' (iter 4/10)
## INFO  [08:02:47.676] [mlr3] Applying learner 'regr.ranger' on task 'PredictionSet[1:13]' (iter 5/10)
## INFO  [08:02:47.717] [mlr3] Applying learner 'regr.ranger' on task 'PredictionSet[1:13]' (iter 6/10)
## INFO  [08:02:47.759] [mlr3] Applying learner 'regr.ranger' on task 'PredictionSet[1:13]' (iter 7/10)
## INFO  [08:02:47.800] [mlr3] Applying learner 'regr.ranger' on task 'PredictionSet[1:13]' (iter 8/10)
## INFO  [08:02:47.842] [mlr3] Applying learner 'regr.ranger' on task 'PredictionSet[1:13]' (iter 9/10)
## INFO  [08:02:47.885] [mlr3] Applying learner 'regr.ranger' on task 'PredictionSet[1:13]' (iter 10/10)
## INFO  [08:02:47.926] [mlr3] Finished benchmark
## INFO  [08:02:47.959] [bbotk] Result of batch 10:
## INFO  [08:02:47.960] [bbotk]  max.depth num.trees regr.rmse warnings errors runtime_learners
## INFO  [08:02:47.960] [bbotk]         15       395  10.22406        0      0            0.317
## INFO  [08:02:47.960] [bbotk]                                 uhash
## INFO  [08:02:47.960] [bbotk]  263c06e8-3d7d-4eb8-a72d-0fefc609a091
## INFO  [08:02:47.966] [bbotk] Finished optimizing after 10 evaluation(s)
## INFO  [08:02:47.966] [bbotk] Result:
## INFO  [08:02:47.967] [bbotk]  max.depth num.trees learner_param_vals  x_domain regr.rmse
## INFO  [08:02:47.967] [bbotk]         15       316          <list[3]> <list[2]>  10.19507
## INFO  [08:02:48.204] [bbotk] Starting to optimize 5 parameter(s) with '<TunerGridSearch>' and '<TerminatorEvals> [n_evals=10, k=0]'
## INFO  [08:02:48.212] [bbotk] Evaluating 1 configuration(s)
## INFO  [08:02:48.227] [mlr3] Running benchmark with 10 resampling iterations
## INFO  [08:02:48.233] [mlr3] Applying learner 'regr.svm' on task 'PredictionSet[1:13]' (iter 1/10)
## INFO  [08:02:48.248] [mlr3] Applying learner 'regr.svm' on task 'PredictionSet[1:13]' (iter 2/10)
## INFO  [08:02:48.265] [mlr3] Applying learner 'regr.svm' on task 'PredictionSet[1:13]' (iter 3/10)
## INFO  [08:02:48.281] [mlr3] Applying learner 'regr.svm' on task 'PredictionSet[1:13]' (iter 4/10)
## INFO  [08:02:48.296] [mlr3] Applying learner 'regr.svm' on task 'PredictionSet[1:13]' (iter 5/10)
## INFO  [08:02:48.311] [mlr3] Applying learner 'regr.svm' on task 'PredictionSet[1:13]' (iter 6/10)
## INFO  [08:02:48.327] [mlr3] Applying learner 'regr.svm' on task 'PredictionSet[1:13]' (iter 7/10)
## INFO  [08:02:48.343] [mlr3] Applying learner 'regr.svm' on task 'PredictionSet[1:13]' (iter 8/10)
## INFO  [08:02:48.367] [mlr3] Applying learner 'regr.svm' on task 'PredictionSet[1:13]' (iter 9/10)
## INFO  [08:02:48.383] [mlr3] Applying learner 'regr.svm' on task 'PredictionSet[1:13]' (iter 10/10)
## INFO  [08:02:48.398] [mlr3] Finished benchmark
## INFO  [08:02:48.428] [bbotk] Result of batch 1:
## INFO  [08:02:48.430] [bbotk]  tolerance    gamma   epsilon           type kernel regr.rmse warnings errors
## INFO  [08:02:48.430] [bbotk]  0.6431429 14.28643 0.6714286 eps-regression radial   13.2019        0      0
## INFO  [08:02:48.430] [bbotk]  runtime_learners                                uhash
## INFO  [08:02:48.430] [bbotk]             0.058 766c0cc8-995e-445a-b320-f5354cc13857
## INFO  [08:02:48.431] [bbotk] Evaluating 1 configuration(s)
## INFO  [08:02:48.446] [mlr3] Running benchmark with 10 resampling iterations
## INFO  [08:02:48.451] [mlr3] Applying learner 'regr.svm' on task 'PredictionSet[1:13]' (iter 1/10)
## INFO  [08:02:48.469] [mlr3] Applying learner 'regr.svm' on task 'PredictionSet[1:13]' (iter 2/10)
## INFO  [08:02:48.489] [mlr3] Applying learner 'regr.svm' on task 'PredictionSet[1:13]' (iter 3/10)
## INFO  [08:02:48.508] [mlr3] Applying learner 'regr.svm' on task 'PredictionSet[1:13]' (iter 4/10)
## INFO  [08:02:48.525] [mlr3] Applying learner 'regr.svm' on task 'PredictionSet[1:13]' (iter 5/10)
## INFO  [08:02:48.543] [mlr3] Applying learner 'regr.svm' on task 'PredictionSet[1:13]' (iter 6/10)
## INFO  [08:02:48.563] [mlr3] Applying learner 'regr.svm' on task 'PredictionSet[1:13]' (iter 7/10)
## INFO  [08:02:48.582] [mlr3] Applying learner 'regr.svm' on task 'PredictionSet[1:13]' (iter 8/10)
## INFO  [08:02:48.600] [mlr3] Applying learner 'regr.svm' on task 'PredictionSet[1:13]' (iter 9/10)
## INFO  [08:02:48.619] [mlr3] Applying learner 'regr.svm' on task 'PredictionSet[1:13]' (iter 10/10)
## INFO  [08:02:48.638] [mlr3] Finished benchmark
## INFO  [08:02:48.669] [bbotk] Result of batch 2:
## INFO  [08:02:48.671] [bbotk]  tolerance    gamma   epsilon           type  kernel regr.rmse warnings errors
## INFO  [08:02:48.671] [bbotk]  0.3220714 17.85779 0.7857143 eps-regression sigmoid   461.622        0      0
## INFO  [08:02:48.671] [bbotk]  runtime_learners                                uhash
## INFO  [08:02:48.671] [bbotk]             0.089 fa4be86d-31cc-4f10-a6df-9fba445c7715
## INFO  [08:02:48.672] [bbotk] Evaluating 1 configuration(s)
## INFO  [08:02:48.688] [mlr3] Running benchmark with 10 resampling iterations
## INFO  [08:02:48.693] [mlr3] Applying learner 'regr.svm' on task 'PredictionSet[1:13]' (iter 1/10)
## INFO  [08:02:48.719] [mlr3] Applying learner 'regr.svm' on task 'PredictionSet[1:13]' (iter 2/10)
## INFO  [08:02:48.737] [mlr3] Applying learner 'regr.svm' on task 'PredictionSet[1:13]' (iter 3/10)
## INFO  [08:02:48.754] [mlr3] Applying learner 'regr.svm' on task 'PredictionSet[1:13]' (iter 4/10)
## INFO  [08:02:48.770] [mlr3] Applying learner 'regr.svm' on task 'PredictionSet[1:13]' (iter 5/10)
## INFO  [08:02:48.789] [mlr3] Applying learner 'regr.svm' on task 'PredictionSet[1:13]' (iter 6/10)
## INFO  [08:02:48.806] [mlr3] Applying learner 'regr.svm' on task 'PredictionSet[1:13]' (iter 7/10)
## INFO  [08:02:48.823] [mlr3] Applying learner 'regr.svm' on task 'PredictionSet[1:13]' (iter 8/10)
## INFO  [08:02:48.840] [mlr3] Applying learner 'regr.svm' on task 'PredictionSet[1:13]' (iter 9/10)
## INFO  [08:02:48.858] [mlr3] Applying learner 'regr.svm' on task 'PredictionSet[1:13]' (iter 10/10)
## INFO  [08:02:48.875] [mlr3] Finished benchmark
## INFO  [08:02:48.906] [bbotk] Result of batch 3:
## INFO  [08:02:48.907] [bbotk]   tolerance    gamma   epsilon           type kernel regr.rmse warnings errors
## INFO  [08:02:48.907] [bbotk]  0.06521429 3.572357 0.2714286 eps-regression radial  10.52345        0      0
## INFO  [08:02:48.907] [bbotk]  runtime_learners                                uhash
## INFO  [08:02:48.907] [bbotk]             0.079 0642f562-d3b7-4c5e-a35e-7756e475e50a
## INFO  [08:02:48.909] [bbotk] Evaluating 1 configuration(s)
## INFO  [08:02:48.924] [mlr3] Running benchmark with 10 resampling iterations
## INFO  [08:02:48.930] [mlr3] Applying learner 'regr.svm' on task 'PredictionSet[1:13]' (iter 1/10)
## INFO  [08:02:48.949] [mlr3] Applying learner 'regr.svm' on task 'PredictionSet[1:13]' (iter 2/10)
## INFO  [08:02:48.966] [mlr3] Applying learner 'regr.svm' on task 'PredictionSet[1:13]' (iter 3/10)
## INFO  [08:02:48.983] [mlr3] Applying learner 'regr.svm' on task 'PredictionSet[1:13]' (iter 4/10)
## INFO  [08:02:49.001] [mlr3] Applying learner 'regr.svm' on task 'PredictionSet[1:13]' (iter 5/10)
## INFO  [08:02:49.020] [mlr3] Applying learner 'regr.svm' on task 'PredictionSet[1:13]' (iter 6/10)
## INFO  [08:02:49.037] [mlr3] Applying learner 'regr.svm' on task 'PredictionSet[1:13]' (iter 7/10)
## INFO  [08:02:49.055] [mlr3] Applying learner 'regr.svm' on task 'PredictionSet[1:13]' (iter 8/10)
## INFO  [08:02:49.081] [mlr3] Applying learner 'regr.svm' on task 'PredictionSet[1:13]' (iter 9/10)
## INFO  [08:02:49.100] [mlr3] Applying learner 'regr.svm' on task 'PredictionSet[1:13]' (iter 10/10)
## INFO  [08:02:49.118] [mlr3] Finished benchmark
## INFO  [08:02:49.148] [bbotk] Result of batch 4:
## INFO  [08:02:49.150] [bbotk]   tolerance    gamma   epsilon           type kernel regr.rmse warnings errors
## INFO  [08:02:49.150] [bbotk]  0.06521429 17.85779 0.1571429 eps-regression radial  10.70608        0      0
## INFO  [08:02:49.150] [bbotk]  runtime_learners                                uhash
## INFO  [08:02:49.150] [bbotk]             0.077 24ba13b2-d516-4d39-bdc3-8ba719842614
## INFO  [08:02:49.151] [bbotk] Evaluating 1 configuration(s)
## INFO  [08:02:49.168] [mlr3] Running benchmark with 10 resampling iterations
## INFO  [08:02:49.173] [mlr3] Applying learner 'regr.svm' on task 'PredictionSet[1:13]' (iter 1/10)
## INFO  [08:02:49.188] [mlr3] Applying learner 'regr.svm' on task 'PredictionSet[1:13]' (iter 2/10)
## INFO  [08:02:49.203] [mlr3] Applying learner 'regr.svm' on task 'PredictionSet[1:13]' (iter 3/10)
## INFO  [08:02:49.218] [mlr3] Applying learner 'regr.svm' on task 'PredictionSet[1:13]' (iter 4/10)
## INFO  [08:02:49.236] [mlr3] Applying learner 'regr.svm' on task 'PredictionSet[1:13]' (iter 5/10)
## INFO  [08:02:49.251] [mlr3] Applying learner 'regr.svm' on task 'PredictionSet[1:13]' (iter 6/10)
## INFO  [08:02:49.267] [mlr3] Applying learner 'regr.svm' on task 'PredictionSet[1:13]' (iter 7/10)
## INFO  [08:02:49.282] [mlr3] Applying learner 'regr.svm' on task 'PredictionSet[1:13]' (iter 8/10)
## INFO  [08:02:49.297] [mlr3] Applying learner 'regr.svm' on task 'PredictionSet[1:13]' (iter 9/10)
## INFO  [08:02:49.314] [mlr3] Applying learner 'regr.svm' on task 'PredictionSet[1:13]' (iter 10/10)
## INFO  [08:02:49.330] [mlr3] Finished benchmark
## INFO  [08:02:49.360] [bbotk] Result of batch 5:
## INFO  [08:02:49.362] [bbotk]  tolerance    gamma   epsilon           type kernel regr.rmse warnings errors
## INFO  [08:02:49.362] [bbotk]  0.7715714 3.572357 0.6142857 eps-regression radial  12.30581        0      0
## INFO  [08:02:49.362] [bbotk]  runtime_learners                                uhash
## INFO  [08:02:49.362] [bbotk]             0.059 514ec915-3008-4d16-a877-6429c2e586bb
## INFO  [08:02:49.364] [bbotk] Evaluating 1 configuration(s)
## INFO  [08:02:49.379] [mlr3] Running benchmark with 10 resampling iterations
## INFO  [08:02:49.385] [mlr3] Applying learner 'regr.svm' on task 'PredictionSet[1:13]' (iter 1/10)
## INFO  [08:02:49.410] [mlr3] Applying learner 'regr.svm' on task 'PredictionSet[1:13]' (iter 2/10)
## INFO  [08:02:49.426] [mlr3] Applying learner 'regr.svm' on task 'PredictionSet[1:13]' (iter 3/10)
## INFO  [08:02:49.441] [mlr3] Applying learner 'regr.svm' on task 'PredictionSet[1:13]' (iter 4/10)
## INFO  [08:02:49.458] [mlr3] Applying learner 'regr.svm' on task 'PredictionSet[1:13]' (iter 5/10)
## INFO  [08:02:49.474] [mlr3] Applying learner 'regr.svm' on task 'PredictionSet[1:13]' (iter 6/10)
## INFO  [08:02:49.490] [mlr3] Applying learner 'regr.svm' on task 'PredictionSet[1:13]' (iter 7/10)
## INFO  [08:02:49.506] [mlr3] Applying learner 'regr.svm' on task 'PredictionSet[1:13]' (iter 8/10)
## INFO  [08:02:49.521] [mlr3] Applying learner 'regr.svm' on task 'PredictionSet[1:13]' (iter 9/10)
## INFO  [08:02:49.538] [mlr3] Applying learner 'regr.svm' on task 'PredictionSet[1:13]' (iter 10/10)
## INFO  [08:02:49.554] [mlr3] Finished benchmark
## INFO  [08:02:49.584] [bbotk] Result of batch 6:
## INFO  [08:02:49.586] [bbotk]  tolerance    gamma epsilon           type kernel regr.rmse warnings errors
## INFO  [08:02:49.586] [bbotk]  0.2578571 35.71457     0.9 eps-regression radial  14.06499        0      0
## INFO  [08:02:49.586] [bbotk]  runtime_learners                                uhash
## INFO  [08:02:49.586] [bbotk]             0.068 eaff6a41-9499-4a5d-a87e-22800c0ce7a6
## INFO  [08:02:49.587] [bbotk] Evaluating 1 configuration(s)
## INFO  [08:02:49.603] [mlr3] Running benchmark with 10 resampling iterations
## INFO  [08:02:49.609] [mlr3] Applying learner 'regr.svm' on task 'PredictionSet[1:13]' (iter 1/10)
## INFO  [08:02:49.625] [mlr3] Applying learner 'regr.svm' on task 'PredictionSet[1:13]' (iter 2/10)
## INFO  [08:02:49.641] [mlr3] Applying learner 'regr.svm' on task 'PredictionSet[1:13]' (iter 3/10)
## INFO  [08:02:49.657] [mlr3] Applying learner 'regr.svm' on task 'PredictionSet[1:13]' (iter 4/10)
## INFO  [08:02:49.672] [mlr3] Applying learner 'regr.svm' on task 'PredictionSet[1:13]' (iter 5/10)
## INFO  [08:02:49.690] [mlr3] Applying learner 'regr.svm' on task 'PredictionSet[1:13]' (iter 6/10)
## INFO  [08:02:49.706] [mlr3] Applying learner 'regr.svm' on task 'PredictionSet[1:13]' (iter 7/10)
## INFO  [08:02:49.721] [mlr3] Applying learner 'regr.svm' on task 'PredictionSet[1:13]' (iter 8/10)
## INFO  [08:02:49.745] [mlr3] Applying learner 'regr.svm' on task 'PredictionSet[1:13]' (iter 9/10)
## INFO  [08:02:49.763] [mlr3] Applying learner 'regr.svm' on task 'PredictionSet[1:13]' (iter 10/10)
## INFO  [08:02:49.779] [mlr3] Finished benchmark
## INFO  [08:02:49.810] [bbotk] Result of batch 7:
## INFO  [08:02:49.811] [bbotk]  tolerance gamma   epsilon           type  kernel regr.rmse warnings errors
## INFO  [08:02:49.811] [bbotk]  0.8357857 0.001 0.5571429 eps-regression sigmoid  13.47823        0      0
## INFO  [08:02:49.811] [bbotk]  runtime_learners                                uhash
## INFO  [08:02:49.811] [bbotk]              0.07 29cbcfcc-aa2d-4f07-a27b-a3e0653957aa
## INFO  [08:02:49.813] [bbotk] Evaluating 1 configuration(s)
## INFO  [08:02:49.828] [mlr3] Running benchmark with 10 resampling iterations
## INFO  [08:02:49.834] [mlr3] Applying learner 'regr.svm' on task 'PredictionSet[1:13]' (iter 1/10)
## INFO  [08:02:49.850] [mlr3] Applying learner 'regr.svm' on task 'PredictionSet[1:13]' (iter 2/10)
## INFO  [08:02:49.865] [mlr3] Applying learner 'regr.svm' on task 'PredictionSet[1:13]' (iter 3/10)
## INFO  [08:02:49.880] [mlr3] Applying learner 'regr.svm' on task 'PredictionSet[1:13]' (iter 4/10)
## INFO  [08:02:49.894] [mlr3] Applying learner 'regr.svm' on task 'PredictionSet[1:13]' (iter 5/10)
## INFO  [08:02:49.911] [mlr3] Applying learner 'regr.svm' on task 'PredictionSet[1:13]' (iter 6/10)
## INFO  [08:02:49.927] [mlr3] Applying learner 'regr.svm' on task 'PredictionSet[1:13]' (iter 7/10)
## INFO  [08:02:49.942] [mlr3] Applying learner 'regr.svm' on task 'PredictionSet[1:13]' (iter 8/10)
## INFO  [08:02:49.957] [mlr3] Applying learner 'regr.svm' on task 'PredictionSet[1:13]' (iter 9/10)
## INFO  [08:02:49.972] [mlr3] Applying learner 'regr.svm' on task 'PredictionSet[1:13]' (iter 10/10)
## INFO  [08:02:49.989] [mlr3] Finished benchmark
## INFO  [08:02:50.020] [bbotk] Result of batch 8:
## INFO  [08:02:50.022] [bbotk]  tolerance gamma   epsilon           type kernel regr.rmse warnings errors
## INFO  [08:02:50.022] [bbotk]  0.8357857 0.001 0.7857143 eps-regression radial  14.13287        0      0
## INFO  [08:02:50.022] [bbotk]  runtime_learners                                uhash
## INFO  [08:02:50.022] [bbotk]             0.052 8edb7b7f-ac6a-44e7-a786-120d12118f9e
## INFO  [08:02:50.023] [bbotk] Evaluating 1 configuration(s)
## INFO  [08:02:50.038] [mlr3] Running benchmark with 10 resampling iterations
## INFO  [08:02:50.051] [mlr3] Applying learner 'regr.svm' on task 'PredictionSet[1:13]' (iter 1/10)
## INFO  [08:02:50.071] [mlr3] Applying learner 'regr.svm' on task 'PredictionSet[1:13]' (iter 2/10)
## INFO  [08:02:50.090] [mlr3] Applying learner 'regr.svm' on task 'PredictionSet[1:13]' (iter 3/10)
## INFO  [08:02:50.107] [mlr3] Applying learner 'regr.svm' on task 'PredictionSet[1:13]' (iter 4/10)
## INFO  [08:02:50.125] [mlr3] Applying learner 'regr.svm' on task 'PredictionSet[1:13]' (iter 5/10)
## INFO  [08:02:50.144] [mlr3] Applying learner 'regr.svm' on task 'PredictionSet[1:13]' (iter 6/10)
## INFO  [08:02:50.162] [mlr3] Applying learner 'regr.svm' on task 'PredictionSet[1:13]' (iter 7/10)
## INFO  [08:02:50.180] [mlr3] Applying learner 'regr.svm' on task 'PredictionSet[1:13]' (iter 8/10)
## INFO  [08:02:50.198] [mlr3] Applying learner 'regr.svm' on task 'PredictionSet[1:13]' (iter 9/10)
## INFO  [08:02:50.217] [mlr3] Applying learner 'regr.svm' on task 'PredictionSet[1:13]' (iter 10/10)
## INFO  [08:02:50.234] [mlr3] Finished benchmark
## INFO  [08:02:50.265] [bbotk] Result of batch 9:
## INFO  [08:02:50.267] [bbotk]  tolerance gamma   epsilon           type  kernel regr.rmse warnings errors
## INFO  [08:02:50.267] [bbotk]  0.7715714    50 0.3857143 eps-regression sigmoid  459.0582        0      0
## INFO  [08:02:50.267] [bbotk]  runtime_learners                                uhash
## INFO  [08:02:50.267] [bbotk]             0.081 2b9dbdd0-d8c6-4426-8fd4-c6a65d6399e0
## INFO  [08:02:50.268] [bbotk] Evaluating 1 configuration(s)
## INFO  [08:02:50.285] [mlr3] Running benchmark with 10 resampling iterations
## INFO  [08:02:50.291] [mlr3] Applying learner 'regr.svm' on task 'PredictionSet[1:13]' (iter 1/10)
## INFO  [08:02:50.307] [mlr3] Applying learner 'regr.svm' on task 'PredictionSet[1:13]' (iter 2/10)
## INFO  [08:02:50.324] [mlr3] Applying learner 'regr.svm' on task 'PredictionSet[1:13]' (iter 3/10)
## INFO  [08:02:50.340] [mlr3] Applying learner 'regr.svm' on task 'PredictionSet[1:13]' (iter 4/10)
## INFO  [08:02:50.358] [mlr3] Applying learner 'regr.svm' on task 'PredictionSet[1:13]' (iter 5/10)
## INFO  [08:02:50.375] [mlr3] Applying learner 'regr.svm' on task 'PredictionSet[1:13]' (iter 6/10)
## INFO  [08:02:50.392] [mlr3] Applying learner 'regr.svm' on task 'PredictionSet[1:13]' (iter 7/10)
## INFO  [08:02:50.417] [mlr3] Applying learner 'regr.svm' on task 'PredictionSet[1:13]' (iter 8/10)
## INFO  [08:02:50.435] [mlr3] Applying learner 'regr.svm' on task 'PredictionSet[1:13]' (iter 9/10)
## INFO  [08:02:50.453] [mlr3] Applying learner 'regr.svm' on task 'PredictionSet[1:13]' (iter 10/10)
## INFO  [08:02:50.469] [mlr3] Finished benchmark
## INFO  [08:02:50.499] [bbotk] Result of batch 10:
## INFO  [08:02:50.501] [bbotk]  tolerance   gamma   epsilon           type kernel regr.rmse warnings errors
## INFO  [08:02:50.501] [bbotk]  0.5789286 25.0005 0.1571429 eps-regression radial  10.95523        0      0
## INFO  [08:02:50.501] [bbotk]  runtime_learners                                uhash
## INFO  [08:02:50.501] [bbotk]             0.078 68b2d82c-c90c-4e35-8195-912eb831d2b2
## INFO  [08:02:50.507] [bbotk] Finished optimizing after 10 evaluation(s)
## INFO  [08:02:50.507] [bbotk] Result:
## INFO  [08:02:50.509] [bbotk]   tolerance    gamma   epsilon           type kernel learner_param_vals
## INFO  [08:02:50.509] [bbotk]  0.06521429 3.572357 0.2714286 eps-regression radial          <list[5]>
## INFO  [08:02:50.509] [bbotk]   x_domain regr.rmse
## INFO  [08:02:50.509] [bbotk]  <list[5]>  10.52345
## INFO  [08:02:50.648] [bbotk] Starting to optimize 3 parameter(s) with '<TunerGridSearch>' and '<TerminatorEvals> [n_evals=10, k=0]'
## INFO  [08:02:50.652] [bbotk] Evaluating 1 configuration(s)
## INFO  [08:02:50.664] [mlr3] Running benchmark with 10 resampling iterations
## INFO  [08:02:50.669] [mlr3] Applying learner 'regr.kknn' on task 'PredictionSet[1:13]' (iter 1/10)
## INFO  [08:02:50.689] [mlr3] Applying learner 'regr.kknn' on task 'PredictionSet[1:13]' (iter 2/10)
## INFO  [08:02:50.717] [mlr3] Applying learner 'regr.kknn' on task 'PredictionSet[1:13]' (iter 3/10)
## INFO  [08:02:50.739] [mlr3] Applying learner 'regr.kknn' on task 'PredictionSet[1:13]' (iter 4/10)
## INFO  [08:02:50.759] [mlr3] Applying learner 'regr.kknn' on task 'PredictionSet[1:13]' (iter 5/10)
## INFO  [08:02:50.779] [mlr3] Applying learner 'regr.kknn' on task 'PredictionSet[1:13]' (iter 6/10)
## INFO  [08:02:50.800] [mlr3] Applying learner 'regr.kknn' on task 'PredictionSet[1:13]' (iter 7/10)
## INFO  [08:02:50.822] [mlr3] Applying learner 'regr.kknn' on task 'PredictionSet[1:13]' (iter 8/10)
## INFO  [08:02:50.842] [mlr3] Applying learner 'regr.kknn' on task 'PredictionSet[1:13]' (iter 9/10)
## INFO  [08:02:50.862] [mlr3] Applying learner 'regr.kknn' on task 'PredictionSet[1:13]' (iter 10/10)
## INFO  [08:02:50.882] [mlr3] Finished benchmark
## INFO  [08:02:50.912] [bbotk] Result of batch 1:
## INFO  [08:02:50.913] [bbotk]  k distance       kernel regr.rmse warnings errors runtime_learners
## INFO  [08:02:50.913] [bbotk]  9 18.57857 epanechnikov  9.133404        0      0            0.104
## INFO  [08:02:50.913] [bbotk]                                 uhash
## INFO  [08:02:50.913] [bbotk]  5c47b827-259e-4aff-975a-8e6ae09157c0
## INFO  [08:02:50.915] [bbotk] Evaluating 1 configuration(s)
## INFO  [08:02:50.925] [mlr3] Running benchmark with 10 resampling iterations
## INFO  [08:02:50.930] [mlr3] Applying learner 'regr.kknn' on task 'PredictionSet[1:13]' (iter 1/10)
## INFO  [08:02:50.951] [mlr3] Applying learner 'regr.kknn' on task 'PredictionSet[1:13]' (iter 2/10)
## INFO  [08:02:50.973] [mlr3] Applying learner 'regr.kknn' on task 'PredictionSet[1:13]' (iter 3/10)
## INFO  [08:02:50.993] [mlr3] Applying learner 'regr.kknn' on task 'PredictionSet[1:13]' (iter 4/10)
## INFO  [08:02:51.013] [mlr3] Applying learner 'regr.kknn' on task 'PredictionSet[1:13]' (iter 5/10)
## INFO  [08:02:51.035] [mlr3] Applying learner 'regr.kknn' on task 'PredictionSet[1:13]' (iter 6/10)
## INFO  [08:02:51.063] [mlr3] Applying learner 'regr.kknn' on task 'PredictionSet[1:13]' (iter 7/10)
## INFO  [08:02:51.084] [mlr3] Applying learner 'regr.kknn' on task 'PredictionSet[1:13]' (iter 8/10)
## INFO  [08:02:51.104] [mlr3] Applying learner 'regr.kknn' on task 'PredictionSet[1:13]' (iter 9/10)
## INFO  [08:02:51.126] [mlr3] Applying learner 'regr.kknn' on task 'PredictionSet[1:13]' (iter 10/10)
## INFO  [08:02:51.145] [mlr3] Finished benchmark
## INFO  [08:02:51.176] [bbotk] Result of batch 2:
## INFO  [08:02:51.178] [bbotk]  k distance   kernel regr.rmse warnings errors runtime_learners
## INFO  [08:02:51.178] [bbotk]  6 4.364286 gaussian  8.574331        0      0            0.109
## INFO  [08:02:51.178] [bbotk]                                 uhash
## INFO  [08:02:51.178] [bbotk]  3986d68b-921b-490d-877e-927a2ef7da1f
## INFO  [08:02:51.179] [bbotk] Evaluating 1 configuration(s)
## INFO  [08:02:51.191] [mlr3] Running benchmark with 10 resampling iterations
## INFO  [08:02:51.196] [mlr3] Applying learner 'regr.kknn' on task 'PredictionSet[1:13]' (iter 1/10)
## INFO  [08:02:51.216] [mlr3] Applying learner 'regr.kknn' on task 'PredictionSet[1:13]' (iter 2/10)
## INFO  [08:02:51.235] [mlr3] Applying learner 'regr.kknn' on task 'PredictionSet[1:13]' (iter 3/10)
## INFO  [08:02:51.256] [mlr3] Applying learner 'regr.kknn' on task 'PredictionSet[1:13]' (iter 4/10)
## INFO  [08:02:51.278] [mlr3] Applying learner 'regr.kknn' on task 'PredictionSet[1:13]' (iter 5/10)
## INFO  [08:02:51.298] [mlr3] Applying learner 'regr.kknn' on task 'PredictionSet[1:13]' (iter 6/10)
## INFO  [08:02:51.318] [mlr3] Applying learner 'regr.kknn' on task 'PredictionSet[1:13]' (iter 7/10)
## INFO  [08:02:51.340] [mlr3] Applying learner 'regr.kknn' on task 'PredictionSet[1:13]' (iter 8/10)
## INFO  [08:02:51.361] [mlr3] Applying learner 'regr.kknn' on task 'PredictionSet[1:13]' (iter 9/10)
## INFO  [08:02:51.381] [mlr3] Applying learner 'regr.kknn' on task 'PredictionSet[1:13]' (iter 10/10)
## INFO  [08:02:51.408] [mlr3] Finished benchmark
## INFO  [08:02:51.441] [bbotk] Result of batch 3:
## INFO  [08:02:51.443] [bbotk]  k distance    kernel regr.rmse warnings errors runtime_learners
## INFO  [08:02:51.443] [bbotk]  5    10.05 triweight  9.883541        0      0            0.099
## INFO  [08:02:51.443] [bbotk]                                 uhash
## INFO  [08:02:51.443] [bbotk]  bbbb0d63-370c-449d-a74f-5b42cd971e8e
## INFO  [08:02:51.444] [bbotk] Evaluating 1 configuration(s)
## INFO  [08:02:51.455] [mlr3] Running benchmark with 10 resampling iterations
## INFO  [08:02:51.460] [mlr3] Applying learner 'regr.kknn' on task 'PredictionSet[1:13]' (iter 1/10)
## INFO  [08:02:51.480] [mlr3] Applying learner 'regr.kknn' on task 'PredictionSet[1:13]' (iter 2/10)
## INFO  [08:02:51.502] [mlr3] Applying learner 'regr.kknn' on task 'PredictionSet[1:13]' (iter 3/10)
## INFO  [08:02:51.522] [mlr3] Applying learner 'regr.kknn' on task 'PredictionSet[1:13]' (iter 4/10)
## INFO  [08:02:51.542] [mlr3] Applying learner 'regr.kknn' on task 'PredictionSet[1:13]' (iter 5/10)
## INFO  [08:02:51.564] [mlr3] Applying learner 'regr.kknn' on task 'PredictionSet[1:13]' (iter 6/10)
## INFO  [08:02:51.584] [mlr3] Applying learner 'regr.kknn' on task 'PredictionSet[1:13]' (iter 7/10)
## INFO  [08:02:51.604] [mlr3] Applying learner 'regr.kknn' on task 'PredictionSet[1:13]' (iter 8/10)
## INFO  [08:02:51.625] [mlr3] Applying learner 'regr.kknn' on task 'PredictionSet[1:13]' (iter 9/10)
## INFO  [08:02:51.648] [mlr3] Applying learner 'regr.kknn' on task 'PredictionSet[1:13]' (iter 10/10)
## INFO  [08:02:51.668] [mlr3] Finished benchmark
## INFO  [08:02:51.699] [bbotk] Result of batch 4:
## INFO  [08:02:51.701] [bbotk]   k distance kernel regr.rmse warnings errors runtime_learners
## INFO  [08:02:51.701] [bbotk]  19 18.57857    cos  9.038889        0      0            0.106
## INFO  [08:02:51.701] [bbotk]                                 uhash
## INFO  [08:02:51.701] [bbotk]  d8fdf866-3201-4acf-931e-51585dfa1420
## INFO  [08:02:51.702] [bbotk] Evaluating 1 configuration(s)
## INFO  [08:02:51.714] [mlr3] Running benchmark with 10 resampling iterations
## INFO  [08:02:51.719] [mlr3] Applying learner 'regr.kknn' on task 'PredictionSet[1:13]' (iter 1/10)
## INFO  [08:02:51.746] [mlr3] Applying learner 'regr.kknn' on task 'PredictionSet[1:13]' (iter 2/10)
## INFO  [08:02:51.767] [mlr3] Applying learner 'regr.kknn' on task 'PredictionSet[1:13]' (iter 3/10)
## INFO  [08:02:51.789] [mlr3] Applying learner 'regr.kknn' on task 'PredictionSet[1:13]' (iter 4/10)
## INFO  [08:02:51.809] [mlr3] Applying learner 'regr.kknn' on task 'PredictionSet[1:13]' (iter 5/10)
## INFO  [08:02:51.829] [mlr3] Applying learner 'regr.kknn' on task 'PredictionSet[1:13]' (iter 6/10)
## INFO  [08:02:51.850] [mlr3] Applying learner 'regr.kknn' on task 'PredictionSet[1:13]' (iter 7/10)
## INFO  [08:02:51.872] [mlr3] Applying learner 'regr.kknn' on task 'PredictionSet[1:13]' (iter 8/10)
## INFO  [08:02:51.892] [mlr3] Applying learner 'regr.kknn' on task 'PredictionSet[1:13]' (iter 9/10)
## INFO  [08:02:51.912] [mlr3] Applying learner 'regr.kknn' on task 'PredictionSet[1:13]' (iter 10/10)
## INFO  [08:02:51.933] [mlr3] Finished benchmark
## INFO  [08:02:51.965] [bbotk] Result of batch 5:
## INFO  [08:02:51.966] [bbotk]   k distance kernel regr.rmse warnings errors runtime_learners
## INFO  [08:02:51.966] [bbotk]  15 18.57857    cos  8.836942        0      0            0.107
## INFO  [08:02:51.966] [bbotk]                                 uhash
## INFO  [08:02:51.966] [bbotk]  f9224c70-c404-42d8-b5cc-e71a0b3a32b6
## INFO  [08:02:51.968] [bbotk] Evaluating 1 configuration(s)
## INFO  [08:02:51.978] [mlr3] Running benchmark with 10 resampling iterations
## INFO  [08:02:51.983] [mlr3] Applying learner 'regr.kknn' on task 'PredictionSet[1:13]' (iter 1/10)
## INFO  [08:02:52.004] [mlr3] Applying learner 'regr.kknn' on task 'PredictionSet[1:13]' (iter 2/10)
## INFO  [08:02:52.026] [mlr3] Applying learner 'regr.kknn' on task 'PredictionSet[1:13]' (iter 3/10)
## INFO  [08:02:52.046] [mlr3] Applying learner 'regr.kknn' on task 'PredictionSet[1:13]' (iter 4/10)
## INFO  [08:02:52.067] [mlr3] Applying learner 'regr.kknn' on task 'PredictionSet[1:13]' (iter 5/10)
## INFO  [08:02:52.096] [mlr3] Applying learner 'regr.kknn' on task 'PredictionSet[1:13]' (iter 6/10)
## INFO  [08:02:52.118] [mlr3] Applying learner 'regr.kknn' on task 'PredictionSet[1:13]' (iter 7/10)
## INFO  [08:02:52.138] [mlr3] Applying learner 'regr.kknn' on task 'PredictionSet[1:13]' (iter 8/10)
## INFO  [08:02:52.159] [mlr3] Applying learner 'regr.kknn' on task 'PredictionSet[1:13]' (iter 9/10)
## INFO  [08:02:52.180] [mlr3] Applying learner 'regr.kknn' on task 'PredictionSet[1:13]' (iter 10/10)
## INFO  [08:02:52.200] [mlr3] Finished benchmark
## INFO  [08:02:52.232] [bbotk] Result of batch 6:
## INFO  [08:02:52.234] [bbotk]   k distance   kernel regr.rmse warnings errors runtime_learners
## INFO  [08:02:52.234] [bbotk]  20 2.942857 biweight  8.424435        0      0            0.106
## INFO  [08:02:52.234] [bbotk]                                 uhash
## INFO  [08:02:52.234] [bbotk]  aff19a8a-64f1-4ce9-9aea-59d926319463
## INFO  [08:02:52.235] [bbotk] Evaluating 1 configuration(s)
## INFO  [08:02:52.246] [mlr3] Running benchmark with 10 resampling iterations
## INFO  [08:02:52.251] [mlr3] Applying learner 'regr.kknn' on task 'PredictionSet[1:13]' (iter 1/10)
## INFO  [08:02:52.272] [mlr3] Applying learner 'regr.kknn' on task 'PredictionSet[1:13]' (iter 2/10)
## INFO  [08:02:52.292] [mlr3] Applying learner 'regr.kknn' on task 'PredictionSet[1:13]' (iter 3/10)
## INFO  [08:02:52.314] [mlr3] Applying learner 'regr.kknn' on task 'PredictionSet[1:13]' (iter 4/10)
## INFO  [08:02:52.335] [mlr3] Applying learner 'regr.kknn' on task 'PredictionSet[1:13]' (iter 5/10)
## INFO  [08:02:52.356] [mlr3] Applying learner 'regr.kknn' on task 'PredictionSet[1:13]' (iter 6/10)
## INFO  [08:02:52.377] [mlr3] Applying learner 'regr.kknn' on task 'PredictionSet[1:13]' (iter 7/10)
## INFO  [08:02:52.400] [mlr3] Applying learner 'regr.kknn' on task 'PredictionSet[1:13]' (iter 8/10)
## INFO  [08:02:52.420] [mlr3] Applying learner 'regr.kknn' on task 'PredictionSet[1:13]' (iter 9/10)
## INFO  [08:02:52.453] [mlr3] Applying learner 'regr.kknn' on task 'PredictionSet[1:13]' (iter 10/10)
## INFO  [08:02:52.476] [mlr3] Finished benchmark
## INFO  [08:02:52.507] [bbotk] Result of batch 7:
## INFO  [08:02:52.509] [bbotk]   k distance kernel regr.rmse warnings errors runtime_learners
## INFO  [08:02:52.509] [bbotk]  18 15.73571   rank  8.240982        0      0            0.118
## INFO  [08:02:52.509] [bbotk]                                 uhash
## INFO  [08:02:52.509] [bbotk]  e5772cde-9d8f-422c-9947-49976069b2c9
## INFO  [08:02:52.510] [bbotk] Evaluating 1 configuration(s)
## INFO  [08:02:52.521] [mlr3] Running benchmark with 10 resampling iterations
## INFO  [08:02:52.526] [mlr3] Applying learner 'regr.kknn' on task 'PredictionSet[1:13]' (iter 1/10)
## INFO  [08:02:52.548] [mlr3] Applying learner 'regr.kknn' on task 'PredictionSet[1:13]' (iter 2/10)
## INFO  [08:02:52.568] [mlr3] Applying learner 'regr.kknn' on task 'PredictionSet[1:13]' (iter 3/10)
## INFO  [08:02:52.588] [mlr3] Applying learner 'regr.kknn' on task 'PredictionSet[1:13]' (iter 4/10)
## INFO  [08:02:52.610] [mlr3] Applying learner 'regr.kknn' on task 'PredictionSet[1:13]' (iter 5/10)
## INFO  [08:02:52.630] [mlr3] Applying learner 'regr.kknn' on task 'PredictionSet[1:13]' (iter 6/10)
## INFO  [08:02:52.650] [mlr3] Applying learner 'regr.kknn' on task 'PredictionSet[1:13]' (iter 7/10)
## INFO  [08:02:52.671] [mlr3] Applying learner 'regr.kknn' on task 'PredictionSet[1:13]' (iter 8/10)
## INFO  [08:02:52.693] [mlr3] Applying learner 'regr.kknn' on task 'PredictionSet[1:13]' (iter 9/10)
## INFO  [08:02:52.714] [mlr3] Applying learner 'regr.kknn' on task 'PredictionSet[1:13]' (iter 10/10)
## INFO  [08:02:52.733] [mlr3] Finished benchmark
## INFO  [08:02:52.766] [bbotk] Result of batch 8:
## INFO  [08:02:52.768] [bbotk]   k distance kernel regr.rmse warnings errors runtime_learners
## INFO  [08:02:52.768] [bbotk]  10 8.628571    cos  9.180605        0      0            0.105
## INFO  [08:02:52.768] [bbotk]                                 uhash
## INFO  [08:02:52.768] [bbotk]  9c6aa111-67b6-48a9-8964-355f63a006c8
## INFO  [08:02:52.769] [bbotk] Evaluating 1 configuration(s)
## INFO  [08:02:52.788] [mlr3] Running benchmark with 10 resampling iterations
## INFO  [08:02:52.793] [mlr3] Applying learner 'regr.kknn' on task 'PredictionSet[1:13]' (iter 1/10)
## INFO  [08:02:52.813] [mlr3] Applying learner 'regr.kknn' on task 'PredictionSet[1:13]' (iter 2/10)
## INFO  [08:02:52.834] [mlr3] Applying learner 'regr.kknn' on task 'PredictionSet[1:13]' (iter 3/10)
## INFO  [08:02:52.855] [mlr3] Applying learner 'regr.kknn' on task 'PredictionSet[1:13]' (iter 4/10)
## INFO  [08:02:52.876] [mlr3] Applying learner 'regr.kknn' on task 'PredictionSet[1:13]' (iter 5/10)
## INFO  [08:02:52.896] [mlr3] Applying learner 'regr.kknn' on task 'PredictionSet[1:13]' (iter 6/10)
## INFO  [08:02:52.918] [mlr3] Applying learner 'regr.kknn' on task 'PredictionSet[1:13]' (iter 7/10)
## INFO  [08:02:52.938] [mlr3] Applying learner 'regr.kknn' on task 'PredictionSet[1:13]' (iter 8/10)
## INFO  [08:02:52.958] [mlr3] Applying learner 'regr.kknn' on task 'PredictionSet[1:13]' (iter 9/10)
## INFO  [08:02:52.980] [mlr3] Applying learner 'regr.kknn' on task 'PredictionSet[1:13]' (iter 10/10)
## INFO  [08:02:53.001] [mlr3] Finished benchmark
## INFO  [08:02:53.033] [bbotk] Result of batch 9:
## INFO  [08:02:53.034] [bbotk]   k distance      kernel regr.rmse warnings errors runtime_learners
## INFO  [08:02:53.034] [bbotk]  18       20 rectangular  8.789107        0      0            0.105
## INFO  [08:02:53.034] [bbotk]                                 uhash
## INFO  [08:02:53.034] [bbotk]  7af383e0-58df-4769-b2fa-3e00e545c082
## INFO  [08:02:53.036] [bbotk] Evaluating 1 configuration(s)
## INFO  [08:02:53.046] [mlr3] Running benchmark with 10 resampling iterations
## INFO  [08:02:53.052] [mlr3] Applying learner 'regr.kknn' on task 'PredictionSet[1:13]' (iter 1/10)
## INFO  [08:02:53.074] [mlr3] Applying learner 'regr.kknn' on task 'PredictionSet[1:13]' (iter 2/10)
## INFO  [08:02:53.094] [mlr3] Applying learner 'regr.kknn' on task 'PredictionSet[1:13]' (iter 3/10)
## INFO  [08:02:53.115] [mlr3] Applying learner 'regr.kknn' on task 'PredictionSet[1:13]' (iter 4/10)
## INFO  [08:02:53.146] [mlr3] Applying learner 'regr.kknn' on task 'PredictionSet[1:13]' (iter 5/10)
## INFO  [08:02:53.166] [mlr3] Applying learner 'regr.kknn' on task 'PredictionSet[1:13]' (iter 6/10)
## INFO  [08:02:53.186] [mlr3] Applying learner 'regr.kknn' on task 'PredictionSet[1:13]' (iter 7/10)
## INFO  [08:02:53.207] [mlr3] Applying learner 'regr.kknn' on task 'PredictionSet[1:13]' (iter 8/10)
## INFO  [08:02:53.229] [mlr3] Applying learner 'regr.kknn' on task 'PredictionSet[1:13]' (iter 9/10)
## INFO  [08:02:53.249] [mlr3] Applying learner 'regr.kknn' on task 'PredictionSet[1:13]' (iter 10/10)
## INFO  [08:02:53.268] [mlr3] Finished benchmark
## INFO  [08:02:53.302] [bbotk] Result of batch 10:
## INFO  [08:02:53.303] [bbotk]   k distance      kernel regr.rmse warnings errors runtime_learners
## INFO  [08:02:53.303] [bbotk]  10 2.942857 rectangular  8.049561        0      0            0.107
## INFO  [08:02:53.303] [bbotk]                                 uhash
## INFO  [08:02:53.303] [bbotk]  4deeafa6-3328-4c92-baf2-5d7927a3e565
## INFO  [08:02:53.308] [bbotk] Finished optimizing after 10 evaluation(s)
## INFO  [08:02:53.309] [bbotk] Result:
## INFO  [08:02:53.310] [bbotk]   k distance      kernel learner_param_vals  x_domain regr.rmse
## INFO  [08:02:53.310] [bbotk]  10 2.942857 rectangular          <list[3]> <list[3]>  8.049561
## INFO  [08:02:53.566] [bbotk] Starting to optimize 2 parameter(s) with '<TunerGridSearch>' and '<TerminatorEvals> [n_evals=10, k=0]'
## INFO  [08:02:53.569] [bbotk] Evaluating 1 configuration(s)
## INFO  [08:02:53.593] [mlr3] Running benchmark with 10 resampling iterations
## INFO  [08:02:53.598] [mlr3] Applying learner 'regr.ranger' on task 'PredictionSet[1:13]' (iter 1/10)
## INFO  [08:02:53.633] [mlr3] Applying learner 'regr.ranger' on task 'PredictionSet[1:13]' (iter 2/10)
## INFO  [08:02:53.667] [mlr3] Applying learner 'regr.ranger' on task 'PredictionSet[1:13]' (iter 3/10)
## INFO  [08:02:53.700] [mlr3] Applying learner 'regr.ranger' on task 'PredictionSet[1:13]' (iter 4/10)
## INFO  [08:02:53.736] [mlr3] Applying learner 'regr.ranger' on task 'PredictionSet[1:13]' (iter 5/10)
## INFO  [08:02:53.769] [mlr3] Applying learner 'regr.ranger' on task 'PredictionSet[1:13]' (iter 6/10)
## INFO  [08:02:53.804] [mlr3] Applying learner 'regr.ranger' on task 'PredictionSet[1:13]' (iter 7/10)
## INFO  [08:02:53.837] [mlr3] Applying learner 'regr.ranger' on task 'PredictionSet[1:13]' (iter 8/10)
## INFO  [08:02:53.869] [mlr3] Applying learner 'regr.ranger' on task 'PredictionSet[1:13]' (iter 9/10)
## INFO  [08:02:53.903] [mlr3] Applying learner 'regr.ranger' on task 'PredictionSet[1:13]' (iter 10/10)
## INFO  [08:02:53.935] [mlr3] Finished benchmark
## INFO  [08:02:53.981] [bbotk] Result of batch 1:
## INFO  [08:02:53.982] [bbotk]  max.depth num.trees regr.rmse warnings errors runtime_learners
## INFO  [08:02:53.982] [bbotk]         14       264  6.588243        0      0            0.232
## INFO  [08:02:53.982] [bbotk]                                 uhash
## INFO  [08:02:53.982] [bbotk]  eb3ba766-9420-480c-8a8e-6b2f894b3a70
## INFO  [08:02:53.984] [bbotk] Evaluating 1 configuration(s)
## INFO  [08:02:54.008] [mlr3] Running benchmark with 10 resampling iterations
## INFO  [08:02:54.013] [mlr3] Applying learner 'regr.ranger' on task 'PredictionSet[1:13]' (iter 1/10)
## INFO  [08:02:54.055] [mlr3] Applying learner 'regr.ranger' on task 'PredictionSet[1:13]' (iter 2/10)
## INFO  [08:02:54.097] [mlr3] Applying learner 'regr.ranger' on task 'PredictionSet[1:13]' (iter 3/10)
## INFO  [08:02:54.139] [mlr3] Applying learner 'regr.ranger' on task 'PredictionSet[1:13]' (iter 4/10)
## INFO  [08:02:54.181] [mlr3] Applying learner 'regr.ranger' on task 'PredictionSet[1:13]' (iter 5/10)
## INFO  [08:02:54.223] [mlr3] Applying learner 'regr.ranger' on task 'PredictionSet[1:13]' (iter 6/10)
## INFO  [08:02:54.267] [mlr3] Applying learner 'regr.ranger' on task 'PredictionSet[1:13]' (iter 7/10)
## INFO  [08:02:54.307] [mlr3] Applying learner 'regr.ranger' on task 'PredictionSet[1:13]' (iter 8/10)
## INFO  [08:02:54.350] [mlr3] Applying learner 'regr.ranger' on task 'PredictionSet[1:13]' (iter 9/10)
## INFO  [08:02:54.391] [mlr3] Applying learner 'regr.ranger' on task 'PredictionSet[1:13]' (iter 10/10)
## INFO  [08:02:54.432] [mlr3] Finished benchmark
## INFO  [08:02:54.464] [bbotk] Result of batch 2:
## INFO  [08:02:54.466] [bbotk]  max.depth num.trees regr.rmse warnings errors runtime_learners
## INFO  [08:02:54.466] [bbotk]         13       395  6.674037        0      0            0.314
## INFO  [08:02:54.466] [bbotk]                                 uhash
## INFO  [08:02:54.466] [bbotk]  f48c4a7d-20e7-4bdb-b293-d586c97e1ca5
## INFO  [08:02:54.467] [bbotk] Evaluating 1 configuration(s)
## INFO  [08:02:54.490] [mlr3] Running benchmark with 10 resampling iterations
## INFO  [08:02:54.495] [mlr3] Applying learner 'regr.ranger' on task 'PredictionSet[1:13]' (iter 1/10)
## INFO  [08:02:54.533] [mlr3] Applying learner 'regr.ranger' on task 'PredictionSet[1:13]' (iter 2/10)
## INFO  [08:02:54.572] [mlr3] Applying learner 'regr.ranger' on task 'PredictionSet[1:13]' (iter 3/10)
## INFO  [08:02:54.622] [mlr3] Applying learner 'regr.ranger' on task 'PredictionSet[1:13]' (iter 4/10)
## INFO  [08:02:54.665] [mlr3] Applying learner 'regr.ranger' on task 'PredictionSet[1:13]' (iter 5/10)
## INFO  [08:02:54.707] [mlr3] Applying learner 'regr.ranger' on task 'PredictionSet[1:13]' (iter 6/10)
## INFO  [08:02:54.747] [mlr3] Applying learner 'regr.ranger' on task 'PredictionSet[1:13]' (iter 7/10)
## INFO  [08:02:54.787] [mlr3] Applying learner 'regr.ranger' on task 'PredictionSet[1:13]' (iter 8/10)
## INFO  [08:02:54.826] [mlr3] Applying learner 'regr.ranger' on task 'PredictionSet[1:13]' (iter 9/10)
## INFO  [08:02:54.866] [mlr3] Applying learner 'regr.ranger' on task 'PredictionSet[1:13]' (iter 10/10)
## INFO  [08:02:54.904] [mlr3] Finished benchmark
## INFO  [08:02:54.936] [bbotk] Result of batch 3:
## INFO  [08:02:54.938] [bbotk]  max.depth num.trees regr.rmse warnings errors runtime_learners
## INFO  [08:02:54.938] [bbotk]          5       448  7.130018        0      0            0.296
## INFO  [08:02:54.938] [bbotk]                                 uhash
## INFO  [08:02:54.938] [bbotk]  f24d4c79-9473-4f8d-a01f-728266410796
## INFO  [08:02:54.940] [bbotk] Evaluating 1 configuration(s)
## INFO  [08:02:54.961] [mlr3] Running benchmark with 10 resampling iterations
## INFO  [08:02:54.966] [mlr3] Applying learner 'regr.ranger' on task 'PredictionSet[1:13]' (iter 1/10)
## INFO  [08:02:55.010] [mlr3] Applying learner 'regr.ranger' on task 'PredictionSet[1:13]' (iter 2/10)
## INFO  [08:02:55.053] [mlr3] Applying learner 'regr.ranger' on task 'PredictionSet[1:13]' (iter 3/10)
## INFO  [08:02:55.098] [mlr3] Applying learner 'regr.ranger' on task 'PredictionSet[1:13]' (iter 4/10)
## INFO  [08:02:55.141] [mlr3] Applying learner 'regr.ranger' on task 'PredictionSet[1:13]' (iter 5/10)
## INFO  [08:02:55.185] [mlr3] Applying learner 'regr.ranger' on task 'PredictionSet[1:13]' (iter 6/10)
## INFO  [08:02:55.228] [mlr3] Applying learner 'regr.ranger' on task 'PredictionSet[1:13]' (iter 7/10)
## INFO  [08:02:55.272] [mlr3] Applying learner 'regr.ranger' on task 'PredictionSet[1:13]' (iter 8/10)
## INFO  [08:02:55.317] [mlr3] Applying learner 'regr.ranger' on task 'PredictionSet[1:13]' (iter 9/10)
## INFO  [08:02:55.360] [mlr3] Applying learner 'regr.ranger' on task 'PredictionSet[1:13]' (iter 10/10)
## INFO  [08:02:55.417] [mlr3] Finished benchmark
## INFO  [08:02:55.453] [bbotk] Result of batch 4:
## INFO  [08:02:55.456] [bbotk]  max.depth num.trees regr.rmse warnings errors runtime_learners
## INFO  [08:02:55.456] [bbotk]         18       422  6.652684        0      0             0.33
## INFO  [08:02:55.456] [bbotk]                                 uhash
## INFO  [08:02:55.456] [bbotk]  075cab77-e753-4514-a86c-a4e1db016130
## INFO  [08:02:55.458] [bbotk] Evaluating 1 configuration(s)
## INFO  [08:02:55.481] [mlr3] Running benchmark with 10 resampling iterations
## INFO  [08:02:55.486] [mlr3] Applying learner 'regr.ranger' on task 'PredictionSet[1:13]' (iter 1/10)
## INFO  [08:02:55.532] [mlr3] Applying learner 'regr.ranger' on task 'PredictionSet[1:13]' (iter 2/10)
## INFO  [08:02:55.577] [mlr3] Applying learner 'regr.ranger' on task 'PredictionSet[1:13]' (iter 3/10)
## INFO  [08:02:55.623] [mlr3] Applying learner 'regr.ranger' on task 'PredictionSet[1:13]' (iter 4/10)
## INFO  [08:02:55.668] [mlr3] Applying learner 'regr.ranger' on task 'PredictionSet[1:13]' (iter 5/10)
## INFO  [08:02:55.713] [mlr3] Applying learner 'regr.ranger' on task 'PredictionSet[1:13]' (iter 6/10)
## INFO  [08:02:55.759] [mlr3] Applying learner 'regr.ranger' on task 'PredictionSet[1:13]' (iter 7/10)
## INFO  [08:02:55.804] [mlr3] Applying learner 'regr.ranger' on task 'PredictionSet[1:13]' (iter 8/10)
## INFO  [08:02:55.849] [mlr3] Applying learner 'regr.ranger' on task 'PredictionSet[1:13]' (iter 9/10)
## INFO  [08:02:55.894] [mlr3] Applying learner 'regr.ranger' on task 'PredictionSet[1:13]' (iter 10/10)
## INFO  [08:02:55.940] [mlr3] Finished benchmark
## INFO  [08:02:55.971] [bbotk] Result of batch 5:
## INFO  [08:02:55.973] [bbotk]  max.depth num.trees regr.rmse warnings errors runtime_learners
## INFO  [08:02:55.973] [bbotk]         16       448  6.624815        0      0            0.349
## INFO  [08:02:55.973] [bbotk]                                 uhash
## INFO  [08:02:55.973] [bbotk]  95f3a3de-0bd1-4e77-8d7b-d43ff0ddf3b5
## INFO  [08:02:55.975] [bbotk] Evaluating 1 configuration(s)
## INFO  [08:02:55.997] [mlr3] Running benchmark with 10 resampling iterations
## INFO  [08:02:56.003] [mlr3] Applying learner 'regr.ranger' on task 'PredictionSet[1:13]' (iter 1/10)
## INFO  [08:02:56.040] [mlr3] Applying learner 'regr.ranger' on task 'PredictionSet[1:13]' (iter 2/10)
## INFO  [08:02:56.071] [mlr3] Applying learner 'regr.ranger' on task 'PredictionSet[1:13]' (iter 3/10)
## INFO  [08:02:56.101] [mlr3] Applying learner 'regr.ranger' on task 'PredictionSet[1:13]' (iter 4/10)
## INFO  [08:02:56.130] [mlr3] Applying learner 'regr.ranger' on task 'PredictionSet[1:13]' (iter 5/10)
## INFO  [08:02:56.158] [mlr3] Applying learner 'regr.ranger' on task 'PredictionSet[1:13]' (iter 6/10)
## INFO  [08:02:56.185] [mlr3] Applying learner 'regr.ranger' on task 'PredictionSet[1:13]' (iter 7/10)
## INFO  [08:02:56.213] [mlr3] Applying learner 'regr.ranger' on task 'PredictionSet[1:13]' (iter 8/10)
## INFO  [08:02:56.240] [mlr3] Applying learner 'regr.ranger' on task 'PredictionSet[1:13]' (iter 9/10)
## INFO  [08:02:56.267] [mlr3] Applying learner 'regr.ranger' on task 'PredictionSet[1:13]' (iter 10/10)
## INFO  [08:02:56.295] [mlr3] Finished benchmark
## INFO  [08:02:56.326] [bbotk] Result of batch 6:
## INFO  [08:02:56.328] [bbotk]  max.depth num.trees regr.rmse warnings errors runtime_learners
## INFO  [08:02:56.328] [bbotk]          7       185  6.777395        0      0             0.18
## INFO  [08:02:56.328] [bbotk]                                 uhash
## INFO  [08:02:56.328] [bbotk]  7eb3b315-c156-4b03-a8b0-2688c3b20488
## INFO  [08:02:56.329] [bbotk] Evaluating 1 configuration(s)
## INFO  [08:02:56.351] [mlr3] Running benchmark with 10 resampling iterations
## INFO  [08:02:56.356] [mlr3] Applying learner 'regr.ranger' on task 'PredictionSet[1:13]' (iter 1/10)
## INFO  [08:02:56.397] [mlr3] Applying learner 'regr.ranger' on task 'PredictionSet[1:13]' (iter 2/10)
## INFO  [08:02:56.439] [mlr3] Applying learner 'regr.ranger' on task 'PredictionSet[1:13]' (iter 3/10)
## INFO  [08:02:56.478] [mlr3] Applying learner 'regr.ranger' on task 'PredictionSet[1:13]' (iter 4/10)
## INFO  [08:02:56.520] [mlr3] Applying learner 'regr.ranger' on task 'PredictionSet[1:13]' (iter 5/10)
## INFO  [08:02:56.559] [mlr3] Applying learner 'regr.ranger' on task 'PredictionSet[1:13]' (iter 6/10)
## INFO  [08:02:56.600] [mlr3] Applying learner 'regr.ranger' on task 'PredictionSet[1:13]' (iter 7/10)
## INFO  [08:02:56.640] [mlr3] Applying learner 'regr.ranger' on task 'PredictionSet[1:13]' (iter 8/10)
## INFO  [08:02:56.693] [mlr3] Applying learner 'regr.ranger' on task 'PredictionSet[1:13]' (iter 9/10)
## INFO  [08:02:56.737] [mlr3] Applying learner 'regr.ranger' on task 'PredictionSet[1:13]' (iter 10/10)
## INFO  [08:02:56.778] [mlr3] Finished benchmark
## INFO  [08:02:56.811] [bbotk] Result of batch 7:
## INFO  [08:02:56.813] [bbotk]  max.depth num.trees regr.rmse warnings errors runtime_learners
## INFO  [08:02:56.813] [bbotk]          9       369  6.604303        0      0            0.303
## INFO  [08:02:56.813] [bbotk]                                 uhash
## INFO  [08:02:56.813] [bbotk]  13874bf7-8f9b-476b-b1da-443c7adb4954
## INFO  [08:02:56.815] [bbotk] Evaluating 1 configuration(s)
## INFO  [08:02:56.836] [mlr3] Running benchmark with 10 resampling iterations
## INFO  [08:02:56.841] [mlr3] Applying learner 'regr.ranger' on task 'PredictionSet[1:13]' (iter 1/10)
## INFO  [08:02:56.861] [mlr3] Applying learner 'regr.ranger' on task 'PredictionSet[1:13]' (iter 2/10)
## INFO  [08:02:56.882] [mlr3] Applying learner 'regr.ranger' on task 'PredictionSet[1:13]' (iter 3/10)
## INFO  [08:02:56.903] [mlr3] Applying learner 'regr.ranger' on task 'PredictionSet[1:13]' (iter 4/10)
## INFO  [08:02:56.924] [mlr3] Applying learner 'regr.ranger' on task 'PredictionSet[1:13]' (iter 5/10)
## INFO  [08:02:56.944] [mlr3] Applying learner 'regr.ranger' on task 'PredictionSet[1:13]' (iter 6/10)
## INFO  [08:02:56.966] [mlr3] Applying learner 'regr.ranger' on task 'PredictionSet[1:13]' (iter 7/10)
## INFO  [08:02:56.986] [mlr3] Applying learner 'regr.ranger' on task 'PredictionSet[1:13]' (iter 8/10)
## INFO  [08:02:57.006] [mlr3] Applying learner 'regr.ranger' on task 'PredictionSet[1:13]' (iter 9/10)
## INFO  [08:02:57.027] [mlr3] Applying learner 'regr.ranger' on task 'PredictionSet[1:13]' (iter 10/10)
## INFO  [08:02:57.048] [mlr3] Finished benchmark
## INFO  [08:02:57.079] [bbotk] Result of batch 8:
## INFO  [08:02:57.081] [bbotk]  max.depth num.trees regr.rmse warnings errors runtime_learners
## INFO  [08:02:57.081] [bbotk]         16        79  6.590366        0      0            0.104
## INFO  [08:02:57.081] [bbotk]                                 uhash
## INFO  [08:02:57.081] [bbotk]  7fa08e08-942f-4877-a5f7-6c937ee1c784
## INFO  [08:02:57.082] [bbotk] Evaluating 1 configuration(s)
## INFO  [08:02:57.117] [mlr3] Running benchmark with 10 resampling iterations
## INFO  [08:02:57.123] [mlr3] Applying learner 'regr.ranger' on task 'PredictionSet[1:13]' (iter 1/10)
## INFO  [08:02:57.142] [mlr3] Applying learner 'regr.ranger' on task 'PredictionSet[1:13]' (iter 2/10)
## INFO  [08:02:57.160] [mlr3] Applying learner 'regr.ranger' on task 'PredictionSet[1:13]' (iter 3/10)
## INFO  [08:02:57.177] [mlr3] Applying learner 'regr.ranger' on task 'PredictionSet[1:13]' (iter 4/10)
## INFO  [08:02:57.196] [mlr3] Applying learner 'regr.ranger' on task 'PredictionSet[1:13]' (iter 5/10)
## INFO  [08:02:57.212] [mlr3] Applying learner 'regr.ranger' on task 'PredictionSet[1:13]' (iter 6/10)
## INFO  [08:02:57.229] [mlr3] Applying learner 'regr.ranger' on task 'PredictionSet[1:13]' (iter 7/10)
## INFO  [08:02:57.245] [mlr3] Applying learner 'regr.ranger' on task 'PredictionSet[1:13]' (iter 8/10)
## INFO  [08:02:57.264] [mlr3] Applying learner 'regr.ranger' on task 'PredictionSet[1:13]' (iter 9/10)
## INFO  [08:02:57.280] [mlr3] Applying learner 'regr.ranger' on task 'PredictionSet[1:13]' (iter 10/10)
## INFO  [08:02:57.297] [mlr3] Finished benchmark
## INFO  [08:02:57.328] [bbotk] Result of batch 9:
## INFO  [08:02:57.330] [bbotk]  max.depth num.trees regr.rmse warnings errors runtime_learners
## INFO  [08:02:57.330] [bbotk]         14        27  6.441427        0      0            0.069
## INFO  [08:02:57.330] [bbotk]                                 uhash
## INFO  [08:02:57.330] [bbotk]  014d3daf-1347-457b-bf55-8ecc09ebed9a
## INFO  [08:02:57.332] [bbotk] Evaluating 1 configuration(s)
## INFO  [08:02:57.354] [mlr3] Running benchmark with 10 resampling iterations
## INFO  [08:02:57.360] [mlr3] Applying learner 'regr.ranger' on task 'PredictionSet[1:13]' (iter 1/10)
## INFO  [08:02:57.385] [mlr3] Applying learner 'regr.ranger' on task 'PredictionSet[1:13]' (iter 2/10)
## INFO  [08:02:57.413] [mlr3] Applying learner 'regr.ranger' on task 'PredictionSet[1:13]' (iter 3/10)
## INFO  [08:02:57.438] [mlr3] Applying learner 'regr.ranger' on task 'PredictionSet[1:13]' (iter 4/10)
## INFO  [08:02:57.463] [mlr3] Applying learner 'regr.ranger' on task 'PredictionSet[1:13]' (iter 5/10)
## INFO  [08:02:57.500] [mlr3] Applying learner 'regr.ranger' on task 'PredictionSet[1:13]' (iter 6/10)
## INFO  [08:02:57.529] [mlr3] Applying learner 'regr.ranger' on task 'PredictionSet[1:13]' (iter 7/10)
## INFO  [08:02:57.560] [mlr3] Applying learner 'regr.ranger' on task 'PredictionSet[1:13]' (iter 8/10)
## INFO  [08:02:57.587] [mlr3] Applying learner 'regr.ranger' on task 'PredictionSet[1:13]' (iter 9/10)
## INFO  [08:02:57.613] [mlr3] Applying learner 'regr.ranger' on task 'PredictionSet[1:13]' (iter 10/10)
## INFO  [08:02:57.640] [mlr3] Finished benchmark
## INFO  [08:02:57.672] [bbotk] Result of batch 10:
## INFO  [08:02:57.673] [bbotk]  max.depth num.trees regr.rmse warnings errors runtime_learners
## INFO  [08:02:57.673] [bbotk]         18       158  6.601412        0      0            0.169
## INFO  [08:02:57.673] [bbotk]                                 uhash
## INFO  [08:02:57.673] [bbotk]  08024379-2d3c-47df-94bf-caea68b7933f
## INFO  [08:02:57.678] [bbotk] Finished optimizing after 10 evaluation(s)
## INFO  [08:02:57.679] [bbotk] Result:
## INFO  [08:02:57.680] [bbotk]  max.depth num.trees learner_param_vals  x_domain regr.rmse
## INFO  [08:02:57.680] [bbotk]         14        27          <list[3]> <list[2]>  6.441427
## INFO  [08:02:57.931] [bbotk] Starting to optimize 5 parameter(s) with '<TunerGridSearch>' and '<TerminatorEvals> [n_evals=10, k=0]'
## INFO  [08:02:57.939] [bbotk] Evaluating 1 configuration(s)
## INFO  [08:02:57.955] [mlr3] Running benchmark with 10 resampling iterations
## INFO  [08:02:57.961] [mlr3] Applying learner 'regr.svm' on task 'PredictionSet[1:13]' (iter 1/10)
## INFO  [08:02:57.979] [mlr3] Applying learner 'regr.svm' on task 'PredictionSet[1:13]' (iter 2/10)
## INFO  [08:02:57.996] [mlr3] Applying learner 'regr.svm' on task 'PredictionSet[1:13]' (iter 3/10)
## INFO  [08:02:58.016] [mlr3] Applying learner 'regr.svm' on task 'PredictionSet[1:13]' (iter 4/10)
## INFO  [08:02:58.034] [mlr3] Applying learner 'regr.svm' on task 'PredictionSet[1:13]' (iter 5/10)
## INFO  [08:02:58.052] [mlr3] Applying learner 'regr.svm' on task 'PredictionSet[1:13]' (iter 6/10)
## INFO  [08:02:58.070] [mlr3] Applying learner 'regr.svm' on task 'PredictionSet[1:13]' (iter 7/10)
## INFO  [08:02:58.090] [mlr3] Applying learner 'regr.svm' on task 'PredictionSet[1:13]' (iter 8/10)
## INFO  [08:02:58.109] [mlr3] Applying learner 'regr.svm' on task 'PredictionSet[1:13]' (iter 9/10)
## INFO  [08:02:58.128] [mlr3] Applying learner 'regr.svm' on task 'PredictionSet[1:13]' (iter 10/10)
## INFO  [08:02:58.145] [mlr3] Finished benchmark
## INFO  [08:02:58.177] [bbotk] Result of batch 1:
## INFO  [08:02:58.178] [bbotk]   tolerance    gamma   epsilon           type  kernel regr.rmse warnings errors
## INFO  [08:02:58.178] [bbotk]  0.06521429 42.85729 0.6142857 eps-regression sigmoid  2.994538        0      0
## INFO  [08:02:58.178] [bbotk]  runtime_learners                                uhash
## INFO  [08:02:58.178] [bbotk]             0.085 7d8f9cf9-62da-4e53-a401-e7ed9c5e72f9
## INFO  [08:02:58.180] [bbotk] Evaluating 1 configuration(s)
## INFO  [08:02:58.203] [mlr3] Running benchmark with 10 resampling iterations
## INFO  [08:02:58.210] [mlr3] Applying learner 'regr.svm' on task 'PredictionSet[1:13]' (iter 1/10)
## INFO  [08:02:58.232] [mlr3] Applying learner 'regr.svm' on task 'PredictionSet[1:13]' (iter 2/10)
## INFO  [08:02:58.255] [mlr3] Applying learner 'regr.svm' on task 'PredictionSet[1:13]' (iter 3/10)
## INFO  [08:02:58.274] [mlr3] Applying learner 'regr.svm' on task 'PredictionSet[1:13]' (iter 4/10)
## INFO  [08:02:58.292] [mlr3] Applying learner 'regr.svm' on task 'PredictionSet[1:13]' (iter 5/10)
## INFO  [08:02:58.313] [mlr3] Applying learner 'regr.svm' on task 'PredictionSet[1:13]' (iter 6/10)
## INFO  [08:02:58.332] [mlr3] Applying learner 'regr.svm' on task 'PredictionSet[1:13]' (iter 7/10)
## INFO  [08:02:58.350] [mlr3] Applying learner 'regr.svm' on task 'PredictionSet[1:13]' (iter 8/10)
## INFO  [08:02:58.368] [mlr3] Applying learner 'regr.svm' on task 'PredictionSet[1:13]' (iter 9/10)
## INFO  [08:02:58.388] [mlr3] Applying learner 'regr.svm' on task 'PredictionSet[1:13]' (iter 10/10)
## INFO  [08:02:58.407] [mlr3] Finished benchmark
## INFO  [08:02:58.437] [bbotk] Result of batch 2:
## INFO  [08:02:58.439] [bbotk]  tolerance    gamma epsilon           type  kernel regr.rmse warnings errors
## INFO  [08:02:58.439] [bbotk]  0.3220714 17.85779     0.5 eps-regression sigmoid  3.001697        0      0
## INFO  [08:02:58.439] [bbotk]  runtime_learners                                uhash
## INFO  [08:02:58.439] [bbotk]             0.091 da547a1d-15f9-440b-a36a-76ced389c48b
## INFO  [08:02:58.441] [bbotk] Evaluating 1 configuration(s)
## INFO  [08:02:58.457] [mlr3] Running benchmark with 10 resampling iterations
## INFO  [08:02:58.463] [mlr3] Applying learner 'regr.svm' on task 'PredictionSet[1:13]' (iter 1/10)
## INFO  [08:02:58.483] [mlr3] Applying learner 'regr.svm' on task 'PredictionSet[1:13]' (iter 2/10)
## INFO  [08:02:58.502] [mlr3] Applying learner 'regr.svm' on task 'PredictionSet[1:13]' (iter 3/10)
## INFO  [08:02:58.521] [mlr3] Applying learner 'regr.svm' on task 'PredictionSet[1:13]' (iter 4/10)
## INFO  [08:02:58.541] [mlr3] Applying learner 'regr.svm' on task 'PredictionSet[1:13]' (iter 5/10)
## INFO  [08:02:58.561] [mlr3] Applying learner 'regr.svm' on task 'PredictionSet[1:13]' (iter 6/10)
## INFO  [08:02:58.588] [mlr3] Applying learner 'regr.svm' on task 'PredictionSet[1:13]' (iter 7/10)
## INFO  [08:02:58.613] [mlr3] Applying learner 'regr.svm' on task 'PredictionSet[1:13]' (iter 8/10)
## INFO  [08:02:58.635] [mlr3] Applying learner 'regr.svm' on task 'PredictionSet[1:13]' (iter 9/10)
## INFO  [08:02:58.655] [mlr3] Applying learner 'regr.svm' on task 'PredictionSet[1:13]' (iter 10/10)
## INFO  [08:02:58.674] [mlr3] Finished benchmark
## INFO  [08:02:58.707] [bbotk] Result of batch 3:
## INFO  [08:02:58.709] [bbotk]  tolerance    gamma   epsilon           type  kernel regr.rmse warnings errors
## INFO  [08:02:58.709] [bbotk]        0.9 7.143714 0.7857143 eps-regression sigmoid  2.961615        0      0
## INFO  [08:02:58.709] [bbotk]  runtime_learners                                uhash
## INFO  [08:02:58.709] [bbotk]             0.104 ee0b1ed8-de98-444c-84bd-20c563ec478a
## INFO  [08:02:58.710] [bbotk] Evaluating 1 configuration(s)
## INFO  [08:02:58.726] [mlr3] Running benchmark with 10 resampling iterations
## INFO  [08:02:58.731] [mlr3] Applying learner 'regr.svm' on task 'PredictionSet[1:13]' (iter 1/10)
## INFO  [08:02:58.749] [mlr3] Applying learner 'regr.svm' on task 'PredictionSet[1:13]' (iter 2/10)
## INFO  [08:02:58.768] [mlr3] Applying learner 'regr.svm' on task 'PredictionSet[1:13]' (iter 3/10)
## INFO  [08:02:58.787] [mlr3] Applying learner 'regr.svm' on task 'PredictionSet[1:13]' (iter 4/10)
## INFO  [08:02:58.805] [mlr3] Applying learner 'regr.svm' on task 'PredictionSet[1:13]' (iter 5/10)
## INFO  [08:02:58.823] [mlr3] Applying learner 'regr.svm' on task 'PredictionSet[1:13]' (iter 6/10)
## INFO  [08:02:58.843] [mlr3] Applying learner 'regr.svm' on task 'PredictionSet[1:13]' (iter 7/10)
## INFO  [08:02:58.861] [mlr3] Applying learner 'regr.svm' on task 'PredictionSet[1:13]' (iter 8/10)
## INFO  [08:02:58.879] [mlr3] Applying learner 'regr.svm' on task 'PredictionSet[1:13]' (iter 9/10)
## INFO  [08:02:58.897] [mlr3] Applying learner 'regr.svm' on task 'PredictionSet[1:13]' (iter 10/10)
## INFO  [08:02:58.917] [mlr3] Finished benchmark
## INFO  [08:02:58.957] [bbotk] Result of batch 4:
## INFO  [08:02:58.959] [bbotk]  tolerance    gamma epsilon           type kernel  regr.rmse warnings errors
## INFO  [08:02:58.959] [bbotk]  0.1294286 28.57186     0.1 eps-regression radial 0.07060421        0      0
## INFO  [08:02:58.959] [bbotk]  runtime_learners                                uhash
## INFO  [08:02:58.959] [bbotk]             0.087 938def64-6e9d-43b6-9de1-90c0194e7d9f
## INFO  [08:02:58.961] [bbotk] Evaluating 1 configuration(s)
## INFO  [08:02:58.981] [mlr3] Running benchmark with 10 resampling iterations
## INFO  [08:02:58.988] [mlr3] Applying learner 'regr.svm' on task 'PredictionSet[1:13]' (iter 1/10)
## INFO  [08:02:59.006] [mlr3] Applying learner 'regr.svm' on task 'PredictionSet[1:13]' (iter 2/10)
## INFO  [08:02:59.023] [mlr3] Applying learner 'regr.svm' on task 'PredictionSet[1:13]' (iter 3/10)
## INFO  [08:02:59.039] [mlr3] Applying learner 'regr.svm' on task 'PredictionSet[1:13]' (iter 4/10)
## INFO  [08:02:59.057] [mlr3] Applying learner 'regr.svm' on task 'PredictionSet[1:13]' (iter 5/10)
## INFO  [08:02:59.075] [mlr3] Applying learner 'regr.svm' on task 'PredictionSet[1:13]' (iter 6/10)
## INFO  [08:02:59.091] [mlr3] Applying learner 'regr.svm' on task 'PredictionSet[1:13]' (iter 7/10)
## INFO  [08:02:59.108] [mlr3] Applying learner 'regr.svm' on task 'PredictionSet[1:13]' (iter 8/10)
## INFO  [08:02:59.124] [mlr3] Applying learner 'regr.svm' on task 'PredictionSet[1:13]' (iter 9/10)
## INFO  [08:02:59.142] [mlr3] Applying learner 'regr.svm' on task 'PredictionSet[1:13]' (iter 10/10)
## INFO  [08:02:59.159] [mlr3] Finished benchmark
## INFO  [08:02:59.190] [bbotk] Result of batch 5:
## INFO  [08:02:59.192] [bbotk]  tolerance gamma   epsilon           type  kernel  regr.rmse warnings errors
## INFO  [08:02:59.192] [bbotk]     0.4505 0.001 0.3285714 eps-regression sigmoid 0.08645955        0      0
## INFO  [08:02:59.192] [bbotk]  runtime_learners                                uhash
## INFO  [08:02:59.192] [bbotk]             0.072 ae656d6d-0ae4-4220-a6a9-bfcbb81e9e3f
## INFO  [08:02:59.193] [bbotk] Evaluating 1 configuration(s)
## INFO  [08:02:59.210] [mlr3] Running benchmark with 10 resampling iterations
## INFO  [08:02:59.216] [mlr3] Applying learner 'regr.svm' on task 'PredictionSet[1:13]' (iter 1/10)
## INFO  [08:02:59.234] [mlr3] Applying learner 'regr.svm' on task 'PredictionSet[1:13]' (iter 2/10)
## INFO  [08:02:59.252] [mlr3] Applying learner 'regr.svm' on task 'PredictionSet[1:13]' (iter 3/10)
## INFO  [08:02:59.269] [mlr3] Applying learner 'regr.svm' on task 'PredictionSet[1:13]' (iter 4/10)
## INFO  [08:02:59.298] [mlr3] Applying learner 'regr.svm' on task 'PredictionSet[1:13]' (iter 5/10)
## INFO  [08:02:59.319] [mlr3] Applying learner 'regr.svm' on task 'PredictionSet[1:13]' (iter 6/10)
## INFO  [08:02:59.340] [mlr3] Applying learner 'regr.svm' on task 'PredictionSet[1:13]' (iter 7/10)
## INFO  [08:02:59.360] [mlr3] Applying learner 'regr.svm' on task 'PredictionSet[1:13]' (iter 8/10)
## INFO  [08:02:59.380] [mlr3] Applying learner 'regr.svm' on task 'PredictionSet[1:13]' (iter 9/10)
## INFO  [08:02:59.398] [mlr3] Applying learner 'regr.svm' on task 'PredictionSet[1:13]' (iter 10/10)
## INFO  [08:02:59.416] [mlr3] Finished benchmark
## INFO  [08:02:59.450] [bbotk] Result of batch 6:
## INFO  [08:02:59.451] [bbotk]  tolerance    gamma   epsilon           type  kernel regr.rmse warnings errors
## INFO  [08:02:59.451] [bbotk]        0.9 42.85729 0.7285714 eps-regression sigmoid  2.987692        0      0
## INFO  [08:02:59.451] [bbotk]  runtime_learners                                uhash
## INFO  [08:02:59.451] [bbotk]             0.085 741491eb-fad3-494c-8536-35a910d31cb3
## INFO  [08:02:59.453] [bbotk] Evaluating 1 configuration(s)
## INFO  [08:02:59.469] [mlr3] Running benchmark with 10 resampling iterations
## INFO  [08:02:59.474] [mlr3] Applying learner 'regr.svm' on task 'PredictionSet[1:13]' (iter 1/10)
## INFO  [08:02:59.492] [mlr3] Applying learner 'regr.svm' on task 'PredictionSet[1:13]' (iter 2/10)
## INFO  [08:02:59.512] [mlr3] Applying learner 'regr.svm' on task 'PredictionSet[1:13]' (iter 3/10)
## INFO  [08:02:59.531] [mlr3] Applying learner 'regr.svm' on task 'PredictionSet[1:13]' (iter 4/10)
## INFO  [08:02:59.549] [mlr3] Applying learner 'regr.svm' on task 'PredictionSet[1:13]' (iter 5/10)
## INFO  [08:02:59.567] [mlr3] Applying learner 'regr.svm' on task 'PredictionSet[1:13]' (iter 6/10)
## INFO  [08:02:59.587] [mlr3] Applying learner 'regr.svm' on task 'PredictionSet[1:13]' (iter 7/10)
## INFO  [08:02:59.606] [mlr3] Applying learner 'regr.svm' on task 'PredictionSet[1:13]' (iter 8/10)
## INFO  [08:02:59.624] [mlr3] Applying learner 'regr.svm' on task 'PredictionSet[1:13]' (iter 9/10)
## INFO  [08:02:59.642] [mlr3] Applying learner 'regr.svm' on task 'PredictionSet[1:13]' (iter 10/10)
## INFO  [08:02:59.672] [mlr3] Finished benchmark
## INFO  [08:02:59.708] [bbotk] Result of batch 7:
## INFO  [08:02:59.710] [bbotk]  tolerance   gamma   epsilon           type  kernel regr.rmse warnings errors
## INFO  [08:02:59.710] [bbotk]  0.2578571 25.0005 0.2714286 eps-regression sigmoid  3.005331        0      0
## INFO  [08:02:59.710] [bbotk]  runtime_learners                                uhash
## INFO  [08:02:59.710] [bbotk]             0.092 3dd5474e-e681-4956-98e2-b4d519bc7e29
## INFO  [08:02:59.712] [bbotk] Evaluating 1 configuration(s)
## INFO  [08:02:59.727] [mlr3] Running benchmark with 10 resampling iterations
## INFO  [08:02:59.734] [mlr3] Applying learner 'regr.svm' on task 'PredictionSet[1:13]' (iter 1/10)
## INFO  [08:02:59.750] [mlr3] Applying learner 'regr.svm' on task 'PredictionSet[1:13]' (iter 2/10)
## INFO  [08:02:59.767] [mlr3] Applying learner 'regr.svm' on task 'PredictionSet[1:13]' (iter 3/10)
## INFO  [08:02:59.783] [mlr3] Applying learner 'regr.svm' on task 'PredictionSet[1:13]' (iter 4/10)
## INFO  [08:02:59.799] [mlr3] Applying learner 'regr.svm' on task 'PredictionSet[1:13]' (iter 5/10)
## INFO  [08:02:59.817] [mlr3] Applying learner 'regr.svm' on task 'PredictionSet[1:13]' (iter 6/10)
## INFO  [08:02:59.833] [mlr3] Applying learner 'regr.svm' on task 'PredictionSet[1:13]' (iter 7/10)
## INFO  [08:02:59.849] [mlr3] Applying learner 'regr.svm' on task 'PredictionSet[1:13]' (iter 8/10)
## INFO  [08:02:59.865] [mlr3] Applying learner 'regr.svm' on task 'PredictionSet[1:13]' (iter 9/10)
## INFO  [08:02:59.883] [mlr3] Applying learner 'regr.svm' on task 'PredictionSet[1:13]' (iter 10/10)
## INFO  [08:02:59.899] [mlr3] Finished benchmark
## INFO  [08:02:59.931] [bbotk] Result of batch 8:
## INFO  [08:02:59.933] [bbotk]  tolerance    gamma   epsilon           type kernel  regr.rmse warnings errors
## INFO  [08:02:59.933] [bbotk]  0.6431429 46.42864 0.5571429 eps-regression radial 0.08074691        0      0
## INFO  [08:02:59.933] [bbotk]  runtime_learners                                uhash
## INFO  [08:02:59.933] [bbotk]             0.066 16edefb6-8fc0-4e74-93bf-a73db6fad7ae
## INFO  [08:02:59.934] [bbotk] Evaluating 1 configuration(s)
## INFO  [08:02:59.950] [mlr3] Running benchmark with 10 resampling iterations
## INFO  [08:02:59.955] [mlr3] Applying learner 'regr.svm' on task 'PredictionSet[1:13]' (iter 1/10)
## INFO  [08:02:59.983] [mlr3] Applying learner 'regr.svm' on task 'PredictionSet[1:13]' (iter 2/10)
## INFO  [08:03:00.005] [mlr3] Applying learner 'regr.svm' on task 'PredictionSet[1:13]' (iter 3/10)
## INFO  [08:03:00.026] [mlr3] Applying learner 'regr.svm' on task 'PredictionSet[1:13]' (iter 4/10)
## INFO  [08:03:00.047] [mlr3] Applying learner 'regr.svm' on task 'PredictionSet[1:13]' (iter 5/10)
## INFO  [08:03:00.066] [mlr3] Applying learner 'regr.svm' on task 'PredictionSet[1:13]' (iter 6/10)
## INFO  [08:03:00.084] [mlr3] Applying learner 'regr.svm' on task 'PredictionSet[1:13]' (iter 7/10)
## INFO  [08:03:00.102] [mlr3] Applying learner 'regr.svm' on task 'PredictionSet[1:13]' (iter 8/10)
## INFO  [08:03:00.122] [mlr3] Applying learner 'regr.svm' on task 'PredictionSet[1:13]' (iter 9/10)
## INFO  [08:03:00.140] [mlr3] Applying learner 'regr.svm' on task 'PredictionSet[1:13]' (iter 10/10)
## INFO  [08:03:00.158] [mlr3] Finished benchmark
## INFO  [08:03:00.190] [bbotk] Result of batch 9:
## INFO  [08:03:00.192] [bbotk]  tolerance    gamma epsilon           type  kernel regr.rmse warnings errors
## INFO  [08:03:00.192] [bbotk]  0.8357857 42.85729     0.5 eps-regression sigmoid  2.994819        0      0
## INFO  [08:03:00.192] [bbotk]  runtime_learners                                uhash
## INFO  [08:03:00.192] [bbotk]             0.082 fe58b117-24c2-4b20-9e6d-c775c68ebc83
## INFO  [08:03:00.194] [bbotk] Evaluating 1 configuration(s)
## INFO  [08:03:00.209] [mlr3] Running benchmark with 10 resampling iterations
## INFO  [08:03:00.214] [mlr3] Applying learner 'regr.svm' on task 'PredictionSet[1:13]' (iter 1/10)
## INFO  [08:03:00.232] [mlr3] Applying learner 'regr.svm' on task 'PredictionSet[1:13]' (iter 2/10)
## INFO  [08:03:00.251] [mlr3] Applying learner 'regr.svm' on task 'PredictionSet[1:13]' (iter 3/10)
## INFO  [08:03:00.271] [mlr3] Applying learner 'regr.svm' on task 'PredictionSet[1:13]' (iter 4/10)
## INFO  [08:03:00.289] [mlr3] Applying learner 'regr.svm' on task 'PredictionSet[1:13]' (iter 5/10)
## INFO  [08:03:00.307] [mlr3] Applying learner 'regr.svm' on task 'PredictionSet[1:13]' (iter 6/10)
## INFO  [08:03:00.325] [mlr3] Applying learner 'regr.svm' on task 'PredictionSet[1:13]' (iter 7/10)
## INFO  [08:03:00.355] [mlr3] Applying learner 'regr.svm' on task 'PredictionSet[1:13]' (iter 8/10)
## INFO  [08:03:00.376] [mlr3] Applying learner 'regr.svm' on task 'PredictionSet[1:13]' (iter 9/10)
## INFO  [08:03:00.396] [mlr3] Applying learner 'regr.svm' on task 'PredictionSet[1:13]' (iter 10/10)
## INFO  [08:03:00.417] [mlr3] Finished benchmark
## INFO  [08:03:00.449] [bbotk] Result of batch 10:
## INFO  [08:03:00.450] [bbotk]  tolerance    gamma   epsilon           type  kernel regr.rmse warnings errors
## INFO  [08:03:00.450] [bbotk]  0.2578571 28.57186 0.7857143 eps-regression sigmoid  2.990865        0      0
## INFO  [08:03:00.450] [bbotk]  runtime_learners                                uhash
## INFO  [08:03:00.450] [bbotk]             0.096 eea84aea-3622-4d93-9e25-e2ee532b7a51
## INFO  [08:03:00.455] [bbotk] Finished optimizing after 10 evaluation(s)
## INFO  [08:03:00.456] [bbotk] Result:
## INFO  [08:03:00.457] [bbotk]  tolerance    gamma epsilon           type kernel learner_param_vals  x_domain
## INFO  [08:03:00.457] [bbotk]  0.1294286 28.57186     0.1 eps-regression radial          <list[5]> <list[5]>
## INFO  [08:03:00.457] [bbotk]   regr.rmse
## INFO  [08:03:00.457] [bbotk]  0.07060421
## INFO  [08:03:00.602] [bbotk] Starting to optimize 3 parameter(s) with '<TunerGridSearch>' and '<TerminatorEvals> [n_evals=10, k=0]'
## INFO  [08:03:00.605] [bbotk] Evaluating 1 configuration(s)
## INFO  [08:03:00.616] [mlr3] Running benchmark with 10 resampling iterations
## INFO  [08:03:00.621] [mlr3] Applying learner 'regr.kknn' on task 'PredictionSet[1:13]' (iter 1/10)
## INFO  [08:03:00.654] [mlr3] Applying learner 'regr.kknn' on task 'PredictionSet[1:13]' (iter 2/10)
## INFO  [08:03:00.678] [mlr3] Applying learner 'regr.kknn' on task 'PredictionSet[1:13]' (iter 3/10)
## INFO  [08:03:00.699] [mlr3] Applying learner 'regr.kknn' on task 'PredictionSet[1:13]' (iter 4/10)
## INFO  [08:03:00.722] [mlr3] Applying learner 'regr.kknn' on task 'PredictionSet[1:13]' (iter 5/10)
## INFO  [08:03:00.742] [mlr3] Applying learner 'regr.kknn' on task 'PredictionSet[1:13]' (iter 6/10)
## INFO  [08:03:00.763] [mlr3] Applying learner 'regr.kknn' on task 'PredictionSet[1:13]' (iter 7/10)
## INFO  [08:03:00.784] [mlr3] Applying learner 'regr.kknn' on task 'PredictionSet[1:13]' (iter 8/10)
## INFO  [08:03:00.805] [mlr3] Applying learner 'regr.kknn' on task 'PredictionSet[1:13]' (iter 9/10)
## INFO  [08:03:00.825] [mlr3] Applying learner 'regr.kknn' on task 'PredictionSet[1:13]' (iter 10/10)
## INFO  [08:03:00.845] [mlr3] Finished benchmark
## INFO  [08:03:00.877] [bbotk] Result of batch 1:
## INFO  [08:03:00.878] [bbotk]  k distance   kernel  regr.rmse warnings errors runtime_learners
## INFO  [08:03:00.878] [bbotk]  6 11.47143 gaussian 0.05795528        0      0            0.115
## INFO  [08:03:00.878] [bbotk]                                 uhash
## INFO  [08:03:00.878] [bbotk]  c18ad11d-71bb-4aba-85a4-e37e64b6a187
## INFO  [08:03:00.880] [bbotk] Evaluating 1 configuration(s)
## INFO  [08:03:00.891] [mlr3] Running benchmark with 10 resampling iterations
## INFO  [08:03:00.896] [mlr3] Applying learner 'regr.kknn' on task 'PredictionSet[1:13]' (iter 1/10)
## INFO  [08:03:00.916] [mlr3] Applying learner 'regr.kknn' on task 'PredictionSet[1:13]' (iter 2/10)
## INFO  [08:03:00.938] [mlr3] Applying learner 'regr.kknn' on task 'PredictionSet[1:13]' (iter 3/10)
## INFO  [08:03:00.958] [mlr3] Applying learner 'regr.kknn' on task 'PredictionSet[1:13]' (iter 4/10)
## INFO  [08:03:00.986] [mlr3] Applying learner 'regr.kknn' on task 'PredictionSet[1:13]' (iter 5/10)
## INFO  [08:03:01.011] [mlr3] Applying learner 'regr.kknn' on task 'PredictionSet[1:13]' (iter 6/10)
## INFO  [08:03:01.035] [mlr3] Applying learner 'regr.kknn' on task 'PredictionSet[1:13]' (iter 7/10)
## INFO  [08:03:01.056] [mlr3] Applying learner 'regr.kknn' on task 'PredictionSet[1:13]' (iter 8/10)
## INFO  [08:03:01.076] [mlr3] Applying learner 'regr.kknn' on task 'PredictionSet[1:13]' (iter 9/10)
## INFO  [08:03:01.098] [mlr3] Applying learner 'regr.kknn' on task 'PredictionSet[1:13]' (iter 10/10)
## INFO  [08:03:01.118] [mlr3] Finished benchmark
## INFO  [08:03:01.150] [bbotk] Result of batch 2:
## INFO  [08:03:01.151] [bbotk]  k distance   kernel regr.rmse warnings errors runtime_learners
## INFO  [08:03:01.151] [bbotk]  2 11.47143 biweight 0.0508602        0      0            0.106
## INFO  [08:03:01.151] [bbotk]                                 uhash
## INFO  [08:03:01.151] [bbotk]  b92f2ddc-0381-4a3a-8bf7-afaf9b9420b5
## INFO  [08:03:01.153] [bbotk] Evaluating 1 configuration(s)
## INFO  [08:03:01.164] [mlr3] Running benchmark with 10 resampling iterations
## INFO  [08:03:01.169] [mlr3] Applying learner 'regr.kknn' on task 'PredictionSet[1:13]' (iter 1/10)
## INFO  [08:03:01.190] [mlr3] Applying learner 'regr.kknn' on task 'PredictionSet[1:13]' (iter 2/10)
## INFO  [08:03:01.210] [mlr3] Applying learner 'regr.kknn' on task 'PredictionSet[1:13]' (iter 3/10)
## INFO  [08:03:01.231] [mlr3] Applying learner 'regr.kknn' on task 'PredictionSet[1:13]' (iter 4/10)
## INFO  [08:03:01.253] [mlr3] Applying learner 'regr.kknn' on task 'PredictionSet[1:13]' (iter 5/10)
## INFO  [08:03:01.273] [mlr3] Applying learner 'regr.kknn' on task 'PredictionSet[1:13]' (iter 6/10)
## INFO  [08:03:01.293] [mlr3] Applying learner 'regr.kknn' on task 'PredictionSet[1:13]' (iter 7/10)
## INFO  [08:03:01.316] [mlr3] Applying learner 'regr.kknn' on task 'PredictionSet[1:13]' (iter 8/10)
## INFO  [08:03:01.346] [mlr3] Applying learner 'regr.kknn' on task 'PredictionSet[1:13]' (iter 9/10)
## INFO  [08:03:01.370] [mlr3] Applying learner 'regr.kknn' on task 'PredictionSet[1:13]' (iter 10/10)
## INFO  [08:03:01.393] [mlr3] Finished benchmark
## INFO  [08:03:01.425] [bbotk] Result of batch 3:
## INFO  [08:03:01.426] [bbotk]   k distance    kernel  regr.rmse warnings errors runtime_learners
## INFO  [08:03:01.426] [bbotk]  10 1.521429 triweight 0.04984206        0      0            0.108
## INFO  [08:03:01.426] [bbotk]                                 uhash
## INFO  [08:03:01.426] [bbotk]  3432bb8c-aa0d-45d8-a7ef-88314ac5e550
## INFO  [08:03:01.428] [bbotk] Evaluating 1 configuration(s)
## INFO  [08:03:01.438] [mlr3] Running benchmark with 10 resampling iterations
## INFO  [08:03:01.443] [mlr3] Applying learner 'regr.kknn' on task 'PredictionSet[1:13]' (iter 1/10)
## INFO  [08:03:01.464] [mlr3] Applying learner 'regr.kknn' on task 'PredictionSet[1:13]' (iter 2/10)
## INFO  [08:03:01.485] [mlr3] Applying learner 'regr.kknn' on task 'PredictionSet[1:13]' (iter 3/10)
## INFO  [08:03:01.505] [mlr3] Applying learner 'regr.kknn' on task 'PredictionSet[1:13]' (iter 4/10)
## INFO  [08:03:01.526] [mlr3] Applying learner 'regr.kknn' on task 'PredictionSet[1:13]' (iter 5/10)
## INFO  [08:03:01.548] [mlr3] Applying learner 'regr.kknn' on task 'PredictionSet[1:13]' (iter 6/10)
## INFO  [08:03:01.569] [mlr3] Applying learner 'regr.kknn' on task 'PredictionSet[1:13]' (iter 7/10)
## INFO  [08:03:01.590] [mlr3] Applying learner 'regr.kknn' on task 'PredictionSet[1:13]' (iter 8/10)
## INFO  [08:03:01.611] [mlr3] Applying learner 'regr.kknn' on task 'PredictionSet[1:13]' (iter 9/10)
## INFO  [08:03:01.632] [mlr3] Applying learner 'regr.kknn' on task 'PredictionSet[1:13]' (iter 10/10)
## INFO  [08:03:01.652] [mlr3] Finished benchmark
## INFO  [08:03:01.698] [bbotk] Result of batch 4:
## INFO  [08:03:01.699] [bbotk]   k distance  kernel regr.rmse warnings errors runtime_learners
## INFO  [08:03:01.699] [bbotk]  16 15.73571 optimal 0.0569935        0      0            0.104
## INFO  [08:03:01.699] [bbotk]                                 uhash
## INFO  [08:03:01.699] [bbotk]  5e98d66b-f3dd-4ccc-839e-b5e9765b97dd
## INFO  [08:03:01.701] [bbotk] Evaluating 1 configuration(s)
## INFO  [08:03:01.713] [mlr3] Running benchmark with 10 resampling iterations
## INFO  [08:03:01.719] [mlr3] Applying learner 'regr.kknn' on task 'PredictionSet[1:13]' (iter 1/10)
## INFO  [08:03:01.739] [mlr3] Applying learner 'regr.kknn' on task 'PredictionSet[1:13]' (iter 2/10)
## INFO  [08:03:01.761] [mlr3] Applying learner 'regr.kknn' on task 'PredictionSet[1:13]' (iter 3/10)
## INFO  [08:03:01.782] [mlr3] Applying learner 'regr.kknn' on task 'PredictionSet[1:13]' (iter 4/10)
## INFO  [08:03:01.802] [mlr3] Applying learner 'regr.kknn' on task 'PredictionSet[1:13]' (iter 5/10)
## INFO  [08:03:01.823] [mlr3] Applying learner 'regr.kknn' on task 'PredictionSet[1:13]' (iter 6/10)
## INFO  [08:03:01.845] [mlr3] Applying learner 'regr.kknn' on task 'PredictionSet[1:13]' (iter 7/10)
## INFO  [08:03:01.866] [mlr3] Applying learner 'regr.kknn' on task 'PredictionSet[1:13]' (iter 8/10)
## INFO  [08:03:01.886] [mlr3] Applying learner 'regr.kknn' on task 'PredictionSet[1:13]' (iter 9/10)
## INFO  [08:03:01.907] [mlr3] Applying learner 'regr.kknn' on task 'PredictionSet[1:13]' (iter 10/10)
## INFO  [08:03:01.929] [mlr3] Finished benchmark
## INFO  [08:03:01.961] [bbotk] Result of batch 5:
## INFO  [08:03:01.963] [bbotk]   k distance      kernel  regr.rmse warnings errors runtime_learners
## INFO  [08:03:01.963] [bbotk]  18 12.89286 rectangular 0.06690244        0      0            0.107
## INFO  [08:03:01.963] [bbotk]                                 uhash
## INFO  [08:03:01.963] [bbotk]  6ab4b498-c431-4fab-90f7-2ad377020d05
## INFO  [08:03:01.965] [bbotk] Evaluating 1 configuration(s)
## INFO  [08:03:01.976] [mlr3] Running benchmark with 10 resampling iterations
## INFO  [08:03:01.981] [mlr3] Applying learner 'regr.kknn' on task 'PredictionSet[1:13]' (iter 1/10)
## INFO  [08:03:02.011] [mlr3] Applying learner 'regr.kknn' on task 'PredictionSet[1:13]' (iter 2/10)
## INFO  [08:03:02.034] [mlr3] Applying learner 'regr.kknn' on task 'PredictionSet[1:13]' (iter 3/10)
## INFO  [08:03:02.057] [mlr3] Applying learner 'regr.kknn' on task 'PredictionSet[1:13]' (iter 4/10)
## INFO  [08:03:02.079] [mlr3] Applying learner 'regr.kknn' on task 'PredictionSet[1:13]' (iter 5/10)
## INFO  [08:03:02.100] [mlr3] Applying learner 'regr.kknn' on task 'PredictionSet[1:13]' (iter 6/10)
## INFO  [08:03:02.120] [mlr3] Applying learner 'regr.kknn' on task 'PredictionSet[1:13]' (iter 7/10)
## INFO  [08:03:02.142] [mlr3] Applying learner 'regr.kknn' on task 'PredictionSet[1:13]' (iter 8/10)
## INFO  [08:03:02.163] [mlr3] Applying learner 'regr.kknn' on task 'PredictionSet[1:13]' (iter 9/10)
## INFO  [08:03:02.183] [mlr3] Applying learner 'regr.kknn' on task 'PredictionSet[1:13]' (iter 10/10)
## INFO  [08:03:02.203] [mlr3] Finished benchmark
## INFO  [08:03:02.236] [bbotk] Result of batch 6:
## INFO  [08:03:02.238] [bbotk]   k distance   kernel  regr.rmse warnings errors runtime_learners
## INFO  [08:03:02.238] [bbotk]  12 11.47143 gaussian 0.05938875        0      0            0.111
## INFO  [08:03:02.238] [bbotk]                                 uhash
## INFO  [08:03:02.238] [bbotk]  2a4660a3-2758-4e8e-baf8-bbcc88727c3b
## INFO  [08:03:02.240] [bbotk] Evaluating 1 configuration(s)
## INFO  [08:03:02.251] [mlr3] Running benchmark with 10 resampling iterations
## INFO  [08:03:02.256] [mlr3] Applying learner 'regr.kknn' on task 'PredictionSet[1:13]' (iter 1/10)
## INFO  [08:03:02.276] [mlr3] Applying learner 'regr.kknn' on task 'PredictionSet[1:13]' (iter 2/10)
## INFO  [08:03:02.299] [mlr3] Applying learner 'regr.kknn' on task 'PredictionSet[1:13]' (iter 3/10)
## INFO  [08:03:02.320] [mlr3] Applying learner 'regr.kknn' on task 'PredictionSet[1:13]' (iter 4/10)
## INFO  [08:03:02.380] [mlr3] Applying learner 'regr.kknn' on task 'PredictionSet[1:13]' (iter 5/10)
## INFO  [08:03:02.409] [mlr3] Applying learner 'regr.kknn' on task 'PredictionSet[1:13]' (iter 6/10)
## INFO  [08:03:02.437] [mlr3] Applying learner 'regr.kknn' on task 'PredictionSet[1:13]' (iter 7/10)
## INFO  [08:03:02.463] [mlr3] Applying learner 'regr.kknn' on task 'PredictionSet[1:13]' (iter 8/10)
## INFO  [08:03:02.487] [mlr3] Applying learner 'regr.kknn' on task 'PredictionSet[1:13]' (iter 9/10)
## INFO  [08:03:02.510] [mlr3] Applying learner 'regr.kknn' on task 'PredictionSet[1:13]' (iter 10/10)
## INFO  [08:03:02.531] [mlr3] Finished benchmark
## INFO  [08:03:02.562] [bbotk] Result of batch 7:
## INFO  [08:03:02.564] [bbotk]  k distance   kernel regr.rmse warnings errors runtime_learners
## INFO  [08:03:02.564] [bbotk]  9 11.47143 biweight 0.0548976        0      0            0.116
## INFO  [08:03:02.564] [bbotk]                                 uhash
## INFO  [08:03:02.564] [bbotk]  a4f26833-366c-4c15-92ba-82c04498b306
## INFO  [08:03:02.565] [bbotk] Evaluating 1 configuration(s)
## INFO  [08:03:02.576] [mlr3] Running benchmark with 10 resampling iterations
## INFO  [08:03:02.582] [mlr3] Applying learner 'regr.kknn' on task 'PredictionSet[1:13]' (iter 1/10)
## INFO  [08:03:02.604] [mlr3] Applying learner 'regr.kknn' on task 'PredictionSet[1:13]' (iter 2/10)
## INFO  [08:03:02.624] [mlr3] Applying learner 'regr.kknn' on task 'PredictionSet[1:13]' (iter 3/10)
## INFO  [08:03:02.644] [mlr3] Applying learner 'regr.kknn' on task 'PredictionSet[1:13]' (iter 4/10)
## INFO  [08:03:02.667] [mlr3] Applying learner 'regr.kknn' on task 'PredictionSet[1:13]' (iter 5/10)
## INFO  [08:03:02.688] [mlr3] Applying learner 'regr.kknn' on task 'PredictionSet[1:13]' (iter 6/10)
## INFO  [08:03:02.709] [mlr3] Applying learner 'regr.kknn' on task 'PredictionSet[1:13]' (iter 7/10)
## INFO  [08:03:02.730] [mlr3] Applying learner 'regr.kknn' on task 'PredictionSet[1:13]' (iter 8/10)
## INFO  [08:03:02.752] [mlr3] Applying learner 'regr.kknn' on task 'PredictionSet[1:13]' (iter 9/10)
## INFO  [08:03:02.773] [mlr3] Applying learner 'regr.kknn' on task 'PredictionSet[1:13]' (iter 10/10)
## INFO  [08:03:02.804] [mlr3] Finished benchmark
## INFO  [08:03:02.845] [bbotk] Result of batch 8:
## INFO  [08:03:02.847] [bbotk]  k distance     kernel regr.rmse warnings errors runtime_learners
## INFO  [08:03:02.847] [bbotk]  9 7.207143 triangular 0.0560325        0      0            0.106
## INFO  [08:03:02.847] [bbotk]                                 uhash
## INFO  [08:03:02.847] [bbotk]  080806a9-d4a0-4d83-a5f9-9d38a3ed4b1b
## INFO  [08:03:02.849] [bbotk] Evaluating 1 configuration(s)
## INFO  [08:03:02.861] [mlr3] Running benchmark with 10 resampling iterations
## INFO  [08:03:02.866] [mlr3] Applying learner 'regr.kknn' on task 'PredictionSet[1:13]' (iter 1/10)
## INFO  [08:03:02.889] [mlr3] Applying learner 'regr.kknn' on task 'PredictionSet[1:13]' (iter 2/10)
## INFO  [08:03:02.910] [mlr3] Applying learner 'regr.kknn' on task 'PredictionSet[1:13]' (iter 3/10)
## INFO  [08:03:02.930] [mlr3] Applying learner 'regr.kknn' on task 'PredictionSet[1:13]' (iter 4/10)
## INFO  [08:03:02.951] [mlr3] Applying learner 'regr.kknn' on task 'PredictionSet[1:13]' (iter 5/10)
## INFO  [08:03:02.973] [mlr3] Applying learner 'regr.kknn' on task 'PredictionSet[1:13]' (iter 6/10)
## INFO  [08:03:02.994] [mlr3] Applying learner 'regr.kknn' on task 'PredictionSet[1:13]' (iter 7/10)
## INFO  [08:03:03.014] [mlr3] Applying learner 'regr.kknn' on task 'PredictionSet[1:13]' (iter 8/10)
## INFO  [08:03:03.036] [mlr3] Applying learner 'regr.kknn' on task 'PredictionSet[1:13]' (iter 9/10)
## INFO  [08:03:03.057] [mlr3] Applying learner 'regr.kknn' on task 'PredictionSet[1:13]' (iter 10/10)
## INFO  [08:03:03.078] [mlr3] Finished benchmark
## INFO  [08:03:03.111] [bbotk] Result of batch 9:
## INFO  [08:03:03.113] [bbotk]   k distance     kernel  regr.rmse warnings errors runtime_learners
## INFO  [08:03:03.113] [bbotk]  16 12.89286 triangular 0.05851896        0      0            0.106
## INFO  [08:03:03.113] [bbotk]                                 uhash
## INFO  [08:03:03.113] [bbotk]  4f816078-93a5-4221-b0af-476578a67558
## INFO  [08:03:03.115] [bbotk] Evaluating 1 configuration(s)
## INFO  [08:03:03.126] [mlr3] Running benchmark with 10 resampling iterations
## INFO  [08:03:03.131] [mlr3] Applying learner 'regr.kknn' on task 'PredictionSet[1:13]' (iter 1/10)
## INFO  [08:03:03.151] [mlr3] Applying learner 'regr.kknn' on task 'PredictionSet[1:13]' (iter 2/10)
## INFO  [08:03:03.183] [mlr3] Applying learner 'regr.kknn' on task 'PredictionSet[1:13]' (iter 3/10)
## INFO  [08:03:03.211] [mlr3] Applying learner 'regr.kknn' on task 'PredictionSet[1:13]' (iter 4/10)
## INFO  [08:03:03.240] [mlr3] Applying learner 'regr.kknn' on task 'PredictionSet[1:13]' (iter 5/10)
## INFO  [08:03:03.265] [mlr3] Applying learner 'regr.kknn' on task 'PredictionSet[1:13]' (iter 6/10)
## INFO  [08:03:03.288] [mlr3] Applying learner 'regr.kknn' on task 'PredictionSet[1:13]' (iter 7/10)
## INFO  [08:03:03.310] [mlr3] Applying learner 'regr.kknn' on task 'PredictionSet[1:13]' (iter 8/10)
## INFO  [08:03:03.331] [mlr3] Applying learner 'regr.kknn' on task 'PredictionSet[1:13]' (iter 9/10)
## INFO  [08:03:03.353] [mlr3] Applying learner 'regr.kknn' on task 'PredictionSet[1:13]' (iter 10/10)
## INFO  [08:03:03.373] [mlr3] Finished benchmark
## INFO  [08:03:03.405] [bbotk] Result of batch 10:
## INFO  [08:03:03.407] [bbotk]  k distance       kernel  regr.rmse warnings errors runtime_learners
## INFO  [08:03:03.407] [bbotk]  3 12.89286 epanechnikov 0.05057202        0      0             0.11
## INFO  [08:03:03.407] [bbotk]                                 uhash
## INFO  [08:03:03.407] [bbotk]  8fdde41d-f405-4256-9fe6-ac5b3dfe8d31
## INFO  [08:03:03.412] [bbotk] Finished optimizing after 10 evaluation(s)
## INFO  [08:03:03.413] [bbotk] Result:
## INFO  [08:03:03.415] [bbotk]   k distance    kernel learner_param_vals  x_domain  regr.rmse
## INFO  [08:03:03.415] [bbotk]  10 1.521429 triweight          <list[3]> <list[3]> 0.04984206
## INFO  [08:03:03.649] [bbotk] Starting to optimize 2 parameter(s) with '<TunerGridSearch>' and '<TerminatorEvals> [n_evals=10, k=0]'
## INFO  [08:03:03.652] [bbotk] Evaluating 1 configuration(s)
## INFO  [08:03:03.674] [mlr3] Running benchmark with 10 resampling iterations
## INFO  [08:03:03.679] [mlr3] Applying learner 'regr.ranger' on task 'PredictionSet[1:13]' (iter 1/10)
## INFO  [08:03:03.703] [mlr3] Applying learner 'regr.ranger' on task 'PredictionSet[1:13]' (iter 2/10)
## INFO  [08:03:03.728] [mlr3] Applying learner 'regr.ranger' on task 'PredictionSet[1:13]' (iter 3/10)
## INFO  [08:03:03.751] [mlr3] Applying learner 'regr.ranger' on task 'PredictionSet[1:13]' (iter 4/10)
## INFO  [08:03:03.775] [mlr3] Applying learner 'regr.ranger' on task 'PredictionSet[1:13]' (iter 5/10)
## INFO  [08:03:03.800] [mlr3] Applying learner 'regr.ranger' on task 'PredictionSet[1:13]' (iter 6/10)
## INFO  [08:03:03.824] [mlr3] Applying learner 'regr.ranger' on task 'PredictionSet[1:13]' (iter 7/10)
## INFO  [08:03:03.847] [mlr3] Applying learner 'regr.ranger' on task 'PredictionSet[1:13]' (iter 8/10)
## INFO  [08:03:03.872] [mlr3] Applying learner 'regr.ranger' on task 'PredictionSet[1:13]' (iter 9/10)
## INFO  [08:03:03.903] [mlr3] Applying learner 'regr.ranger' on task 'PredictionSet[1:13]' (iter 10/10)
## INFO  [08:03:03.931] [mlr3] Finished benchmark
## INFO  [08:03:03.969] [bbotk] Result of batch 1:
## INFO  [08:03:03.971] [bbotk]  max.depth num.trees  regr.rmse warnings errors runtime_learners
## INFO  [08:03:03.971] [bbotk]          2       290 0.07631511        0      0            0.137
## INFO  [08:03:03.971] [bbotk]                                 uhash
## INFO  [08:03:03.971] [bbotk]  5064f501-db33-4086-a2b3-c0a7288d1eb2
## INFO  [08:03:03.972] [bbotk] Evaluating 1 configuration(s)
## INFO  [08:03:03.995] [mlr3] Running benchmark with 10 resampling iterations
## INFO  [08:03:04.001] [mlr3] Applying learner 'regr.ranger' on task 'PredictionSet[1:13]' (iter 1/10)
## INFO  [08:03:04.019] [mlr3] Applying learner 'regr.ranger' on task 'PredictionSet[1:13]' (iter 2/10)
## INFO  [08:03:04.036] [mlr3] Applying learner 'regr.ranger' on task 'PredictionSet[1:13]' (iter 3/10)
## INFO  [08:03:04.053] [mlr3] Applying learner 'regr.ranger' on task 'PredictionSet[1:13]' (iter 4/10)
## INFO  [08:03:04.069] [mlr3] Applying learner 'regr.ranger' on task 'PredictionSet[1:13]' (iter 5/10)
## INFO  [08:03:04.088] [mlr3] Applying learner 'regr.ranger' on task 'PredictionSet[1:13]' (iter 6/10)
## INFO  [08:03:04.105] [mlr3] Applying learner 'regr.ranger' on task 'PredictionSet[1:13]' (iter 7/10)
## INFO  [08:03:04.122] [mlr3] Applying learner 'regr.ranger' on task 'PredictionSet[1:13]' (iter 8/10)
## INFO  [08:03:04.138] [mlr3] Applying learner 'regr.ranger' on task 'PredictionSet[1:13]' (iter 9/10)
## INFO  [08:03:04.156] [mlr3] Applying learner 'regr.ranger' on task 'PredictionSet[1:13]' (iter 10/10)
## INFO  [08:03:04.174] [mlr3] Finished benchmark
## INFO  [08:03:04.206] [bbotk] Result of batch 2:
## INFO  [08:03:04.207] [bbotk]  max.depth num.trees  regr.rmse warnings errors runtime_learners
## INFO  [08:03:04.207] [bbotk]          1        79 0.08237479        0      0            0.071
## INFO  [08:03:04.207] [bbotk]                                 uhash
## INFO  [08:03:04.207] [bbotk]  8bca443d-1747-43dd-9d1a-99d7df479b25
## INFO  [08:03:04.209] [bbotk] Evaluating 1 configuration(s)
## INFO  [08:03:04.231] [mlr3] Running benchmark with 10 resampling iterations
## INFO  [08:03:04.237] [mlr3] Applying learner 'regr.ranger' on task 'PredictionSet[1:13]' (iter 1/10)
## INFO  [08:03:04.285] [mlr3] Applying learner 'regr.ranger' on task 'PredictionSet[1:13]' (iter 2/10)
## INFO  [08:03:04.327] [mlr3] Applying learner 'regr.ranger' on task 'PredictionSet[1:13]' (iter 3/10)
## INFO  [08:03:04.368] [mlr3] Applying learner 'regr.ranger' on task 'PredictionSet[1:13]' (iter 4/10)
## INFO  [08:03:04.410] [mlr3] Applying learner 'regr.ranger' on task 'PredictionSet[1:13]' (iter 5/10)
## INFO  [08:03:04.447] [mlr3] Applying learner 'regr.ranger' on task 'PredictionSet[1:13]' (iter 6/10)
## INFO  [08:03:04.486] [mlr3] Applying learner 'regr.ranger' on task 'PredictionSet[1:13]' (iter 7/10)
## INFO  [08:03:04.523] [mlr3] Applying learner 'regr.ranger' on task 'PredictionSet[1:13]' (iter 8/10)
## INFO  [08:03:04.562] [mlr3] Applying learner 'regr.ranger' on task 'PredictionSet[1:13]' (iter 9/10)
## INFO  [08:03:04.599] [mlr3] Applying learner 'regr.ranger' on task 'PredictionSet[1:13]' (iter 10/10)
## INFO  [08:03:04.636] [mlr3] Finished benchmark
## INFO  [08:03:04.668] [bbotk] Result of batch 3:
## INFO  [08:03:04.669] [bbotk]  max.depth num.trees  regr.rmse warnings errors runtime_learners
## INFO  [08:03:04.669] [bbotk]          4       500 0.06649119        0      0            0.285
## INFO  [08:03:04.669] [bbotk]                                 uhash
## INFO  [08:03:04.669] [bbotk]  3d8dd0e5-6e82-4a94-b491-a9137fc09b70
## INFO  [08:03:04.671] [bbotk] Evaluating 1 configuration(s)
## INFO  [08:03:04.694] [mlr3] Running benchmark with 10 resampling iterations
## INFO  [08:03:04.699] [mlr3] Applying learner 'regr.ranger' on task 'PredictionSet[1:13]' (iter 1/10)
## INFO  [08:03:04.715] [mlr3] Applying learner 'regr.ranger' on task 'PredictionSet[1:13]' (iter 2/10)
## INFO  [08:03:04.731] [mlr3] Applying learner 'regr.ranger' on task 'PredictionSet[1:13]' (iter 3/10)
## INFO  [08:03:04.748] [mlr3] Applying learner 'regr.ranger' on task 'PredictionSet[1:13]' (iter 4/10)
## INFO  [08:03:04.766] [mlr3] Applying learner 'regr.ranger' on task 'PredictionSet[1:13]' (iter 5/10)
## INFO  [08:03:04.783] [mlr3] Applying learner 'regr.ranger' on task 'PredictionSet[1:13]' (iter 6/10)
## INFO  [08:03:04.799] [mlr3] Applying learner 'regr.ranger' on task 'PredictionSet[1:13]' (iter 7/10)
## INFO  [08:03:04.827] [mlr3] Applying learner 'regr.ranger' on task 'PredictionSet[1:13]' (iter 8/10)
## INFO  [08:03:04.848] [mlr3] Applying learner 'regr.ranger' on task 'PredictionSet[1:13]' (iter 9/10)
## INFO  [08:03:04.868] [mlr3] Applying learner 'regr.ranger' on task 'PredictionSet[1:13]' (iter 10/10)
## INFO  [08:03:04.885] [mlr3] Finished benchmark
## INFO  [08:03:04.920] [bbotk] Result of batch 4:
## INFO  [08:03:04.922] [bbotk]  max.depth num.trees  regr.rmse warnings errors runtime_learners
## INFO  [08:03:04.922] [bbotk]          3        27 0.07160683        0      0            0.071
## INFO  [08:03:04.922] [bbotk]                                 uhash
## INFO  [08:03:04.922] [bbotk]  c3dc8b0a-a191-46c0-9192-d92e66494d7c
## INFO  [08:03:04.924] [bbotk] Evaluating 1 configuration(s)
## INFO  [08:03:04.945] [mlr3] Running benchmark with 10 resampling iterations
## INFO  [08:03:04.950] [mlr3] Applying learner 'regr.ranger' on task 'PredictionSet[1:13]' (iter 1/10)
## INFO  [08:03:04.967] [mlr3] Applying learner 'regr.ranger' on task 'PredictionSet[1:13]' (iter 2/10)
## INFO  [08:03:04.987] [mlr3] Applying learner 'regr.ranger' on task 'PredictionSet[1:13]' (iter 3/10)
## INFO  [08:03:05.005] [mlr3] Applying learner 'regr.ranger' on task 'PredictionSet[1:13]' (iter 4/10)
## INFO  [08:03:05.022] [mlr3] Applying learner 'regr.ranger' on task 'PredictionSet[1:13]' (iter 5/10)
## INFO  [08:03:05.039] [mlr3] Applying learner 'regr.ranger' on task 'PredictionSet[1:13]' (iter 6/10)
## INFO  [08:03:05.058] [mlr3] Applying learner 'regr.ranger' on task 'PredictionSet[1:13]' (iter 7/10)
## INFO  [08:03:05.077] [mlr3] Applying learner 'regr.ranger' on task 'PredictionSet[1:13]' (iter 8/10)
## INFO  [08:03:05.094] [mlr3] Applying learner 'regr.ranger' on task 'PredictionSet[1:13]' (iter 9/10)
## INFO  [08:03:05.112] [mlr3] Applying learner 'regr.ranger' on task 'PredictionSet[1:13]' (iter 10/10)
## INFO  [08:03:05.129] [mlr3] Finished benchmark
## INFO  [08:03:05.170] [bbotk] Result of batch 5:
## INFO  [08:03:05.172] [bbotk]  max.depth num.trees  regr.rmse warnings errors runtime_learners
## INFO  [08:03:05.172] [bbotk]          1       106 0.08230617        0      0            0.074
## INFO  [08:03:05.172] [bbotk]                                 uhash
## INFO  [08:03:05.172] [bbotk]  2d509b1b-e97c-4142-a4f9-81e77af7311b
## INFO  [08:03:05.173] [bbotk] Evaluating 1 configuration(s)
## INFO  [08:03:05.203] [mlr3] Running benchmark with 10 resampling iterations
## INFO  [08:03:05.210] [mlr3] Applying learner 'regr.ranger' on task 'PredictionSet[1:13]' (iter 1/10)
## INFO  [08:03:05.227] [mlr3] Applying learner 'regr.ranger' on task 'PredictionSet[1:13]' (iter 2/10)
## INFO  [08:03:05.243] [mlr3] Applying learner 'regr.ranger' on task 'PredictionSet[1:13]' (iter 3/10)
## INFO  [08:03:05.258] [mlr3] Applying learner 'regr.ranger' on task 'PredictionSet[1:13]' (iter 4/10)
## INFO  [08:03:05.273] [mlr3] Applying learner 'regr.ranger' on task 'PredictionSet[1:13]' (iter 5/10)
## INFO  [08:03:05.290] [mlr3] Applying learner 'regr.ranger' on task 'PredictionSet[1:13]' (iter 6/10)
## INFO  [08:03:05.306] [mlr3] Applying learner 'regr.ranger' on task 'PredictionSet[1:13]' (iter 7/10)
## INFO  [08:03:05.321] [mlr3] Applying learner 'regr.ranger' on task 'PredictionSet[1:13]' (iter 8/10)
## INFO  [08:03:05.335] [mlr3] Applying learner 'regr.ranger' on task 'PredictionSet[1:13]' (iter 9/10)
## INFO  [08:03:05.350] [mlr3] Applying learner 'regr.ranger' on task 'PredictionSet[1:13]' (iter 10/10)
## INFO  [08:03:05.367] [mlr3] Finished benchmark
## INFO  [08:03:05.399] [bbotk] Result of batch 6:
## INFO  [08:03:05.400] [bbotk]  max.depth num.trees regr.rmse warnings errors runtime_learners
## INFO  [08:03:05.400] [bbotk]         17         1 0.0703971        0      0             0.05
## INFO  [08:03:05.400] [bbotk]                                 uhash
## INFO  [08:03:05.400] [bbotk]  f9cf5ab8-05bb-4f7c-93ef-01561d33bdf9
## INFO  [08:03:05.402] [bbotk] Evaluating 1 configuration(s)
## INFO  [08:03:05.424] [mlr3] Running benchmark with 10 resampling iterations
## INFO  [08:03:05.429] [mlr3] Applying learner 'regr.ranger' on task 'PredictionSet[1:13]' (iter 1/10)
## INFO  [08:03:05.464] [mlr3] Applying learner 'regr.ranger' on task 'PredictionSet[1:13]' (iter 2/10)
## INFO  [08:03:05.497] [mlr3] Applying learner 'regr.ranger' on task 'PredictionSet[1:13]' (iter 3/10)
## INFO  [08:03:05.539] [mlr3] Applying learner 'regr.ranger' on task 'PredictionSet[1:13]' (iter 4/10)
## INFO  [08:03:05.577] [mlr3] Applying learner 'regr.ranger' on task 'PredictionSet[1:13]' (iter 5/10)
## INFO  [08:03:05.616] [mlr3] Applying learner 'regr.ranger' on task 'PredictionSet[1:13]' (iter 6/10)
## INFO  [08:03:05.653] [mlr3] Applying learner 'regr.ranger' on task 'PredictionSet[1:13]' (iter 7/10)
## INFO  [08:03:05.688] [mlr3] Applying learner 'regr.ranger' on task 'PredictionSet[1:13]' (iter 8/10)
## INFO  [08:03:05.721] [mlr3] Applying learner 'regr.ranger' on task 'PredictionSet[1:13]' (iter 9/10)
## INFO  [08:03:05.756] [mlr3] Applying learner 'regr.ranger' on task 'PredictionSet[1:13]' (iter 10/10)
## INFO  [08:03:05.789] [mlr3] Finished benchmark
## INFO  [08:03:05.822] [bbotk] Result of batch 7:
## INFO  [08:03:05.824] [bbotk]  max.depth num.trees  regr.rmse warnings errors runtime_learners
## INFO  [08:03:05.824] [bbotk]          8       264 0.05601802        0      0            0.237
## INFO  [08:03:05.824] [bbotk]                                 uhash
## INFO  [08:03:05.824] [bbotk]  c062101e-6b63-43c3-9574-be2f332d687b
## INFO  [08:03:05.825] [bbotk] Evaluating 1 configuration(s)
## INFO  [08:03:05.847] [mlr3] Running benchmark with 10 resampling iterations
## INFO  [08:03:05.852] [mlr3] Applying learner 'regr.ranger' on task 'PredictionSet[1:13]' (iter 1/10)
## INFO  [08:03:05.875] [mlr3] Applying learner 'regr.ranger' on task 'PredictionSet[1:13]' (iter 2/10)
## INFO  [08:03:05.899] [mlr3] Applying learner 'regr.ranger' on task 'PredictionSet[1:13]' (iter 3/10)
## INFO  [08:03:05.921] [mlr3] Applying learner 'regr.ranger' on task 'PredictionSet[1:13]' (iter 4/10)
## INFO  [08:03:05.943] [mlr3] Applying learner 'regr.ranger' on task 'PredictionSet[1:13]' (iter 5/10)
## INFO  [08:03:05.967] [mlr3] Applying learner 'regr.ranger' on task 'PredictionSet[1:13]' (iter 6/10)
## INFO  [08:03:05.990] [mlr3] Applying learner 'regr.ranger' on task 'PredictionSet[1:13]' (iter 7/10)
## INFO  [08:03:06.012] [mlr3] Applying learner 'regr.ranger' on task 'PredictionSet[1:13]' (iter 8/10)
## INFO  [08:03:06.035] [mlr3] Applying learner 'regr.ranger' on task 'PredictionSet[1:13]' (iter 9/10)
## INFO  [08:03:06.069] [mlr3] Applying learner 'regr.ranger' on task 'PredictionSet[1:13]' (iter 10/10)
## INFO  [08:03:06.094] [mlr3] Finished benchmark
## INFO  [08:03:06.135] [bbotk] Result of batch 8:
## INFO  [08:03:06.137] [bbotk]  max.depth num.trees regr.rmse warnings errors runtime_learners
## INFO  [08:03:06.137] [bbotk]          5       132 0.0616988        0      0            0.127
## INFO  [08:03:06.137] [bbotk]                                 uhash
## INFO  [08:03:06.137] [bbotk]  65832584-83ff-4e39-936c-1d50cb48871c
## INFO  [08:03:06.138] [bbotk] Evaluating 1 configuration(s)
## INFO  [08:03:06.160] [mlr3] Running benchmark with 10 resampling iterations
## INFO  [08:03:06.165] [mlr3] Applying learner 'regr.ranger' on task 'PredictionSet[1:13]' (iter 1/10)
## INFO  [08:03:06.212] [mlr3] Applying learner 'regr.ranger' on task 'PredictionSet[1:13]' (iter 2/10)
## INFO  [08:03:06.257] [mlr3] Applying learner 'regr.ranger' on task 'PredictionSet[1:13]' (iter 3/10)
## INFO  [08:03:06.302] [mlr3] Applying learner 'regr.ranger' on task 'PredictionSet[1:13]' (iter 4/10)
## INFO  [08:03:06.348] [mlr3] Applying learner 'regr.ranger' on task 'PredictionSet[1:13]' (iter 5/10)
## INFO  [08:03:06.392] [mlr3] Applying learner 'regr.ranger' on task 'PredictionSet[1:13]' (iter 6/10)
## INFO  [08:03:06.438] [mlr3] Applying learner 'regr.ranger' on task 'PredictionSet[1:13]' (iter 7/10)
## INFO  [08:03:06.483] [mlr3] Applying learner 'regr.ranger' on task 'PredictionSet[1:13]' (iter 8/10)
## INFO  [08:03:06.528] [mlr3] Applying learner 'regr.ranger' on task 'PredictionSet[1:13]' (iter 9/10)
## INFO  [08:03:06.574] [mlr3] Applying learner 'regr.ranger' on task 'PredictionSet[1:13]' (iter 10/10)
## INFO  [08:03:06.618] [mlr3] Finished benchmark
## INFO  [08:03:06.652] [bbotk] Result of batch 9:
## INFO  [08:03:06.653] [bbotk]  max.depth num.trees  regr.rmse warnings errors runtime_learners
## INFO  [08:03:06.653] [bbotk]         17       422 0.05553635        0      0            0.347
## INFO  [08:03:06.653] [bbotk]                                 uhash
## INFO  [08:03:06.653] [bbotk]  0cc3ab3d-87a9-480d-8a63-27626fdf64aa
## INFO  [08:03:06.655] [bbotk] Evaluating 1 configuration(s)
## INFO  [08:03:06.689] [mlr3] Running benchmark with 10 resampling iterations
## INFO  [08:03:06.695] [mlr3] Applying learner 'regr.ranger' on task 'PredictionSet[1:13]' (iter 1/10)
## INFO  [08:03:06.748] [mlr3] Applying learner 'regr.ranger' on task 'PredictionSet[1:13]' (iter 2/10)
## INFO  [08:03:06.800] [mlr3] Applying learner 'regr.ranger' on task 'PredictionSet[1:13]' (iter 3/10)
## INFO  [08:03:06.848] [mlr3] Applying learner 'regr.ranger' on task 'PredictionSet[1:13]' (iter 4/10)
## INFO  [08:03:06.897] [mlr3] Applying learner 'regr.ranger' on task 'PredictionSet[1:13]' (iter 5/10)
## INFO  [08:03:06.947] [mlr3] Applying learner 'regr.ranger' on task 'PredictionSet[1:13]' (iter 6/10)
## INFO  [08:03:06.994] [mlr3] Applying learner 'regr.ranger' on task 'PredictionSet[1:13]' (iter 7/10)
## INFO  [08:03:07.042] [mlr3] Applying learner 'regr.ranger' on task 'PredictionSet[1:13]' (iter 8/10)
## INFO  [08:03:07.092] [mlr3] Applying learner 'regr.ranger' on task 'PredictionSet[1:13]' (iter 9/10)
## INFO  [08:03:07.138] [mlr3] Applying learner 'regr.ranger' on task 'PredictionSet[1:13]' (iter 10/10)
## INFO  [08:03:07.186] [mlr3] Finished benchmark
## INFO  [08:03:07.217] [bbotk] Result of batch 10:
## INFO  [08:03:07.219] [bbotk]  max.depth num.trees  regr.rmse warnings errors runtime_learners
## INFO  [08:03:07.219] [bbotk]          8       474 0.05586556        0      0             0.38
## INFO  [08:03:07.219] [bbotk]                                 uhash
## INFO  [08:03:07.219] [bbotk]  e46192aa-ebb3-4f8b-9777-58eb9bcb0b14
## INFO  [08:03:07.224] [bbotk] Finished optimizing after 10 evaluation(s)
## INFO  [08:03:07.225] [bbotk] Result:
## INFO  [08:03:07.226] [bbotk]  max.depth num.trees learner_param_vals  x_domain  regr.rmse
## INFO  [08:03:07.226] [bbotk]         17       422          <list[3]> <list[2]> 0.05553635
## INFO  [08:03:07.489] [bbotk] Starting to optimize 5 parameter(s) with '<TunerGridSearch>' and '<TerminatorEvals> [n_evals=10, k=0]'
## INFO  [08:03:07.497] [bbotk] Evaluating 1 configuration(s)
## INFO  [08:03:07.515] [mlr3] Running benchmark with 10 resampling iterations
## INFO  [08:03:07.521] [mlr3] Applying learner 'regr.svm' on task 'PredictionSet[1:13]' (iter 1/10)
## INFO  [08:03:07.538] [mlr3] Applying learner 'regr.svm' on task 'PredictionSet[1:13]' (iter 2/10)
## INFO  [08:03:07.554] [mlr3] Applying learner 'regr.svm' on task 'PredictionSet[1:13]' (iter 3/10)
## INFO  [08:03:07.570] [mlr3] Applying learner 'regr.svm' on task 'PredictionSet[1:13]' (iter 4/10)
## INFO  [08:03:07.585] [mlr3] Applying learner 'regr.svm' on task 'PredictionSet[1:13]' (iter 5/10)
## INFO  [08:03:07.601] [mlr3] Applying learner 'regr.svm' on task 'PredictionSet[1:13]' (iter 6/10)
## INFO  [08:03:07.627] [mlr3] Applying learner 'regr.svm' on task 'PredictionSet[1:13]' (iter 7/10)
## INFO  [08:03:07.646] [mlr3] Applying learner 'regr.svm' on task 'PredictionSet[1:13]' (iter 8/10)
## INFO  [08:03:07.664] [mlr3] Applying learner 'regr.svm' on task 'PredictionSet[1:13]' (iter 9/10)
## INFO  [08:03:07.685] [mlr3] Applying learner 'regr.svm' on task 'PredictionSet[1:13]' (iter 10/10)
## INFO  [08:03:07.701] [mlr3] Finished benchmark
## INFO  [08:03:07.731] [bbotk] Result of batch 1:
## INFO  [08:03:07.733] [bbotk]  tolerance    gamma   epsilon           type kernel  regr.rmse warnings errors
## INFO  [08:03:07.733] [bbotk]  0.7073571 7.143714 0.7285714 eps-regression radial 0.05576525        0      0
## INFO  [08:03:07.733] [bbotk]  runtime_learners                                uhash
## INFO  [08:03:07.733] [bbotk]             0.071 82a997b3-1f67-43fe-bfe4-b2443ac561ea
## INFO  [08:03:07.734] [bbotk] Evaluating 1 configuration(s)
## INFO  [08:03:07.750] [mlr3] Running benchmark with 10 resampling iterations
## INFO  [08:03:07.756] [mlr3] Applying learner 'regr.svm' on task 'PredictionSet[1:13]' (iter 1/10)
## INFO  [08:03:07.772] [mlr3] Applying learner 'regr.svm' on task 'PredictionSet[1:13]' (iter 2/10)
## INFO  [08:03:07.787] [mlr3] Applying learner 'regr.svm' on task 'PredictionSet[1:13]' (iter 3/10)
## INFO  [08:03:07.802] [mlr3] Applying learner 'regr.svm' on task 'PredictionSet[1:13]' (iter 4/10)
## INFO  [08:03:07.817] [mlr3] Applying learner 'regr.svm' on task 'PredictionSet[1:13]' (iter 5/10)
## INFO  [08:03:07.833] [mlr3] Applying learner 'regr.svm' on task 'PredictionSet[1:13]' (iter 6/10)
## INFO  [08:03:07.849] [mlr3] Applying learner 'regr.svm' on task 'PredictionSet[1:13]' (iter 7/10)
## INFO  [08:03:07.865] [mlr3] Applying learner 'regr.svm' on task 'PredictionSet[1:13]' (iter 8/10)
## INFO  [08:03:07.880] [mlr3] Applying learner 'regr.svm' on task 'PredictionSet[1:13]' (iter 9/10)
## INFO  [08:03:07.895] [mlr3] Applying learner 'regr.svm' on task 'PredictionSet[1:13]' (iter 10/10)
## INFO  [08:03:07.911] [mlr3] Finished benchmark
## INFO  [08:03:07.954] [bbotk] Result of batch 2:
## INFO  [08:03:07.956] [bbotk]  tolerance   gamma   epsilon           type kernel  regr.rmse warnings errors
## INFO  [08:03:07.956] [bbotk]        0.9 25.0005 0.7857143 eps-regression radial 0.06106614        0      0
## INFO  [08:03:07.956] [bbotk]  runtime_learners                                uhash
## INFO  [08:03:07.956] [bbotk]             0.051 7962575e-a361-45e6-87ac-b2d7ddb68024
## INFO  [08:03:07.958] [bbotk] Evaluating 1 configuration(s)
## INFO  [08:03:07.979] [mlr3] Running benchmark with 10 resampling iterations
## INFO  [08:03:07.986] [mlr3] Applying learner 'regr.svm' on task 'PredictionSet[1:13]' (iter 1/10)
## INFO  [08:03:08.006] [mlr3] Applying learner 'regr.svm' on task 'PredictionSet[1:13]' (iter 2/10)
## INFO  [08:03:08.025] [mlr3] Applying learner 'regr.svm' on task 'PredictionSet[1:13]' (iter 3/10)
## INFO  [08:03:08.044] [mlr3] Applying learner 'regr.svm' on task 'PredictionSet[1:13]' (iter 4/10)
## INFO  [08:03:08.064] [mlr3] Applying learner 'regr.svm' on task 'PredictionSet[1:13]' (iter 5/10)
## INFO  [08:03:08.082] [mlr3] Applying learner 'regr.svm' on task 'PredictionSet[1:13]' (iter 6/10)
## INFO  [08:03:08.100] [mlr3] Applying learner 'regr.svm' on task 'PredictionSet[1:13]' (iter 7/10)
## INFO  [08:03:08.118] [mlr3] Applying learner 'regr.svm' on task 'PredictionSet[1:13]' (iter 8/10)
## INFO  [08:03:08.138] [mlr3] Applying learner 'regr.svm' on task 'PredictionSet[1:13]' (iter 9/10)
## INFO  [08:03:08.156] [mlr3] Applying learner 'regr.svm' on task 'PredictionSet[1:13]' (iter 10/10)
## INFO  [08:03:08.174] [mlr3] Finished benchmark
## INFO  [08:03:08.207] [bbotk] Result of batch 3:
## INFO  [08:03:08.209] [bbotk]  tolerance    gamma   epsilon           type  kernel regr.rmse warnings errors
## INFO  [08:03:08.209] [bbotk]  0.8357857 39.28593 0.4428571 eps-regression sigmoid  2.164307        0      0
## INFO  [08:03:08.209] [bbotk]  runtime_learners                                uhash
## INFO  [08:03:08.209] [bbotk]             0.081 2cc5b32c-60d4-487c-87f3-6311d9b515c6
## INFO  [08:03:08.211] [bbotk] Evaluating 1 configuration(s)
## INFO  [08:03:08.227] [mlr3] Running benchmark with 10 resampling iterations
## INFO  [08:03:08.232] [mlr3] Applying learner 'regr.svm' on task 'PredictionSet[1:13]' (iter 1/10)
## INFO  [08:03:08.249] [mlr3] Applying learner 'regr.svm' on task 'PredictionSet[1:13]' (iter 2/10)
## INFO  [08:03:08.266] [mlr3] Applying learner 'regr.svm' on task 'PredictionSet[1:13]' (iter 3/10)
## INFO  [08:03:08.295] [mlr3] Applying learner 'regr.svm' on task 'PredictionSet[1:13]' (iter 4/10)
## INFO  [08:03:08.316] [mlr3] Applying learner 'regr.svm' on task 'PredictionSet[1:13]' (iter 5/10)
## INFO  [08:03:08.337] [mlr3] Applying learner 'regr.svm' on task 'PredictionSet[1:13]' (iter 6/10)
## INFO  [08:03:08.357] [mlr3] Applying learner 'regr.svm' on task 'PredictionSet[1:13]' (iter 7/10)
## INFO  [08:03:08.376] [mlr3] Applying learner 'regr.svm' on task 'PredictionSet[1:13]' (iter 8/10)
## INFO  [08:03:08.394] [mlr3] Applying learner 'regr.svm' on task 'PredictionSet[1:13]' (iter 9/10)
## INFO  [08:03:08.411] [mlr3] Applying learner 'regr.svm' on task 'PredictionSet[1:13]' (iter 10/10)
## INFO  [08:03:08.428] [mlr3] Finished benchmark
## INFO  [08:03:08.460] [bbotk] Result of batch 4:
## INFO  [08:03:08.462] [bbotk]  tolerance   gamma   epsilon           type kernel  regr.rmse warnings errors
## INFO  [08:03:08.462] [bbotk]  0.1294286 25.0005 0.7285714 eps-regression radial 0.05230034        0      0
## INFO  [08:03:08.462] [bbotk]  runtime_learners                                uhash
## INFO  [08:03:08.462] [bbotk]             0.079 6d611e6b-adb5-45e7-83b6-3f1a0145bc54
## INFO  [08:03:08.464] [bbotk] Evaluating 1 configuration(s)
## INFO  [08:03:08.479] [mlr3] Running benchmark with 10 resampling iterations
## INFO  [08:03:08.484] [mlr3] Applying learner 'regr.svm' on task 'PredictionSet[1:13]' (iter 1/10)
## INFO  [08:03:08.502] [mlr3] Applying learner 'regr.svm' on task 'PredictionSet[1:13]' (iter 2/10)
## INFO  [08:03:08.522] [mlr3] Applying learner 'regr.svm' on task 'PredictionSet[1:13]' (iter 3/10)
## INFO  [08:03:08.540] [mlr3] Applying learner 'regr.svm' on task 'PredictionSet[1:13]' (iter 4/10)
## INFO  [08:03:08.558] [mlr3] Applying learner 'regr.svm' on task 'PredictionSet[1:13]' (iter 5/10)
## INFO  [08:03:08.577] [mlr3] Applying learner 'regr.svm' on task 'PredictionSet[1:13]' (iter 6/10)
## INFO  [08:03:08.596] [mlr3] Applying learner 'regr.svm' on task 'PredictionSet[1:13]' (iter 7/10)
## INFO  [08:03:08.614] [mlr3] Applying learner 'regr.svm' on task 'PredictionSet[1:13]' (iter 8/10)
## INFO  [08:03:08.642] [mlr3] Applying learner 'regr.svm' on task 'PredictionSet[1:13]' (iter 9/10)
## INFO  [08:03:08.666] [mlr3] Applying learner 'regr.svm' on task 'PredictionSet[1:13]' (iter 10/10)
## INFO  [08:03:08.687] [mlr3] Finished benchmark
## INFO  [08:03:08.722] [bbotk] Result of batch 5:
## INFO  [08:03:08.724] [bbotk]  tolerance    gamma   epsilon           type kernel  regr.rmse warnings errors
## INFO  [08:03:08.724] [bbotk]      0.001 14.28643 0.3285714 eps-regression radial 0.04039087        0      0
## INFO  [08:03:08.724] [bbotk]  runtime_learners                                uhash
## INFO  [08:03:08.724] [bbotk]             0.094 94495ff4-0f69-4ee1-bc2c-c3b1c01ea982
## INFO  [08:03:08.726] [bbotk] Evaluating 1 configuration(s)
## INFO  [08:03:08.743] [mlr3] Running benchmark with 10 resampling iterations
## INFO  [08:03:08.748] [mlr3] Applying learner 'regr.svm' on task 'PredictionSet[1:13]' (iter 1/10)
## INFO  [08:03:08.763] [mlr3] Applying learner 'regr.svm' on task 'PredictionSet[1:13]' (iter 2/10)
## INFO  [08:03:08.778] [mlr3] Applying learner 'regr.svm' on task 'PredictionSet[1:13]' (iter 3/10)
## INFO  [08:03:08.792] [mlr3] Applying learner 'regr.svm' on task 'PredictionSet[1:13]' (iter 4/10)
## INFO  [08:03:08.809] [mlr3] Applying learner 'regr.svm' on task 'PredictionSet[1:13]' (iter 5/10)
## INFO  [08:03:08.824] [mlr3] Applying learner 'regr.svm' on task 'PredictionSet[1:13]' (iter 6/10)
## INFO  [08:03:08.839] [mlr3] Applying learner 'regr.svm' on task 'PredictionSet[1:13]' (iter 7/10)
## INFO  [08:03:08.854] [mlr3] Applying learner 'regr.svm' on task 'PredictionSet[1:13]' (iter 8/10)
## INFO  [08:03:08.869] [mlr3] Applying learner 'regr.svm' on task 'PredictionSet[1:13]' (iter 9/10)
## INFO  [08:03:08.886] [mlr3] Applying learner 'regr.svm' on task 'PredictionSet[1:13]' (iter 10/10)
## INFO  [08:03:08.902] [mlr3] Finished benchmark
## INFO  [08:03:08.933] [bbotk] Result of batch 6:
## INFO  [08:03:08.935] [bbotk]  tolerance gamma epsilon           type kernel  regr.rmse warnings errors
## INFO  [08:03:08.935] [bbotk]  0.8357857    50     0.9 eps-regression radial 0.06199687        0      0
## INFO  [08:03:08.935] [bbotk]  runtime_learners                                uhash
## INFO  [08:03:08.935] [bbotk]             0.051 5028e75e-da61-4dda-b32f-463de821cc57
## INFO  [08:03:08.943] [bbotk] Evaluating 1 configuration(s)
## INFO  [08:03:08.966] [mlr3] Running benchmark with 10 resampling iterations
## INFO  [08:03:08.973] [mlr3] Applying learner 'regr.svm' on task 'PredictionSet[1:13]' (iter 1/10)
## INFO  [08:03:08.993] [mlr3] Applying learner 'regr.svm' on task 'PredictionSet[1:13]' (iter 2/10)
## INFO  [08:03:09.012] [mlr3] Applying learner 'regr.svm' on task 'PredictionSet[1:13]' (iter 3/10)
## INFO  [08:03:09.030] [mlr3] Applying learner 'regr.svm' on task 'PredictionSet[1:13]' (iter 4/10)
## INFO  [08:03:09.049] [mlr3] Applying learner 'regr.svm' on task 'PredictionSet[1:13]' (iter 5/10)
## INFO  [08:03:09.066] [mlr3] Applying learner 'regr.svm' on task 'PredictionSet[1:13]' (iter 6/10)
## INFO  [08:03:09.083] [mlr3] Applying learner 'regr.svm' on task 'PredictionSet[1:13]' (iter 7/10)
## INFO  [08:03:09.100] [mlr3] Applying learner 'regr.svm' on task 'PredictionSet[1:13]' (iter 8/10)
## INFO  [08:03:09.118] [mlr3] Applying learner 'regr.svm' on task 'PredictionSet[1:13]' (iter 9/10)
## INFO  [08:03:09.135] [mlr3] Applying learner 'regr.svm' on task 'PredictionSet[1:13]' (iter 10/10)
## INFO  [08:03:09.151] [mlr3] Finished benchmark
## INFO  [08:03:09.184] [bbotk] Result of batch 7:
## INFO  [08:03:09.186] [bbotk]  tolerance    gamma epsilon           type kernel  regr.rmse warnings errors
## INFO  [08:03:09.186] [bbotk]  0.5147143 28.57186     0.5 eps-regression radial 0.04901563        0      0
## INFO  [08:03:09.186] [bbotk]  runtime_learners                                uhash
## INFO  [08:03:09.186] [bbotk]              0.07 38b41229-4890-4012-b951-bebae191f06b
## INFO  [08:03:09.188] [bbotk] Evaluating 1 configuration(s)
## INFO  [08:03:09.204] [mlr3] Running benchmark with 10 resampling iterations
## INFO  [08:03:09.209] [mlr3] Applying learner 'regr.svm' on task 'PredictionSet[1:13]' (iter 1/10)
## INFO  [08:03:09.225] [mlr3] Applying learner 'regr.svm' on task 'PredictionSet[1:13]' (iter 2/10)
## INFO  [08:03:09.240] [mlr3] Applying learner 'regr.svm' on task 'PredictionSet[1:13]' (iter 3/10)
## INFO  [08:03:09.257] [mlr3] Applying learner 'regr.svm' on task 'PredictionSet[1:13]' (iter 4/10)
## INFO  [08:03:09.285] [mlr3] Applying learner 'regr.svm' on task 'PredictionSet[1:13]' (iter 5/10)
## INFO  [08:03:09.303] [mlr3] Applying learner 'regr.svm' on task 'PredictionSet[1:13]' (iter 6/10)
## INFO  [08:03:09.322] [mlr3] Applying learner 'regr.svm' on task 'PredictionSet[1:13]' (iter 7/10)
## INFO  [08:03:09.341] [mlr3] Applying learner 'regr.svm' on task 'PredictionSet[1:13]' (iter 8/10)
## INFO  [08:03:09.358] [mlr3] Applying learner 'regr.svm' on task 'PredictionSet[1:13]' (iter 9/10)
## INFO  [08:03:09.374] [mlr3] Applying learner 'regr.svm' on task 'PredictionSet[1:13]' (iter 10/10)
## INFO  [08:03:09.389] [mlr3] Finished benchmark
## INFO  [08:03:09.422] [bbotk] Result of batch 8:
## INFO  [08:03:09.423] [bbotk]  tolerance    gamma   epsilon           type kernel regr.rmse warnings errors
## INFO  [08:03:09.423] [bbotk]        0.9 10.71507 0.6142857 eps-regression radial 0.0552093        0      0
## INFO  [08:03:09.423] [bbotk]  runtime_learners                                uhash
## INFO  [08:03:09.423] [bbotk]             0.062 47678324-1ede-4443-918a-aee4c512a071
## INFO  [08:03:09.425] [bbotk] Evaluating 1 configuration(s)
## INFO  [08:03:09.441] [mlr3] Running benchmark with 10 resampling iterations
## INFO  [08:03:09.446] [mlr3] Applying learner 'regr.svm' on task 'PredictionSet[1:13]' (iter 1/10)
## INFO  [08:03:09.463] [mlr3] Applying learner 'regr.svm' on task 'PredictionSet[1:13]' (iter 2/10)
## INFO  [08:03:09.481] [mlr3] Applying learner 'regr.svm' on task 'PredictionSet[1:13]' (iter 3/10)
## INFO  [08:03:09.500] [mlr3] Applying learner 'regr.svm' on task 'PredictionSet[1:13]' (iter 4/10)
## INFO  [08:03:09.517] [mlr3] Applying learner 'regr.svm' on task 'PredictionSet[1:13]' (iter 5/10)
## INFO  [08:03:09.535] [mlr3] Applying learner 'regr.svm' on task 'PredictionSet[1:13]' (iter 6/10)
## INFO  [08:03:09.552] [mlr3] Applying learner 'regr.svm' on task 'PredictionSet[1:13]' (iter 7/10)
## INFO  [08:03:09.571] [mlr3] Applying learner 'regr.svm' on task 'PredictionSet[1:13]' (iter 8/10)
## INFO  [08:03:09.596] [mlr3] Applying learner 'regr.svm' on task 'PredictionSet[1:13]' (iter 9/10)
## INFO  [08:03:09.617] [mlr3] Applying learner 'regr.svm' on task 'PredictionSet[1:13]' (iter 10/10)
## INFO  [08:03:09.640] [mlr3] Finished benchmark
## INFO  [08:03:09.676] [bbotk] Result of batch 9:
## INFO  [08:03:09.678] [bbotk]  tolerance    gamma   epsilon           type kernel  regr.rmse warnings errors
## INFO  [08:03:09.678] [bbotk]  0.1294286 10.71507 0.2142857 eps-regression radial 0.03798496        0      0
## INFO  [08:03:09.678] [bbotk]  runtime_learners                                uhash
## INFO  [08:03:09.678] [bbotk]             0.074 40b6c6f1-0d30-4841-9ef8-77021397ae81
## INFO  [08:03:09.680] [bbotk] Evaluating 1 configuration(s)
## INFO  [08:03:09.696] [mlr3] Running benchmark with 10 resampling iterations
## INFO  [08:03:09.701] [mlr3] Applying learner 'regr.svm' on task 'PredictionSet[1:13]' (iter 1/10)
## INFO  [08:03:09.720] [mlr3] Applying learner 'regr.svm' on task 'PredictionSet[1:13]' (iter 2/10)
## INFO  [08:03:09.736] [mlr3] Applying learner 'regr.svm' on task 'PredictionSet[1:13]' (iter 3/10)
## INFO  [08:03:09.753] [mlr3] Applying learner 'regr.svm' on task 'PredictionSet[1:13]' (iter 4/10)
## INFO  [08:03:09.770] [mlr3] Applying learner 'regr.svm' on task 'PredictionSet[1:13]' (iter 5/10)
## INFO  [08:03:09.788] [mlr3] Applying learner 'regr.svm' on task 'PredictionSet[1:13]' (iter 6/10)
## INFO  [08:03:09.805] [mlr3] Applying learner 'regr.svm' on task 'PredictionSet[1:13]' (iter 7/10)
## INFO  [08:03:09.822] [mlr3] Applying learner 'regr.svm' on task 'PredictionSet[1:13]' (iter 8/10)
## INFO  [08:03:09.839] [mlr3] Applying learner 'regr.svm' on task 'PredictionSet[1:13]' (iter 9/10)
## INFO  [08:03:09.857] [mlr3] Applying learner 'regr.svm' on task 'PredictionSet[1:13]' (iter 10/10)
## INFO  [08:03:09.874] [mlr3] Finished benchmark
## INFO  [08:03:09.907] [bbotk] Result of batch 10:
## INFO  [08:03:09.909] [bbotk]  tolerance    gamma   epsilon           type kernel regr.rmse warnings errors
## INFO  [08:03:09.909] [bbotk]  0.2578571 17.85779 0.6142857 eps-regression radial 0.0486866        0      0
## INFO  [08:03:09.909] [bbotk]  runtime_learners                                uhash
## INFO  [08:03:09.909] [bbotk]             0.069 1ba29bb2-06cc-4d2a-afe8-cf1ff5bb60b6
## INFO  [08:03:09.921] [bbotk] Finished optimizing after 10 evaluation(s)
## INFO  [08:03:09.922] [bbotk] Result:
## INFO  [08:03:09.924] [bbotk]  tolerance    gamma   epsilon           type kernel learner_param_vals
## INFO  [08:03:09.924] [bbotk]  0.1294286 10.71507 0.2142857 eps-regression radial          <list[5]>
## INFO  [08:03:09.924] [bbotk]   x_domain  regr.rmse
## INFO  [08:03:09.924] [bbotk]  <list[5]> 0.03798496
## INFO  [08:03:10.079] [bbotk] Starting to optimize 3 parameter(s) with '<TunerGridSearch>' and '<TerminatorEvals> [n_evals=10, k=0]'
## INFO  [08:03:10.084] [bbotk] Evaluating 1 configuration(s)
## INFO  [08:03:10.098] [mlr3] Running benchmark with 10 resampling iterations
## INFO  [08:03:10.104] [mlr3] Applying learner 'regr.kknn' on task 'PredictionSet[1:13]' (iter 1/10)
## INFO  [08:03:10.124] [mlr3] Applying learner 'regr.kknn' on task 'PredictionSet[1:13]' (iter 2/10)
## INFO  [08:03:10.145] [mlr3] Applying learner 'regr.kknn' on task 'PredictionSet[1:13]' (iter 3/10)
## INFO  [08:03:10.167] [mlr3] Applying learner 'regr.kknn' on task 'PredictionSet[1:13]' (iter 4/10)
## INFO  [08:03:10.188] [mlr3] Applying learner 'regr.kknn' on task 'PredictionSet[1:13]' (iter 5/10)
## INFO  [08:03:10.217] [mlr3] Applying learner 'regr.kknn' on task 'PredictionSet[1:13]' (iter 6/10)
## INFO  [08:03:10.245] [mlr3] Applying learner 'regr.kknn' on task 'PredictionSet[1:13]' (iter 7/10)
## INFO  [08:03:10.270] [mlr3] Applying learner 'regr.kknn' on task 'PredictionSet[1:13]' (iter 8/10)
## INFO  [08:03:10.293] [mlr3] Applying learner 'regr.kknn' on task 'PredictionSet[1:13]' (iter 9/10)
## INFO  [08:03:10.316] [mlr3] Applying learner 'regr.kknn' on task 'PredictionSet[1:13]' (iter 10/10)
## INFO  [08:03:10.336] [mlr3] Finished benchmark
## INFO  [08:03:10.365] [bbotk] Result of batch 1:
## INFO  [08:03:10.367] [bbotk]   k distance      kernel  regr.rmse warnings errors runtime_learners
## INFO  [08:03:10.367] [bbotk]  12 18.57857 rectangular 0.02623506        0      0            0.112
## INFO  [08:03:10.367] [bbotk]                                 uhash
## INFO  [08:03:10.367] [bbotk]  36023f44-cac1-4b9e-8094-ba9ddf3f30ac
## INFO  [08:03:10.368] [bbotk] Evaluating 1 configuration(s)
## INFO  [08:03:10.379] [mlr3] Running benchmark with 10 resampling iterations
## INFO  [08:03:10.385] [mlr3] Applying learner 'regr.kknn' on task 'PredictionSet[1:13]' (iter 1/10)
## INFO  [08:03:10.406] [mlr3] Applying learner 'regr.kknn' on task 'PredictionSet[1:13]' (iter 2/10)
## INFO  [08:03:10.427] [mlr3] Applying learner 'regr.kknn' on task 'PredictionSet[1:13]' (iter 3/10)
## INFO  [08:03:10.447] [mlr3] Applying learner 'regr.kknn' on task 'PredictionSet[1:13]' (iter 4/10)
## INFO  [08:03:10.470] [mlr3] Applying learner 'regr.kknn' on task 'PredictionSet[1:13]' (iter 5/10)
## INFO  [08:03:10.492] [mlr3] Applying learner 'regr.kknn' on task 'PredictionSet[1:13]' (iter 6/10)
## INFO  [08:03:10.512] [mlr3] Applying learner 'regr.kknn' on task 'PredictionSet[1:13]' (iter 7/10)
## INFO  [08:03:10.534] [mlr3] Applying learner 'regr.kknn' on task 'PredictionSet[1:13]' (iter 8/10)
## INFO  [08:03:10.589] [mlr3] Applying learner 'regr.kknn' on task 'PredictionSet[1:13]' (iter 9/10)
## INFO  [08:03:10.620] [mlr3] Applying learner 'regr.kknn' on task 'PredictionSet[1:13]' (iter 10/10)
## INFO  [08:03:10.647] [mlr3] Finished benchmark
## INFO  [08:03:10.689] [bbotk] Result of batch 2:
## INFO  [08:03:10.691] [bbotk]   k distance   kernel regr.rmse warnings errors runtime_learners
## INFO  [08:03:10.691] [bbotk]  15 18.57857 biweight 0.0231981        0      0            0.144
## INFO  [08:03:10.691] [bbotk]                                 uhash
## INFO  [08:03:10.691] [bbotk]  bee92efd-9b53-437c-a948-e0aaca643d68
## INFO  [08:03:10.693] [bbotk] Evaluating 1 configuration(s)
## INFO  [08:03:10.705] [mlr3] Running benchmark with 10 resampling iterations
## INFO  [08:03:10.710] [mlr3] Applying learner 'regr.kknn' on task 'PredictionSet[1:13]' (iter 1/10)
## INFO  [08:03:10.731] [mlr3] Applying learner 'regr.kknn' on task 'PredictionSet[1:13]' (iter 2/10)
## INFO  [08:03:10.752] [mlr3] Applying learner 'regr.kknn' on task 'PredictionSet[1:13]' (iter 3/10)
## INFO  [08:03:10.775] [mlr3] Applying learner 'regr.kknn' on task 'PredictionSet[1:13]' (iter 4/10)
## INFO  [08:03:10.795] [mlr3] Applying learner 'regr.kknn' on task 'PredictionSet[1:13]' (iter 5/10)
## INFO  [08:03:10.816] [mlr3] Applying learner 'regr.kknn' on task 'PredictionSet[1:13]' (iter 6/10)
## INFO  [08:03:10.838] [mlr3] Applying learner 'regr.kknn' on task 'PredictionSet[1:13]' (iter 7/10)
## INFO  [08:03:10.859] [mlr3] Applying learner 'regr.kknn' on task 'PredictionSet[1:13]' (iter 8/10)
## INFO  [08:03:10.880] [mlr3] Applying learner 'regr.kknn' on task 'PredictionSet[1:13]' (iter 9/10)
## INFO  [08:03:10.901] [mlr3] Applying learner 'regr.kknn' on task 'PredictionSet[1:13]' (iter 10/10)
## INFO  [08:03:10.923] [mlr3] Finished benchmark
## INFO  [08:03:10.955] [bbotk] Result of batch 3:
## INFO  [08:03:10.957] [bbotk]  k distance kernel  regr.rmse warnings errors runtime_learners
## INFO  [08:03:10.957] [bbotk]  8 11.47143    inv 0.02104013        0      0            0.104
## INFO  [08:03:10.957] [bbotk]                                 uhash
## INFO  [08:03:10.957] [bbotk]  2d376f60-026e-472d-bb94-3b941493aa47
## INFO  [08:03:10.959] [bbotk] Evaluating 1 configuration(s)
## INFO  [08:03:10.978] [mlr3] Running benchmark with 10 resampling iterations
## INFO  [08:03:10.986] [mlr3] Applying learner 'regr.kknn' on task 'PredictionSet[1:13]' (iter 1/10)
## INFO  [08:03:11.011] [mlr3] Applying learner 'regr.kknn' on task 'PredictionSet[1:13]' (iter 2/10)
## INFO  [08:03:11.036] [mlr3] Applying learner 'regr.kknn' on task 'PredictionSet[1:13]' (iter 3/10)
## INFO  [08:03:11.063] [mlr3] Applying learner 'regr.kknn' on task 'PredictionSet[1:13]' (iter 4/10)
## INFO  [08:03:11.088] [mlr3] Applying learner 'regr.kknn' on task 'PredictionSet[1:13]' (iter 5/10)
## INFO  [08:03:11.109] [mlr3] Applying learner 'regr.kknn' on task 'PredictionSet[1:13]' (iter 6/10)
## INFO  [08:03:11.130] [mlr3] Applying learner 'regr.kknn' on task 'PredictionSet[1:13]' (iter 7/10)
## INFO  [08:03:11.153] [mlr3] Applying learner 'regr.kknn' on task 'PredictionSet[1:13]' (iter 8/10)
## INFO  [08:03:11.173] [mlr3] Applying learner 'regr.kknn' on task 'PredictionSet[1:13]' (iter 9/10)
## INFO  [08:03:11.194] [mlr3] Applying learner 'regr.kknn' on task 'PredictionSet[1:13]' (iter 10/10)
## INFO  [08:03:11.215] [mlr3] Finished benchmark
## INFO  [08:03:11.248] [bbotk] Result of batch 4:
## INFO  [08:03:11.249] [bbotk]  k distance kernel  regr.rmse warnings errors runtime_learners
## INFO  [08:03:11.249] [bbotk]  5 5.785714    cos 0.02024845        0      0            0.113
## INFO  [08:03:11.249] [bbotk]                                 uhash
## INFO  [08:03:11.249] [bbotk]  49dbca3e-5305-4372-9853-5f0315d35bf3
## INFO  [08:03:11.251] [bbotk] Evaluating 1 configuration(s)
## INFO  [08:03:11.262] [mlr3] Running benchmark with 10 resampling iterations
## INFO  [08:03:11.267] [mlr3] Applying learner 'regr.kknn' on task 'PredictionSet[1:13]' (iter 1/10)
## INFO  [08:03:11.289] [mlr3] Applying learner 'regr.kknn' on task 'PredictionSet[1:13]' (iter 2/10)
## INFO  [08:03:11.310] [mlr3] Applying learner 'regr.kknn' on task 'PredictionSet[1:13]' (iter 3/10)
## INFO  [08:03:11.331] [mlr3] Applying learner 'regr.kknn' on task 'PredictionSet[1:13]' (iter 4/10)
## INFO  [08:03:11.352] [mlr3] Applying learner 'regr.kknn' on task 'PredictionSet[1:13]' (iter 5/10)
## INFO  [08:03:11.387] [mlr3] Applying learner 'regr.kknn' on task 'PredictionSet[1:13]' (iter 6/10)
## INFO  [08:03:11.412] [mlr3] Applying learner 'regr.kknn' on task 'PredictionSet[1:13]' (iter 7/10)
## INFO  [08:03:11.439] [mlr3] Applying learner 'regr.kknn' on task 'PredictionSet[1:13]' (iter 8/10)
## INFO  [08:03:11.465] [mlr3] Applying learner 'regr.kknn' on task 'PredictionSet[1:13]' (iter 9/10)
## INFO  [08:03:11.489] [mlr3] Applying learner 'regr.kknn' on task 'PredictionSet[1:13]' (iter 10/10)
## INFO  [08:03:11.511] [mlr3] Finished benchmark
## INFO  [08:03:11.544] [bbotk] Result of batch 5:
## INFO  [08:03:11.546] [bbotk]  k distance   kernel  regr.rmse warnings errors runtime_learners
## INFO  [08:03:11.546] [bbotk]  8 11.47143 biweight 0.02097089        0      0            0.125
## INFO  [08:03:11.546] [bbotk]                                 uhash
## INFO  [08:03:11.546] [bbotk]  230934f4-7185-4917-91d0-ed1e45acf326
## INFO  [08:03:11.547] [bbotk] Evaluating 1 configuration(s)
## INFO  [08:03:11.558] [mlr3] Running benchmark with 10 resampling iterations
## INFO  [08:03:11.563] [mlr3] Applying learner 'regr.kknn' on task 'PredictionSet[1:13]' (iter 1/10)
## INFO  [08:03:11.584] [mlr3] Applying learner 'regr.kknn' on task 'PredictionSet[1:13]' (iter 2/10)
## INFO  [08:03:11.605] [mlr3] Applying learner 'regr.kknn' on task 'PredictionSet[1:13]' (iter 3/10)
## INFO  [08:03:11.626] [mlr3] Applying learner 'regr.kknn' on task 'PredictionSet[1:13]' (iter 4/10)
## INFO  [08:03:11.646] [mlr3] Applying learner 'regr.kknn' on task 'PredictionSet[1:13]' (iter 5/10)
## INFO  [08:03:11.669] [mlr3] Applying learner 'regr.kknn' on task 'PredictionSet[1:13]' (iter 6/10)
## INFO  [08:03:11.690] [mlr3] Applying learner 'regr.kknn' on task 'PredictionSet[1:13]' (iter 7/10)
## INFO  [08:03:11.711] [mlr3] Applying learner 'regr.kknn' on task 'PredictionSet[1:13]' (iter 8/10)
## INFO  [08:03:11.732] [mlr3] Applying learner 'regr.kknn' on task 'PredictionSet[1:13]' (iter 9/10)
## INFO  [08:03:11.762] [mlr3] Applying learner 'regr.kknn' on task 'PredictionSet[1:13]' (iter 10/10)
## INFO  [08:03:11.787] [mlr3] Finished benchmark
## INFO  [08:03:11.830] [bbotk] Result of batch 6:
## INFO  [08:03:11.832] [bbotk]  k distance       kernel  regr.rmse warnings errors runtime_learners
## INFO  [08:03:11.832] [bbotk]  9 14.31429 epanechnikov 0.02110186        0      0            0.106
## INFO  [08:03:11.832] [bbotk]                                 uhash
## INFO  [08:03:11.832] [bbotk]  67ab2846-07d6-4636-ba09-d8f774f1f80f
## INFO  [08:03:11.833] [bbotk] Evaluating 1 configuration(s)
## INFO  [08:03:11.847] [mlr3] Running benchmark with 10 resampling iterations
## INFO  [08:03:11.853] [mlr3] Applying learner 'regr.kknn' on task 'PredictionSet[1:13]' (iter 1/10)
## INFO  [08:03:11.875] [mlr3] Applying learner 'regr.kknn' on task 'PredictionSet[1:13]' (iter 2/10)
## INFO  [08:03:11.899] [mlr3] Applying learner 'regr.kknn' on task 'PredictionSet[1:13]' (iter 3/10)
## INFO  [08:03:11.920] [mlr3] Applying learner 'regr.kknn' on task 'PredictionSet[1:13]' (iter 4/10)
## INFO  [08:03:11.940] [mlr3] Applying learner 'regr.kknn' on task 'PredictionSet[1:13]' (iter 5/10)
## INFO  [08:03:11.962] [mlr3] Applying learner 'regr.kknn' on task 'PredictionSet[1:13]' (iter 6/10)
## INFO  [08:03:11.983] [mlr3] Applying learner 'regr.kknn' on task 'PredictionSet[1:13]' (iter 7/10)
## INFO  [08:03:12.004] [mlr3] Applying learner 'regr.kknn' on task 'PredictionSet[1:13]' (iter 8/10)
## INFO  [08:03:12.025] [mlr3] Applying learner 'regr.kknn' on task 'PredictionSet[1:13]' (iter 9/10)
## INFO  [08:03:12.048] [mlr3] Applying learner 'regr.kknn' on task 'PredictionSet[1:13]' (iter 10/10)
## INFO  [08:03:12.068] [mlr3] Finished benchmark
## INFO  [08:03:12.101] [bbotk] Result of batch 7:
## INFO  [08:03:12.103] [bbotk]   k distance  kernel  regr.rmse warnings errors runtime_learners
## INFO  [08:03:12.103] [bbotk]  10 7.207143 optimal 0.02226823        0      0            0.108
## INFO  [08:03:12.103] [bbotk]                                 uhash
## INFO  [08:03:12.103] [bbotk]  588cef39-285d-4dca-8e9e-6df48e81c832
## INFO  [08:03:12.104] [bbotk] Evaluating 1 configuration(s)
## INFO  [08:03:12.116] [mlr3] Running benchmark with 10 resampling iterations
## INFO  [08:03:12.121] [mlr3] Applying learner 'regr.kknn' on task 'PredictionSet[1:13]' (iter 1/10)
## INFO  [08:03:12.152] [mlr3] Applying learner 'regr.kknn' on task 'PredictionSet[1:13]' (iter 2/10)
## INFO  [08:03:12.177] [mlr3] Applying learner 'regr.kknn' on task 'PredictionSet[1:13]' (iter 3/10)
## INFO  [08:03:12.205] [mlr3] Applying learner 'regr.kknn' on task 'PredictionSet[1:13]' (iter 4/10)
## INFO  [08:03:12.230] [mlr3] Applying learner 'regr.kknn' on task 'PredictionSet[1:13]' (iter 5/10)
## INFO  [08:03:12.255] [mlr3] Applying learner 'regr.kknn' on task 'PredictionSet[1:13]' (iter 6/10)
## INFO  [08:03:12.278] [mlr3] Applying learner 'regr.kknn' on task 'PredictionSet[1:13]' (iter 7/10)
## INFO  [08:03:12.299] [mlr3] Applying learner 'regr.kknn' on task 'PredictionSet[1:13]' (iter 8/10)
## INFO  [08:03:12.320] [mlr3] Applying learner 'regr.kknn' on task 'PredictionSet[1:13]' (iter 9/10)
## INFO  [08:03:12.343] [mlr3] Applying learner 'regr.kknn' on task 'PredictionSet[1:13]' (iter 10/10)
## INFO  [08:03:12.363] [mlr3] Finished benchmark
## INFO  [08:03:12.395] [bbotk] Result of batch 8:
## INFO  [08:03:12.396] [bbotk]  k distance kernel  regr.rmse warnings errors runtime_learners
## INFO  [08:03:12.396] [bbotk]  8    10.05    cos 0.02111868        0      0            0.118
## INFO  [08:03:12.396] [bbotk]                                 uhash
## INFO  [08:03:12.396] [bbotk]  cf4c1232-f49a-41c4-820a-88b049a7cf81
## INFO  [08:03:12.398] [bbotk] Evaluating 1 configuration(s)
## INFO  [08:03:12.410] [mlr3] Running benchmark with 10 resampling iterations
## INFO  [08:03:12.416] [mlr3] Applying learner 'regr.kknn' on task 'PredictionSet[1:13]' (iter 1/10)
## INFO  [08:03:12.437] [mlr3] Applying learner 'regr.kknn' on task 'PredictionSet[1:13]' (iter 2/10)
## INFO  [08:03:12.458] [mlr3] Applying learner 'regr.kknn' on task 'PredictionSet[1:13]' (iter 3/10)
## INFO  [08:03:12.479] [mlr3] Applying learner 'regr.kknn' on task 'PredictionSet[1:13]' (iter 4/10)
## INFO  [08:03:12.503] [mlr3] Applying learner 'regr.kknn' on task 'PredictionSet[1:13]' (iter 5/10)
## INFO  [08:03:12.532] [mlr3] Applying learner 'regr.kknn' on task 'PredictionSet[1:13]' (iter 6/10)
## INFO  [08:03:12.560] [mlr3] Applying learner 'regr.kknn' on task 'PredictionSet[1:13]' (iter 7/10)
## INFO  [08:03:12.586] [mlr3] Applying learner 'regr.kknn' on task 'PredictionSet[1:13]' (iter 8/10)
## INFO  [08:03:12.612] [mlr3] Applying learner 'regr.kknn' on task 'PredictionSet[1:13]' (iter 9/10)
## INFO  [08:03:12.637] [mlr3] Applying learner 'regr.kknn' on task 'PredictionSet[1:13]' (iter 10/10)
## INFO  [08:03:12.660] [mlr3] Finished benchmark
## INFO  [08:03:12.693] [bbotk] Result of batch 9:
## INFO  [08:03:12.695] [bbotk]  k distance      kernel  regr.rmse warnings errors runtime_learners
## INFO  [08:03:12.695] [bbotk]  6 18.57857 rectangular 0.02543259        0      0            0.114
## INFO  [08:03:12.695] [bbotk]                                 uhash
## INFO  [08:03:12.695] [bbotk]  e4c30c91-6547-4663-8ea9-1e4f40e76154
## INFO  [08:03:12.696] [bbotk] Evaluating 1 configuration(s)
## INFO  [08:03:12.708] [mlr3] Running benchmark with 10 resampling iterations
## INFO  [08:03:12.714] [mlr3] Applying learner 'regr.kknn' on task 'PredictionSet[1:13]' (iter 1/10)
## INFO  [08:03:12.735] [mlr3] Applying learner 'regr.kknn' on task 'PredictionSet[1:13]' (iter 2/10)
## INFO  [08:03:12.755] [mlr3] Applying learner 'regr.kknn' on task 'PredictionSet[1:13]' (iter 3/10)
## INFO  [08:03:12.776] [mlr3] Applying learner 'regr.kknn' on task 'PredictionSet[1:13]' (iter 4/10)
## INFO  [08:03:12.798] [mlr3] Applying learner 'regr.kknn' on task 'PredictionSet[1:13]' (iter 5/10)
## INFO  [08:03:12.819] [mlr3] Applying learner 'regr.kknn' on task 'PredictionSet[1:13]' (iter 6/10)
## INFO  [08:03:12.840] [mlr3] Applying learner 'regr.kknn' on task 'PredictionSet[1:13]' (iter 7/10)
## INFO  [08:03:12.862] [mlr3] Applying learner 'regr.kknn' on task 'PredictionSet[1:13]' (iter 8/10)
## INFO  [08:03:12.883] [mlr3] Applying learner 'regr.kknn' on task 'PredictionSet[1:13]' (iter 9/10)
## INFO  [08:03:12.906] [mlr3] Applying learner 'regr.kknn' on task 'PredictionSet[1:13]' (iter 10/10)
## INFO  [08:03:12.940] [mlr3] Finished benchmark
## INFO  [08:03:12.981] [bbotk] Result of batch 10:
## INFO  [08:03:12.984] [bbotk]  k distance      kernel regr.rmse warnings errors runtime_learners
## INFO  [08:03:12.984] [bbotk]  2 11.47143 rectangular 0.0230524        0      0            0.107
## INFO  [08:03:12.984] [bbotk]                                 uhash
## INFO  [08:03:12.984] [bbotk]  9b375e20-911b-4e20-8317-e2d35f625520
## INFO  [08:03:12.989] [bbotk] Finished optimizing after 10 evaluation(s)
## INFO  [08:03:12.990] [bbotk] Result:
## INFO  [08:03:12.992] [bbotk]  k distance kernel learner_param_vals  x_domain  regr.rmse
## INFO  [08:03:12.992] [bbotk]  5 5.785714    cos          <list[3]> <list[3]> 0.02024845
## INFO  [08:03:13.226] [bbotk] Starting to optimize 2 parameter(s) with '<TunerGridSearch>' and '<TerminatorEvals> [n_evals=10, k=0]'
## INFO  [08:03:13.229] [bbotk] Evaluating 1 configuration(s)
## INFO  [08:03:13.261] [mlr3] Running benchmark with 10 resampling iterations
## INFO  [08:03:13.267] [mlr3] Applying learner 'regr.ranger' on task 'PredictionSet[1:13]' (iter 1/10)
## INFO  [08:03:13.305] [mlr3] Applying learner 'regr.ranger' on task 'PredictionSet[1:13]' (iter 2/10)
## INFO  [08:03:13.341] [mlr3] Applying learner 'regr.ranger' on task 'PredictionSet[1:13]' (iter 3/10)
## INFO  [08:03:13.374] [mlr3] Applying learner 'regr.ranger' on task 'PredictionSet[1:13]' (iter 4/10)
## INFO  [08:03:13.409] [mlr3] Applying learner 'regr.ranger' on task 'PredictionSet[1:13]' (iter 5/10)
## INFO  [08:03:13.442] [mlr3] Applying learner 'regr.ranger' on task 'PredictionSet[1:13]' (iter 6/10)
## INFO  [08:03:13.476] [mlr3] Applying learner 'regr.ranger' on task 'PredictionSet[1:13]' (iter 7/10)
## INFO  [08:03:13.510] [mlr3] Applying learner 'regr.ranger' on task 'PredictionSet[1:13]' (iter 8/10)
## INFO  [08:03:13.545] [mlr3] Applying learner 'regr.ranger' on task 'PredictionSet[1:13]' (iter 9/10)
## INFO  [08:03:13.578] [mlr3] Applying learner 'regr.ranger' on task 'PredictionSet[1:13]' (iter 10/10)
## INFO  [08:03:13.612] [mlr3] Finished benchmark
## INFO  [08:03:13.642] [bbotk] Result of batch 1:
## INFO  [08:03:13.644] [bbotk]  max.depth num.trees  regr.rmse warnings errors runtime_learners
## INFO  [08:03:13.644] [bbotk]         13       264 0.01701851        0      0            0.233
## INFO  [08:03:13.644] [bbotk]                                 uhash
## INFO  [08:03:13.644] [bbotk]  de7e3457-e3fe-4b74-9d1c-6b9a87d23e0f
## INFO  [08:03:13.645] [bbotk] Evaluating 1 configuration(s)
## INFO  [08:03:13.667] [mlr3] Running benchmark with 10 resampling iterations
## INFO  [08:03:13.673] [mlr3] Applying learner 'regr.ranger' on task 'PredictionSet[1:13]' (iter 1/10)
## INFO  [08:03:13.692] [mlr3] Applying learner 'regr.ranger' on task 'PredictionSet[1:13]' (iter 2/10)
## INFO  [08:03:13.719] [mlr3] Applying learner 'regr.ranger' on task 'PredictionSet[1:13]' (iter 3/10)
## INFO  [08:03:13.740] [mlr3] Applying learner 'regr.ranger' on task 'PredictionSet[1:13]' (iter 4/10)
## INFO  [08:03:13.762] [mlr3] Applying learner 'regr.ranger' on task 'PredictionSet[1:13]' (iter 5/10)
## INFO  [08:03:13.783] [mlr3] Applying learner 'regr.ranger' on task 'PredictionSet[1:13]' (iter 6/10)
## INFO  [08:03:13.803] [mlr3] Applying learner 'regr.ranger' on task 'PredictionSet[1:13]' (iter 7/10)
## INFO  [08:03:13.822] [mlr3] Applying learner 'regr.ranger' on task 'PredictionSet[1:13]' (iter 8/10)
## INFO  [08:03:13.842] [mlr3] Applying learner 'regr.ranger' on task 'PredictionSet[1:13]' (iter 9/10)
## INFO  [08:03:13.860] [mlr3] Applying learner 'regr.ranger' on task 'PredictionSet[1:13]' (iter 10/10)
## INFO  [08:03:13.876] [mlr3] Finished benchmark
## INFO  [08:03:13.909] [bbotk] Result of batch 2:
## INFO  [08:03:13.911] [bbotk]  max.depth num.trees  regr.rmse warnings errors runtime_learners
## INFO  [08:03:13.911] [bbotk]         16        27 0.01744056        0      0            0.075
## INFO  [08:03:13.911] [bbotk]                                 uhash
## INFO  [08:03:13.911] [bbotk]  78590542-6fed-47eb-8cea-96d04e0743a5
## INFO  [08:03:13.913] [bbotk] Evaluating 1 configuration(s)
## INFO  [08:03:13.935] [mlr3] Running benchmark with 10 resampling iterations
## INFO  [08:03:13.940] [mlr3] Applying learner 'regr.ranger' on task 'PredictionSet[1:13]' (iter 1/10)
## INFO  [08:03:13.955] [mlr3] Applying learner 'regr.ranger' on task 'PredictionSet[1:13]' (iter 2/10)
## INFO  [08:03:13.970] [mlr3] Applying learner 'regr.ranger' on task 'PredictionSet[1:13]' (iter 3/10)
## INFO  [08:03:13.987] [mlr3] Applying learner 'regr.ranger' on task 'PredictionSet[1:13]' (iter 4/10)
## INFO  [08:03:14.003] [mlr3] Applying learner 'regr.ranger' on task 'PredictionSet[1:13]' (iter 5/10)
## INFO  [08:03:14.018] [mlr3] Applying learner 'regr.ranger' on task 'PredictionSet[1:13]' (iter 6/10)
## INFO  [08:03:14.033] [mlr3] Applying learner 'regr.ranger' on task 'PredictionSet[1:13]' (iter 7/10)
## INFO  [08:03:14.057] [mlr3] Applying learner 'regr.ranger' on task 'PredictionSet[1:13]' (iter 8/10)
## INFO  [08:03:14.077] [mlr3] Applying learner 'regr.ranger' on task 'PredictionSet[1:13]' (iter 9/10)
## INFO  [08:03:14.096] [mlr3] Applying learner 'regr.ranger' on task 'PredictionSet[1:13]' (iter 10/10)
## INFO  [08:03:14.114] [mlr3] Finished benchmark
## INFO  [08:03:14.155] [bbotk] Result of batch 3:
## INFO  [08:03:14.157] [bbotk]  max.depth num.trees  regr.rmse warnings errors runtime_learners
## INFO  [08:03:14.157] [bbotk]          8         1 0.02803257        0      0            0.052
## INFO  [08:03:14.157] [bbotk]                                 uhash
## INFO  [08:03:14.157] [bbotk]  2320b616-c1c4-4114-9baf-18d4c0041940
## INFO  [08:03:14.158] [bbotk] Evaluating 1 configuration(s)
## INFO  [08:03:14.181] [mlr3] Running benchmark with 10 resampling iterations
## INFO  [08:03:14.186] [mlr3] Applying learner 'regr.ranger' on task 'PredictionSet[1:13]' (iter 1/10)
## INFO  [08:03:14.214] [mlr3] Applying learner 'regr.ranger' on task 'PredictionSet[1:13]' (iter 2/10)
## INFO  [08:03:14.240] [mlr3] Applying learner 'regr.ranger' on task 'PredictionSet[1:13]' (iter 3/10)
## INFO  [08:03:14.266] [mlr3] Applying learner 'regr.ranger' on task 'PredictionSet[1:13]' (iter 4/10)
## INFO  [08:03:14.293] [mlr3] Applying learner 'regr.ranger' on task 'PredictionSet[1:13]' (iter 5/10)
## INFO  [08:03:14.320] [mlr3] Applying learner 'regr.ranger' on task 'PredictionSet[1:13]' (iter 6/10)
## INFO  [08:03:14.345] [mlr3] Applying learner 'regr.ranger' on task 'PredictionSet[1:13]' (iter 7/10)
## INFO  [08:03:14.373] [mlr3] Applying learner 'regr.ranger' on task 'PredictionSet[1:13]' (iter 8/10)
## INFO  [08:03:14.399] [mlr3] Applying learner 'regr.ranger' on task 'PredictionSet[1:13]' (iter 9/10)
## INFO  [08:03:14.425] [mlr3] Applying learner 'regr.ranger' on task 'PredictionSet[1:13]' (iter 10/10)
## INFO  [08:03:14.452] [mlr3] Finished benchmark
## INFO  [08:03:14.493] [bbotk] Result of batch 4:
## INFO  [08:03:14.495] [bbotk]  max.depth num.trees  regr.rmse warnings errors runtime_learners
## INFO  [08:03:14.495] [bbotk]         16       158 0.01706609        0      0             0.16
## INFO  [08:03:14.495] [bbotk]                                 uhash
## INFO  [08:03:14.495] [bbotk]  634f693e-36a4-4cf1-bd14-69c97e278662
## INFO  [08:03:14.497] [bbotk] Evaluating 1 configuration(s)
## INFO  [08:03:14.529] [mlr3] Running benchmark with 10 resampling iterations
## INFO  [08:03:14.535] [mlr3] Applying learner 'regr.ranger' on task 'PredictionSet[1:13]' (iter 1/10)
## INFO  [08:03:14.569] [mlr3] Applying learner 'regr.ranger' on task 'PredictionSet[1:13]' (iter 2/10)
## INFO  [08:03:14.605] [mlr3] Applying learner 'regr.ranger' on task 'PredictionSet[1:13]' (iter 3/10)
## INFO  [08:03:14.636] [mlr3] Applying learner 'regr.ranger' on task 'PredictionSet[1:13]' (iter 4/10)
## INFO  [08:03:14.671] [mlr3] Applying learner 'regr.ranger' on task 'PredictionSet[1:13]' (iter 5/10)
## INFO  [08:03:14.703] [mlr3] Applying learner 'regr.ranger' on task 'PredictionSet[1:13]' (iter 6/10)
## INFO  [08:03:14.736] [mlr3] Applying learner 'regr.ranger' on task 'PredictionSet[1:13]' (iter 7/10)
## INFO  [08:03:14.768] [mlr3] Applying learner 'regr.ranger' on task 'PredictionSet[1:13]' (iter 8/10)
## INFO  [08:03:14.798] [mlr3] Applying learner 'regr.ranger' on task 'PredictionSet[1:13]' (iter 9/10)
## INFO  [08:03:14.830] [mlr3] Applying learner 'regr.ranger' on task 'PredictionSet[1:13]' (iter 10/10)
## INFO  [08:03:14.861] [mlr3] Finished benchmark
## INFO  [08:03:14.894] [bbotk] Result of batch 5:
## INFO  [08:03:14.895] [bbotk]  max.depth num.trees  regr.rmse warnings errors runtime_learners
## INFO  [08:03:14.895] [bbotk]          3       422 0.02478361        0      0            0.213
## INFO  [08:03:14.895] [bbotk]                                 uhash
## INFO  [08:03:14.895] [bbotk]  c9a5cd0b-67de-4b5c-9862-978d959c2121
## INFO  [08:03:14.897] [bbotk] Evaluating 1 configuration(s)
## INFO  [08:03:14.920] [mlr3] Running benchmark with 10 resampling iterations
## INFO  [08:03:14.925] [mlr3] Applying learner 'regr.ranger' on task 'PredictionSet[1:13]' (iter 1/10)
## INFO  [08:03:14.947] [mlr3] Applying learner 'regr.ranger' on task 'PredictionSet[1:13]' (iter 2/10)
## INFO  [08:03:14.980] [mlr3] Applying learner 'regr.ranger' on task 'PredictionSet[1:13]' (iter 3/10)
## INFO  [08:03:15.006] [mlr3] Applying learner 'regr.ranger' on task 'PredictionSet[1:13]' (iter 4/10)
## INFO  [08:03:15.034] [mlr3] Applying learner 'regr.ranger' on task 'PredictionSet[1:13]' (iter 5/10)
## INFO  [08:03:15.061] [mlr3] Applying learner 'regr.ranger' on task 'PredictionSet[1:13]' (iter 6/10)
## INFO  [08:03:15.087] [mlr3] Applying learner 'regr.ranger' on task 'PredictionSet[1:13]' (iter 7/10)
## INFO  [08:03:15.113] [mlr3] Applying learner 'regr.ranger' on task 'PredictionSet[1:13]' (iter 8/10)
## INFO  [08:03:15.137] [mlr3] Applying learner 'regr.ranger' on task 'PredictionSet[1:13]' (iter 9/10)
## INFO  [08:03:15.159] [mlr3] Applying learner 'regr.ranger' on task 'PredictionSet[1:13]' (iter 10/10)
## INFO  [08:03:15.182] [mlr3] Finished benchmark
## INFO  [08:03:15.214] [bbotk] Result of batch 6:
## INFO  [08:03:15.216] [bbotk]  max.depth num.trees  regr.rmse warnings errors runtime_learners
## INFO  [08:03:15.216] [bbotk]          0       106 0.01709126        0      0            0.139
## INFO  [08:03:15.216] [bbotk]                                 uhash
## INFO  [08:03:15.216] [bbotk]  4477fba6-b9d5-4f39-9e09-7798e9a20282
## INFO  [08:03:15.217] [bbotk] Evaluating 1 configuration(s)
## INFO  [08:03:15.239] [mlr3] Running benchmark with 10 resampling iterations
## INFO  [08:03:15.244] [mlr3] Applying learner 'regr.ranger' on task 'PredictionSet[1:13]' (iter 1/10)
## INFO  [08:03:15.264] [mlr3] Applying learner 'regr.ranger' on task 'PredictionSet[1:13]' (iter 2/10)
## INFO  [08:03:15.283] [mlr3] Applying learner 'regr.ranger' on task 'PredictionSet[1:13]' (iter 3/10)
## INFO  [08:03:15.301] [mlr3] Applying learner 'regr.ranger' on task 'PredictionSet[1:13]' (iter 4/10)
## INFO  [08:03:15.319] [mlr3] Applying learner 'regr.ranger' on task 'PredictionSet[1:13]' (iter 5/10)
## INFO  [08:03:15.340] [mlr3] Applying learner 'regr.ranger' on task 'PredictionSet[1:13]' (iter 6/10)
## INFO  [08:03:15.358] [mlr3] Applying learner 'regr.ranger' on task 'PredictionSet[1:13]' (iter 7/10)
## INFO  [08:03:15.386] [mlr3] Applying learner 'regr.ranger' on task 'PredictionSet[1:13]' (iter 8/10)
## INFO  [08:03:15.410] [mlr3] Applying learner 'regr.ranger' on task 'PredictionSet[1:13]' (iter 9/10)
## INFO  [08:03:15.433] [mlr3] Applying learner 'regr.ranger' on task 'PredictionSet[1:13]' (iter 10/10)
## INFO  [08:03:15.454] [mlr3] Finished benchmark
## INFO  [08:03:15.492] [bbotk] Result of batch 7:
## INFO  [08:03:15.494] [bbotk]  max.depth num.trees  regr.rmse warnings errors runtime_learners
## INFO  [08:03:15.494] [bbotk]          2       106 0.03019681        0      0            0.097
## INFO  [08:03:15.494] [bbotk]                                 uhash
## INFO  [08:03:15.494] [bbotk]  f00552a3-4a15-4aee-a25e-a2dff9c4c247
## INFO  [08:03:15.495] [bbotk] Evaluating 1 configuration(s)
## INFO  [08:03:15.518] [mlr3] Running benchmark with 10 resampling iterations
## INFO  [08:03:15.523] [mlr3] Applying learner 'regr.ranger' on task 'PredictionSet[1:13]' (iter 1/10)
## INFO  [08:03:15.562] [mlr3] Applying learner 'regr.ranger' on task 'PredictionSet[1:13]' (iter 2/10)
## INFO  [08:03:15.599] [mlr3] Applying learner 'regr.ranger' on task 'PredictionSet[1:13]' (iter 3/10)
## INFO  [08:03:15.637] [mlr3] Applying learner 'regr.ranger' on task 'PredictionSet[1:13]' (iter 4/10)
## INFO  [08:03:15.674] [mlr3] Applying learner 'regr.ranger' on task 'PredictionSet[1:13]' (iter 5/10)
## INFO  [08:03:15.713] [mlr3] Applying learner 'regr.ranger' on task 'PredictionSet[1:13]' (iter 6/10)
## INFO  [08:03:15.750] [mlr3] Applying learner 'regr.ranger' on task 'PredictionSet[1:13]' (iter 7/10)
## INFO  [08:03:15.789] [mlr3] Applying learner 'regr.ranger' on task 'PredictionSet[1:13]' (iter 8/10)
## INFO  [08:03:15.826] [mlr3] Applying learner 'regr.ranger' on task 'PredictionSet[1:13]' (iter 9/10)
## INFO  [08:03:15.865] [mlr3] Applying learner 'regr.ranger' on task 'PredictionSet[1:13]' (iter 10/10)
## INFO  [08:03:15.901] [mlr3] Finished benchmark
## INFO  [08:03:15.949] [bbotk] Result of batch 8:
## INFO  [08:03:15.951] [bbotk]  max.depth num.trees  regr.rmse warnings errors runtime_learners
## INFO  [08:03:15.951] [bbotk]         12       316 0.01719334        0      0            0.271
## INFO  [08:03:15.951] [bbotk]                                 uhash
## INFO  [08:03:15.951] [bbotk]  9dfdfc6e-ec02-4429-aecd-91889ce30ac1
## INFO  [08:03:15.953] [bbotk] Evaluating 1 configuration(s)
## INFO  [08:03:15.983] [mlr3] Running benchmark with 10 resampling iterations
## INFO  [08:03:15.989] [mlr3] Applying learner 'regr.ranger' on task 'PredictionSet[1:13]' (iter 1/10)
## INFO  [08:03:16.016] [mlr3] Applying learner 'regr.ranger' on task 'PredictionSet[1:13]' (iter 2/10)
## INFO  [08:03:16.039] [mlr3] Applying learner 'regr.ranger' on task 'PredictionSet[1:13]' (iter 3/10)
## INFO  [08:03:16.062] [mlr3] Applying learner 'regr.ranger' on task 'PredictionSet[1:13]' (iter 4/10)
## INFO  [08:03:16.086] [mlr3] Applying learner 'regr.ranger' on task 'PredictionSet[1:13]' (iter 5/10)
## INFO  [08:03:16.109] [mlr3] Applying learner 'regr.ranger' on task 'PredictionSet[1:13]' (iter 6/10)
## INFO  [08:03:16.131] [mlr3] Applying learner 'regr.ranger' on task 'PredictionSet[1:13]' (iter 7/10)
## INFO  [08:03:16.154] [mlr3] Applying learner 'regr.ranger' on task 'PredictionSet[1:13]' (iter 8/10)
## INFO  [08:03:16.177] [mlr3] Applying learner 'regr.ranger' on task 'PredictionSet[1:13]' (iter 9/10)
## INFO  [08:03:16.199] [mlr3] Applying learner 'regr.ranger' on task 'PredictionSet[1:13]' (iter 10/10)
## INFO  [08:03:16.221] [mlr3] Finished benchmark
## INFO  [08:03:16.255] [bbotk] Result of batch 9:
## INFO  [08:03:16.257] [bbotk]  max.depth num.trees  regr.rmse warnings errors runtime_learners
## INFO  [08:03:16.257] [bbotk]          4       158 0.02149864        0      0            0.126
## INFO  [08:03:16.257] [bbotk]                                 uhash
## INFO  [08:03:16.257] [bbotk]  6231c546-4da8-46d9-a470-0084e973b8a5
## INFO  [08:03:16.258] [bbotk] Evaluating 1 configuration(s)
## INFO  [08:03:16.280] [mlr3] Running benchmark with 10 resampling iterations
## INFO  [08:03:16.286] [mlr3] Applying learner 'regr.ranger' on task 'PredictionSet[1:13]' (iter 1/10)
## INFO  [08:03:16.314] [mlr3] Applying learner 'regr.ranger' on task 'PredictionSet[1:13]' (iter 2/10)
## INFO  [08:03:16.333] [mlr3] Applying learner 'regr.ranger' on task 'PredictionSet[1:13]' (iter 3/10)
## INFO  [08:03:16.352] [mlr3] Applying learner 'regr.ranger' on task 'PredictionSet[1:13]' (iter 4/10)
## INFO  [08:03:16.370] [mlr3] Applying learner 'regr.ranger' on task 'PredictionSet[1:13]' (iter 5/10)
## INFO  [08:03:16.391] [mlr3] Applying learner 'regr.ranger' on task 'PredictionSet[1:13]' (iter 6/10)
## INFO  [08:03:16.407] [mlr3] Applying learner 'regr.ranger' on task 'PredictionSet[1:13]' (iter 7/10)
## INFO  [08:03:16.423] [mlr3] Applying learner 'regr.ranger' on task 'PredictionSet[1:13]' (iter 8/10)
## INFO  [08:03:16.439] [mlr3] Applying learner 'regr.ranger' on task 'PredictionSet[1:13]' (iter 9/10)
## INFO  [08:03:16.455] [mlr3] Applying learner 'regr.ranger' on task 'PredictionSet[1:13]' (iter 10/10)
## INFO  [08:03:16.471] [mlr3] Finished benchmark
## INFO  [08:03:16.503] [bbotk] Result of batch 10:
## INFO  [08:03:16.505] [bbotk]  max.depth num.trees  regr.rmse warnings errors runtime_learners
## INFO  [08:03:16.505] [bbotk]         12         1 0.03017326        0      0            0.066
## INFO  [08:03:16.505] [bbotk]                                 uhash
## INFO  [08:03:16.505] [bbotk]  b40b4936-a6e8-4e4f-a2cb-fc84d61cfff1
## INFO  [08:03:16.510] [bbotk] Finished optimizing after 10 evaluation(s)
## INFO  [08:03:16.510] [bbotk] Result:
## INFO  [08:03:16.511] [bbotk]  max.depth num.trees learner_param_vals  x_domain  regr.rmse
## INFO  [08:03:16.511] [bbotk]         13       264          <list[3]> <list[2]> 0.01701851
## INFO  [08:03:16.773] [bbotk] Starting to optimize 5 parameter(s) with '<TunerGridSearch>' and '<TerminatorEvals> [n_evals=10, k=0]'
## INFO  [08:03:16.781] [bbotk] Evaluating 1 configuration(s)
## INFO  [08:03:16.799] [mlr3] Running benchmark with 10 resampling iterations
## INFO  [08:03:16.804] [mlr3] Applying learner 'regr.svm' on task 'PredictionSet[1:13]' (iter 1/10)
## INFO  [08:03:16.823] [mlr3] Applying learner 'regr.svm' on task 'PredictionSet[1:13]' (iter 2/10)
## INFO  [08:03:16.843] [mlr3] Applying learner 'regr.svm' on task 'PredictionSet[1:13]' (iter 3/10)
## INFO  [08:03:16.862] [mlr3] Applying learner 'regr.svm' on task 'PredictionSet[1:13]' (iter 4/10)
## INFO  [08:03:16.881] [mlr3] Applying learner 'regr.svm' on task 'PredictionSet[1:13]' (iter 5/10)
## INFO  [08:03:16.900] [mlr3] Applying learner 'regr.svm' on task 'PredictionSet[1:13]' (iter 6/10)
## INFO  [08:03:16.951] [mlr3] Applying learner 'regr.svm' on task 'PredictionSet[1:13]' (iter 7/10)
## INFO  [08:03:16.978] [mlr3] Applying learner 'regr.svm' on task 'PredictionSet[1:13]' (iter 8/10)
## INFO  [08:03:17.004] [mlr3] Applying learner 'regr.svm' on task 'PredictionSet[1:13]' (iter 9/10)
## INFO  [08:03:17.027] [mlr3] Applying learner 'regr.svm' on task 'PredictionSet[1:13]' (iter 10/10)
## INFO  [08:03:17.050] [mlr3] Finished benchmark
## INFO  [08:03:17.087] [bbotk] Result of batch 1:
## INFO  [08:03:17.089] [bbotk]  tolerance    gamma   epsilon           type  kernel regr.rmse warnings errors
## INFO  [08:03:17.089] [bbotk]  0.7073571 14.28643 0.6714286 eps-regression sigmoid 0.5914616        0      0
## INFO  [08:03:17.089] [bbotk]  runtime_learners                                uhash
## INFO  [08:03:17.089] [bbotk]             0.102 37f32cbb-8c40-4675-a698-ee138a81d692
## INFO  [08:03:17.091] [bbotk] Evaluating 1 configuration(s)
## INFO  [08:03:17.107] [mlr3] Running benchmark with 10 resampling iterations
## INFO  [08:03:17.112] [mlr3] Applying learner 'regr.svm' on task 'PredictionSet[1:13]' (iter 1/10)
## INFO  [08:03:17.133] [mlr3] Applying learner 'regr.svm' on task 'PredictionSet[1:13]' (iter 2/10)
## INFO  [08:03:17.153] [mlr3] Applying learner 'regr.svm' on task 'PredictionSet[1:13]' (iter 3/10)
## INFO  [08:03:17.172] [mlr3] Applying learner 'regr.svm' on task 'PredictionSet[1:13]' (iter 4/10)
## INFO  [08:03:17.191] [mlr3] Applying learner 'regr.svm' on task 'PredictionSet[1:13]' (iter 5/10)
## INFO  [08:03:17.212] [mlr3] Applying learner 'regr.svm' on task 'PredictionSet[1:13]' (iter 6/10)
## INFO  [08:03:17.231] [mlr3] Applying learner 'regr.svm' on task 'PredictionSet[1:13]' (iter 7/10)
## INFO  [08:03:17.250] [mlr3] Applying learner 'regr.svm' on task 'PredictionSet[1:13]' (iter 8/10)
## INFO  [08:03:17.269] [mlr3] Applying learner 'regr.svm' on task 'PredictionSet[1:13]' (iter 9/10)
## INFO  [08:03:17.290] [mlr3] Applying learner 'regr.svm' on task 'PredictionSet[1:13]' (iter 10/10)
## INFO  [08:03:17.309] [mlr3] Finished benchmark
## INFO  [08:03:17.341] [bbotk] Result of batch 2:
## INFO  [08:03:17.351] [bbotk]  tolerance    gamma   epsilon           type  kernel regr.rmse warnings errors
## INFO  [08:03:17.351] [bbotk]  0.1294286 10.71507 0.6714286 eps-regression sigmoid 0.6061586        0      0
## INFO  [08:03:17.351] [bbotk]  runtime_learners                                uhash
## INFO  [08:03:17.351] [bbotk]             0.092 7eb9b5c7-a82e-4d71-b006-b9790d57b191
## INFO  [08:03:17.353] [bbotk] Evaluating 1 configuration(s)
## INFO  [08:03:17.376] [mlr3] Running benchmark with 10 resampling iterations
## INFO  [08:03:17.383] [mlr3] Applying learner 'regr.svm' on task 'PredictionSet[1:13]' (iter 1/10)
## INFO  [08:03:17.403] [mlr3] Applying learner 'regr.svm' on task 'PredictionSet[1:13]' (iter 2/10)
## INFO  [08:03:17.424] [mlr3] Applying learner 'regr.svm' on task 'PredictionSet[1:13]' (iter 3/10)
## INFO  [08:03:17.447] [mlr3] Applying learner 'regr.svm' on task 'PredictionSet[1:13]' (iter 4/10)
## INFO  [08:03:17.467] [mlr3] Applying learner 'regr.svm' on task 'PredictionSet[1:13]' (iter 5/10)
## INFO  [08:03:17.484] [mlr3] Applying learner 'regr.svm' on task 'PredictionSet[1:13]' (iter 6/10)
## INFO  [08:03:17.502] [mlr3] Applying learner 'regr.svm' on task 'PredictionSet[1:13]' (iter 7/10)
## INFO  [08:03:17.522] [mlr3] Applying learner 'regr.svm' on task 'PredictionSet[1:13]' (iter 8/10)
## INFO  [08:03:17.539] [mlr3] Applying learner 'regr.svm' on task 'PredictionSet[1:13]' (iter 9/10)
## INFO  [08:03:17.556] [mlr3] Applying learner 'regr.svm' on task 'PredictionSet[1:13]' (iter 10/10)
## INFO  [08:03:17.572] [mlr3] Finished benchmark
## INFO  [08:03:17.605] [bbotk] Result of batch 3:
## INFO  [08:03:17.607] [bbotk]  tolerance    gamma   epsilon           type kernel  regr.rmse warnings errors
## INFO  [08:03:17.607] [bbotk]      0.001 35.71457 0.5571429 eps-regression radial 0.01492091        0      0
## INFO  [08:03:17.607] [bbotk]  runtime_learners                                uhash
## INFO  [08:03:17.607] [bbotk]             0.076 e5e18fbd-ee8b-448e-83cb-6d3211383a61
## INFO  [08:03:17.608] [bbotk] Evaluating 1 configuration(s)
## INFO  [08:03:17.625] [mlr3] Running benchmark with 10 resampling iterations
## INFO  [08:03:17.630] [mlr3] Applying learner 'regr.svm' on task 'PredictionSet[1:13]' (iter 1/10)
## INFO  [08:03:17.648] [mlr3] Applying learner 'regr.svm' on task 'PredictionSet[1:13]' (iter 2/10)
## INFO  [08:03:17.668] [mlr3] Applying learner 'regr.svm' on task 'PredictionSet[1:13]' (iter 3/10)
## INFO  [08:03:17.687] [mlr3] Applying learner 'regr.svm' on task 'PredictionSet[1:13]' (iter 4/10)
## INFO  [08:03:17.705] [mlr3] Applying learner 'regr.svm' on task 'PredictionSet[1:13]' (iter 5/10)
## INFO  [08:03:17.734] [mlr3] Applying learner 'regr.svm' on task 'PredictionSet[1:13]' (iter 6/10)
## INFO  [08:03:17.758] [mlr3] Applying learner 'regr.svm' on task 'PredictionSet[1:13]' (iter 7/10)
## INFO  [08:03:17.780] [mlr3] Applying learner 'regr.svm' on task 'PredictionSet[1:13]' (iter 8/10)
## INFO  [08:03:17.802] [mlr3] Applying learner 'regr.svm' on task 'PredictionSet[1:13]' (iter 9/10)
## INFO  [08:03:17.826] [mlr3] Applying learner 'regr.svm' on task 'PredictionSet[1:13]' (iter 10/10)
## INFO  [08:03:17.847] [mlr3] Finished benchmark
## INFO  [08:03:17.882] [bbotk] Result of batch 4:
## INFO  [08:03:17.884] [bbotk]  tolerance    gamma   epsilon           type  kernel regr.rmse warnings errors
## INFO  [08:03:17.884] [bbotk]  0.3220714 46.42864 0.7285714 eps-regression sigmoid 0.6069505        0      0
## INFO  [08:03:17.884] [bbotk]  runtime_learners                                uhash
## INFO  [08:03:17.884] [bbotk]             0.096 0060f93c-2ebc-456b-8e0b-ce6bd710acd8
## INFO  [08:03:17.886] [bbotk] Evaluating 1 configuration(s)
## INFO  [08:03:17.903] [mlr3] Running benchmark with 10 resampling iterations
## INFO  [08:03:17.908] [mlr3] Applying learner 'regr.svm' on task 'PredictionSet[1:13]' (iter 1/10)
## INFO  [08:03:17.923] [mlr3] Applying learner 'regr.svm' on task 'PredictionSet[1:13]' (iter 2/10)
## INFO  [08:03:17.939] [mlr3] Applying learner 'regr.svm' on task 'PredictionSet[1:13]' (iter 3/10)
## INFO  [08:03:17.955] [mlr3] Applying learner 'regr.svm' on task 'PredictionSet[1:13]' (iter 4/10)
## INFO  [08:03:17.972] [mlr3] Applying learner 'regr.svm' on task 'PredictionSet[1:13]' (iter 5/10)
## INFO  [08:03:17.988] [mlr3] Applying learner 'regr.svm' on task 'PredictionSet[1:13]' (iter 6/10)
## INFO  [08:03:18.004] [mlr3] Applying learner 'regr.svm' on task 'PredictionSet[1:13]' (iter 7/10)
## INFO  [08:03:18.019] [mlr3] Applying learner 'regr.svm' on task 'PredictionSet[1:13]' (iter 8/10)
## INFO  [08:03:18.037] [mlr3] Applying learner 'regr.svm' on task 'PredictionSet[1:13]' (iter 9/10)
## INFO  [08:03:18.053] [mlr3] Applying learner 'regr.svm' on task 'PredictionSet[1:13]' (iter 10/10)
## INFO  [08:03:18.068] [mlr3] Finished benchmark
## INFO  [08:03:18.116] [bbotk] Result of batch 5:
## INFO  [08:03:18.118] [bbotk]  tolerance    gamma   epsilon           type kernel  regr.rmse warnings errors
## INFO  [08:03:18.118] [bbotk]  0.6431429 28.57186 0.7857143 eps-regression radial 0.01795567        0      0
## INFO  [08:03:18.118] [bbotk]  runtime_learners                                uhash
## INFO  [08:03:18.118] [bbotk]             0.056 2ada17fb-5572-4171-96d5-441c641e0599
## INFO  [08:03:18.120] [bbotk] Evaluating 1 configuration(s)
## INFO  [08:03:18.141] [mlr3] Running benchmark with 10 resampling iterations
## INFO  [08:03:18.148] [mlr3] Applying learner 'regr.svm' on task 'PredictionSet[1:13]' (iter 1/10)
## INFO  [08:03:18.171] [mlr3] Applying learner 'regr.svm' on task 'PredictionSet[1:13]' (iter 2/10)
## INFO  [08:03:18.196] [mlr3] Applying learner 'regr.svm' on task 'PredictionSet[1:13]' (iter 3/10)
## INFO  [08:03:18.217] [mlr3] Applying learner 'regr.svm' on task 'PredictionSet[1:13]' (iter 4/10)
## INFO  [08:03:18.237] [mlr3] Applying learner 'regr.svm' on task 'PredictionSet[1:13]' (iter 5/10)
## INFO  [08:03:18.258] [mlr3] Applying learner 'regr.svm' on task 'PredictionSet[1:13]' (iter 6/10)
## INFO  [08:03:18.279] [mlr3] Applying learner 'regr.svm' on task 'PredictionSet[1:13]' (iter 7/10)
## INFO  [08:03:18.298] [mlr3] Applying learner 'regr.svm' on task 'PredictionSet[1:13]' (iter 8/10)
## INFO  [08:03:18.318] [mlr3] Applying learner 'regr.svm' on task 'PredictionSet[1:13]' (iter 9/10)
## INFO  [08:03:18.339] [mlr3] Applying learner 'regr.svm' on task 'PredictionSet[1:13]' (iter 10/10)
## INFO  [08:03:18.359] [mlr3] Finished benchmark
## INFO  [08:03:18.390] [bbotk] Result of batch 6:
## INFO  [08:03:18.392] [bbotk]  tolerance    gamma   epsilon           type  kernel regr.rmse warnings errors
## INFO  [08:03:18.392] [bbotk]      0.001 7.143714 0.1571429 eps-regression sigmoid 0.6026232        0      0
## INFO  [08:03:18.392] [bbotk]  runtime_learners                                uhash
## INFO  [08:03:18.392] [bbotk]             0.099 457cb66d-136c-44b0-9dfe-6576d823eb79
## INFO  [08:03:18.394] [bbotk] Evaluating 1 configuration(s)
## INFO  [08:03:18.412] [mlr3] Running benchmark with 10 resampling iterations
## INFO  [08:03:18.417] [mlr3] Applying learner 'regr.svm' on task 'PredictionSet[1:13]' (iter 1/10)
## INFO  [08:03:18.436] [mlr3] Applying learner 'regr.svm' on task 'PredictionSet[1:13]' (iter 2/10)
## INFO  [08:03:18.454] [mlr3] Applying learner 'regr.svm' on task 'PredictionSet[1:13]' (iter 3/10)
## INFO  [08:03:18.484] [mlr3] Applying learner 'regr.svm' on task 'PredictionSet[1:13]' (iter 4/10)
## INFO  [08:03:18.508] [mlr3] Applying learner 'regr.svm' on task 'PredictionSet[1:13]' (iter 5/10)
## INFO  [08:03:18.530] [mlr3] Applying learner 'regr.svm' on task 'PredictionSet[1:13]' (iter 6/10)
## INFO  [08:03:18.552] [mlr3] Applying learner 'regr.svm' on task 'PredictionSet[1:13]' (iter 7/10)
## INFO  [08:03:18.576] [mlr3] Applying learner 'regr.svm' on task 'PredictionSet[1:13]' (iter 8/10)
## INFO  [08:03:18.598] [mlr3] Applying learner 'regr.svm' on task 'PredictionSet[1:13]' (iter 9/10)
## INFO  [08:03:18.617] [mlr3] Applying learner 'regr.svm' on task 'PredictionSet[1:13]' (iter 10/10)
## INFO  [08:03:18.638] [mlr3] Finished benchmark
## INFO  [08:03:18.671] [bbotk] Result of batch 7:
## INFO  [08:03:18.672] [bbotk]  tolerance    gamma epsilon           type  kernel regr.rmse warnings errors
## INFO  [08:03:18.672] [bbotk]  0.1936429 28.57186     0.5 eps-regression sigmoid 0.6078612        0      0
## INFO  [08:03:18.672] [bbotk]  runtime_learners                                uhash
## INFO  [08:03:18.672] [bbotk]               0.1 1c5c51ce-66fc-4bd3-96a6-7f3dcb812bab
## INFO  [08:03:18.674] [bbotk] Evaluating 1 configuration(s)
## INFO  [08:03:18.689] [mlr3] Running benchmark with 10 resampling iterations
## INFO  [08:03:18.694] [mlr3] Applying learner 'regr.svm' on task 'PredictionSet[1:13]' (iter 1/10)
## INFO  [08:03:18.715] [mlr3] Applying learner 'regr.svm' on task 'PredictionSet[1:13]' (iter 2/10)
## INFO  [08:03:18.733] [mlr3] Applying learner 'regr.svm' on task 'PredictionSet[1:13]' (iter 3/10)
## INFO  [08:03:18.752] [mlr3] Applying learner 'regr.svm' on task 'PredictionSet[1:13]' (iter 4/10)
## INFO  [08:03:18.770] [mlr3] Applying learner 'regr.svm' on task 'PredictionSet[1:13]' (iter 5/10)
## INFO  [08:03:18.790] [mlr3] Applying learner 'regr.svm' on task 'PredictionSet[1:13]' (iter 6/10)
## INFO  [08:03:18.809] [mlr3] Applying learner 'regr.svm' on task 'PredictionSet[1:13]' (iter 7/10)
## INFO  [08:03:18.827] [mlr3] Applying learner 'regr.svm' on task 'PredictionSet[1:13]' (iter 8/10)
## INFO  [08:03:18.854] [mlr3] Applying learner 'regr.svm' on task 'PredictionSet[1:13]' (iter 9/10)
## INFO  [08:03:18.878] [mlr3] Applying learner 'regr.svm' on task 'PredictionSet[1:13]' (iter 10/10)
## INFO  [08:03:18.901] [mlr3] Finished benchmark
## INFO  [08:03:18.943] [bbotk] Result of batch 8:
## INFO  [08:03:18.945] [bbotk]  tolerance    gamma   epsilon           type  kernel regr.rmse warnings errors
## INFO  [08:03:18.945] [bbotk]  0.7073571 42.85729 0.4428571 eps-regression sigmoid 0.6086582        0      0
## INFO  [08:03:18.945] [bbotk]  runtime_learners                                uhash
## INFO  [08:03:18.945] [bbotk]             0.095 40982200-6e82-498f-a4ae-0182df788ef3
## INFO  [08:03:18.947] [bbotk] Evaluating 1 configuration(s)
## INFO  [08:03:18.967] [mlr3] Running benchmark with 10 resampling iterations
## INFO  [08:03:18.972] [mlr3] Applying learner 'regr.svm' on task 'PredictionSet[1:13]' (iter 1/10)
## INFO  [08:03:18.991] [mlr3] Applying learner 'regr.svm' on task 'PredictionSet[1:13]' (iter 2/10)
## INFO  [08:03:19.013] [mlr3] Applying learner 'regr.svm' on task 'PredictionSet[1:13]' (iter 3/10)
## INFO  [08:03:19.032] [mlr3] Applying learner 'regr.svm' on task 'PredictionSet[1:13]' (iter 4/10)
## INFO  [08:03:19.051] [mlr3] Applying learner 'regr.svm' on task 'PredictionSet[1:13]' (iter 5/10)
## INFO  [08:03:19.069] [mlr3] Applying learner 'regr.svm' on task 'PredictionSet[1:13]' (iter 6/10)
## INFO  [08:03:19.090] [mlr3] Applying learner 'regr.svm' on task 'PredictionSet[1:13]' (iter 7/10)
## INFO  [08:03:19.108] [mlr3] Applying learner 'regr.svm' on task 'PredictionSet[1:13]' (iter 8/10)
## INFO  [08:03:19.127] [mlr3] Applying learner 'regr.svm' on task 'PredictionSet[1:13]' (iter 9/10)
## INFO  [08:03:19.146] [mlr3] Applying learner 'regr.svm' on task 'PredictionSet[1:13]' (iter 10/10)
## INFO  [08:03:19.166] [mlr3] Finished benchmark
## INFO  [08:03:19.198] [bbotk] Result of batch 9:
## INFO  [08:03:19.200] [bbotk]  tolerance    gamma   epsilon           type  kernel regr.rmse warnings errors
## INFO  [08:03:19.200] [bbotk]  0.8357857 17.85779 0.5571429 eps-regression sigmoid 0.6033047        0      0
## INFO  [08:03:19.200] [bbotk]  runtime_learners                                uhash
## INFO  [08:03:19.200] [bbotk]              0.09 df1c1896-8289-4ad6-aa1c-c4ae6aadf25b
## INFO  [08:03:19.201] [bbotk] Evaluating 1 configuration(s)
## INFO  [08:03:19.445] [mlr3] Running benchmark with 10 resampling iterations
## INFO  [08:03:19.452] [mlr3] Applying learner 'regr.svm' on task 'PredictionSet[1:13]' (iter 1/10)
## INFO  [08:03:19.471] [mlr3] Applying learner 'regr.svm' on task 'PredictionSet[1:13]' (iter 2/10)
## INFO  [08:03:19.487] [mlr3] Applying learner 'regr.svm' on task 'PredictionSet[1:13]' (iter 3/10)
## INFO  [08:03:19.502] [mlr3] Applying learner 'regr.svm' on task 'PredictionSet[1:13]' (iter 4/10)
## INFO  [08:03:19.517] [mlr3] Applying learner 'regr.svm' on task 'PredictionSet[1:13]' (iter 5/10)
## INFO  [08:03:19.534] [mlr3] Applying learner 'regr.svm' on task 'PredictionSet[1:13]' (iter 6/10)
## INFO  [08:03:19.550] [mlr3] Applying learner 'regr.svm' on task 'PredictionSet[1:13]' (iter 7/10)
## INFO  [08:03:19.566] [mlr3] Applying learner 'regr.svm' on task 'PredictionSet[1:13]' (iter 8/10)
## INFO  [08:03:19.581] [mlr3] Applying learner 'regr.svm' on task 'PredictionSet[1:13]' (iter 9/10)
## INFO  [08:03:19.596] [mlr3] Applying learner 'regr.svm' on task 'PredictionSet[1:13]' (iter 10/10)
## INFO  [08:03:19.613] [mlr3] Finished benchmark
## INFO  [08:03:19.644] [bbotk] Result of batch 10:
## INFO  [08:03:19.646] [bbotk]  tolerance gamma   epsilon           type kernel regr.rmse warnings errors
## INFO  [08:03:19.646] [bbotk]  0.5147143    50 0.6714286 eps-regression radial 0.0168573        0      0
## INFO  [08:03:19.646] [bbotk]  runtime_learners                                uhash
## INFO  [08:03:19.646] [bbotk]              0.06 b5bd879f-b969-4a50-9324-20491c864fee
## INFO  [08:03:19.650] [bbotk] Finished optimizing after 10 evaluation(s)
## INFO  [08:03:19.651] [bbotk] Result:
## INFO  [08:03:19.652] [bbotk]  tolerance    gamma   epsilon           type kernel learner_param_vals
## INFO  [08:03:19.652] [bbotk]      0.001 35.71457 0.5571429 eps-regression radial          <list[5]>
## INFO  [08:03:19.652] [bbotk]   x_domain  regr.rmse
## INFO  [08:03:19.652] [bbotk]  <list[5]> 0.01492091
## INFO  [08:03:19.799] [bbotk] Starting to optimize 3 parameter(s) with '<TunerGridSearch>' and '<TerminatorEvals> [n_evals=10, k=0]'
## INFO  [08:03:19.803] [bbotk] Evaluating 1 configuration(s)
## INFO  [08:03:19.816] [mlr3] Running benchmark with 10 resampling iterations
## INFO  [08:03:19.821] [mlr3] Applying learner 'regr.kknn' on task 'PredictionSet[1:13]' (iter 1/10)
## INFO  [08:03:19.843] [mlr3] Applying learner 'regr.kknn' on task 'PredictionSet[1:13]' (iter 2/10)
## INFO  [08:03:19.864] [mlr3] Applying learner 'regr.kknn' on task 'PredictionSet[1:13]' (iter 3/10)
## INFO  [08:03:19.884] [mlr3] Applying learner 'regr.kknn' on task 'PredictionSet[1:13]' (iter 4/10)
## INFO  [08:03:19.905] [mlr3] Applying learner 'regr.kknn' on task 'PredictionSet[1:13]' (iter 5/10)
## INFO  [08:03:19.927] [mlr3] Applying learner 'regr.kknn' on task 'PredictionSet[1:13]' (iter 6/10)
## INFO  [08:03:19.947] [mlr3] Applying learner 'regr.kknn' on task 'PredictionSet[1:13]' (iter 7/10)
## INFO  [08:03:19.968] [mlr3] Applying learner 'regr.kknn' on task 'PredictionSet[1:13]' (iter 8/10)
## INFO  [08:03:19.989] [mlr3] Applying learner 'regr.kknn' on task 'PredictionSet[1:13]' (iter 9/10)
## INFO  [08:03:20.010] [mlr3] Applying learner 'regr.kknn' on task 'PredictionSet[1:13]' (iter 10/10)
## INFO  [08:03:20.030] [mlr3] Finished benchmark
## INFO  [08:03:20.060] [bbotk] Result of batch 1:
## INFO  [08:03:20.062] [bbotk]  k distance kernel  regr.rmse warnings errors runtime_learners
## INFO  [08:03:20.062] [bbotk]  3 12.89286   rank 0.01119511        0      0            0.106
## INFO  [08:03:20.062] [bbotk]                                 uhash
## INFO  [08:03:20.062] [bbotk]  9e286604-8f9f-4d5c-a61a-d2fc08c6e1ce
## INFO  [08:03:20.063] [bbotk] Evaluating 1 configuration(s)
## INFO  [08:03:20.074] [mlr3] Running benchmark with 10 resampling iterations
## INFO  [08:03:20.079] [mlr3] Applying learner 'regr.kknn' on task 'PredictionSet[1:13]' (iter 1/10)
## INFO  [08:03:20.099] [mlr3] Applying learner 'regr.kknn' on task 'PredictionSet[1:13]' (iter 2/10)
## INFO  [08:03:20.120] [mlr3] Applying learner 'regr.kknn' on task 'PredictionSet[1:13]' (iter 3/10)
## INFO  [08:03:20.149] [mlr3] Applying learner 'regr.kknn' on task 'PredictionSet[1:13]' (iter 4/10)
## INFO  [08:03:20.169] [mlr3] Applying learner 'regr.kknn' on task 'PredictionSet[1:13]' (iter 5/10)
## INFO  [08:03:20.190] [mlr3] Applying learner 'regr.kknn' on task 'PredictionSet[1:13]' (iter 6/10)
## INFO  [08:03:20.211] [mlr3] Applying learner 'regr.kknn' on task 'PredictionSet[1:13]' (iter 7/10)
## INFO  [08:03:20.232] [mlr3] Applying learner 'regr.kknn' on task 'PredictionSet[1:13]' (iter 8/10)
## INFO  [08:03:20.252] [mlr3] Applying learner 'regr.kknn' on task 'PredictionSet[1:13]' (iter 9/10)
## INFO  [08:03:20.273] [mlr3] Applying learner 'regr.kknn' on task 'PredictionSet[1:13]' (iter 10/10)
## INFO  [08:03:20.294] [mlr3] Finished benchmark
## INFO  [08:03:20.325] [bbotk] Result of batch 2:
## INFO  [08:03:20.327] [bbotk]   k distance   kernel  regr.rmse warnings errors runtime_learners
## INFO  [08:03:20.327] [bbotk]  15 12.89286 biweight 0.01160163        0      0            0.107
## INFO  [08:03:20.327] [bbotk]                                 uhash
## INFO  [08:03:20.327] [bbotk]  bf723aeb-9135-43d4-8cce-9ecdad1581b4
## INFO  [08:03:20.328] [bbotk] Evaluating 1 configuration(s)
## INFO  [08:03:20.339] [mlr3] Running benchmark with 10 resampling iterations
## INFO  [08:03:20.344] [mlr3] Applying learner 'regr.kknn' on task 'PredictionSet[1:13]' (iter 1/10)
## INFO  [08:03:20.365] [mlr3] Applying learner 'regr.kknn' on task 'PredictionSet[1:13]' (iter 2/10)
## INFO  [08:03:20.385] [mlr3] Applying learner 'regr.kknn' on task 'PredictionSet[1:13]' (iter 3/10)
## INFO  [08:03:20.405] [mlr3] Applying learner 'regr.kknn' on task 'PredictionSet[1:13]' (iter 4/10)
## INFO  [08:03:20.426] [mlr3] Applying learner 'regr.kknn' on task 'PredictionSet[1:13]' (iter 5/10)
## INFO  [08:03:20.448] [mlr3] Applying learner 'regr.kknn' on task 'PredictionSet[1:13]' (iter 6/10)
## INFO  [08:03:20.468] [mlr3] Applying learner 'regr.kknn' on task 'PredictionSet[1:13]' (iter 7/10)
## INFO  [08:03:20.488] [mlr3] Applying learner 'regr.kknn' on task 'PredictionSet[1:13]' (iter 8/10)
## INFO  [08:03:20.510] [mlr3] Applying learner 'regr.kknn' on task 'PredictionSet[1:13]' (iter 9/10)
## INFO  [08:03:20.531] [mlr3] Applying learner 'regr.kknn' on task 'PredictionSet[1:13]' (iter 10/10)
## INFO  [08:03:20.557] [mlr3] Finished benchmark
## INFO  [08:03:20.590] [bbotk] Result of batch 3:
## INFO  [08:03:20.591] [bbotk]  k distance     kernel   regr.rmse warnings errors runtime_learners
## INFO  [08:03:20.591] [bbotk]  3      0.1 triangular 0.009026602        0      0            0.104
## INFO  [08:03:20.591] [bbotk]                                 uhash
## INFO  [08:03:20.591] [bbotk]  52e2c4ea-9a15-4543-80f9-01fca60ba7ee
## INFO  [08:03:20.593] [bbotk] Evaluating 1 configuration(s)
## INFO  [08:03:20.603] [mlr3] Running benchmark with 10 resampling iterations
## INFO  [08:03:20.608] [mlr3] Applying learner 'regr.kknn' on task 'PredictionSet[1:13]' (iter 1/10)
## INFO  [08:03:20.628] [mlr3] Applying learner 'regr.kknn' on task 'PredictionSet[1:13]' (iter 2/10)
## INFO  [08:03:20.648] [mlr3] Applying learner 'regr.kknn' on task 'PredictionSet[1:13]' (iter 3/10)
## INFO  [08:03:20.670] [mlr3] Applying learner 'regr.kknn' on task 'PredictionSet[1:13]' (iter 4/10)
## INFO  [08:03:20.691] [mlr3] Applying learner 'regr.kknn' on task 'PredictionSet[1:13]' (iter 5/10)
## INFO  [08:03:20.711] [mlr3] Applying learner 'regr.kknn' on task 'PredictionSet[1:13]' (iter 6/10)
## INFO  [08:03:20.732] [mlr3] Applying learner 'regr.kknn' on task 'PredictionSet[1:13]' (iter 7/10)
## INFO  [08:03:20.753] [mlr3] Applying learner 'regr.kknn' on task 'PredictionSet[1:13]' (iter 8/10)
## INFO  [08:03:20.773] [mlr3] Applying learner 'regr.kknn' on task 'PredictionSet[1:13]' (iter 9/10)
## INFO  [08:03:20.794] [mlr3] Applying learner 'regr.kknn' on task 'PredictionSet[1:13]' (iter 10/10)
## INFO  [08:03:20.815] [mlr3] Finished benchmark
## INFO  [08:03:20.846] [bbotk] Result of batch 4:
## INFO  [08:03:20.848] [bbotk]   k distance       kernel  regr.rmse warnings errors runtime_learners
## INFO  [08:03:20.848] [bbotk]  13 5.785714 epanechnikov 0.01162227        0      0            0.104
## INFO  [08:03:20.848] [bbotk]                                 uhash
## INFO  [08:03:20.848] [bbotk]  21095f1f-cb2f-49fd-8ad7-e911fe42d5db
## INFO  [08:03:20.849] [bbotk] Evaluating 1 configuration(s)
## INFO  [08:03:20.860] [mlr3] Running benchmark with 10 resampling iterations
## INFO  [08:03:20.865] [mlr3] Applying learner 'regr.kknn' on task 'PredictionSet[1:13]' (iter 1/10)
## INFO  [08:03:20.886] [mlr3] Applying learner 'regr.kknn' on task 'PredictionSet[1:13]' (iter 2/10)
## INFO  [08:03:20.907] [mlr3] Applying learner 'regr.kknn' on task 'PredictionSet[1:13]' (iter 3/10)
## INFO  [08:03:20.934] [mlr3] Applying learner 'regr.kknn' on task 'PredictionSet[1:13]' (iter 4/10)
## INFO  [08:03:20.954] [mlr3] Applying learner 'regr.kknn' on task 'PredictionSet[1:13]' (iter 5/10)
## INFO  [08:03:20.976] [mlr3] Applying learner 'regr.kknn' on task 'PredictionSet[1:13]' (iter 6/10)
## INFO  [08:03:20.996] [mlr3] Applying learner 'regr.kknn' on task 'PredictionSet[1:13]' (iter 7/10)
## INFO  [08:03:21.016] [mlr3] Applying learner 'regr.kknn' on task 'PredictionSet[1:13]' (iter 8/10)
## INFO  [08:03:21.038] [mlr3] Applying learner 'regr.kknn' on task 'PredictionSet[1:13]' (iter 9/10)
## INFO  [08:03:21.059] [mlr3] Applying learner 'regr.kknn' on task 'PredictionSet[1:13]' (iter 10/10)
## INFO  [08:03:21.078] [mlr3] Finished benchmark
## INFO  [08:03:21.110] [bbotk] Result of batch 5:
## INFO  [08:03:21.112] [bbotk]  k distance      kernel  regr.rmse warnings errors runtime_learners
## INFO  [08:03:21.112] [bbotk]  6 8.628571 rectangular 0.01241484        0      0            0.101
## INFO  [08:03:21.112] [bbotk]                                 uhash
## INFO  [08:03:21.112] [bbotk]  728e1b98-b980-4a35-8851-a38898549640
## INFO  [08:03:21.114] [bbotk] Evaluating 1 configuration(s)
## INFO  [08:03:21.124] [mlr3] Running benchmark with 10 resampling iterations
## INFO  [08:03:21.129] [mlr3] Applying learner 'regr.kknn' on task 'PredictionSet[1:13]' (iter 1/10)
## INFO  [08:03:21.150] [mlr3] Applying learner 'regr.kknn' on task 'PredictionSet[1:13]' (iter 2/10)
## INFO  [08:03:21.170] [mlr3] Applying learner 'regr.kknn' on task 'PredictionSet[1:13]' (iter 3/10)
## INFO  [08:03:21.192] [mlr3] Applying learner 'regr.kknn' on task 'PredictionSet[1:13]' (iter 4/10)
## INFO  [08:03:21.213] [mlr3] Applying learner 'regr.kknn' on task 'PredictionSet[1:13]' (iter 5/10)
## INFO  [08:03:21.234] [mlr3] Applying learner 'regr.kknn' on task 'PredictionSet[1:13]' (iter 6/10)
## INFO  [08:03:21.256] [mlr3] Applying learner 'regr.kknn' on task 'PredictionSet[1:13]' (iter 7/10)
## INFO  [08:03:21.278] [mlr3] Applying learner 'regr.kknn' on task 'PredictionSet[1:13]' (iter 8/10)
## INFO  [08:03:21.305] [mlr3] Applying learner 'regr.kknn' on task 'PredictionSet[1:13]' (iter 9/10)
## INFO  [08:03:21.326] [mlr3] Applying learner 'regr.kknn' on task 'PredictionSet[1:13]' (iter 10/10)
## INFO  [08:03:21.348] [mlr3] Finished benchmark
## INFO  [08:03:21.379] [bbotk] Result of batch 6:
## INFO  [08:03:21.381] [bbotk]   k distance kernel  regr.rmse warnings errors runtime_learners
## INFO  [08:03:21.381] [bbotk]  20       20   rank 0.01222089        0      0            0.115
## INFO  [08:03:21.381] [bbotk]                                 uhash
## INFO  [08:03:21.381] [bbotk]  28bb6814-2fb6-4faa-840e-701d8e8b66e3
## INFO  [08:03:21.382] [bbotk] Evaluating 1 configuration(s)
## INFO  [08:03:21.392] [mlr3] Running benchmark with 10 resampling iterations
## INFO  [08:03:21.397] [mlr3] Applying learner 'regr.kknn' on task 'PredictionSet[1:13]' (iter 1/10)
## INFO  [08:03:21.419] [mlr3] Applying learner 'regr.kknn' on task 'PredictionSet[1:13]' (iter 2/10)
## INFO  [08:03:21.440] [mlr3] Applying learner 'regr.kknn' on task 'PredictionSet[1:13]' (iter 3/10)
## INFO  [08:03:21.460] [mlr3] Applying learner 'regr.kknn' on task 'PredictionSet[1:13]' (iter 4/10)
## INFO  [08:03:21.481] [mlr3] Applying learner 'regr.kknn' on task 'PredictionSet[1:13]' (iter 5/10)
## INFO  [08:03:21.503] [mlr3] Applying learner 'regr.kknn' on task 'PredictionSet[1:13]' (iter 6/10)
## INFO  [08:03:21.523] [mlr3] Applying learner 'regr.kknn' on task 'PredictionSet[1:13]' (iter 7/10)
## INFO  [08:03:21.543] [mlr3] Applying learner 'regr.kknn' on task 'PredictionSet[1:13]' (iter 8/10)
## INFO  [08:03:21.564] [mlr3] Applying learner 'regr.kknn' on task 'PredictionSet[1:13]' (iter 9/10)
## INFO  [08:03:21.585] [mlr3] Applying learner 'regr.kknn' on task 'PredictionSet[1:13]' (iter 10/10)
## INFO  [08:03:21.605] [mlr3] Finished benchmark
## INFO  [08:03:21.638] [bbotk] Result of batch 7:
## INFO  [08:03:21.640] [bbotk]   k distance  kernel  regr.rmse warnings errors runtime_learners
## INFO  [08:03:21.640] [bbotk]  15       20 optimal 0.01169285        0      0            0.102
## INFO  [08:03:21.640] [bbotk]                                 uhash
## INFO  [08:03:21.640] [bbotk]  60f3fe77-c86f-4679-83fc-60315259b305
## INFO  [08:03:21.641] [bbotk] Evaluating 1 configuration(s)
## INFO  [08:03:21.652] [mlr3] Running benchmark with 10 resampling iterations
## INFO  [08:03:21.657] [mlr3] Applying learner 'regr.kknn' on task 'PredictionSet[1:13]' (iter 1/10)
## INFO  [08:03:21.683] [mlr3] Applying learner 'regr.kknn' on task 'PredictionSet[1:13]' (iter 2/10)
## INFO  [08:03:21.704] [mlr3] Applying learner 'regr.kknn' on task 'PredictionSet[1:13]' (iter 3/10)
## INFO  [08:03:21.726] [mlr3] Applying learner 'regr.kknn' on task 'PredictionSet[1:13]' (iter 4/10)
## INFO  [08:03:21.746] [mlr3] Applying learner 'regr.kknn' on task 'PredictionSet[1:13]' (iter 5/10)
## INFO  [08:03:21.766] [mlr3] Applying learner 'regr.kknn' on task 'PredictionSet[1:13]' (iter 6/10)
## INFO  [08:03:21.788] [mlr3] Applying learner 'regr.kknn' on task 'PredictionSet[1:13]' (iter 7/10)
## INFO  [08:03:21.809] [mlr3] Applying learner 'regr.kknn' on task 'PredictionSet[1:13]' (iter 8/10)
## INFO  [08:03:21.829] [mlr3] Applying learner 'regr.kknn' on task 'PredictionSet[1:13]' (iter 9/10)
## INFO  [08:03:21.849] [mlr3] Applying learner 'regr.kknn' on task 'PredictionSet[1:13]' (iter 10/10)
## INFO  [08:03:21.871] [mlr3] Finished benchmark
## INFO  [08:03:21.902] [bbotk] Result of batch 8:
## INFO  [08:03:21.904] [bbotk]   k distance      kernel  regr.rmse warnings errors runtime_learners
## INFO  [08:03:21.904] [bbotk]  16 14.31429 rectangular 0.01278176        0      0            0.105
## INFO  [08:03:21.904] [bbotk]                                 uhash
## INFO  [08:03:21.904] [bbotk]  1dfab85c-2d2d-487b-9986-d79ed4068e82
## INFO  [08:03:21.905] [bbotk] Evaluating 1 configuration(s)
## INFO  [08:03:21.915] [mlr3] Running benchmark with 10 resampling iterations
## INFO  [08:03:21.920] [mlr3] Applying learner 'regr.kknn' on task 'PredictionSet[1:13]' (iter 1/10)
## INFO  [08:03:21.942] [mlr3] Applying learner 'regr.kknn' on task 'PredictionSet[1:13]' (iter 2/10)
## INFO  [08:03:21.962] [mlr3] Applying learner 'regr.kknn' on task 'PredictionSet[1:13]' (iter 3/10)
## INFO  [08:03:21.982] [mlr3] Applying learner 'regr.kknn' on task 'PredictionSet[1:13]' (iter 4/10)
## INFO  [08:03:22.003] [mlr3] Applying learner 'regr.kknn' on task 'PredictionSet[1:13]' (iter 5/10)
## INFO  [08:03:22.025] [mlr3] Applying learner 'regr.kknn' on task 'PredictionSet[1:13]' (iter 6/10)
## INFO  [08:03:22.045] [mlr3] Applying learner 'regr.kknn' on task 'PredictionSet[1:13]' (iter 7/10)
## INFO  [08:03:22.072] [mlr3] Applying learner 'regr.kknn' on task 'PredictionSet[1:13]' (iter 8/10)
## INFO  [08:03:22.094] [mlr3] Applying learner 'regr.kknn' on task 'PredictionSet[1:13]' (iter 9/10)
## INFO  [08:03:22.114] [mlr3] Applying learner 'regr.kknn' on task 'PredictionSet[1:13]' (iter 10/10)
## INFO  [08:03:22.133] [mlr3] Finished benchmark
## INFO  [08:03:22.166] [bbotk] Result of batch 9:
## INFO  [08:03:22.168] [bbotk]  k distance     kernel  regr.rmse warnings errors runtime_learners
## INFO  [08:03:22.168] [bbotk]  5 17.15714 triangular 0.01160543        0      0            0.105
## INFO  [08:03:22.168] [bbotk]                                 uhash
## INFO  [08:03:22.168] [bbotk]  e9cf01c8-c08f-446d-a7f1-860b6e28db3d
## INFO  [08:03:22.169] [bbotk] Evaluating 1 configuration(s)
## INFO  [08:03:22.180] [mlr3] Running benchmark with 10 resampling iterations
## INFO  [08:03:22.185] [mlr3] Applying learner 'regr.kknn' on task 'PredictionSet[1:13]' (iter 1/10)
## INFO  [08:03:22.204] [mlr3] Applying learner 'regr.kknn' on task 'PredictionSet[1:13]' (iter 2/10)
## INFO  [08:03:22.225] [mlr3] Applying learner 'regr.kknn' on task 'PredictionSet[1:13]' (iter 3/10)
## INFO  [08:03:22.247] [mlr3] Applying learner 'regr.kknn' on task 'PredictionSet[1:13]' (iter 4/10)
## INFO  [08:03:22.267] [mlr3] Applying learner 'regr.kknn' on task 'PredictionSet[1:13]' (iter 5/10)
## INFO  [08:03:22.287] [mlr3] Applying learner 'regr.kknn' on task 'PredictionSet[1:13]' (iter 6/10)
## INFO  [08:03:22.308] [mlr3] Applying learner 'regr.kknn' on task 'PredictionSet[1:13]' (iter 7/10)
## INFO  [08:03:22.330] [mlr3] Applying learner 'regr.kknn' on task 'PredictionSet[1:13]' (iter 8/10)
## INFO  [08:03:22.350] [mlr3] Applying learner 'regr.kknn' on task 'PredictionSet[1:13]' (iter 9/10)
## INFO  [08:03:22.370] [mlr3] Applying learner 'regr.kknn' on task 'PredictionSet[1:13]' (iter 10/10)
## INFO  [08:03:22.392] [mlr3] Finished benchmark
## INFO  [08:03:22.430] [bbotk] Result of batch 10:
## INFO  [08:03:22.432] [bbotk]  k distance       kernel  regr.rmse warnings errors runtime_learners
## INFO  [08:03:22.432] [bbotk]  5 1.521429 epanechnikov 0.01113409        0      0              0.1
## INFO  [08:03:22.432] [bbotk]                                 uhash
## INFO  [08:03:22.432] [bbotk]  ad601af7-633a-463a-86fc-47f0060ef63d
## INFO  [08:03:22.437] [bbotk] Finished optimizing after 10 evaluation(s)
## INFO  [08:03:22.437] [bbotk] Result:
## INFO  [08:03:22.438] [bbotk]  k distance     kernel learner_param_vals  x_domain   regr.rmse
## INFO  [08:03:22.438] [bbotk]  3      0.1 triangular          <list[3]> <list[3]> 0.009026602
## INFO  [08:03:22.646] [bbotk] Starting to optimize 2 parameter(s) with '<TunerGridSearch>' and '<TerminatorEvals> [n_evals=10, k=0]'
## INFO  [08:03:22.649] [bbotk] Evaluating 1 configuration(s)
## INFO  [08:03:22.671] [mlr3] Running benchmark with 10 resampling iterations
## INFO  [08:03:22.676] [mlr3] Applying learner 'regr.ranger' on task 'PredictionSet[1:13]' (iter 1/10)
## INFO  [08:03:22.718] [mlr3] Applying learner 'regr.ranger' on task 'PredictionSet[1:13]' (iter 2/10)
## INFO  [08:03:22.751] [mlr3] Applying learner 'regr.ranger' on task 'PredictionSet[1:13]' (iter 3/10)
## INFO  [08:03:22.785] [mlr3] Applying learner 'regr.ranger' on task 'PredictionSet[1:13]' (iter 4/10)
## INFO  [08:03:22.817] [mlr3] Applying learner 'regr.ranger' on task 'PredictionSet[1:13]' (iter 5/10)
## INFO  [08:03:22.851] [mlr3] Applying learner 'regr.ranger' on task 'PredictionSet[1:13]' (iter 6/10)
## INFO  [08:03:22.884] [mlr3] Applying learner 'regr.ranger' on task 'PredictionSet[1:13]' (iter 7/10)
## INFO  [08:03:22.918] [mlr3] Applying learner 'regr.ranger' on task 'PredictionSet[1:13]' (iter 8/10)
## INFO  [08:03:22.951] [mlr3] Applying learner 'regr.ranger' on task 'PredictionSet[1:13]' (iter 9/10)
## INFO  [08:03:22.986] [mlr3] Applying learner 'regr.ranger' on task 'PredictionSet[1:13]' (iter 10/10)
## INFO  [08:03:23.018] [mlr3] Finished benchmark
## INFO  [08:03:23.046] [bbotk] Result of batch 1:
## INFO  [08:03:23.048] [bbotk]  max.depth num.trees   regr.rmse warnings errors runtime_learners
## INFO  [08:03:23.048] [bbotk]          0       264 0.009071193        0      0             0.23
## INFO  [08:03:23.048] [bbotk]                                 uhash
## INFO  [08:03:23.048] [bbotk]  6bc15d93-bbd5-442b-98cf-f16d37cf2be6
## INFO  [08:03:23.050] [bbotk] Evaluating 1 configuration(s)
## INFO  [08:03:23.072] [mlr3] Running benchmark with 10 resampling iterations
## INFO  [08:03:23.077] [mlr3] Applying learner 'regr.ranger' on task 'PredictionSet[1:13]' (iter 1/10)
## INFO  [08:03:23.095] [mlr3] Applying learner 'regr.ranger' on task 'PredictionSet[1:13]' (iter 2/10)
## INFO  [08:03:23.113] [mlr3] Applying learner 'regr.ranger' on task 'PredictionSet[1:13]' (iter 3/10)
## INFO  [08:03:23.133] [mlr3] Applying learner 'regr.ranger' on task 'PredictionSet[1:13]' (iter 4/10)
## INFO  [08:03:23.152] [mlr3] Applying learner 'regr.ranger' on task 'PredictionSet[1:13]' (iter 5/10)
## INFO  [08:03:23.170] [mlr3] Applying learner 'regr.ranger' on task 'PredictionSet[1:13]' (iter 6/10)
## INFO  [08:03:23.189] [mlr3] Applying learner 'regr.ranger' on task 'PredictionSet[1:13]' (iter 7/10)
## INFO  [08:03:23.208] [mlr3] Applying learner 'regr.ranger' on task 'PredictionSet[1:13]' (iter 8/10)
## INFO  [08:03:23.236] [mlr3] Applying learner 'regr.ranger' on task 'PredictionSet[1:13]' (iter 9/10)
## INFO  [08:03:23.254] [mlr3] Applying learner 'regr.ranger' on task 'PredictionSet[1:13]' (iter 10/10)
## INFO  [08:03:23.272] [mlr3] Finished benchmark
## INFO  [08:03:23.306] [bbotk] Result of batch 2:
## INFO  [08:03:23.307] [bbotk]  max.depth num.trees   regr.rmse warnings errors runtime_learners
## INFO  [08:03:23.307] [bbotk]         20        53 0.008983313        0      0            0.092
## INFO  [08:03:23.307] [bbotk]                                 uhash
## INFO  [08:03:23.307] [bbotk]  82011d39-f66a-4dde-a00c-c2449f539842
## INFO  [08:03:23.309] [bbotk] Evaluating 1 configuration(s)
## INFO  [08:03:23.330] [mlr3] Running benchmark with 10 resampling iterations
## INFO  [08:03:23.335] [mlr3] Applying learner 'regr.ranger' on task 'PredictionSet[1:13]' (iter 1/10)
## INFO  [08:03:23.360] [mlr3] Applying learner 'regr.ranger' on task 'PredictionSet[1:13]' (iter 2/10)
## INFO  [08:03:23.384] [mlr3] Applying learner 'regr.ranger' on task 'PredictionSet[1:13]' (iter 3/10)
## INFO  [08:03:23.407] [mlr3] Applying learner 'regr.ranger' on task 'PredictionSet[1:13]' (iter 4/10)
## INFO  [08:03:23.432] [mlr3] Applying learner 'regr.ranger' on task 'PredictionSet[1:13]' (iter 5/10)
## INFO  [08:03:23.457] [mlr3] Applying learner 'regr.ranger' on task 'PredictionSet[1:13]' (iter 6/10)
## INFO  [08:03:23.481] [mlr3] Applying learner 'regr.ranger' on task 'PredictionSet[1:13]' (iter 7/10)
## INFO  [08:03:23.505] [mlr3] Applying learner 'regr.ranger' on task 'PredictionSet[1:13]' (iter 8/10)
## INFO  [08:03:23.530] [mlr3] Applying learner 'regr.ranger' on task 'PredictionSet[1:13]' (iter 9/10)
## INFO  [08:03:23.554] [mlr3] Applying learner 'regr.ranger' on task 'PredictionSet[1:13]' (iter 10/10)
## INFO  [08:03:23.577] [mlr3] Finished benchmark
## INFO  [08:03:23.610] [bbotk] Result of batch 3:
## INFO  [08:03:23.612] [bbotk]  max.depth num.trees  regr.rmse warnings errors runtime_learners
## INFO  [08:03:23.612] [bbotk]         12       132 0.00908525        0      0            0.134
## INFO  [08:03:23.612] [bbotk]                                 uhash
## INFO  [08:03:23.612] [bbotk]  bdce2edf-be8d-4808-9926-76671f5855d4
## INFO  [08:03:23.613] [bbotk] Evaluating 1 configuration(s)
## INFO  [08:03:23.642] [mlr3] Running benchmark with 10 resampling iterations
## INFO  [08:03:23.647] [mlr3] Applying learner 'regr.ranger' on task 'PredictionSet[1:13]' (iter 1/10)
## INFO  [08:03:23.687] [mlr3] Applying learner 'regr.ranger' on task 'PredictionSet[1:13]' (iter 2/10)
## INFO  [08:03:23.725] [mlr3] Applying learner 'regr.ranger' on task 'PredictionSet[1:13]' (iter 3/10)
## INFO  [08:03:23.764] [mlr3] Applying learner 'regr.ranger' on task 'PredictionSet[1:13]' (iter 4/10)
## INFO  [08:03:23.802] [mlr3] Applying learner 'regr.ranger' on task 'PredictionSet[1:13]' (iter 5/10)
## INFO  [08:03:23.841] [mlr3] Applying learner 'regr.ranger' on task 'PredictionSet[1:13]' (iter 6/10)
## INFO  [08:03:23.880] [mlr3] Applying learner 'regr.ranger' on task 'PredictionSet[1:13]' (iter 7/10)
## INFO  [08:03:23.919] [mlr3] Applying learner 'regr.ranger' on task 'PredictionSet[1:13]' (iter 8/10)
## INFO  [08:03:23.958] [mlr3] Applying learner 'regr.ranger' on task 'PredictionSet[1:13]' (iter 9/10)
## INFO  [08:03:23.997] [mlr3] Applying learner 'regr.ranger' on task 'PredictionSet[1:13]' (iter 10/10)
## INFO  [08:03:24.035] [mlr3] Finished benchmark
## INFO  [08:03:24.066] [bbotk] Result of batch 4:
## INFO  [08:03:24.068] [bbotk]  max.depth num.trees  regr.rmse warnings errors runtime_learners
## INFO  [08:03:24.068] [bbotk]         14       343 0.00903371        0      0            0.281
## INFO  [08:03:24.068] [bbotk]                                 uhash
## INFO  [08:03:24.068] [bbotk]  a9543e21-33e6-4195-83fc-2866495506c0
## INFO  [08:03:24.070] [bbotk] Evaluating 1 configuration(s)
## INFO  [08:03:24.090] [mlr3] Running benchmark with 10 resampling iterations
## INFO  [08:03:24.095] [mlr3] Applying learner 'regr.ranger' on task 'PredictionSet[1:13]' (iter 1/10)
## INFO  [08:03:24.119] [mlr3] Applying learner 'regr.ranger' on task 'PredictionSet[1:13]' (iter 2/10)
## INFO  [08:03:24.141] [mlr3] Applying learner 'regr.ranger' on task 'PredictionSet[1:13]' (iter 3/10)
## INFO  [08:03:24.163] [mlr3] Applying learner 'regr.ranger' on task 'PredictionSet[1:13]' (iter 4/10)
## INFO  [08:03:24.186] [mlr3] Applying learner 'regr.ranger' on task 'PredictionSet[1:13]' (iter 5/10)
## INFO  [08:03:24.209] [mlr3] Applying learner 'regr.ranger' on task 'PredictionSet[1:13]' (iter 6/10)
## INFO  [08:03:24.239] [mlr3] Applying learner 'regr.ranger' on task 'PredictionSet[1:13]' (iter 7/10)
## INFO  [08:03:24.263] [mlr3] Applying learner 'regr.ranger' on task 'PredictionSet[1:13]' (iter 8/10)
## INFO  [08:03:24.285] [mlr3] Applying learner 'regr.ranger' on task 'PredictionSet[1:13]' (iter 9/10)
## INFO  [08:03:24.307] [mlr3] Applying learner 'regr.ranger' on task 'PredictionSet[1:13]' (iter 10/10)
## INFO  [08:03:24.329] [mlr3] Finished benchmark
## INFO  [08:03:24.360] [bbotk] Result of batch 5:
## INFO  [08:03:24.362] [bbotk]  max.depth num.trees   regr.rmse warnings errors runtime_learners
## INFO  [08:03:24.362] [bbotk]          8       106 0.009153907        0      0            0.127
## INFO  [08:03:24.362] [bbotk]                                 uhash
## INFO  [08:03:24.362] [bbotk]  48faf7f1-6743-41e1-b375-111a69b4d33d
## INFO  [08:03:24.364] [bbotk] Evaluating 1 configuration(s)
## INFO  [08:03:24.384] [mlr3] Running benchmark with 10 resampling iterations
## INFO  [08:03:24.389] [mlr3] Applying learner 'regr.ranger' on task 'PredictionSet[1:13]' (iter 1/10)
## INFO  [08:03:24.407] [mlr3] Applying learner 'regr.ranger' on task 'PredictionSet[1:13]' (iter 2/10)
## INFO  [08:03:24.424] [mlr3] Applying learner 'regr.ranger' on task 'PredictionSet[1:13]' (iter 3/10)
## INFO  [08:03:24.440] [mlr3] Applying learner 'regr.ranger' on task 'PredictionSet[1:13]' (iter 4/10)
## INFO  [08:03:24.457] [mlr3] Applying learner 'regr.ranger' on task 'PredictionSet[1:13]' (iter 5/10)
## INFO  [08:03:24.473] [mlr3] Applying learner 'regr.ranger' on task 'PredictionSet[1:13]' (iter 6/10)
## INFO  [08:03:24.491] [mlr3] Applying learner 'regr.ranger' on task 'PredictionSet[1:13]' (iter 7/10)
## INFO  [08:03:24.508] [mlr3] Applying learner 'regr.ranger' on task 'PredictionSet[1:13]' (iter 8/10)
## INFO  [08:03:24.524] [mlr3] Applying learner 'regr.ranger' on task 'PredictionSet[1:13]' (iter 9/10)
## INFO  [08:03:24.541] [mlr3] Applying learner 'regr.ranger' on task 'PredictionSet[1:13]' (iter 10/10)
## INFO  [08:03:24.558] [mlr3] Finished benchmark
## INFO  [08:03:24.597] [bbotk] Result of batch 6:
## INFO  [08:03:24.598] [bbotk]  max.depth num.trees   regr.rmse warnings errors runtime_learners
## INFO  [08:03:24.598] [bbotk]         20        27 0.009430811        0      0            0.068
## INFO  [08:03:24.598] [bbotk]                                 uhash
## INFO  [08:03:24.598] [bbotk]  7c206ff9-d7ca-4a4a-a714-055cad276fa5
## INFO  [08:03:24.600] [bbotk] Evaluating 1 configuration(s)
## INFO  [08:03:24.621] [mlr3] Running benchmark with 10 resampling iterations
## INFO  [08:03:24.626] [mlr3] Applying learner 'regr.ranger' on task 'PredictionSet[1:13]' (iter 1/10)
## INFO  [08:03:24.675] [mlr3] Applying learner 'regr.ranger' on task 'PredictionSet[1:13]' (iter 2/10)
## INFO  [08:03:24.723] [mlr3] Applying learner 'regr.ranger' on task 'PredictionSet[1:13]' (iter 3/10)
## INFO  [08:03:24.770] [mlr3] Applying learner 'regr.ranger' on task 'PredictionSet[1:13]' (iter 4/10)
## INFO  [08:03:24.817] [mlr3] Applying learner 'regr.ranger' on task 'PredictionSet[1:13]' (iter 5/10)
## INFO  [08:03:24.866] [mlr3] Applying learner 'regr.ranger' on task 'PredictionSet[1:13]' (iter 6/10)
## INFO  [08:03:24.913] [mlr3] Applying learner 'regr.ranger' on task 'PredictionSet[1:13]' (iter 7/10)
## INFO  [08:03:24.960] [mlr3] Applying learner 'regr.ranger' on task 'PredictionSet[1:13]' (iter 8/10)
## INFO  [08:03:25.007] [mlr3] Applying learner 'regr.ranger' on task 'PredictionSet[1:13]' (iter 9/10)
## INFO  [08:03:25.054] [mlr3] Applying learner 'regr.ranger' on task 'PredictionSet[1:13]' (iter 10/10)
## INFO  [08:03:25.102] [mlr3] Finished benchmark
## INFO  [08:03:25.133] [bbotk] Result of batch 7:
## INFO  [08:03:25.134] [bbotk]  max.depth num.trees   regr.rmse warnings errors runtime_learners
## INFO  [08:03:25.134] [bbotk]         19       474 0.009108865        0      0            0.372
## INFO  [08:03:25.134] [bbotk]                                 uhash
## INFO  [08:03:25.134] [bbotk]  06fcde34-0b2f-43ec-a7a5-c69c785070b1
## INFO  [08:03:25.136] [bbotk] Evaluating 1 configuration(s)
## INFO  [08:03:25.158] [mlr3] Running benchmark with 10 resampling iterations
## INFO  [08:03:25.164] [mlr3] Applying learner 'regr.ranger' on task 'PredictionSet[1:13]' (iter 1/10)
## INFO  [08:03:25.207] [mlr3] Applying learner 'regr.ranger' on task 'PredictionSet[1:13]' (iter 2/10)
## INFO  [08:03:25.252] [mlr3] Applying learner 'regr.ranger' on task 'PredictionSet[1:13]' (iter 3/10)
## INFO  [08:03:25.303] [mlr3] Applying learner 'regr.ranger' on task 'PredictionSet[1:13]' (iter 4/10)
## INFO  [08:03:25.349] [mlr3] Applying learner 'regr.ranger' on task 'PredictionSet[1:13]' (iter 5/10)
## INFO  [08:03:25.394] [mlr3] Applying learner 'regr.ranger' on task 'PredictionSet[1:13]' (iter 6/10)
## INFO  [08:03:25.437] [mlr3] Applying learner 'regr.ranger' on task 'PredictionSet[1:13]' (iter 7/10)
## INFO  [08:03:25.481] [mlr3] Applying learner 'regr.ranger' on task 'PredictionSet[1:13]' (iter 8/10)
## INFO  [08:03:25.525] [mlr3] Applying learner 'regr.ranger' on task 'PredictionSet[1:13]' (iter 9/10)
## INFO  [08:03:25.569] [mlr3] Applying learner 'regr.ranger' on task 'PredictionSet[1:13]' (iter 10/10)
## INFO  [08:03:25.613] [mlr3] Finished benchmark
## INFO  [08:03:25.644] [bbotk] Result of batch 8:
## INFO  [08:03:25.646] [bbotk]  max.depth num.trees   regr.rmse warnings errors runtime_learners
## INFO  [08:03:25.646] [bbotk]         20       422 0.008984264        0      0            0.335
## INFO  [08:03:25.646] [bbotk]                                 uhash
## INFO  [08:03:25.646] [bbotk]  3d220056-1fa2-4bdd-9d6c-45d3068b23f6
## INFO  [08:03:25.647] [bbotk] Evaluating 1 configuration(s)
## INFO  [08:03:25.668] [mlr3] Running benchmark with 10 resampling iterations
## INFO  [08:03:25.673] [mlr3] Applying learner 'regr.ranger' on task 'PredictionSet[1:13]' (iter 1/10)
## INFO  [08:03:25.697] [mlr3] Applying learner 'regr.ranger' on task 'PredictionSet[1:13]' (iter 2/10)
## INFO  [08:03:25.720] [mlr3] Applying learner 'regr.ranger' on task 'PredictionSet[1:13]' (iter 3/10)
## INFO  [08:03:25.742] [mlr3] Applying learner 'regr.ranger' on task 'PredictionSet[1:13]' (iter 4/10)
## INFO  [08:03:25.767] [mlr3] Applying learner 'regr.ranger' on task 'PredictionSet[1:13]' (iter 5/10)
## INFO  [08:03:25.790] [mlr3] Applying learner 'regr.ranger' on task 'PredictionSet[1:13]' (iter 6/10)
## INFO  [08:03:25.812] [mlr3] Applying learner 'regr.ranger' on task 'PredictionSet[1:13]' (iter 7/10)
## INFO  [08:03:25.837] [mlr3] Applying learner 'regr.ranger' on task 'PredictionSet[1:13]' (iter 8/10)
## INFO  [08:03:25.860] [mlr3] Applying learner 'regr.ranger' on task 'PredictionSet[1:13]' (iter 9/10)
## INFO  [08:03:25.891] [mlr3] Applying learner 'regr.ranger' on task 'PredictionSet[1:13]' (iter 10/10)
## INFO  [08:03:25.916] [mlr3] Finished benchmark
## INFO  [08:03:25.947] [bbotk] Result of batch 9:
## INFO  [08:03:25.948] [bbotk]  max.depth num.trees  regr.rmse warnings errors runtime_learners
## INFO  [08:03:25.948] [bbotk]          3       211 0.01137342        0      0            0.135
## INFO  [08:03:25.948] [bbotk]                                 uhash
## INFO  [08:03:25.948] [bbotk]  35c3ce10-536f-4d42-b3df-c25321b1fa7c
## INFO  [08:03:25.950] [bbotk] Evaluating 1 configuration(s)
## INFO  [08:03:25.971] [mlr3] Running benchmark with 10 resampling iterations
## INFO  [08:03:25.976] [mlr3] Applying learner 'regr.ranger' on task 'PredictionSet[1:13]' (iter 1/10)
## INFO  [08:03:25.997] [mlr3] Applying learner 'regr.ranger' on task 'PredictionSet[1:13]' (iter 2/10)
## INFO  [08:03:26.017] [mlr3] Applying learner 'regr.ranger' on task 'PredictionSet[1:13]' (iter 3/10)
## INFO  [08:03:26.036] [mlr3] Applying learner 'regr.ranger' on task 'PredictionSet[1:13]' (iter 4/10)
## INFO  [08:03:26.057] [mlr3] Applying learner 'regr.ranger' on task 'PredictionSet[1:13]' (iter 5/10)
## INFO  [08:03:26.077] [mlr3] Applying learner 'regr.ranger' on task 'PredictionSet[1:13]' (iter 6/10)
## INFO  [08:03:26.096] [mlr3] Applying learner 'regr.ranger' on task 'PredictionSet[1:13]' (iter 7/10)
## INFO  [08:03:26.115] [mlr3] Applying learner 'regr.ranger' on task 'PredictionSet[1:13]' (iter 8/10)
## INFO  [08:03:26.137] [mlr3] Applying learner 'regr.ranger' on task 'PredictionSet[1:13]' (iter 9/10)
## INFO  [08:03:26.157] [mlr3] Applying learner 'regr.ranger' on task 'PredictionSet[1:13]' (iter 10/10)
## INFO  [08:03:26.176] [mlr3] Finished benchmark
## INFO  [08:03:26.208] [bbotk] Result of batch 10:
## INFO  [08:03:26.210] [bbotk]  max.depth num.trees  regr.rmse warnings errors runtime_learners
## INFO  [08:03:26.210] [bbotk]          1       211 0.01385901        0      0            0.098
## INFO  [08:03:26.210] [bbotk]                                 uhash
## INFO  [08:03:26.210] [bbotk]  37ea65c9-b7f6-4191-84ef-1731d4bde644
## INFO  [08:03:26.215] [bbotk] Finished optimizing after 10 evaluation(s)
## INFO  [08:03:26.216] [bbotk] Result:
## INFO  [08:03:26.217] [bbotk]  max.depth num.trees learner_param_vals  x_domain   regr.rmse
## INFO  [08:03:26.217] [bbotk]         20        53          <list[3]> <list[2]> 0.008983313
## INFO  [08:03:26.450] [bbotk] Starting to optimize 5 parameter(s) with '<TunerGridSearch>' and '<TerminatorEvals> [n_evals=10, k=0]'
## INFO  [08:03:26.458] [bbotk] Evaluating 1 configuration(s)
## INFO  [08:03:26.474] [mlr3] Running benchmark with 10 resampling iterations
## INFO  [08:03:26.479] [mlr3] Applying learner 'regr.svm' on task 'PredictionSet[1:13]' (iter 1/10)
## INFO  [08:03:26.494] [mlr3] Applying learner 'regr.svm' on task 'PredictionSet[1:13]' (iter 2/10)
## INFO  [08:03:26.512] [mlr3] Applying learner 'regr.svm' on task 'PredictionSet[1:13]' (iter 3/10)
## INFO  [08:03:26.535] [mlr3] Applying learner 'regr.svm' on task 'PredictionSet[1:13]' (iter 4/10)
## INFO  [08:03:26.551] [mlr3] Applying learner 'regr.svm' on task 'PredictionSet[1:13]' (iter 5/10)
## INFO  [08:03:26.567] [mlr3] Applying learner 'regr.svm' on task 'PredictionSet[1:13]' (iter 6/10)
## INFO  [08:03:26.583] [mlr3] Applying learner 'regr.svm' on task 'PredictionSet[1:13]' (iter 7/10)
## INFO  [08:03:26.600] [mlr3] Applying learner 'regr.svm' on task 'PredictionSet[1:13]' (iter 8/10)
## INFO  [08:03:26.615] [mlr3] Applying learner 'regr.svm' on task 'PredictionSet[1:13]' (iter 9/10)
## INFO  [08:03:26.631] [mlr3] Applying learner 'regr.svm' on task 'PredictionSet[1:13]' (iter 10/10)
## INFO  [08:03:26.646] [mlr3] Finished benchmark
## INFO  [08:03:26.676] [bbotk] Result of batch 1:
## INFO  [08:03:26.678] [bbotk]  tolerance gamma epsilon           type kernel  regr.rmse warnings errors
## INFO  [08:03:26.678] [bbotk]        0.9 0.001     0.1 eps-regression radial 0.05942343        0      0
## INFO  [08:03:26.678] [bbotk]  runtime_learners                                uhash
## INFO  [08:03:26.678] [bbotk]             0.066 eac5b9d6-9f1d-4035-8061-746642390fbc
## INFO  [08:03:26.679] [bbotk] Evaluating 1 configuration(s)
## INFO  [08:03:26.695] [mlr3] Running benchmark with 10 resampling iterations
## INFO  [08:03:26.700] [mlr3] Applying learner 'regr.svm' on task 'PredictionSet[1:13]' (iter 1/10)
## INFO  [08:03:26.718] [mlr3] Applying learner 'regr.svm' on task 'PredictionSet[1:13]' (iter 2/10)
## INFO  [08:03:26.738] [mlr3] Applying learner 'regr.svm' on task 'PredictionSet[1:13]' (iter 3/10)
## INFO  [08:03:26.756] [mlr3] Applying learner 'regr.svm' on task 'PredictionSet[1:13]' (iter 4/10)
## INFO  [08:03:26.774] [mlr3] Applying learner 'regr.svm' on task 'PredictionSet[1:13]' (iter 5/10)
## INFO  [08:03:26.792] [mlr3] Applying learner 'regr.svm' on task 'PredictionSet[1:13]' (iter 6/10)
## INFO  [08:03:26.811] [mlr3] Applying learner 'regr.svm' on task 'PredictionSet[1:13]' (iter 7/10)
## INFO  [08:03:26.830] [mlr3] Applying learner 'regr.svm' on task 'PredictionSet[1:13]' (iter 8/10)
## INFO  [08:03:26.848] [mlr3] Applying learner 'regr.svm' on task 'PredictionSet[1:13]' (iter 9/10)
## INFO  [08:03:26.874] [mlr3] Applying learner 'regr.svm' on task 'PredictionSet[1:13]' (iter 10/10)
## INFO  [08:03:26.894] [mlr3] Finished benchmark
## INFO  [08:03:26.924] [bbotk] Result of batch 2:
## INFO  [08:03:26.926] [bbotk]  tolerance    gamma epsilon           type  kernel regr.rmse warnings errors
## INFO  [08:03:26.926] [bbotk]  0.5147143 35.71457     0.1 eps-regression sigmoid  1.981296        0      0
## INFO  [08:03:26.926] [bbotk]  runtime_learners                                uhash
## INFO  [08:03:26.926] [bbotk]             0.087 aa0de917-5bb3-4ba9-9e40-fe73187eaeeb
## INFO  [08:03:26.928] [bbotk] Evaluating 1 configuration(s)
## INFO  [08:03:26.943] [mlr3] Running benchmark with 10 resampling iterations
## INFO  [08:03:26.948] [mlr3] Applying learner 'regr.svm' on task 'PredictionSet[1:13]' (iter 1/10)
## INFO  [08:03:26.966] [mlr3] Applying learner 'regr.svm' on task 'PredictionSet[1:13]' (iter 2/10)
## INFO  [08:03:26.981] [mlr3] Applying learner 'regr.svm' on task 'PredictionSet[1:13]' (iter 3/10)
## INFO  [08:03:26.997] [mlr3] Applying learner 'regr.svm' on task 'PredictionSet[1:13]' (iter 4/10)
## INFO  [08:03:27.012] [mlr3] Applying learner 'regr.svm' on task 'PredictionSet[1:13]' (iter 5/10)
## INFO  [08:03:27.028] [mlr3] Applying learner 'regr.svm' on task 'PredictionSet[1:13]' (iter 6/10)
## INFO  [08:03:27.045] [mlr3] Applying learner 'regr.svm' on task 'PredictionSet[1:13]' (iter 7/10)
## INFO  [08:03:27.060] [mlr3] Applying learner 'regr.svm' on task 'PredictionSet[1:13]' (iter 8/10)
## INFO  [08:03:27.076] [mlr3] Applying learner 'regr.svm' on task 'PredictionSet[1:13]' (iter 9/10)
## INFO  [08:03:27.091] [mlr3] Applying learner 'regr.svm' on task 'PredictionSet[1:13]' (iter 10/10)
## INFO  [08:03:27.107] [mlr3] Finished benchmark
## INFO  [08:03:27.139] [bbotk] Result of batch 3:
## INFO  [08:03:27.141] [bbotk]  tolerance    gamma   epsilon           type kernel  regr.rmse warnings errors
## INFO  [08:03:27.141] [bbotk]        0.9 3.572357 0.5571429 eps-regression radial 0.06299123        0      0
## INFO  [08:03:27.141] [bbotk]  runtime_learners                                uhash
## INFO  [08:03:27.141] [bbotk]             0.059 5d60eb31-c467-4c82-9fd1-a840d3c5a064
## INFO  [08:03:27.142] [bbotk] Evaluating 1 configuration(s)
## INFO  [08:03:27.158] [mlr3] Running benchmark with 10 resampling iterations
## INFO  [08:03:27.170] [mlr3] Applying learner 'regr.svm' on task 'PredictionSet[1:13]' (iter 1/10)
## INFO  [08:03:27.191] [mlr3] Applying learner 'regr.svm' on task 'PredictionSet[1:13]' (iter 2/10)
## INFO  [08:03:27.210] [mlr3] Applying learner 'regr.svm' on task 'PredictionSet[1:13]' (iter 3/10)
## INFO  [08:03:27.228] [mlr3] Applying learner 'regr.svm' on task 'PredictionSet[1:13]' (iter 4/10)
## INFO  [08:03:27.246] [mlr3] Applying learner 'regr.svm' on task 'PredictionSet[1:13]' (iter 5/10)
## INFO  [08:03:27.266] [mlr3] Applying learner 'regr.svm' on task 'PredictionSet[1:13]' (iter 6/10)
## INFO  [08:03:27.284] [mlr3] Applying learner 'regr.svm' on task 'PredictionSet[1:13]' (iter 7/10)
## INFO  [08:03:27.302] [mlr3] Applying learner 'regr.svm' on task 'PredictionSet[1:13]' (iter 8/10)
## INFO  [08:03:27.321] [mlr3] Applying learner 'regr.svm' on task 'PredictionSet[1:13]' (iter 9/10)
## INFO  [08:03:27.341] [mlr3] Applying learner 'regr.svm' on task 'PredictionSet[1:13]' (iter 10/10)
## INFO  [08:03:27.359] [mlr3] Finished benchmark
## INFO  [08:03:27.389] [bbotk] Result of batch 4:
## INFO  [08:03:27.391] [bbotk]  tolerance    gamma   epsilon           type  kernel regr.rmse warnings errors
## INFO  [08:03:27.391] [bbotk]     0.4505 21.42914 0.3285714 eps-regression sigmoid  1.962328        0      0
## INFO  [08:03:27.391] [bbotk]  runtime_learners                                uhash
## INFO  [08:03:27.391] [bbotk]             0.087 3d735fb3-1269-4fb7-b4a4-96d55053f501
## INFO  [08:03:27.392] [bbotk] Evaluating 1 configuration(s)
## INFO  [08:03:27.409] [mlr3] Running benchmark with 10 resampling iterations
## INFO  [08:03:27.415] [mlr3] Applying learner 'regr.svm' on task 'PredictionSet[1:13]' (iter 1/10)
## INFO  [08:03:27.430] [mlr3] Applying learner 'regr.svm' on task 'PredictionSet[1:13]' (iter 2/10)
## INFO  [08:03:27.446] [mlr3] Applying learner 'regr.svm' on task 'PredictionSet[1:13]' (iter 3/10)
## INFO  [08:03:27.461] [mlr3] Applying learner 'regr.svm' on task 'PredictionSet[1:13]' (iter 4/10)
## INFO  [08:03:27.477] [mlr3] Applying learner 'regr.svm' on task 'PredictionSet[1:13]' (iter 5/10)
## INFO  [08:03:27.495] [mlr3] Applying learner 'regr.svm' on task 'PredictionSet[1:13]' (iter 6/10)
## INFO  [08:03:27.518] [mlr3] Applying learner 'regr.svm' on task 'PredictionSet[1:13]' (iter 7/10)
## INFO  [08:03:27.534] [mlr3] Applying learner 'regr.svm' on task 'PredictionSet[1:13]' (iter 8/10)
## INFO  [08:03:27.550] [mlr3] Applying learner 'regr.svm' on task 'PredictionSet[1:13]' (iter 9/10)
## INFO  [08:03:27.567] [mlr3] Applying learner 'regr.svm' on task 'PredictionSet[1:13]' (iter 10/10)
## INFO  [08:03:27.583] [mlr3] Finished benchmark
## INFO  [08:03:27.613] [bbotk] Result of batch 5:
## INFO  [08:03:27.615] [bbotk]  tolerance    gamma   epsilon           type kernel  regr.rmse warnings errors
## INFO  [08:03:27.615] [bbotk]  0.5789286 46.42864 0.7857143 eps-regression radial 0.07437859        0      0
## INFO  [08:03:27.615] [bbotk]  runtime_learners                                uhash
## INFO  [08:03:27.615] [bbotk]             0.058 5f31c100-dbf1-4523-a110-913cb58e2f24
## INFO  [08:03:27.617] [bbotk] Evaluating 1 configuration(s)
## INFO  [08:03:27.633] [mlr3] Running benchmark with 10 resampling iterations
## INFO  [08:03:27.639] [mlr3] Applying learner 'regr.svm' on task 'PredictionSet[1:13]' (iter 1/10)
## INFO  [08:03:27.656] [mlr3] Applying learner 'regr.svm' on task 'PredictionSet[1:13]' (iter 2/10)
## INFO  [08:03:27.672] [mlr3] Applying learner 'regr.svm' on task 'PredictionSet[1:13]' (iter 3/10)
## INFO  [08:03:27.688] [mlr3] Applying learner 'regr.svm' on task 'PredictionSet[1:13]' (iter 4/10)
## INFO  [08:03:27.705] [mlr3] Applying learner 'regr.svm' on task 'PredictionSet[1:13]' (iter 5/10)
## INFO  [08:03:27.723] [mlr3] Applying learner 'regr.svm' on task 'PredictionSet[1:13]' (iter 6/10)
## INFO  [08:03:27.739] [mlr3] Applying learner 'regr.svm' on task 'PredictionSet[1:13]' (iter 7/10)
## INFO  [08:03:27.756] [mlr3] Applying learner 'regr.svm' on task 'PredictionSet[1:13]' (iter 8/10)
## INFO  [08:03:27.772] [mlr3] Applying learner 'regr.svm' on task 'PredictionSet[1:13]' (iter 9/10)
## INFO  [08:03:27.791] [mlr3] Applying learner 'regr.svm' on task 'PredictionSet[1:13]' (iter 10/10)
## INFO  [08:03:27.807] [mlr3] Finished benchmark
## INFO  [08:03:27.887] [bbotk] Result of batch 6:
## INFO  [08:03:27.891] [bbotk]  tolerance    gamma   epsilon           type kernel  regr.rmse warnings errors
## INFO  [08:03:27.891] [bbotk]  0.1936429 14.28643 0.6714286 eps-regression radial 0.05952064        0      0
## INFO  [08:03:27.891] [bbotk]  runtime_learners                                uhash
## INFO  [08:03:27.891] [bbotk]             0.067 3278d0ec-7a9a-4d36-bb27-7997f250012d
## INFO  [08:03:27.893] [bbotk] Evaluating 1 configuration(s)
## INFO  [08:03:27.911] [mlr3] Running benchmark with 10 resampling iterations
## INFO  [08:03:27.917] [mlr3] Applying learner 'regr.svm' on task 'PredictionSet[1:13]' (iter 1/10)
## INFO  [08:03:27.938] [mlr3] Applying learner 'regr.svm' on task 'PredictionSet[1:13]' (iter 2/10)
## INFO  [08:03:27.957] [mlr3] Applying learner 'regr.svm' on task 'PredictionSet[1:13]' (iter 3/10)
## INFO  [08:03:27.976] [mlr3] Applying learner 'regr.svm' on task 'PredictionSet[1:13]' (iter 4/10)
## INFO  [08:03:27.994] [mlr3] Applying learner 'regr.svm' on task 'PredictionSet[1:13]' (iter 5/10)
## INFO  [08:03:28.015] [mlr3] Applying learner 'regr.svm' on task 'PredictionSet[1:13]' (iter 6/10)
## INFO  [08:03:28.033] [mlr3] Applying learner 'regr.svm' on task 'PredictionSet[1:13]' (iter 7/10)
## INFO  [08:03:28.051] [mlr3] Applying learner 'regr.svm' on task 'PredictionSet[1:13]' (iter 8/10)
## INFO  [08:03:28.070] [mlr3] Applying learner 'regr.svm' on task 'PredictionSet[1:13]' (iter 9/10)
## INFO  [08:03:28.090] [mlr3] Applying learner 'regr.svm' on task 'PredictionSet[1:13]' (iter 10/10)
## INFO  [08:03:28.108] [mlr3] Finished benchmark
## INFO  [08:03:28.139] [bbotk] Result of batch 7:
## INFO  [08:03:28.141] [bbotk]  tolerance    gamma   epsilon           type  kernel regr.rmse warnings errors
## INFO  [08:03:28.141] [bbotk]  0.2578571 21.42914 0.3857143 eps-regression sigmoid  1.959482        0      0
## INFO  [08:03:28.141] [bbotk]  runtime_learners                                uhash
## INFO  [08:03:28.141] [bbotk]             0.088 63a4a412-1229-4748-a527-7a58efb89d2f
## INFO  [08:03:28.142] [bbotk] Evaluating 1 configuration(s)
## INFO  [08:03:28.160] [mlr3] Running benchmark with 10 resampling iterations
## INFO  [08:03:28.166] [mlr3] Applying learner 'regr.svm' on task 'PredictionSet[1:13]' (iter 1/10)
## INFO  [08:03:28.185] [mlr3] Applying learner 'regr.svm' on task 'PredictionSet[1:13]' (iter 2/10)
## INFO  [08:03:28.203] [mlr3] Applying learner 'regr.svm' on task 'PredictionSet[1:13]' (iter 3/10)
## INFO  [08:03:28.222] [mlr3] Applying learner 'regr.svm' on task 'PredictionSet[1:13]' (iter 4/10)
## INFO  [08:03:28.242] [mlr3] Applying learner 'regr.svm' on task 'PredictionSet[1:13]' (iter 5/10)
## INFO  [08:03:28.268] [mlr3] Applying learner 'regr.svm' on task 'PredictionSet[1:13]' (iter 6/10)
## INFO  [08:03:28.291] [mlr3] Applying learner 'regr.svm' on task 'PredictionSet[1:13]' (iter 7/10)
## INFO  [08:03:28.313] [mlr3] Applying learner 'regr.svm' on task 'PredictionSet[1:13]' (iter 8/10)
## INFO  [08:03:28.332] [mlr3] Applying learner 'regr.svm' on task 'PredictionSet[1:13]' (iter 9/10)
## INFO  [08:03:28.351] [mlr3] Applying learner 'regr.svm' on task 'PredictionSet[1:13]' (iter 10/10)
## INFO  [08:03:28.369] [mlr3] Finished benchmark
## INFO  [08:03:28.401] [bbotk] Result of batch 8:
## INFO  [08:03:28.403] [bbotk]  tolerance   gamma   epsilon           type  kernel regr.rmse warnings errors
## INFO  [08:03:28.403] [bbotk]        0.9 25.0005 0.2142857 eps-regression sigmoid  2.041518        0      0
## INFO  [08:03:28.403] [bbotk]  runtime_learners                                uhash
## INFO  [08:03:28.403] [bbotk]             0.088 798c30df-a53e-451c-8547-7258d7334d4f
## INFO  [08:03:28.405] [bbotk] Evaluating 1 configuration(s)
## INFO  [08:03:28.420] [mlr3] Running benchmark with 10 resampling iterations
## INFO  [08:03:28.425] [mlr3] Applying learner 'regr.svm' on task 'PredictionSet[1:13]' (iter 1/10)
## INFO  [08:03:28.442] [mlr3] Applying learner 'regr.svm' on task 'PredictionSet[1:13]' (iter 2/10)
## INFO  [08:03:28.459] [mlr3] Applying learner 'regr.svm' on task 'PredictionSet[1:13]' (iter 3/10)
## INFO  [08:03:28.476] [mlr3] Applying learner 'regr.svm' on task 'PredictionSet[1:13]' (iter 4/10)
## INFO  [08:03:28.492] [mlr3] Applying learner 'regr.svm' on task 'PredictionSet[1:13]' (iter 5/10)
## INFO  [08:03:28.508] [mlr3] Applying learner 'regr.svm' on task 'PredictionSet[1:13]' (iter 6/10)
## INFO  [08:03:28.525] [mlr3] Applying learner 'regr.svm' on task 'PredictionSet[1:13]' (iter 7/10)
## INFO  [08:03:28.543] [mlr3] Applying learner 'regr.svm' on task 'PredictionSet[1:13]' (iter 8/10)
## INFO  [08:03:28.559] [mlr3] Applying learner 'regr.svm' on task 'PredictionSet[1:13]' (iter 9/10)
## INFO  [08:03:28.575] [mlr3] Applying learner 'regr.svm' on task 'PredictionSet[1:13]' (iter 10/10)
## INFO  [08:03:28.591] [mlr3] Finished benchmark
## INFO  [08:03:28.624] [bbotk] Result of batch 9:
## INFO  [08:03:28.626] [bbotk]  tolerance gamma   epsilon           type kernel  regr.rmse warnings errors
## INFO  [08:03:28.626] [bbotk]  0.3862857    50 0.8428571 eps-regression radial 0.07181709        0      0
## INFO  [08:03:28.626] [bbotk]  runtime_learners                                uhash
## INFO  [08:03:28.626] [bbotk]             0.065 ec0870ed-5a17-4c1a-a5ff-afa591e1a296
## INFO  [08:03:28.627] [bbotk] Evaluating 1 configuration(s)
## INFO  [08:03:28.653] [mlr3] Running benchmark with 10 resampling iterations
## INFO  [08:03:28.659] [mlr3] Applying learner 'regr.svm' on task 'PredictionSet[1:13]' (iter 1/10)
## INFO  [08:03:28.680] [mlr3] Applying learner 'regr.svm' on task 'PredictionSet[1:13]' (iter 2/10)
## INFO  [08:03:28.701] [mlr3] Applying learner 'regr.svm' on task 'PredictionSet[1:13]' (iter 3/10)
## INFO  [08:03:28.719] [mlr3] Applying learner 'regr.svm' on task 'PredictionSet[1:13]' (iter 4/10)
## INFO  [08:03:28.738] [mlr3] Applying learner 'regr.svm' on task 'PredictionSet[1:13]' (iter 5/10)
## INFO  [08:03:28.757] [mlr3] Applying learner 'regr.svm' on task 'PredictionSet[1:13]' (iter 6/10)
## INFO  [08:03:28.777] [mlr3] Applying learner 'regr.svm' on task 'PredictionSet[1:13]' (iter 7/10)
## INFO  [08:03:28.796] [mlr3] Applying learner 'regr.svm' on task 'PredictionSet[1:13]' (iter 8/10)
## INFO  [08:03:28.814] [mlr3] Applying learner 'regr.svm' on task 'PredictionSet[1:13]' (iter 9/10)
## INFO  [08:03:28.834] [mlr3] Applying learner 'regr.svm' on task 'PredictionSet[1:13]' (iter 10/10)
## INFO  [08:03:28.853] [mlr3] Finished benchmark
## INFO  [08:03:28.884] [bbotk] Result of batch 10:
## INFO  [08:03:28.886] [bbotk]  tolerance    gamma   epsilon           type kernel  regr.rmse warnings errors
## INFO  [08:03:28.886] [bbotk]      0.001 3.572357 0.7857143 eps-regression radial 0.06118896        0      0
## INFO  [08:03:28.886] [bbotk]  runtime_learners                                uhash
## INFO  [08:03:28.886] [bbotk]             0.086 4a7ced63-3063-4816-b7d4-4a45d898a1fa
## INFO  [08:03:28.891] [bbotk] Finished optimizing after 10 evaluation(s)
## INFO  [08:03:28.891] [bbotk] Result:
## INFO  [08:03:28.892] [bbotk]  tolerance gamma epsilon           type kernel learner_param_vals  x_domain
## INFO  [08:03:28.892] [bbotk]        0.9 0.001     0.1 eps-regression radial          <list[5]> <list[5]>
## INFO  [08:03:28.892] [bbotk]   regr.rmse
## INFO  [08:03:28.892] [bbotk]  0.05942343
## INFO  [08:03:29.047] [bbotk] Starting to optimize 3 parameter(s) with '<TunerGridSearch>' and '<TerminatorEvals> [n_evals=10, k=0]'
## INFO  [08:03:29.051] [bbotk] Evaluating 1 configuration(s)
## INFO  [08:03:29.063] [mlr3] Running benchmark with 10 resampling iterations
## INFO  [08:03:29.068] [mlr3] Applying learner 'regr.kknn' on task 'PredictionSet[1:13]' (iter 1/10)
## INFO  [08:03:29.088] [mlr3] Applying learner 'regr.kknn' on task 'PredictionSet[1:13]' (iter 2/10)
## INFO  [08:03:29.109] [mlr3] Applying learner 'regr.kknn' on task 'PredictionSet[1:13]' (iter 3/10)
## INFO  [08:03:29.130] [mlr3] Applying learner 'regr.kknn' on task 'PredictionSet[1:13]' (iter 4/10)
## INFO  [08:03:29.153] [mlr3] Applying learner 'regr.kknn' on task 'PredictionSet[1:13]' (iter 5/10)
## INFO  [08:03:29.173] [mlr3] Applying learner 'regr.kknn' on task 'PredictionSet[1:13]' (iter 6/10)
## INFO  [08:03:29.193] [mlr3] Applying learner 'regr.kknn' on task 'PredictionSet[1:13]' (iter 7/10)
## INFO  [08:03:29.216] [mlr3] Applying learner 'regr.kknn' on task 'PredictionSet[1:13]' (iter 8/10)
## INFO  [08:03:29.237] [mlr3] Applying learner 'regr.kknn' on task 'PredictionSet[1:13]' (iter 9/10)
## INFO  [08:03:29.257] [mlr3] Applying learner 'regr.kknn' on task 'PredictionSet[1:13]' (iter 10/10)
## INFO  [08:03:29.278] [mlr3] Finished benchmark
## INFO  [08:03:29.308] [bbotk] Result of batch 1:
## INFO  [08:03:29.310] [bbotk]   k distance   kernel  regr.rmse warnings errors runtime_learners
## INFO  [08:03:29.310] [bbotk]  12       20 biweight 0.03335749        0      0            0.104
## INFO  [08:03:29.310] [bbotk]                                 uhash
## INFO  [08:03:29.310] [bbotk]  924aab5e-6bcc-4987-a479-f462ef1c23ed
## INFO  [08:03:29.312] [bbotk] Evaluating 1 configuration(s)
## INFO  [08:03:29.322] [mlr3] Running benchmark with 10 resampling iterations
## INFO  [08:03:29.327] [mlr3] Applying learner 'regr.kknn' on task 'PredictionSet[1:13]' (iter 1/10)
## INFO  [08:03:29.347] [mlr3] Applying learner 'regr.kknn' on task 'PredictionSet[1:13]' (iter 2/10)
## INFO  [08:03:29.380] [mlr3] Applying learner 'regr.kknn' on task 'PredictionSet[1:13]' (iter 3/10)
## INFO  [08:03:29.404] [mlr3] Applying learner 'regr.kknn' on task 'PredictionSet[1:13]' (iter 4/10)
## INFO  [08:03:29.425] [mlr3] Applying learner 'regr.kknn' on task 'PredictionSet[1:13]' (iter 5/10)
## INFO  [08:03:29.447] [mlr3] Applying learner 'regr.kknn' on task 'PredictionSet[1:13]' (iter 6/10)
## INFO  [08:03:29.468] [mlr3] Applying learner 'regr.kknn' on task 'PredictionSet[1:13]' (iter 7/10)
## INFO  [08:03:29.489] [mlr3] Applying learner 'regr.kknn' on task 'PredictionSet[1:13]' (iter 8/10)
## INFO  [08:03:29.510] [mlr3] Applying learner 'regr.kknn' on task 'PredictionSet[1:13]' (iter 9/10)
## INFO  [08:03:29.531] [mlr3] Applying learner 'regr.kknn' on task 'PredictionSet[1:13]' (iter 10/10)
## INFO  [08:03:29.551] [mlr3] Finished benchmark
## INFO  [08:03:29.583] [bbotk] Result of batch 2:
## INFO  [08:03:29.585] [bbotk]   k distance    kernel regr.rmse warnings errors runtime_learners
## INFO  [08:03:29.585] [bbotk]  19       20 triweight 0.0338515        0      0            0.105
## INFO  [08:03:29.585] [bbotk]                                 uhash
## INFO  [08:03:29.585] [bbotk]  a5a8ec01-20d5-4cfa-a99a-4ac52bc4de60
## INFO  [08:03:29.587] [bbotk] Evaluating 1 configuration(s)
## INFO  [08:03:29.598] [mlr3] Running benchmark with 10 resampling iterations
## INFO  [08:03:29.603] [mlr3] Applying learner 'regr.kknn' on task 'PredictionSet[1:13]' (iter 1/10)
## INFO  [08:03:29.624] [mlr3] Applying learner 'regr.kknn' on task 'PredictionSet[1:13]' (iter 2/10)
## INFO  [08:03:29.644] [mlr3] Applying learner 'regr.kknn' on task 'PredictionSet[1:13]' (iter 3/10)
## INFO  [08:03:29.666] [mlr3] Applying learner 'regr.kknn' on task 'PredictionSet[1:13]' (iter 4/10)
## INFO  [08:03:29.687] [mlr3] Applying learner 'regr.kknn' on task 'PredictionSet[1:13]' (iter 5/10)
## INFO  [08:03:29.707] [mlr3] Applying learner 'regr.kknn' on task 'PredictionSet[1:13]' (iter 6/10)
## INFO  [08:03:29.727] [mlr3] Applying learner 'regr.kknn' on task 'PredictionSet[1:13]' (iter 7/10)
## INFO  [08:03:29.757] [mlr3] Applying learner 'regr.kknn' on task 'PredictionSet[1:13]' (iter 8/10)
## INFO  [08:03:29.781] [mlr3] Applying learner 'regr.kknn' on task 'PredictionSet[1:13]' (iter 9/10)
## INFO  [08:03:29.803] [mlr3] Applying learner 'regr.kknn' on task 'PredictionSet[1:13]' (iter 10/10)
## INFO  [08:03:29.825] [mlr3] Finished benchmark
## INFO  [08:03:29.856] [bbotk] Result of batch 3:
## INFO  [08:03:29.858] [bbotk]  k distance kernel  regr.rmse warnings errors runtime_learners
## INFO  [08:03:29.858] [bbotk]  5       20    cos 0.03654608        0      0            0.103
## INFO  [08:03:29.858] [bbotk]                                 uhash
## INFO  [08:03:29.858] [bbotk]  7c9ec6ee-80e3-4c12-88ab-f0749f70a62c
## INFO  [08:03:29.859] [bbotk] Evaluating 1 configuration(s)
## INFO  [08:03:29.870] [mlr3] Running benchmark with 10 resampling iterations
## INFO  [08:03:29.875] [mlr3] Applying learner 'regr.kknn' on task 'PredictionSet[1:13]' (iter 1/10)
## INFO  [08:03:29.897] [mlr3] Applying learner 'regr.kknn' on task 'PredictionSet[1:13]' (iter 2/10)
## INFO  [08:03:29.918] [mlr3] Applying learner 'regr.kknn' on task 'PredictionSet[1:13]' (iter 3/10)
## INFO  [08:03:29.939] [mlr3] Applying learner 'regr.kknn' on task 'PredictionSet[1:13]' (iter 4/10)
## INFO  [08:03:29.960] [mlr3] Applying learner 'regr.kknn' on task 'PredictionSet[1:13]' (iter 5/10)
## INFO  [08:03:29.981] [mlr3] Applying learner 'regr.kknn' on task 'PredictionSet[1:13]' (iter 6/10)
## INFO  [08:03:30.002] [mlr3] Applying learner 'regr.kknn' on task 'PredictionSet[1:13]' (iter 7/10)
## INFO  [08:03:30.023] [mlr3] Applying learner 'regr.kknn' on task 'PredictionSet[1:13]' (iter 8/10)
## INFO  [08:03:30.045] [mlr3] Applying learner 'regr.kknn' on task 'PredictionSet[1:13]' (iter 9/10)
## INFO  [08:03:30.066] [mlr3] Applying learner 'regr.kknn' on task 'PredictionSet[1:13]' (iter 10/10)
## INFO  [08:03:30.086] [mlr3] Finished benchmark
## INFO  [08:03:30.119] [bbotk] Result of batch 4:
## INFO  [08:03:30.121] [bbotk]   k distance      kernel  regr.rmse warnings errors runtime_learners
## INFO  [08:03:30.121] [bbotk]  19 15.73571 rectangular 0.04185348        0      0            0.105
## INFO  [08:03:30.121] [bbotk]                                 uhash
## INFO  [08:03:30.121] [bbotk]  fb53bbf9-6f99-4907-b5bc-a9a3df54d9b1
## INFO  [08:03:30.122] [bbotk] Evaluating 1 configuration(s)
## INFO  [08:03:30.142] [mlr3] Running benchmark with 10 resampling iterations
## INFO  [08:03:30.148] [mlr3] Applying learner 'regr.kknn' on task 'PredictionSet[1:13]' (iter 1/10)
## INFO  [08:03:30.170] [mlr3] Applying learner 'regr.kknn' on task 'PredictionSet[1:13]' (iter 2/10)
## INFO  [08:03:30.192] [mlr3] Applying learner 'regr.kknn' on task 'PredictionSet[1:13]' (iter 3/10)
## INFO  [08:03:30.213] [mlr3] Applying learner 'regr.kknn' on task 'PredictionSet[1:13]' (iter 4/10)
## INFO  [08:03:30.233] [mlr3] Applying learner 'regr.kknn' on task 'PredictionSet[1:13]' (iter 5/10)
## INFO  [08:03:30.253] [mlr3] Applying learner 'regr.kknn' on task 'PredictionSet[1:13]' (iter 6/10)
## INFO  [08:03:30.275] [mlr3] Applying learner 'regr.kknn' on task 'PredictionSet[1:13]' (iter 7/10)
## INFO  [08:03:30.295] [mlr3] Applying learner 'regr.kknn' on task 'PredictionSet[1:13]' (iter 8/10)
## INFO  [08:03:30.315] [mlr3] Applying learner 'regr.kknn' on task 'PredictionSet[1:13]' (iter 9/10)
## INFO  [08:03:30.337] [mlr3] Applying learner 'regr.kknn' on task 'PredictionSet[1:13]' (iter 10/10)
## INFO  [08:03:30.357] [mlr3] Finished benchmark
## INFO  [08:03:30.389] [bbotk] Result of batch 5:
## INFO  [08:03:30.390] [bbotk]  k distance  kernel  regr.rmse warnings errors runtime_learners
## INFO  [08:03:30.390] [bbotk]  1 4.364286 optimal 0.03763125        0      0            0.105
## INFO  [08:03:30.390] [bbotk]                                 uhash
## INFO  [08:03:30.390] [bbotk]  e8a0bd14-3c3a-4ccc-b60a-924a5a2fda87
## INFO  [08:03:30.392] [bbotk] Evaluating 1 configuration(s)
## INFO  [08:03:30.403] [mlr3] Running benchmark with 10 resampling iterations
## INFO  [08:03:30.409] [mlr3] Applying learner 'regr.kknn' on task 'PredictionSet[1:13]' (iter 1/10)
## INFO  [08:03:30.430] [mlr3] Applying learner 'regr.kknn' on task 'PredictionSet[1:13]' (iter 2/10)
## INFO  [08:03:30.451] [mlr3] Applying learner 'regr.kknn' on task 'PredictionSet[1:13]' (iter 3/10)
## INFO  [08:03:30.471] [mlr3] Applying learner 'regr.kknn' on task 'PredictionSet[1:13]' (iter 4/10)
## INFO  [08:03:30.493] [mlr3] Applying learner 'regr.kknn' on task 'PredictionSet[1:13]' (iter 5/10)
## INFO  [08:03:30.523] [mlr3] Applying learner 'regr.kknn' on task 'PredictionSet[1:13]' (iter 6/10)
## INFO  [08:03:30.547] [mlr3] Applying learner 'regr.kknn' on task 'PredictionSet[1:13]' (iter 7/10)
## INFO  [08:03:30.570] [mlr3] Applying learner 'regr.kknn' on task 'PredictionSet[1:13]' (iter 8/10)
## INFO  [08:03:30.590] [mlr3] Applying learner 'regr.kknn' on task 'PredictionSet[1:13]' (iter 9/10)
## INFO  [08:03:30.611] [mlr3] Applying learner 'regr.kknn' on task 'PredictionSet[1:13]' (iter 10/10)
## INFO  [08:03:30.631] [mlr3] Finished benchmark
## INFO  [08:03:30.664] [bbotk] Result of batch 6:
## INFO  [08:03:30.666] [bbotk]   k distance   kernel  regr.rmse warnings errors runtime_learners
## INFO  [08:03:30.666] [bbotk]  15 18.57857 biweight 0.03325726        0      0            0.113
## INFO  [08:03:30.666] [bbotk]                                 uhash
## INFO  [08:03:30.666] [bbotk]  4804fdd9-0408-453b-b5ea-6ce024a82720
## INFO  [08:03:30.667] [bbotk] Evaluating 1 configuration(s)
## INFO  [08:03:30.678] [mlr3] Running benchmark with 10 resampling iterations
## INFO  [08:03:30.683] [mlr3] Applying learner 'regr.kknn' on task 'PredictionSet[1:13]' (iter 1/10)
## INFO  [08:03:30.703] [mlr3] Applying learner 'regr.kknn' on task 'PredictionSet[1:13]' (iter 2/10)
## INFO  [08:03:30.725] [mlr3] Applying learner 'regr.kknn' on task 'PredictionSet[1:13]' (iter 3/10)
## INFO  [08:03:30.746] [mlr3] Applying learner 'regr.kknn' on task 'PredictionSet[1:13]' (iter 4/10)
## INFO  [08:03:30.767] [mlr3] Applying learner 'regr.kknn' on task 'PredictionSet[1:13]' (iter 5/10)
## INFO  [08:03:30.789] [mlr3] Applying learner 'regr.kknn' on task 'PredictionSet[1:13]' (iter 6/10)
## INFO  [08:03:30.810] [mlr3] Applying learner 'regr.kknn' on task 'PredictionSet[1:13]' (iter 7/10)
## INFO  [08:03:30.831] [mlr3] Applying learner 'regr.kknn' on task 'PredictionSet[1:13]' (iter 8/10)
## INFO  [08:03:30.852] [mlr3] Applying learner 'regr.kknn' on task 'PredictionSet[1:13]' (iter 9/10)
## INFO  [08:03:30.874] [mlr3] Applying learner 'regr.kknn' on task 'PredictionSet[1:13]' (iter 10/10)
## INFO  [08:03:30.904] [mlr3] Finished benchmark
## INFO  [08:03:30.941] [bbotk] Result of batch 7:
## INFO  [08:03:30.943] [bbotk]  k distance kernel  regr.rmse warnings errors runtime_learners
## INFO  [08:03:30.943] [bbotk]  9 14.31429   rank 0.03445547        0      0            0.117
## INFO  [08:03:30.943] [bbotk]                                 uhash
## INFO  [08:03:30.943] [bbotk]  a2221bf9-6f71-4f27-aaf1-90d664b28f61
## INFO  [08:03:30.944] [bbotk] Evaluating 1 configuration(s)
## INFO  [08:03:30.955] [mlr3] Running benchmark with 10 resampling iterations
## INFO  [08:03:30.961] [mlr3] Applying learner 'regr.kknn' on task 'PredictionSet[1:13]' (iter 1/10)
## INFO  [08:03:30.981] [mlr3] Applying learner 'regr.kknn' on task 'PredictionSet[1:13]' (iter 2/10)
## INFO  [08:03:31.001] [mlr3] Applying learner 'regr.kknn' on task 'PredictionSet[1:13]' (iter 3/10)
## INFO  [08:03:31.024] [mlr3] Applying learner 'regr.kknn' on task 'PredictionSet[1:13]' (iter 4/10)
## INFO  [08:03:31.044] [mlr3] Applying learner 'regr.kknn' on task 'PredictionSet[1:13]' (iter 5/10)
## INFO  [08:03:31.065] [mlr3] Applying learner 'regr.kknn' on task 'PredictionSet[1:13]' (iter 6/10)
## INFO  [08:03:31.086] [mlr3] Applying learner 'regr.kknn' on task 'PredictionSet[1:13]' (iter 7/10)
## INFO  [08:03:31.107] [mlr3] Applying learner 'regr.kknn' on task 'PredictionSet[1:13]' (iter 8/10)
## INFO  [08:03:31.128] [mlr3] Applying learner 'regr.kknn' on task 'PredictionSet[1:13]' (iter 9/10)
## INFO  [08:03:31.148] [mlr3] Applying learner 'regr.kknn' on task 'PredictionSet[1:13]' (iter 10/10)
## INFO  [08:03:31.170] [mlr3] Finished benchmark
## INFO  [08:03:31.203] [bbotk] Result of batch 8:
## INFO  [08:03:31.204] [bbotk]   k distance kernel  regr.rmse warnings errors runtime_learners
## INFO  [08:03:31.204] [bbotk]  12 1.521429    inv 0.02929553        0      0            0.101
## INFO  [08:03:31.204] [bbotk]                                 uhash
## INFO  [08:03:31.204] [bbotk]  c211ea53-ad0b-4fd5-a412-34956362adf5
## INFO  [08:03:31.206] [bbotk] Evaluating 1 configuration(s)
## INFO  [08:03:31.216] [mlr3] Running benchmark with 10 resampling iterations
## INFO  [08:03:31.222] [mlr3] Applying learner 'regr.kknn' on task 'PredictionSet[1:13]' (iter 1/10)
## INFO  [08:03:31.243] [mlr3] Applying learner 'regr.kknn' on task 'PredictionSet[1:13]' (iter 2/10)
## INFO  [08:03:31.273] [mlr3] Applying learner 'regr.kknn' on task 'PredictionSet[1:13]' (iter 3/10)
## INFO  [08:03:31.297] [mlr3] Applying learner 'regr.kknn' on task 'PredictionSet[1:13]' (iter 4/10)
## INFO  [08:03:31.320] [mlr3] Applying learner 'regr.kknn' on task 'PredictionSet[1:13]' (iter 5/10)
## INFO  [08:03:31.341] [mlr3] Applying learner 'regr.kknn' on task 'PredictionSet[1:13]' (iter 6/10)
## INFO  [08:03:31.361] [mlr3] Applying learner 'regr.kknn' on task 'PredictionSet[1:13]' (iter 7/10)
## INFO  [08:03:31.383] [mlr3] Applying learner 'regr.kknn' on task 'PredictionSet[1:13]' (iter 8/10)
## INFO  [08:03:31.404] [mlr3] Applying learner 'regr.kknn' on task 'PredictionSet[1:13]' (iter 9/10)
## INFO  [08:03:31.425] [mlr3] Applying learner 'regr.kknn' on task 'PredictionSet[1:13]' (iter 10/10)
## INFO  [08:03:31.445] [mlr3] Finished benchmark
## INFO  [08:03:31.478] [bbotk] Result of batch 9:
## INFO  [08:03:31.480] [bbotk]   k distance    kernel  regr.rmse warnings errors runtime_learners
## INFO  [08:03:31.480] [bbotk]  16 4.364286 triweight 0.03230064        0      0            0.112
## INFO  [08:03:31.480] [bbotk]                                 uhash
## INFO  [08:03:31.480] [bbotk]  9c624ecf-54c6-4ea6-a493-55fd4e646e31
## INFO  [08:03:31.481] [bbotk] Evaluating 1 configuration(s)
## INFO  [08:03:31.492] [mlr3] Running benchmark with 10 resampling iterations
## INFO  [08:03:31.497] [mlr3] Applying learner 'regr.kknn' on task 'PredictionSet[1:13]' (iter 1/10)
## INFO  [08:03:31.517] [mlr3] Applying learner 'regr.kknn' on task 'PredictionSet[1:13]' (iter 2/10)
## INFO  [08:03:31.540] [mlr3] Applying learner 'regr.kknn' on task 'PredictionSet[1:13]' (iter 3/10)
## INFO  [08:03:31.561] [mlr3] Applying learner 'regr.kknn' on task 'PredictionSet[1:13]' (iter 4/10)
## INFO  [08:03:31.581] [mlr3] Applying learner 'regr.kknn' on task 'PredictionSet[1:13]' (iter 5/10)
## INFO  [08:03:31.602] [mlr3] Applying learner 'regr.kknn' on task 'PredictionSet[1:13]' (iter 6/10)
## INFO  [08:03:31.625] [mlr3] Applying learner 'regr.kknn' on task 'PredictionSet[1:13]' (iter 7/10)
## INFO  [08:03:31.654] [mlr3] Applying learner 'regr.kknn' on task 'PredictionSet[1:13]' (iter 8/10)
## INFO  [08:03:31.679] [mlr3] Applying learner 'regr.kknn' on task 'PredictionSet[1:13]' (iter 9/10)
## INFO  [08:03:31.701] [mlr3] Applying learner 'regr.kknn' on task 'PredictionSet[1:13]' (iter 10/10)
## INFO  [08:03:31.722] [mlr3] Finished benchmark
## INFO  [08:03:31.754] [bbotk] Result of batch 10:
## INFO  [08:03:31.755] [bbotk]   k distance    kernel  regr.rmse warnings errors runtime_learners
## INFO  [08:03:31.755] [bbotk]  16 15.73571 triweight 0.03330121        0      0            0.117
## INFO  [08:03:31.755] [bbotk]                                 uhash
## INFO  [08:03:31.755] [bbotk]  d9760fef-12cb-474d-8cb6-97d4f35e32d9
## INFO  [08:03:31.761] [bbotk] Finished optimizing after 10 evaluation(s)
## INFO  [08:03:31.762] [bbotk] Result:
## INFO  [08:03:31.764] [bbotk]   k distance kernel learner_param_vals  x_domain  regr.rmse
## INFO  [08:03:31.764] [bbotk]  12 1.521429    inv          <list[3]> <list[3]> 0.02929553
## INFO  [08:03:31.987] [bbotk] Starting to optimize 2 parameter(s) with '<TunerGridSearch>' and '<TerminatorEvals> [n_evals=10, k=0]'
## INFO  [08:03:31.990] [bbotk] Evaluating 1 configuration(s)
## INFO  [08:03:32.013] [mlr3] Running benchmark with 10 resampling iterations
## INFO  [08:03:32.018] [mlr3] Applying learner 'regr.ranger' on task 'PredictionSet[1:13]' (iter 1/10)
## INFO  [08:03:32.033] [mlr3] Applying learner 'regr.ranger' on task 'PredictionSet[1:13]' (iter 2/10)
## INFO  [08:03:32.048] [mlr3] Applying learner 'regr.ranger' on task 'PredictionSet[1:13]' (iter 3/10)
## INFO  [08:03:32.065] [mlr3] Applying learner 'regr.ranger' on task 'PredictionSet[1:13]' (iter 4/10)
## INFO  [08:03:32.080] [mlr3] Applying learner 'regr.ranger' on task 'PredictionSet[1:13]' (iter 5/10)
## INFO  [08:03:32.095] [mlr3] Applying learner 'regr.ranger' on task 'PredictionSet[1:13]' (iter 6/10)
## INFO  [08:03:32.110] [mlr3] Applying learner 'regr.ranger' on task 'PredictionSet[1:13]' (iter 7/10)
## INFO  [08:03:32.125] [mlr3] Applying learner 'regr.ranger' on task 'PredictionSet[1:13]' (iter 8/10)
## INFO  [08:03:32.142] [mlr3] Applying learner 'regr.ranger' on task 'PredictionSet[1:13]' (iter 9/10)
## INFO  [08:03:32.157] [mlr3] Applying learner 'regr.ranger' on task 'PredictionSet[1:13]' (iter 10/10)
## INFO  [08:03:32.172] [mlr3] Finished benchmark
## INFO  [08:03:32.201] [bbotk] Result of batch 1:
## INFO  [08:03:32.203] [bbotk]  max.depth num.trees  regr.rmse warnings errors runtime_learners
## INFO  [08:03:32.203] [bbotk]          7         1 0.05209372        0      0            0.048
## INFO  [08:03:32.203] [bbotk]                                 uhash
## INFO  [08:03:32.203] [bbotk]  9d0439d5-5f72-4b27-8be8-2730211e23ec
## INFO  [08:03:32.205] [bbotk] Evaluating 1 configuration(s)
## INFO  [08:03:32.228] [mlr3] Running benchmark with 10 resampling iterations
## INFO  [08:03:32.240] [mlr3] Applying learner 'regr.ranger' on task 'PredictionSet[1:13]' (iter 1/10)
## INFO  [08:03:32.258] [mlr3] Applying learner 'regr.ranger' on task 'PredictionSet[1:13]' (iter 2/10)
## INFO  [08:03:32.275] [mlr3] Applying learner 'regr.ranger' on task 'PredictionSet[1:13]' (iter 3/10)
## INFO  [08:03:32.292] [mlr3] Applying learner 'regr.ranger' on task 'PredictionSet[1:13]' (iter 4/10)
## INFO  [08:03:32.308] [mlr3] Applying learner 'regr.ranger' on task 'PredictionSet[1:13]' (iter 5/10)
## INFO  [08:03:32.323] [mlr3] Applying learner 'regr.ranger' on task 'PredictionSet[1:13]' (iter 6/10)
## INFO  [08:03:32.338] [mlr3] Applying learner 'regr.ranger' on task 'PredictionSet[1:13]' (iter 7/10)
## INFO  [08:03:32.353] [mlr3] Applying learner 'regr.ranger' on task 'PredictionSet[1:13]' (iter 8/10)
## INFO  [08:03:32.370] [mlr3] Applying learner 'regr.ranger' on task 'PredictionSet[1:13]' (iter 9/10)
## INFO  [08:03:32.384] [mlr3] Applying learner 'regr.ranger' on task 'PredictionSet[1:13]' (iter 10/10)
## INFO  [08:03:32.399] [mlr3] Finished benchmark
## INFO  [08:03:32.430] [bbotk] Result of batch 2:
## INFO  [08:03:32.432] [bbotk]  max.depth num.trees  regr.rmse warnings errors runtime_learners
## INFO  [08:03:32.432] [bbotk]         16         1 0.04276241        0      0            0.052
## INFO  [08:03:32.432] [bbotk]                                 uhash
## INFO  [08:03:32.432] [bbotk]  90052612-af11-49a4-aab4-c3eacd1996c1
## INFO  [08:03:32.434] [bbotk] Evaluating 1 configuration(s)
## INFO  [08:03:32.457] [mlr3] Running benchmark with 10 resampling iterations
## INFO  [08:03:32.462] [mlr3] Applying learner 'regr.ranger' on task 'PredictionSet[1:13]' (iter 1/10)
## INFO  [08:03:32.496] [mlr3] Applying learner 'regr.ranger' on task 'PredictionSet[1:13]' (iter 2/10)
## INFO  [08:03:32.530] [mlr3] Applying learner 'regr.ranger' on task 'PredictionSet[1:13]' (iter 3/10)
## INFO  [08:03:32.565] [mlr3] Applying learner 'regr.ranger' on task 'PredictionSet[1:13]' (iter 4/10)
## INFO  [08:03:32.600] [mlr3] Applying learner 'regr.ranger' on task 'PredictionSet[1:13]' (iter 5/10)
## INFO  [08:03:32.632] [mlr3] Applying learner 'regr.ranger' on task 'PredictionSet[1:13]' (iter 6/10)
## INFO  [08:03:32.676] [mlr3] Applying learner 'regr.ranger' on task 'PredictionSet[1:13]' (iter 7/10)
## INFO  [08:03:32.713] [mlr3] Applying learner 'regr.ranger' on task 'PredictionSet[1:13]' (iter 8/10)
## INFO  [08:03:32.750] [mlr3] Applying learner 'regr.ranger' on task 'PredictionSet[1:13]' (iter 9/10)
## INFO  [08:03:32.783] [mlr3] Applying learner 'regr.ranger' on task 'PredictionSet[1:13]' (iter 10/10)
## INFO  [08:03:32.817] [mlr3] Finished benchmark
## INFO  [08:03:32.848] [bbotk] Result of batch 3:
## INFO  [08:03:32.850] [bbotk]  max.depth num.trees regr.rmse warnings errors runtime_learners
## INFO  [08:03:32.850] [bbotk]         19       264  0.032653        0      0            0.236
## INFO  [08:03:32.850] [bbotk]                                 uhash
## INFO  [08:03:32.850] [bbotk]  0e7b14c5-fcbd-4864-b535-b4c6d6ce9b60
## INFO  [08:03:32.852] [bbotk] Evaluating 1 configuration(s)
## INFO  [08:03:32.873] [mlr3] Running benchmark with 10 resampling iterations
## INFO  [08:03:32.878] [mlr3] Applying learner 'regr.ranger' on task 'PredictionSet[1:13]' (iter 1/10)
## INFO  [08:03:32.929] [mlr3] Applying learner 'regr.ranger' on task 'PredictionSet[1:13]' (iter 2/10)
## INFO  [08:03:32.979] [mlr3] Applying learner 'regr.ranger' on task 'PredictionSet[1:13]' (iter 3/10)
## INFO  [08:03:33.027] [mlr3] Applying learner 'regr.ranger' on task 'PredictionSet[1:13]' (iter 4/10)
## INFO  [08:03:33.076] [mlr3] Applying learner 'regr.ranger' on task 'PredictionSet[1:13]' (iter 5/10)
## INFO  [08:03:33.126] [mlr3] Applying learner 'regr.ranger' on task 'PredictionSet[1:13]' (iter 6/10)
## INFO  [08:03:33.175] [mlr3] Applying learner 'regr.ranger' on task 'PredictionSet[1:13]' (iter 7/10)
## INFO  [08:03:33.225] [mlr3] Applying learner 'regr.ranger' on task 'PredictionSet[1:13]' (iter 8/10)
## INFO  [08:03:33.275] [mlr3] Applying learner 'regr.ranger' on task 'PredictionSet[1:13]' (iter 9/10)
## INFO  [08:03:33.323] [mlr3] Applying learner 'regr.ranger' on task 'PredictionSet[1:13]' (iter 10/10)
## INFO  [08:03:33.373] [mlr3] Finished benchmark
## INFO  [08:03:33.418] [bbotk] Result of batch 4:
## INFO  [08:03:33.420] [bbotk]  max.depth num.trees  regr.rmse warnings errors runtime_learners
## INFO  [08:03:33.420] [bbotk]         12       500 0.03304637        0      0            0.388
## INFO  [08:03:33.420] [bbotk]                                 uhash
## INFO  [08:03:33.420] [bbotk]  57ca7bbd-67c0-4564-880d-d146f463876b
## INFO  [08:03:33.422] [bbotk] Evaluating 1 configuration(s)
## INFO  [08:03:33.446] [mlr3] Running benchmark with 10 resampling iterations
## INFO  [08:03:33.451] [mlr3] Applying learner 'regr.ranger' on task 'PredictionSet[1:13]' (iter 1/10)
## INFO  [08:03:33.480] [mlr3] Applying learner 'regr.ranger' on task 'PredictionSet[1:13]' (iter 2/10)
## INFO  [08:03:33.509] [mlr3] Applying learner 'regr.ranger' on task 'PredictionSet[1:13]' (iter 3/10)
## INFO  [08:03:33.536] [mlr3] Applying learner 'regr.ranger' on task 'PredictionSet[1:13]' (iter 4/10)
## INFO  [08:03:33.566] [mlr3] Applying learner 'regr.ranger' on task 'PredictionSet[1:13]' (iter 5/10)
## INFO  [08:03:33.594] [mlr3] Applying learner 'regr.ranger' on task 'PredictionSet[1:13]' (iter 6/10)
## INFO  [08:03:33.621] [mlr3] Applying learner 'regr.ranger' on task 'PredictionSet[1:13]' (iter 7/10)
## INFO  [08:03:33.650] [mlr3] Applying learner 'regr.ranger' on task 'PredictionSet[1:13]' (iter 8/10)
## INFO  [08:03:33.677] [mlr3] Applying learner 'regr.ranger' on task 'PredictionSet[1:13]' (iter 9/10)
## INFO  [08:03:33.706] [mlr3] Applying learner 'regr.ranger' on task 'PredictionSet[1:13]' (iter 10/10)
## INFO  [08:03:33.734] [mlr3] Finished benchmark
## INFO  [08:03:33.765] [bbotk] Result of batch 5:
## INFO  [08:03:33.767] [bbotk]  max.depth num.trees  regr.rmse warnings errors runtime_learners
## INFO  [08:03:33.767] [bbotk]          9       185 0.03244653        0      0            0.176
## INFO  [08:03:33.767] [bbotk]                                 uhash
## INFO  [08:03:33.767] [bbotk]  bc37e73a-e1c3-4233-976b-800dedf452b3
## INFO  [08:03:33.769] [bbotk] Evaluating 1 configuration(s)
## INFO  [08:03:33.792] [mlr3] Running benchmark with 10 resampling iterations
## INFO  [08:03:33.797] [mlr3] Applying learner 'regr.ranger' on task 'PredictionSet[1:13]' (iter 1/10)
## INFO  [08:03:33.841] [mlr3] Applying learner 'regr.ranger' on task 'PredictionSet[1:13]' (iter 2/10)
## INFO  [08:03:33.895] [mlr3] Applying learner 'regr.ranger' on task 'PredictionSet[1:13]' (iter 3/10)
## INFO  [08:03:33.947] [mlr3] Applying learner 'regr.ranger' on task 'PredictionSet[1:13]' (iter 4/10)
## INFO  [08:03:33.995] [mlr3] Applying learner 'regr.ranger' on task 'PredictionSet[1:13]' (iter 5/10)
## INFO  [08:03:34.042] [mlr3] Applying learner 'regr.ranger' on task 'PredictionSet[1:13]' (iter 6/10)
## INFO  [08:03:34.089] [mlr3] Applying learner 'regr.ranger' on task 'PredictionSet[1:13]' (iter 7/10)
## INFO  [08:03:34.135] [mlr3] Applying learner 'regr.ranger' on task 'PredictionSet[1:13]' (iter 8/10)
## INFO  [08:03:34.181] [mlr3] Applying learner 'regr.ranger' on task 'PredictionSet[1:13]' (iter 9/10)
## INFO  [08:03:34.226] [mlr3] Applying learner 'regr.ranger' on task 'PredictionSet[1:13]' (iter 10/10)
## INFO  [08:03:34.272] [mlr3] Finished benchmark
## INFO  [08:03:34.303] [bbotk] Result of batch 6:
## INFO  [08:03:34.305] [bbotk]  max.depth num.trees  regr.rmse warnings errors runtime_learners
## INFO  [08:03:34.305] [bbotk]         19       448 0.03289853        0      0            0.357
## INFO  [08:03:34.305] [bbotk]                                 uhash
## INFO  [08:03:34.305] [bbotk]  7abdf280-7919-45da-8c51-a07ba7b6d7a1
## INFO  [08:03:34.307] [bbotk] Evaluating 1 configuration(s)
## INFO  [08:03:34.329] [mlr3] Running benchmark with 10 resampling iterations
## INFO  [08:03:34.334] [mlr3] Applying learner 'regr.ranger' on task 'PredictionSet[1:13]' (iter 1/10)
## INFO  [08:03:34.362] [mlr3] Applying learner 'regr.ranger' on task 'PredictionSet[1:13]' (iter 2/10)
## INFO  [08:03:34.391] [mlr3] Applying learner 'regr.ranger' on task 'PredictionSet[1:13]' (iter 3/10)
## INFO  [08:03:34.419] [mlr3] Applying learner 'regr.ranger' on task 'PredictionSet[1:13]' (iter 4/10)
## INFO  [08:03:34.446] [mlr3] Applying learner 'regr.ranger' on task 'PredictionSet[1:13]' (iter 5/10)
## INFO  [08:03:34.475] [mlr3] Applying learner 'regr.ranger' on task 'PredictionSet[1:13]' (iter 6/10)
## INFO  [08:03:34.502] [mlr3] Applying learner 'regr.ranger' on task 'PredictionSet[1:13]' (iter 7/10)
## INFO  [08:03:34.530] [mlr3] Applying learner 'regr.ranger' on task 'PredictionSet[1:13]' (iter 8/10)
## INFO  [08:03:34.571] [mlr3] Applying learner 'regr.ranger' on task 'PredictionSet[1:13]' (iter 9/10)
## INFO  [08:03:34.602] [mlr3] Applying learner 'regr.ranger' on task 'PredictionSet[1:13]' (iter 10/10)
## INFO  [08:03:34.632] [mlr3] Finished benchmark
## INFO  [08:03:34.663] [bbotk] Result of batch 7:
## INFO  [08:03:34.665] [bbotk]  max.depth num.trees  regr.rmse warnings errors runtime_learners
## INFO  [08:03:34.665] [bbotk]         19       185 0.03270799        0      0            0.187
## INFO  [08:03:34.665] [bbotk]                                 uhash
## INFO  [08:03:34.665] [bbotk]  2f8ab374-1cfb-4144-b33e-3df4b18a9ad0
## INFO  [08:03:34.666] [bbotk] Evaluating 1 configuration(s)
## INFO  [08:03:34.689] [mlr3] Running benchmark with 10 resampling iterations
## INFO  [08:03:34.695] [mlr3] Applying learner 'regr.ranger' on task 'PredictionSet[1:13]' (iter 1/10)
## INFO  [08:03:34.721] [mlr3] Applying learner 'regr.ranger' on task 'PredictionSet[1:13]' (iter 2/10)
## INFO  [08:03:34.748] [mlr3] Applying learner 'regr.ranger' on task 'PredictionSet[1:13]' (iter 3/10)
## INFO  [08:03:34.776] [mlr3] Applying learner 'regr.ranger' on task 'PredictionSet[1:13]' (iter 4/10)
## INFO  [08:03:34.802] [mlr3] Applying learner 'regr.ranger' on task 'PredictionSet[1:13]' (iter 5/10)
## INFO  [08:03:34.829] [mlr3] Applying learner 'regr.ranger' on task 'PredictionSet[1:13]' (iter 6/10)
## INFO  [08:03:34.857] [mlr3] Applying learner 'regr.ranger' on task 'PredictionSet[1:13]' (iter 7/10)
## INFO  [08:03:34.884] [mlr3] Applying learner 'regr.ranger' on task 'PredictionSet[1:13]' (iter 8/10)
## INFO  [08:03:34.913] [mlr3] Applying learner 'regr.ranger' on task 'PredictionSet[1:13]' (iter 9/10)
## INFO  [08:03:34.941] [mlr3] Applying learner 'regr.ranger' on task 'PredictionSet[1:13]' (iter 10/10)
## INFO  [08:03:34.967] [mlr3] Finished benchmark
## INFO  [08:03:35.000] [bbotk] Result of batch 8:
## INFO  [08:03:35.002] [bbotk]  max.depth num.trees  regr.rmse warnings errors runtime_learners
## INFO  [08:03:35.002] [bbotk]          2       395 0.05018765        0      0            0.169
## INFO  [08:03:35.002] [bbotk]                                 uhash
## INFO  [08:03:35.002] [bbotk]  7d096b6b-29fc-4439-a15d-ea1c848d3c4b
## INFO  [08:03:35.003] [bbotk] Evaluating 1 configuration(s)
## INFO  [08:03:35.037] [mlr3] Running benchmark with 10 resampling iterations
## INFO  [08:03:35.042] [mlr3] Applying learner 'regr.ranger' on task 'PredictionSet[1:13]' (iter 1/10)
## INFO  [08:03:35.061] [mlr3] Applying learner 'regr.ranger' on task 'PredictionSet[1:13]' (iter 2/10)
## INFO  [08:03:35.078] [mlr3] Applying learner 'regr.ranger' on task 'PredictionSet[1:13]' (iter 3/10)
## INFO  [08:03:35.095] [mlr3] Applying learner 'regr.ranger' on task 'PredictionSet[1:13]' (iter 4/10)
## INFO  [08:03:35.111] [mlr3] Applying learner 'regr.ranger' on task 'PredictionSet[1:13]' (iter 5/10)
## INFO  [08:03:35.128] [mlr3] Applying learner 'regr.ranger' on task 'PredictionSet[1:13]' (iter 6/10)
## INFO  [08:03:35.146] [mlr3] Applying learner 'regr.ranger' on task 'PredictionSet[1:13]' (iter 7/10)
## INFO  [08:03:35.162] [mlr3] Applying learner 'regr.ranger' on task 'PredictionSet[1:13]' (iter 8/10)
## INFO  [08:03:35.178] [mlr3] Applying learner 'regr.ranger' on task 'PredictionSet[1:13]' (iter 9/10)
## INFO  [08:03:35.195] [mlr3] Applying learner 'regr.ranger' on task 'PredictionSet[1:13]' (iter 10/10)
## INFO  [08:03:35.213] [mlr3] Finished benchmark
## INFO  [08:03:35.244] [bbotk] Result of batch 9:
## INFO  [08:03:35.246] [bbotk]  max.depth num.trees  regr.rmse warnings errors runtime_learners
## INFO  [08:03:35.246] [bbotk]          5        27 0.03611823        0      0            0.065
## INFO  [08:03:35.246] [bbotk]                                 uhash
## INFO  [08:03:35.246] [bbotk]  8e4d0a36-590d-4350-8f5b-f9f0293018be
## INFO  [08:03:35.247] [bbotk] Evaluating 1 configuration(s)
## INFO  [08:03:35.269] [mlr3] Running benchmark with 10 resampling iterations
## INFO  [08:03:35.275] [mlr3] Applying learner 'regr.ranger' on task 'PredictionSet[1:13]' (iter 1/10)
## INFO  [08:03:35.313] [mlr3] Applying learner 'regr.ranger' on task 'PredictionSet[1:13]' (iter 2/10)
## INFO  [08:03:35.350] [mlr3] Applying learner 'regr.ranger' on task 'PredictionSet[1:13]' (iter 3/10)
## INFO  [08:03:35.397] [mlr3] Applying learner 'regr.ranger' on task 'PredictionSet[1:13]' (iter 4/10)
## INFO  [08:03:35.441] [mlr3] Applying learner 'regr.ranger' on task 'PredictionSet[1:13]' (iter 5/10)
## INFO  [08:03:35.480] [mlr3] Applying learner 'regr.ranger' on task 'PredictionSet[1:13]' (iter 6/10)
## INFO  [08:03:35.520] [mlr3] Applying learner 'regr.ranger' on task 'PredictionSet[1:13]' (iter 7/10)
## INFO  [08:03:35.558] [mlr3] Applying learner 'regr.ranger' on task 'PredictionSet[1:13]' (iter 8/10)
## INFO  [08:03:35.597] [mlr3] Applying learner 'regr.ranger' on task 'PredictionSet[1:13]' (iter 9/10)
## INFO  [08:03:35.634] [mlr3] Applying learner 'regr.ranger' on task 'PredictionSet[1:13]' (iter 10/10)
## INFO  [08:03:35.673] [mlr3] Finished benchmark
## INFO  [08:03:35.704] [bbotk] Result of batch 10:
## INFO  [08:03:35.705] [bbotk]  max.depth num.trees  regr.rmse warnings errors runtime_learners
## INFO  [08:03:35.705] [bbotk]          4       500 0.03932736        0      0             0.28
## INFO  [08:03:35.705] [bbotk]                                 uhash
## INFO  [08:03:35.705] [bbotk]  3e60957a-df3c-4689-9752-d566f4e5bc98
## INFO  [08:03:35.710] [bbotk] Finished optimizing after 10 evaluation(s)
## INFO  [08:03:35.711] [bbotk] Result:
## INFO  [08:03:35.712] [bbotk]  max.depth num.trees learner_param_vals  x_domain  regr.rmse
## INFO  [08:03:35.712] [bbotk]          9       185          <list[3]> <list[2]> 0.03244653
## INFO  [08:03:35.958] [bbotk] Starting to optimize 5 parameter(s) with '<TunerGridSearch>' and '<TerminatorEvals> [n_evals=10, k=0]'
## INFO  [08:03:35.965] [bbotk] Evaluating 1 configuration(s)
## INFO  [08:03:35.981] [mlr3] Running benchmark with 10 resampling iterations
## INFO  [08:03:35.986] [mlr3] Applying learner 'regr.svm' on task 'PredictionSet[1:13]' (iter 1/10)
## INFO  [08:03:36.004] [mlr3] Applying learner 'regr.svm' on task 'PredictionSet[1:13]' (iter 2/10)
## INFO  [08:03:36.022] [mlr3] Applying learner 'regr.svm' on task 'PredictionSet[1:13]' (iter 3/10)
## INFO  [08:03:36.042] [mlr3] Applying learner 'regr.svm' on task 'PredictionSet[1:13]' (iter 4/10)
## INFO  [08:03:36.061] [mlr3] Applying learner 'regr.svm' on task 'PredictionSet[1:13]' (iter 5/10)
## INFO  [08:03:36.079] [mlr3] Applying learner 'regr.svm' on task 'PredictionSet[1:13]' (iter 6/10)
## INFO  [08:03:36.097] [mlr3] Applying learner 'regr.svm' on task 'PredictionSet[1:13]' (iter 7/10)
## INFO  [08:03:36.117] [mlr3] Applying learner 'regr.svm' on task 'PredictionSet[1:13]' (iter 8/10)
## INFO  [08:03:36.136] [mlr3] Applying learner 'regr.svm' on task 'PredictionSet[1:13]' (iter 9/10)
## INFO  [08:03:36.154] [mlr3] Applying learner 'regr.svm' on task 'PredictionSet[1:13]' (iter 10/10)
## INFO  [08:03:36.181] [mlr3] Finished benchmark
## INFO  [08:03:36.216] [bbotk] Result of batch 1:
## INFO  [08:03:36.218] [bbotk]  tolerance   gamma   epsilon           type  kernel regr.rmse warnings errors
## INFO  [08:03:36.218] [bbotk]  0.6431429 25.0005 0.3857143 eps-regression sigmoid  1.886334        0      0
## INFO  [08:03:36.218] [bbotk]  runtime_learners                                uhash
## INFO  [08:03:36.218] [bbotk]             0.082 ae3362c4-a778-4db1-b727-64935e9ed1ee
## INFO  [08:03:36.219] [bbotk] Evaluating 1 configuration(s)
## INFO  [08:03:36.235] [mlr3] Running benchmark with 10 resampling iterations
## INFO  [08:03:36.240] [mlr3] Applying learner 'regr.svm' on task 'PredictionSet[1:13]' (iter 1/10)
## INFO  [08:03:36.258] [mlr3] Applying learner 'regr.svm' on task 'PredictionSet[1:13]' (iter 2/10)
## INFO  [08:03:36.275] [mlr3] Applying learner 'regr.svm' on task 'PredictionSet[1:13]' (iter 3/10)
## INFO  [08:03:36.292] [mlr3] Applying learner 'regr.svm' on task 'PredictionSet[1:13]' (iter 4/10)
## INFO  [08:03:36.308] [mlr3] Applying learner 'regr.svm' on task 'PredictionSet[1:13]' (iter 5/10)
## INFO  [08:03:36.325] [mlr3] Applying learner 'regr.svm' on task 'PredictionSet[1:13]' (iter 6/10)
## INFO  [08:03:36.343] [mlr3] Applying learner 'regr.svm' on task 'PredictionSet[1:13]' (iter 7/10)
## INFO  [08:03:36.360] [mlr3] Applying learner 'regr.svm' on task 'PredictionSet[1:13]' (iter 8/10)
## INFO  [08:03:36.377] [mlr3] Applying learner 'regr.svm' on task 'PredictionSet[1:13]' (iter 9/10)
## INFO  [08:03:36.393] [mlr3] Applying learner 'regr.svm' on task 'PredictionSet[1:13]' (iter 10/10)
## INFO  [08:03:36.411] [mlr3] Finished benchmark
## INFO  [08:03:36.444] [bbotk] Result of batch 2:
## INFO  [08:03:36.445] [bbotk]  tolerance   gamma   epsilon           type kernel  regr.rmse warnings errors
## INFO  [08:03:36.445] [bbotk]  0.1936429 25.0005 0.5571429 eps-regression radial 0.04249034        0      0
## INFO  [08:03:36.445] [bbotk]  runtime_learners                                uhash
## INFO  [08:03:36.445] [bbotk]             0.072 68bc1d3c-3b6e-4b2a-8d71-42c27a1de217
## INFO  [08:03:36.447] [bbotk] Evaluating 1 configuration(s)
## INFO  [08:03:36.463] [mlr3] Running benchmark with 10 resampling iterations
## INFO  [08:03:36.468] [mlr3] Applying learner 'regr.svm' on task 'PredictionSet[1:13]' (iter 1/10)
## INFO  [08:03:36.519] [mlr3] Applying learner 'regr.svm' on task 'PredictionSet[1:13]' (iter 2/10)
## INFO  [08:03:36.542] [mlr3] Applying learner 'regr.svm' on task 'PredictionSet[1:13]' (iter 3/10)
## INFO  [08:03:36.565] [mlr3] Applying learner 'regr.svm' on task 'PredictionSet[1:13]' (iter 4/10)
## INFO  [08:03:36.586] [mlr3] Applying learner 'regr.svm' on task 'PredictionSet[1:13]' (iter 5/10)
## INFO  [08:03:36.604] [mlr3] Applying learner 'regr.svm' on task 'PredictionSet[1:13]' (iter 6/10)
## INFO  [08:03:36.621] [mlr3] Applying learner 'regr.svm' on task 'PredictionSet[1:13]' (iter 7/10)
## INFO  [08:03:36.640] [mlr3] Applying learner 'regr.svm' on task 'PredictionSet[1:13]' (iter 8/10)
## INFO  [08:03:36.657] [mlr3] Applying learner 'regr.svm' on task 'PredictionSet[1:13]' (iter 9/10)
## INFO  [08:03:36.674] [mlr3] Applying learner 'regr.svm' on task 'PredictionSet[1:13]' (iter 10/10)
## INFO  [08:03:36.690] [mlr3] Finished benchmark
## INFO  [08:03:36.723] [bbotk] Result of batch 3:
## INFO  [08:03:36.725] [bbotk]  tolerance    gamma epsilon           type kernel  regr.rmse warnings errors
## INFO  [08:03:36.725] [bbotk]  0.1936429 7.143714     0.1 eps-regression radial 0.03324965        0      0
## INFO  [08:03:36.725] [bbotk]  runtime_learners                                uhash
## INFO  [08:03:36.725] [bbotk]             0.104 b6e139b0-0c8c-4285-9270-8529412d93c4
## INFO  [08:03:36.726] [bbotk] Evaluating 1 configuration(s)
## INFO  [08:03:36.742] [mlr3] Running benchmark with 10 resampling iterations
## INFO  [08:03:36.747] [mlr3] Applying learner 'regr.svm' on task 'PredictionSet[1:13]' (iter 1/10)
## INFO  [08:03:36.765] [mlr3] Applying learner 'regr.svm' on task 'PredictionSet[1:13]' (iter 2/10)
## INFO  [08:03:36.784] [mlr3] Applying learner 'regr.svm' on task 'PredictionSet[1:13]' (iter 3/10)
## INFO  [08:03:36.803] [mlr3] Applying learner 'regr.svm' on task 'PredictionSet[1:13]' (iter 4/10)
## INFO  [08:03:36.822] [mlr3] Applying learner 'regr.svm' on task 'PredictionSet[1:13]' (iter 5/10)
## INFO  [08:03:36.840] [mlr3] Applying learner 'regr.svm' on task 'PredictionSet[1:13]' (iter 6/10)
## INFO  [08:03:36.859] [mlr3] Applying learner 'regr.svm' on task 'PredictionSet[1:13]' (iter 7/10)
## INFO  [08:03:36.878] [mlr3] Applying learner 'regr.svm' on task 'PredictionSet[1:13]' (iter 8/10)
## INFO  [08:03:36.897] [mlr3] Applying learner 'regr.svm' on task 'PredictionSet[1:13]' (iter 9/10)
## INFO  [08:03:36.925] [mlr3] Applying learner 'regr.svm' on task 'PredictionSet[1:13]' (iter 10/10)
## INFO  [08:03:36.949] [mlr3] Finished benchmark
## INFO  [08:03:36.987] [bbotk] Result of batch 4:
## INFO  [08:03:36.989] [bbotk]  tolerance    gamma   epsilon           type  kernel regr.rmse warnings errors
## INFO  [08:03:36.989] [bbotk]  0.6431429 28.57186 0.7285714 eps-regression sigmoid  1.890104        0      0
## INFO  [08:03:36.989] [bbotk]  runtime_learners                                uhash
## INFO  [08:03:36.989] [bbotk]             0.087 364d6e67-e12c-47ba-8420-136c2774f310
## INFO  [08:03:36.990] [bbotk] Evaluating 1 configuration(s)
## INFO  [08:03:37.007] [mlr3] Running benchmark with 10 resampling iterations
## INFO  [08:03:37.013] [mlr3] Applying learner 'regr.svm' on task 'PredictionSet[1:13]' (iter 1/10)
## INFO  [08:03:37.032] [mlr3] Applying learner 'regr.svm' on task 'PredictionSet[1:13]' (iter 2/10)
## INFO  [08:03:37.050] [mlr3] Applying learner 'regr.svm' on task 'PredictionSet[1:13]' (iter 3/10)
## INFO  [08:03:37.068] [mlr3] Applying learner 'regr.svm' on task 'PredictionSet[1:13]' (iter 4/10)
## INFO  [08:03:37.088] [mlr3] Applying learner 'regr.svm' on task 'PredictionSet[1:13]' (iter 5/10)
## INFO  [08:03:37.107] [mlr3] Applying learner 'regr.svm' on task 'PredictionSet[1:13]' (iter 6/10)
## INFO  [08:03:37.125] [mlr3] Applying learner 'regr.svm' on task 'PredictionSet[1:13]' (iter 7/10)
## INFO  [08:03:37.143] [mlr3] Applying learner 'regr.svm' on task 'PredictionSet[1:13]' (iter 8/10)
## INFO  [08:03:37.163] [mlr3] Applying learner 'regr.svm' on task 'PredictionSet[1:13]' (iter 9/10)
## INFO  [08:03:37.181] [mlr3] Applying learner 'regr.svm' on task 'PredictionSet[1:13]' (iter 10/10)
## INFO  [08:03:37.199] [mlr3] Finished benchmark
## INFO  [08:03:37.232] [bbotk] Result of batch 5:
## INFO  [08:03:37.234] [bbotk]  tolerance    gamma   epsilon           type  kernel regr.rmse warnings errors
## INFO  [08:03:37.234] [bbotk]  0.3220714 42.85729 0.1571429 eps-regression sigmoid  1.879635        0      0
## INFO  [08:03:37.234] [bbotk]  runtime_learners                                uhash
## INFO  [08:03:37.234] [bbotk]             0.082 fb7f6d36-c7f8-4311-a15e-b44e9281381e
## INFO  [08:03:37.236] [bbotk] Evaluating 1 configuration(s)
## INFO  [08:03:37.253] [mlr3] Running benchmark with 10 resampling iterations
## INFO  [08:03:37.258] [mlr3] Applying learner 'regr.svm' on task 'PredictionSet[1:13]' (iter 1/10)
## INFO  [08:03:37.275] [mlr3] Applying learner 'regr.svm' on task 'PredictionSet[1:13]' (iter 2/10)
## INFO  [08:03:37.301] [mlr3] Applying learner 'regr.svm' on task 'PredictionSet[1:13]' (iter 3/10)
## INFO  [08:03:37.323] [mlr3] Applying learner 'regr.svm' on task 'PredictionSet[1:13]' (iter 4/10)
## INFO  [08:03:37.344] [mlr3] Applying learner 'regr.svm' on task 'PredictionSet[1:13]' (iter 5/10)
## INFO  [08:03:37.363] [mlr3] Applying learner 'regr.svm' on task 'PredictionSet[1:13]' (iter 6/10)
## INFO  [08:03:37.382] [mlr3] Applying learner 'regr.svm' on task 'PredictionSet[1:13]' (iter 7/10)
## INFO  [08:03:37.401] [mlr3] Applying learner 'regr.svm' on task 'PredictionSet[1:13]' (iter 8/10)
## INFO  [08:03:37.417] [mlr3] Applying learner 'regr.svm' on task 'PredictionSet[1:13]' (iter 9/10)
## INFO  [08:03:37.434] [mlr3] Applying learner 'regr.svm' on task 'PredictionSet[1:13]' (iter 10/10)
## INFO  [08:03:37.451] [mlr3] Finished benchmark
## INFO  [08:03:37.484] [bbotk] Result of batch 6:
## INFO  [08:03:37.486] [bbotk]  tolerance    gamma   epsilon           type kernel  regr.rmse warnings errors
## INFO  [08:03:37.486] [bbotk]  0.5147143 39.28593 0.2714286 eps-regression radial 0.03968989        0      0
## INFO  [08:03:37.486] [bbotk]  runtime_learners                                uhash
## INFO  [08:03:37.486] [bbotk]             0.083 71090156-d1d9-45d6-99c6-1cac015d74e8
## INFO  [08:03:37.487] [bbotk] Evaluating 1 configuration(s)
## INFO  [08:03:37.503] [mlr3] Running benchmark with 10 resampling iterations
## INFO  [08:03:37.508] [mlr3] Applying learner 'regr.svm' on task 'PredictionSet[1:13]' (iter 1/10)
## INFO  [08:03:37.526] [mlr3] Applying learner 'regr.svm' on task 'PredictionSet[1:13]' (iter 2/10)
## INFO  [08:03:37.546] [mlr3] Applying learner 'regr.svm' on task 'PredictionSet[1:13]' (iter 3/10)
## INFO  [08:03:37.564] [mlr3] Applying learner 'regr.svm' on task 'PredictionSet[1:13]' (iter 4/10)
## INFO  [08:03:37.582] [mlr3] Applying learner 'regr.svm' on task 'PredictionSet[1:13]' (iter 5/10)
## INFO  [08:03:37.600] [mlr3] Applying learner 'regr.svm' on task 'PredictionSet[1:13]' (iter 6/10)
## INFO  [08:03:37.619] [mlr3] Applying learner 'regr.svm' on task 'PredictionSet[1:13]' (iter 7/10)
## INFO  [08:03:37.638] [mlr3] Applying learner 'regr.svm' on task 'PredictionSet[1:13]' (iter 8/10)
## INFO  [08:03:37.656] [mlr3] Applying learner 'regr.svm' on task 'PredictionSet[1:13]' (iter 9/10)
## INFO  [08:03:37.684] [mlr3] Applying learner 'regr.svm' on task 'PredictionSet[1:13]' (iter 10/10)
## INFO  [08:03:37.706] [mlr3] Finished benchmark
## INFO  [08:03:37.745] [bbotk] Result of batch 7:
## INFO  [08:03:37.747] [bbotk]  tolerance    gamma   epsilon           type  kernel regr.rmse warnings errors
## INFO  [08:03:37.747] [bbotk]  0.6431429 42.85729 0.5571429 eps-regression sigmoid  1.879792        0      0
## INFO  [08:03:37.747] [bbotk]  runtime_learners                                uhash
## INFO  [08:03:37.747] [bbotk]             0.087 e996e0a8-3c69-4ea9-bdf0-cfb10ca7ac38
## INFO  [08:03:37.749] [bbotk] Evaluating 1 configuration(s)
## INFO  [08:03:37.767] [mlr3] Running benchmark with 10 resampling iterations
## INFO  [08:03:37.772] [mlr3] Applying learner 'regr.svm' on task 'PredictionSet[1:13]' (iter 1/10)
## INFO  [08:03:37.788] [mlr3] Applying learner 'regr.svm' on task 'PredictionSet[1:13]' (iter 2/10)
## INFO  [08:03:37.804] [mlr3] Applying learner 'regr.svm' on task 'PredictionSet[1:13]' (iter 3/10)
## INFO  [08:03:37.821] [mlr3] Applying learner 'regr.svm' on task 'PredictionSet[1:13]' (iter 4/10)
## INFO  [08:03:37.839] [mlr3] Applying learner 'regr.svm' on task 'PredictionSet[1:13]' (iter 5/10)
## INFO  [08:03:37.855] [mlr3] Applying learner 'regr.svm' on task 'PredictionSet[1:13]' (iter 6/10)
## INFO  [08:03:37.872] [mlr3] Applying learner 'regr.svm' on task 'PredictionSet[1:13]' (iter 7/10)
## INFO  [08:03:37.887] [mlr3] Applying learner 'regr.svm' on task 'PredictionSet[1:13]' (iter 8/10)
## INFO  [08:03:37.904] [mlr3] Applying learner 'regr.svm' on task 'PredictionSet[1:13]' (iter 9/10)
## INFO  [08:03:37.922] [mlr3] Applying learner 'regr.svm' on task 'PredictionSet[1:13]' (iter 10/10)
## INFO  [08:03:37.938] [mlr3] Finished benchmark
## INFO  [08:03:37.970] [bbotk] Result of batch 8:
## INFO  [08:03:37.972] [bbotk]  tolerance    gamma   epsilon           type kernel regr.rmse warnings errors
## INFO  [08:03:37.972] [bbotk]  0.6431429 14.28643 0.3285714 eps-regression radial 0.0372495        0      0
## INFO  [08:03:37.972] [bbotk]  runtime_learners                                uhash
## INFO  [08:03:37.972] [bbotk]             0.066 08d906bc-ecdb-454a-a9c7-6e88740e7cca
## INFO  [08:03:37.974] [bbotk] Evaluating 1 configuration(s)
## INFO  [08:03:37.991] [mlr3] Running benchmark with 10 resampling iterations
## INFO  [08:03:37.996] [mlr3] Applying learner 'regr.svm' on task 'PredictionSet[1:13]' (iter 1/10)
## INFO  [08:03:38.013] [mlr3] Applying learner 'regr.svm' on task 'PredictionSet[1:13]' (iter 2/10)
## INFO  [08:03:38.038] [mlr3] Applying learner 'regr.svm' on task 'PredictionSet[1:13]' (iter 3/10)
## INFO  [08:03:38.060] [mlr3] Applying learner 'regr.svm' on task 'PredictionSet[1:13]' (iter 4/10)
## INFO  [08:03:38.081] [mlr3] Applying learner 'regr.svm' on task 'PredictionSet[1:13]' (iter 5/10)
## INFO  [08:03:38.101] [mlr3] Applying learner 'regr.svm' on task 'PredictionSet[1:13]' (iter 6/10)
## INFO  [08:03:38.118] [mlr3] Applying learner 'regr.svm' on task 'PredictionSet[1:13]' (iter 7/10)
## INFO  [08:03:38.137] [mlr3] Applying learner 'regr.svm' on task 'PredictionSet[1:13]' (iter 8/10)
## INFO  [08:03:38.154] [mlr3] Applying learner 'regr.svm' on task 'PredictionSet[1:13]' (iter 9/10)
## INFO  [08:03:38.171] [mlr3] Applying learner 'regr.svm' on task 'PredictionSet[1:13]' (iter 10/10)
## INFO  [08:03:38.187] [mlr3] Finished benchmark
## INFO  [08:03:38.220] [bbotk] Result of batch 9:
## INFO  [08:03:38.222] [bbotk]   tolerance    gamma   epsilon           type kernel  regr.rmse warnings errors
## INFO  [08:03:38.222] [bbotk]  0.06521429 46.42864 0.6714286 eps-regression radial 0.04605819        0      0
## INFO  [08:03:38.222] [bbotk]  runtime_learners                                uhash
## INFO  [08:03:38.222] [bbotk]             0.077 c46968dd-3ed9-444f-91d2-9659b9ce94e4
## INFO  [08:03:38.223] [bbotk] Evaluating 1 configuration(s)
## INFO  [08:03:38.239] [mlr3] Running benchmark with 10 resampling iterations
## INFO  [08:03:38.244] [mlr3] Applying learner 'regr.svm' on task 'PredictionSet[1:13]' (iter 1/10)
## INFO  [08:03:38.260] [mlr3] Applying learner 'regr.svm' on task 'PredictionSet[1:13]' (iter 2/10)
## INFO  [08:03:38.276] [mlr3] Applying learner 'regr.svm' on task 'PredictionSet[1:13]' (iter 3/10)
## INFO  [08:03:38.294] [mlr3] Applying learner 'regr.svm' on task 'PredictionSet[1:13]' (iter 4/10)
## INFO  [08:03:38.311] [mlr3] Applying learner 'regr.svm' on task 'PredictionSet[1:13]' (iter 5/10)
## INFO  [08:03:38.327] [mlr3] Applying learner 'regr.svm' on task 'PredictionSet[1:13]' (iter 6/10)
## INFO  [08:03:38.344] [mlr3] Applying learner 'regr.svm' on task 'PredictionSet[1:13]' (iter 7/10)
## INFO  [08:03:38.362] [mlr3] Applying learner 'regr.svm' on task 'PredictionSet[1:13]' (iter 8/10)
## INFO  [08:03:38.378] [mlr3] Applying learner 'regr.svm' on task 'PredictionSet[1:13]' (iter 9/10)
## INFO  [08:03:38.404] [mlr3] Applying learner 'regr.svm' on task 'PredictionSet[1:13]' (iter 10/10)
## INFO  [08:03:38.424] [mlr3] Finished benchmark
## INFO  [08:03:38.465] [bbotk] Result of batch 10:
## INFO  [08:03:38.467] [bbotk]  tolerance    gamma   epsilon           type kernel  regr.rmse warnings errors
## INFO  [08:03:38.467] [bbotk]     0.4505 21.42914 0.6142857 eps-regression radial 0.04276512        0      0
## INFO  [08:03:38.467] [bbotk]  runtime_learners                                uhash
## INFO  [08:03:38.467] [bbotk]              0.07 9474da56-75d3-4d5e-9b9c-fdc43c983e7d
## INFO  [08:03:38.472] [bbotk] Finished optimizing after 10 evaluation(s)
## INFO  [08:03:38.472] [bbotk] Result:
## INFO  [08:03:38.474] [bbotk]  tolerance    gamma epsilon           type kernel learner_param_vals  x_domain
## INFO  [08:03:38.474] [bbotk]  0.1936429 7.143714     0.1 eps-regression radial          <list[5]> <list[5]>
## INFO  [08:03:38.474] [bbotk]   regr.rmse
## INFO  [08:03:38.474] [bbotk]  0.03324965
## INFO  [08:03:38.616] [bbotk] Starting to optimize 3 parameter(s) with '<TunerGridSearch>' and '<TerminatorEvals> [n_evals=10, k=0]'
## INFO  [08:03:38.620] [bbotk] Evaluating 1 configuration(s)
## INFO  [08:03:38.631] [mlr3] Running benchmark with 10 resampling iterations
## INFO  [08:03:38.636] [mlr3] Applying learner 'regr.kknn' on task 'PredictionSet[1:13]' (iter 1/10)
## INFO  [08:03:38.657] [mlr3] Applying learner 'regr.kknn' on task 'PredictionSet[1:13]' (iter 2/10)
## INFO  [08:03:38.679] [mlr3] Applying learner 'regr.kknn' on task 'PredictionSet[1:13]' (iter 3/10)
## INFO  [08:03:38.700] [mlr3] Applying learner 'regr.kknn' on task 'PredictionSet[1:13]' (iter 4/10)
## INFO  [08:03:38.731] [mlr3] Applying learner 'regr.kknn' on task 'PredictionSet[1:13]' (iter 5/10)
## INFO  [08:03:38.759] [mlr3] Applying learner 'regr.kknn' on task 'PredictionSet[1:13]' (iter 6/10)
## INFO  [08:03:38.783] [mlr3] Applying learner 'regr.kknn' on task 'PredictionSet[1:13]' (iter 7/10)
## INFO  [08:03:38.805] [mlr3] Applying learner 'regr.kknn' on task 'PredictionSet[1:13]' (iter 8/10)
## INFO  [08:03:38.827] [mlr3] Applying learner 'regr.kknn' on task 'PredictionSet[1:13]' (iter 9/10)
## INFO  [08:03:38.847] [mlr3] Applying learner 'regr.kknn' on task 'PredictionSet[1:13]' (iter 10/10)
## INFO  [08:03:38.867] [mlr3] Finished benchmark
## INFO  [08:03:38.899] [bbotk] Result of batch 1:
## INFO  [08:03:38.901] [bbotk]  k distance       kernel  regr.rmse warnings errors runtime_learners
## INFO  [08:03:38.901] [bbotk]  5    10.05 epanechnikov 0.02138245        0      0             0.11
## INFO  [08:03:38.901] [bbotk]                                 uhash
## INFO  [08:03:38.901] [bbotk]  25aa1947-22df-416d-b20f-af24c9979b21
## INFO  [08:03:38.902] [bbotk] Evaluating 1 configuration(s)
## INFO  [08:03:38.913] [mlr3] Running benchmark with 10 resampling iterations
## INFO  [08:03:38.918] [mlr3] Applying learner 'regr.kknn' on task 'PredictionSet[1:13]' (iter 1/10)
## INFO  [08:03:38.938] [mlr3] Applying learner 'regr.kknn' on task 'PredictionSet[1:13]' (iter 2/10)
## INFO  [08:03:38.960] [mlr3] Applying learner 'regr.kknn' on task 'PredictionSet[1:13]' (iter 3/10)
## INFO  [08:03:38.981] [mlr3] Applying learner 'regr.kknn' on task 'PredictionSet[1:13]' (iter 4/10)
## INFO  [08:03:39.003] [mlr3] Applying learner 'regr.kknn' on task 'PredictionSet[1:13]' (iter 5/10)
## INFO  [08:03:39.024] [mlr3] Applying learner 'regr.kknn' on task 'PredictionSet[1:13]' (iter 6/10)
## INFO  [08:03:39.047] [mlr3] Applying learner 'regr.kknn' on task 'PredictionSet[1:13]' (iter 7/10)
## INFO  [08:03:39.068] [mlr3] Applying learner 'regr.kknn' on task 'PredictionSet[1:13]' (iter 8/10)
## INFO  [08:03:39.097] [mlr3] Applying learner 'regr.kknn' on task 'PredictionSet[1:13]' (iter 9/10)
## INFO  [08:03:39.125] [mlr3] Applying learner 'regr.kknn' on task 'PredictionSet[1:13]' (iter 10/10)
## INFO  [08:03:39.150] [mlr3] Finished benchmark
## INFO  [08:03:39.186] [bbotk] Result of batch 2:
## INFO  [08:03:39.188] [bbotk]  k distance kernel  regr.rmse warnings errors runtime_learners
## INFO  [08:03:39.188] [bbotk]  8 8.628571   rank 0.02093132        0      0            0.121
## INFO  [08:03:39.188] [bbotk]                                 uhash
## INFO  [08:03:39.188] [bbotk]  4c0e99bc-72ea-460c-8c1b-36791b9249d6
## INFO  [08:03:39.190] [bbotk] Evaluating 1 configuration(s)
## INFO  [08:03:39.201] [mlr3] Running benchmark with 10 resampling iterations
## INFO  [08:03:39.206] [mlr3] Applying learner 'regr.kknn' on task 'PredictionSet[1:13]' (iter 1/10)
## INFO  [08:03:39.227] [mlr3] Applying learner 'regr.kknn' on task 'PredictionSet[1:13]' (iter 2/10)
## INFO  [08:03:39.247] [mlr3] Applying learner 'regr.kknn' on task 'PredictionSet[1:13]' (iter 3/10)
## INFO  [08:03:39.270] [mlr3] Applying learner 'regr.kknn' on task 'PredictionSet[1:13]' (iter 4/10)
## INFO  [08:03:39.291] [mlr3] Applying learner 'regr.kknn' on task 'PredictionSet[1:13]' (iter 5/10)
## INFO  [08:03:39.311] [mlr3] Applying learner 'regr.kknn' on task 'PredictionSet[1:13]' (iter 6/10)
## INFO  [08:03:39.334] [mlr3] Applying learner 'regr.kknn' on task 'PredictionSet[1:13]' (iter 7/10)
## INFO  [08:03:39.355] [mlr3] Applying learner 'regr.kknn' on task 'PredictionSet[1:13]' (iter 8/10)
## INFO  [08:03:39.376] [mlr3] Applying learner 'regr.kknn' on task 'PredictionSet[1:13]' (iter 9/10)
## INFO  [08:03:39.397] [mlr3] Applying learner 'regr.kknn' on task 'PredictionSet[1:13]' (iter 10/10)
## INFO  [08:03:39.420] [mlr3] Finished benchmark
## INFO  [08:03:39.452] [bbotk] Result of batch 3:
## INFO  [08:03:39.454] [bbotk]   k distance   kernel  regr.rmse warnings errors runtime_learners
## INFO  [08:03:39.454] [bbotk]  20 2.942857 gaussian 0.02074962        0      0            0.107
## INFO  [08:03:39.454] [bbotk]                                 uhash
## INFO  [08:03:39.454] [bbotk]  698593e6-c291-4501-ab9c-438e1fd13c95
## INFO  [08:03:39.455] [bbotk] Evaluating 1 configuration(s)
## INFO  [08:03:39.476] [mlr3] Running benchmark with 10 resampling iterations
## INFO  [08:03:39.483] [mlr3] Applying learner 'regr.kknn' on task 'PredictionSet[1:13]' (iter 1/10)
## INFO  [08:03:39.510] [mlr3] Applying learner 'regr.kknn' on task 'PredictionSet[1:13]' (iter 2/10)
## INFO  [08:03:39.534] [mlr3] Applying learner 'regr.kknn' on task 'PredictionSet[1:13]' (iter 3/10)
## INFO  [08:03:39.556] [mlr3] Applying learner 'regr.kknn' on task 'PredictionSet[1:13]' (iter 4/10)
## INFO  [08:03:39.579] [mlr3] Applying learner 'regr.kknn' on task 'PredictionSet[1:13]' (iter 5/10)
## INFO  [08:03:39.600] [mlr3] Applying learner 'regr.kknn' on task 'PredictionSet[1:13]' (iter 6/10)
## INFO  [08:03:39.620] [mlr3] Applying learner 'regr.kknn' on task 'PredictionSet[1:13]' (iter 7/10)
## INFO  [08:03:39.644] [mlr3] Applying learner 'regr.kknn' on task 'PredictionSet[1:13]' (iter 8/10)
## INFO  [08:03:39.665] [mlr3] Applying learner 'regr.kknn' on task 'PredictionSet[1:13]' (iter 9/10)
## INFO  [08:03:39.685] [mlr3] Applying learner 'regr.kknn' on task 'PredictionSet[1:13]' (iter 10/10)
## INFO  [08:03:39.706] [mlr3] Finished benchmark
## INFO  [08:03:39.740] [bbotk] Result of batch 4:
## INFO  [08:03:39.741] [bbotk]   k distance       kernel  regr.rmse warnings errors runtime_learners
## INFO  [08:03:39.741] [bbotk]  20 8.628571 epanechnikov 0.02272176        0      0            0.112
## INFO  [08:03:39.741] [bbotk]                                 uhash
## INFO  [08:03:39.741] [bbotk]  08d99ab0-5185-4fe3-8fd1-935274c36307
## INFO  [08:03:39.743] [bbotk] Evaluating 1 configuration(s)
## INFO  [08:03:39.754] [mlr3] Running benchmark with 10 resampling iterations
## INFO  [08:03:39.759] [mlr3] Applying learner 'regr.kknn' on task 'PredictionSet[1:13]' (iter 1/10)
## INFO  [08:03:39.780] [mlr3] Applying learner 'regr.kknn' on task 'PredictionSet[1:13]' (iter 2/10)
## INFO  [08:03:39.803] [mlr3] Applying learner 'regr.kknn' on task 'PredictionSet[1:13]' (iter 3/10)
## INFO  [08:03:39.824] [mlr3] Applying learner 'regr.kknn' on task 'PredictionSet[1:13]' (iter 4/10)
## INFO  [08:03:39.854] [mlr3] Applying learner 'regr.kknn' on task 'PredictionSet[1:13]' (iter 5/10)
## INFO  [08:03:39.881] [mlr3] Applying learner 'regr.kknn' on task 'PredictionSet[1:13]' (iter 6/10)
## INFO  [08:03:39.906] [mlr3] Applying learner 'regr.kknn' on task 'PredictionSet[1:13]' (iter 7/10)
## INFO  [08:03:39.929] [mlr3] Applying learner 'regr.kknn' on task 'PredictionSet[1:13]' (iter 8/10)
## INFO  [08:03:39.953] [mlr3] Applying learner 'regr.kknn' on task 'PredictionSet[1:13]' (iter 9/10)
## INFO  [08:03:39.973] [mlr3] Applying learner 'regr.kknn' on task 'PredictionSet[1:13]' (iter 10/10)
## INFO  [08:03:39.993] [mlr3] Finished benchmark
## INFO  [08:03:40.027] [bbotk] Result of batch 5:
## INFO  [08:03:40.029] [bbotk]   k distance kernel  regr.rmse warnings errors runtime_learners
## INFO  [08:03:40.029] [bbotk]  19 11.47143    inv 0.01977043        0      0            0.119
## INFO  [08:03:40.029] [bbotk]                                 uhash
## INFO  [08:03:40.029] [bbotk]  c4e56c62-3d5f-4c5d-b955-2441cf5c732b
## INFO  [08:03:40.030] [bbotk] Evaluating 1 configuration(s)
## INFO  [08:03:40.041] [mlr3] Running benchmark with 10 resampling iterations
## INFO  [08:03:40.046] [mlr3] Applying learner 'regr.kknn' on task 'PredictionSet[1:13]' (iter 1/10)
## INFO  [08:03:40.066] [mlr3] Applying learner 'regr.kknn' on task 'PredictionSet[1:13]' (iter 2/10)
## INFO  [08:03:40.087] [mlr3] Applying learner 'regr.kknn' on task 'PredictionSet[1:13]' (iter 3/10)
## INFO  [08:03:40.108] [mlr3] Applying learner 'regr.kknn' on task 'PredictionSet[1:13]' (iter 4/10)
## INFO  [08:03:40.129] [mlr3] Applying learner 'regr.kknn' on task 'PredictionSet[1:13]' (iter 5/10)
## INFO  [08:03:40.149] [mlr3] Applying learner 'regr.kknn' on task 'PredictionSet[1:13]' (iter 6/10)
## INFO  [08:03:40.172] [mlr3] Applying learner 'regr.kknn' on task 'PredictionSet[1:13]' (iter 7/10)
## INFO  [08:03:40.193] [mlr3] Applying learner 'regr.kknn' on task 'PredictionSet[1:13]' (iter 8/10)
## INFO  [08:03:40.221] [mlr3] Applying learner 'regr.kknn' on task 'PredictionSet[1:13]' (iter 9/10)
## INFO  [08:03:40.248] [mlr3] Applying learner 'regr.kknn' on task 'PredictionSet[1:13]' (iter 10/10)
## INFO  [08:03:40.273] [mlr3] Finished benchmark
## INFO  [08:03:40.310] [bbotk] Result of batch 6:
## INFO  [08:03:40.312] [bbotk]  k distance     kernel  regr.rmse warnings errors runtime_learners
## INFO  [08:03:40.312] [bbotk]  6 17.15714 triangular 0.02143143        0      0            0.106
## INFO  [08:03:40.312] [bbotk]                                 uhash
## INFO  [08:03:40.312] [bbotk]  9d626010-f82e-4ce9-923a-903c6a835a9a
## INFO  [08:03:40.314] [bbotk] Evaluating 1 configuration(s)
## INFO  [08:03:40.325] [mlr3] Running benchmark with 10 resampling iterations
## INFO  [08:03:40.330] [mlr3] Applying learner 'regr.kknn' on task 'PredictionSet[1:13]' (iter 1/10)
## INFO  [08:03:40.351] [mlr3] Applying learner 'regr.kknn' on task 'PredictionSet[1:13]' (iter 2/10)
## INFO  [08:03:40.371] [mlr3] Applying learner 'regr.kknn' on task 'PredictionSet[1:13]' (iter 3/10)
## INFO  [08:03:40.393] [mlr3] Applying learner 'regr.kknn' on task 'PredictionSet[1:13]' (iter 4/10)
## INFO  [08:03:40.414] [mlr3] Applying learner 'regr.kknn' on task 'PredictionSet[1:13]' (iter 5/10)
## INFO  [08:03:40.435] [mlr3] Applying learner 'regr.kknn' on task 'PredictionSet[1:13]' (iter 6/10)
## INFO  [08:03:40.456] [mlr3] Applying learner 'regr.kknn' on task 'PredictionSet[1:13]' (iter 7/10)
## INFO  [08:03:40.478] [mlr3] Applying learner 'regr.kknn' on task 'PredictionSet[1:13]' (iter 8/10)
## INFO  [08:03:40.499] [mlr3] Applying learner 'regr.kknn' on task 'PredictionSet[1:13]' (iter 9/10)
## INFO  [08:03:40.520] [mlr3] Applying learner 'regr.kknn' on task 'PredictionSet[1:13]' (iter 10/10)
## INFO  [08:03:40.542] [mlr3] Finished benchmark
## INFO  [08:03:40.574] [bbotk] Result of batch 7:
## INFO  [08:03:40.576] [bbotk]   k distance    kernel  regr.rmse warnings errors runtime_learners
## INFO  [08:03:40.576] [bbotk]  13 14.31429 triweight 0.02092405        0      0            0.102
## INFO  [08:03:40.576] [bbotk]                                 uhash
## INFO  [08:03:40.576] [bbotk]  3900af40-e188-4a3b-8960-c0bb5be954a0
## INFO  [08:03:40.577] [bbotk] Evaluating 1 configuration(s)
## INFO  [08:03:40.597] [mlr3] Running benchmark with 10 resampling iterations
## INFO  [08:03:40.604] [mlr3] Applying learner 'regr.kknn' on task 'PredictionSet[1:13]' (iter 1/10)
## INFO  [08:03:40.630] [mlr3] Applying learner 'regr.kknn' on task 'PredictionSet[1:13]' (iter 2/10)
## INFO  [08:03:40.654] [mlr3] Applying learner 'regr.kknn' on task 'PredictionSet[1:13]' (iter 3/10)
## INFO  [08:03:40.676] [mlr3] Applying learner 'regr.kknn' on task 'PredictionSet[1:13]' (iter 4/10)
## INFO  [08:03:40.699] [mlr3] Applying learner 'regr.kknn' on task 'PredictionSet[1:13]' (iter 5/10)
## INFO  [08:03:40.719] [mlr3] Applying learner 'regr.kknn' on task 'PredictionSet[1:13]' (iter 6/10)
## INFO  [08:03:40.740] [mlr3] Applying learner 'regr.kknn' on task 'PredictionSet[1:13]' (iter 7/10)
## INFO  [08:03:40.762] [mlr3] Applying learner 'regr.kknn' on task 'PredictionSet[1:13]' (iter 8/10)
## INFO  [08:03:40.783] [mlr3] Applying learner 'regr.kknn' on task 'PredictionSet[1:13]' (iter 9/10)
## INFO  [08:03:40.803] [mlr3] Applying learner 'regr.kknn' on task 'PredictionSet[1:13]' (iter 10/10)
## INFO  [08:03:40.824] [mlr3] Finished benchmark
## INFO  [08:03:40.857] [bbotk] Result of batch 8:
## INFO  [08:03:40.859] [bbotk]   k distance     kernel  regr.rmse warnings errors runtime_learners
## INFO  [08:03:40.859] [bbotk]  20 14.31429 triangular 0.02201368        0      0             0.11
## INFO  [08:03:40.859] [bbotk]                                 uhash
## INFO  [08:03:40.859] [bbotk]  24749a01-1568-40e5-b431-ee727e18d4fe
## INFO  [08:03:40.861] [bbotk] Evaluating 1 configuration(s)
## INFO  [08:03:40.871] [mlr3] Running benchmark with 10 resampling iterations
## INFO  [08:03:40.877] [mlr3] Applying learner 'regr.kknn' on task 'PredictionSet[1:13]' (iter 1/10)
## INFO  [08:03:40.897] [mlr3] Applying learner 'regr.kknn' on task 'PredictionSet[1:13]' (iter 2/10)
## INFO  [08:03:40.919] [mlr3] Applying learner 'regr.kknn' on task 'PredictionSet[1:13]' (iter 3/10)
## INFO  [08:03:40.940] [mlr3] Applying learner 'regr.kknn' on task 'PredictionSet[1:13]' (iter 4/10)
## INFO  [08:03:40.970] [mlr3] Applying learner 'regr.kknn' on task 'PredictionSet[1:13]' (iter 5/10)
## INFO  [08:03:40.998] [mlr3] Applying learner 'regr.kknn' on task 'PredictionSet[1:13]' (iter 6/10)
## INFO  [08:03:41.023] [mlr3] Applying learner 'regr.kknn' on task 'PredictionSet[1:13]' (iter 7/10)
## INFO  [08:03:41.045] [mlr3] Applying learner 'regr.kknn' on task 'PredictionSet[1:13]' (iter 8/10)
## INFO  [08:03:41.067] [mlr3] Applying learner 'regr.kknn' on task 'PredictionSet[1:13]' (iter 9/10)
## INFO  [08:03:41.088] [mlr3] Applying learner 'regr.kknn' on task 'PredictionSet[1:13]' (iter 10/10)
## INFO  [08:03:41.108] [mlr3] Finished benchmark
## INFO  [08:03:41.141] [bbotk] Result of batch 9:
## INFO  [08:03:41.143] [bbotk]   k distance      kernel  regr.rmse warnings errors runtime_learners
## INFO  [08:03:41.143] [bbotk]  15      0.1 rectangular 0.02013517        0      0            0.115
## INFO  [08:03:41.143] [bbotk]                                 uhash
## INFO  [08:03:41.143] [bbotk]  3589ae6b-0ae5-47f5-b1aa-e1c2dd12b44f
## INFO  [08:03:41.145] [bbotk] Evaluating 1 configuration(s)
## INFO  [08:03:41.155] [mlr3] Running benchmark with 10 resampling iterations
## INFO  [08:03:41.161] [mlr3] Applying learner 'regr.kknn' on task 'PredictionSet[1:13]' (iter 1/10)
## INFO  [08:03:41.181] [mlr3] Applying learner 'regr.kknn' on task 'PredictionSet[1:13]' (iter 2/10)
## INFO  [08:03:41.201] [mlr3] Applying learner 'regr.kknn' on task 'PredictionSet[1:13]' (iter 3/10)
## INFO  [08:03:41.224] [mlr3] Applying learner 'regr.kknn' on task 'PredictionSet[1:13]' (iter 4/10)
## INFO  [08:03:41.244] [mlr3] Applying learner 'regr.kknn' on task 'PredictionSet[1:13]' (iter 5/10)
## INFO  [08:03:41.265] [mlr3] Applying learner 'regr.kknn' on task 'PredictionSet[1:13]' (iter 6/10)
## INFO  [08:03:41.287] [mlr3] Applying learner 'regr.kknn' on task 'PredictionSet[1:13]' (iter 7/10)
## INFO  [08:03:41.308] [mlr3] Applying learner 'regr.kknn' on task 'PredictionSet[1:13]' (iter 8/10)
## INFO  [08:03:41.338] [mlr3] Applying learner 'regr.kknn' on task 'PredictionSet[1:13]' (iter 9/10)
## INFO  [08:03:41.365] [mlr3] Applying learner 'regr.kknn' on task 'PredictionSet[1:13]' (iter 10/10)
## INFO  [08:03:41.389] [mlr3] Finished benchmark
## INFO  [08:03:41.423] [bbotk] Result of batch 10:
## INFO  [08:03:41.425] [bbotk]  k distance kernel  regr.rmse warnings errors runtime_learners
## INFO  [08:03:41.425] [bbotk]  6 2.942857    inv 0.01629784        0      0            0.116
## INFO  [08:03:41.425] [bbotk]                                 uhash
## INFO  [08:03:41.425] [bbotk]  9ed6d591-2a87-4603-b35e-10aeb2f9baad
## INFO  [08:03:41.431] [bbotk] Finished optimizing after 10 evaluation(s)
## INFO  [08:03:41.432] [bbotk] Result:
## INFO  [08:03:41.433] [bbotk]  k distance kernel learner_param_vals  x_domain  regr.rmse
## INFO  [08:03:41.433] [bbotk]  6 2.942857    inv          <list[3]> <list[3]> 0.01629784
## INFO  [08:03:41.663] [bbotk] Starting to optimize 2 parameter(s) with '<TunerGridSearch>' and '<TerminatorEvals> [n_evals=10, k=0]'
## INFO  [08:03:41.666] [bbotk] Evaluating 1 configuration(s)
## INFO  [08:03:41.690] [mlr3] Running benchmark with 10 resampling iterations
## INFO  [08:03:41.695] [mlr3] Applying learner 'regr.ranger' on task 'PredictionSet[1:13]' (iter 1/10)
## INFO  [08:03:41.727] [mlr3] Applying learner 'regr.ranger' on task 'PredictionSet[1:13]' (iter 2/10)
## INFO  [08:03:41.761] [mlr3] Applying learner 'regr.ranger' on task 'PredictionSet[1:13]' (iter 3/10)
## INFO  [08:03:41.792] [mlr3] Applying learner 'regr.ranger' on task 'PredictionSet[1:13]' (iter 4/10)
## INFO  [08:03:41.825] [mlr3] Applying learner 'regr.ranger' on task 'PredictionSet[1:13]' (iter 5/10)
## INFO  [08:03:41.857] [mlr3] Applying learner 'regr.ranger' on task 'PredictionSet[1:13]' (iter 6/10)
## INFO  [08:03:41.891] [mlr3] Applying learner 'regr.ranger' on task 'PredictionSet[1:13]' (iter 7/10)
## INFO  [08:03:41.923] [mlr3] Applying learner 'regr.ranger' on task 'PredictionSet[1:13]' (iter 8/10)
## INFO  [08:03:41.956] [mlr3] Applying learner 'regr.ranger' on task 'PredictionSet[1:13]' (iter 9/10)
## INFO  [08:03:41.989] [mlr3] Applying learner 'regr.ranger' on task 'PredictionSet[1:13]' (iter 10/10)
## INFO  [08:03:42.021] [mlr3] Finished benchmark
## INFO  [08:03:42.053] [bbotk] Result of batch 1:
## INFO  [08:03:42.054] [bbotk]  max.depth num.trees  regr.rmse warnings errors runtime_learners
## INFO  [08:03:42.054] [bbotk]         14       316 0.01139871        0      0            0.223
## INFO  [08:03:42.054] [bbotk]                                 uhash
## INFO  [08:03:42.054] [bbotk]  388aa027-9bd2-40da-888d-458db006779c
## INFO  [08:03:42.056] [bbotk] Evaluating 1 configuration(s)
## INFO  [08:03:42.089] [mlr3] Running benchmark with 10 resampling iterations
## INFO  [08:03:42.095] [mlr3] Applying learner 'regr.ranger' on task 'PredictionSet[1:13]' (iter 1/10)
## INFO  [08:03:42.121] [mlr3] Applying learner 'regr.ranger' on task 'PredictionSet[1:13]' (iter 2/10)
## INFO  [08:03:42.144] [mlr3] Applying learner 'regr.ranger' on task 'PredictionSet[1:13]' (iter 3/10)
## INFO  [08:03:42.164] [mlr3] Applying learner 'regr.ranger' on task 'PredictionSet[1:13]' (iter 4/10)
## INFO  [08:03:42.187] [mlr3] Applying learner 'regr.ranger' on task 'PredictionSet[1:13]' (iter 5/10)
## INFO  [08:03:42.207] [mlr3] Applying learner 'regr.ranger' on task 'PredictionSet[1:13]' (iter 6/10)
## INFO  [08:03:42.227] [mlr3] Applying learner 'regr.ranger' on task 'PredictionSet[1:13]' (iter 7/10)
## INFO  [08:03:42.246] [mlr3] Applying learner 'regr.ranger' on task 'PredictionSet[1:13]' (iter 8/10)
## INFO  [08:03:42.268] [mlr3] Applying learner 'regr.ranger' on task 'PredictionSet[1:13]' (iter 9/10)
## INFO  [08:03:42.287] [mlr3] Applying learner 'regr.ranger' on task 'PredictionSet[1:13]' (iter 10/10)
## INFO  [08:03:42.306] [mlr3] Finished benchmark
## INFO  [08:03:42.340] [bbotk] Result of batch 2:
## INFO  [08:03:42.342] [bbotk]  max.depth num.trees  regr.rmse warnings errors runtime_learners
## INFO  [08:03:42.342] [bbotk]          3       132 0.01947626        0      0            0.103
## INFO  [08:03:42.342] [bbotk]                                 uhash
## INFO  [08:03:42.342] [bbotk]  3ed74968-689e-4609-9949-556c08a9b3f9
## INFO  [08:03:42.343] [bbotk] Evaluating 1 configuration(s)
## INFO  [08:03:42.365] [mlr3] Running benchmark with 10 resampling iterations
## INFO  [08:03:42.371] [mlr3] Applying learner 'regr.ranger' on task 'PredictionSet[1:13]' (iter 1/10)
## INFO  [08:03:42.409] [mlr3] Applying learner 'regr.ranger' on task 'PredictionSet[1:13]' (iter 2/10)
## INFO  [08:03:42.446] [mlr3] Applying learner 'regr.ranger' on task 'PredictionSet[1:13]' (iter 3/10)
## INFO  [08:03:42.484] [mlr3] Applying learner 'regr.ranger' on task 'PredictionSet[1:13]' (iter 4/10)
## INFO  [08:03:42.530] [mlr3] Applying learner 'regr.ranger' on task 'PredictionSet[1:13]' (iter 5/10)
## INFO  [08:03:42.573] [mlr3] Applying learner 'regr.ranger' on task 'PredictionSet[1:13]' (iter 6/10)
## INFO  [08:03:42.616] [mlr3] Applying learner 'regr.ranger' on task 'PredictionSet[1:13]' (iter 7/10)
## INFO  [08:03:42.657] [mlr3] Applying learner 'regr.ranger' on task 'PredictionSet[1:13]' (iter 8/10)
## INFO  [08:03:42.694] [mlr3] Applying learner 'regr.ranger' on task 'PredictionSet[1:13]' (iter 9/10)
## INFO  [08:03:42.731] [mlr3] Applying learner 'regr.ranger' on task 'PredictionSet[1:13]' (iter 10/10)
## INFO  [08:03:42.767] [mlr3] Finished benchmark
## INFO  [08:03:42.801] [bbotk] Result of batch 3:
## INFO  [08:03:42.802] [bbotk]  max.depth num.trees  regr.rmse warnings errors runtime_learners
## INFO  [08:03:42.802] [bbotk]         12       395 0.01119778        0      0            0.279
## INFO  [08:03:42.802] [bbotk]                                 uhash
## INFO  [08:03:42.802] [bbotk]  5613e4c3-f916-4bc0-9a13-976a7f499e74
## INFO  [08:03:42.804] [bbotk] Evaluating 1 configuration(s)
## INFO  [08:03:42.825] [mlr3] Running benchmark with 10 resampling iterations
## INFO  [08:03:42.830] [mlr3] Applying learner 'regr.ranger' on task 'PredictionSet[1:13]' (iter 1/10)
## INFO  [08:03:42.850] [mlr3] Applying learner 'regr.ranger' on task 'PredictionSet[1:13]' (iter 2/10)
## INFO  [08:03:42.871] [mlr3] Applying learner 'regr.ranger' on task 'PredictionSet[1:13]' (iter 3/10)
## INFO  [08:03:42.892] [mlr3] Applying learner 'regr.ranger' on task 'PredictionSet[1:13]' (iter 4/10)
## INFO  [08:03:42.912] [mlr3] Applying learner 'regr.ranger' on task 'PredictionSet[1:13]' (iter 5/10)
## INFO  [08:03:42.934] [mlr3] Applying learner 'regr.ranger' on task 'PredictionSet[1:13]' (iter 6/10)
## INFO  [08:03:42.954] [mlr3] Applying learner 'regr.ranger' on task 'PredictionSet[1:13]' (iter 7/10)
## INFO  [08:03:42.974] [mlr3] Applying learner 'regr.ranger' on task 'PredictionSet[1:13]' (iter 8/10)
## INFO  [08:03:42.993] [mlr3] Applying learner 'regr.ranger' on task 'PredictionSet[1:13]' (iter 9/10)
## INFO  [08:03:43.025] [mlr3] Applying learner 'regr.ranger' on task 'PredictionSet[1:13]' (iter 10/10)
## INFO  [08:03:43.048] [mlr3] Finished benchmark
## INFO  [08:03:43.087] [bbotk] Result of batch 4:
## INFO  [08:03:43.089] [bbotk]  max.depth num.trees  regr.rmse warnings errors runtime_learners
## INFO  [08:03:43.089] [bbotk]         13        79 0.01187452        0      0            0.108
## INFO  [08:03:43.089] [bbotk]                                 uhash
## INFO  [08:03:43.089] [bbotk]  f6425eff-12ab-41db-b9f3-5dad00e3112d
## INFO  [08:03:43.091] [bbotk] Evaluating 1 configuration(s)
## INFO  [08:03:43.114] [mlr3] Running benchmark with 10 resampling iterations
## INFO  [08:03:43.119] [mlr3] Applying learner 'regr.ranger' on task 'PredictionSet[1:13]' (iter 1/10)
## INFO  [08:03:43.142] [mlr3] Applying learner 'regr.ranger' on task 'PredictionSet[1:13]' (iter 2/10)
## INFO  [08:03:43.168] [mlr3] Applying learner 'regr.ranger' on task 'PredictionSet[1:13]' (iter 3/10)
## INFO  [08:03:43.192] [mlr3] Applying learner 'regr.ranger' on task 'PredictionSet[1:13]' (iter 4/10)
## INFO  [08:03:43.215] [mlr3] Applying learner 'regr.ranger' on task 'PredictionSet[1:13]' (iter 5/10)
## INFO  [08:03:43.241] [mlr3] Applying learner 'regr.ranger' on task 'PredictionSet[1:13]' (iter 6/10)
## INFO  [08:03:43.265] [mlr3] Applying learner 'regr.ranger' on task 'PredictionSet[1:13]' (iter 7/10)
## INFO  [08:03:43.288] [mlr3] Applying learner 'regr.ranger' on task 'PredictionSet[1:13]' (iter 8/10)
## INFO  [08:03:43.313] [mlr3] Applying learner 'regr.ranger' on task 'PredictionSet[1:13]' (iter 9/10)
## INFO  [08:03:43.338] [mlr3] Applying learner 'regr.ranger' on task 'PredictionSet[1:13]' (iter 10/10)
## INFO  [08:03:43.361] [mlr3] Finished benchmark
## INFO  [08:03:43.394] [bbotk] Result of batch 5:
## INFO  [08:03:43.396] [bbotk]  max.depth num.trees  regr.rmse warnings errors runtime_learners
## INFO  [08:03:43.396] [bbotk]         13       158 0.01097585        0      0            0.136
## INFO  [08:03:43.396] [bbotk]                                 uhash
## INFO  [08:03:43.396] [bbotk]  28774fa2-424e-4685-b9bf-24b88e89bc16
## INFO  [08:03:43.398] [bbotk] Evaluating 1 configuration(s)
## INFO  [08:03:43.433] [mlr3] Running benchmark with 10 resampling iterations
## INFO  [08:03:43.439] [mlr3] Applying learner 'regr.ranger' on task 'PredictionSet[1:13]' (iter 1/10)
## INFO  [08:03:43.483] [mlr3] Applying learner 'regr.ranger' on task 'PredictionSet[1:13]' (iter 2/10)
## INFO  [08:03:43.524] [mlr3] Applying learner 'regr.ranger' on task 'PredictionSet[1:13]' (iter 3/10)
## INFO  [08:03:43.565] [mlr3] Applying learner 'regr.ranger' on task 'PredictionSet[1:13]' (iter 4/10)
## INFO  [08:03:43.604] [mlr3] Applying learner 'regr.ranger' on task 'PredictionSet[1:13]' (iter 5/10)
## INFO  [08:03:43.645] [mlr3] Applying learner 'regr.ranger' on task 'PredictionSet[1:13]' (iter 6/10)
## INFO  [08:03:43.685] [mlr3] Applying learner 'regr.ranger' on task 'PredictionSet[1:13]' (iter 7/10)
## INFO  [08:03:43.725] [mlr3] Applying learner 'regr.ranger' on task 'PredictionSet[1:13]' (iter 8/10)
## INFO  [08:03:43.766] [mlr3] Applying learner 'regr.ranger' on task 'PredictionSet[1:13]' (iter 9/10)
## INFO  [08:03:43.805] [mlr3] Applying learner 'regr.ranger' on task 'PredictionSet[1:13]' (iter 10/10)
## INFO  [08:03:43.846] [mlr3] Finished benchmark
## INFO  [08:03:43.877] [bbotk] Result of batch 6:
## INFO  [08:03:43.879] [bbotk]  max.depth num.trees  regr.rmse warnings errors runtime_learners
## INFO  [08:03:43.879] [bbotk]          6       474 0.01182881        0      0            0.293
## INFO  [08:03:43.879] [bbotk]                                 uhash
## INFO  [08:03:43.879] [bbotk]  91c2baac-e85b-4f99-bfe8-2d1fc5715078
## INFO  [08:03:43.880] [bbotk] Evaluating 1 configuration(s)
## INFO  [08:03:43.903] [mlr3] Running benchmark with 10 resampling iterations
## INFO  [08:03:43.909] [mlr3] Applying learner 'regr.ranger' on task 'PredictionSet[1:13]' (iter 1/10)
## INFO  [08:03:43.926] [mlr3] Applying learner 'regr.ranger' on task 'PredictionSet[1:13]' (iter 2/10)
## INFO  [08:03:43.943] [mlr3] Applying learner 'regr.ranger' on task 'PredictionSet[1:13]' (iter 3/10)
## INFO  [08:03:43.970] [mlr3] Applying learner 'regr.ranger' on task 'PredictionSet[1:13]' (iter 4/10)
## INFO  [08:03:43.993] [mlr3] Applying learner 'regr.ranger' on task 'PredictionSet[1:13]' (iter 5/10)
## INFO  [08:03:44.013] [mlr3] Applying learner 'regr.ranger' on task 'PredictionSet[1:13]' (iter 6/10)
## INFO  [08:03:44.031] [mlr3] Applying learner 'regr.ranger' on task 'PredictionSet[1:13]' (iter 7/10)
## INFO  [08:03:44.048] [mlr3] Applying learner 'regr.ranger' on task 'PredictionSet[1:13]' (iter 8/10)
## INFO  [08:03:44.067] [mlr3] Applying learner 'regr.ranger' on task 'PredictionSet[1:13]' (iter 9/10)
## INFO  [08:03:44.083] [mlr3] Applying learner 'regr.ranger' on task 'PredictionSet[1:13]' (iter 10/10)
## INFO  [08:03:44.099] [mlr3] Finished benchmark
## INFO  [08:03:44.131] [bbotk] Result of batch 7:
## INFO  [08:03:44.133] [bbotk]  max.depth num.trees regr.rmse warnings errors runtime_learners
## INFO  [08:03:44.133] [bbotk]         13        27 0.0122371        0      0            0.071
## INFO  [08:03:44.133] [bbotk]                                 uhash
## INFO  [08:03:44.133] [bbotk]  f69aaff7-dc89-4307-a45d-ff76a01854f5
## INFO  [08:03:44.135] [bbotk] Evaluating 1 configuration(s)
## INFO  [08:03:44.157] [mlr3] Running benchmark with 10 resampling iterations
## INFO  [08:03:44.162] [mlr3] Applying learner 'regr.ranger' on task 'PredictionSet[1:13]' (iter 1/10)
## INFO  [08:03:44.192] [mlr3] Applying learner 'regr.ranger' on task 'PredictionSet[1:13]' (iter 2/10)
## INFO  [08:03:44.224] [mlr3] Applying learner 'regr.ranger' on task 'PredictionSet[1:13]' (iter 3/10)
## INFO  [08:03:44.255] [mlr3] Applying learner 'regr.ranger' on task 'PredictionSet[1:13]' (iter 4/10)
## INFO  [08:03:44.287] [mlr3] Applying learner 'regr.ranger' on task 'PredictionSet[1:13]' (iter 5/10)
## INFO  [08:03:44.318] [mlr3] Applying learner 'regr.ranger' on task 'PredictionSet[1:13]' (iter 6/10)
## INFO  [08:03:44.348] [mlr3] Applying learner 'regr.ranger' on task 'PredictionSet[1:13]' (iter 7/10)
## INFO  [08:03:44.390] [mlr3] Applying learner 'regr.ranger' on task 'PredictionSet[1:13]' (iter 8/10)
## INFO  [08:03:44.424] [mlr3] Applying learner 'regr.ranger' on task 'PredictionSet[1:13]' (iter 9/10)
## INFO  [08:03:44.461] [mlr3] Applying learner 'regr.ranger' on task 'PredictionSet[1:13]' (iter 10/10)
## INFO  [08:03:44.494] [mlr3] Finished benchmark
## INFO  [08:03:44.528] [bbotk] Result of batch 8:
## INFO  [08:03:44.530] [bbotk]  max.depth num.trees  regr.rmse warnings errors runtime_learners
## INFO  [08:03:44.530] [bbotk]          7       290 0.01135048        0      0             0.21
## INFO  [08:03:44.530] [bbotk]                                 uhash
## INFO  [08:03:44.530] [bbotk]  875b12a3-899d-454e-9073-9293df8bcf87
## INFO  [08:03:44.531] [bbotk] Evaluating 1 configuration(s)
## INFO  [08:03:44.553] [mlr3] Running benchmark with 10 resampling iterations
## INFO  [08:03:44.558] [mlr3] Applying learner 'regr.ranger' on task 'PredictionSet[1:13]' (iter 1/10)
## INFO  [08:03:44.576] [mlr3] Applying learner 'regr.ranger' on task 'PredictionSet[1:13]' (iter 2/10)
## INFO  [08:03:44.595] [mlr3] Applying learner 'regr.ranger' on task 'PredictionSet[1:13]' (iter 3/10)
## INFO  [08:03:44.613] [mlr3] Applying learner 'regr.ranger' on task 'PredictionSet[1:13]' (iter 4/10)
## INFO  [08:03:44.631] [mlr3] Applying learner 'regr.ranger' on task 'PredictionSet[1:13]' (iter 5/10)
## INFO  [08:03:44.649] [mlr3] Applying learner 'regr.ranger' on task 'PredictionSet[1:13]' (iter 6/10)
## INFO  [08:03:44.669] [mlr3] Applying learner 'regr.ranger' on task 'PredictionSet[1:13]' (iter 7/10)
## INFO  [08:03:44.687] [mlr3] Applying learner 'regr.ranger' on task 'PredictionSet[1:13]' (iter 8/10)
## INFO  [08:03:44.705] [mlr3] Applying learner 'regr.ranger' on task 'PredictionSet[1:13]' (iter 9/10)
## INFO  [08:03:44.723] [mlr3] Applying learner 'regr.ranger' on task 'PredictionSet[1:13]' (iter 10/10)
## INFO  [08:03:44.743] [mlr3] Finished benchmark
## INFO  [08:03:44.824] [bbotk] Result of batch 9:
## INFO  [08:03:44.826] [bbotk]  max.depth num.trees  regr.rmse warnings errors runtime_learners
## INFO  [08:03:44.826] [bbotk]         12        53 0.01019492        0      0            0.077
## INFO  [08:03:44.826] [bbotk]                                 uhash
## INFO  [08:03:44.826] [bbotk]  5ac85f4a-89cb-4438-a5cc-f1b120dfe61e
## INFO  [08:03:44.828] [bbotk] Evaluating 1 configuration(s)
## INFO  [08:03:44.858] [mlr3] Running benchmark with 10 resampling iterations
## INFO  [08:03:44.865] [mlr3] Applying learner 'regr.ranger' on task 'PredictionSet[1:13]' (iter 1/10)
## INFO  [08:03:44.894] [mlr3] Applying learner 'regr.ranger' on task 'PredictionSet[1:13]' (iter 2/10)
## INFO  [08:03:44.920] [mlr3] Applying learner 'regr.ranger' on task 'PredictionSet[1:13]' (iter 3/10)
## INFO  [08:03:44.946] [mlr3] Applying learner 'regr.ranger' on task 'PredictionSet[1:13]' (iter 4/10)
## INFO  [08:03:44.973] [mlr3] Applying learner 'regr.ranger' on task 'PredictionSet[1:13]' (iter 5/10)
## INFO  [08:03:44.997] [mlr3] Applying learner 'regr.ranger' on task 'PredictionSet[1:13]' (iter 6/10)
## INFO  [08:03:45.022] [mlr3] Applying learner 'regr.ranger' on task 'PredictionSet[1:13]' (iter 7/10)
## INFO  [08:03:45.049] [mlr3] Applying learner 'regr.ranger' on task 'PredictionSet[1:13]' (iter 8/10)
## INFO  [08:03:45.074] [mlr3] Applying learner 'regr.ranger' on task 'PredictionSet[1:13]' (iter 9/10)
## INFO  [08:03:45.099] [mlr3] Applying learner 'regr.ranger' on task 'PredictionSet[1:13]' (iter 10/10)
## INFO  [08:03:45.125] [mlr3] Finished benchmark
## INFO  [08:03:45.157] [bbotk] Result of batch 10:
## INFO  [08:03:45.158] [bbotk]  max.depth num.trees  regr.rmse warnings errors runtime_learners
## INFO  [08:03:45.158] [bbotk]          9       185 0.01116216        0      0            0.153
## INFO  [08:03:45.158] [bbotk]                                 uhash
## INFO  [08:03:45.158] [bbotk]  22a9d9e2-50e4-4a1b-be86-665b4ae72783
## INFO  [08:03:45.163] [bbotk] Finished optimizing after 10 evaluation(s)
## INFO  [08:03:45.164] [bbotk] Result:
## INFO  [08:03:45.165] [bbotk]  max.depth num.trees learner_param_vals  x_domain  regr.rmse
## INFO  [08:03:45.165] [bbotk]         12        53          <list[3]> <list[2]> 0.01019492
## INFO  [08:03:45.427] [bbotk] Starting to optimize 5 parameter(s) with '<TunerGridSearch>' and '<TerminatorEvals> [n_evals=10, k=0]'
## INFO  [08:03:45.435] [bbotk] Evaluating 1 configuration(s)
## INFO  [08:03:45.453] [mlr3] Running benchmark with 10 resampling iterations
## INFO  [08:03:45.458] [mlr3] Applying learner 'regr.svm' on task 'PredictionSet[1:13]' (iter 1/10)
## INFO  [08:03:45.475] [mlr3] Applying learner 'regr.svm' on task 'PredictionSet[1:13]' (iter 2/10)
## INFO  [08:03:45.493] [mlr3] Applying learner 'regr.svm' on task 'PredictionSet[1:13]' (iter 3/10)
## INFO  [08:03:45.510] [mlr3] Applying learner 'regr.svm' on task 'PredictionSet[1:13]' (iter 4/10)
## INFO  [08:03:45.526] [mlr3] Applying learner 'regr.svm' on task 'PredictionSet[1:13]' (iter 5/10)
## INFO  [08:03:45.542] [mlr3] Applying learner 'regr.svm' on task 'PredictionSet[1:13]' (iter 6/10)
## INFO  [08:03:45.570] [mlr3] Applying learner 'regr.svm' on task 'PredictionSet[1:13]' (iter 7/10)
## INFO  [08:03:45.591] [mlr3] Applying learner 'regr.svm' on task 'PredictionSet[1:13]' (iter 8/10)
## INFO  [08:03:45.610] [mlr3] Applying learner 'regr.svm' on task 'PredictionSet[1:13]' (iter 9/10)
## INFO  [08:03:45.631] [mlr3] Applying learner 'regr.svm' on task 'PredictionSet[1:13]' (iter 10/10)
## INFO  [08:03:45.652] [mlr3] Finished benchmark
## INFO  [08:03:45.682] [bbotk] Result of batch 1:
## INFO  [08:03:45.684] [bbotk]  tolerance gamma epsilon           type kernel regr.rmse warnings errors
## INFO  [08:03:45.684] [bbotk]  0.3862857    50     0.5 eps-regression radial  40348633        0      0
## INFO  [08:03:45.684] [bbotk]  runtime_learners                                uhash
## INFO  [08:03:45.684] [bbotk]              0.08 aa6578f6-28c6-46a8-97f8-fc1d82ba4f58
## INFO  [08:03:45.686] [bbotk] Evaluating 1 configuration(s)
## INFO  [08:03:45.702] [mlr3] Running benchmark with 10 resampling iterations
## INFO  [08:03:45.708] [mlr3] Applying learner 'regr.svm' on task 'PredictionSet[1:13]' (iter 1/10)
## INFO  [08:03:45.728] [mlr3] Applying learner 'regr.svm' on task 'PredictionSet[1:13]' (iter 2/10)
## INFO  [08:03:45.746] [mlr3] Applying learner 'regr.svm' on task 'PredictionSet[1:13]' (iter 3/10)
## INFO  [08:03:45.765] [mlr3] Applying learner 'regr.svm' on task 'PredictionSet[1:13]' (iter 4/10)
## INFO  [08:03:45.785] [mlr3] Applying learner 'regr.svm' on task 'PredictionSet[1:13]' (iter 5/10)
## INFO  [08:03:45.804] [mlr3] Applying learner 'regr.svm' on task 'PredictionSet[1:13]' (iter 6/10)
## INFO  [08:03:45.823] [mlr3] Applying learner 'regr.svm' on task 'PredictionSet[1:13]' (iter 7/10)
## INFO  [08:03:45.841] [mlr3] Applying learner 'regr.svm' on task 'PredictionSet[1:13]' (iter 8/10)
## INFO  [08:03:45.862] [mlr3] Applying learner 'regr.svm' on task 'PredictionSet[1:13]' (iter 9/10)
## INFO  [08:03:45.882] [mlr3] Applying learner 'regr.svm' on task 'PredictionSet[1:13]' (iter 10/10)
## INFO  [08:03:45.900] [mlr3] Finished benchmark
## INFO  [08:03:45.941] [bbotk] Result of batch 2:
## INFO  [08:03:45.944] [bbotk]  tolerance    gamma   epsilon           type kernel regr.rmse warnings errors
## INFO  [08:03:45.944] [bbotk]      0.001 39.28593 0.2714286 eps-regression radial  36354691        0      0
## INFO  [08:03:45.944] [bbotk]  runtime_learners                                uhash
## INFO  [08:03:45.944] [bbotk]             0.088 6b0674e8-6235-4f8a-b121-24dc6a44ebd5
## INFO  [08:03:45.946] [bbotk] Evaluating 1 configuration(s)
## INFO  [08:03:45.967] [mlr3] Running benchmark with 10 resampling iterations
## INFO  [08:03:45.973] [mlr3] Applying learner 'regr.svm' on task 'PredictionSet[1:13]' (iter 1/10)
## INFO  [08:03:45.996] [mlr3] Applying learner 'regr.svm' on task 'PredictionSet[1:13]' (iter 2/10)
## INFO  [08:03:46.020] [mlr3] Applying learner 'regr.svm' on task 'PredictionSet[1:13]' (iter 3/10)
## INFO  [08:03:46.040] [mlr3] Applying learner 'regr.svm' on task 'PredictionSet[1:13]' (iter 4/10)
## INFO  [08:03:46.059] [mlr3] Applying learner 'regr.svm' on task 'PredictionSet[1:13]' (iter 5/10)
## INFO  [08:03:46.078] [mlr3] Applying learner 'regr.svm' on task 'PredictionSet[1:13]' (iter 6/10)
## INFO  [08:03:46.097] [mlr3] Applying learner 'regr.svm' on task 'PredictionSet[1:13]' (iter 7/10)
## INFO  [08:03:46.116] [mlr3] Applying learner 'regr.svm' on task 'PredictionSet[1:13]' (iter 8/10)
## INFO  [08:03:46.134] [mlr3] Applying learner 'regr.svm' on task 'PredictionSet[1:13]' (iter 9/10)
## INFO  [08:03:46.153] [mlr3] Applying learner 'regr.svm' on task 'PredictionSet[1:13]' (iter 10/10)
## INFO  [08:03:46.173] [mlr3] Finished benchmark
## INFO  [08:03:46.204] [bbotk] Result of batch 3:
## INFO  [08:03:46.205] [bbotk]  tolerance    gamma   epsilon           type  kernel  regr.rmse warnings errors
## INFO  [08:03:46.205] [bbotk]  0.3862857 35.71457 0.8428571 eps-regression sigmoid 1375732619        0      0
## INFO  [08:03:46.205] [bbotk]  runtime_learners                                uhash
## INFO  [08:03:46.205] [bbotk]             0.089 d734d268-9b95-45ef-bf50-ee80e9885eb8
## INFO  [08:03:46.207] [bbotk] Evaluating 1 configuration(s)
## INFO  [08:03:46.223] [mlr3] Running benchmark with 10 resampling iterations
## INFO  [08:03:46.229] [mlr3] Applying learner 'regr.svm' on task 'PredictionSet[1:13]' (iter 1/10)
## INFO  [08:03:46.246] [mlr3] Applying learner 'regr.svm' on task 'PredictionSet[1:13]' (iter 2/10)
## INFO  [08:03:46.262] [mlr3] Applying learner 'regr.svm' on task 'PredictionSet[1:13]' (iter 3/10)
## INFO  [08:03:46.277] [mlr3] Applying learner 'regr.svm' on task 'PredictionSet[1:13]' (iter 4/10)
## INFO  [08:03:46.293] [mlr3] Applying learner 'regr.svm' on task 'PredictionSet[1:13]' (iter 5/10)
## INFO  [08:03:46.321] [mlr3] Applying learner 'regr.svm' on task 'PredictionSet[1:13]' (iter 6/10)
## INFO  [08:03:46.340] [mlr3] Applying learner 'regr.svm' on task 'PredictionSet[1:13]' (iter 7/10)
## INFO  [08:03:46.359] [mlr3] Applying learner 'regr.svm' on task 'PredictionSet[1:13]' (iter 8/10)
## INFO  [08:03:46.378] [mlr3] Applying learner 'regr.svm' on task 'PredictionSet[1:13]' (iter 9/10)
## INFO  [08:03:46.398] [mlr3] Applying learner 'regr.svm' on task 'PredictionSet[1:13]' (iter 10/10)
## INFO  [08:03:46.414] [mlr3] Finished benchmark
## INFO  [08:03:46.445] [bbotk] Result of batch 4:
## INFO  [08:03:46.447] [bbotk]  tolerance    gamma   epsilon           type kernel regr.rmse warnings errors
## INFO  [08:03:46.447] [bbotk]  0.8357857 10.71507 0.8428571 eps-regression radial  54700488        0      0
## INFO  [08:03:46.447] [bbotk]  runtime_learners                                uhash
## INFO  [08:03:46.447] [bbotk]              0.06 ea64ca51-7a6d-45d5-90ce-7a97b007f141
## INFO  [08:03:46.449] [bbotk] Evaluating 1 configuration(s)
## INFO  [08:03:46.466] [mlr3] Running benchmark with 10 resampling iterations
## INFO  [08:03:46.471] [mlr3] Applying learner 'regr.svm' on task 'PredictionSet[1:13]' (iter 1/10)
## INFO  [08:03:46.487] [mlr3] Applying learner 'regr.svm' on task 'PredictionSet[1:13]' (iter 2/10)
## INFO  [08:03:46.503] [mlr3] Applying learner 'regr.svm' on task 'PredictionSet[1:13]' (iter 3/10)
## INFO  [08:03:46.518] [mlr3] Applying learner 'regr.svm' on task 'PredictionSet[1:13]' (iter 4/10)
## INFO  [08:03:46.536] [mlr3] Applying learner 'regr.svm' on task 'PredictionSet[1:13]' (iter 5/10)
## INFO  [08:03:46.553] [mlr3] Applying learner 'regr.svm' on task 'PredictionSet[1:13]' (iter 6/10)
## INFO  [08:03:46.569] [mlr3] Applying learner 'regr.svm' on task 'PredictionSet[1:13]' (iter 7/10)
## INFO  [08:03:46.585] [mlr3] Applying learner 'regr.svm' on task 'PredictionSet[1:13]' (iter 8/10)
## INFO  [08:03:46.601] [mlr3] Applying learner 'regr.svm' on task 'PredictionSet[1:13]' (iter 9/10)
## INFO  [08:03:46.619] [mlr3] Applying learner 'regr.svm' on task 'PredictionSet[1:13]' (iter 10/10)
## INFO  [08:03:46.635] [mlr3] Finished benchmark
## INFO  [08:03:46.682] [bbotk] Result of batch 5:
## INFO  [08:03:46.684] [bbotk]  tolerance    gamma   epsilon           type kernel regr.rmse warnings errors
## INFO  [08:03:46.684] [bbotk]  0.7715714 3.572357 0.2714286 eps-regression radial  34854718        0      0
## INFO  [08:03:46.684] [bbotk]  runtime_learners                                uhash
## INFO  [08:03:46.684] [bbotk]             0.061 5cd0b267-d208-4162-8238-438f0c82de3f
## INFO  [08:03:46.687] [bbotk] Evaluating 1 configuration(s)
## INFO  [08:03:46.708] [mlr3] Running benchmark with 10 resampling iterations
## INFO  [08:03:46.714] [mlr3] Applying learner 'regr.svm' on task 'PredictionSet[1:13]' (iter 1/10)
## INFO  [08:03:46.732] [mlr3] Applying learner 'regr.svm' on task 'PredictionSet[1:13]' (iter 2/10)
## INFO  [08:03:46.749] [mlr3] Applying learner 'regr.svm' on task 'PredictionSet[1:13]' (iter 3/10)
## INFO  [08:03:46.767] [mlr3] Applying learner 'regr.svm' on task 'PredictionSet[1:13]' (iter 4/10)
## INFO  [08:03:46.783] [mlr3] Applying learner 'regr.svm' on task 'PredictionSet[1:13]' (iter 5/10)
## INFO  [08:03:46.799] [mlr3] Applying learner 'regr.svm' on task 'PredictionSet[1:13]' (iter 6/10)
## INFO  [08:03:46.814] [mlr3] Applying learner 'regr.svm' on task 'PredictionSet[1:13]' (iter 7/10)
## INFO  [08:03:46.831] [mlr3] Applying learner 'regr.svm' on task 'PredictionSet[1:13]' (iter 8/10)
## INFO  [08:03:46.848] [mlr3] Applying learner 'regr.svm' on task 'PredictionSet[1:13]' (iter 9/10)
## INFO  [08:03:46.863] [mlr3] Applying learner 'regr.svm' on task 'PredictionSet[1:13]' (iter 10/10)
## INFO  [08:03:46.878] [mlr3] Finished benchmark
## INFO  [08:03:46.912] [bbotk] Result of batch 6:
## INFO  [08:03:46.914] [bbotk]  tolerance    gamma   epsilon           type kernel regr.rmse warnings errors
## INFO  [08:03:46.914] [bbotk]  0.8357857 35.71457 0.7285714 eps-regression radial  53362225        0      0
## INFO  [08:03:46.914] [bbotk]  runtime_learners                                uhash
## INFO  [08:03:46.914] [bbotk]             0.059 0b89bd44-730c-45af-9abf-0dba1efb219a
## INFO  [08:03:46.915] [bbotk] Evaluating 1 configuration(s)
## INFO  [08:03:46.932] [mlr3] Running benchmark with 10 resampling iterations
## INFO  [08:03:46.937] [mlr3] Applying learner 'regr.svm' on task 'PredictionSet[1:13]' (iter 1/10)
## INFO  [08:03:46.954] [mlr3] Applying learner 'regr.svm' on task 'PredictionSet[1:13]' (iter 2/10)
## INFO  [08:03:46.970] [mlr3] Applying learner 'regr.svm' on task 'PredictionSet[1:13]' (iter 3/10)
## INFO  [08:03:46.999] [mlr3] Applying learner 'regr.svm' on task 'PredictionSet[1:13]' (iter 4/10)
## INFO  [08:03:47.020] [mlr3] Applying learner 'regr.svm' on task 'PredictionSet[1:13]' (iter 5/10)
## INFO  [08:03:47.040] [mlr3] Applying learner 'regr.svm' on task 'PredictionSet[1:13]' (iter 6/10)
## INFO  [08:03:47.063] [mlr3] Applying learner 'regr.svm' on task 'PredictionSet[1:13]' (iter 7/10)
## INFO  [08:03:47.082] [mlr3] Applying learner 'regr.svm' on task 'PredictionSet[1:13]' (iter 8/10)
## INFO  [08:03:47.100] [mlr3] Applying learner 'regr.svm' on task 'PredictionSet[1:13]' (iter 9/10)
## INFO  [08:03:47.117] [mlr3] Applying learner 'regr.svm' on task 'PredictionSet[1:13]' (iter 10/10)
## INFO  [08:03:47.135] [mlr3] Finished benchmark
## INFO  [08:03:47.167] [bbotk] Result of batch 7:
## INFO  [08:03:47.169] [bbotk]   tolerance    gamma epsilon           type kernel regr.rmse warnings errors
## INFO  [08:03:47.169] [bbotk]  0.06521429 14.28643     0.9 eps-regression radial  45626368        0      0
## INFO  [08:03:47.169] [bbotk]  runtime_learners                                uhash
## INFO  [08:03:47.169] [bbotk]             0.076 2e8d704d-14e7-4cd0-9afe-d3b9075a725f
## INFO  [08:03:47.170] [bbotk] Evaluating 1 configuration(s)
## INFO  [08:03:47.186] [mlr3] Running benchmark with 10 resampling iterations
## INFO  [08:03:47.191] [mlr3] Applying learner 'regr.svm' on task 'PredictionSet[1:13]' (iter 1/10)
## INFO  [08:03:47.209] [mlr3] Applying learner 'regr.svm' on task 'PredictionSet[1:13]' (iter 2/10)
## INFO  [08:03:47.226] [mlr3] Applying learner 'regr.svm' on task 'PredictionSet[1:13]' (iter 3/10)
## INFO  [08:03:47.243] [mlr3] Applying learner 'regr.svm' on task 'PredictionSet[1:13]' (iter 4/10)
## INFO  [08:03:47.259] [mlr3] Applying learner 'regr.svm' on task 'PredictionSet[1:13]' (iter 5/10)
## INFO  [08:03:47.276] [mlr3] Applying learner 'regr.svm' on task 'PredictionSet[1:13]' (iter 6/10)
## INFO  [08:03:47.294] [mlr3] Applying learner 'regr.svm' on task 'PredictionSet[1:13]' (iter 7/10)
## INFO  [08:03:47.311] [mlr3] Applying learner 'regr.svm' on task 'PredictionSet[1:13]' (iter 8/10)
## INFO  [08:03:47.328] [mlr3] Applying learner 'regr.svm' on task 'PredictionSet[1:13]' (iter 9/10)
## INFO  [08:03:47.355] [mlr3] Applying learner 'regr.svm' on task 'PredictionSet[1:13]' (iter 10/10)
## INFO  [08:03:47.377] [mlr3] Finished benchmark
## INFO  [08:03:47.417] [bbotk] Result of batch 8:
## INFO  [08:03:47.419] [bbotk]   tolerance gamma epsilon           type kernel regr.rmse warnings errors
## INFO  [08:03:47.419] [bbotk]  0.06521429 0.001     0.1 eps-regression radial  36752335        0      0
## INFO  [08:03:47.419] [bbotk]  runtime_learners                                uhash
## INFO  [08:03:47.419] [bbotk]             0.076 c9561659-26d3-4d96-a284-14b9dc93709d
## INFO  [08:03:47.421] [bbotk] Evaluating 1 configuration(s)
## INFO  [08:03:47.440] [mlr3] Running benchmark with 10 resampling iterations
## INFO  [08:03:47.445] [mlr3] Applying learner 'regr.svm' on task 'PredictionSet[1:13]' (iter 1/10)
## INFO  [08:03:47.463] [mlr3] Applying learner 'regr.svm' on task 'PredictionSet[1:13]' (iter 2/10)
## INFO  [08:03:47.480] [mlr3] Applying learner 'regr.svm' on task 'PredictionSet[1:13]' (iter 3/10)
## INFO  [08:03:47.498] [mlr3] Applying learner 'regr.svm' on task 'PredictionSet[1:13]' (iter 4/10)
## INFO  [08:03:47.517] [mlr3] Applying learner 'regr.svm' on task 'PredictionSet[1:13]' (iter 5/10)
## INFO  [08:03:47.534] [mlr3] Applying learner 'regr.svm' on task 'PredictionSet[1:13]' (iter 6/10)
## INFO  [08:03:47.551] [mlr3] Applying learner 'regr.svm' on task 'PredictionSet[1:13]' (iter 7/10)
## INFO  [08:03:47.568] [mlr3] Applying learner 'regr.svm' on task 'PredictionSet[1:13]' (iter 8/10)
## INFO  [08:03:47.587] [mlr3] Applying learner 'regr.svm' on task 'PredictionSet[1:13]' (iter 9/10)
## INFO  [08:03:47.605] [mlr3] Applying learner 'regr.svm' on task 'PredictionSet[1:13]' (iter 10/10)
## INFO  [08:03:47.622] [mlr3] Finished benchmark
## INFO  [08:03:47.654] [bbotk] Result of batch 9:
## INFO  [08:03:47.656] [bbotk]  tolerance    gamma   epsilon           type kernel regr.rmse warnings errors
## INFO  [08:03:47.656] [bbotk]  0.1294286 35.71457 0.7857143 eps-regression radial  43987129        0      0
## INFO  [08:03:47.656] [bbotk]  runtime_learners                                uhash
## INFO  [08:03:47.656] [bbotk]             0.074 057a8bb9-1495-418a-b544-8cd6412849fd
## INFO  [08:03:47.658] [bbotk] Evaluating 1 configuration(s)
## INFO  [08:03:47.675] [mlr3] Running benchmark with 10 resampling iterations
## INFO  [08:03:47.688] [mlr3] Applying learner 'regr.svm' on task 'PredictionSet[1:13]' (iter 1/10)
## INFO  [08:03:47.707] [mlr3] Applying learner 'regr.svm' on task 'PredictionSet[1:13]' (iter 2/10)
## INFO  [08:03:47.727] [mlr3] Applying learner 'regr.svm' on task 'PredictionSet[1:13]' (iter 3/10)
## INFO  [08:03:47.749] [mlr3] Applying learner 'regr.svm' on task 'PredictionSet[1:13]' (iter 4/10)
## INFO  [08:03:47.768] [mlr3] Applying learner 'regr.svm' on task 'PredictionSet[1:13]' (iter 5/10)
## INFO  [08:03:47.785] [mlr3] Applying learner 'regr.svm' on task 'PredictionSet[1:13]' (iter 6/10)
## INFO  [08:03:47.802] [mlr3] Applying learner 'regr.svm' on task 'PredictionSet[1:13]' (iter 7/10)
## INFO  [08:03:47.820] [mlr3] Applying learner 'regr.svm' on task 'PredictionSet[1:13]' (iter 8/10)
## INFO  [08:03:47.836] [mlr3] Applying learner 'regr.svm' on task 'PredictionSet[1:13]' (iter 9/10)
## INFO  [08:03:47.852] [mlr3] Applying learner 'regr.svm' on task 'PredictionSet[1:13]' (iter 10/10)
## INFO  [08:03:47.867] [mlr3] Finished benchmark
## INFO  [08:03:47.900] [bbotk] Result of batch 10:
## INFO  [08:03:47.902] [bbotk]  tolerance    gamma epsilon           type kernel regr.rmse warnings errors
## INFO  [08:03:47.902] [bbotk]  0.8357857 42.85729     0.5 eps-regression radial  48496643        0      0
## INFO  [08:03:47.902] [bbotk]  runtime_learners                                uhash
## INFO  [08:03:47.902] [bbotk]             0.063 5d8414f0-b62f-4785-83c1-3be1a438f107
## INFO  [08:03:47.907] [bbotk] Finished optimizing after 10 evaluation(s)
## INFO  [08:03:47.908] [bbotk] Result:
## INFO  [08:03:47.909] [bbotk]  tolerance    gamma   epsilon           type kernel learner_param_vals
## INFO  [08:03:47.909] [bbotk]  0.7715714 3.572357 0.2714286 eps-regression radial          <list[5]>
## INFO  [08:03:47.909] [bbotk]   x_domain regr.rmse
## INFO  [08:03:47.909] [bbotk]  <list[5]>  34854718
## INFO  [08:03:48.074] [bbotk] Starting to optimize 3 parameter(s) with '<TunerGridSearch>' and '<TerminatorEvals> [n_evals=10, k=0]'
## INFO  [08:03:48.078] [bbotk] Evaluating 1 configuration(s)
## INFO  [08:03:48.090] [mlr3] Running benchmark with 10 resampling iterations
## INFO  [08:03:48.095] [mlr3] Applying learner 'regr.kknn' on task 'PredictionSet[1:13]' (iter 1/10)
## INFO  [08:03:48.118] [mlr3] Applying learner 'regr.kknn' on task 'PredictionSet[1:13]' (iter 2/10)
## INFO  [08:03:48.138] [mlr3] Applying learner 'regr.kknn' on task 'PredictionSet[1:13]' (iter 3/10)
## INFO  [08:03:48.159] [mlr3] Applying learner 'regr.kknn' on task 'PredictionSet[1:13]' (iter 4/10)
## INFO  [08:03:48.180] [mlr3] Applying learner 'regr.kknn' on task 'PredictionSet[1:13]' (iter 5/10)
## INFO  [08:03:48.202] [mlr3] Applying learner 'regr.kknn' on task 'PredictionSet[1:13]' (iter 6/10)
## INFO  [08:03:48.222] [mlr3] Applying learner 'regr.kknn' on task 'PredictionSet[1:13]' (iter 7/10)
## INFO  [08:03:48.243] [mlr3] Applying learner 'regr.kknn' on task 'PredictionSet[1:13]' (iter 8/10)
## INFO  [08:03:48.265] [mlr3] Applying learner 'regr.kknn' on task 'PredictionSet[1:13]' (iter 9/10)
## INFO  [08:03:48.286] [mlr3] Applying learner 'regr.kknn' on task 'PredictionSet[1:13]' (iter 10/10)
## INFO  [08:03:48.306] [mlr3] Finished benchmark
## INFO  [08:03:48.337] [bbotk] Result of batch 1:
## INFO  [08:03:48.339] [bbotk]   k distance  kernel regr.rmse warnings errors runtime_learners
## INFO  [08:03:48.339] [bbotk]  15 17.15714 optimal  18747894        0      0            0.104
## INFO  [08:03:48.339] [bbotk]                                 uhash
## INFO  [08:03:48.339] [bbotk]  c3da40e2-b447-4fcc-b42b-d75ec7fc742f
## INFO  [08:03:48.348] [bbotk] Evaluating 1 configuration(s)
## INFO  [08:03:48.362] [mlr3] Running benchmark with 10 resampling iterations
## INFO  [08:03:48.369] [mlr3] Applying learner 'regr.kknn' on task 'PredictionSet[1:13]' (iter 1/10)
## INFO  [08:03:48.393] [mlr3] Applying learner 'regr.kknn' on task 'PredictionSet[1:13]' (iter 2/10)
## INFO  [08:03:48.421] [mlr3] Applying learner 'regr.kknn' on task 'PredictionSet[1:13]' (iter 3/10)
## INFO  [08:03:48.445] [mlr3] Applying learner 'regr.kknn' on task 'PredictionSet[1:13]' (iter 4/10)
## INFO  [08:03:48.466] [mlr3] Applying learner 'regr.kknn' on task 'PredictionSet[1:13]' (iter 5/10)
## INFO  [08:03:48.488] [mlr3] Applying learner 'regr.kknn' on task 'PredictionSet[1:13]' (iter 6/10)
## INFO  [08:03:48.509] [mlr3] Applying learner 'regr.kknn' on task 'PredictionSet[1:13]' (iter 7/10)
## INFO  [08:03:48.529] [mlr3] Applying learner 'regr.kknn' on task 'PredictionSet[1:13]' (iter 8/10)
## INFO  [08:03:48.550] [mlr3] Applying learner 'regr.kknn' on task 'PredictionSet[1:13]' (iter 9/10)
## INFO  [08:03:48.572] [mlr3] Applying learner 'regr.kknn' on task 'PredictionSet[1:13]' (iter 10/10)
## INFO  [08:03:48.592] [mlr3] Finished benchmark
## INFO  [08:03:48.623] [bbotk] Result of batch 2:
## INFO  [08:03:48.625] [bbotk]  k distance    kernel regr.rmse warnings errors runtime_learners
## INFO  [08:03:48.625] [bbotk]  1 2.942857 triweight  23632460        0      0             0.11
## INFO  [08:03:48.625] [bbotk]                                 uhash
## INFO  [08:03:48.625] [bbotk]  a6ea8ee6-a467-4135-b64a-1d50c768ee94
## INFO  [08:03:48.627] [bbotk] Evaluating 1 configuration(s)
## INFO  [08:03:48.639] [mlr3] Running benchmark with 10 resampling iterations
## INFO  [08:03:48.644] [mlr3] Applying learner 'regr.kknn' on task 'PredictionSet[1:13]' (iter 1/10)
## INFO  [08:03:48.665] [mlr3] Applying learner 'regr.kknn' on task 'PredictionSet[1:13]' (iter 2/10)
## INFO  [08:03:48.686] [mlr3] Applying learner 'regr.kknn' on task 'PredictionSet[1:13]' (iter 3/10)
## INFO  [08:03:48.718] [mlr3] Applying learner 'regr.kknn' on task 'PredictionSet[1:13]' (iter 4/10)
## INFO  [08:03:48.744] [mlr3] Applying learner 'regr.kknn' on task 'PredictionSet[1:13]' (iter 5/10)
## INFO  [08:03:48.768] [mlr3] Applying learner 'regr.kknn' on task 'PredictionSet[1:13]' (iter 6/10)
## INFO  [08:03:48.796] [mlr3] Applying learner 'regr.kknn' on task 'PredictionSet[1:13]' (iter 7/10)
## INFO  [08:03:48.819] [mlr3] Applying learner 'regr.kknn' on task 'PredictionSet[1:13]' (iter 8/10)
## INFO  [08:03:48.840] [mlr3] Applying learner 'regr.kknn' on task 'PredictionSet[1:13]' (iter 9/10)
## INFO  [08:03:48.862] [mlr3] Applying learner 'regr.kknn' on task 'PredictionSet[1:13]' (iter 10/10)
## INFO  [08:03:48.883] [mlr3] Finished benchmark
## INFO  [08:03:48.914] [bbotk] Result of batch 3:
## INFO  [08:03:48.916] [bbotk]   k distance    kernel regr.rmse warnings errors runtime_learners
## INFO  [08:03:48.916] [bbotk]  18 1.521429 triweight  19667382        0      0            0.122
## INFO  [08:03:48.916] [bbotk]                                 uhash
## INFO  [08:03:48.916] [bbotk]  2db35129-e552-4213-9cd8-2a62c2bf1632
## INFO  [08:03:48.917] [bbotk] Evaluating 1 configuration(s)
## INFO  [08:03:48.928] [mlr3] Running benchmark with 10 resampling iterations
## INFO  [08:03:48.934] [mlr3] Applying learner 'regr.kknn' on task 'PredictionSet[1:13]' (iter 1/10)
## INFO  [08:03:48.956] [mlr3] Applying learner 'regr.kknn' on task 'PredictionSet[1:13]' (iter 2/10)
## INFO  [08:03:48.977] [mlr3] Applying learner 'regr.kknn' on task 'PredictionSet[1:13]' (iter 3/10)
## INFO  [08:03:48.998] [mlr3] Applying learner 'regr.kknn' on task 'PredictionSet[1:13]' (iter 4/10)
## INFO  [08:03:49.021] [mlr3] Applying learner 'regr.kknn' on task 'PredictionSet[1:13]' (iter 5/10)
## INFO  [08:03:49.042] [mlr3] Applying learner 'regr.kknn' on task 'PredictionSet[1:13]' (iter 6/10)
## INFO  [08:03:49.062] [mlr3] Applying learner 'regr.kknn' on task 'PredictionSet[1:13]' (iter 7/10)
## INFO  [08:03:49.095] [mlr3] Applying learner 'regr.kknn' on task 'PredictionSet[1:13]' (iter 8/10)
## INFO  [08:03:49.122] [mlr3] Applying learner 'regr.kknn' on task 'PredictionSet[1:13]' (iter 9/10)
## INFO  [08:03:49.147] [mlr3] Applying learner 'regr.kknn' on task 'PredictionSet[1:13]' (iter 10/10)
## INFO  [08:03:49.174] [mlr3] Finished benchmark
## INFO  [08:03:49.207] [bbotk] Result of batch 4:
## INFO  [08:03:49.209] [bbotk]   k distance  kernel regr.rmse warnings errors runtime_learners
## INFO  [08:03:49.209] [bbotk]  12 11.47143 optimal  18229262        0      0            0.118
## INFO  [08:03:49.209] [bbotk]                                 uhash
## INFO  [08:03:49.209] [bbotk]  f464f40d-2e35-40dd-b6b6-64ae03f94472
## INFO  [08:03:49.210] [bbotk] Evaluating 1 configuration(s)
## INFO  [08:03:49.221] [mlr3] Running benchmark with 10 resampling iterations
## INFO  [08:03:49.226] [mlr3] Applying learner 'regr.kknn' on task 'PredictionSet[1:13]' (iter 1/10)
## INFO  [08:03:49.249] [mlr3] Applying learner 'regr.kknn' on task 'PredictionSet[1:13]' (iter 2/10)
## INFO  [08:03:49.269] [mlr3] Applying learner 'regr.kknn' on task 'PredictionSet[1:13]' (iter 3/10)
## INFO  [08:03:49.290] [mlr3] Applying learner 'regr.kknn' on task 'PredictionSet[1:13]' (iter 4/10)
## INFO  [08:03:49.311] [mlr3] Applying learner 'regr.kknn' on task 'PredictionSet[1:13]' (iter 5/10)
## INFO  [08:03:49.333] [mlr3] Applying learner 'regr.kknn' on task 'PredictionSet[1:13]' (iter 6/10)
## INFO  [08:03:49.353] [mlr3] Applying learner 'regr.kknn' on task 'PredictionSet[1:13]' (iter 7/10)
## INFO  [08:03:49.374] [mlr3] Applying learner 'regr.kknn' on task 'PredictionSet[1:13]' (iter 8/10)
## INFO  [08:03:49.397] [mlr3] Applying learner 'regr.kknn' on task 'PredictionSet[1:13]' (iter 9/10)
## INFO  [08:03:49.418] [mlr3] Applying learner 'regr.kknn' on task 'PredictionSet[1:13]' (iter 10/10)
## INFO  [08:03:49.446] [mlr3] Finished benchmark
## INFO  [08:03:49.489] [bbotk] Result of batch 5:
## INFO  [08:03:49.491] [bbotk]  k distance      kernel regr.rmse warnings errors runtime_learners
## INFO  [08:03:49.491] [bbotk]  6 18.57857 rectangular  21651122        0      0            0.103
## INFO  [08:03:49.491] [bbotk]                                 uhash
## INFO  [08:03:49.491] [bbotk]  3c65f714-1efd-405a-a25f-9b77bac0813e
## INFO  [08:03:49.493] [bbotk] Evaluating 1 configuration(s)
## INFO  [08:03:49.507] [mlr3] Running benchmark with 10 resampling iterations
## INFO  [08:03:49.513] [mlr3] Applying learner 'regr.kknn' on task 'PredictionSet[1:13]' (iter 1/10)
## INFO  [08:03:49.537] [mlr3] Applying learner 'regr.kknn' on task 'PredictionSet[1:13]' (iter 2/10)
## INFO  [08:03:49.558] [mlr3] Applying learner 'regr.kknn' on task 'PredictionSet[1:13]' (iter 3/10)
## INFO  [08:03:49.579] [mlr3] Applying learner 'regr.kknn' on task 'PredictionSet[1:13]' (iter 4/10)
## INFO  [08:03:49.599] [mlr3] Applying learner 'regr.kknn' on task 'PredictionSet[1:13]' (iter 5/10)
## INFO  [08:03:49.622] [mlr3] Applying learner 'regr.kknn' on task 'PredictionSet[1:13]' (iter 6/10)
## INFO  [08:03:49.642] [mlr3] Applying learner 'regr.kknn' on task 'PredictionSet[1:13]' (iter 7/10)
## INFO  [08:03:49.663] [mlr3] Applying learner 'regr.kknn' on task 'PredictionSet[1:13]' (iter 8/10)
## INFO  [08:03:49.684] [mlr3] Applying learner 'regr.kknn' on task 'PredictionSet[1:13]' (iter 9/10)
## INFO  [08:03:49.705] [mlr3] Applying learner 'regr.kknn' on task 'PredictionSet[1:13]' (iter 10/10)
## INFO  [08:03:49.726] [mlr3] Finished benchmark
## INFO  [08:03:49.759] [bbotk] Result of batch 6:
## INFO  [08:03:49.761] [bbotk]  k distance   kernel regr.rmse warnings errors runtime_learners
## INFO  [08:03:49.761] [bbotk]  2 15.73571 gaussian  17558972        0      0            0.103
## INFO  [08:03:49.761] [bbotk]                                 uhash
## INFO  [08:03:49.761] [bbotk]  e2f696a5-b4fe-4e8e-9451-5e5b4ebd9768
## INFO  [08:03:49.763] [bbotk] Evaluating 1 configuration(s)
## INFO  [08:03:49.774] [mlr3] Running benchmark with 10 resampling iterations
## INFO  [08:03:49.780] [mlr3] Applying learner 'regr.kknn' on task 'PredictionSet[1:13]' (iter 1/10)
## INFO  [08:03:49.810] [mlr3] Applying learner 'regr.kknn' on task 'PredictionSet[1:13]' (iter 2/10)
## INFO  [08:03:49.836] [mlr3] Applying learner 'regr.kknn' on task 'PredictionSet[1:13]' (iter 3/10)
## INFO  [08:03:49.862] [mlr3] Applying learner 'regr.kknn' on task 'PredictionSet[1:13]' (iter 4/10)
## INFO  [08:03:49.886] [mlr3] Applying learner 'regr.kknn' on task 'PredictionSet[1:13]' (iter 5/10)
## INFO  [08:03:49.909] [mlr3] Applying learner 'regr.kknn' on task 'PredictionSet[1:13]' (iter 6/10)
## INFO  [08:03:49.930] [mlr3] Applying learner 'regr.kknn' on task 'PredictionSet[1:13]' (iter 7/10)
## INFO  [08:03:49.951] [mlr3] Applying learner 'regr.kknn' on task 'PredictionSet[1:13]' (iter 8/10)
## INFO  [08:03:49.971] [mlr3] Applying learner 'regr.kknn' on task 'PredictionSet[1:13]' (iter 9/10)
## INFO  [08:03:49.993] [mlr3] Applying learner 'regr.kknn' on task 'PredictionSet[1:13]' (iter 10/10)
## INFO  [08:03:50.013] [mlr3] Finished benchmark
## INFO  [08:03:50.045] [bbotk] Result of batch 7:
## INFO  [08:03:50.047] [bbotk]  k distance  kernel regr.rmse warnings errors runtime_learners
## INFO  [08:03:50.047] [bbotk]  9 2.942857 optimal  19209967        0      0            0.111
## INFO  [08:03:50.047] [bbotk]                                 uhash
## INFO  [08:03:50.047] [bbotk]  6a8d9fd2-3d6e-4b0e-a5bc-c239ed26a40f
## INFO  [08:03:50.048] [bbotk] Evaluating 1 configuration(s)
## INFO  [08:03:50.061] [mlr3] Running benchmark with 10 resampling iterations
## INFO  [08:03:50.067] [mlr3] Applying learner 'regr.kknn' on task 'PredictionSet[1:13]' (iter 1/10)
## INFO  [08:03:50.087] [mlr3] Applying learner 'regr.kknn' on task 'PredictionSet[1:13]' (iter 2/10)
## INFO  [08:03:50.108] [mlr3] Applying learner 'regr.kknn' on task 'PredictionSet[1:13]' (iter 3/10)
## INFO  [08:03:50.129] [mlr3] Applying learner 'regr.kknn' on task 'PredictionSet[1:13]' (iter 4/10)
## INFO  [08:03:50.160] [mlr3] Applying learner 'regr.kknn' on task 'PredictionSet[1:13]' (iter 5/10)
## INFO  [08:03:50.186] [mlr3] Applying learner 'regr.kknn' on task 'PredictionSet[1:13]' (iter 6/10)
## INFO  [08:03:50.212] [mlr3] Applying learner 'regr.kknn' on task 'PredictionSet[1:13]' (iter 7/10)
## INFO  [08:03:50.238] [mlr3] Applying learner 'regr.kknn' on task 'PredictionSet[1:13]' (iter 8/10)
## INFO  [08:03:50.259] [mlr3] Applying learner 'regr.kknn' on task 'PredictionSet[1:13]' (iter 9/10)
## INFO  [08:03:50.281] [mlr3] Applying learner 'regr.kknn' on task 'PredictionSet[1:13]' (iter 10/10)
## INFO  [08:03:50.302] [mlr3] Finished benchmark
## INFO  [08:03:50.334] [bbotk] Result of batch 8:
## INFO  [08:03:50.335] [bbotk]  k distance     kernel regr.rmse warnings errors runtime_learners
## INFO  [08:03:50.335] [bbotk]  8 7.207143 triangular  18756595        0      0            0.119
## INFO  [08:03:50.335] [bbotk]                                 uhash
## INFO  [08:03:50.335] [bbotk]  70787b74-75ae-40c4-ad9c-395ffd6f6224
## INFO  [08:03:50.337] [bbotk] Evaluating 1 configuration(s)
## INFO  [08:03:50.348] [mlr3] Running benchmark with 10 resampling iterations
## INFO  [08:03:50.353] [mlr3] Applying learner 'regr.kknn' on task 'PredictionSet[1:13]' (iter 1/10)
## INFO  [08:03:50.376] [mlr3] Applying learner 'regr.kknn' on task 'PredictionSet[1:13]' (iter 2/10)
## INFO  [08:03:50.397] [mlr3] Applying learner 'regr.kknn' on task 'PredictionSet[1:13]' (iter 3/10)
## INFO  [08:03:50.417] [mlr3] Applying learner 'regr.kknn' on task 'PredictionSet[1:13]' (iter 4/10)
## INFO  [08:03:50.440] [mlr3] Applying learner 'regr.kknn' on task 'PredictionSet[1:13]' (iter 5/10)
## INFO  [08:03:50.462] [mlr3] Applying learner 'regr.kknn' on task 'PredictionSet[1:13]' (iter 6/10)
## INFO  [08:03:50.483] [mlr3] Applying learner 'regr.kknn' on task 'PredictionSet[1:13]' (iter 7/10)
## INFO  [08:03:50.514] [mlr3] Applying learner 'regr.kknn' on task 'PredictionSet[1:13]' (iter 8/10)
## INFO  [08:03:50.541] [mlr3] Applying learner 'regr.kknn' on task 'PredictionSet[1:13]' (iter 9/10)
## INFO  [08:03:50.566] [mlr3] Applying learner 'regr.kknn' on task 'PredictionSet[1:13]' (iter 10/10)
## INFO  [08:03:50.592] [mlr3] Finished benchmark
## INFO  [08:03:50.626] [bbotk] Result of batch 9:
## INFO  [08:03:50.628] [bbotk]   k distance kernel regr.rmse warnings errors runtime_learners
## INFO  [08:03:50.628] [bbotk]  16 1.521429   rank  22698614        0      0            0.121
## INFO  [08:03:50.628] [bbotk]                                 uhash
## INFO  [08:03:50.628] [bbotk]  cefda094-b271-4076-973e-af15002e075f
## INFO  [08:03:50.629] [bbotk] Evaluating 1 configuration(s)
## INFO  [08:03:50.640] [mlr3] Running benchmark with 10 resampling iterations
## INFO  [08:03:50.645] [mlr3] Applying learner 'regr.kknn' on task 'PredictionSet[1:13]' (iter 1/10)
## INFO  [08:03:50.667] [mlr3] Applying learner 'regr.kknn' on task 'PredictionSet[1:13]' (iter 2/10)
## INFO  [08:03:50.687] [mlr3] Applying learner 'regr.kknn' on task 'PredictionSet[1:13]' (iter 3/10)
## INFO  [08:03:50.708] [mlr3] Applying learner 'regr.kknn' on task 'PredictionSet[1:13]' (iter 4/10)
## INFO  [08:03:50.729] [mlr3] Applying learner 'regr.kknn' on task 'PredictionSet[1:13]' (iter 5/10)
## INFO  [08:03:50.751] [mlr3] Applying learner 'regr.kknn' on task 'PredictionSet[1:13]' (iter 6/10)
## INFO  [08:03:50.771] [mlr3] Applying learner 'regr.kknn' on task 'PredictionSet[1:13]' (iter 7/10)
## INFO  [08:03:50.792] [mlr3] Applying learner 'regr.kknn' on task 'PredictionSet[1:13]' (iter 8/10)
## INFO  [08:03:50.814] [mlr3] Applying learner 'regr.kknn' on task 'PredictionSet[1:13]' (iter 9/10)
## INFO  [08:03:50.835] [mlr3] Applying learner 'regr.kknn' on task 'PredictionSet[1:13]' (iter 10/10)
## INFO  [08:03:50.864] [mlr3] Finished benchmark
## INFO  [08:03:50.906] [bbotk] Result of batch 10:
## INFO  [08:03:50.908] [bbotk]   k distance    kernel regr.rmse warnings errors runtime_learners
## INFO  [08:03:50.908] [bbotk]  16 15.73571 triweight  18167489        0      0            0.114
## INFO  [08:03:50.908] [bbotk]                                 uhash
## INFO  [08:03:50.908] [bbotk]  e8c89379-9cd2-4c1e-96e8-0c57ed7e8a03
## INFO  [08:03:50.915] [bbotk] Finished optimizing after 10 evaluation(s)
## INFO  [08:03:50.915] [bbotk] Result:
## INFO  [08:03:50.917] [bbotk]  k distance   kernel learner_param_vals  x_domain regr.rmse
## INFO  [08:03:50.917] [bbotk]  2 15.73571 gaussian          <list[3]> <list[3]>  17558972
## INFO  [08:03:51.148] [bbotk] Starting to optimize 2 parameter(s) with '<TunerGridSearch>' and '<TerminatorEvals> [n_evals=10, k=0]'
## INFO  [08:03:51.151] [bbotk] Evaluating 1 configuration(s)
## INFO  [08:03:51.179] [mlr3] Running benchmark with 10 resampling iterations
## INFO  [08:03:51.185] [mlr3] Applying learner 'regr.ranger' on task 'PredictionSet[1:13]' (iter 1/10)
## INFO  [08:03:51.232] [mlr3] Applying learner 'regr.ranger' on task 'PredictionSet[1:13]' (iter 2/10)
## INFO  [08:03:51.279] [mlr3] Applying learner 'regr.ranger' on task 'PredictionSet[1:13]' (iter 3/10)
## INFO  [08:03:51.324] [mlr3] Applying learner 'regr.ranger' on task 'PredictionSet[1:13]' (iter 4/10)
## INFO  [08:03:51.370] [mlr3] Applying learner 'regr.ranger' on task 'PredictionSet[1:13]' (iter 5/10)
## INFO  [08:03:51.417] [mlr3] Applying learner 'regr.ranger' on task 'PredictionSet[1:13]' (iter 6/10)
## INFO  [08:03:51.461] [mlr3] Applying learner 'regr.ranger' on task 'PredictionSet[1:13]' (iter 7/10)
## INFO  [08:03:51.507] [mlr3] Applying learner 'regr.ranger' on task 'PredictionSet[1:13]' (iter 8/10)
## INFO  [08:03:51.552] [mlr3] Applying learner 'regr.ranger' on task 'PredictionSet[1:13]' (iter 9/10)
## INFO  [08:03:51.597] [mlr3] Applying learner 'regr.ranger' on task 'PredictionSet[1:13]' (iter 10/10)
## INFO  [08:03:51.644] [mlr3] Finished benchmark
## INFO  [08:03:51.674] [bbotk] Result of batch 1:
## INFO  [08:03:51.675] [bbotk]  max.depth num.trees regr.rmse warnings errors runtime_learners
## INFO  [08:03:51.675] [bbotk]         15       422  19170021        0      0             0.35
## INFO  [08:03:51.675] [bbotk]                                 uhash
## INFO  [08:03:51.675] [bbotk]  08598586-57ae-46c5-aacf-f582c068d112
## INFO  [08:03:51.677] [bbotk] Evaluating 1 configuration(s)
## INFO  [08:03:51.713] [mlr3] Running benchmark with 10 resampling iterations
## INFO  [08:03:51.720] [mlr3] Applying learner 'regr.ranger' on task 'PredictionSet[1:13]' (iter 1/10)
## INFO  [08:03:51.766] [mlr3] Applying learner 'regr.ranger' on task 'PredictionSet[1:13]' (iter 2/10)
## INFO  [08:03:51.812] [mlr3] Applying learner 'regr.ranger' on task 'PredictionSet[1:13]' (iter 3/10)
## INFO  [08:03:51.857] [mlr3] Applying learner 'regr.ranger' on task 'PredictionSet[1:13]' (iter 4/10)
## INFO  [08:03:51.901] [mlr3] Applying learner 'regr.ranger' on task 'PredictionSet[1:13]' (iter 5/10)
## INFO  [08:03:51.944] [mlr3] Applying learner 'regr.ranger' on task 'PredictionSet[1:13]' (iter 6/10)
## INFO  [08:03:51.985] [mlr3] Applying learner 'regr.ranger' on task 'PredictionSet[1:13]' (iter 7/10)
## INFO  [08:03:52.027] [mlr3] Applying learner 'regr.ranger' on task 'PredictionSet[1:13]' (iter 8/10)
## INFO  [08:03:52.068] [mlr3] Applying learner 'regr.ranger' on task 'PredictionSet[1:13]' (iter 9/10)
## INFO  [08:03:52.110] [mlr3] Applying learner 'regr.ranger' on task 'PredictionSet[1:13]' (iter 10/10)
## INFO  [08:03:52.150] [mlr3] Finished benchmark
## INFO  [08:03:52.183] [bbotk] Result of batch 2:
## INFO  [08:03:52.185] [bbotk]  max.depth num.trees regr.rmse warnings errors runtime_learners
## INFO  [08:03:52.185] [bbotk]         20       369  18932395        0      0            0.313
## INFO  [08:03:52.185] [bbotk]                                 uhash
## INFO  [08:03:52.185] [bbotk]  f15570e5-e4eb-4742-8ea4-57c6d9013c73
## INFO  [08:03:52.186] [bbotk] Evaluating 1 configuration(s)
## INFO  [08:03:52.208] [mlr3] Running benchmark with 10 resampling iterations
## INFO  [08:03:52.214] [mlr3] Applying learner 'regr.ranger' on task 'PredictionSet[1:13]' (iter 1/10)
## INFO  [08:03:52.256] [mlr3] Applying learner 'regr.ranger' on task 'PredictionSet[1:13]' (iter 2/10)
## INFO  [08:03:52.297] [mlr3] Applying learner 'regr.ranger' on task 'PredictionSet[1:13]' (iter 3/10)
## INFO  [08:03:52.350] [mlr3] Applying learner 'regr.ranger' on task 'PredictionSet[1:13]' (iter 4/10)
## INFO  [08:03:52.398] [mlr3] Applying learner 'regr.ranger' on task 'PredictionSet[1:13]' (iter 5/10)
## INFO  [08:03:52.444] [mlr3] Applying learner 'regr.ranger' on task 'PredictionSet[1:13]' (iter 6/10)
## INFO  [08:03:52.491] [mlr3] Applying learner 'regr.ranger' on task 'PredictionSet[1:13]' (iter 7/10)
## INFO  [08:03:52.537] [mlr3] Applying learner 'regr.ranger' on task 'PredictionSet[1:13]' (iter 8/10)
## INFO  [08:03:52.579] [mlr3] Applying learner 'regr.ranger' on task 'PredictionSet[1:13]' (iter 9/10)
## INFO  [08:03:52.624] [mlr3] Applying learner 'regr.ranger' on task 'PredictionSet[1:13]' (iter 10/10)
## INFO  [08:03:52.666] [mlr3] Finished benchmark
## INFO  [08:03:52.699] [bbotk] Result of batch 3:
## INFO  [08:03:52.701] [bbotk]  max.depth num.trees regr.rmse warnings errors runtime_learners
## INFO  [08:03:52.701] [bbotk]         16       369  18592688        0      0            0.322
## INFO  [08:03:52.701] [bbotk]                                 uhash
## INFO  [08:03:52.701] [bbotk]  d848ae08-6739-4a7a-b092-7ba3c5fa6f29
## INFO  [08:03:52.703] [bbotk] Evaluating 1 configuration(s)
## INFO  [08:03:52.725] [mlr3] Running benchmark with 10 resampling iterations
## INFO  [08:03:52.730] [mlr3] Applying learner 'regr.ranger' on task 'PredictionSet[1:13]' (iter 1/10)
## INFO  [08:03:52.766] [mlr3] Applying learner 'regr.ranger' on task 'PredictionSet[1:13]' (iter 2/10)
## INFO  [08:03:52.801] [mlr3] Applying learner 'regr.ranger' on task 'PredictionSet[1:13]' (iter 3/10)
## INFO  [08:03:52.839] [mlr3] Applying learner 'regr.ranger' on task 'PredictionSet[1:13]' (iter 4/10)
## INFO  [08:03:52.873] [mlr3] Applying learner 'regr.ranger' on task 'PredictionSet[1:13]' (iter 5/10)
## INFO  [08:03:52.909] [mlr3] Applying learner 'regr.ranger' on task 'PredictionSet[1:13]' (iter 6/10)
## INFO  [08:03:52.976] [mlr3] Applying learner 'regr.ranger' on task 'PredictionSet[1:13]' (iter 7/10)
## INFO  [08:03:53.020] [mlr3] Applying learner 'regr.ranger' on task 'PredictionSet[1:13]' (iter 8/10)
## INFO  [08:03:53.060] [mlr3] Applying learner 'regr.ranger' on task 'PredictionSet[1:13]' (iter 9/10)
## INFO  [08:03:53.099] [mlr3] Applying learner 'regr.ranger' on task 'PredictionSet[1:13]' (iter 10/10)
## INFO  [08:03:53.139] [mlr3] Finished benchmark
## INFO  [08:03:53.177] [bbotk] Result of batch 4:
## INFO  [08:03:53.179] [bbotk]  max.depth num.trees regr.rmse warnings errors runtime_learners
## INFO  [08:03:53.179] [bbotk]          3       500  25173140        0      0            0.252
## INFO  [08:03:53.179] [bbotk]                                 uhash
## INFO  [08:03:53.179] [bbotk]  8abec120-cffc-4c17-8c6a-cbe4760d5ec5
## INFO  [08:03:53.181] [bbotk] Evaluating 1 configuration(s)
## INFO  [08:03:53.204] [mlr3] Running benchmark with 10 resampling iterations
## INFO  [08:03:53.210] [mlr3] Applying learner 'regr.ranger' on task 'PredictionSet[1:13]' (iter 1/10)
## INFO  [08:03:53.227] [mlr3] Applying learner 'regr.ranger' on task 'PredictionSet[1:13]' (iter 2/10)
## INFO  [08:03:53.242] [mlr3] Applying learner 'regr.ranger' on task 'PredictionSet[1:13]' (iter 3/10)
## INFO  [08:03:53.257] [mlr3] Applying learner 'regr.ranger' on task 'PredictionSet[1:13]' (iter 4/10)
## INFO  [08:03:53.272] [mlr3] Applying learner 'regr.ranger' on task 'PredictionSet[1:13]' (iter 5/10)
## INFO  [08:03:53.289] [mlr3] Applying learner 'regr.ranger' on task 'PredictionSet[1:13]' (iter 6/10)
## INFO  [08:03:53.305] [mlr3] Applying learner 'regr.ranger' on task 'PredictionSet[1:13]' (iter 7/10)
## INFO  [08:03:53.320] [mlr3] Applying learner 'regr.ranger' on task 'PredictionSet[1:13]' (iter 8/10)
## INFO  [08:03:53.335] [mlr3] Applying learner 'regr.ranger' on task 'PredictionSet[1:13]' (iter 9/10)
## INFO  [08:03:53.351] [mlr3] Applying learner 'regr.ranger' on task 'PredictionSet[1:13]' (iter 10/10)
## INFO  [08:03:53.368] [mlr3] Finished benchmark
## INFO  [08:03:53.410] [bbotk] Result of batch 5:
## INFO  [08:03:53.413] [bbotk]  max.depth num.trees regr.rmse warnings errors runtime_learners
## INFO  [08:03:53.413] [bbotk]          3         1  32029122        0      0            0.049
## INFO  [08:03:53.413] [bbotk]                                 uhash
## INFO  [08:03:53.413] [bbotk]  b956b9b5-a775-405f-8024-5df3b3c6980a
## INFO  [08:03:53.414] [bbotk] Evaluating 1 configuration(s)
## INFO  [08:03:53.446] [mlr3] Running benchmark with 10 resampling iterations
## INFO  [08:03:53.452] [mlr3] Applying learner 'regr.ranger' on task 'PredictionSet[1:13]' (iter 1/10)
## INFO  [08:03:53.507] [mlr3] Applying learner 'regr.ranger' on task 'PredictionSet[1:13]' (iter 2/10)
## INFO  [08:03:53.561] [mlr3] Applying learner 'regr.ranger' on task 'PredictionSet[1:13]' (iter 3/10)
## INFO  [08:03:53.612] [mlr3] Applying learner 'regr.ranger' on task 'PredictionSet[1:13]' (iter 4/10)
## INFO  [08:03:53.663] [mlr3] Applying learner 'regr.ranger' on task 'PredictionSet[1:13]' (iter 5/10)
## INFO  [08:03:53.712] [mlr3] Applying learner 'regr.ranger' on task 'PredictionSet[1:13]' (iter 6/10)
## INFO  [08:03:53.763] [mlr3] Applying learner 'regr.ranger' on task 'PredictionSet[1:13]' (iter 7/10)
## INFO  [08:03:53.813] [mlr3] Applying learner 'regr.ranger' on task 'PredictionSet[1:13]' (iter 8/10)
## INFO  [08:03:53.861] [mlr3] Applying learner 'regr.ranger' on task 'PredictionSet[1:13]' (iter 9/10)
## INFO  [08:03:53.911] [mlr3] Applying learner 'regr.ranger' on task 'PredictionSet[1:13]' (iter 10/10)
## INFO  [08:03:53.959] [mlr3] Finished benchmark
## INFO  [08:03:53.992] [bbotk] Result of batch 6:
## INFO  [08:03:53.994] [bbotk]  max.depth num.trees regr.rmse warnings errors runtime_learners
## INFO  [08:03:53.994] [bbotk]          9       474  18591152        0      0            0.387
## INFO  [08:03:53.994] [bbotk]                                 uhash
## INFO  [08:03:53.994] [bbotk]  65fb813b-f09b-46a9-9efe-22d6e0a7e49c
## INFO  [08:03:53.996] [bbotk] Evaluating 1 configuration(s)
## INFO  [08:03:54.018] [mlr3] Running benchmark with 10 resampling iterations
## INFO  [08:03:54.024] [mlr3] Applying learner 'regr.ranger' on task 'PredictionSet[1:13]' (iter 1/10)
## INFO  [08:03:54.068] [mlr3] Applying learner 'regr.ranger' on task 'PredictionSet[1:13]' (iter 2/10)
## INFO  [08:03:54.124] [mlr3] Applying learner 'regr.ranger' on task 'PredictionSet[1:13]' (iter 3/10)
## INFO  [08:03:54.172] [mlr3] Applying learner 'regr.ranger' on task 'PredictionSet[1:13]' (iter 4/10)
## INFO  [08:03:54.220] [mlr3] Applying learner 'regr.ranger' on task 'PredictionSet[1:13]' (iter 5/10)
## INFO  [08:03:54.270] [mlr3] Applying learner 'regr.ranger' on task 'PredictionSet[1:13]' (iter 6/10)
## INFO  [08:03:54.316] [mlr3] Applying learner 'regr.ranger' on task 'PredictionSet[1:13]' (iter 7/10)
## INFO  [08:03:54.365] [mlr3] Applying learner 'regr.ranger' on task 'PredictionSet[1:13]' (iter 8/10)
## INFO  [08:03:54.412] [mlr3] Applying learner 'regr.ranger' on task 'PredictionSet[1:13]' (iter 9/10)
## INFO  [08:03:54.456] [mlr3] Applying learner 'regr.ranger' on task 'PredictionSet[1:13]' (iter 10/10)
## INFO  [08:03:54.501] [mlr3] Finished benchmark
## INFO  [08:03:54.532] [bbotk] Result of batch 7:
## INFO  [08:03:54.534] [bbotk]  max.depth num.trees regr.rmse warnings errors runtime_learners
## INFO  [08:03:54.534] [bbotk]         15       395  18760082        0      0            0.342
## INFO  [08:03:54.534] [bbotk]                                 uhash
## INFO  [08:03:54.534] [bbotk]  3c3b986e-9df4-414b-a489-15425668aa7e
## INFO  [08:03:54.536] [bbotk] Evaluating 1 configuration(s)
## INFO  [08:03:54.559] [mlr3] Running benchmark with 10 resampling iterations
## INFO  [08:03:54.565] [mlr3] Applying learner 'regr.ranger' on task 'PredictionSet[1:13]' (iter 1/10)
## INFO  [08:03:54.611] [mlr3] Applying learner 'regr.ranger' on task 'PredictionSet[1:13]' (iter 2/10)
## INFO  [08:03:54.658] [mlr3] Applying learner 'regr.ranger' on task 'PredictionSet[1:13]' (iter 3/10)
## INFO  [08:03:54.705] [mlr3] Applying learner 'regr.ranger' on task 'PredictionSet[1:13]' (iter 4/10)
## INFO  [08:03:54.752] [mlr3] Applying learner 'regr.ranger' on task 'PredictionSet[1:13]' (iter 5/10)
## INFO  [08:03:54.800] [mlr3] Applying learner 'regr.ranger' on task 'PredictionSet[1:13]' (iter 6/10)
## INFO  [08:03:54.846] [mlr3] Applying learner 'regr.ranger' on task 'PredictionSet[1:13]' (iter 7/10)
## INFO  [08:03:54.893] [mlr3] Applying learner 'regr.ranger' on task 'PredictionSet[1:13]' (iter 8/10)
## INFO  [08:03:54.955] [mlr3] Applying learner 'regr.ranger' on task 'PredictionSet[1:13]' (iter 9/10)
## INFO  [08:03:55.006] [mlr3] Applying learner 'regr.ranger' on task 'PredictionSet[1:13]' (iter 10/10)
## INFO  [08:03:55.058] [mlr3] Finished benchmark
## INFO  [08:03:55.101] [bbotk] Result of batch 8:
## INFO  [08:03:55.103] [bbotk]  max.depth num.trees regr.rmse warnings errors runtime_learners
## INFO  [08:03:55.103] [bbotk]          8       448  18903059        0      0            0.365
## INFO  [08:03:55.103] [bbotk]                                 uhash
## INFO  [08:03:55.103] [bbotk]  368d11da-fec7-4567-9eb7-4b8e8728a0b2
## INFO  [08:03:55.105] [bbotk] Evaluating 1 configuration(s)
## INFO  [08:03:55.129] [mlr3] Running benchmark with 10 resampling iterations
## INFO  [08:03:55.134] [mlr3] Applying learner 'regr.ranger' on task 'PredictionSet[1:13]' (iter 1/10)
## INFO  [08:03:55.171] [mlr3] Applying learner 'regr.ranger' on task 'PredictionSet[1:13]' (iter 2/10)
## INFO  [08:03:55.204] [mlr3] Applying learner 'regr.ranger' on task 'PredictionSet[1:13]' (iter 3/10)
## INFO  [08:03:55.240] [mlr3] Applying learner 'regr.ranger' on task 'PredictionSet[1:13]' (iter 4/10)
## INFO  [08:03:55.274] [mlr3] Applying learner 'regr.ranger' on task 'PredictionSet[1:13]' (iter 5/10)
## INFO  [08:03:55.309] [mlr3] Applying learner 'regr.ranger' on task 'PredictionSet[1:13]' (iter 6/10)
## INFO  [08:03:55.343] [mlr3] Applying learner 'regr.ranger' on task 'PredictionSet[1:13]' (iter 7/10)
## INFO  [08:03:55.377] [mlr3] Applying learner 'regr.ranger' on task 'PredictionSet[1:13]' (iter 8/10)
## INFO  [08:03:55.412] [mlr3] Applying learner 'regr.ranger' on task 'PredictionSet[1:13]' (iter 9/10)
## INFO  [08:03:55.446] [mlr3] Applying learner 'regr.ranger' on task 'PredictionSet[1:13]' (iter 10/10)
## INFO  [08:03:55.481] [mlr3] Finished benchmark
## INFO  [08:03:55.522] [bbotk] Result of batch 9:
## INFO  [08:03:55.525] [bbotk]  max.depth num.trees regr.rmse warnings errors runtime_learners
## INFO  [08:03:55.525] [bbotk]          0       264  18807037        0      0            0.235
## INFO  [08:03:55.525] [bbotk]                                 uhash
## INFO  [08:03:55.525] [bbotk]  75057e2c-5078-45c6-bd6f-b040fb76343b
## INFO  [08:03:55.527] [bbotk] Evaluating 1 configuration(s)
## INFO  [08:03:55.558] [mlr3] Running benchmark with 10 resampling iterations
## INFO  [08:03:55.565] [mlr3] Applying learner 'regr.ranger' on task 'PredictionSet[1:13]' (iter 1/10)
## INFO  [08:03:55.601] [mlr3] Applying learner 'regr.ranger' on task 'PredictionSet[1:13]' (iter 2/10)
## INFO  [08:03:55.639] [mlr3] Applying learner 'regr.ranger' on task 'PredictionSet[1:13]' (iter 3/10)
## INFO  [08:03:55.675] [mlr3] Applying learner 'regr.ranger' on task 'PredictionSet[1:13]' (iter 4/10)
## INFO  [08:03:55.709] [mlr3] Applying learner 'regr.ranger' on task 'PredictionSet[1:13]' (iter 5/10)
## INFO  [08:03:55.741] [mlr3] Applying learner 'regr.ranger' on task 'PredictionSet[1:13]' (iter 6/10)
## INFO  [08:03:55.775] [mlr3] Applying learner 'regr.ranger' on task 'PredictionSet[1:13]' (iter 7/10)
## INFO  [08:03:55.807] [mlr3] Applying learner 'regr.ranger' on task 'PredictionSet[1:13]' (iter 8/10)
## INFO  [08:03:55.841] [mlr3] Applying learner 'regr.ranger' on task 'PredictionSet[1:13]' (iter 9/10)
## INFO  [08:03:55.873] [mlr3] Applying learner 'regr.ranger' on task 'PredictionSet[1:13]' (iter 10/10)
## INFO  [08:03:55.905] [mlr3] Finished benchmark
## INFO  [08:03:55.938] [bbotk] Result of batch 10:
## INFO  [08:03:55.940] [bbotk]  max.depth num.trees regr.rmse warnings errors runtime_learners
## INFO  [08:03:55.940] [bbotk]         11       237  18908760        0      0            0.224
## INFO  [08:03:55.940] [bbotk]                                 uhash
## INFO  [08:03:55.940] [bbotk]  f4e41894-6f7a-4606-8308-bf0c5cee7d96
## INFO  [08:03:55.945] [bbotk] Finished optimizing after 10 evaluation(s)
## INFO  [08:03:55.946] [bbotk] Result:
## INFO  [08:03:55.947] [bbotk]  max.depth num.trees learner_param_vals  x_domain regr.rmse
## INFO  [08:03:55.947] [bbotk]          9       474          <list[3]> <list[2]>  18591152
```

## Evolutionary Algorithm

Here, we start the evolutionary algorithm. First, we initialize and
train with the overall data using the best model from above. Then, we
have two setting for the EA and use the GA package to optimize towards
them.

```
  ######start GA with help of MLresult
  EADataSet <- list()
  for(l in 1:12){
    indexOfOutput <- l+19
    EADataSet[[indexOfOutput]] <- data.frame(fullDataSet[[3]][2], fullDataSet[[3]][4:11], fullDataSet[[3]][13:14], fullDataSet[[3]][19], fullDataSet[[3]][indexOfOutput])
    taskEA = as_task_regr(EADataSet[[indexOfOutput]][1:13], target = outputNames[l])
    taskEA$col_roles$stratum = "SEQUENCE"
    taskEA$col_roles$feature <- c("EPI.FACTOR", "ETL", "READOUT.DURATION", "REFERENCE.LINES", "REFOCUSSING.ANGLE", "TE", "TR", "numberOfColumns", "numberOfRows", "MEASUREMENTS", "NUMBER.PRESCANS")
    MLresult[[l]][[3]]$train(taskEA)
  }  

    REALLYHIGHVALUE <- 10000000
  ps <- 50
  is = 100
  
  cind <- 1
  cgen <- 1
  best <- -REALLYHIGHVALUE
  total <- 0
  collectData <- data.frame(num=rep(NA, 100), num=rep("",100))
  
    #####first setting
  REALLYHIGHVALUE <- 10000
  mainFitness <- function(EPI.FACTOR, ETL, READOUT.DURATION, REFERENCE.LINES, REFOCUSSING.ANGLE, TE, TR, numberOfColumns, numberOfRows, MEASUREMENTS, NUMBER.PRESCANS){
    
    features <- data.frame(EPI.FACTOR, ETL, READOUT.DURATION, REFERENCE.LINES, REFOCUSSING.ANGLE, TE, TR, numberOfColumns, numberOfRows, MEASUREMENTS, NUMBER.PRESCANS)
    SNR_CSF_value <- MLresult[[4]][[3]]$predict_newdata(features)
    SNR_WM_value <- MLresult[[6]][[3]]$predict_newdata(features)
    SNR_GM_value <- MLresult[[5]][[3]]$predict_newdata(features)
    TA_value <- MLresult[[12]][[3]]$predict_newdata(features)
    GHOST_value <- MLresult[[7]][[3]]$predict_newdata(features)
    MOTION_value <- MLresult[[10]][[3]]$predict_newdata(features)
    DISTORTION_value <- MLresult[[11]][[3]]$predict_newdata(features)
    GWC_value <- MLresult[[1]][[3]]$predict_newdata(features)
    strictConstraints <- 0
    artifactConstraints <- -exp(10*(GHOST_value$response-0.05))
    vagues <- 0
 
    if(SNR_WM_value$response <30){
      vagues <- vagues -10000
      
    }
    if(SNR_GM_value$response <30){
      vagues <- vagues -10000
      
    }
    
    if(SNR_GM_value$response < SNR_WM_value$response){
      vagues <- vagues -10000
    }
    
    return(GWC_value$response + strictConstraints +artifactConstraints + vagues)
    
  }
  
  suggestedSolution <- c(32, 1, 500, 3, 180, 60, 1500, 32, 32, 10, 0)
  GA7 <- ga(type="real-valued", fitness = function(x) mainFitness(x[1], x[2], x[3], x[4], x[5], x[6], x[7], x[8], x[9], x[10], x[11]), 
            lower=c(1, 1, 400, 0, 0, 0, 0, 32, 32, 0, 0), upper = c(64, 64, 2000, 3, 180, 160, 6000, 64, 64, 10, 8 ), 
            popSize=50, maxiter = 100, run=100, pmutation = function(...) ga_pmutation(..., p0=0.6), suggestions = suggestedSolution)
  summary(GA7)
```

```
## ── Genetic Algorithm ─────────────────── 
## 
## GA settings: 
## Type                  =  real-valued 
## Population size       =  50 
## Number of generations =  100 
## Elitism               =  2 
## Crossover probability =  0.8 
## Mutation probability  =  NA 
## Search domain = 
##       x1 x2   x3 x4  x5  x6   x7 x8 x9 x10 x11
## lower  1  1  400  0   0   0    0 32 32   0   0
## upper 64 64 2000  3 180 160 6000 64 64  10   8
## Suggestions = 
##   x1 x2  x3 x4  x5 x6   x7 x8 x9 x10 x11
## 1 32  1 500  3 180 60 1500 32 32  10   0
## 
## GA results: 
## Iterations             = 100 
## Fitness function value = 0.5255402 
## Solution = 
##            x1       x2       x3       x4       x5       x6       x7       x8
## [1,] 63.36984 58.71223 1355.806 1.414466 93.09793 39.34402 4738.881 55.60049
##            x9      x10      x11
## [1,] 43.69423 4.106451 4.650326
```

```
  plot(GA7)
```

```
  ##setting 2
  REALLYHIGHVALUE <- 10000
  fourthFitness <- function(EPI.FACTOR, ETL, READOUT.DURATION, REFERENCE.LINES, REFOCUSSING.ANGLE, TE, TR, numberOfColumns, numberOfRows, MEASUREMENTS, NUMBER.PRESCANS){
    ####because SNR_CSF is the goal
    features <- data.frame(EPI.FACTOR, ETL, READOUT.DURATION, REFERENCE.LINES, REFOCUSSING.ANGLE, TE, TR, numberOfColumns, numberOfRows, MEASUREMENTS, NUMBER.PRESCANS)
    SNR_CSF_value <- MLresult[[4]][[3]]$predict_newdata(features)
    SNR_WM_value <- MLresult[[5]][[3]]$predict_newdata(features)
    TA_value <- MLresult[[12]][[3]]$predict_newdata(features)
    GHOST_value <- MLresult[[7]][[3]]$predict_newdata(features)
    MOTION_value <- MLresult[[10]][[3]]$predict_newdata(features)
    SHARPNESS_value <- MLresult[[8]][[3]]$predict_newdata(features)
    DISTORTION_value <- MLresult[[11]][[3]]$predict_newdata(features)
    GWC_value <- MLresult[[1]][[3]]$predict_newdata(features)
    CWC_value <- MLresult[[2]][[3]]$predict_newdata(features)
    CGC_value <- MLresult[[3]][[3]]$predict_newdata(features)
    strictConstraints <- 0
    artifactConstraints <- -(exp(10*(GHOST_value$response-0.05)) + exp(10*(MOTION_value$response-0.05)) + exp(10*(DISTORTION_value$response -0.05)))
    vagues <- 0
    if(CGC_value$response <10){
      vagues <- vagues - 100
    }
    if(CWC_value$response <10){
      vagues <- vagues - 100
    }
    if(SHARPNESS_value$response <0.85){
      vagues <- vagues -1000
    }
    
    return(SNR_CSF_value$response + strictConstraints +artifactConstraints + vagues)
    
  }
  
  
  GA4 <- ga(type="real-valued", fitness = function(x) fourthFitness(x[1], x[2], x[3], x[4], x[5], x[6], x[7], x[8], x[9], x[10], x[11]), 
            lower=c(0, 0, 400, 0, 0, 0, 0, 32, 32, 0, 0), upper = c(64, 64, 2000, 3, 180, 160, 6000, 64, 64, 10, 8 ), 
            popSize=50, maxiter = 100, run=100)
  summary(GA4)
```

```
## ── Genetic Algorithm ─────────────────── 
## 
## GA settings: 
## Type                  =  real-valued 
## Population size       =  50 
## Number of generations =  100 
## Elitism               =  2 
## Crossover probability =  0.8 
## Mutation probability  =  0.1 
## Search domain = 
##       x1 x2   x3 x4  x5  x6   x7 x8 x9 x10 x11
## lower  0  0  400  0   0   0    0 32 32   0   0
## upper 64 64 2000  3 180 160 6000 64 64  10   8
## 
## GA results: 
## Iterations             = 100 
## Fitness function value = 80.00602 
## Solution = 
##            x1       x2      x3       x4       x5       x6       x7      x8
## [1,] 8.954408 45.90755 1510.83 1.117171 52.41634 96.71443 676.5535 54.2359
##            x9      x10      x11
## [1,] 52.15304 1.854553 5.218513
```

```
  plot(GA4)
```
